# Thio-modified trianglimines, a novel group of chiral macrocyclic compounds of high structural dynamics

**DOI:** 10.1038/s41598-025-85179-9

**Published:** 2025-01-06

**Authors:** Natalia Prusinowska, Agnieszka Czapik, Joanna Szymkowiak, Marcin Kwit

**Affiliations:** 1 Faculty of Chemistry, Adam Mickiewicz University, Uniwersytetu Poznańskiego 8, Poznan, 61 614 Poland; 2 Faculty of Science, Department of Chemistry, University of British Columbia, 2036 Main Mall, Vancouver, BC V6T 1Z1 Canada

**Keywords:** Macrocycle, Chirality, Sulfur-containing compounds, Polyimine, Circular dichroism, X-ray structure, Organic chemistry, Supramolecular chemistry

## Abstract

The embellishing of the macrocycle core with sulfur substituents of varied sterical requirements changes the structural dynamics of chiral, triangular polyimines. Despite their formal high symmetry, these compounds adopt diverse conformations, in which the macrocycle core represents a non-changeable unit. DFT calculations reveal that the mutual arrangement of sulfur-containing substituents is controlled mainly by sterical interactions. The presence of sulfur atoms affects the chiroptical properties of these compounds and causes a red shift of respective absorption bands compared to the basic trianglimine. Unexpectedly, the aromatic fragments attached to the sulfur atom have less impact on ECD spectra, visible only in particular spectral regions. Such a possibility to adapt various conformations is also seen in the crystalline phase; however, a stiff basic unit – the triangular macrocycle core – caused macrocycles’ self-assembly into columnar-like aggregates. In the crystal lattice, around the macrocycle having bulky SCPh_3_ groups, a space filled with solvent is formed; however, the macrocycle’s internal cavity is closed and unavailable for guest molecules. Titration of the solutions of basic SBn-substituted imine and amine macrocycles by AgOTf results in significant changes in the ECD spectra, confirming possible binding interactions between macrocycle and metal cations.

**Supplementary Information:**

The online version contains supplementary material available at 10.1038/s41598-025-85179-9.

## Introduction

Due to their wide-ranging applications, macrocyclic compounds are highly sought-after synthetic targets^1,2^. Unfortunately, the challenge of obtaining cyclic macromolecules with defined molecular weights and shapes persists. On the other hand, the molecule’s shape persistency and the use of reactions based on dynamical bond formation led to obtaining compounds with regular and symmetrical structures resembling geometrical figures^3–5^.

The synthesis of triangular chiral hexaimines (trianglimines) involves the direct formation of macrocycles from vicinal diamine (usually optically pure *trans*-1,2-diaminocyclohexane, DACH, **1**) and aromatic 1,4-dialdehyde^6^. In the simplest case, terephthalaldehyde (**2**) serves this function, and the products’ high, usually quantitative yields are an added advantage^7^. Further functionalization of the macrocycle is achieved by reducing C = N imine bonds and subsequently modifying nitrogen atoms. These modifications lead to the formation of more chemically resistant materials, and the proper *N*-functionalization allows for control over molecular dynamics^8^.

The nature of the π-electronic system and functional groups in the aromatic linkers and control of the conformational liability of a molecule is crucial for applications of the macrocycles. Recently, it has been shown that simple trianglimines and trianglamines enable efficient separation of linear over branched hydrocarbons, constitutional and geometrical isomers, and enantiomers. Trianglamine-containing membranes have turned out to be effective nanofiltration agents that allow for the purification of organic solvents^9–22^.

The particular case of the molecule of properties differs in the solid state and the solution, representing trianglsalen containing two hydroxyl groups in the *para* position of each aromatic linker. In one of the polymorphs, the trianglsalen molecules form a tubular architecture composed of supramolecular segments, each consisting of six macrocycles. These nanoporous, chromogenic crystals can release water even at − 70 °C, which is associated with the crystal’s color change, allowing for the quantification of water uptake^23^.

It has been known that polyaza-macrocycles characterized by different structural properties, containing or not heteroatoms attached to the molecule core, may form metal complexes and metallogels. Zinc(II)- or copper(II)-trianglamine complexes used in asymmetric catalytic reactions and *N*,*N*’-thiourea-derived trianglamine supergelators, forming chiral metallogels with Ag(I), Cu(I), and Cu(II) salts are two distinct examples^24–29^.

Feeling that the chemistry of chiral and shape-persistent macrocyclic polyimines containing heteroatoms attached to the skeleton is still at an early stage of research, we have decided to expand our interest on trianglimine derivatives embellished with sulfur derivatives in each aromatic linker.

In principle, the incorporation of thioether moieties into the trianglimine skeleton would affect the structural preferences of the mother compound. The trianglimine and its reduced congener represent extreme cases of compounds with contrasting structural dynamics. Therefore, incorporating flexible “arms” into the skeleton will represent a missing link connecting rigid trianglimine macrocycle with highly flexible trianglamine. As the macrocycles planned to be synthesized are chiral, the impact of sulfur atoms on the chiroptical properties, namely electronic circular dichroism (ECD), of these compounds is worth interest. ECD spectroscopy is a convenient method that provides structural information regarding the structure of chiral macrocyclic species and their supramolecular assemblies^30,31^. Additionally, ECD spectroscopy may be used to directly observe macrocyclic receptors’ interactions with metal cations.

## Results and discussion

**Scheme 1:**
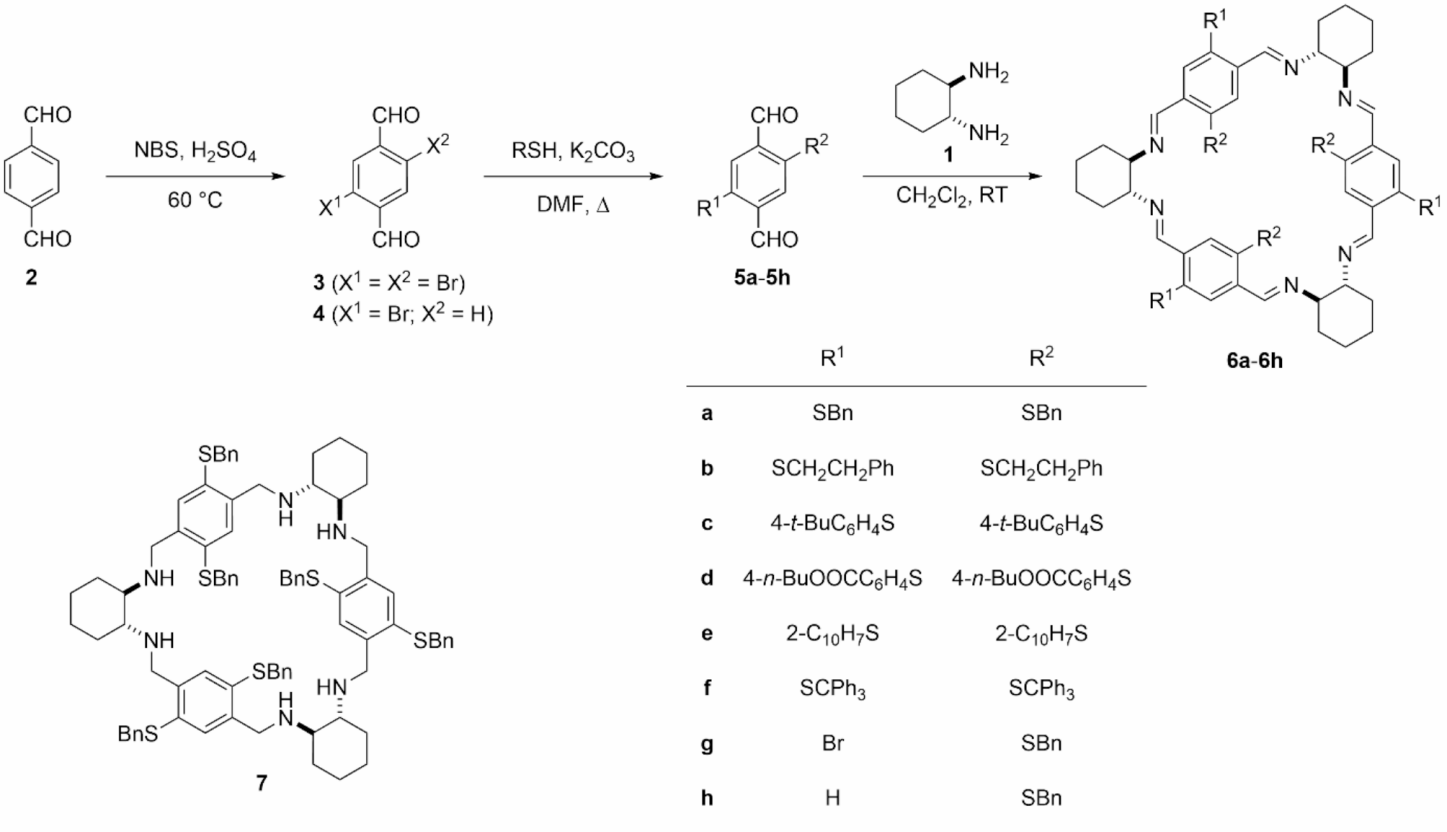
The general synthetic route provides chiral thio-modified macrocycles.

In most cases known so far, the heteroatoms (oxygen-containing groups) are already present in the aromatic unit, and subsequent modifications rely on introducing formyl groups to the molecule skeleton^32–34^. The bromine is an exception, which may be conveniently introduced to the terephthalaldehyde through direct bromination, using *N*-bromosuccinoimide (NBS) in concentrated sulfuric acid. The product of the reaction, namely 2,5-dibromoterephthalaldehyde (**3**, see Scheme 1), is obtained with a high yield. Further transformations are based on well-established palladium-catalyzed reactions that form aryl- or alkynyl-substituted terephthalaldehydes^35^.

Having established a convenient procedure for introducing bromine atoms into the terephthaldehyde skeleton, we used bromination product **3** as the starting point in the synthetic path to obtaining macrocycle compounds (Scheme 1). Subsequent substitution of the bromine atom(s) by thiol will lead to the formation of respective thioethers **5**^36^. Using thiols of the higher molecular mass as nucleophiles yielded respective thioethers, even if the substituent attached to the sulfur atom was as bulky as the trityl group. Some monosubstituted thioether **5 g** was isolated alongside the double-substituted product in the selected case. The thioether **5 h** was synthesized intentionally for comparison purposes, starting from mono-brominated terephthalaldehyde **4**. The latter is either isolated in small amounts as by-products of terephthalaldehyde dibromination or might be synthesized when only half of NBS has been used. Although the thioethers thus obtained are stable, there is one exception – thioether **5f**, which changed the color from yellow to deep red upon standing. It is worth noting that during the synthesis of **5f**, some trityl peroxide was formed, which is barely separable from the aldehyde by crystallization.

**Fig. 1:**
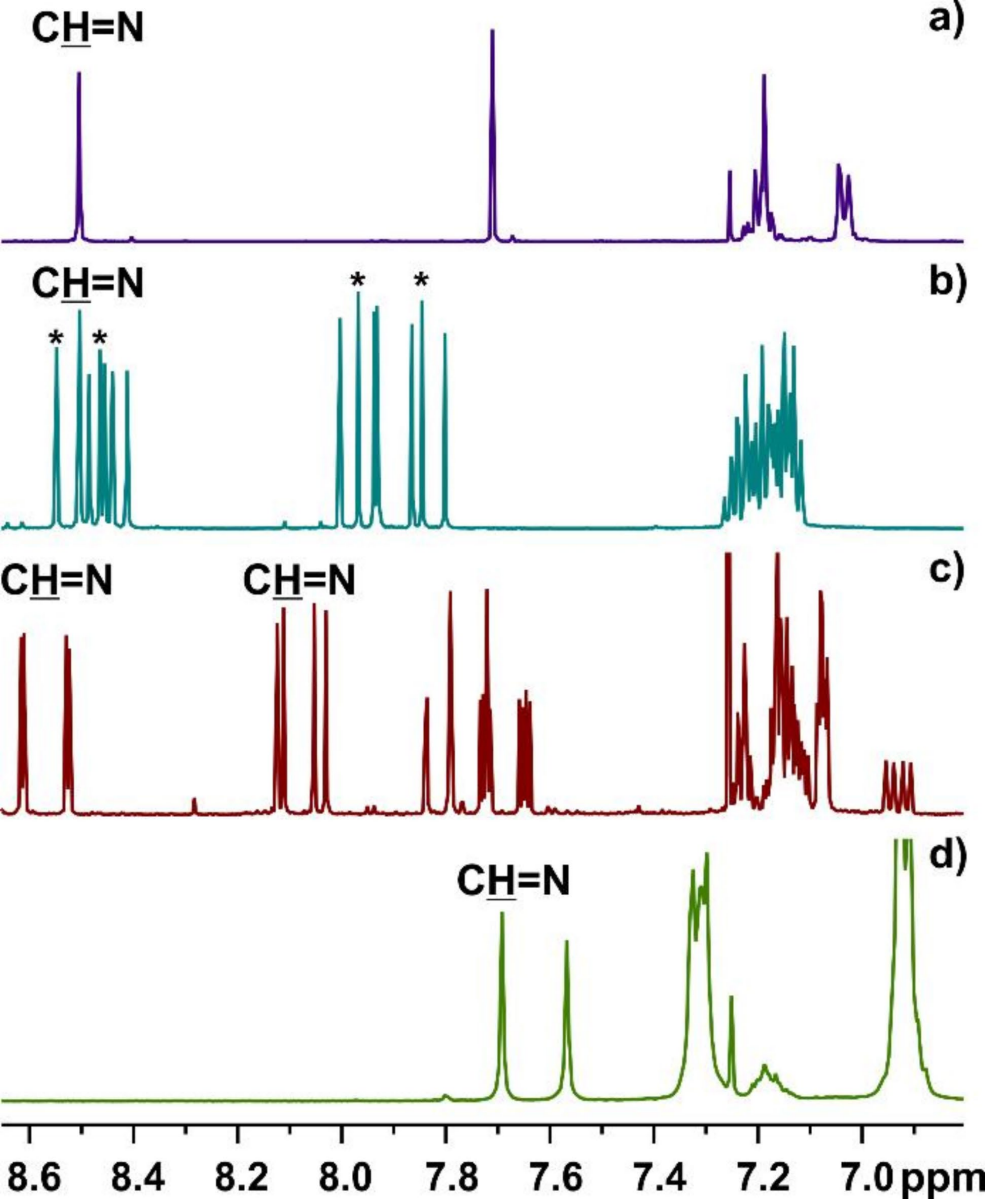
Diagnostic part of ^1^H NMR spectra (CDCl_3_) of (**a**) symmetrical macrocycle **6a**, a mixture of symmetrical and non-symmetrical macrocycles: (**b**) **6 g** and (**c**) **6 h**, and (**d**) sterically crowded macrocycle **6f**. Asterisks indicate signals of protons originating from the *C*_3_-symmetrical macrocycle **6 g**.

With the semi-products in hands, the macrocycles **6** were synthesized at the next stage through cyclocondensation reactions between equimolar amounts of dialdehyde and (*R*,*R*)-**1**^7^. The cyclic structure of a given product was confirmed by NMR spectroscopy and mass spectra. The number of peaks on the NMR spectra directly reflects the symmetry of the given compound (see Fig. 1a-c). For macrocycles obtained from symmetrical, double-substituted aldehydes, the highest available symmetry is *D*_3_. On the other hand, when non-symmetrical aldehydes **5 g** and **5 h** were used for reactions, a mixture of *C*_3_-symmetrical and non-symmetrical (trivial, *C*_1_-symmetrical) products **6 g** and **6 h**, respectively, was obtained. The proportion of the products that differ in symmetry has not been altered upon the change of reaction conditions. Additionally, the ^1^H NMR spectra measured for **6 g** during two weeks and in the time intervals have shown no changes in the composition of the mixture. In both cases, the non-symmetrical product amount was three times as big as the symmetrical one, as indicated by the integration of diagnostic signals originating from imine C*H* = N protons.

While for most macrocyclic compounds discussed here, the diagnostic protons appeared at around 8.5 ppm, and there is one exception. For the particular case of **6 h**, the imine C*H* = N protons appeared in a wide range of chemical shifts. The deshielded signals are observed between 8.7 and 8.5 ppm and originated from C*H* = N imine protons near the heteroatoms. In contrast, more shielded signals (observed between 8.15 and 8.05 ppm) correspond to the resonances of the remaining azomethine protons.

Having neglected the symmetry, the reactions mostly provided chemically homogeneous products. Some additional, very small peaks on ^1^H NMR spectra were visible for macrocycle **6a**. Although the macrocyclic product exhibited trimeric and triangular structure, resulting from [3 + 3] cyclocondensation reaction, additional peaks originating from expanded [4 + 4] congener were also observed on the mass spectra measured for crude reaction mixture. These peaks have vanished after purification. It should be noted that the temperature-dependent ^1^H NMR measurements did not exhibit any spectacular changes. Apart from minimal shifts in the downfield region, the respective pics remain sharp.

The particular case represents a macrocycle **6f** embellished with six *S*-trityl groups. The presence of bulky substituents in the aldehyde skeleton may cause some problems with the macrocyclization reaction. However, the reaction proceeded smoothly, providing a triangular macrocycle with an almost quantitative yield. Significant crowding within the macrocycle ring has not been revealed in the shape of the ^1^H NMR spectrum. The broadening of the signals is visible mainly for aliphatic protons. Unexpectedly, the presence of trityl groups caused a shielding effect for azomethine protons, which appeared at 7.69 ppm (see Fig. 1d). This is apparently due to the possible perpendicular (or close to it) orientation of one of the phenyl rings from the neighboring trityl group to the azomethine proton. The same trend is observed in the ^1^H NMR spectrum of parent dialdehyde **5f**, where the C*H*O proton appeared at 9.5 ppm.

As mentioned earlier, the presence of STr moieties has caused problems with the stability of the compounds, which is revealed by naked-eye visible changes in the color of the samples, both aldehyde and macrocycle. Protecting the product against moisture and direct light slows the decomposition process but does not eliminate it.

The post-synthetic modification of the model macrocycle **6a**, which involved reducing C = N imine bonds, proceeded without complications and led to the quantitative formation of trianglamine **7**^8^.

### X-ray structure of macrocycles **6b**, **6f**, and **6 g**

**Fig. 2:**
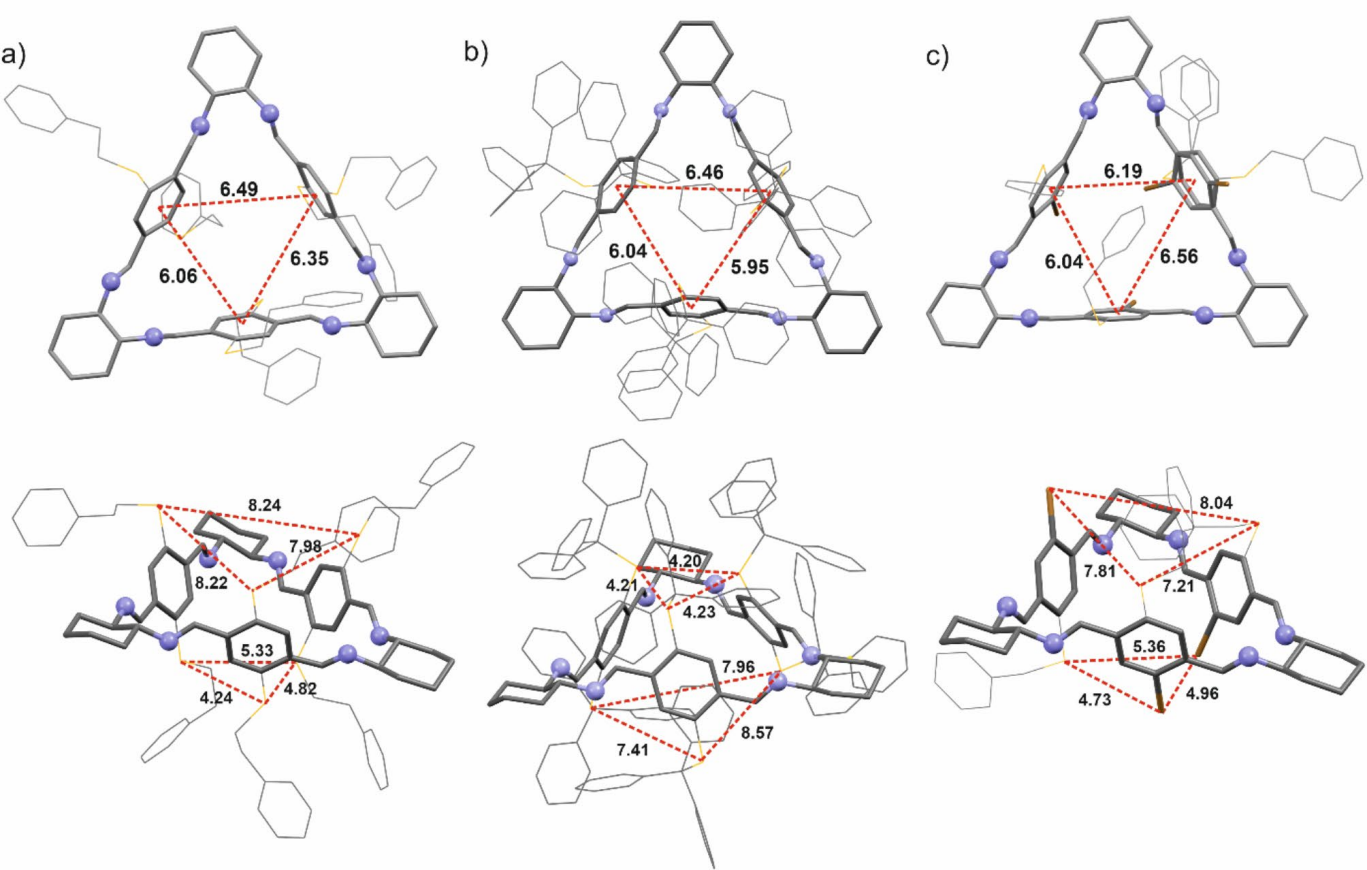
Top and side views of the molecular structure of macrocycles: (**a**) **6b**, (**b**) **6f**, and (**c**) **6 g**. Hydrogen atoms were omitted for clarity. The proper distances are shown as dashed lines and are given in Å.

Due to their poor shape complementarity, we were fortunate to obtain crystals suitable for X-ray study only for few examples. These included symmetrical macrocycles **6b** and **6f** and the macrocycle **6 g** (Fig. 2).

Generally, the triangular backbone of macrocycles **6b**, **6f**, and **6 g** display similar conformations in the crystal structure. The C*-N = C-C_*ipso*_ torsion angles have similar values that concentrate around 180°. Furthermore, in all cases, the molecule assumes a bowl-like configuration.

Compound **6b** crystallizes in monoclinic space group *P*2_1_ with one macrocycle molecule in an asymmetric unit. The molecule has the *D*_3_ symmetry, but due to the conformational lability of the substituents, it assumes the *C*_1_ symmetry in the crystal lattice. The molecule takes a bowl-like shape with an upper rim diameter of approximately 8.15 Å and a lower rim of 4.80 Å. The position of S atoms of substituents determines both rims. The size of the internal gap is determined by the distances between the centroids of the phenyl rings in the macrocycle skeleton, and they range from 6.06 to 6.49 Å. However, it should be emphasized that the macrocycle molecules fill the space, and no gaps are available for the solvent molecules in the crystal.

As it has been emphasized earlier, while the triangular skeleton of the molecule is relatively rigid, the carbon chains of the substituents are flexible. This means that their conformation can change and adapt to the available space in the crystal lattice. As a result, the substituents on the triangular core of the molecule are disordered. Notably, despite the disorder that results from the flexibility of the aliphatic linker in the substituent, the bowl in the upper part remains open. A phenyl ring in the neighboring molecule’s lower part is inserted into the macrocycle cavity in the crystal structure. The phenyl ring of the substituent is arranged almost parallel to the aromatic system of the macrocycle skeleton (the angle between the rings is about 5.3°), and a system is stabilized by the π∙∙∙π interaction (the distance between the planes of the aromatic rings is about 3.5 Å) (Figure S129 in SI). In the crystal structure, supramolecular columns are formed, which are additionally stabilized by C-H∙∙∙π and C-H∙∙∙S interactions.

The three-dimensional structure is stabilized by weak interactions involving aromatic systems (C-H∙∙∙π interactions) and van der Waals interactions.

Compound **6f** crystallizes in the monoclinic system in the *P*2_1_ space group, comprising two macrocyclic molecules and solvents: hexane, dichloromethane, and water. The host-guests ratio in the crystal structure is 1:1:1:3. The guest molecules are situated within the extrinsic space – in a void with an elongated shape created by the macrocyclic host molecules. Therefore, the disorder in the solvent-accessible space was quantified using a solvent mask instruction, as implemented in Olex2 software^37^, resulting in the identification of 256 electrons within a volume of 1930 Å³ in a void per unit cell. This finding is consistent with one hexane, one dichloromethane, and three water molecules per asymmetric unit, accounting for 244 electrons per unit cell.

In the molecular structure of **6f**, the C*-N = C-C_ipso_ torsion angles exhibit similar values, approximately 180°. The triangular skeleton assumes a bowl-like shape. The inner cavity size of the molecular bowl is determined by the rigid building blocks that comprise the macrocyclic skeleton. The distances between the three centroids of the phenyl rings that define the cavity size range from 6 to 6.5 Å. Macrocycle **6f** assumes a rigid pillararene-like conformation with three -STr substitutes at aromatic linkers on both sides of the skeleton. The presence of bulky -STr groups precludes the rotation of the aromatic linkers, compelling the macrocycle to assume a conformation that optimizes the size of the cavity. Consequently, in **6f**, the mean cavity diameter is 6.5 Å, with a depth of 18 Å. Notably, guest molecules do not penetrate into the macrocycle cavity despite the bulky and partially open structure.

Although macrocycle **6f** formally exhibits *D*_3_ symmetry, it remains in *C*_1_ symmetry in the solid state. It should be noted that not all of the -STr substituents present in the crystalline structure exhibit the same arrangement. Four groups are oriented towards the center of the cavity, one group is oriented towards the exterior, and another is partially twisted, resulting in a partially open macrocycle structure with three distinct arrangements of aromatic building blocks. The configuration of the aromatic linkers within the macrocycle skeleton can be described by *pseudo*torsion angles, specifically C(Tr)-S-(Ar)-S-C(Tr). The indicated angles have values of 163°, 16°, and 91°, respectively. Additionally, each trityl group attached to the linker exhibits a distinct helicity (Figure S130 and S131 in SI). This observed ‘asymmetry’ may be attributed to the limited potential for intermolecular interactions (the ‘asymmetry’ of the molecule can be reinforced by creating supramolecular interactions).

**Fig. 3:**
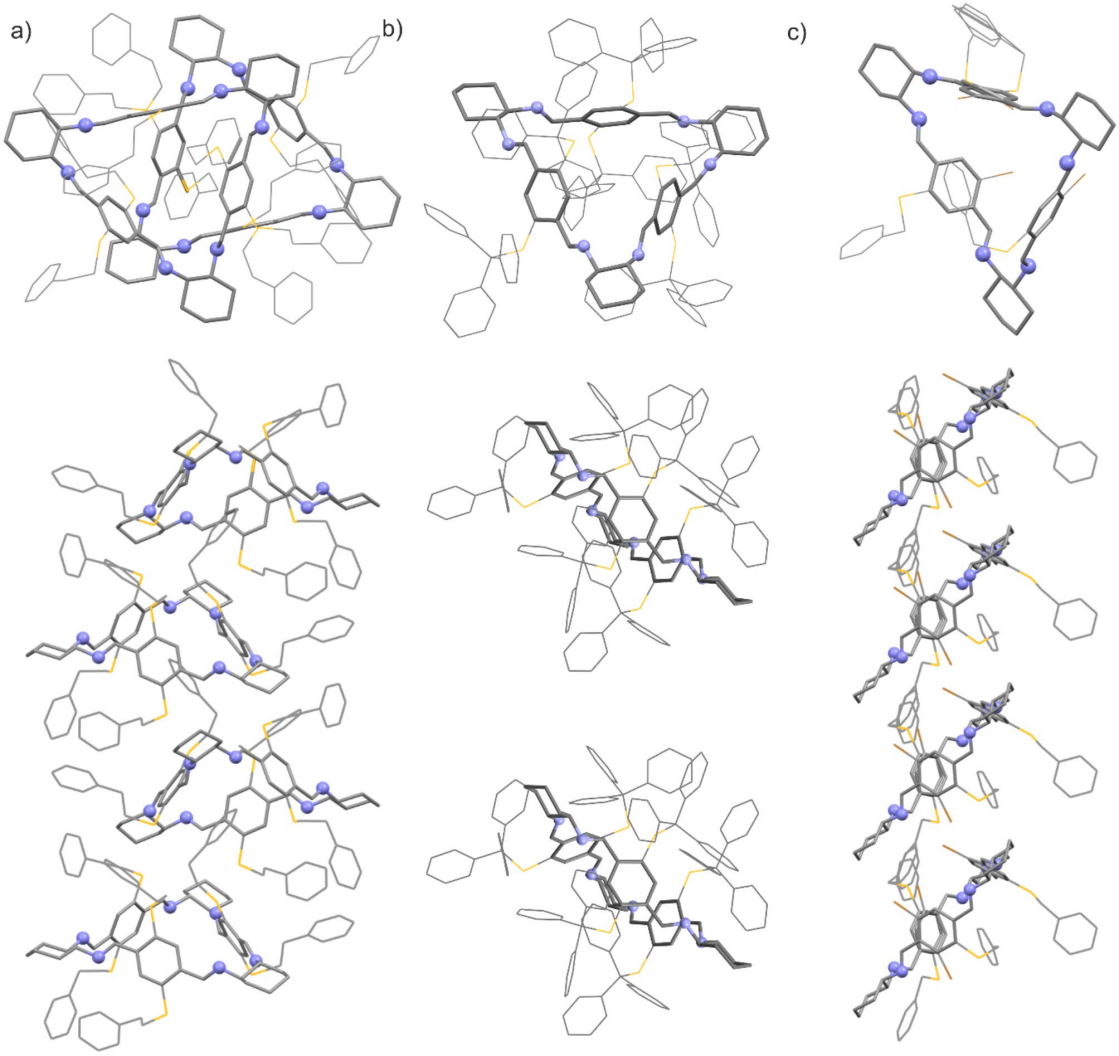
Supramolecular columns in crystals of compound (top and side view) (**a**) **6b**, (**b**) **6f** and (**c**) **6 g**.

Imine macrocycles in the **6f** crystalline form establish a supramolecular network through the utilization of weak C-H∙∙∙π and C-H∙∙∙S intermolecular interactions facilitated by the -STr groups of neighboring molecules. In the **6f** crystal structure, the macrocycles self-assemble into columnar-like aggregates, and the angle of inclination of the stack axis to the plane of the macrocycle ring is approximately 45°. The distinguished columnar motif is also observed in simple trianglimines and trianglsalenes crystal structures (See Fig. 3)^34,38^.

Compound **6 g** crystallizes in triclinic space group *P*1 with one macrocycle molecule. The crystal structure contains some disordered solvent molecules caged in intermolecular holes. A solvent mask was calculated, and 46 electrons were found in a volume of 203 Å^3^ in 1 void per unit cell. This is consistent with diethyl ether (C_4_H_10_O) per asymmetric unit, which accounts for 42 electrons per unit cell. Therefore, the estimated host-guest ratio in the crystal structure of compound **6 g** is 1:1.

The macrocycle molecule shows *C*_1_ symmetry, with one of the three bromine atoms on the opposite side of the ring plane. However, as shown in the structural analysis, the molecule is disordered and, alternatively, takes a conformation where all three Br atoms are situated on the same side of the molecule (the refined occupancy factor is 0.19). As in macrocycles **6b** and **6f** crystals, the molecule takes a bowl-like shape. The rims are determined by the position of S and Br atoms of substituent, and their lengths vary depending on the arrangement of the substituents on the triangular backbone. For a molecule with two bromine atoms at the bottom of the bowl, the upper rim diameter is approximately 7.68 Å, and the lower rim is 5.02 Å. For the molecule where all three bromine atoms are located on the lower part of the bowl, the upper rim diameter is approximately 8.18 Å, and the lower rim is 4.59 Å. The size of the internal gap is determined by the distances between the centroids of the phenyl rings in the macrocycle skeleton, and they range from 6.04 to 6.59 Å.

As it has been mentioned, the crystal contains some solvent but is located in the spaces between the molecules. The top of the molecular bowl is covered by a benzyl substituent, which makes its interior inaccessible to guest molecules. In addition, the aromatic ring is slightly inserted into the cavity of the macrocycle, and C-H∙∙∙π interactions stabilize its position (Figure S132 in SI).

In the crystal structure, the molecules are arranged in columns, and the triangular skeleton of the molecule is significantly inclined to the column axis (the nitrogen atoms of diaminocyclohexane determined the plane, and the angle between the column axis and the plane normal is about 48°). The structure of the column is stabilized by interactions of the bromine atom with the imine N-atom (Br∙∙∙N 3.3 Å) as well as C-H∙∙∙Br interactions (distances H∙∙∙Br are 2.94 Å and 2.99 Å). The molecules are stacked so that the cyclohexane ring of the next macrocycle covers the lower part of the bowl of one of them. The three-dimensional structure of the crystal is stabilized by C-H∙∙∙N interaction as well as C-H∙∙∙π interactions between adjacent stacks of molecules.

### The structure and chiroptical properties of macrocycles from ECD spectroscopy and DFT calculations

As the compounds in hands are chiral and optically active, we have taken advantage of determining their chiroptical properties, emphasizing ECD as the method of choice^39^. The ECD spectroscopy, supported by theoretical calculations, would shed light on the structure in the solution of these compounds and, therefore, may be considered complementary to X-ray crystallography^40,41^. A feature that significantly facilitates the experimental and theoretical analyses is the solubility of these compounds in non-polar solvents, such as cyclohexane. Thus, routine calculations performed for isolated molecules in the gas phase will reflect experimental conditions.

**Fig. 4:**
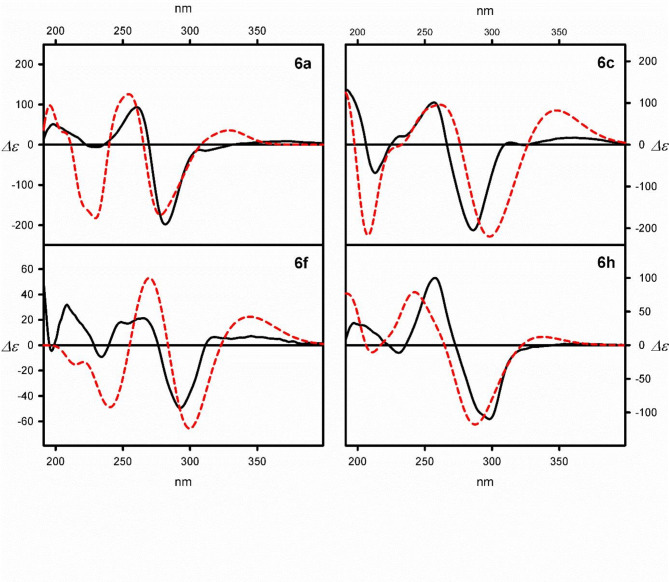
Examples of ECD spectra of macrocycles **6a**, **6c**, **6f**, and **6 h**: experimental, measured in cyclohexane (black lines) and calculated at the TD-DFT level (red dashed lines). The calculated ECD spectra have been Boltzmann averaged. Wavelengths have been corrected to match the experimental UV maxima (Δε values are given in mol^−1^ cm^−1^ dm^3^ units).

An analysis of experimental ECD data led to the formulation of some general conclusions. Regardless of the substitution patterns, the measured CD spectra are similar (see Fig. 4 for representative examples and SI for the remaining data).

The lowest-energy positive signed Cotton effects (C.es) in ECD spectra are visible at around 370 nm. This band is associated with a low-intense UV band, which appears in the 380 − 360 nm energy range. For most of the examined macrocyclic imines, the magnitude of the lowest-energy C.e. oscillates around *Δε* = 10 mol^− 1^ cm^− 1^ dm^3^. The exception is **6 h**, for which this particular band is barely visible.

The following ECD bands form a negative exciton-like couplet. The first negative C.e. is visible at around 280 nm, whereas the second, the positive one, appears at around 260 nm. The exciton effect is related to the UV band of middle intensity, which is seen in the UV spectrum of a given compound at around 275 nm. The amplitude (*A*) of these exciton couplets varied, depending on the type of substituent attached to the sulfur atom. The lowest amplitude was found for the most crowded macrocycle **6f** (*A* = -70), whereas macrocycle **6d** of A = -400 is placed on the opposite pole.

Interestingly, the highest intensity UV band, which appears at the higher-energy region of the spectrum (usually at around 190 nm), does not reflect in high magnitude C.es in the ECD spectrum of a given compound, as one can expect. Usually, the negative/positive C.es sequence is repeated in the higher-energy region of the spectrum. The magnitude of the positive C.e., which appears in this spectral region, is up to *Δε* = 100 mol^− 1^ cm^− 1^ dm^3^, while a negative one rarely exceeds *Δε* = -25 mol^− 1^ cm^− 1^ dm^3^.

One feature of these compounds is their indifference to solvent polarity. For example, the ECD spectra measured for model compound **6a** have shown no significant alternations when the solvent polarity was changed from non-polar cyclohexane through dichloromethane to highly polar acetonitrile. Even using methanol as a co-solvent did not alter the spectra’ shape or the magnitudes of respective bands. An exception is STr-substituted macrocycle **6f**, where the magnitude of respective Cotton effects increases with solvent polarity.

In contrast to the chiroptical properties of parent imine **6a**, the ECD spectrum of its reduced congener **7** is characterized by a shallow magnitude of C.es. The first low-energy ECD band of the negative sign appeared at 275 nm and is associated with a middle-intense UV band visible in the same spectral region. The following C.es form sequence +/-/+. The increase of the solvent polarity is reflected in the rise in the magnitude of respective C.es, and the highest values are found in the ECD spectrum measured in dichloromethane containing 20% methanol. This suggests that the hydrogen bonds may significantly stabilize the structure, and these non-covalent interactions may be broken or weakened in a more polar solvent.

It is worth noting that the ECD spectra of the imines **6a**-**6 h** are entirely different in shape from those of their oxygen-containing congeners^42^.

To determine the structural dynamics of the macrocyclic compounds and the chiroptical properties of the compounds under study, we have performed extensive calculations at the DFT level of their structure and UV/ECD spectra. Before these, we chose representative examples for these calculations. This set includes symmetrical and non-symmetrical macrocycles **6a**, **6c**, **6 h**, **6 g**, and the most challenging example, **6f**. Details regarding the calculation scheme and the method used are provided in Experimental Section and the SI.

Without going into the structure details, one significant trend is visible when one analyzes the results of calculations – the number of thermally available structures (i.e., these having relative energies within the range 0 ÷ 2 kcal mol^− 1^) varied depending on the functional used. The highest number of stable conformers was found for structures calculated using the B3LYP functional^43,44^. However, considering empirical dispersion correction led to calculating only a few stable structures^45^. The results obtained by the pure M06L functional were placed between^46^, albeit closer to, B3LYP-GD3BJ, at least in terms of the number of stable structures.

**Fig. 5:**
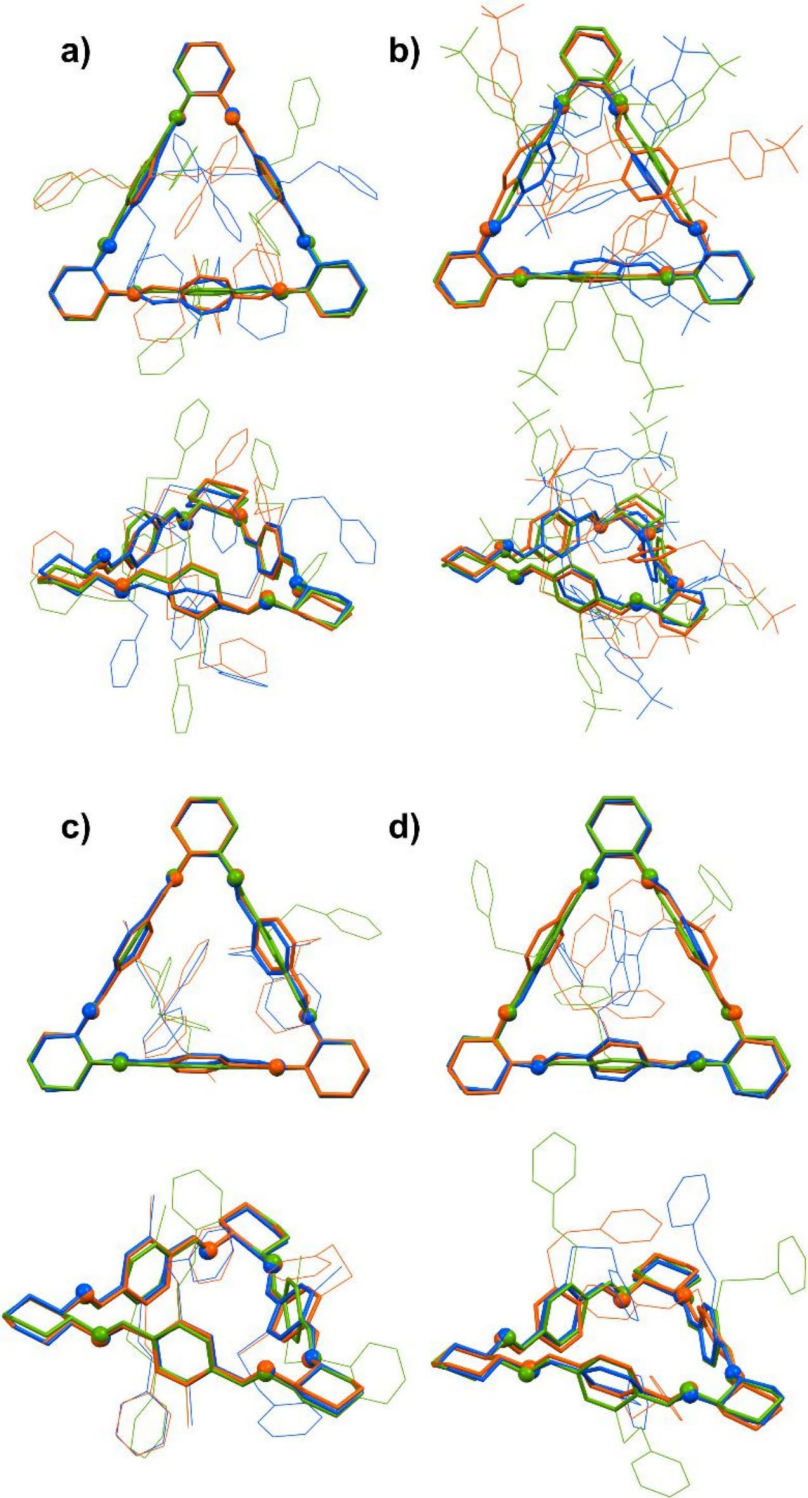
Top and side view on overlays of the structures of the lowest energy conformers of macrocycles (**a**) **6a**; (**b**) **6c**; (**c**) **6 g**, and (**d**) **6 h**. Green-colored structures were optimized using the B3LYP functional, orange-colored structures were optimized using the B3LYP-GD3BJ method, and blue-colored structures were optimized using the M06L functional. Hydrogen atoms were omitted for clarity.

The “nature” of a method is reflected in the structure of the stable conformers, i.e., the more non-covalent interactions are considered, the more “compact” the structure becomes. The latter may be understood as the tendency to maximize the interactions and, in this way, the mutual approach of these structurally labile aromatic fragments attached directly or through a carbon atom to a sulfur atom. Conversely, in the case of a functional such as B3LYP, the structure of the given conformer is mainly controlled by sterical interactions. Figure 5 compares structures of the lowest energy conformers of macrocycles **6a**, **6c**, **6 g**, and **6 h**, calculated using different methods.

It is worth adding that the calculated structures only partially reflect the structures determined experimentally. While the macrocycle core remains (almost) unchanged, the differences between experimental and calculated structures rely on the conformation of backbones, as one may expect.

Since there is no direct possibility to asses which method provides the correct results, we have done it by the comparison of experimental ECD spectra with those calculated for low-energy conformers and Boltzmann averaged (see Fig. 4 and SI).

Unexpectedly, in most cases, the best agreement between experimental and theoretical results was seen when the ECD spectra were calculated for geometries previously optimized using B3LYP hybrid functional. On the opposite pole are the results obtained for geometries optimized when the B3LYP-GD3BJ functional was used. The pure M06L functional shows its advantage over others for two extremal cases, the simplest mono-substituted **6 h** macrocycle and the most complex molecular system **6f**. All the methods gave similar results among the functionals used for excited state calculations. Therefore, we limited the discussion to the ECD spectra calculated using CAM-B3LYP functional^47^.

The direct inspection of the obtained data led to the conclusion that the conformations of the backbones have only a limited impact on the low-energy region of the ECD spectra. The comparison of the ECD data obtained after Boltzmann averaging of spectra calculated for individual conformers with those calculated for the lowest energy structures show their almost perfect overlay (see SI). Therefore, we have limited the further structural discussion to the lowest energy conformers of the given compounds.

A set of 5 torsion angles can conveniently describe each optimized conformer. The first three, α = N-C*-C*-N, β = H-C*-N = C, and γ = N = C-C-C(X), are related to the conformation of the macrocycle core. In contrast, the remaining two, φ = (H)C-C-X-C and χ = C-X-C-C describe the conformation of the substituent concerning the macrocycle core. In the case of **6f**, the angles ω = S-C-C_*ipso*_-C_*ortho*_ determine conformation, therefore *P* (90°>ω > 0°) or *M* (-90°<ω < 0°) helicity, of the trityl groups. The values of the α, β, γ, φ, χ, and ω angles, found for the lowest energy conformers of **6a**, **6c**, **6f**, **6 g**, and **6 h** macrocycles, have been juxtaposed in Table 1.

**Table 1:** The values of the α, β, γ, φ, and χ angles (in degrees) found for the calculated at the DFT level the lowest energy conformers of **6a**, **6c**, **6f**, **6 g**, and **6 h** macrocycles. The number of the lowest energy conformers and the percentage population estimated based on ΔΔG energies are in parentheses.

Comp.^[a]^	α	β	γ	φ	χ
**6a**^[b]^ (91, 33%)	– 64	5	179	17	170
		0	– 172	– 81	– 71
	– 63	7	179	– 79	71
		5	178	100	171
	– 62	6	– 178	93	176
	– 64	3	177	– 80	– 71
**6c**^[c]^ (26, 23%)	– 65	1	– 177	0	84
		4	– 172	– 109	11
	– 64	4	– 172	73	11
		1	– 177	0	84
	– 66	1	– 172	– 117	33
		1	– 173	– 116	32
**6f**^[d]^ (23, 73%)	– 62	– 17	171	– 92	58^[e]^
		18	– 160	67	44^[f]^
	– 62	– 12	176	– 86	65^[g]^
		3	– 159	– 84	– 55^[h]^
	– 58	– 15	– 168	– 83	– 51^[i]^
		0	– 161	– 15	– 58^[j]^
**6 g**^[b]^ (12, 22%)	– – 64	3	179		
		2	– 172	– 104	65
	– 64	6	– 178		
		4	– 178	83	– 177
	– 64	4	– 178	81	– 178
		5	– 177		
**6 h**^[k]^ (62, 59%)	– 67	– 20	170	11	169
		29	– 163		
	– 59	– 7	– 175		
		18	159	111	– 48
	– 58	10	169		
		2	– 167	16	167

^[a]^Conformers are numbered according to their appearance during conformational search.

^[b]^Optimized at the B3LYP/6-311G(d, p) level.

^[c]^Optimized at the B3LYP/6-31G(d, p) level.

^[d]^Optimized at the B3LYP/6-31G(d) level.

^[e]^ω = 33, − 86, − 21; helicity: *PMM*.

^[f]^ω = 38, − 21, 71; helicity: *PMP*.

^[g]^ω = 29, 81, 50; helicity: *PPP*.

^[h]^ω = 30, 50, 76; helicity: *PPP*.

^[i]^ω = 80, 48, 26; helicity: *PPP*.

^[j]^ω = − 58, − 54, 5; helicity: *PMP*.

^[k]^Optimized at the M06L/6-311G(d, p) level.

Despite the substitution pattern within the macrocycle core, the values adopted by the first three angles may be considered constant. For example, only for the lowest energy conformer of the **6 h** macrocycle, the α angles deviated by ± 6° from the preferred value of -64°.

In each calculated structure of the macrocycles under study, the imine proton is *synperiplanar* to the C*H proton — the values of β angle range from − 17° to 18°. Without exception, the γ angle is *antiperiplanar*.

The lowest energy conformer of the simplest macrocycle, **6 h**, is characterized by the most distorted structure, which may result from deficiencies in the calculation method.

The remaining torsion angles, which indicate the structure of a given macrocycle, may adopt different, mutually non-correlated values. This is especially seen for the χ angles. However, one should consider that this angle determines the mutual orientation of different π-electronic systems. In each arm of the macrocycle, one π-electronic system is relatively constant. It constitutes a part of the macrocycle core, whereas the second is directly, or through the carbon atom, attached to the sulfur atom. Conformation of the φ angle is mainly affected by possible electron conjunction between the aromatic π-electron system and *n* orbital at the sulfur atom.

The orientation of aromatic fragments, related to the χ angle, may be treated as a compromise between sterical repulsions and attractive dispersion interactions, where the first ones seem more critical. For example, in the lowest-energy conformer of the basic macrocycle, **6a**, three of the six benzyl groups are placed outside the macrocycle cavity, one is a prolongation of the rim (the aromatic parts are perpendicular to each other), and the remaining two close the macrocycle cavity. In the latter cases, the aromatic parts of the macrocycle and the benzyl groups are oriented parallelly.

When the possibility to take different conformations is limited, as in the case of the lowest energy conformer of **6c**, none of the aromatic substituents can be oriented neither parallelly nor perpendicular to the mean macrocycle plane. Instead, four of the six aromatic units are placed outside the macrocycle, whereas the remaining two form an umbrella over cyclohexane rings.

The conformational behavior of the structurally most complex macrocycle **6f** is an exception. First, only few stable conformers were found by computational methods, regardless of the functional used. Secondly, the calculated conformers are close to this found experimentally in the crystal (*vide infra*), suggesting that intramolecular interactions control the molecule’s gas and the crystalline phase structure. Thirdly, the calculated average length of the C_alk_-S bond is more significant than one can expect and is 1.9 Å, which suggests that the trityl groups are weakly bound to the rest of the molecule and justify the intense red color of the compound sample.

In the lowest energy conformer of **6f**, four STr groups are placed outside the macrocycle, corresponding to the values of φ angles oscillating around − 90°. One STr group obscures the macrocycle cavity (the φ angle is 67°), and the remaining one is placed over one of the macrocycle rims – the φ angle is *synperiplanar*. Such a molecule conformation will prevent the mutual interpenetration of macrocycles in the solid state and the penetration of the guest molecules into the macrocycle cavity. In the previous study, we have shown that trityl groups, when in chiral surroundings, can adapt their conformation to the structure of the permanently chiral inducer. In the case of **6f**, the trityl groups tame many helicities, and there is no direct correlation between the helicity of a given trityl group and the absolute configuration of the nearest stereogenic center. Therefore, the conformations of trityl groups result rather from their mutual fitting than from chirality transmission.

**Fig. 6:**
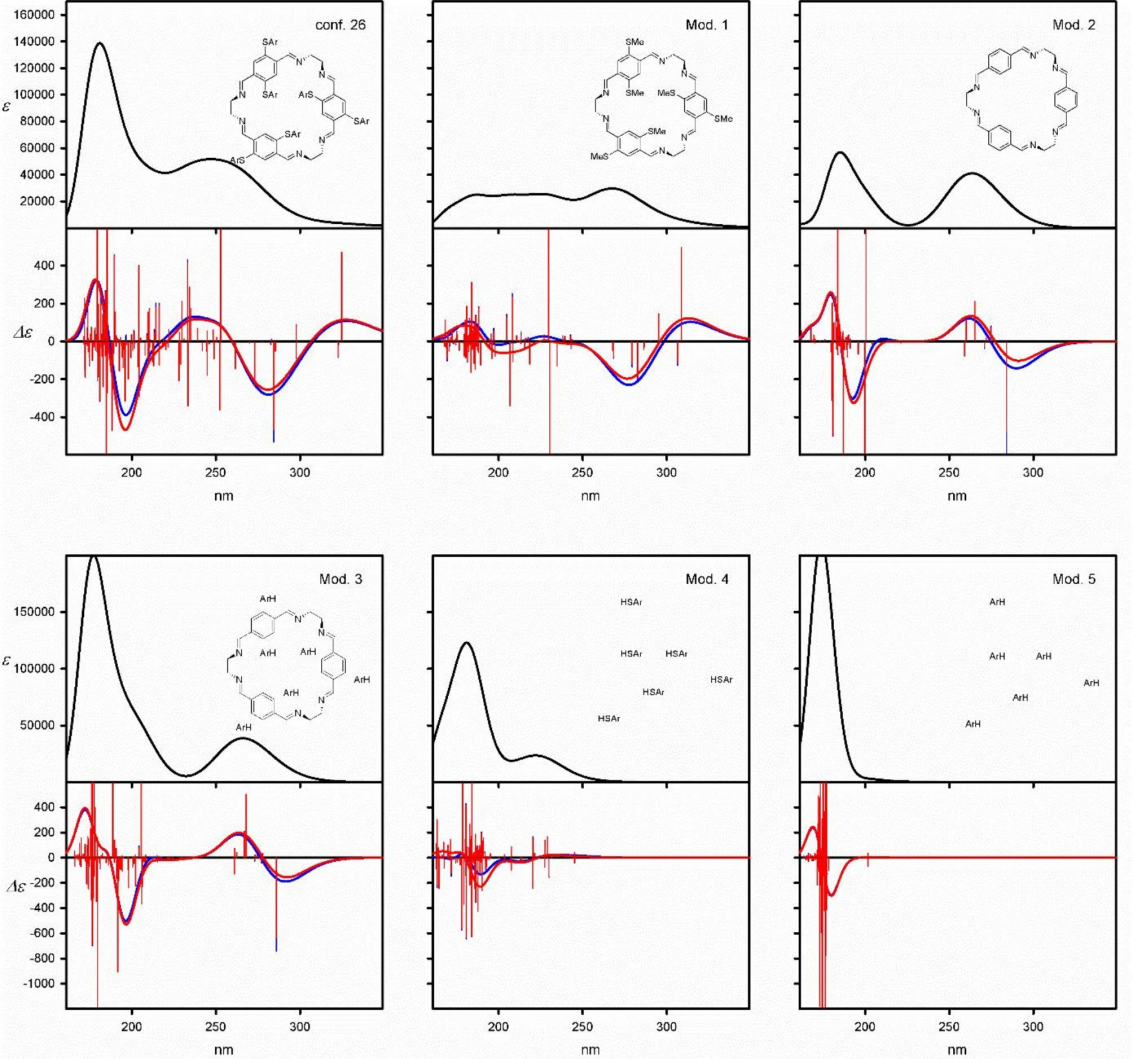
UV (upper panels) and ECD (lower panels) spectra calculated at the TD-CAM-B3LYP/6-311G(d, p) level for the model compounds originated from the lowest energy conformer of macrocycle **6c**. Wavelengths have not been corrected. The vertical bars represent calculated rotatory strengths, and the *ε* and *Δε* values are given in mol^−1^ cm^−1^ dm^3^ units.

The direct comparison of the ECD spectra, calculated for each thermally available conformer of a given compound, has shown significant similarity in the long-wavelength spectral regions and a considerable variety in the higher-energy region of the spectra. Thus, the substituents’ impact on these compounds’ spectral properties relies somewhat on the red shift of the excitation energies, compared to the basic trianglimine, subsequently on the significant change of the spectra shape. We have conducted a series of calculations for model molecules to confirm this working hypothesis. The models (Mod. 1-Mod. 6) have constituted parts of the lowest-energy conformers of macrocycles **6a** and **6c** (see Fig. 6 and SI).

At first glance, the presence of sulfur atoms is responsible for the appearance of the positive low-energy Cotton effects. The aromatic substituents attached to the sulfur atoms are sources of positive C.es appeared at around 250 nm. Noticeably, when the model system (Mod. 3) is deprived of the sulfur atoms, the calculated ECD spectrum is almost the same as the ECD spectrum calculated for the macrocycle skeleton (Mod. 2). This confirms the crucial role of the heteroatom in generating C.es.

The effect of aromatic substituent on ECD spectra of model systems Mod. 4 and Mod. 5 is visible in the higher-energy region, whether or not sulfur is present in the system. Unfortunately, this particular spectral region is rather difficult to interpret as the observed bands are superpositions of effects originating from the entire macrocycle core, interactions between aromatic substituents and the macrocycle core, and sole interactions between aromatic substituents.

**Fig. 7:**
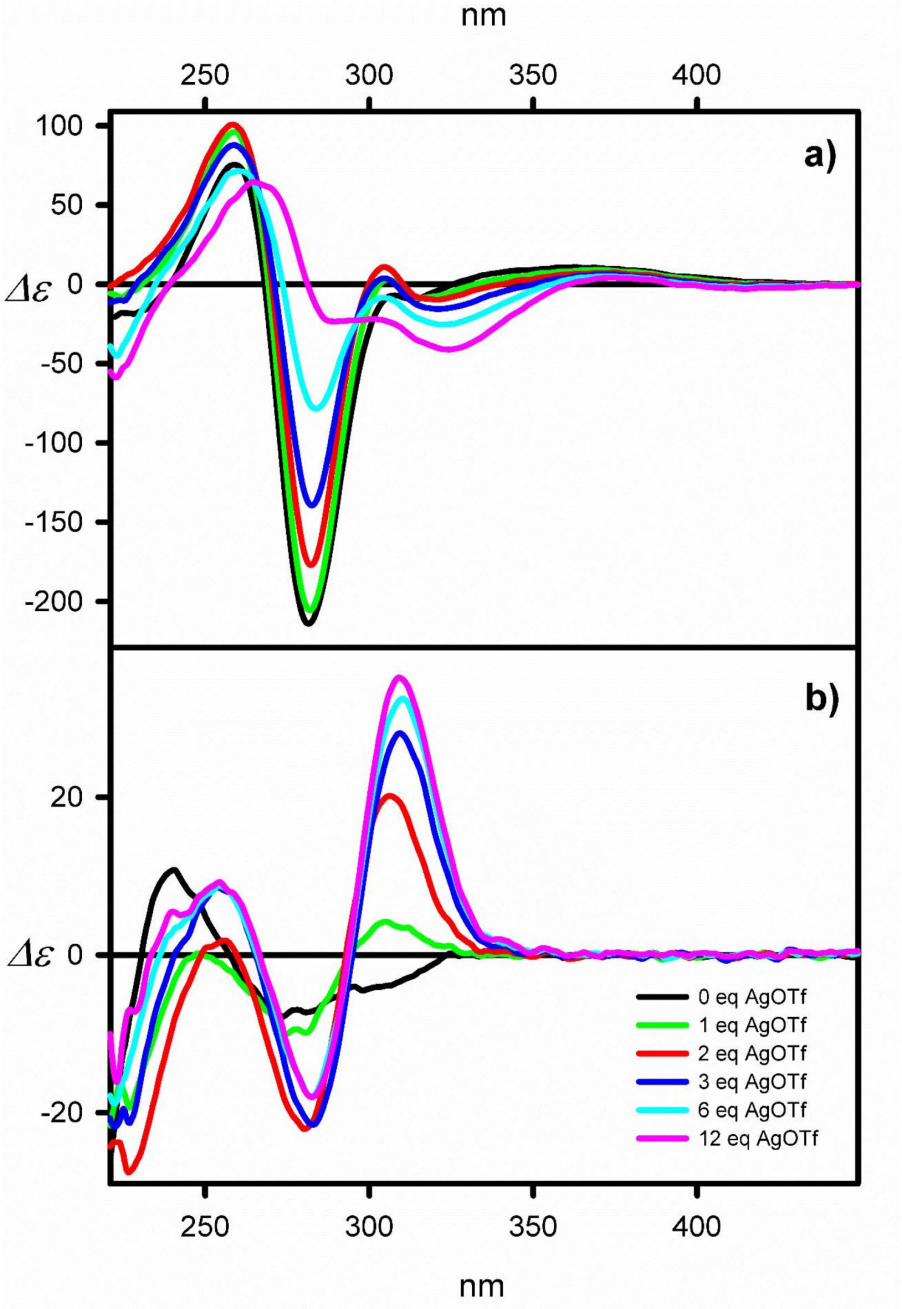
ECD spectra of (**a**) **6a** and (**b**) **7**, measured in dichloromethane-methanol (4:1 v/v) during titration of the macrocycle by AgOTf (*Δε* values are given in mol^−1^ cm^−1^ dm^3^ units).

At the final stage of the study, we checked the possibility of in situ forming the complexes between basic imine **6a**, its reducer congener **7**, and various cations (See Fig. 7 and SI).

Among the cations used, the titration of imine **6a** and amine **7** with silver (I) and copper(II) cations changed the ECD spectra of the parent macrocyclic compounds. Whereas for **6a**, the changes were limited to decreasing the amplitude of C.es; even one equivalent of Ag(I) or Cu(II) caused a significant change in the shape of the ECD spectrum of the macrocycle **7**. Compared to the ECD spectrum of **7**, the presence of metal cations led to the appearance of new ECD bands in the low-energy region in place of the broad negative ECD band observed between 325 and 250 nm for **7**. The newly-appeared C.es have a +/-/+ sequence. In the presence of Cu(II) cations, the measured absolute magnitudes of C.es are comparable to those observed for free amine, and spectra were noisy. In contrast, titration of the macrocycle **7** by Ag(I) cations led to the appearance of prominent absorption bands and an almost eight-fold increase in their magnitude. The ^1^H NMR spectra measured during titration of **6a** and **7** with the silver salt show significant changes in aliphatic and aromatic regions (see Figures S167, and S168, SI). The diagnostic imine signals in **6a** at first multiplied and then, with an excess of the silver salt, almost wholly blurred. The same situation is observed for amine **7**. Here, the diagnostic signals originate from benzyl -CH_2_- protons. After adding just one equivalent of the silver salt, the signals blurred and appeared, while more equivalents of the silver salt were added as broad peaks. These observations are rather qualitative, as partial gelation of the sample was observed during titration.

## Conclusions

In summary, we have reported the synthesis and structural studies on a new class of trianglimine derivatives. These compounds are readily available through relatively simple synthetic steps, and except for **6f**, these compounds are stable. Despite a simple constitution, the structural analysis of these compounds represents a rather challenging task. Whereas conformation of the macrocycle core remains constant, regardless of the substitution pattern, embellishing the trianglimine with flexible arms significantly increases the structural dynamics. These structural dynamics can only be indirectly established for molecules in solution using computational methods. On the other hand, the structure in the crystal is rarely an accurate equivalent of the structure in the solution.

In the case of compounds discussed here, there is only one example of macrocycle **6f**, of which the solid-state and calculated gas phase structures remain in good agreement. Aromatic substituents at sulfur atoms are associated with the danger of computational methods overestimating dispersion effects. The conformation of the macrocycle backbones may be considered as controlled by sterical interactions; however, the chiroptical properties of the macrocycles are due to the presence of sulfur atoms. In contrast, the mutual interactions of the substituents at the sulfur atoms or interactions between macrocycle core and aromatic substituents at sulfur are less critical. They are only revealed at higher-energy spectral regions and are difficult to extract and interpret from other interactions.

Although the results may be considered preliminary, both imines and amines form in situ complexes with some metal cations. Among them, the titration of basic trianglamine **7** by Ag(I) cations revealed significant changes in the ECD spectrum’s magnitude and/or shape. Therefore, this or similar compounds may have potential applications in catalysis, synthesis, and supramolecular chemistry.

## Materials and methods

### Experimental details

All commercially available reagents were obtained from commercial suppliers and, unless specified otherwise, used in reactions without further purification. The anhydrous dichloromethane and chloroform were distilled over calcium hydride under an inert atmosphere. Flash column chromatography was performed on Merck Kieselgel type 60 (250–400 mesh). Merck Kieselgel type 60F_254_ analytical plates were used for TLC analysis.

^1^H and ^13^C NMR spectra were recorded on a Bruker 400 MHz or Bruker 600 MHz at ambient or at low temperature. All NMR spectra are reported in parts per million (ppm) downfield of TMS and were measured relative to the signals for residual CDCl_3_ (7.27 ppm and 77.0 ppm, respectively for ^1^H and ^13^C NMR spectra). All ^13^C NMR spectra were obtained with 1H decoupling. Mass spectra were recorded on AB Sciex TripleTOF^®^ 5600 + System. Melting points were measured by using open glass capillaries in a Büchi Melting Point B-545 apparatus.

A Jasco P-2000 polarimeter was used for optical rotation measurements (at 20 °C). UV and CD spectra were recorded on a Jasco J-810 spectropolarimeter at room temperature in cyclohexane and acetonitrile. In selected cases, dichloromethane has been used as the solvent. The UV and CD measurements have been done using a quartz cell of optical lengths 0.1 cm. The concentration of analytes ranged from 1.0 to 2.0 × 10^–4^ mol L^–1^. Background spectra of the pure solvents were recorded from 400 to 185 (225 nm in the case of dichloromethane) nm with the scan speed of 100 nm min^–1^. The ECD spectra of analytes were measured with 8 accumulations.

**Dialdehyde 3** was obtained according to the previously published procedure^35^.

**Dialdehyde 4**: terephthalaldehyde (2.6 g, 19.4 mmol) was dissolved in concentrated sulfuric acid (25 mL) and heated to 60 °C. *N*-Bromosuccinimide (3.98 g, 22.4 mmol) was added portionwise over 15 min, and the solution was heated at 60 °C for 3 h. The solution was poured onto ice, and the white precipitate was filtered off. The solid was dissolved in dichloromethane and extracted with sat. NaHCO_3_, then brine, and dried over anhydrous Na_2_SO_4_. The solvent was removed in vacuo. Purification of the residue by column chromatography (DCM/hexane 2:1 to DCM) furnished the product [yield 729 mg, (18%)] and dialdehyde **3** [yield 1.796 g (32%)]. All spectra are following the literature data^48^.

### General procedure for synthesis of symmetrical dialdehydes with sulfur substituents

A suspension of 2,5-dibromoterephthaldehyde (x mmol, 1 equiv.), corresponding thiol (2 equiv.) and K_2_CO_3_ (4 equiv.) in anhydrous DMF (25 mL) was mixed at 85 °C overnight. The mixture was cooled, then water (25 mL) was added and extracted with ethyl acetate (3 × 25 mL). The organic layers were washed with water and brine and dried over Na_2_SO_4_. The solvent was evaporated, and the residue was chromatographed through a silica gel column using hexane: DCM 1:1 as eluent to afford the product.

**Dialdehyde 5a**: orange amorphous solid, yield 547 mg (81%, x = 2 mmol); mp 147–149 °C; IR (ATR): 3062, 3029, 2919, 2835, 1675, 1603, 1582, 1495, 1451, 1351, 1294, 1177, 1158, 1100, 1065, 1033, 922, 869, 830, 809, 793, 700 cm^− 1^; ^1^H NMR (400 MHz, CDCl_3_) *δ*: 10.19 (s, 2 H), 7.86 (s, 2 H), 7.28–7.21 (m, 10 H), 4.14 (s, 4 H) ppm; ^13^C{H} NMR (101 MHz, CDCl_3_) *δ*: 190.3, 138.4, 137.8, 135.6, 133.0, 129.0, 128.7, 127.7, 39.3 ppm; HRMS (ESI) *m/z*: [M + Na]^+^ calcd for C_22_H_18_O_2_S_2_Na 401.0635, found 401.0627.

**Dialdehyde 5b**: orange amorphous solid, yield 314 mg (42%, x = 1.85 mmol); mp 163–165 °C; IR (ATR): 3062, 3026, 2955, 2918, 2871, 2790, 1675, 1601, 1582, 1480, 1447, 1428, 1418, 1209, 1176, 1108, 876, 783, 756, 729, 696, 660 cm^− 1^; ^1^H NMR (400 MHz, CDCl_3_) *δ*: 10.41 (s, 2 H), 7.86 (s, 2 H), 7.33–7.20 (m, 10 H), 3.29–3.25 (m, 4 H), 3.01–2.97 (m, 4 H) ppm; ^13^C{H} NMR (101 MHz, CDCl_3_) *δ*: 190.4, 139.2, 138.7, 137.1, 131.4, 128.7, 128.5, 126.8, 35.4, 35.1 ppm; HRMS (ESI) *m/z*: [M + Na]^+^ calcd for C_24_H_22_O_2_S_2_Na 429.0953, found 429.0950.

**Dialdehyde 5c**: yellow amorphous solid, yield 666 mg (72%, x = 2 mmol); mp 159–162 °C; IR (ATR): 3077, 2956, 2900, 2867, 2774, 1684, 1594, 1488, 1448, 1399, 1361, 1286, 1173, 1101, 1013, 891, 830, 779, 734, 652, 559 cm^− 1^; ^1^H NMR (300 MHz, CDCl_3_) *δ*: 10.26 (s, 2 H), 7.56 (s, 2 H), 7.43 (d, *J* = 8.5 Hz, 4 H), 7.36 (d, *J* = 8.5 Hz, 1.34 (s, 18 H) ppm; ^13^C{H} NMR (75 MHz, CDCl_3_) *δ*: 190.7, 152.5, 139.7, 136.4, 133.4, 133.0, 128.5, 127.1, 34.8, 31.2 ppm; HRMS (ESI) *m/z*: [M + Na]^+^ calcd for C_28_H_30_O_2_S_2_Na 485.1579, found 485.1580.

**Dialdehyde 5d**: yellow amorphous solid, yield 836 mg (77%, x = 2 mmol); mp 245–248 °C; IR (ATR): 3099, 2957, 2869, 2775, 1711, 1675, 1592, 1564, 1463, 1396, 1355, 1267, 1170, 1153, 1109, 1092, 1014, 948, 890, 825, 761, 692 cm^− 1^; ^1^H NMR (400 MHz, CDCl_3_) *δ*: 10.31 (s, 2 H), 8.04 (d, *J* = 8.6 Hz, 4 H), 7.76 (s, 2 H), 7.41 (d, *J* = 8.6 Hz, 4 H), 4.34 (t, *J* = 6.6 Hz, 4 H), 1.79–1.72 (m, 4 H), 1.53–1.42 (m, 4 H), 0.98 (t, *J* = 7.4 Hz, 6 H) ppm; ^13^C{H} NMR (101 MHz, CDCl_3_) *δ*: 189.9, 165.7, 138.9, 138.2, 137.6, 134.7, 131.1, 130.9, 130.4, 65.1, 30.7, 19.2, 13.7 ppm; HRMS (ESI) *m/z*: [M + Na]^+^ calcd for C_30_H_30_O_6_S_2_Na 573.1376, found 573.1369.

**Dialdehyde 5e**: yellow amorphous solid, yield 598 mg (72%, x = 1.85 mmol); mp 185–188 °C; IR (ATR): 3049, 2987, 2869, 2782, 1680, 1582, 1496, 1445, 1414, 1338, 1286, 11,751,128, 1097, 895, 876, 860, 790, 745, 473 cm^− 1^; ^1^H NMR (400 MHz, CDCl_3_) *δ*: 10.24 (s, 2 H), 7.98 (d, *J* = 1.7 Hz, 2 H), 7.89–7.6 (m, 4 H), 7.82–7.80 (m, 2 H), 7.63 (s, 2 H), 7.58–7.52 (m, 4 H), 7.44 (dd, *J* = 8.6, 1.9 Hz, 2 H) ppm; ^13^C{H} NMR (101 MHz, CDCl_3_) *δ*: 190.4, 139.3, 136.5, 133.9, 133.7, 133.0, 132.8, 129.9, 129.5, 129.3, 127.9, 127.7, 127.2, 127.0 ppm; HRMS (ESI) *m/z*: [M + Na]^+^ calcd for C_28_H_18_O_2_S_2_Na 473.0640, found 473.0639.

**Dialdehyde 5f**: the crude residue was then purified by column chromatography (Aluminum oxide, hexane-DCM 1:1) to afford product as a yellow crystalline solid, yield 828 mg (66%, x = 2 mmol); mp 178–180 °C; IR (ATR): 3059, 3029, 2923, 2875, 1682, 1658, 1597, 1489, 1444, 1383, 1339, 1319, 1277, 1146, 1010, 753, 740, 693, 637 cm^− 1^; ^1^H NMR (600 MHz, CDCl_3_) *δ*: 9.57 (s, 2 H), 7.60 (s, 2 H), 7.32–7.13 (m, 30 H) ppm; ^13^C{H} NMR (151 MHz, CDCl_3_) *δ*: 189.6, 142.9, 138.1, 136.5, 129.8, 128.0, 127.3, 127.1, 72.6 ppm; HRMS (ESI) *m/z*: [M + Na]^+^ calcd for C_46_H_34_O_2_S_2_Na 705.1892, found 705.1870.

**Dialdehyde 5 g**: A suspension of 2,5-dibromoterephthaldehyde (592 mg, 2 mmol, 1 equiv.), benzyl mercaptan (0.25 mL, 1 equiv.) and K_2_CO_3_ (590 mg, 2 equiv.) in anhydrous DMF (20 mL) was mixed at room temperature overnight. Water (25 mL) was added and extracted with ethyl acetate (3 × 25 mL). The organic layer was washed with water, brine and dried over Na_2_SO_4_. The solvent was evaporated and the residue was chromatographed through a silica gel using hexane: DCM 1:1 as eluent to afford the product as a yellow solid. Yield 353 mg (52%); mp 104–106 °C; IR (ATR): 3067, 3026, 2926, 2876, 2848, 1681, 1603, 1579, 1525, 1495, 1445, 1406, 1335, 1291, 1167, 1085, 826, 774, 696, 497, 478 cm^− 1^; ^1^H NMR (400 MHz, CDCl_3_) *δ*: 10.35 (s, 1H), 10.20 (s, 1H), 8.03 (s, 1H), 7.98 (s, 1H), 7.32–7.23 (m, 5 H), 4.19 (s, 2 H) ppm; ^13^C{H} NMR (101 MHz, CDCl_3_) *δ*: 190.7, 189.2, 140.8, 138.5, 136.0, 135.96, 135.3, 131.0, 129.0, 128.8, 127.9, 123.5, 38.7 ppm; HRMS (ESI) *m/z*: [M + Na]^+^ calcd for C_15_H_11_O_2_SBrNa 356.9555/358.9535, found 356.9543/358.9526.

**Dialdehyde 5 h**: A suspension of 2-bromoterephthaldehyde (405 mg, 1.9 mmol, 1 equiv.), benzyl mercaptan (0.25 mL, 1 equiv.) and K_2_CO_3_ (530 mg, 2 equiv.) in anhydrous DMF (20 mL) was mixed at room temperature overnight. Water (25 mL) was added and extracted with ethyl acetate (3 × 25 mL). The organic layer was washed with water, brine and dried over Na_2_SO_4_. The solvent was evaporated, and the residue was chromatographed through a silica gel column using hexane: DCM 1:1 as eluent to afford the product as a colorless crystalline solid. Yield 258 mg (53%); mp 98–105 °C; IR (ATR): 3058, 3029, 2987, 2972, 2926 cm^− 1^; ^1^H NMR (600 MHz, CDCl_3_) *δ*: 10.31 (s, 1H), 10.04 (s, 1H), 7.96 (d, *J* = 7.8 Hz, 1H), 7.95 (d, *J* = 1.4 Hz, 1H), 7.78 (dd, *J* = 7.8, 1.4 Hz, 1H), 7.31–7.24 (m, 5 H), 4.21 (s, 2 H) ppm; ^13^C{H} NMR (151 MHz, CDCl_3_) *δ*: 191.1, 190.7, 142.3, 139.2, 138.0, 135.5, 132.0, 130.3, 129.0, 128.7, 127.8, 126.8, 38.7 ppm; HRMS (ESI) *m/z*: [M + Na]^+^ calcd for C_15_H_12_O_2_SNa 279.0450, found 279.0454.

### General procedure for synthesis of macrocycles **6a**-**6 h**

To a solution of (*R*,* R*)-DACH (0.5 mmol, 1 equiv.) in DCM (1 mL) was added solution of dialdehyde (0.5 mmol, 1 eq) in DCM (4 mL). The solution was stirred at room temperature for 24 h. The solvent was evaporated to obtain a crude product.

**Macrocycle 6a**: yellow amorphous solid, yield 229 mg (100%); mp 109–113 °C; IR ATR: 2970, 2924, 2855, 1631, 1494, 1450, 1364, 1230, 1068, 1029, 695 cm^− 1^; ^1^H NMR (400 MHz, CDCl_3_) *δ*: 8.50 (s, 6 H), 7.71 (s, 6 H), 7.23–7.16 (m, 18 H), 7.05–7.02 (m,12 H), 3.79 (d, *J* = 12 Hz, 6 H), 3.58 (d, *J* = 11.9 Hz, 6 H), 3.34–3.31 (m, 6 H), 1.87–1.85 (m, 6 H),1.76 (bs, 12 H), 1.47 (m, 1H) ppm; ^13^C{H} NMR (101 MHz, CDCl_3_) *δ*: 158.0, 137.2, 136.5, 135.7, 129.1, 129.0, 128.4, 127.2, 74.1, 39.3, 32.7, 24.4 ppm; HRMS (ESI) *m/z*: [M + H]^+^ calcd for C_84_H_85_N_6_S_6_ 1369.5154, found 1369.5117.

**Macrocycle 6b**: pale yellow crystalline solid, yield 215 mg (100%); mp 160–162 °C; IR ATR: 3061, 2926, 2856, 1631, 1592, 1490, 1450, 1398, 1364, 1177, 1102, 1014, 936, 840, 758, 692, 426 cm^− 1^; ^1^H NMR (400 MHz, CDCl_3_) *δ*: 8.60 (s, 6 H), 7.85 (s, 6 H), 7.29–7.14 (m, 30 H), 3.48–3.41 (m, 6 H), 3.01–2.94 (m, 6 H), 2.88–2.81 (m, 6 H), 2.70 (t, *J* = 7.7 Hz, 12 H), 1.90–1.84 (m, 18 H), 1.54–1.52 (m, 6 H) ppm; ^13^C{H} NMR (101 MHz, CDCl_3_) *δ*: 157.8, 140.0, 137.1, 135.1, 128.6, 128.4, 126.4, 74.2, 35.6, 35.2, 32.8, 24.5 ppm; HRMS (ESI) *m/z*: [M + H]^+^ calcd for C_90_H_97_N_6_S_6_ 1454.6127, found 1454.6109.

**Macrocycle 6c**: pale yellow amorphous solid; yield 255 mg (100%); mp 172–174 °C; IR ATR: 2929, 2857, 1633, 1578, 1487, 1460, 1361, 1340, 1267, 1118, 1081, 1011, 821, 730, 545 cm^− 1^; ^1^H NMR (400 MHz, CDCl_3_) *δ*: 8.42 (s, 6 H), 7.88 (s, 6 H), 7.20 (d, *J* = 8.6 Hz, 12 H), 7.00 (d, *J* = 8.6 Hz, 12 H), 3.08–3.06 (m 6 H), 1.70–1.37 (m, 8 H), 1.27 (s,54 H) ppm; ^13^C{H} NMR (100 MHz, CDCl_3_) *δ*: 158.7, 149.5, 138.8, 135.2, 133.2, 132.7, 129.3, 126.2, 73.4, 34.5, 32.2,31.3, 24.2 ppm; HRMS (ESI) *m/z*: [M + H]^+^ calcd for C_102_H_121_N_6_S_6_, 1622.8055; found, 1622.7999.

**Macrocycle 6d**: pale yellow amorphous solid; yield 316 mg (100%); mp 94–96 °C; IR ATR: 2926, 2857, 1713, 1631, 1592, 1489, 1450, 1398, 1363, 1267, 1177, 1102, 1031, 840, 758, 690 cm^− 1^; ^1^H NMR (600 MHz, CDCl_3_) *δ*: 8.44 (s, 6 H), 8.09 (s, 6 H), 7.84 (d, *J* = 8.8 Hz, 12 H), 7.04 (d, *J* = 8.7 Hz, 12 H), 4.33–4.31 (m, 12 H), 3.19–3.14 (m, 6 H), 1.77–1.69 (m, 18 H), 1.57–1.42 (m, 24 H), 1.28 (t, *J* = 9.8 Hz, 6 H), 0.97 (t, *J* = 7.4 Hz, 18 H) ppm; ^13^C{H} NMR (151 MHz, CDCl_3_) *δ*: 166.0, 157.9, 142.9, 140.4, 135.0, 133.6, 130.2, 128.0, 126.9, 73.7, 64.9, 32.2, 30.8, 24.1, 19.2, 13.7 ppm; HRMS (ESI) *m/z*: [M + H]^+^ calcd for C_108_H_121_N_6_O_12_S_6_, 1886.7395, found 1886.7420.

**Macrocycle 6e**: total volume of DCM used for reaction was 18 mL; yellow amorphous solid; yield 267 mg (100%); mp 148–149 °C; IR ATR: 3051, 2924, 2854, 1626, 1587, 1499, 1477, 1361, 1339, 1132, 1069, 940, 848, 809, 740, 472 cm^− 1^; ^1^H NMR (600 MHz, CDCl_3_) *δ*: 8.45 (s, 6 H), 8.02 (s, 6 H), 7.74–7.73 (m, 6 H), 7.60–7.58 (m, 6 H), 7.50 (d, *J* = 1.6 Hz, 6 H), 7.45–7.42 (m, 18 H), 6.95 (dd, *J* = 8.6, 1.9 Hz, 1H), 3.07–3.02 (m, 6 H), 1.52 (d, *J* = 8.3 Hz, 6 H), 1.40 (d, *J* = 8.6 Hz, 6 H), 1.18 (d, *J* = 13.6 Hz, 6 H), 1.12 (t, *J* = 9.9 Hz, 6 H) ppm; ^13^C{H} NMR (151 MHz, CDCl_3_) *δ*: 158.3, 139.3, 134.9, 133.6, 133.5, 133.5, 131.8, 128.7, 127.8, 127.5, 127.2, 126.7, 126.5, 125.9, 73.6, 32.1, 24.0 ppm; HRMS (ESI) *m/z*: [M + H]^+^ calcd for C_102_H_85_N_2_S_6_ 1586.5188, found 1586.5203.

**Macrocycle 6f**: scale *n* = 0.64 mmol; the product was crystallized from DCM/hexane as pale yellow crystalline solid; yield 259 mg (53%); mp 124–134 °C; IR ATR: 3055, 3031, 2920, 2857, 1633, 1594, 1488, 1444, 1357, 1336, 1035, 735, 698, 614 cm^− 1^; ^1^H NMR (300 MHz, CDCl_3_) *δ*: 7.69 (s, 6 H), 7.57 (s, 6 H), 7.33–7.30 (m, 36 H), 6.93–6.91 (m, 54 H), 2.55 (bs, 6 H), 1.74–1.62 (m, 10 H), 1.27 (bs, 14 H) ppm; ^13^C{H} NMR (75 MHz, CDCl_3_) *δ*: 156.0, 143.9, 140.9, 135.7, 134.2, 129.8, 127.6, 126.4, 73.4, 71.6, 32.5, 24.5 ppm; HRMS (ESI) *m/z*: [M + H]^+^ calcd for C_156_H_133_N_6_S_6_ 2282.8944, found 2282.8954.

**Macrocycle 6 g**: the product crystallized from Et_2_O as mixture of isomers (symmetry *C*_1_ and *C*_3_ 3:1); pale yellow crystalline solid, yield 206 mg (100%); mp 155–159 °C; IR ATR: 3060,3027, 2925, 2854, 1631, 1494, 1448, 1361, 1070, 936, 836, 697, 418 cm^− 1^; ^1^H NMR (400 MHz, CDCl_3_) *δ*: 8.55 (s, 1H), 8.50 (s, 2 H), 8.49 (s, 1H), 8.46 (s, 1H), 8.46 (s, 1H), 8.44 (s, 1H), 8.41 (s, 1H), 8.00 (s, 1H), 7.97 (s, 1H), 7.94 (s, 2 H), 7.93 (s, 1H), 7.93 (s, 1H), 7.87 (s, 1H), 7.85 (s, 1H), 7.80 (s, 1H), 7.27–7.12 (m, 20 H), 4.00 (s, 2 H), 3.97–3.86 (m, 6 H), 3.53–3.42 (m, 4 H) 3.36–3.26 (m, 4 H), 1.89–1.74 (m, 24 H), 1.52–1.43 (m, 8 H) ppm; ^13^C{H} NMR (151 MHz, CDCl_3_) *δ*: 159.0, 158.97, 158.9, 158.7, 157.38, 157.35, 157.2, 138.6, 138.50, 138.48, 138.42, 136.41, 136.36, 136.31, 136.31, 136.3, 136.2, 136.1, 135.81, 135.78, 135.7, 135.59, 135.57, 131.5, 131.3, 131.2, 131.1, 130.9, 130.40, 130.39, 129.1, 129.0, 128.92, 128.89, 128.41, 128.38, 128.36, 127.29, 127.26, 127.2, 123.8, 123.7, 123.6, 123.5, 74.6, 74.4, 74.3, 74.2, 74.0, 73.9, 73.8, 73.5, 39.9, 39.6, 39.4, 39.36, 32.8, 32.63, 32.61, 32.4, 32.3, 24.31, 24.25, 24.19, 24.19 ppm; HRMS (ESI) *m/z*: [M + H]^+^ calcd for C_63_H_64_Br_3_N_6_S_3_ 1239.1879/1241.1858, found 1239.1893/1241.1881.

**Macrocycle 6 h**: mixture of isomers; pale yellow amorphous solid, yield 170 mg (100%); mp 117–123 °C; IR ATR: 3054, 2924, 2853, 1632, 1590, 1495, 1448, 1370, 1339, 1068, 109, 936, 812, 696, 472 cm^− 1^; ^1^H NMR (600 MHz, CDCl_3_) *δ*: 8.62 (d, *J* = 2.0 Hz, 6 H), 8.53 (d, *J* = 2.5 Hz, 6 H), 8.12 (s, 3 H), 8.11 (s, 3 H), 8.05 (s, 3 H), 8.03 (s, 3 H), 7.84 (d, *J* = 1.4 Hz, 3 H), 7.79 (s, 6 H), 7.73 (dd, *J* = 7.4, 3.4 Hz, 6 H), 7.72 (s, 3 H), 7.65 (dd, *J* = 7.9, 4.3 Hz, 6 H), 7.24–7.20 (m, 8 H), 7.18–7.10 (m, 36 H), 7.08–7.06 (m, 16 H), 6.94 (dd, *J* = 7.9, 1.3 Hz, 3 H), 6.91 (dd, *J* = 7.9, 1.3 Hz, 3 H), 3.89–3.70 (m, 24 H), 3.41–3.31 (m, 24 H), 1.85–1.75 (m, 72 H), 1.54–1.40 (m, 24 H) ppm; ^13^C{H} NMR (151 MHz, CDCl_3_) *δ*: 159.5, 158.6, 158.5, 158.4, 158.4, 137.6, 137.7, 137.7, 137.7, 137.6, 137.1, 137.1, 137.0, 136.8, 136.6, 136.5, 136.4, 136.4, 129.0, 128.9, 128.9, 128.8, 128.4, 128.3, 128.3, 128.3, 128.1, 128.1, 127.9, 127.9, 127.8, 127.8, 127.7, 127.5, 127.4, 127.4, 127.2, 127.2, 127.1, 127.1, 74.4, 74.4, 74.2, 74.1, 73.9, 73.7, 39.7, 39.4, 39.2, 39.2, 32.8, 32.7, 32.6, 32.6, 32.5, 32.5, 29.6, 29.6, 24.4, 24.4, 24.3, 24.3 ppm; HRMS (ESI) *m/z*: [M + H]^+^ calcd for C_63_H_67_N_6_S_3_ 1003.4584, found 1003.4581.

**Macrocycle 7**: To a solution of macrocycle **6a** (0.077 mmol, 1 equiv.) in DCM/MeOH (4 mL, 1:1, v/v) at 0 °C, NaBH_4_was added in one portion (15 mg, 4 mmol, 5.2 equiv.). The mixture was mixed overnight at room temperature. The solvents were evaporated and the residue was dissolved in DCM and washed twice with saturated solution of Na_2_CO_3_ and brine, dried over Na_2_SO_4_. The solvent was removed in vacuo to obtain the product as colourless foam, yield 75 mg (71%); mp 89–90 °C; IR ATR: 3287, 3059, 3026, 2922, 2851, 1583, 1493, 1450, 1356, 1335, 1175, 1092, 1069, 1028, 859, 764, 695, 561, 458 cm^− 1^; ^1^H NMR (600 MHz, CDCl_3_) *δ*: 7.28 (s, 6 H), 7.16–7.15 (m, 18 H), 7.06–7.05 (m, 12 H), 3.94–3.89 (m,12 H), 3.74 (d, *J* = 13.3 Hz, 6 H), 3.52 (d, *J* = 13.2 Hz, 6 H), 2.13–2.11 (m, 12 H), 1.71 (d, *J* = 8.4 Hz, 12 H),1.19 (t, *J* = 10.0 Hz, 6 H), 0.94 (bs, 6 H) ppm; ^13^C{H} NMR (101 MHz, CDCl_3_) *δ*: 139.8, 137.6, 133.5, 130.7, 128.9, 128.3, 127.0, 60.7, 48.5, 39.0, 31.3, 25.0. ppm; HRMS (ESI) *m/z*: [M + H]^+^ calcd for C_84_H_97_N_6_S_6_ 1381.6093, found 1381.6081.

### Calculation details

The theoretical approach that has been used in this work is common to all studied structures and includes (i) conformational search at molecular mechanics level (MM3); (ii) pre-optimization at the B3LYP/6-31G(d) level to reduce the number of thermally accessible conformers; (iii) parallel re-optimization of conformers found at the low-DFT level with the use of B3LYP hybrid functional, its modifications B3LYP-GD3BJ, which include the D3 version of Grimme’s dispersion with Becke-Johnson damping, and Truhlar’s pure functional M06L in the gas phase, followed by frequency calculations to confirm stability of received structures; (iv) calculations on relative energies using Boltzmann distribution at T = 298.15 K; (v) rotatory strengths calculations at the TD-DFT level for all stable conformers of relative energies ranging from 0.0 to 2.0 kcal mol^− 1^.

The preliminary conformer distribution search was performed by the Scigress package^49^ using the MM3 molecular mechanics force field for the macrocycle **6a**, **6c**, **6 h**, **6 g**, and the most challenging example, **6f**, molecules all with the assumed *R* configuration at the stereogenic centers. The possible conformers were analyzed using the systematic search methodology. Minimum energy conformers of relative steric energies (ΔESE) up to 10 kcal mol^− 1^ found by molecular mechanics were further fully optimized at the B3LYP/6-31G(d) level as implemented in the Gaussian16 package^43,44^, which significantly reduced the number of conformers. Higher accuracy calculations were performed at the B3LYP, B3LYP-GD3BJ, and M06L levels^44–46^. The conformers obtained at the DFT level were the real minima (no imaginary frequencies have been found). Total and free energy values have been calculated and used to get the Boltzmann population of conformers at 298.15 K. Only the results for conformers that differ from the most stable one by less than 2 kcal mol^− 1^ were considered for further calculations, following a generally accepted protocol^40,50^. The TD-DFT calculations of ECD were performed for all structures re-optimized at higher levels of theory. We used three different density functionals to calculate rotatory strengths, namely CAM-B3LYP^47^, M06-2X^46^, and ωB97XD functional^48^. Rotatory strengths were calculated using both length and velocity representations. In the present study, the differences between the length and velocity representations of the computed values of rotatory strengths were relatively small, so only the velocity representations were used further. The CD spectra were simulated by overlapping Gaussian functions for each transition according to the procedure previously described^50,51^. It should be noted that there are no substantial differences between ECD spectra calculated with these three functionals for the same molecule.

### Single crystals X-ray analysis

All crystals subjected to X-ray analysis were mounted on loops by crystal protection grease. Reflection intensities for all samples were measured on an Oxford Diffraction SuperNova Atlas diffractometer equipped with a Cu *Kα* radiation source (λ = 1.54184 Å) and an Atlas CCD detector. In all experiments, the diffraction data were collected at 130 K and the temperature was controlled with an Oxford Instruments Cryosystem cold nitrogen-gas blower. Data collection, reduction and analysis were carried out with CrysAlisPro software^52^. All crystal structures were solved by direct methods using SHELXT-2018 program^53^, and refined by full matrix least squares method on F2 using SHELXL-2018 program^54^. Non-hydrogen atoms were refined using anisotropic thermal parameters. Hydrogen atoms bonded to carbon atoms were placed in idealized positions and refined using the riding model, and their isotropic displacement parameters were set equal to 1.2Ueq(C).

The synthetic procedure specified absolute structures of the compounds – from the known absolute configuration of *trans*-(*R*,*R*)-1,2-diaminocyclohexane, which was used as a starting material in the syntheses and for measurements with a Cu *Kα* radiation source also confirmed using Flack parameter^55^. Graphical images were prepared using Olex2^37^, and Mercury programs^56^. Crystallographic data and refinement details are collected in **Table S17**. Selected geometrical parameters are juxtaposed in **Table S18**.

Analyzed crystal of **6f** was twinned and modeled with BASF parameter refined to 0.4212(9). In crystal **6f** solvent molecules have been identified on subsequent difference electron density maps but their disorder has not been precisely modelled. Instead, the electron density corresponding to these included solvent molecules was taken into account using the solvent mask procedure as implemented in Olex2 software^37^. A solvent mask was calculated and 256 electrons were found in a Volume of 1930 Å^3^ in 1 void per unit cell. This is consistent with the presence of 1 hexane, 1 dichloromethane, 3 water molecules per asymmetric unit, which account for 244 electrons per unit cell. In macrocycle **6f**, one phenyl ring was modelled for disorder with the site occupation factors 0.55 and 0.45.

The crystals structure of **6 g** contains a some amount of disordered solvent caged in intermolecular hole. A solvent mask was calculated and 46 electrons were found in a volume of 203 Å^3^ in 1 void per unit cell. This is consistent with the presence of diethyl ether (C_4_H_10_O) per asymmetric unit which account for 42 electrons per unit cell. The solvent mask procedure, implemented in Olex2 software was included in structure refinement. Additionally, the molecule of **6 g** is disordered. In crystal structure symmetry of macrocyclic molecule is C_1_ with one of the three bromine atoms on the opposite side of the ring plane. However, the arrangement of substituent for 20% of the molecules is different and all three bromine atoms are on the same side of the molecule (the refined occupancy factor is 0.194).

CCDC **2,394,844** (**6b**), **2,387,654** (**6f**) and **2,394,845** (**6 g**) contain the supplementary crystallographic data for this paper. These data can be obtained free of charge from The Cambridge Crystallographic Data Centre via www.ccdc.cam.ac.uk/data%5Frequest/cif.

## Electronic supplementary material

Below is the link to the electronic supplementary material.

Supplementary Material 1

## Data Availability

The original contributions presented in this study are included in this article/Supplementary Materials. Further inquiries can be directed to the corresponding author.
