# Supplementary material for: Thio-modified trianglimines, a novel group of chiral macrocyclic compounds of high structural dynamics
Source: Sci Rep. 2025 Jan 6;15:890. doi: 10.1038/s41598-025-85179-9 (PMC11704344; doi:10.1038/s41598-025-85179-9)
Supplement: Supplementary file 1 — Supplementary Material 1 [file 41598_2025_85179_MOESM1_ESM.pdf]

Supporting Information for:

Thio-modified trianglimines, a novel group of chiral macrocyclic compounds of high structural dynamics

Natalia Prusinowska[1], Agnieszka Czapik[1], Joanna Szymkowiak[2], and Marcin Kwit[1]*

1) Faculty of Chemistry, Adam Mickiewicz University, Uniwersytetu Poznańskiego 8, 61 614 Poznan, Poland. E-mail: marcin.kwit@amu.edu.pl

2) Faculty of Science, Department of Chemistry University of British Columbia, 2036 Main Mall, Vancouver, British Columbia, BC Canada V6T 1Z1.

TABLE OF CONTENTS

1 Experimental details 3

2 UV and ECD spectra 6

3 Calculation details 20

4 Single crystals X-ray analysis 153

5 Copies of ^{1}H and ^{13}C NMR spectra 162

6 References 201

1 Experimental details

All commercially available reagents were obtained from commercial suppliers and, unless specified otherwise, used in reactions without further purification. The anhydrous dichloromethane and chloroform were distilled over calcium hydride under an inert atmosphere. Flash column chromatography was performed on Merck Kieselgel type 60 (250- 400 mesh). Merck Kieselgel type $60F_{254}$ analytical plates were used for TLC analysis.

^{1}H and ^{13}C NMR spectra were recorded on a Bruker 400 MHz or Bruker 600 MHz at ambient or at low temperature. All NMR spectra are reported in parts per million (ppm) downfield of TMS and were measured relative to the signals for residual $CDCl_3$ (7.27 ppm and 77.0 ppm, respectively for ^{1}H and ^{13}C NMR spectra). All ^{13}C NMR spectra were obtained with 1H decoupling. Mass spectra were recorded on AB Sciex TripleTOF® 5600+ System. Melting points were measured by using open glass capillaries in a Büchi Melting Point B-545 apparatus.

A Jasco P-2000 polarimeter was used for optical rotation measurements (at 20 °C). UV and CD spectra were recorded on a Jasco J-810 spectropolarimeter at room temperature in cyclohexane and acetonitrile. In selected cases, dichloromethane has been used as the solvent. The UV and CD measurements have been done with the use of a quartz cell of optical lengths 0.1 cm. The concentration of analytes ranged from 1.0 to 2.0 × 10^{-4} mol L^{-1}. Background spectra of the pure solvents were recorded from 400 to 185 (225 nm in the case of dichloromethane) nm with the scan speed of 100 nm min^{-1}. The ECD spectra of analytes were measured with 8 accumulations.

Dialdehyde 3 was obtained according to the previously published procedure.[1]

Dialdehyde 4: terephthalaldehyde (2.6 g, 19.4 mmol) was dissolved in concentrated sulfuric acid (25 mL) and heated to 60 °C. *N*-Bromosuccinimide (3.98 g, 22.4 mmol) was added portionwise over 15 min, and the solution was heated at 60 °C for 3 h. The solution was poured onto ice, and the white precipitate was filtered off. The solid was dissolved in dichloromethane and extracted with sat. NaHCO3, then brine and dried over anhydrous Na_2SO_4. The solvent was removed in vacuo. Purification of the residue by column chromatography (DCM/hexane 2:1 to DCM) furnished the product [yield 729 mg, (18%)] and dialdehyde **3** [yield 1.796g (32%)]. All spectra are in accordance with literature data.

General procedure for synthesis of symmetrical dialdehydes with sulfur substituents

A suspension of 2,5-dibromoterephthaldehyde (x mmol, 1 equiv.), corresponding thiol (2 equiv.) and K_2CO_3 (4 equiv.) in anhydrous DMF (25 mL) was mixed at 85 °C for overnight. The mixture was cooled, the water (25 mL) was added and extracted with ethyl acetate (3 x 25 mL). The organic layer was washed with water, brine and dried over Na_2SO_4. The solvent was evaporated and the residue was chromatographed through a silica gel column using hexane:DCM 1:1 as eluent to afford product.

Dialdehyde 5a: orange amorphous solid, yield 547 mg (81%, x = 2 mmol); mp 147-149 °C; IR (ATR): 3062, 3029, 2919, 2835, 1675, 1603, 1582, 1495, 1451, 1351, 1294, 1177, 1158, 1100, 1065, 1033, 922, 869, 830, 809, 793, 700 cm^{-1}; ^{1}H NMR (400 MHz, $CDCl_3$) δ: 10.19 (s, 2H), 7.86 (s, 2H), 7.28-7.21 (m, 10H), 4.14 (s, 4H) ppm; $^{13}C\{H\}$ NMR (101 MHz, $CDCl_3$) δ: 190.3, 138.4, 137.8, 135.6, 133.0, 129.0, 128.7, 127.7, 39.3 ppm; HRMS (ESI) *m/z*: $[M + Na]^+$ calcd for $C_{22}H_{18}O_2S_2Na$ 401.0635, found 401.0627.

Dialdehyde 5b: orange amorphous solid, yield 314 mg (42%, x = 1.85 mmol); mp 163-165 °C; IR (ATR): 3062, 3026, 2955, 2918, 2871, 2790, 1675, 1601, 1582, 1480, 1447, 1428, 1418, 1209, 1176, 1108, 876, 783, 756, 729, 696, 660 cm^{-1}; ^{1}H NMR (400 MHz, $CDCl_3$) δ: 10.41 (s, 2H), 7.86 (s, 2H), 7.33-7.20 (m, 10H), 3.29-3.25 (m, 4H), 3.01-2.97 (m, 4H) ppm; $^{13}C\{H\}$ NMR (101 MHz, $CDCl_3$) δ: 190.4, 139.2, 138.7, 137.1, 131.4, 128.7, 128.5, 126.8, 35.4, 35.1 ppm; HRMS (ESI) *m/z*: $[M + Na]^+$ calcd for $C_{24}H_{22}O_2S_2Na$ 429.0953, found 429.0950.

Dialdehyde 5c: yellow amorphous solid, yield 666 mg (72%, x = 2 mmol); mp 159-162 °C; IR (ATR): 3077, 2956, 2900, 2867, 2774, 1684, 1594, 1488, 1448, 1399, 1361, 1286, 1173, 1101, 1013, 891, 830, 779, 734, 652, 559 cm^{-1}; ^{1}H NMR (300 MHz, $CDCl_3$) δ: 10.26 (s, 2H), 7.56 (s, 2H), 7.43 (d, *J* = 8.5 Hz, 4H), 7.36 (d, *J* = 8.5 Hz, 1.34 (s, 18H) ppm; $^{13}C\{H\}$ NMR (75 MHz, $CDCl_3$) δ: 190.7, 152.5, 139.7, 136.4, 133.4, 133.0, 128.5, 127.1, 34.8, 31.2 ppm; HRMS (ESI) *m/z*: $[M + Na]^+$ calcd for $C_{28}H_{30}O_2S_2Na$ 485.1579, found 485.1580.

Dialdehyde 5d: yellow amorphous solid, yield 836 mg (77%, x = 2 mmol); mp 245-248 °C; IR (ATR): 3099, 2957, 2869, 2775, 1711, 1675, 1592, 1564, 1463, 1396, 1355, 1267, 1170, 1153, 1109, 1092, 1014, 948, 890, 825, 761, 692 cm^{-1}; ^{1}H NMR (400 MHz, $CDCl_3$) δ: 10.31 (s, 2H), 8.04 (d, *J* = 8.6 Hz, 4H), 7.76 (s, 2H), 7.41 (d, *J* = 8.6 Hz, 4H), 4.34 (t, *J* = 6.6 Hz, 4H), 1.79-1.72 (m, 4H), 1.53-1.42 (m, 4H), 0.98 (t, *J* = 7.4 Hz, 6H) ppm; $^{13}C\{H\}$ NMR (101 MHz, $CDCl_3$) δ: 189.9, 165.7, 138.9, 138.2, 137.6, 134.7, 131.1, 130.9, 130.4, 65.1, 30.7, 19.2, 13.7 ppm; HRMS (ESI) *m/z*: $[M + Na]^+$ calcd for $C_{30}H_{30}O_6S_2Na$ 573.1376, found 573.1369.

Dialdehyde 5e: yellow amorphous solid, yield 598 mg (72%, x = 1.85 mmol); mp 185-188 °C; IR (ATR): 3049, 2987, 2869, 2782, 1680, 1582, 1496, 1445, 1414, 1338, 1286, 11751128, 1097, 895, 876, 860, 790, 745, 473 cm^{-1}; ^{1}H NMR (400 MHz, $CDCl_3$) δ:

10.24 (s, 2H), 7.98 (d, J = 1.7 Hz, 2H), 7.89-7.6 (m, 4H), 7.82-7.80 (m, 2H), 7.63 (s, 2H), 7.58-7.52 (m, 4H), 7.44 (dd, J = 8.6, 1.9 Hz, 2H) ppm; $^{13}C\{H\}$ NMR (101 MHz, $CDCl_3$) δ: 190.4, 139.3, 136.5, 133.9, 133.7, 133.0, 132.8, 129.9, 129.5, 129.3, 127.9, 127.7, 127.2, 127.0 ppm; HRMS (ESI) *m/z*: $[M + Na]^+$ calcd for $C_{28}H_{18}O_2S_2Na$ 473.0640, found 473.0639.

Dialdehyde 5f: the crude residue was then purified by column chromatography (Aluminum oxide, hexane-DCM 1:1) to afford product as a yellow crystalline solid, yield 828 mg (66%, x = 2 mmol); mp 178-180 °C; IR (ATR): 3059, 3029, 2923, 2875, 1682, 1658, 1597, 1489, 1444, 1383, 1339, 1319, 1277, 1146, 1010, 753, 740, 693, 637 cm^{-1}; ^1H NMR (600 MHz, $CDCl_3$) δ: 9.57 (s, 2H), 7.60 (s, 2H), 7.32-7.13 (m, 30H) ppm; $^{13}C\{H\}$ NMR (151 MHz, $CDCl_3$) δ: 189.6, 142.9, 138.1, 136.5, 129.8, 128.0, 127.3, 127.1, 72.6 ppm; HRMS (ESI) *m/z*: $[M + Na]^+$ calcd for $C_{46}H_{34}O_2S_2Na$ 705.1892, found 705.1870.

Dialdehyde 5g: A suspension of 2,5-dibromoterephthaldehyde (592 mg, 2 mmol, 1 equiv.), benzyl mercaptan (0.25 mL, 1 equiv.) and K_2CO_3 (590 mg, 2 equiv.) in anhydrous DMF (20 mL) was mixed at room temperature for overnight. The water (25 mL) was added and extracted with ethyl acetate (3 x 25 mL). The organic layer was washed with water, brine and dried over Na_2SO_4. The solvent was evaporated and the residue was chromatographed through a silica gel column using hexane:DCM 1:1 as eluent to afford product as yellow solid. Yield 353 mg (52%); mp 104-106 °C; IR (ATR): 3067, 3026, 2926, 2876, 2848, 1681, 1603, 1579, 1525, 1495, 1445, 1406, 1335, 1291, 1167, 1085, 826, 774, 696, 497, 478 cm^{-1}; ^1H NMR (400 MHz, $CDCl_3$) δ: 10.35 (s, 1H), 10.20 (s, 1H), 8.03 (s, 1H), 7.98 (s, 1H), 7.32-7.23 (m, 5H), 4.19 (s, 2H) ppm; $^{13}C\{H\}$ NMR (101 MHz, $CDCl_3$) δ: 190.7, 189.2, 140.8, 138.5, 136.0, 135.96, 135.3, 131.0, 129.0, 128.8, 127.9, 123.5, 38.7 ppm; HRMS (ESI) *m/z*: $[M + Na]^+$ calcd for $C_{15}H_{11}O_2SBrNa$ 356.9555/358.9535, found 356.9543/358.9526.

Dialdehyde 5h: A suspension of 2-bromoterephthaldehyde (405 mg, 1.9 mmol, 1 equiv.), benzyl mercaptan (0.25 mL, 1 equiv.) and K_2CO_3 (530 mg, 2 equiv.) in anhydrous DMF (20 mL) was mixed at room temperature for overnight. The water (25 mL) was added and extracted with ethyl acetate (3 x 25 mL). The organic layer was washed with water, brine and dried over Na_2SO_4. The solvent was evaporated and the residue was chromatographed through a silica gel column using hexane:DCM 1:1 as eluent to afford product as colourless crystalline solid. Yield 258 mg (53%); mp 98-105 °C; IR (ATR): 3058, 3029, 2987, 2972, 2926 cm^{-1}; ^1H NMR (600 MHz, $CDCl_3$) δ: 10.31 (s, 1H), 10.04 (s, 1H), 7.96 (d, J = 7.8 Hz, 1H), 7.95 (d, J = 1.4 Hz, 1H), 7.78 (dd, J = 7.8, 1.4 Hz, 1H), 7.31-7.24 (m, 5H), 4.21 (s, 2H) ppm; $^{13}C\{H\}$ NMR (151 MHz, $CDCl_3$) δ: 191.1, 190.7, 142.3, 139.2, 138.0, 135.5, 132.0, 130.3, 129.0, 128.7, 127.8, 126.8, 38.7 ppm; HRMS (ESI) *m/z*: $[M + Na]^+$ calcd for $C_{15}H_{12}O_2SNa$ 279.0450, found 279.0454.

General procedure for synthesis of macrocycles 6a-6h.

To a solution of (*R,R*)-DACH (0.5 mmol, 1 equiv.) in DCM (1 mL) was added solution of dialdehyde (0.5 mmol, 1 eq) in DCM (4 mL). The solution was stirred at room temperature for 24 h. The solvent was evaporated to obtain crude product.

Macrocycle 6a: yellow amorphous solid, yield 229 mg (100%); mp 109-113 °C; IR ATR: 2970, 2924, 2855, 1631, 1494, 1450, 1364, 1230, 1068, 1029, 695 cm^{-1}; ^1H NMR (400 MHz, $CDCl_3$) δ: 8.50 (s, 6H), 7.71 (s, 6H), 7.23-7.16 (m, 18H), 7.05-7.02 (m,12H), 3.79 (d, J = 12 Hz, 6H), 3.58 (d, J = 11.9 Hz, 6H), 3.34-3.31 (m, 6H), 1.87-1.85 (m, 6H),1.76 (bs, 12H), 1.47 (m, 1H) ppm; $^{13}C\{H\}$ NMR (101 MHz, $CDCl_3$) δ: 158.0, 137.2, 136.5, 135.7, 129.1, 129.0, 128.4, 127.2, 74.1, 39.3, 32.7, 24.4 ppm; HRMS (ESI) *m/z*: $[M + H]^+$ calcd for $C_{84}H_{85}N_6S_6$ 1369.5154, found 1369.5117/499.

Macrocycle 6b: pale yellow crystalline solid, yield 215 mg (100%); mp 160-162 °C; IR ATR: 3061, 2926, 2856, 1631, 1592, 1490, 1450, 1398, 1364, 1177, 1102, 1014, 936, 840, 758, 692, 426 cm^{-1}; ^1H NMR (400 MHz, $CDCl_3$) δ: 8.60 (s, 6H), 7.85 (s, 6H), 7.29-7.14 (m, 30H), 3.48-3.41 (m, 6H), 3.01-2.94 (m, 6H), 2.88-2.81 (m, 6H), 2.70 (t, J = 7.7 Hz, 12H), 1.90-1.84 (m, 18H), 1.54-1.52 (m, 6H) ppm; $^{13}C\{H\}$ NMR (101 MHz, $CDCl_3$) δ: 157.8, 140.0, 137.1, 135.1, 128.6, 128.4, 126.4, 74.2, 35.6, 35.2, 32.8, 24.5 ppm; HRMS (ESI) *m/z*: $[M + H]^+$ calcd for $C_{90}H_{97}N_6S_6$ 1454.6127, found 1454.6109.

Macrocycle 6c: pale yellow amorphous solid; yield 255 mg (100%); mp 172-174 °C; IR ATR: 2929, 2857, 1633, 1578, 1487, 1460, 1361, 1340, 1267, 1118, 1081, 1011, 821, 730, 545 cm^{-1}; ^1H NMR (400 MHz, $CDCl_3$) δ: 8.42 (s, 6H), 7.88 (s, 6H), 7.20 (d, J = 8.6 Hz, 12H), 7.00 (d, J = 8.6 Hz, 12H), 3.08-3.06 (m 6H), 1.70-1.37 (m, 8H), 1.27 (s,54H) ppm; $^{13}C\{H\}$ NMR (100 MHz, $CDCl_3$) δ: 158.7, 149.5, 138.8, 135.2, 133.2, 132.7, 129.3, 126.2, 73.4, 34.5, 32.2,31.3, 24.2 ppm; HRMS (ESI) *m/z*: $[M + H]^+$ calcd for $C_{102}H_{121}N_6S_6$, 1622.8055; found, 1622.7999.

Macrocycle 6d: pale yellow amorphous solid; yield 316 mg (100%); mp 94-96 °C; IR ATR: 2926, 2857, 1713, 1631, 1592, 1489, 1450, 1398, 1363, 1267, 1177, 1102, 1031, 840, 758, 690 cm^{-1}; ^1H NMR (600 MHz, $CDCl_3$) δ: 8.44 (s, 6H), 8.09 (s, 6H), 7.84 (d, J = 8.8 Hz, 12H), 7.04 (d, J = 8.7 Hz, 12H), 4.33-4.31 (m, 12H), 3.19-3.14 (m, 6H), 1.77-1.69 (m, 18H), 1.57-1.42 (m, 24H), 1.28 (t, J = 9.8 Hz, 6H), 0.97 (t, J = 7.4 Hz, 18H) ppm; $^{13}C\{H\}$ NMR (151 MHz, $CDCl_3$) δ: 166.0, 157.9, 142.9, 140.4, 135.0, 133.6, 130.2, 128.0, 126.9, 73.7, 64.9, 32.2, 30.8, 24.1, 19.2, 13.7 ppm; HRMS (ESI) *m/z*: $[M + H]^+$ calcd for $C_{108}H_{121}N_6O_{12}S_6$, 1886.7395, found 1886.7420.

Macrocycle 6e: total volume of DCM used for reaction was 18 mL; yellow amorphous solid; yield 267 mg (100%); mp 148-149 °C; IR ATR: 3051, 2924, 2854, 1626, 1587, 1499, 1477, 1361, 1339, 1132, 1069, 940, 848, 809, 740, 472 cm^{-1}; ^1H NMR (600

MHz, $CDCl_3$) δ: 8.45 (s, 6H), 8.02 (s, 6H), 7.74 – 7.73 (m, 6H), 7.60 – 7.58 (m, 6H), 7.50 (d, J = 1.6 Hz, 6H), 7.45 – 7.42 (m, 18H), 6.95 (dd, J = 8.6, 1.9 Hz, 1H), 3.07-3.02 (m, 6H), 1.52 (d, J = 8.3 Hz, 6H), 1.40 (d, J = 8.6 Hz, 6H), 1.18 (d, J = 13.6 Hz, 6H), 1.12 (t, J = 9.9 Hz, 6H) ppm; $^{13}C\{H\}$ NMR (151 MHz, $CDCl_3$) δ: 158.3, 139.3, 134.9, 133.6, 133.5, 133.5, 131.8, 128.7, 127.8, 127.5, 127.2, 126.7, 126.5, 125.9, 73.6, 32.1, 24.0 ppm; HRMS (ESI) *m/z*: [M + H]$^+$ calcd for $C_{102}H_{85}N_2S_6$ 1586.5188, found 1586.5203.

Macrocycle 6f: scale n = 0.64 mmol; the product was crystallized from DCM/hexane as pale yellow crystalline solid; yield 259 mg (53%); mp 124-134 °C; IR ATR: 3055, 3031, 2920, 2857, 1633, 1594, 1488, 1444, 1357, 1336, 1035, 735, 698, 614 cm^{-1}; ^1H NMR (300 MHz, $CDCl_3$) δ: 7.69 (s, 6H), 7.57 (s, 6H), 7.33-7.30 (m, 36H), 6.93-6.91 (m, 54H), 2.55 (bs, 6H), 1.74-1.62 (m, 10H), 1.27 (bs, 14H) ppm; $^{13}C\{H\}$ NMR (75 MHz, $CDCl_3$) δ: 156.0, 143.9, 140.9, 135.7, 134.2, 129.8, 127.6, 126.4, 73.4, 71.6, 32.5, 24.5 ppm; HRMS (ESI) *m/z*: [M + H]$^+$ calcd for $C_{156}H_{133}N_6S_6$ 2282.8944, found 2282.8954.

Macrocycle 6g: the product crystallized from Et_2O as mixture of isomers (symmetry C_1 and C_3 3:1); pale yellow crystalline solid, yield 206 mg (100%); mp 155-159 °C; IR ATR: 3060,3027, 2925, 2854, 1631, 1494, 1448, 1361, 1070, 936, 836, 697, 418 cm^{-1}; ^1H NMR (400 MHz, $CDCl_3$) δ: 8.55 (s, 1H), 8.50 (s, 2H), 8.49 (s, 1H), 8.46 (s, 1H), 8.46 (s, 1H), 8.44 (s, 1H), 8.41 (s, 1H), 8.00 (s, 1H), 7.97 (s, 1H), 7.94 (s, 2H), 7.93 (s, 1H), 7.93 (s, 1H), 7.87 (s, 1H), 7.85 (s, 1H), 7.80 (s, 1H), 7.27-7.12 (m, 20H), 4.00 (s, 2H), 3.97-3.86 (m, 6H), 3.53-3.42 (m, 4H) 3.36-3.26 (m, 4H), 1.89-1.74 (m, 24H), 1.52-1.43 (m, 8H) ppm; $^{13}C\{H\}$ NMR (151 MHz, $CDCl_3$) δ: 159.0, 158.97, 158.9, 158.7, 157.38, 157.35, 157.2, 138.6, 138.50, 138.48, 138.42, 136.41, 136.36, 136.31, 136.31, 136.3, 136.2, 136.1, 135.81, 135.78, 135.7, 135.59, 135.57, 131.5, 131.3, 131.2, 131.1, 130.9, 130.40, 130.39, 129.1, 129.0, 128.92, 128.89, 128.41, 128.38, 128.36, 127.29, 127.26, 127.2, 123.8, 123.7, 123.6, 123.5, 74.6, 74.4, 74.3, 74.2, 74.0, 73.9, 73.8, 73.5, 39.9, 39.6, 39.4, 39.36, 32.8, 32.63, 32.61, 32.4, 32.3, 24.31, 24.25, 24.19, 24.19 ppm; HRMS (ESI) *m/z*: [M + H]$^+$ calcd for $C_{63}H_{64}Br_3N_6S_3$ 1239.1879/1241.1858, found 1239.1893/1241.1881.

Macrocycle 6h: mixture of isomers; pale yellow amorphous solid, yield 170 mg (100%); mp 117-123 °C; IR ATR: 3054, 2924, 2853, 1632, 1590, 1495, 1448, 1370, 1339, 1068, 109, 936, 812, 696, 472 cm^{-1}; ^1H NMR (600 MHz, $CDCl_3$) δ: 8.62 (d, J = 2.0 Hz, 6H), 8.53 (d, J = 2.5 Hz, 6H), 8.12 (s, 3H), 8.11 (s, 3H), 8.05 (s, 3H), 8.03 (s, 3H), 7.84 (d, J = 1.4 Hz, 3H), 7.79 (s, 6H), 7.73 (dd, J = 7.4, 3.4 Hz, 6H), 7.72 (s, 3H), 7.65 (dd, J = 7.9, 4.3 Hz, 6H), 7.24 – 7.20 (m, 8H), 7.18 – 7.10 (m, 36H), 7.08 – 7.06 (m, 16H), 6.94 (dd, J = 7.9, 1.3 Hz, 3H), 6.91 (dd, J = 7.9, 1.3 Hz, 3H), 3.89 – 3.70 (m, 24H), 3.41 – 3.31 (m, 24H), 1.85 – 1.75 (m, 72H), 1.54 – 1.40 (m, 24H) ppm; $^{13}C\{H\}$ NMR (151 MHz, $CDCl_3$) δ: 159.5, 158.6, 158.5, 158.4, 158.4, 137.6, 137.7, 137.7, 137.7, 137.6, 137.1, 137.1, 137.0, 136.8, 136.6, 136.5, 136.4, 136.4, 129.0, 128.9, 128.9, 128.8, 128.4, 128.3, 128.3, 128.3, 128.1, 128.1, 127.9, 127.9, 127.8, 127.8, 127.7, 127.5, 127.4, 127.4, 127.2, 127.2, 127.1, 127.1, 74.4, 74.4, 74.2, 74.1, 73.9, 73.7, 39.7, 39.4, 39.2, 39.2, 32.8, 32.7, 32.6, 32.6, 32.5, 32.5, 29.6, 29.6, 24.4, 24.4, 24.3, 24.3 ppm; HRMS (ESI) *m/z*: [M + H]$^+$ calcd for $C_{63}H_{67}N_6S_3$ 1003.4584, found 1003.4581.

Macrocycle 7: To a solution of macrocycle **6a** (0.077 mmol, 1 equiv.) in DCM/MeOH (4 mL, 1:1, v/v) at 0 °C was added in one portion $NaBH_4$ (15mg, 4 mmol, 5.2 equiv.). The mixture was mixed overnight at room temperature. The solvents was evaporated and the residue was dissolved in DCM and washed twice with saturated solution of Na_2CO_3 and brine, dried over Na_2SO_4. The solvent was removed in vacuo to obtain the product as colourless foam, yield 75 mg (71%); mp 89-90 °C; IR ATR: 3287, 3059, 3026, 2922, 2851, 1583, 1493, 1450, 1356, 1335, 1175, 1092, 1069, 1028, 859, 764, 695, 561, 458 cm^{-1}; ^1H NMR (600 MHz, $CDCl_3$) δ: 7.28 (s, 6H), 7.16-7.15 (m, 18H), 7.06-7.05 (m, 12H), 3.94-3.89 (m,12H), 3.74 (d, J = 13.3 Hz, 6H), 3.52 (d, J = 13.2 Hz, 6H), 2.13-2.11 (m, 12H), 1.71 (d, J = 8.4 Hz, 12H),1.19 (t, J = 10.0 Hz, 6H), 0.94 (bs, 6H) ppm; $^{13}C\{H\}$ NMR (101 MHz, $CDCl_3$) δ: 139.8, 137.6, 133.5, 130.7, 128.9, 128.3, 127.0, 60.7, 48.5, 39.0, 31.3, 25.0. ppm; HRMS (ESI) *m/z*: [M + H]$^+$ calcd for $C_{84}H_{97}N_6S_6$ 1381.6093, found 1381.6081.

2 UV and ECD spectra

Table S1. Concentrations (c, in mol L-1) of the samples used for UV and ECD measurements.

	Cyclohexane	Dichloromethane	Acetonitrile
**6a**	7.31×10^{-4}	7.4×10^{-4}	4.5×10^{-5}
**6b**	6.44×10^{-5}	6.92×10^{-5}	3.75×10^{-5}
**6c**	6.21×10^{-5}	6.21×10^{-5}	6.23×10^{-5}
**6d**	5.38×10^{-5}	5.33×10^{-5}	5.4×10^{-5}
**6e**	5.33×10^{-5}	6.37×10^{-5}	3.77×10^{-5}
**6f**	8.79×10^{-5}	8.77×10^{-5}	8.8×10^{-5}
**6g**	6.51×10^{-5}	8.18×10^{-5}	1.47×10^{-5}
**6h**	9.98×10^{-5}	1.0005×10^{-4}	insoluble
**7**	6.85×10^{-5}	6.83×10^{-5}	5.86×10^{-5}

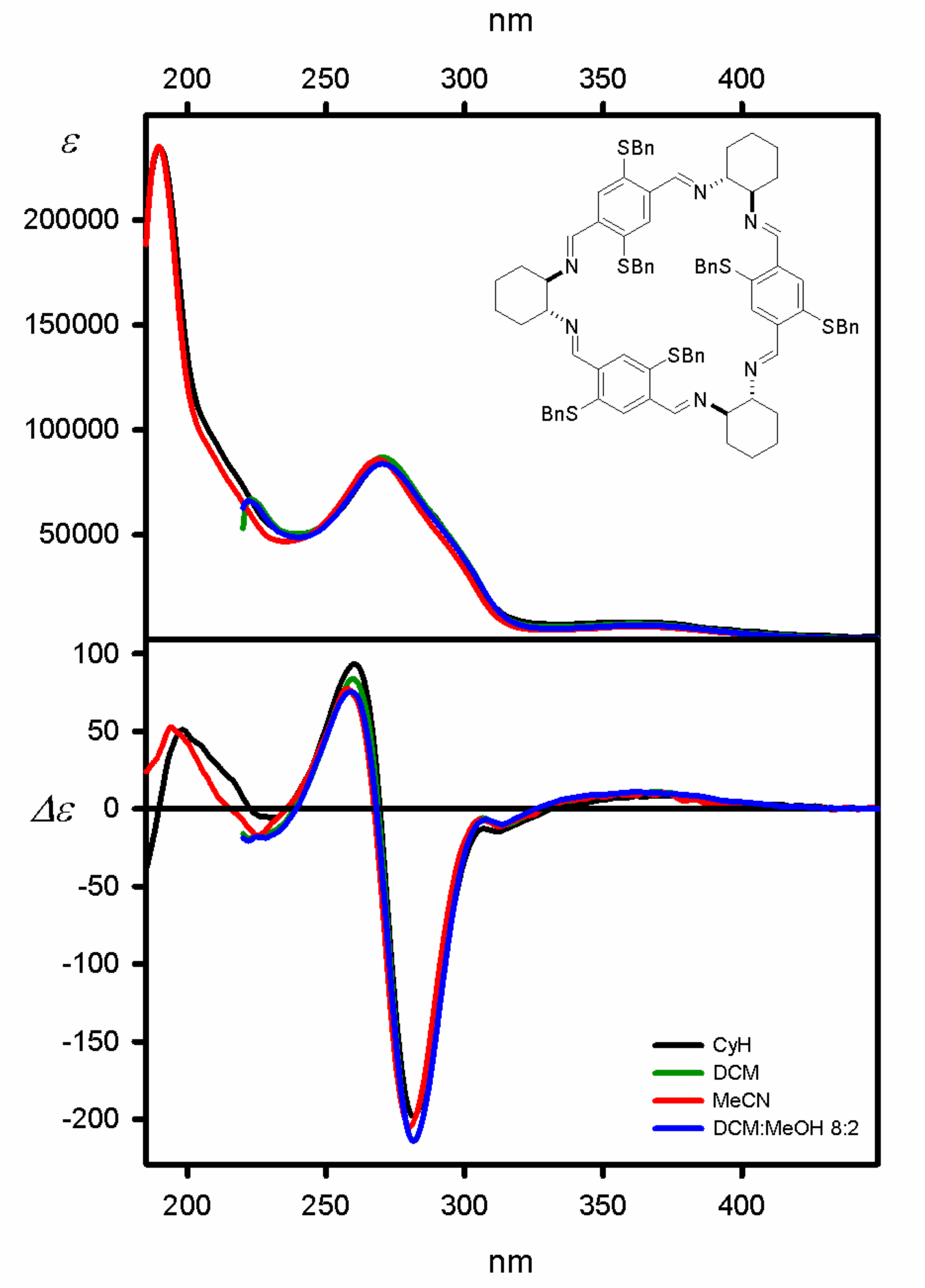

Figure S1. UV (upper panel) and ECD (lower panel) spectra of trianglimine **6a**, measured in cyclohexane (black lines), dichloromethane (dark-green lines), acetonitrile (red lines), and dichloromethane containing 20% of methanol (blue lines). $\Delta\varepsilon$ values are given in mol^{-1} cm^{-1} dm^{3}.

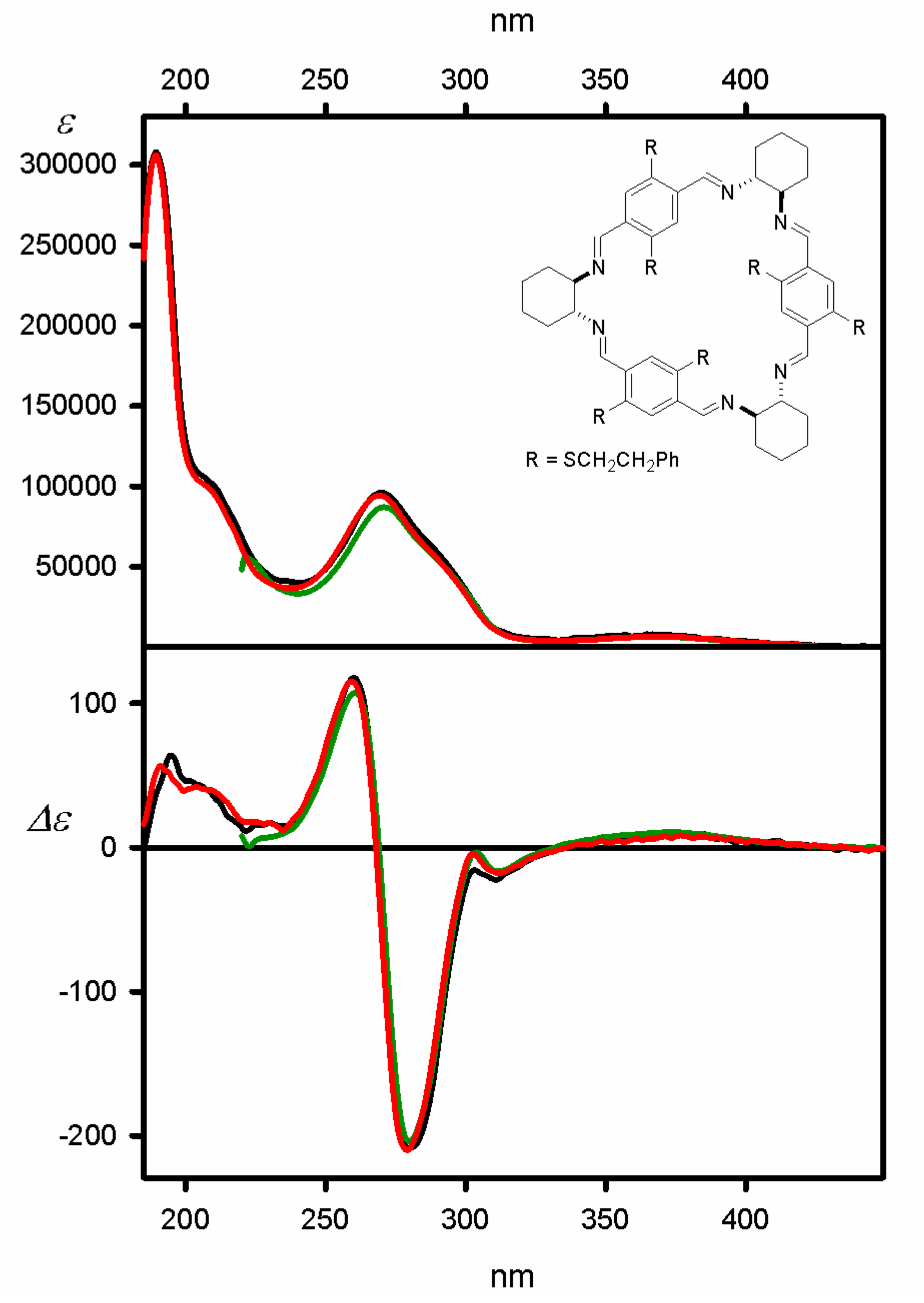

Figure S2. UV (upper panel) and ECD (lower panel) spectra of trianglimine **6b**, measured in cyclohexane (black lines), dichloromethane (dark-green lines), and acetonitrile (red lines). *Δε* values are given in mol^{-1} cm^{-1} dm^{3}.

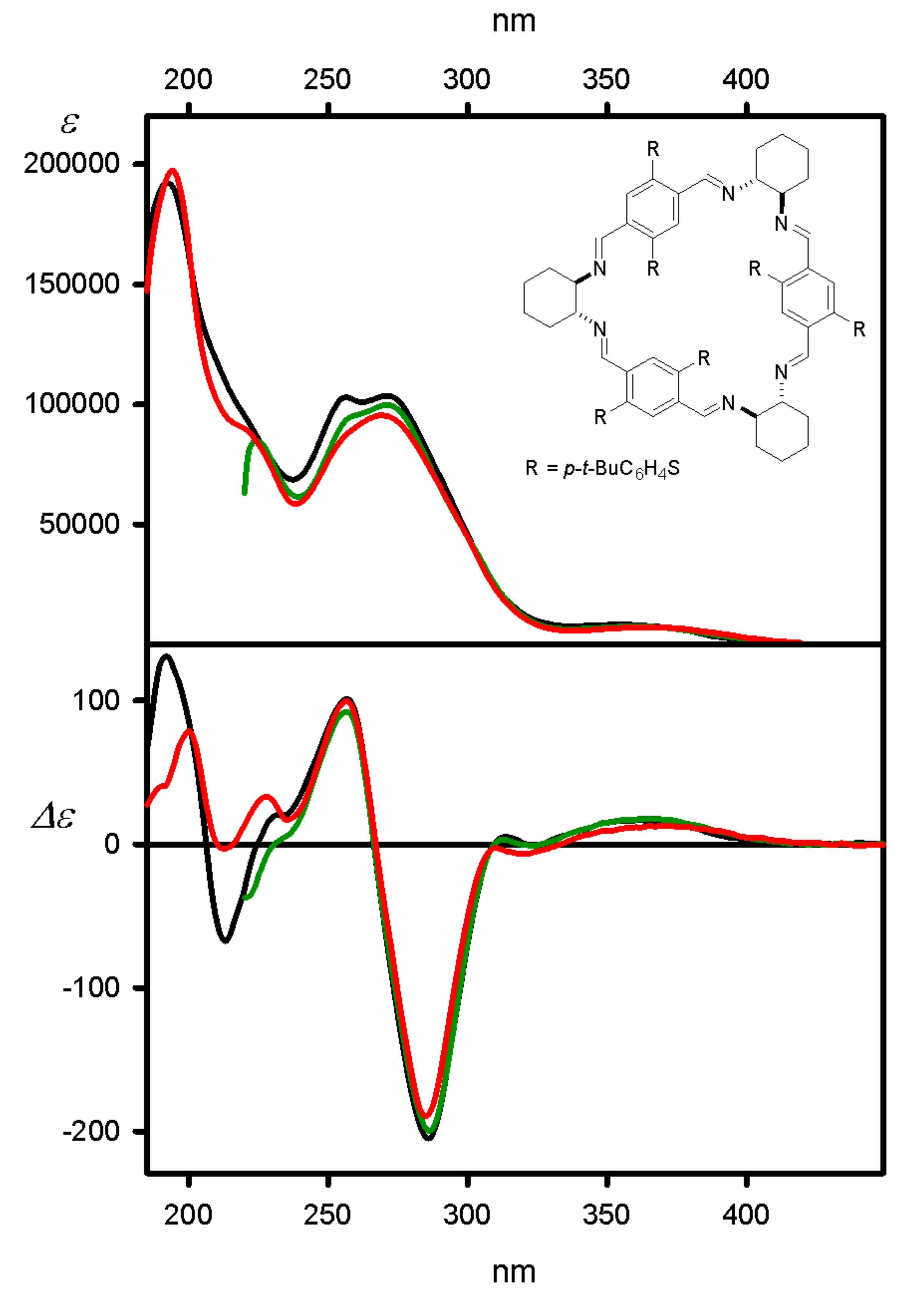

Figure S3. UV (upper panel) and ECD (lower panel) spectra of trianglimine **6c**, measured in cyclohexane (black lines), dichloromethane (dark-green lines), and acetonitrile (red lines). *Δε* values are given in mol^{-1} cm^{-1} dm^3.

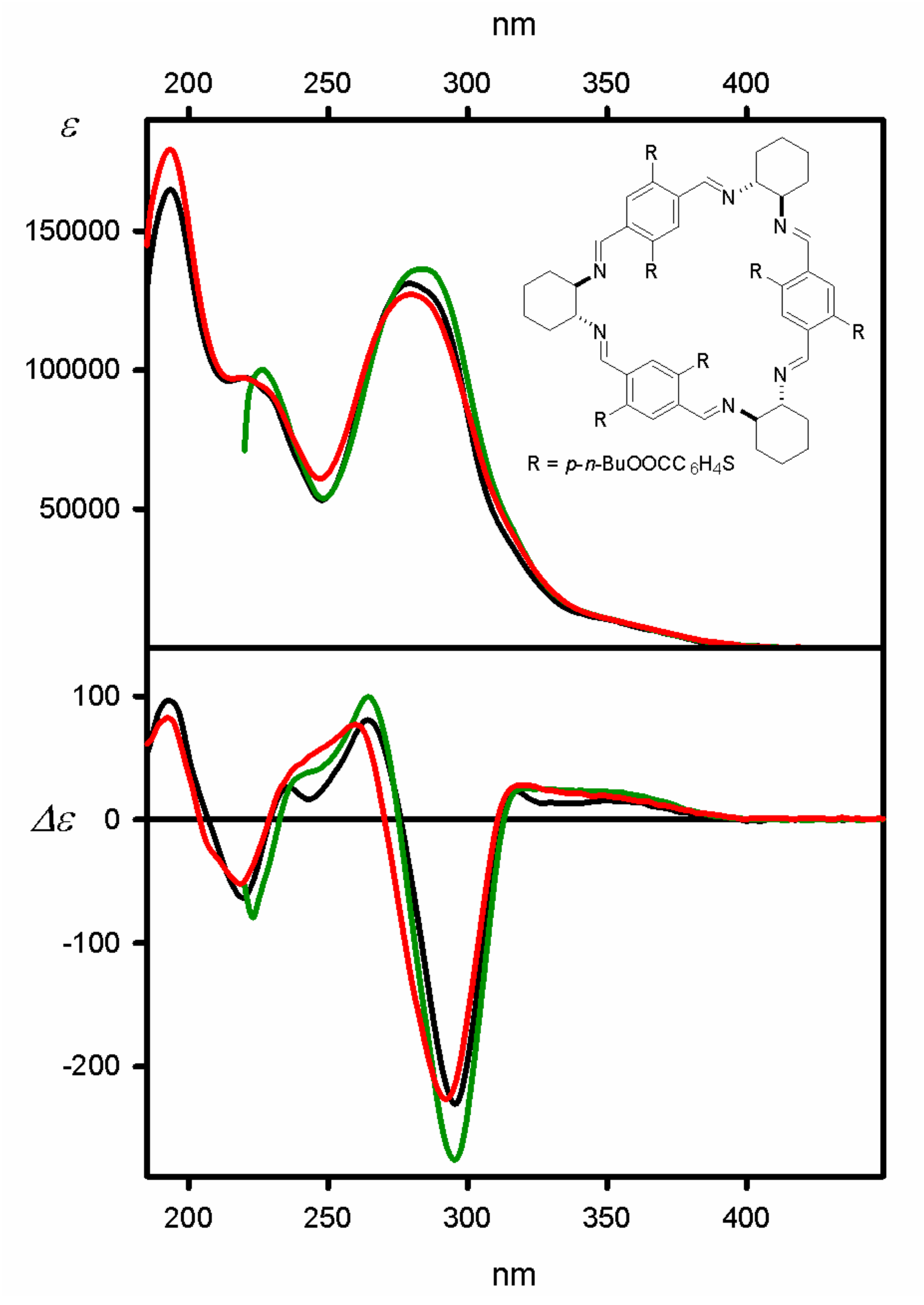

Figure S4. UV (upper panel) and ECD (lower panel) spectra of trianglimine **6d**, measured in cyclohexane (black lines), dichloromethane (dark-green lines), and acetonitrile (red lines). *Δε* values are given in mol^{-1} cm^{-1} dm^{3}.

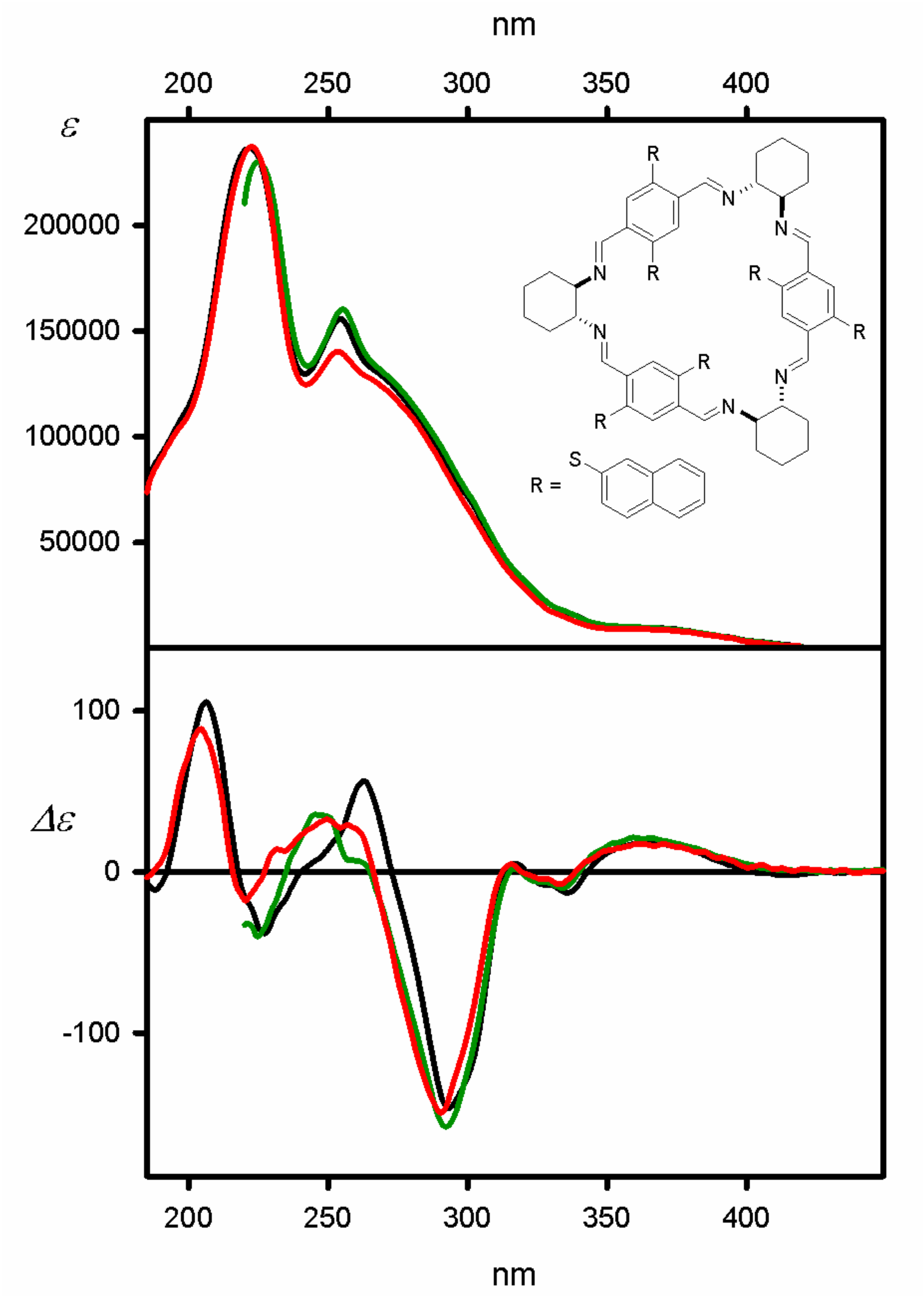

Figure S5. UV (upper panel) and ECD (lower panel) spectra of trianglimine **6e**, measured in cyclohexane (black lines), dichloromethane (dark-green lines), and acetonitrile (red lines). $\Delta\varepsilon$ values are given in mol^{-1} cm^{-1} dm^3.

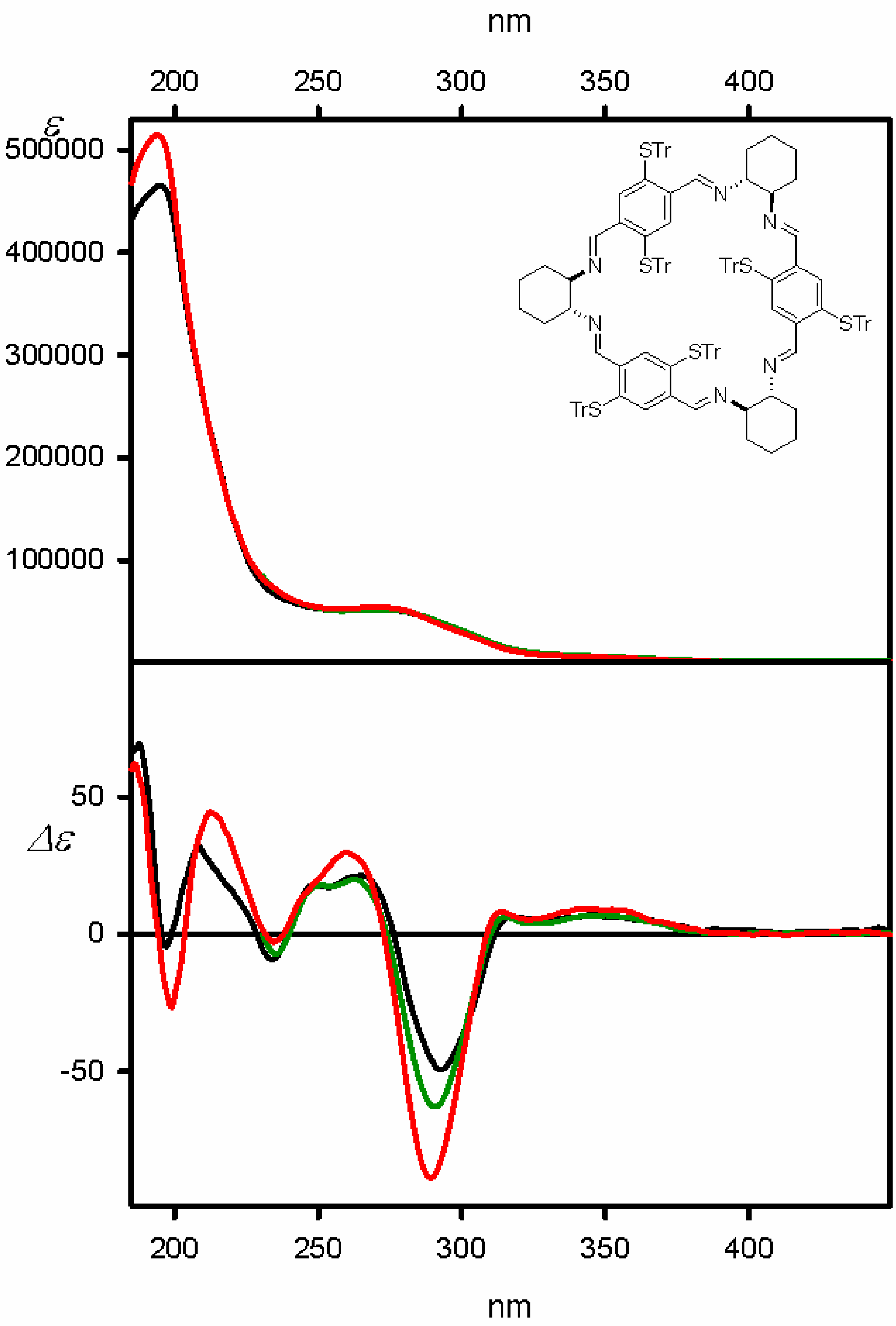

Figure S6. UV (upper panel) and ECD (lower panel) spectra of trianglimine **6f**, measured in cyclohexane (black lines), dichloromethane (dark-green lines), and acetonitrile (red lines). $\Delta\varepsilon$ values are given in mol^{-1} cm^{-1} dm^{3}.

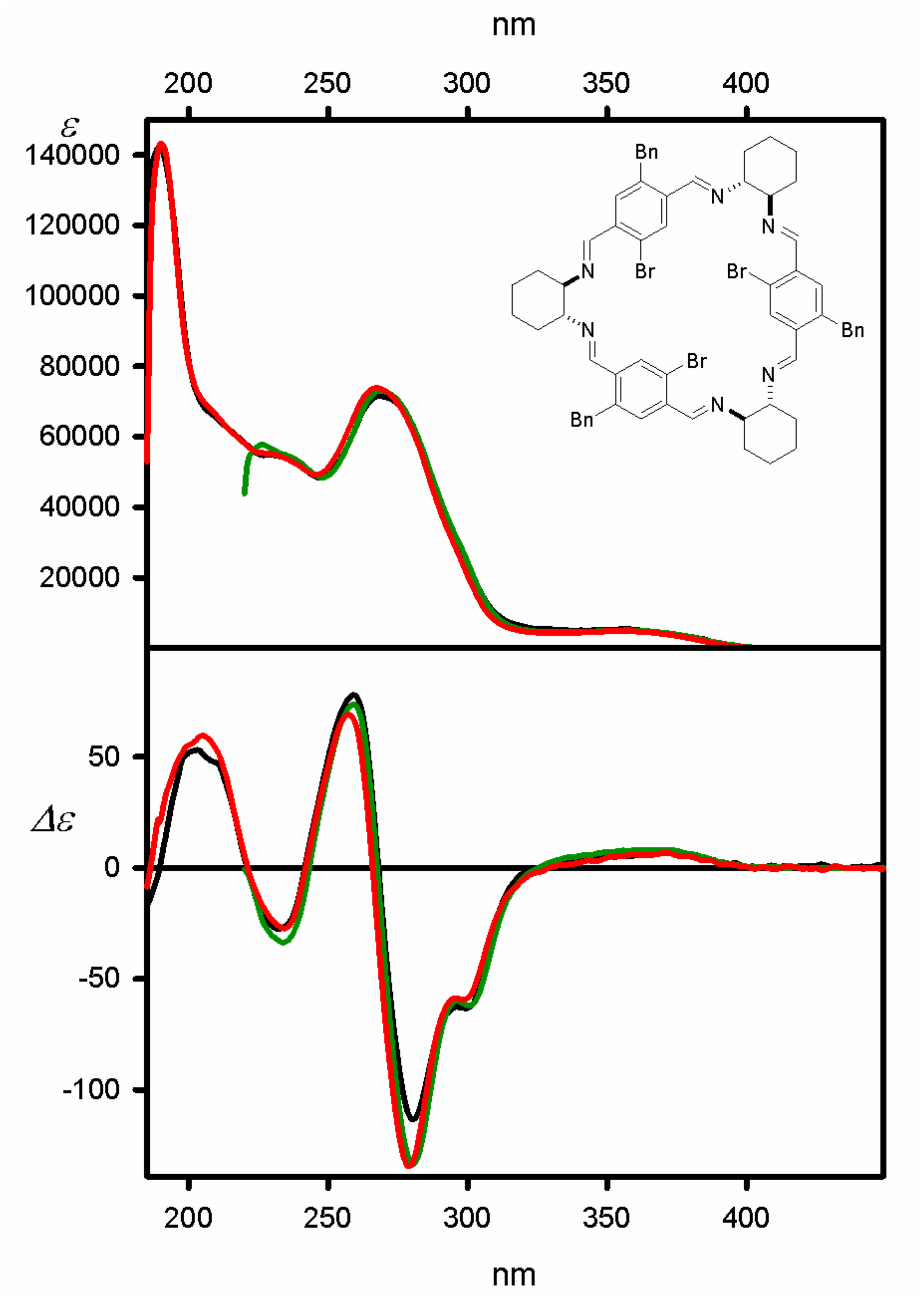

Figure S7. UV (upper panel) and ECD (lower panel) spectra of trianglimine **6g**, measured in cyclohexane (black lines), dichloromethane (dark-green lines), and acetonitrile (red lines). *Δε* values are given in mol^{-1} cm^{-1} dm^3.

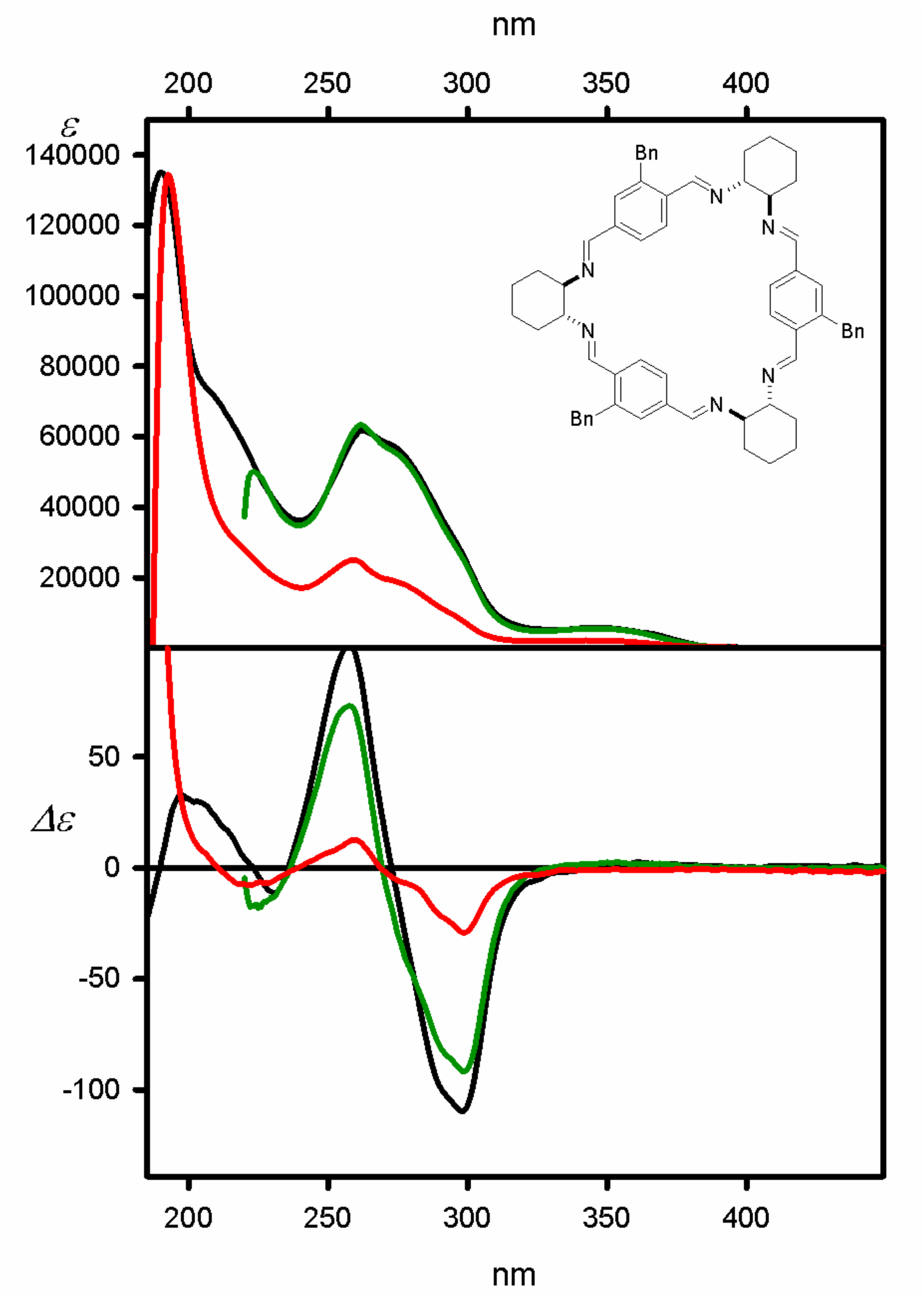

Figure S8. UV (upper panel) and ECD (lower panel) spectra of trianglimine **6h**, measured in cyclohexane (black lines), dichloromethane (dark-green lines), and acetonitrile (red lines). $\Delta\varepsilon$ values are given in mol^{-1} cm^{-1} dm^{3}.

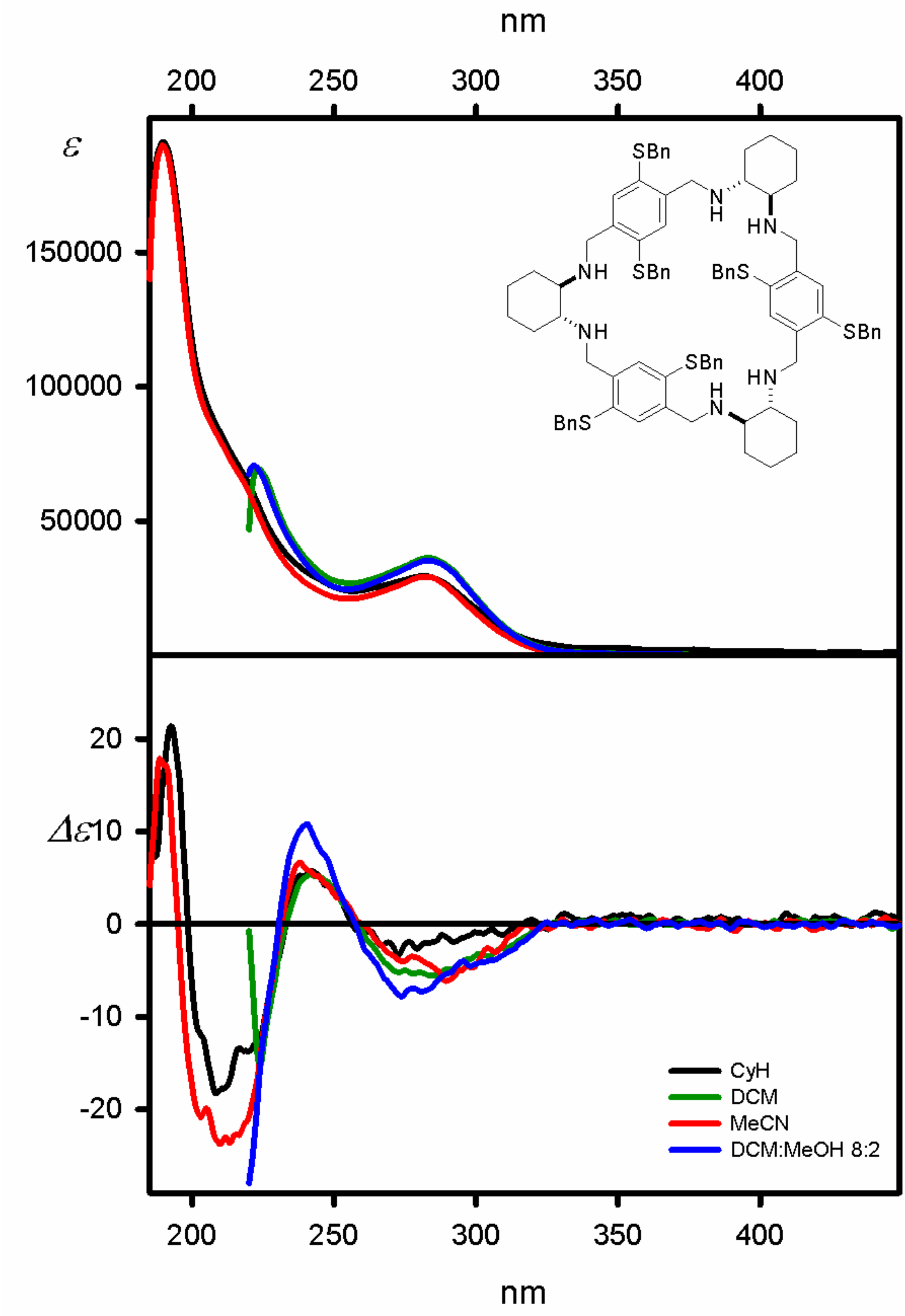

Figure S9. UV (upper panel) and ECD (lower panel) spectra of trianglamine **7**, measured in cyclohexane (black lines), dichloromethane (dark-green lines), acetonitrile (red lines), and dichloromethane containing 20% of methanol (blue lines). $\Delta\varepsilon$ values are given in mol^{-1} cm^{-1} dm^{3}.

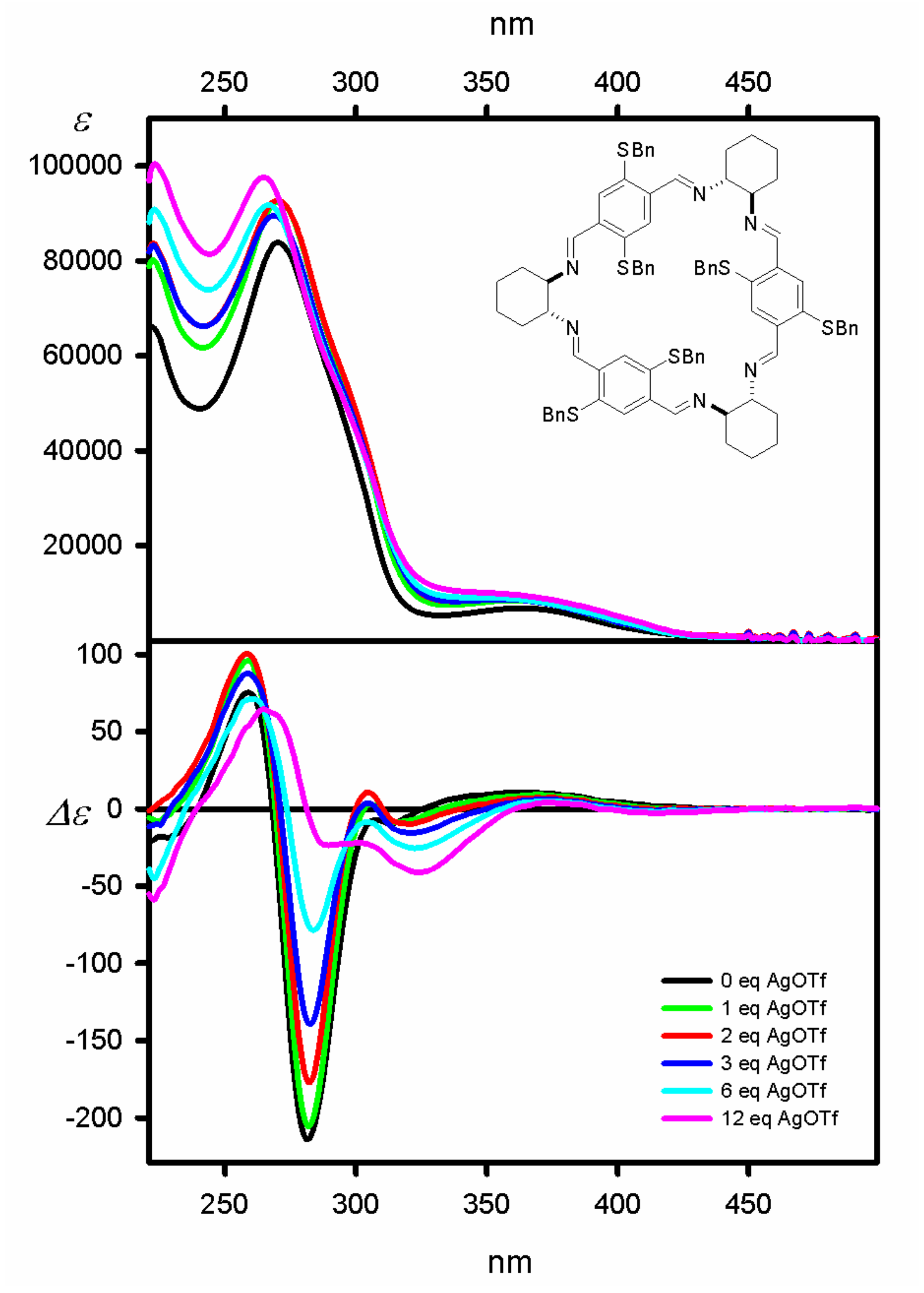

Figure S10. UV (upper panel) and ECD (lower panel) spectra measured during the titration of trianglimine **6a** with AgOTf. Spectra were measured in dichloromethane containing 20% of methanol. *Δε* values are given in mol^{-1} cm^{-1} dm^{3}.

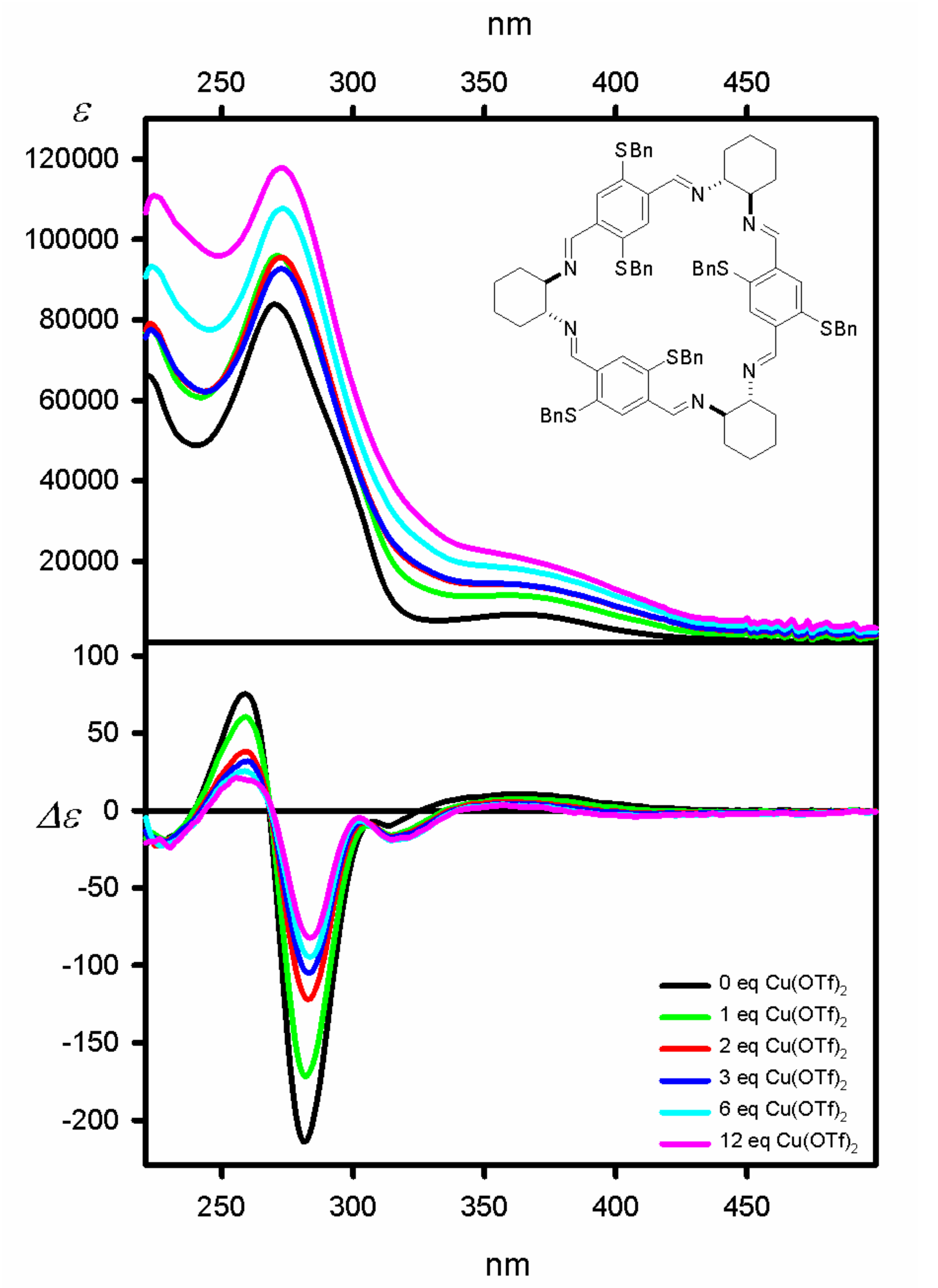

Figure S11. UV (upper panel) and ECD (lower panel) spectra measured during the titration of trianglimine **6a** with $Cu(OTf)_2 \cdot C_6H_6$. Spectra were measured in dichloromethane containing 20% of methanol. *Δε* values are given in $mol^{-1}\ cm^{-1}\ dm^3$.

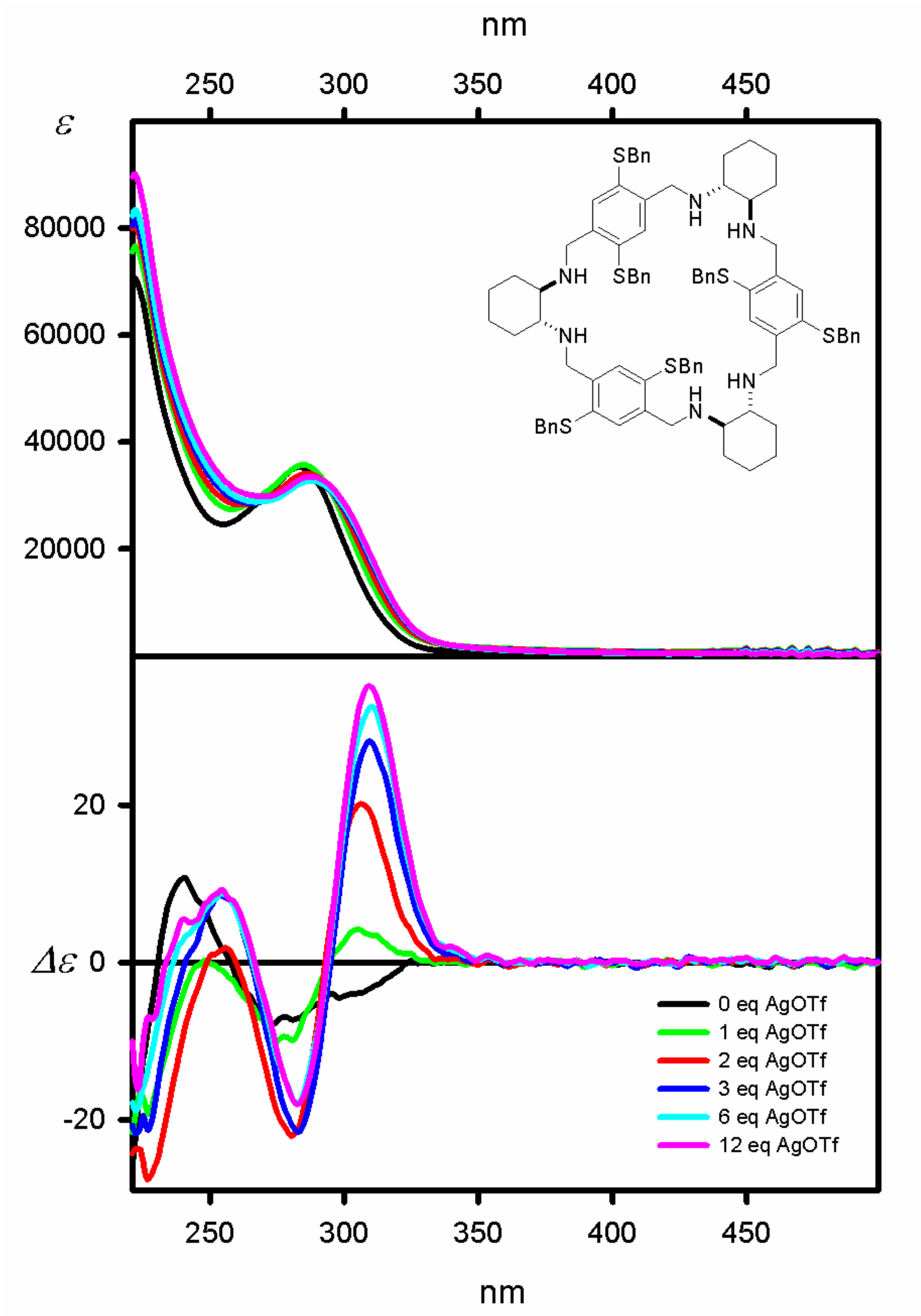

Figure S12. UV (upper panel) and ECD (lower panel) spectra measured during the titration of trianglamine **7** with AgOTf. Spectra were measured in dichloromethane containing 20% of methanol. *Δε* values are given in mol^{-1} cm^{-1} dm^{3}.

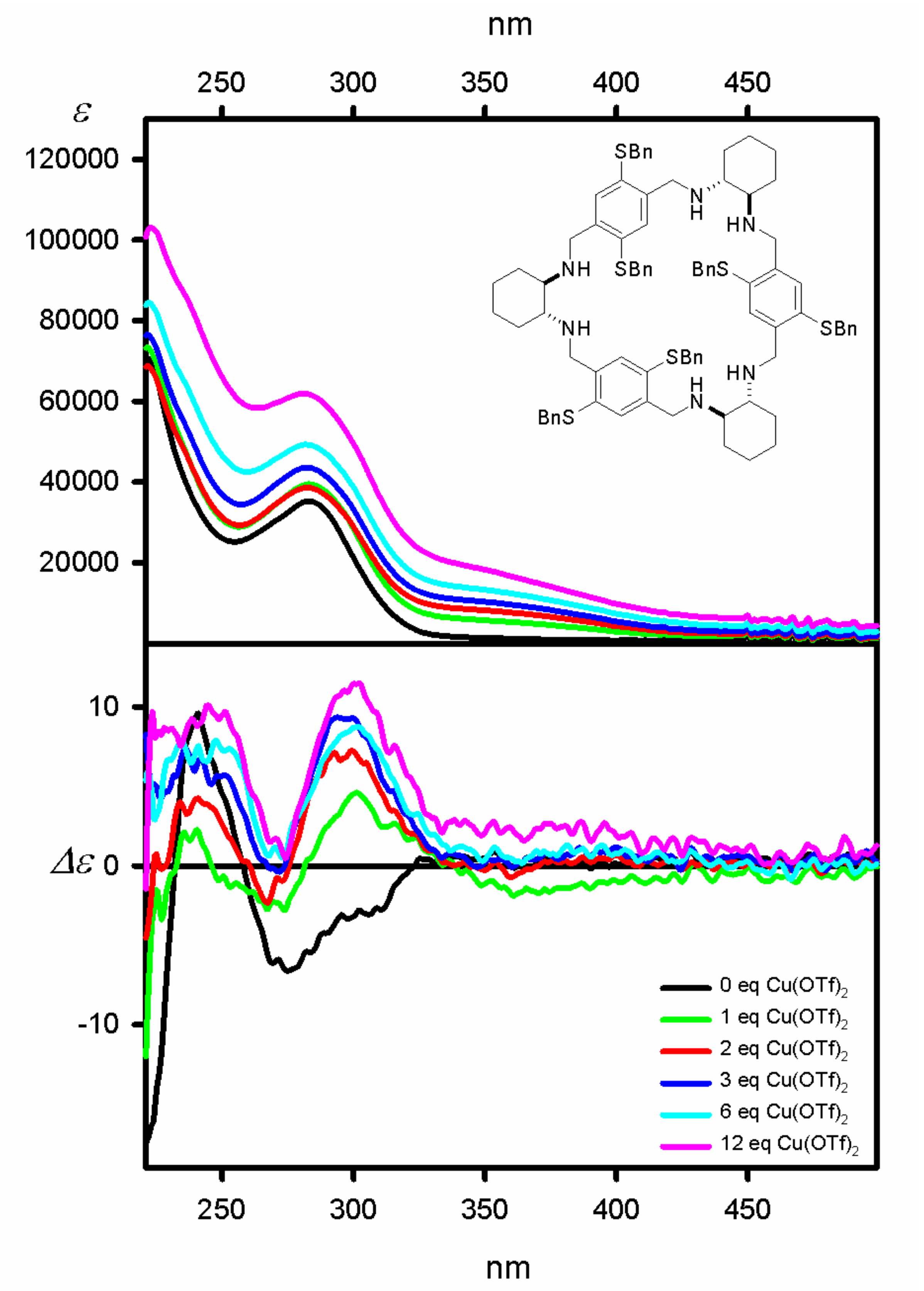

Figure S13. UV (upper panel) and ECD (lower panel) spectra measured during the titration of trianglamine **7** with $Cu(OTf)_2{\cdot}C_6H_6$. Spectra were measured in dichloromethane containing 20% of methanol *Δε* values are given in $mol^{-1}\ cm^{-1}\ dm^3$.

3 Calculation details

The calculation scheme was as follows: after building a model of the molecule, an extensive conformation search was performed at the molecular mechanics (MM) level.[2] Then, the conformers found at the MM level were pre-optimized at the B3LYP/6-31G(d) level of theory, which, on the one hand, allowed for the elimination of duplicates and, on the other hand, constituted a good starting point for the higher-level calculations.[3,4] The structures thus obtained were optimized with the use of three different functionals: a golden standard – B3LYP hybrid functional,[4] its modifications B3LYP-GD3BJ, which include the D3 version of Grimme's dispersion with Becke-Johnson damping,[5] and Truhlar's pure functional M06L, which we successfully used for calculations of structure and supramolecular assemblies of the calixsalen macrocycles.[6,7]

In fact, the structure of the compounds under study dictated the addition of dispersion correction to the B3LYP function. All of them contain aromatic parts attached to more or less flexible arms, and they may non-covalently interact, thus, affecting the structure of a given conformer. Except for **6f**, the structural calculations were performed using an enhanced basis set.

In principle, knowing the structure-energy relationships within a set of thermally available conformers of chiral and optically active compounds and their aggregates allows for quickly determining their preferred structure (configuration and/or conformation) by comparing experimental and calculated optical spectra.[8,9,10] Thus, calculations of UV and ECD spectra and their Boltzmann averaging concerning the differences in their electronic energies and the differences in Gibb's energies were the penultimate stage of the study. The UV and ECD spectra were calculated using three different hybrid functionals, CAM-B3LYP,[11] M06-2X,[6] and ωB97XD,[12] each previously successfully used for these purposes.[13] In the last step of the analysis, the theoretical results were confronted with the experimental ones, and on this basis, the most reliable calculation method can be indicated. Additionally, the lowest energy structures were directly compared with the structures found in the solid state (when available).

3.1 Computational data

Table S2 Total (*E*, in Hartree) and elative energies (*ΔE*, *ΔΔG*, in kcal mol^{-1}), percentage populations (Pop.) and number of imaginary frequencies calculated at the B3LYP/6-311G(d,p) level for low-energy conformers of **6a**.

Conformer[a]	*E*	*ΔE*	Pop.	*ΔΔG*	Pop.	#ImFreq
16	-5970.18125	0.58	6.56	3.59		0
20	-5970.17912	1.92	0.68	2		0
22	-5970.1795	1.68	1.03	2.34		0
27	-5970.18057	1.01	3.17	2.23		0
31	-5970.17905	1.96	0.64	3.07		0
39	-5970.18186	0.2	12.53	2.51		0
43	-5970.18013	1.28	2	0.41	16.69	0
49	-5970.17948	1.7	1	4.42		0
50.	-5970.18218	0	17.5	3.02		0
63	-5970.18076	0.89	3.91	0.68	10.52	0
68	-5970.18052	1.04	3.02	1.79	1.62	0
72	-5970.1818	0.24	11.73	0.84	8.11	0
73	-5970.17965	1.59	1.2	2.27		0
74	-5970.1818	0.24	11.66	0.49	14.64	0
75	-5970.18012	1.29	1.98	5.04		0
76	-5970.18043	1.09	2.75	1.11	5.11	0
83	-5970.18001	1.36	1.76	4.39		0
86	-5970.1814	0.49	7.66	0.95	6.75	0
91	-5970.18113	0.66	5.76	0	33.38	0
92	-5970.17988	1.44	1.54	2.73		*0*
97	-5970.18009	1.31	1.91	1.39	3.17	0

[a] conformers are numbered according to their appearance during conformational search

Table S3. Total (E, in Hartree) and relative energies (ΔE, $\Delta\Delta G$, in kcal mol^{-1}), percentage populations (Pop.) and number of imaginary frequencies calculated at the B3LYP-GD3BJ/6-311G(d,p) level for low-energy conformers of **6a**.

Conformer[a]	*E*	*ΔE*	Pop.	*ΔΔG*	Pop.	#ImFreq
32	-5970.71602	0.93	8.8	3.15		0
53	-5970.71611	0.88	9.68	4.29		0
60	-5970.71742	0.05	38.89	0	85.35	0
93	-5970.71751	0	42.63	1.04	14.65	0

[a] conformers are numbered according to their appearance during conformational search

Table S4. Total (E, in Hartree) and relative energies (ΔE, $\Delta\Delta G$, in kcal mol^{-1}), percentage populations (Pop.) and number of imaginary frequencies calculated at the M06L/6-311G(d,p) level for low-energy conformers of **6a**.

Conformer[a]	*E*	*ΔE*	Pop.	*ΔΔG*	Pop.	#ImFreq
5	-5969.63595	2.13		1.75	3.49	0
7	-5969.63608	2.05		1.99	2.34	0
32	-5969.63584	2.2		0.67	21.77	0
53	-5969.63703	1.45	6	4.06		0
60	-5969.63836	0.62	24.43	0	67.15	0
81	-5969.63934	0	69.56	2.17		0
82	-5969.63561	2.34		1.51	5.26	0

[a] conformers are numbered according to their appearance during conformational search

Table S5. Total (E, in Hartree) and relative energies (ΔE, $\Delta\Delta G$, in kcal mol^{-1}), percentage populations (Pop.) and number of imaginary frequencies calculated at the B3LYP/6-31G(d,p) level for low-energy conformers of **6c**.

Conformer[a]	*E*	*ΔE*	Pop.	*ΔΔG*	Pop.	#ImFreq
15	-6676.98863	0.55	7.44	0.85	5.54	0
18	-6676.98728	1.4	1.77	0.41	11.72	0
20	-6676.98914	0.23	12.81	2.48		0
25	-6676.98674	1.74	1.01	1.76	1.2	0
26	-6676.98734	1.36	1.91	0	23.26	0
27	-6676.98733	1.37	1.87	0.29	14.36	0
29	-6676.98739	1.33	2	1.28	2.68	0
30	-6676.9885	0.63	6.48	0.27	14.62	0
31	-6676.98745	1.29	2.13	1.63	1.48	0
38	-6676.98743	1.31	2.08	2.35		0
40	-6676.98742	1.31	2.06	1.73	1.25	0
42	-6676.9874	1.32	2.02	1.66	1.4	0
46	-6676.98785	1.04	3.27	2.06		0
47	-6676.98809	0.89	4.19	2.77		0
48	-6676.98557	2.47		1.79	1.13	0
49	-6676.98554	2.49		1.76	1.18	0
53	-6676.98697	1.59	1.28	1.24	2.88	0
61	-6676.98788	1.02	3.36	1.95	0.87	0
71	-6676.98812	0.87	4.36	1.66	1.42	0
77	-6676.98612	2.13		1.98	0.82	0
78	-6676.9892	0.19	13.69	2.32		0
79	-6676.98847	0.65	6.32	1.84	1.04	0
80	-6676.98951	0	18.94	1.92	0.91	0
82	-6676.98542	2.56		1.22	2.95	0
84	-6676.98544	2.55		1.05	3.96	0
86	-6676.98433	3.25		1.8	1.11	0
90	-6676.98672	1.75	0.98	4.6		0
92	-6676.98536	2.6		1.01	4.21	0

[a] conformers are numbered according to their appearance during conformational search

Table S6. Total (E, in Hartree) and relative energies (ΔE, $\Delta\Delta G$, in kcal mol^{-1}), percentage populations (Pop.) and number of imaginary frequencies calculated at the B3LYP-GD3BJ/6-31G(d,p) level for low-energy conformers of **6c**.

Conformer[a]	*E*	*ΔE*	Pop.	*ΔΔG*	Pop.	#ImFreq
59	-6677.65174	1.79	3.3	1.2	10.1	0
65	-6677.6534	0.75	19.2	1.6	5.15	0
85	-6677.6546	0	68.28	0	76.77	0
93	-6677.65271	1.19	9.22	1.34	7.98	0

[a] conformers are numbered according to their appearance during conformational search

Table S7. Total (E, in Hartree) and relative energies (ΔE, $\Delta\Delta G$, in kcal mol^{-1}), percentage populations (Pop.) and number of imaginary frequencies calculated at the M06L/6-31G(d,p) level for low-energy conformers of **6c**.

Conformer[a]	*E*	*ΔE*	Pop.	*ΔΔG*	Pop.	#ImFreq
3	-6676.40442	1.17	3.64	5.65		0
4	-6676.40542	0.55	10.54	2.31		0
6	-6676.40419	1.32	2.85	0	51.3	0
7	-6676.40455	1.09	4.19	5.12		0
8	-6676.40357	1.71	1.49	2.25		0
13	-6676.40333	1.86	1.15	5.57		0
21	-6676.40482	0.92	5.6	3.95		0
37	-6676.40569	0.37	14.07	3.42		0
45	-6676.40398	1.45	2.29	0.69	15.99	0
64	-6676.40225	2.54		0.76	14.14	0
65	-6676.40629	0	26.47	5.38		0
70	-6676.40327	1.89	1.08	3.56		0
80	-6676.40446	1.15	3.8	2.07		0
99	-6676.40615	0.09	22.83	0.6	18.57	0

[a] conformers are numbered according to their appearance during conformational search

Table S8. Total (E, in Hartree) and relative energies (ΔE, $\Delta\Delta G$, in kcal mol^{-1}), percentage populations (Pop.) and number of imaginary frequencies calculated at the B3LYP/6-31G(d) level for low-energy conformers of **6f**.

Conformer[a]	*E*	*ΔE*	Pop.	*ΔΔG*	Pop.	#ImFreq
8	-8741.6415	0	51.57	0.58	27.37	0
23	-8741.64144	0.04	48.43	0	72.63	0

[a] conformers are numbered according to their appearance during conformational search

Table S9. Total (E, in Hartree) and relative energies (ΔE, $\Delta\Delta G$, in kcal mol^{-1}), percentage populations (Pop.) and number of imaginary frequencies calculated at the B3LYP-GD3BJ/6-31G(d) level for low-energy conformers of **6f**.

Conformer[a]	*E*	*ΔE*	Pop.	*ΔΔG*	Pop.	#ImFreq
23	-8742.673102	0	100	0	100	0

[a] conformers are numbered according to their appearance during conformational search

Table S10. Total (E, in Hartree) and relative energies (ΔE, $\Delta\Delta G$, in kcal mol^{-1}), percentage populations (Pop.) and number of imaginary frequencies calculated at the M06L/6-31G(d) level for low-energy conformers of **6f**.

Conformer[a]	*E*	*ΔE*	Pop.	*ΔΔG*	Pop.	#ImFreq
23	-8740.873455	0	100	0	100	0

[a] conformers are numbered according to their appearance during conformational search

Table S11. Total (*E*, in Hartree) and relative energies (*ΔE*, *ΔΔG*, in kcal mol^{-1}), percentage populations (Pop.) and number of imaginary frequencies calculated at the B3LYP/6-311G(d,p) level for low-energy conformers of **6g**.

Conformer[a,b]	*E*	*ΔE*	Pop.	*ΔΔG*	Pop.	#ImFreq
sym_1	-11684.8833	1.02	1.2	3.57		0
sym_29	-11684.8822	1.71	0.38	4.12		0
sym_41	-11684.8841	0.48	3	4.05		0
sym_67	-11684.8824	1.58	0.47	2.66		0
sym_68	-11684.8822	1.68	0.4	4.01		0
sym_74	-11684.8846	0.19	4.9	5.25		0
sym_77	-11684.8822	1.71	0.38	3.81		0
sym_87	-11684.8834	0.96	1.34	1.16	3.06	0
sym_98	-11684.8834	0.97	1.33	1.49	1.78	0
2	-11684.8818	1.95	0.25	3.74		0
6	-11684.8847	0.12	5.56	0.3	13.23	0
8	-11684.8845	0.25	4.42	0.55	8.67	0
9	-11684.8829	1.26	0.81	3.59		0
10	-11684.8836	0.83	1.68	2.92		0
11	-11684.8849	0	6.79	0.43	10.61	0
12	-11684.8843	0.37	3.64	0	21.84	0
13	-11684.8844	0.29	4.13	1.19	2.93	0
14	-11684.8828	1.31	0.74	3.45		0
15.	-11684.8842	0.44	3.24	2.12		0
16	-11684.8827	1.37	0.67	3.59		0
17	-11684.8826	1.46	0.58	2.87		0
20	-11684.8823	1.64	0.42	4.96		0
22	-11684.8819	1.86	0.29	3.44		0
24	-11684.8822	1.69	0.39	3.77		0
25	-11684.8818	1.94	0.26	3.82		0
26	-11684.8824	1.57	0.48	5.04		0
27	-11684.883	1.18	0.92	3.05		0
28	-11684.8825	1.54	0.5	3.32		0
30	-11684.8823	1.65	0.42	3.77		0
31	-11684.8826	1.43	0.6	3.06		0
34	-11684.8831	1.11	1.04	3.85		0
36	-11684.8841	0.51	2.86	2.72		0
37	-11684.8819	1.88	0.28	3.15		0
40	-11684.8847	0.14	5.4	1.42	1.98	0
41	-11684.8824	1.55	0.5	2.9		0
44	-11684.8822	1.68	0.4	3.96		0
45	-11684.8829	1.24	0.83	3.19		0
46	-11684.882	1.8	0.32	3.75		0
48	-11684.8824	1.55	0.5	2.99		0
49	-11684.8837	0.78	1.82	2.02		0
52	-11684.883	1.2	0.9	3.4		0
53	-11684.8819	1.89	0.28	3.47		0
58	-11684.8819	1.88	0.29	2.83		0
59	-11684.8819	1.87	0.29	4.26		0
61	-11684.8841	0.49	2.96	2.06		0
62	-11684.8828	1.3	0.75	4.88		0
63	-11684.8833	0.99	1.28	3.11		0
64	-11684.8825	1.48	0.55	2.38		0
65	-11684.8848	0.08	5.97	1.37	2.14	0
66	-11684.8839	0.61	2.41	1.35	2.23	0
72	-11684.8838	0.7	2.1	2.27		0
73	-11684.8831	1.14	0.99	3.57		0
75	-11684.8838	0.72	2.02	2.27		0
76	-11684.882	1.82	0.31	3.98		0
77	-11684.8828	1.34	0.71	3.47		0
78	-11684.8844	0.32	3.96	0.42	10.68	0
79	-11684.8823	1.64	0.42	3.97		0
82	-11684.8824	1.57	0.48	3.59		0
83	-11684.882	1.81	0.32	3.92		0

84	-11684.8832	1.09	1.08	3.2		0
87	-11684.8824	1.6	0.46	4.45		0
89	-11684.8837	0.77	1.86	1.45	1.9	0
90	-11684.882	1.8	0.33	4.3		0
92	-11684.8841	0.53	2.77	0.4	11.06	0
94	-11684.8828	1.3	0.75	1.89	0.9	0
95	-11684.8836	0.85	1.62	0.67	7	0

[a] conformers are numbered according to their appearance during conformational search; [b] prefix “sym_” denotes symmetrical conformer

Table S12. Total (*E*, in Hartree) and relative energies (*ΔE*, *ΔΔG*, in kcal mol^{-1}), percentage populations (Pop.) and number of imaginary frequencies calculated at the B3LYP-GD3BJ/6-311G(d,p) level for low-energy conformers of **6g**.

Conformer[a,b]	*E*	*ΔE*	Pop.	*ΔΔG*	Pop.	#ImFreq
sym_15	-11685.2862	3.15		1.52	5.14	0
sym_66	-11685.2907	3.52		1.34	7	0
5	-11685.2849	2.82		1.16	9.49	0
7	-11685.2861	0	100	0	67.5	0
18	-11685.2862	3.64		1.43	6.05	0
22	-11685.2907	2.89		1.56	4.81	0

[a] conformers are numbered according to their appearance during conformational search; [b] prefix “sym_” denotes symmetrical conformer

Table S13. Total (*E*, in Hartree) and relative energies (*ΔE*, *ΔΔG*, in kcal mol^{-1}), percentage populations (Pop.) and number of imaginary frequencies calculated at the M06L/6-311G(d,p) level for low-energy conformers of **6g**.

Conformer[a,b]	*E*	*ΔE*	Pop.	*ΔΔG*	Pop.	#ImFreq
sym_61.	-11684.1069	1.1	13.09	1.25	4.01	0
7	-11684.1087	0	83.51	0.23	22.5	0
8	-11684.1029	3.62		0.12	27.26	0
18	-11684.1045	2.63		1.11	5.12	0
22	-11684.1057	1.9	3.4	0	33.13	0
87	-11684.1039	2.98		1.87	1.42	0
90	-11684.1033	3.37		0.96	6.56	0

[a] conformers are numbered according to their appearance during conformational search; [b] prefix “sym_” denotes symmetrical conformer

Table S14. Total (*E*, in Hartree) and relative energies (*ΔE*, *ΔΔG*, in kcal mol^{-1}), percentage populations (Pop.) and number of imaginary frequencies calculated at the B3LYP/6-311G(d,p) level for low-energy conformers of **6h**.

Conformer[a,b]	*E*	*ΔE*	Pop.	*ΔΔG*	Pop.	#ImFreq
sym_1	-3964.26719	1.38	0.75	2.22		0
sym_29	-3964.26654	1.79	0.37	2.52		0
sym_41	-3964.26913	0.16	5.85	3.19		0
sym_45	-3964.26911	0.17	5.75	2.35		0
sym_67	-3964.26651	1.81	0.36	1.3	1.26	0
sym_68	-3964.26637	1.89	0.32	3.19		0
sym_74	-3964.26795	0.9	1.67	3.4		0
sym_77	-3964.26633	1.92	0.3	2.75		0
sym_87	-3964.26704	1.47	0.64	0.59	4.18	0
sym_98	-3964.26692	1.55	0.56	0.57	4.36	0
2	-3964.26688	1.58	0.54	3.27		0
6	-3964.26939	0	7.69	0.49	4.95	0
8	-3964.26825	0.71	2.31	0.26	7.28	0

9	-3964.26695	1.53	0.58	1.99	0.39	0
10	-3964.2675	1.18	1.04	2.26		0
11	-3964.26883	0.35	4.27	0.82	2.84	0
12	-3964.26898	0.25	5.01	0.49	4.98	0
13	-3964.26839	0.63	2.67	1.03	1.99	0
14	-3964.26708	1.45	0.67	2.27		0
15	-3964.26815	0.78	2.07	1.08	1.84	0
16	-3964.26682	1.61	0.51	2.39		0
17	-3964.26745	1.22	0.98	2		0
19	-3964.26645	1.84	0.34	2.81		0
20	-3964.26746	1.21	1	2.55		0
21	-3964.26738	1.26	0.92	3.01		0
22	-3964.26748	1.2	1.02	2.18		0
23	-3964.26738	1.26	0.92	1.72	0.62	0
24	-3964.2678	1	1.43	1.37	1.11	0
25	-3964.26637	1.89	0.32	2.22		0
26	-3964.26799	0.88	1.74	2.99		0
27	-3964.26706	1.46	0.65	1.74	0.6	0
28	-3964.26671	1.68	0.45	1.75	0.59	0
30	-3964.26694	1.54	0.57	2.25		0
31	-3964.26689	1.57	0.55	1.86	0.49	0
33	-3964.26714	1.41	0.71	1.92	0.44	0
34	-3964.26779	1	1.41	2.76		0
36	-3964.26868	0.44	3.65	1.24	1.4	0
38	-3964.26757	1.14	1.12	2.8		0
40	-3964.26837	0.64	2.62	0.94	2.31	0
42	-3964.26641	1.87	0.33	2.21		0
44	-3964.26723	1.35	0.78	2.18		0
45	-3964.26738	1.26	0.92	2.18		0
46	-3964.26653	1.79	0.37	2.47		0
48	-3964.26713	1.42	0.7	1.42	1.03	0
49	-3964.26828	0.7	2.37	1.04	1.94	0
52	-3964.26777	1.02	1.38	1.75	0.59	0
53	-3964.26677	1.65	0.48	2.71		0
57	-3964.26714	1.41	0.71	1.81	0.53	0
58	-3964.26704	1.48	0.64	1.14	1.65	0
59	-3964.26719	1.38	0.75	2.16		0
60	-3964.26679	1.63	0.49	3.66		0
61	-3964.26788	0.95	1.55	1.93	0.44	0
62	-3964.26735	1.28	0.89	2.4		0
63	-3964.26743	1.23	0.96	2.7		0
64	-3964.26702	1.49	0.62	2.21		0
65	-3964.26795	0.9	1.68	0.88	2.55	0
66	-3964.2682	0.75	2.18	0.85	2.7	0
69	-3964.26647	1.83	0.35	1.62	0.73	0
72	-3964.26775	1.03	1.36	1.34	1.17	0
74	-3964.26664	1.72	0.42	1.59	0.77	0
75	-3964.26775	1.03	1.36	1.61	0.75	0
77	-3964.26723	1.36	0.78	2.74		0
78	-3964.26837	0.64	2.61	0.15	8.78	0
79	-3964.26773	1.04	1.32	1.99	0.39	0
82	-3964.26683	1.6	0.51	2.52		0
83	-3964.26646	1.84	0.34	1.36	1.13	0
84	-3964.26668	1.7	0.44	2.21		0
86	-3964.26772	1.04	1.32	1.48	0.93	0
87	-3964.26802	0.86	1.81	1.63	0.73	0
89	-3964.26728	1.32	0.83	0.47	5.09	0
90	-3964.26653	1.8	0.37	2.52		0
91	-3964.26711	1.43	0.69	1.24	1.39	0
92	-3964.26778	1.01	1.4	0.17	8.49	0
94	-3964.26718	1.39	0.74	0.46	5.24	0
95	-3964.26764	1.1	1.21	0	11.33	0

[a] conformers are numbered according to their appearance during conformational search; [b] prefix “sym_” denotes symmetrical conformer

Table S15. Total (*E*, in Hartree) and relative energies (*ΔE*, *ΔΔG*, in kcal mol^{-1}), percentage populations (Pop.) and number of imaginary frequencies calculated at the B3LYP-GD3BJ/6-311G(d,p) level for low-energy conformers of **6h**.

Conformer[a,b]	*E*	*ΔE*	Pop.	*ΔΔG*	Pop.	#ImFreq
sym_15	-3964.64529	3.44		1.52	3.48	0
sym_66	-3964.64431	4.05		1.45	3.87	0
2	-3964.65077	0	100	0	45.1	0
5	-3964.64463	3.85		1.19	6.07	0
6	-3964.64544	3.34		1.19	6	0
22	-3964.64613	2.91		1.25	5.43	0
79	-3964.64458	3.88		1	8.33	0
87	-3964.64616	2.89		0.54	18.13	0
98	-3964.64597	3.01		1.5	3.58	0

[a] conformers are numbered according to their appearance during conformational search; [b] prefix “sym_” denotes symmetrical conformer

Table S16. Total (*E*, in Hartree) and relative energies (*ΔE*, *ΔΔG*, in kcal mol^{-1}), percentage populations (Pop.) and number of imaginary frequencies calculated at the M06L/6-311G(d,p) level for low-energy conformers of **6h**.

Conformer[a,b]	*E*	*ΔE*	Pop.	*ΔΔG*	Pop.	#ImFreq
sym_15	-3963.86447	1.95	0.84	1.98	2.09	0
sym_61	-3963.86757	0	22.48	2.41		0
2	-3963.86602	0.97	4.36	0.77	16.3	0
5	-3963.86461	1.86	0.98	3.46		0
6	-3963.86651	0.67	7.28	1.34	6.17	0
7	-3963.86735	0.14	17.78	1.01	10.83	0
8	-3963.86325	2.71		1.77	2.97	0
20	-3963.86586	1.07	3.68	2.27		0
22	-3963.86653	0.65	7.47	2.33		0
60	-3963.86606	0.95	4.52	3.14		0
62	-3963.86584	1.09	3.58	0	59.5	0
79	-3963.86448	1.94	0.85	3.14		0
81	-3963.86461	1.86	0.98	4.12		0
87	-3963.8664	0.74	6.49	1.97	2.14	0
88	-3963.86532	1.41	2.08	3.58		0
98	-3963.86729	0.18	16.64	2.51		0

[a] conformers are numbered according to their appearance during conformational search; [b] prefix “sym_” denotes symmetrical conformer

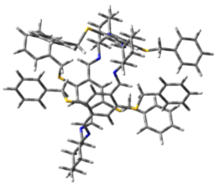

conf. 16

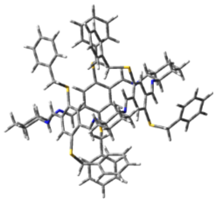

conf. 20

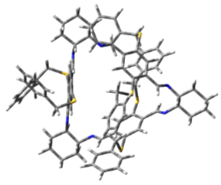

conf. 22

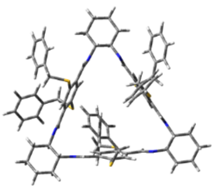

conf. 27

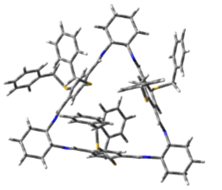

conf. 31

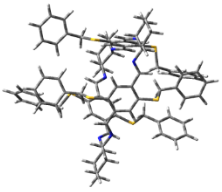

conf. 39

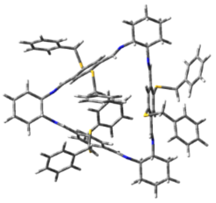

conf. 43

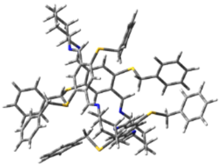

conf. 49

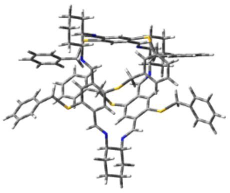

conf. 50

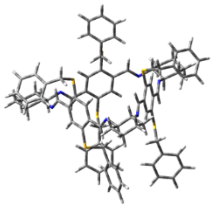

conf. 63

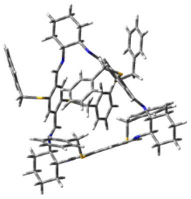

conf. 68

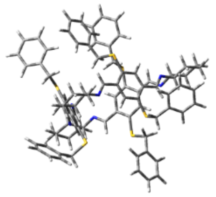

conf. 72

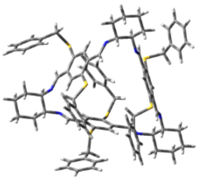

conf. 73

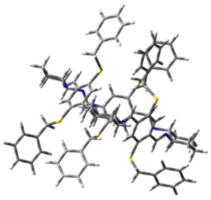

conf. 74

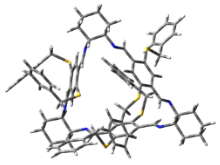

conf. 75

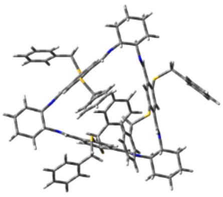

conf. 76

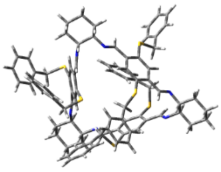

conf. 83

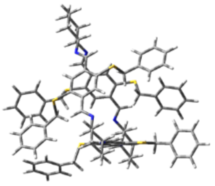

conf. 86

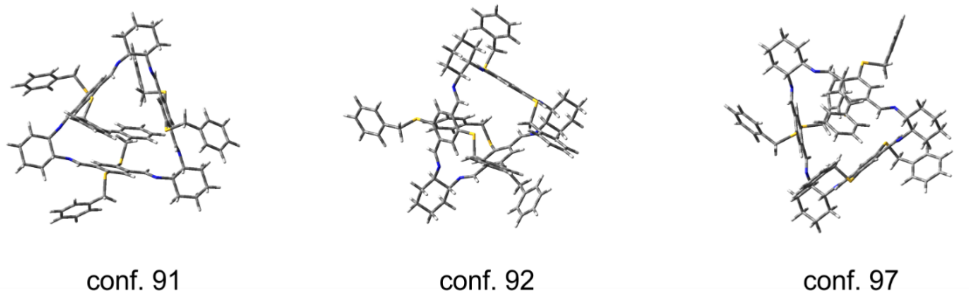

Figure S14. Structures of the low-energy conformers of **6a**, calculated at the B3LYP/6-311G(d,p) level.

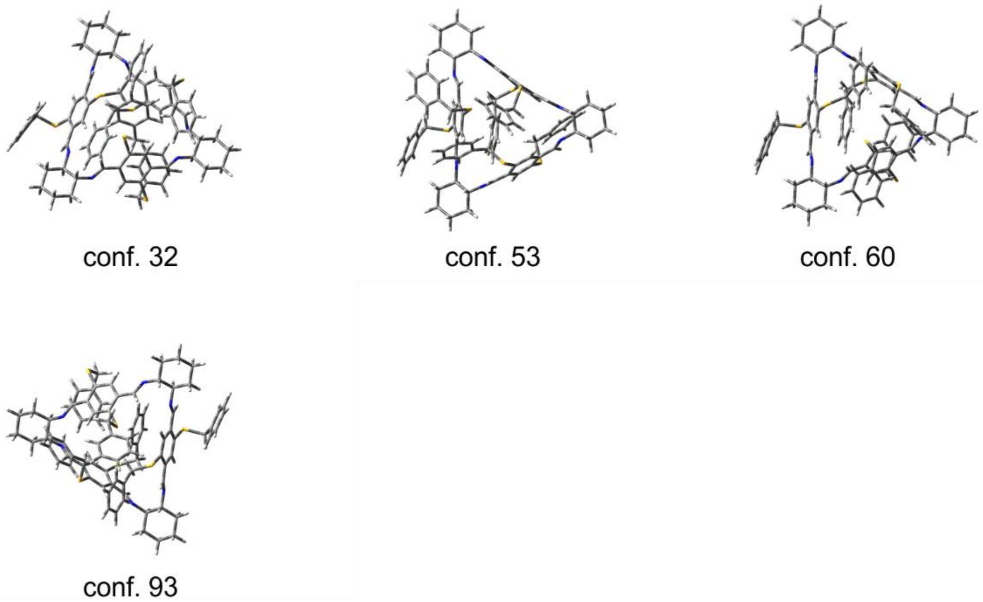

Figure S15. Structures of the low-energy conformers of **6a**, calculated at the B3LYP-GD3BJ/6-311G(d,p) level.

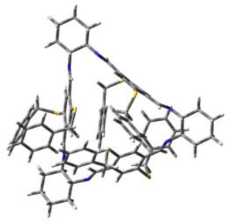

conf. 5

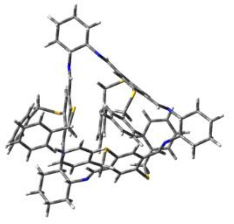

conf. 7

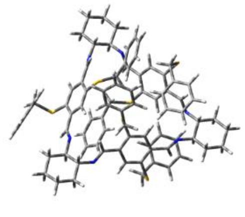

conf. 32

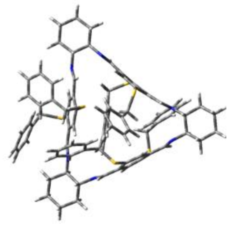

conf. 53

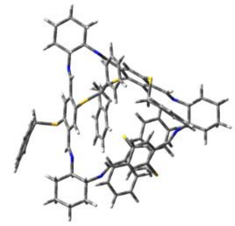

conf. 60

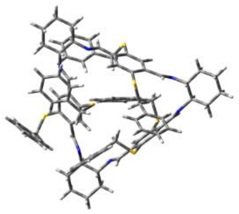

conf. 81

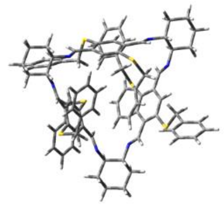

conf. 82

Figure S16. Structures of the low-energy conformers of **6a**, calculated at the M06L/6-311G(d,p) level.

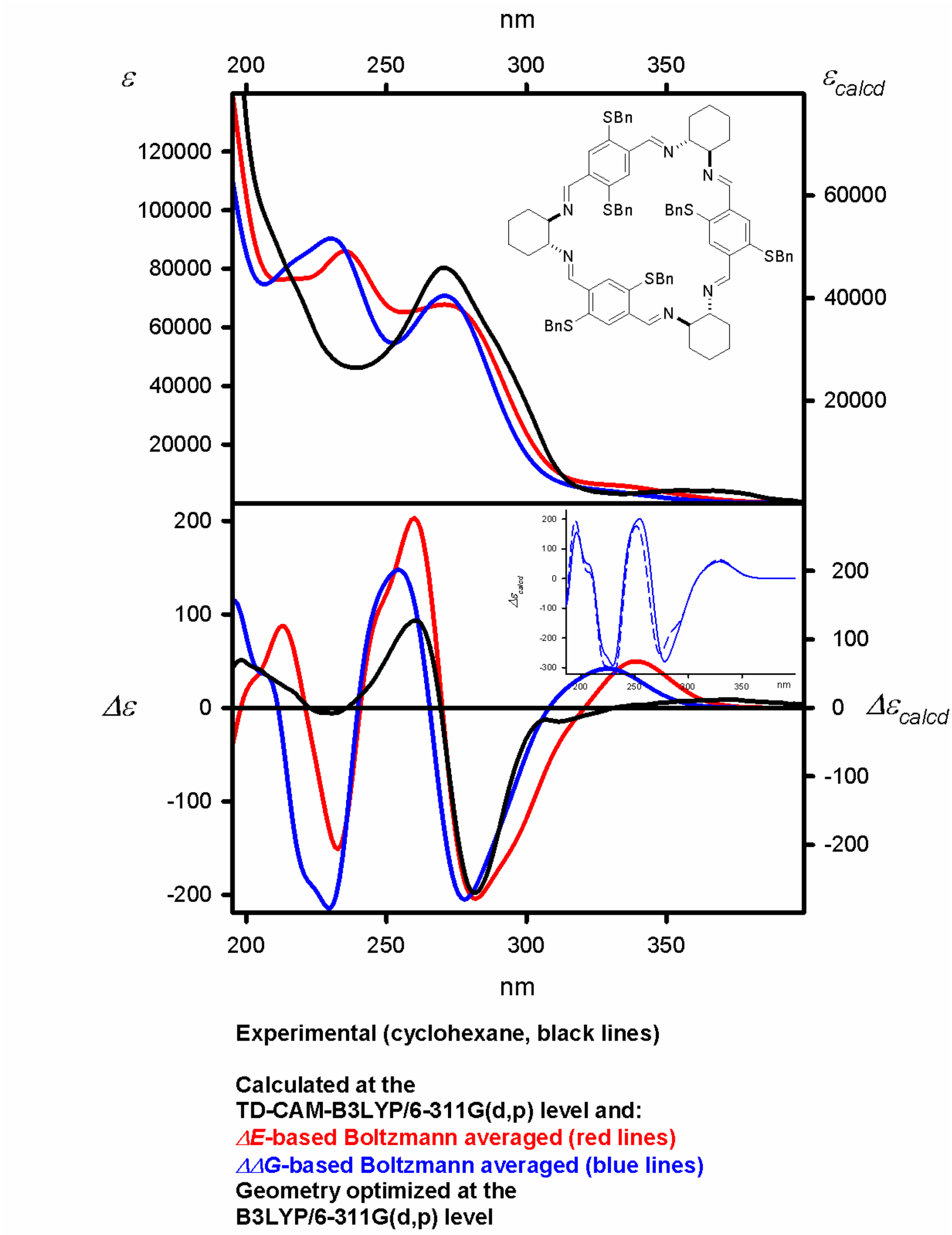

Figure S17. UV (upper panel) and ECD (lower panel) spectra of **6a** measured in cyclohexane (solid black lines) and calculated at the TD-CAM-B3LYP/6-311G(d,p) level for geometries optimized at the B3LYP/6-311G(d,p) level. The calculated ECD spectra were Boltzmann-averaged based on *ΔE* (red lines) and *ΔΔG* values (blue lines). Wavelengths were corrected to match the experimental UV maxima. The insert shows the comparison between the ECD spectra calculated for the lowest energy conformer of a given compound (dashed blue lines) and the *ΔΔ*G-based and Boltzmann averaged (solid blue lines). *Δε* values are given in mol^{-1} cm^{-1} dm^{3}.

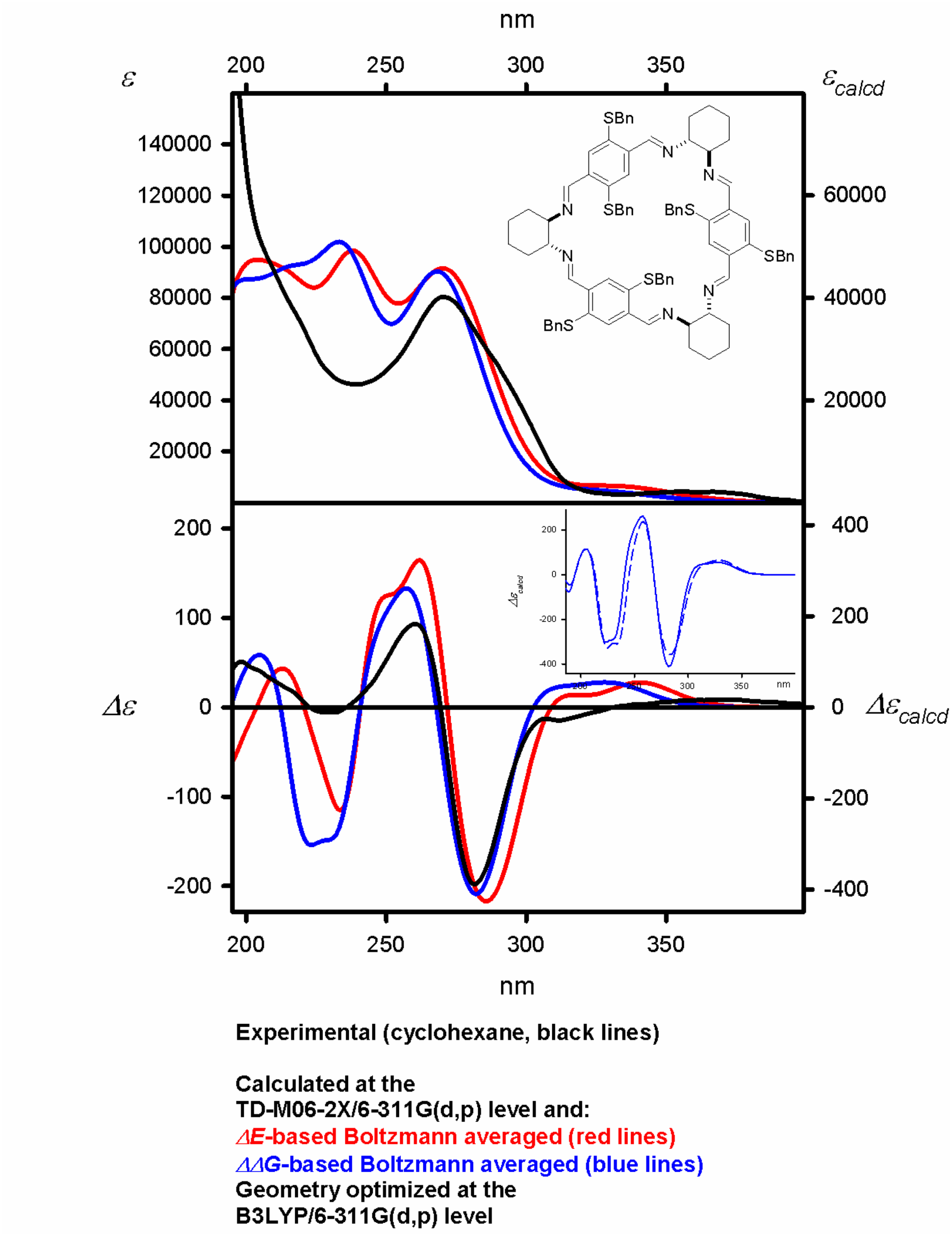

Figure S18. UV (upper panel) and ECD (lower panel) spectra of **6a** measured in cyclohexane (solid black lines) and calculated at the TD-M06-2X/6-311G(d,p) level for geometries optimized at the B3LYP/6-311G(d,p) level. The calculated ECD spectra were Boltzmann-averaged based on ΔE (red lines) and $\Delta\Delta G$ values (blue lines). Wavelengths were corrected to match the experimental UV maxima. The insert shows the comparison between the ECD spectra calculated for the lowest energy conformer of a given compound (dashed blue lines) and the $\Delta\Delta$G-based and Boltzmann averaged (solid blue lines). $\Delta\varepsilon$ values are given in mol^{-1} cm^{-1} dm^{3}.

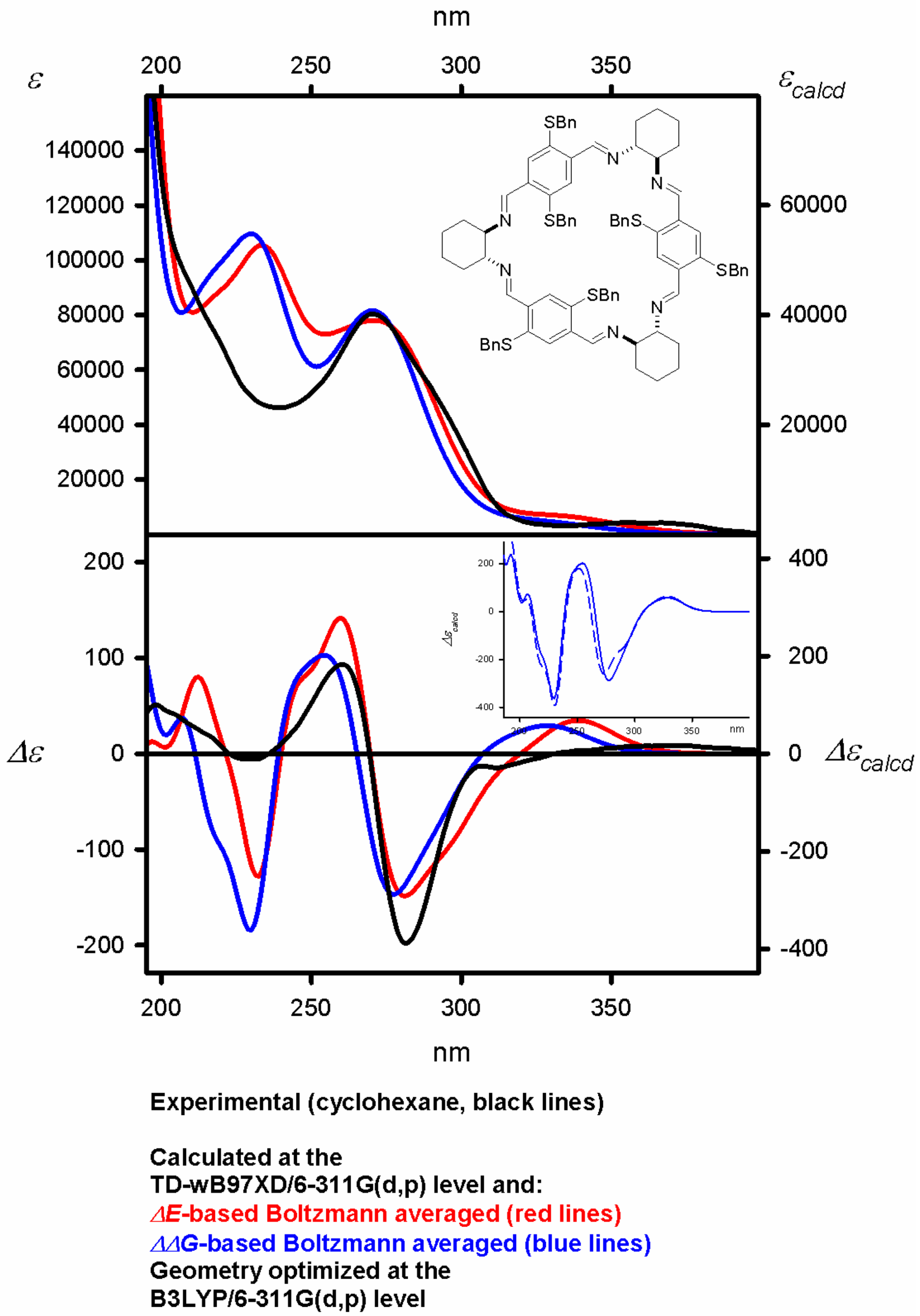

Figure S19. UV (upper panel) and ECD (lower panel) spectra of **6a** measured in cyclohexane (solid black lines) and calculated at the TD-wB97XD/6-311G(d,p) level for geometries optimized at the B3LYP/6-311G(d,p) level. The calculated ECD spectra were Boltzmann-averaged based on ΔE (red lines) and $\Delta\Delta G$ values (blue lines). Wavelengths were corrected to match the experimental UV maxima. The insert shows the comparison between the ECD spectra calculated for the lowest energy conformer of a given compound (dashed blue lines) and the $\Delta\Delta$G-based and Boltzmann averaged (solid blue lines). $\Delta\varepsilon$ values are given in mol^{-1} cm^{-1} dm^{3}.

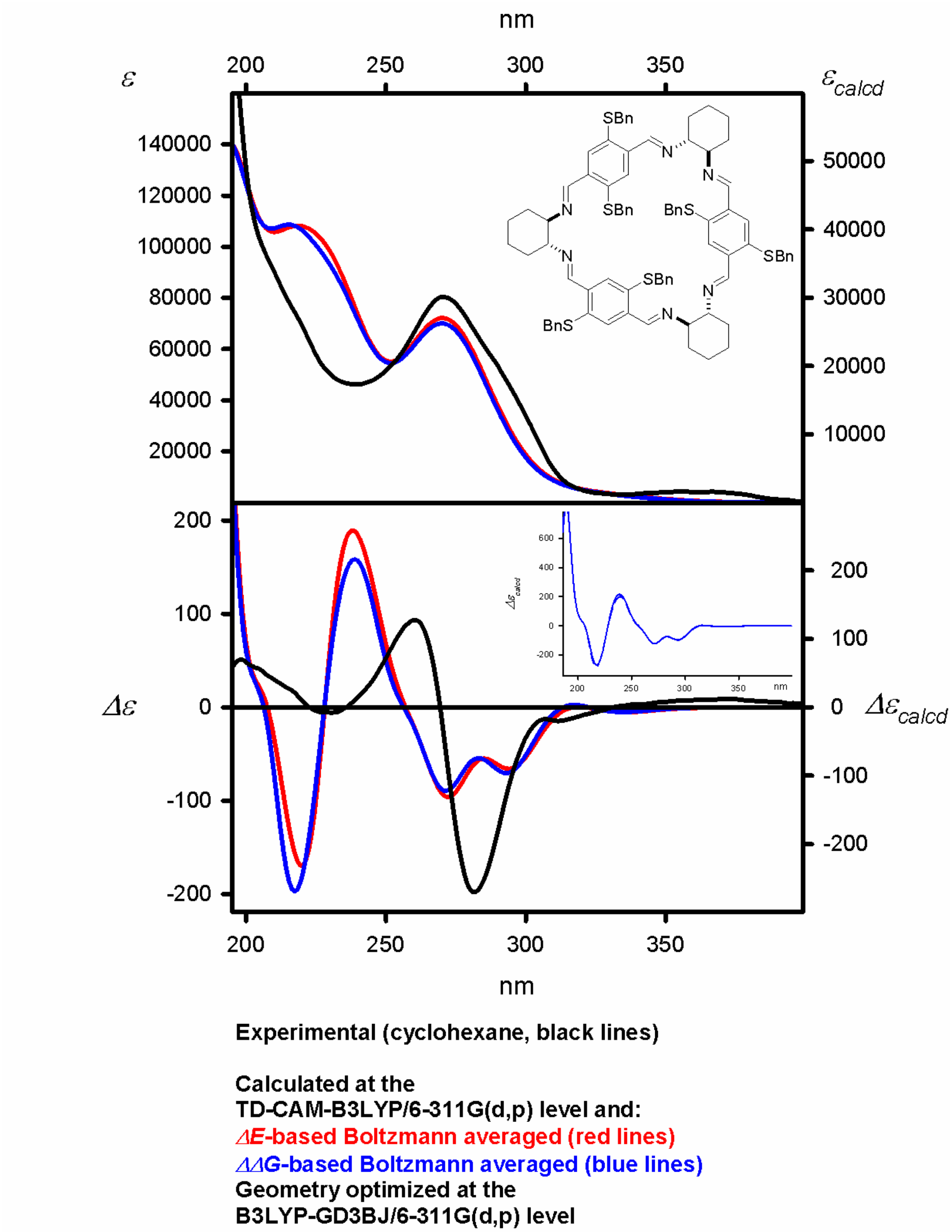

Figure S20. UV (upper panel) and ECD (lower panel) spectra of **6a** measured in cyclohexane (solid black lines) and calculated at the TD-CAM-B3LYP/6-311G(d,p) level for geometries optimized at the B3LYP-GD3BJ/6-311G(d,p) level. The calculated ECD spectra were Boltzmann-averaged based on ΔE (red lines) and $\Delta\Delta G$ values (blue lines). Wavelengths were corrected to match the experimental UV maxima. The insert shows the comparison between the ECD spectra calculated for the lowest energy conformer of a given compound (dashed blue lines) and the $\Delta\Delta$G-based and Boltzmann averaged (solid blue lines). $\Delta\varepsilon$ values are given in mol^{-1} cm^{-1} dm^{3}.

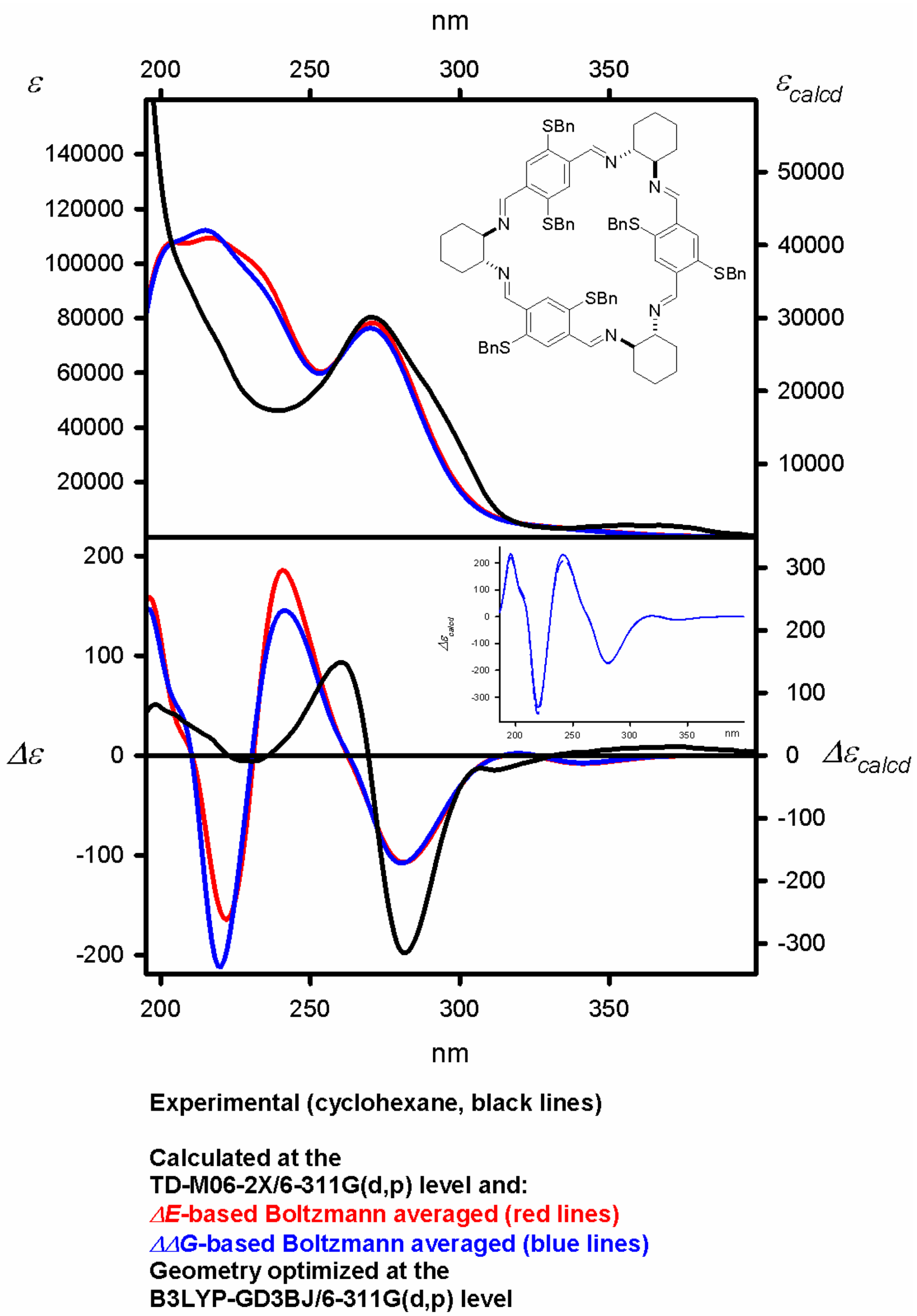

Figure S21. UV (upper panel) and ECD (lower panel) spectra of **6a** measured in cyclohexane (solid black lines) and calculated at the TD-M06-2X/6-311G(d,p) level for geometries optimized at the B3LYP-GD3BJ/6-311G(d,p) level. The calculated ECD spectra were Boltzmann-averaged based on *ΔE* (red lines) and *ΔΔG* values (blue lines). Wavelengths were corrected to match the experimental UV maxima. The insert shows the comparison between the ECD spectra calculated for the lowest energy conformer of a given compound (dashed blue lines) and the *ΔΔ*G-based and Boltzmann averaged (solid blue lines). *Δε* values are given in $mol^{-1}\ cm^{-1}\ dm^{3}$.

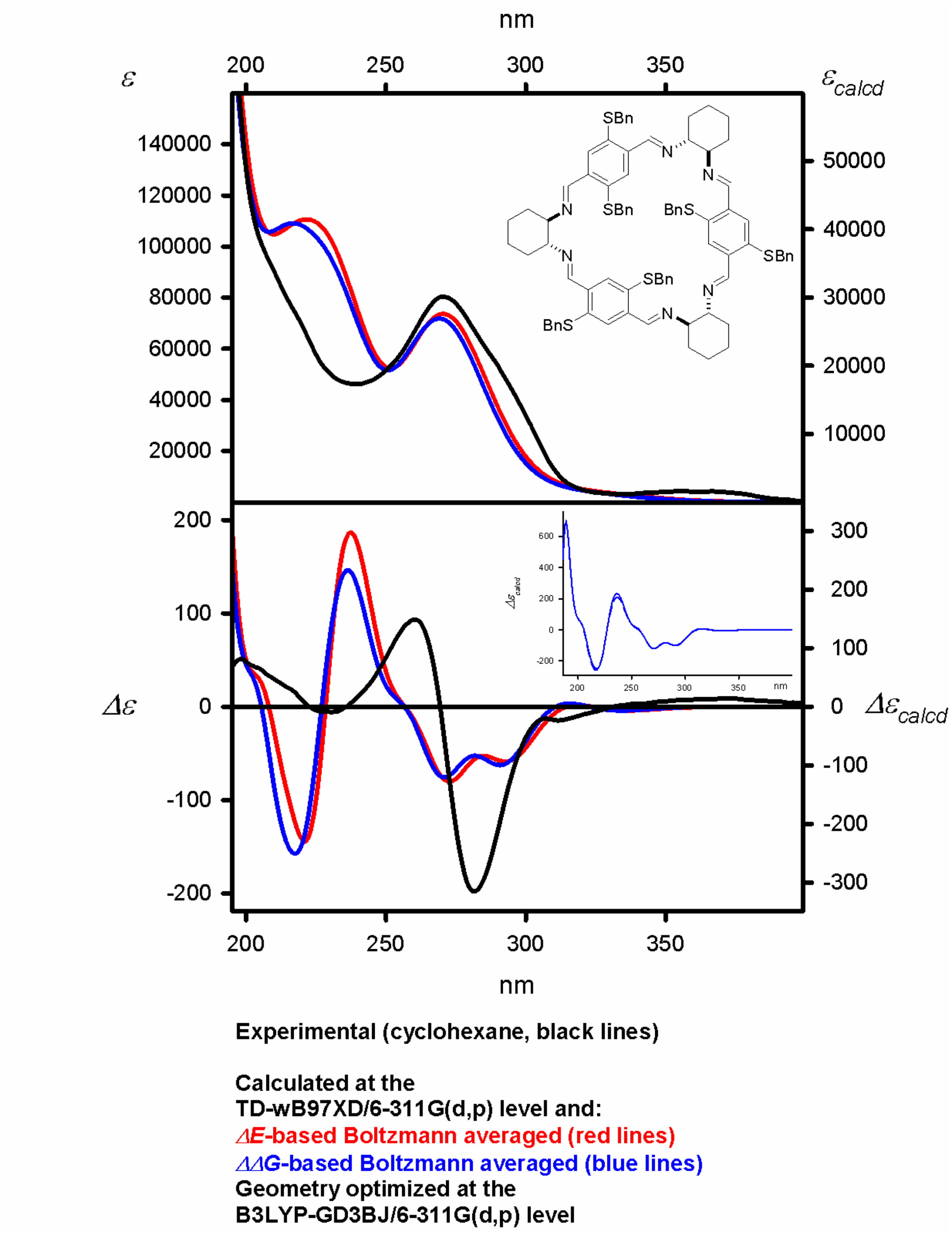

Figure S22. UV (upper panel) and ECD (lower panel) spectra of **6a** measured in cyclohexane (solid black lines) and calculated at the TD-wB97XD/6-311G(d,p) level for geometries optimized at the B3LYP-GD3BJ/6-311G(d,p) level. The calculated ECD spectra were Boltzmann-averaged based on *ΔE* (red lines) and *ΔΔG* values (blue lines). Wavelengths were corrected to match the experimental UV maxima. The insert shows the comparison between the ECD spectra calculated for the lowest energy conformer of a given compound (dashed blue lines) and the *ΔΔ*G-based and Boltzmann averaged (solid blue lines). *Δε* values are given in mol^{-1} cm^{-1} dm^{3}.

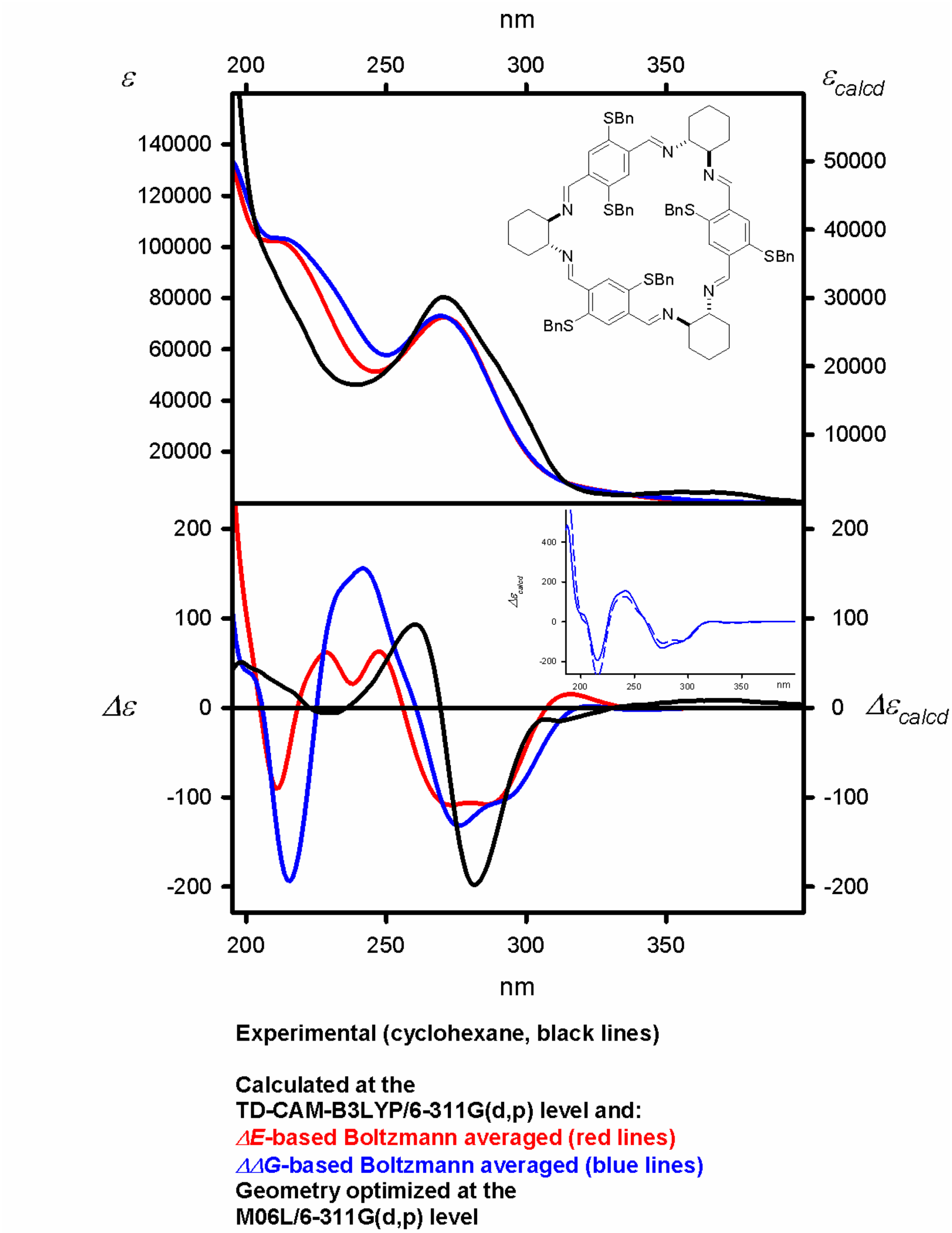

Figure S23. UV (upper panel) and ECD (lower panel) spectra of **6a** measured in cyclohexane (solid black lines) and calculated at the TD-CAM-B3LYP/6-311G(d,p) level for geometries optimized at the M06L/6-311G(d,p) level. The calculated ECD spectra were Boltzmann-averaged based on *ΔE* (red lines) and *ΔΔG* values (blue lines). Wavelengths were corrected to match the experimental UV maxima. The insert shows the comparison between the ECD spectra calculated for the lowest energy conformer of a given compound (dashed blue lines) and the *ΔΔ*G-based and Boltzmann averaged (solid blue lines *Δε* values are given in mol^{-1} cm^{-1} dm^{3}.

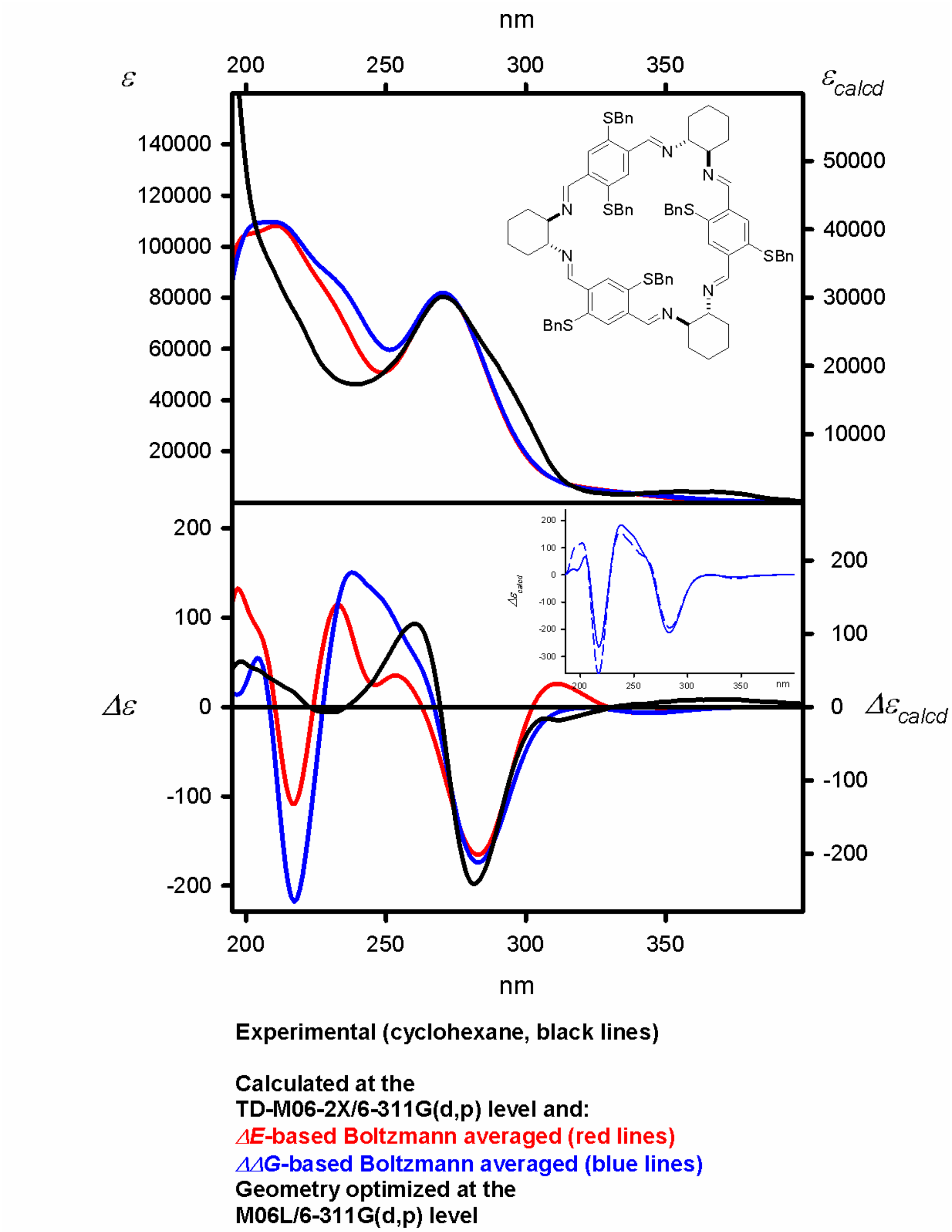

Figure S24. UV (upper panel) and ECD (lower panel) spectra of **6a** measured in cyclohexane (solid black lines) and calculated at the TD-M06-2X/6-311G(d,p) level for geometries optimized at the M06L/6-311G(d,p) level. The calculated ECD spectra were Boltzmann-averaged based on ΔE (red lines) and $\Delta\Delta G$ values (blue lines). Wavelengths were corrected to match the experimental UV maxima. The insert shows the comparison between the ECD spectra calculated for the lowest energy conformer of a given compound (dashed blue lines) and the $\Delta\Delta$G-based and Boltzmann averaged (solid blue lines). $\Delta\varepsilon$ values are given in mol^{-1} cm^{-1} dm^{3}.

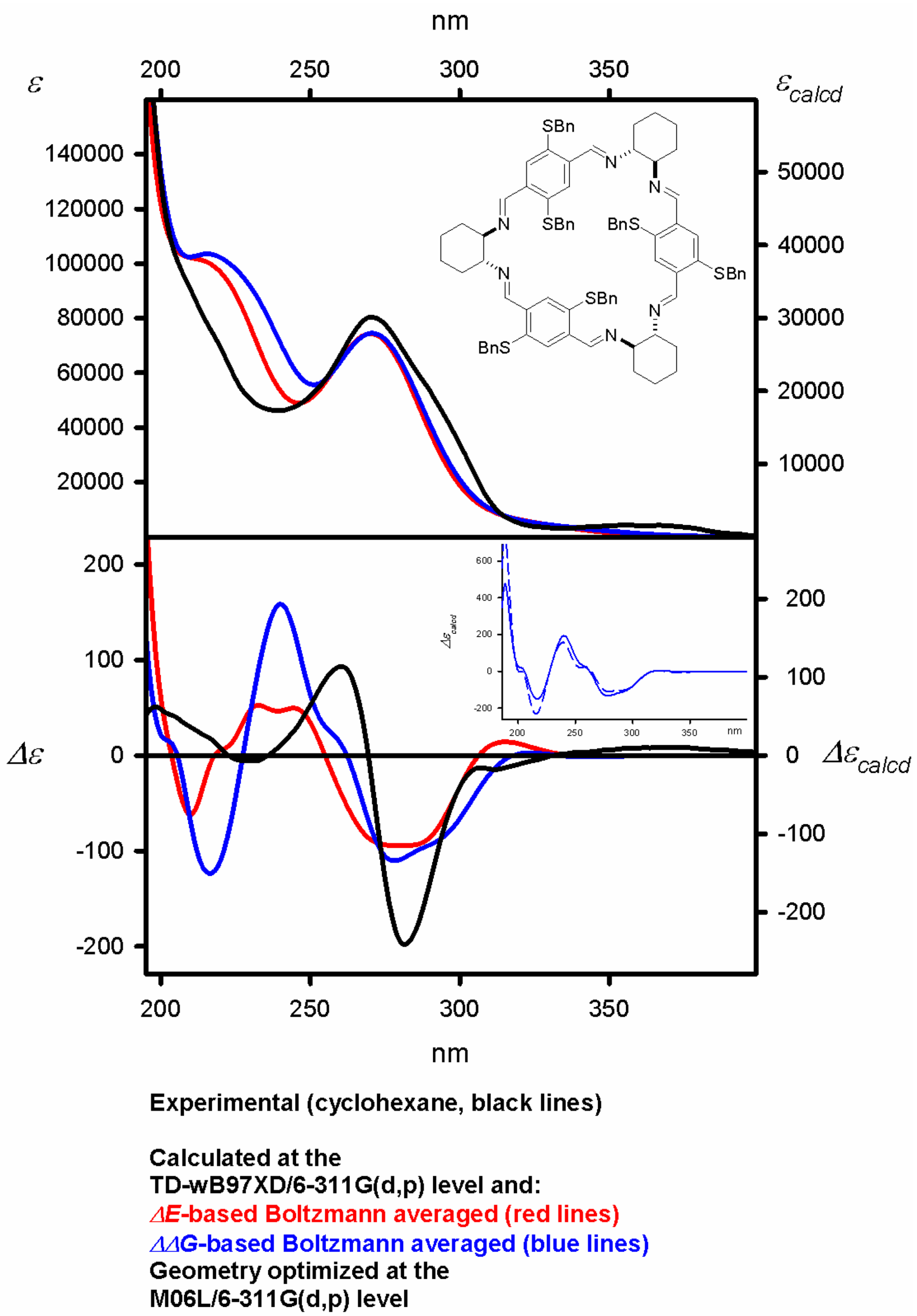

Figure S25. UV (upper panel) and ECD (lower panel) spectra of **6a** measured in cyclohexane (solid black lines) and calculated at the TD-wB97XD/6-311G(d,p) level for geometries optimized at the M06L/6-311G(d,p) level. The calculated ECD spectra were Boltzmann-averaged based on ΔE (red lines) and $\Delta\Delta G$ values (blue lines). Wavelengths were corrected to match the experimental UV maxima. The insert shows the comparison between the ECD spectra calculated for the lowest energy conformer of a given compound (dashed blue lines) and the $\Delta\Delta$G-based and Boltzmann averaged (solid blue lines). $\Delta\varepsilon$ values are given in mol^{-1} cm^{-1} dm^{3}.

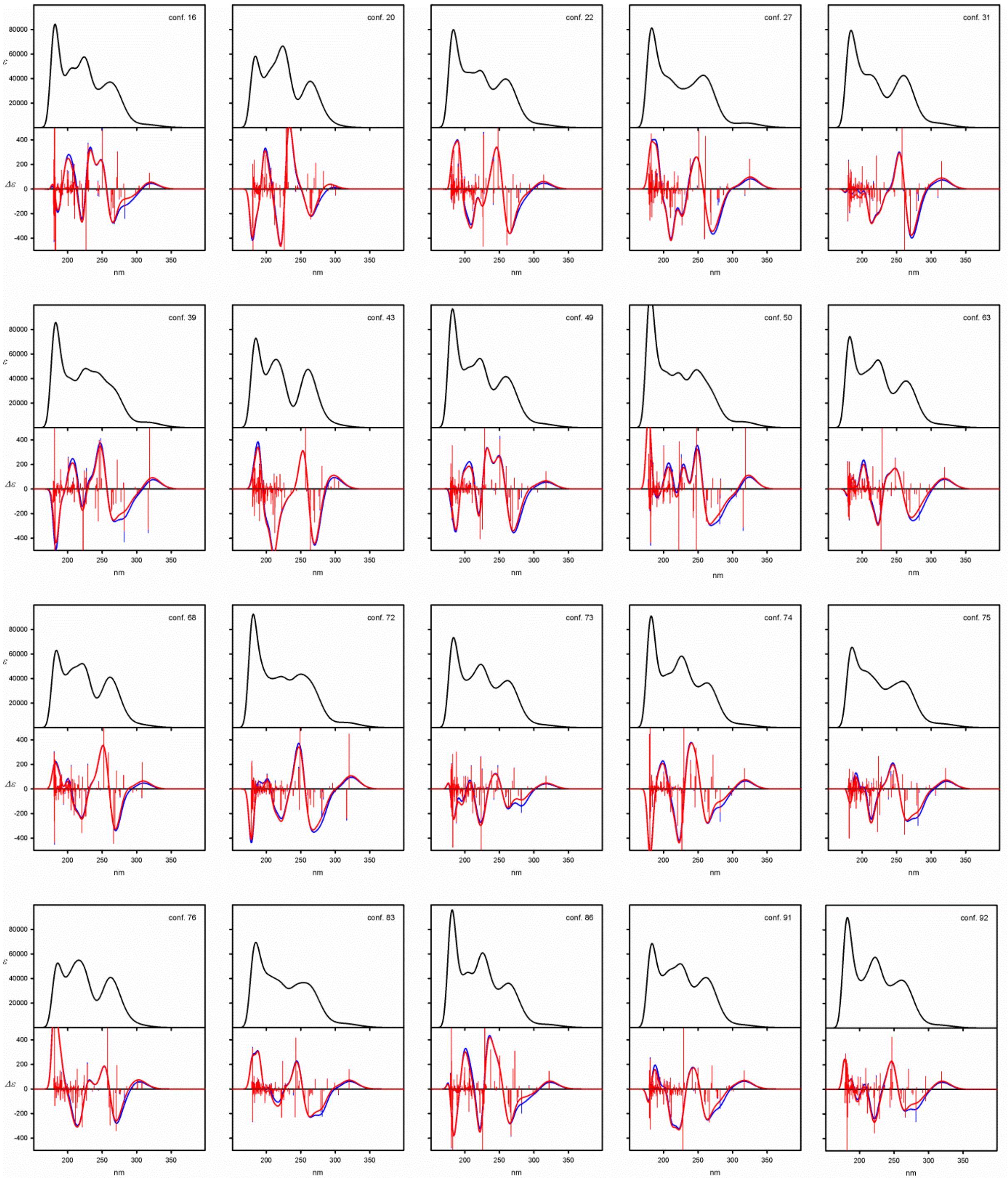

Figure S26. UV (upper panels) and ECD (lower panels) spectra calculated at the TD-CAM-B3LYP/6-311G(d,p) level for individual low-energy conformers of **6a**. Wavelengths were not corrected. Geometries were optimized at the B3LYP/6-311G(d,p) level. $\Delta\varepsilon$ values are given in mol^{-1} cm^{-1} dm^{3}.

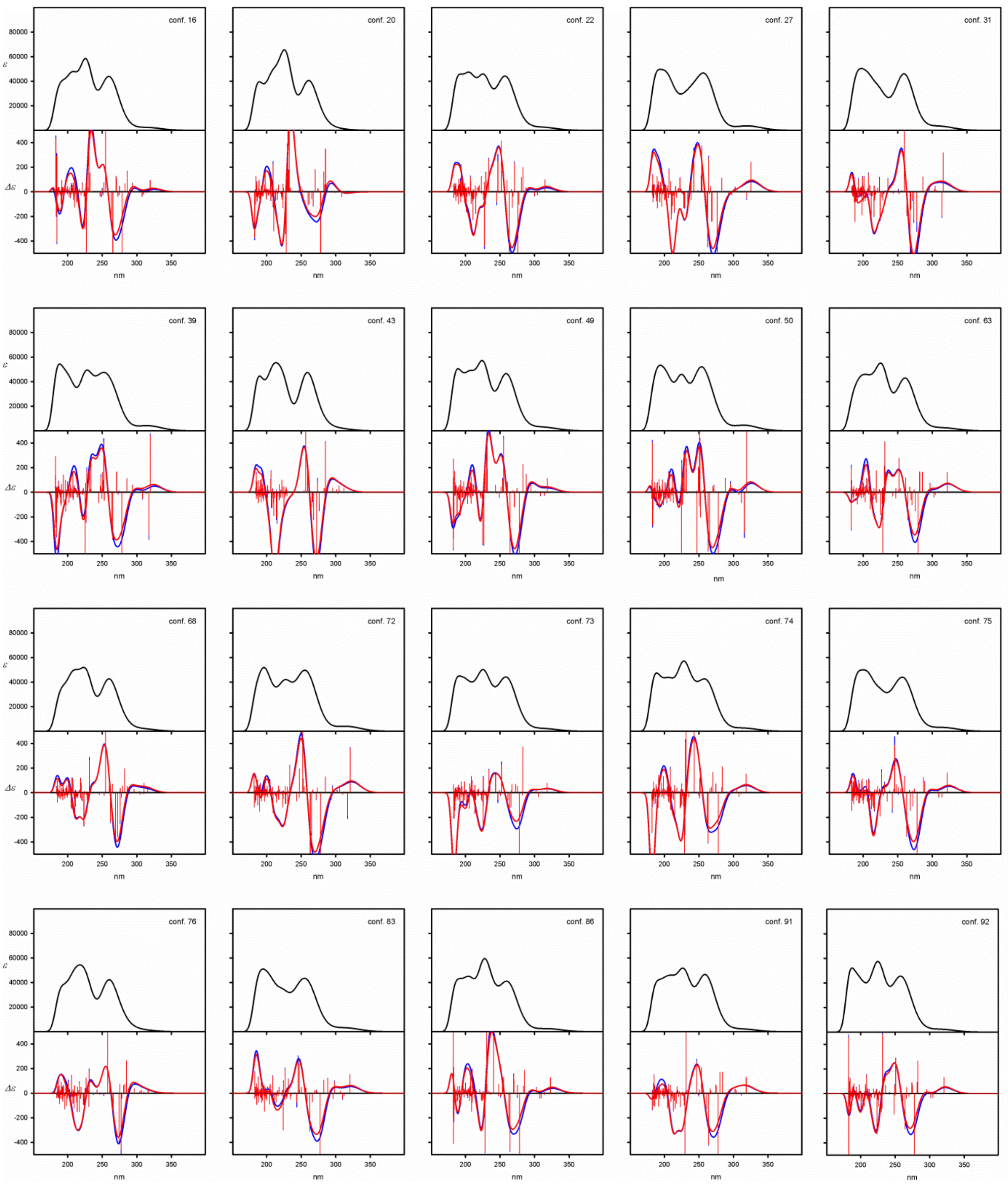

Figure S27. UV (upper panels) and ECD (lower panels) spectra calculated at the TD-M06-2X/6-311G(d,p) level for individual low-energy conformers of **6a**. Wavelengths were not corrected. Geometries were optimized at the B3LYP/6-311G(d,p) level. $\Delta\varepsilon$ values are given in mol^{-1} cm^{-1} dm^{3}.

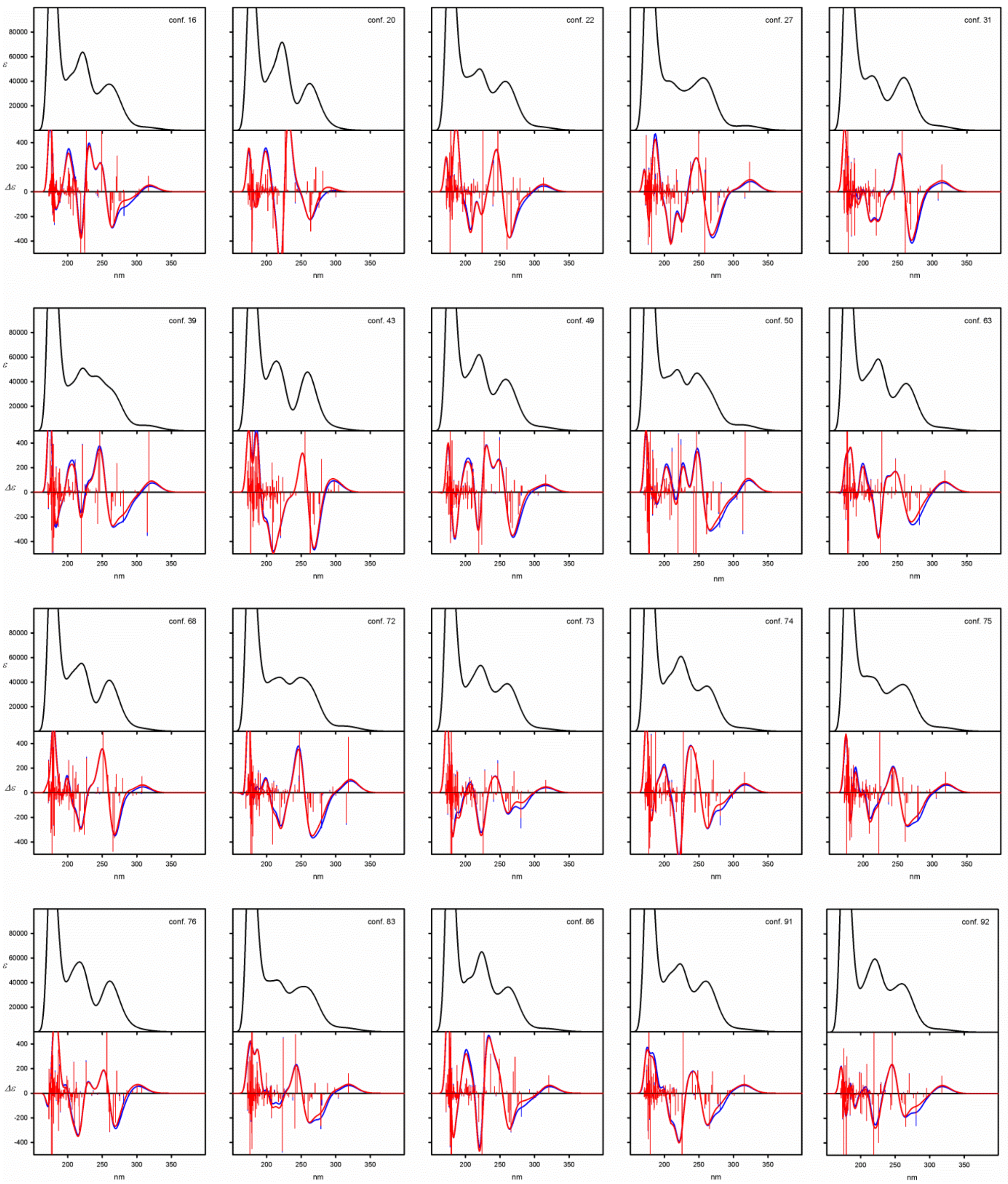

Figure S28. UV (upper panels) and ECD (lower panels) spectra calculated at the TD-wB97XD/6-311G(d,p) level for individual low-energy conformers of **6a**. Wavelengths were not corrected. Geometries were optimized at the B3LYP/6-311G(d,p) level. $\Delta\varepsilon$ values are given in mol^{-1} cm^{-1} dm^{3}.

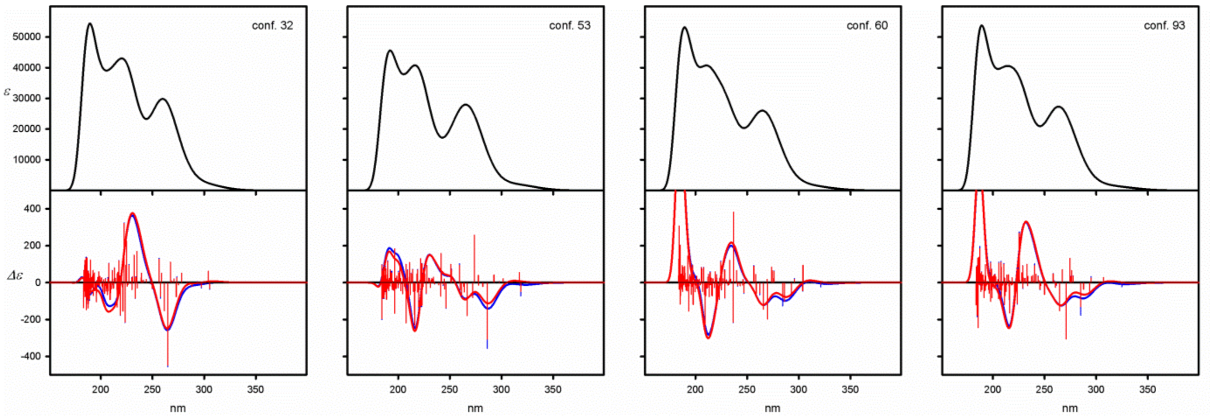

Figure S29. UV (upper panels) and ECD (lower panels) spectra calculated at the TD-CAM-B3LYP/6-311G(d,p) level for individual low-energy conformers of **6a**. Wavelengths were not corrected. Geometries were optimized at the B3LYP-GD3BJ/6-311G(d,p) level. *Δε* values are given in mol^{-1} cm^{-1} dm^3.

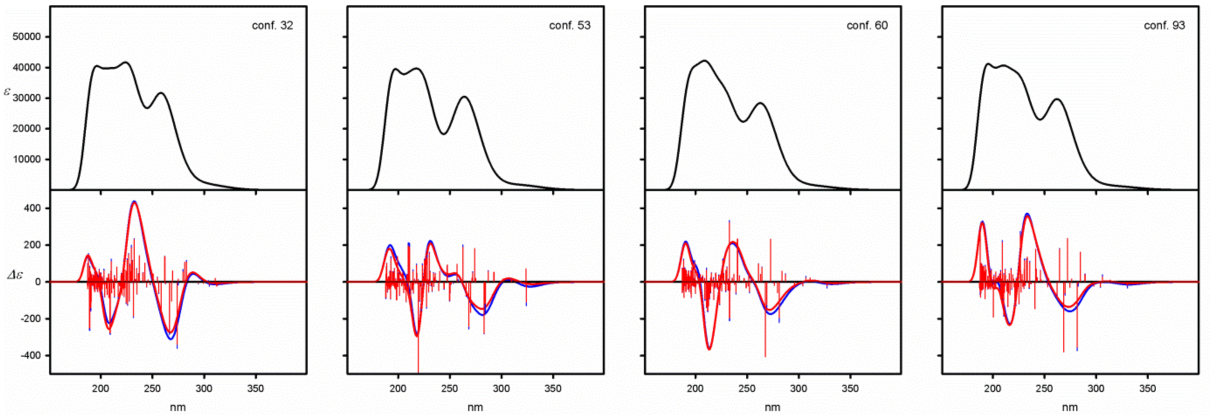

Figure S30. UV (upper panels) and ECD (lower panels) spectra calculated at the TD-M06-2X/6-311G(d,p) level for individual low-energy conformers of **6a**. Wavelengths were not corrected. Geometries were optimized at the B3LYP-GD3BJ/6-311G(d,p) level. *Δε* values are given in mol^{-1} cm^{-1} dm^3.

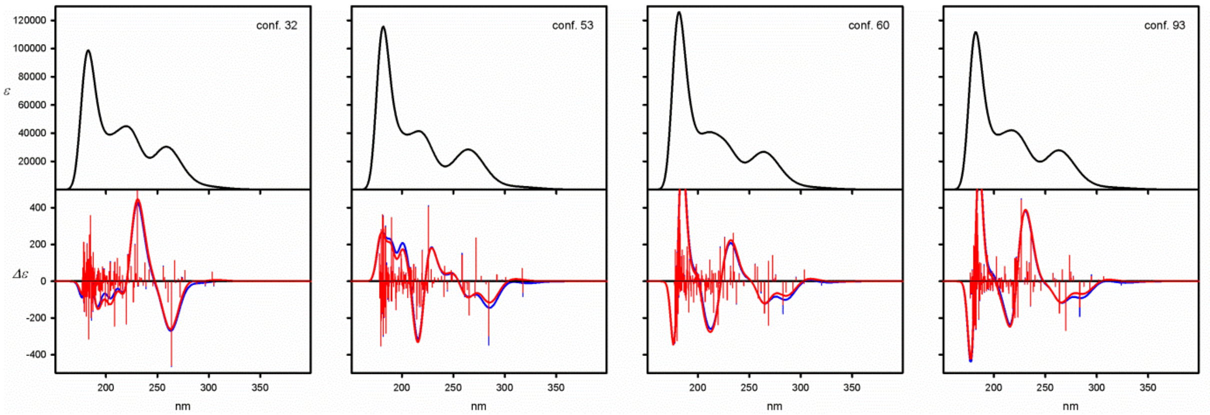

Figure S31. UV (upper panels) and ECD (lower panels) spectra calculated at the TD-wB97XD/6-311G(d,p) level for individual low-energy conformers of **6a**. Wavelengths were not corrected. Geometries were optimized at the B3LYP-GD3BJ/6-311G(d,p) level. *Δε* values are given in mol^{-1} cm^{-1} dm^3.

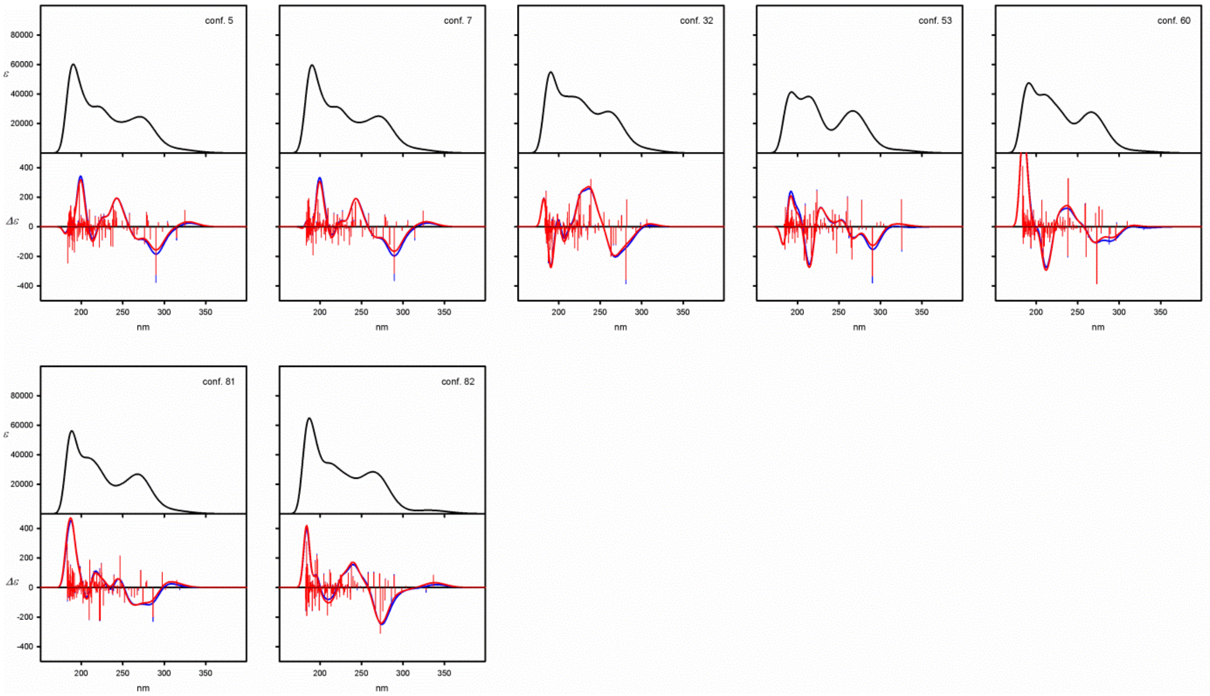

Figure S32. UV (upper panels) and ECD (lower panels) spectra calculated at the TD-CAM-B3LYP/6-311G(d,p) level for individual low-energy conformers of **6a**. Wavelengths were not corrected. Geometries were optimized at the M06L/6-311G(d,p) level. *Δε* values are given in mol^{-1} cm^{-1} dm^3.

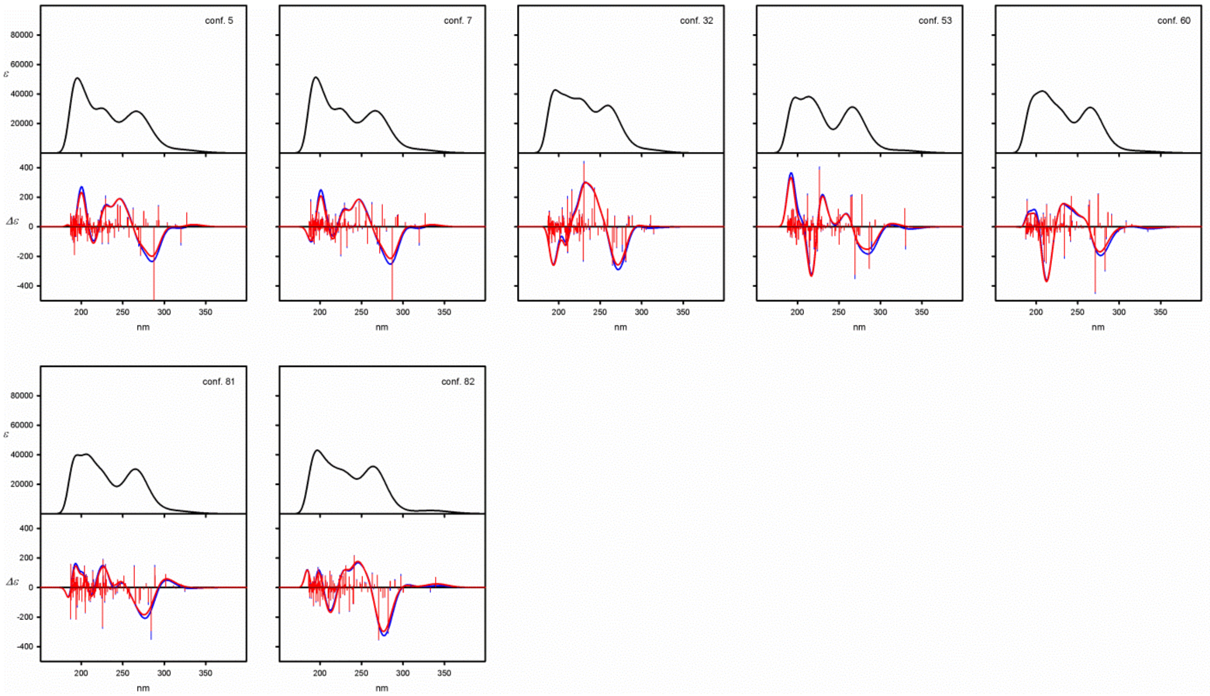

Figure S33. UV (upper panels) and ECD (lower panels) spectra calculated at the TD-M06-2X/6-311G(d,p) level for individual low-energy conformers of **6a**. Wavelengths were not corrected. Geometries were optimized at the M06L/6-311G(d,p) level. *Δε* values are given in mol^{-1} cm^{-1} dm^3.

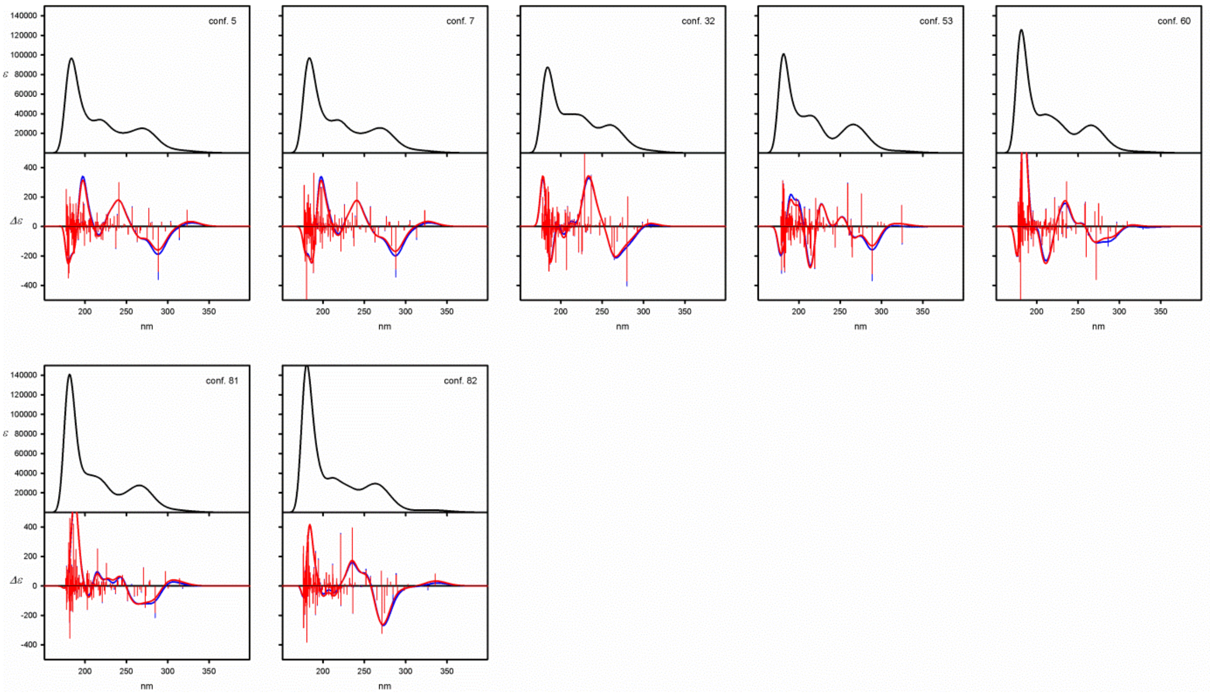

Figure S34. UV (upper panels) and ECD (lower panels) spectra calculated at the TD-wB97XD/6-311G(d,p) level for individual low-energy conformers of **6a**. Wavelengths were not corrected. Geometries were optimized at the M06L/6-311G(d,p) level. $\Delta\varepsilon$ values are given in mol^{-1} cm^{-1} dm^{3}.

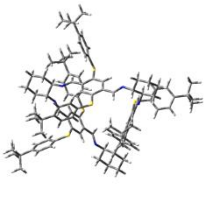

conf. 15

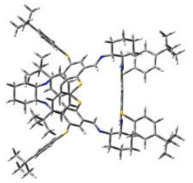

conf. 18

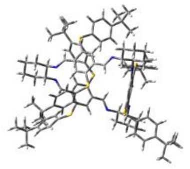

conf. 20

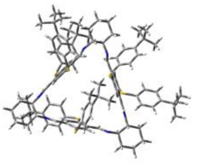

conf. 25

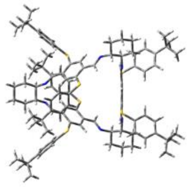

conf. 26

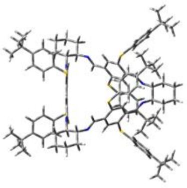

conf. 27

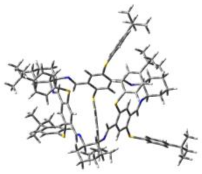

conf. 29

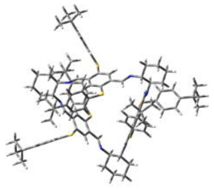

conf. 30

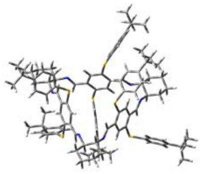

conf. 31

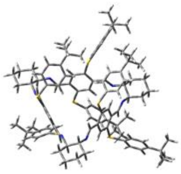

conf. 38

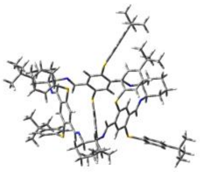

conf. 40

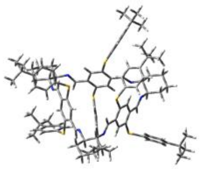

conf. 42

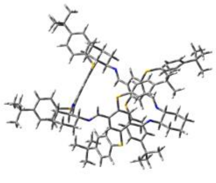

conf. 46

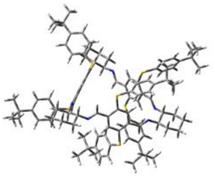

conf. 47

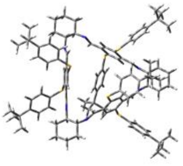

conf. 48

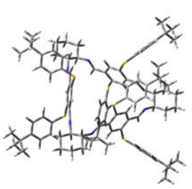

conf. 49

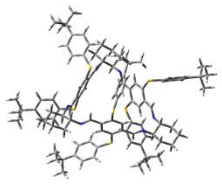

conf. 53

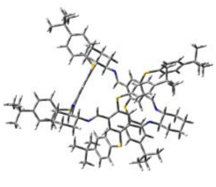

conf. 61

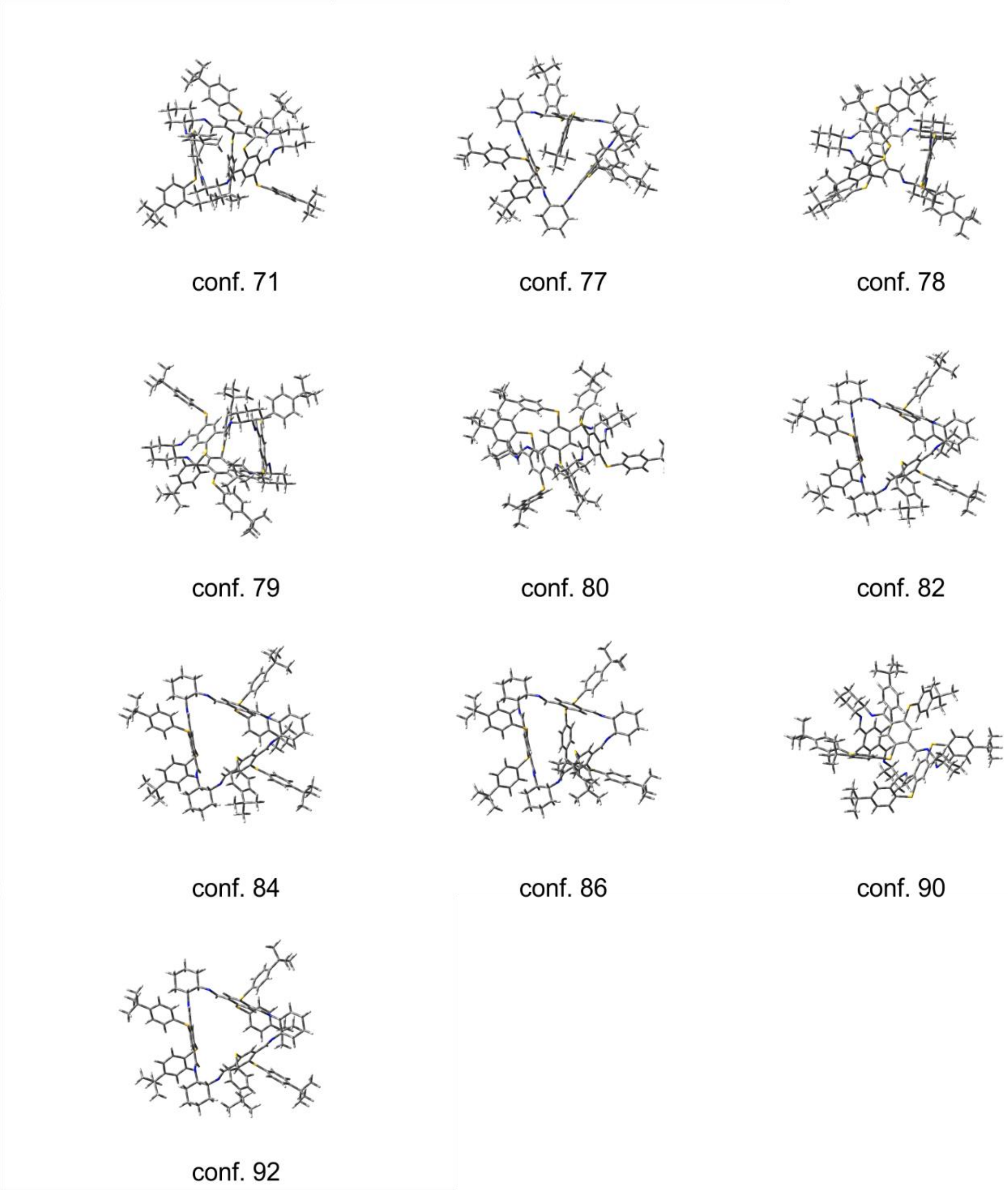

Figure S35. Structures of the low-energy conformers of **6c**, calculated at the B3LYP/6-31G(d,p) level.

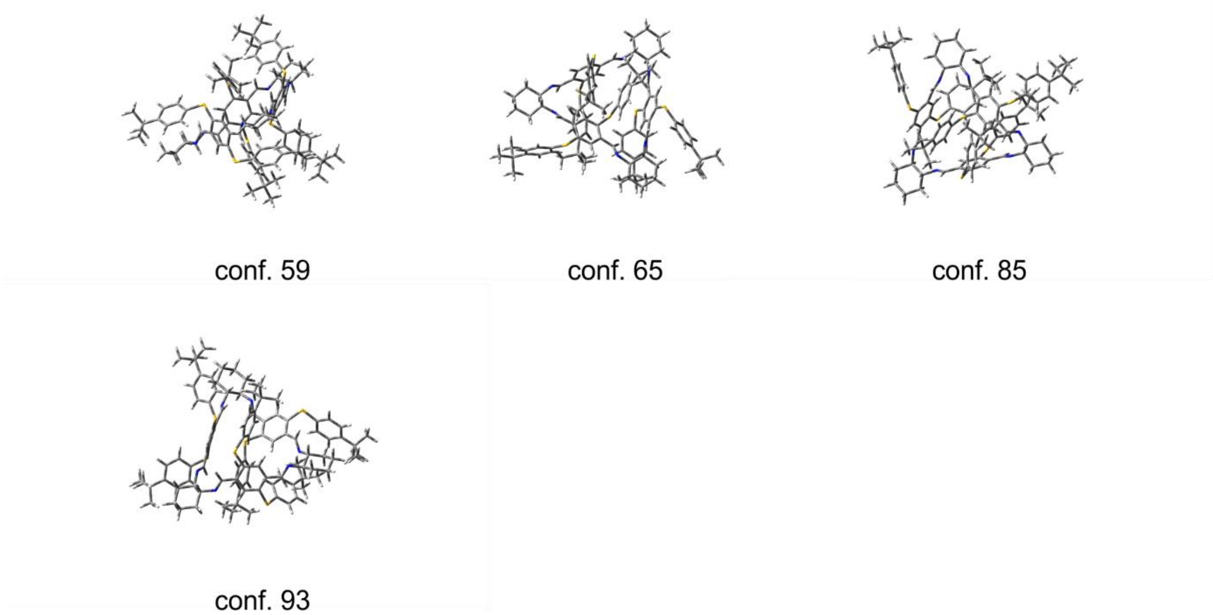

Figure S36. Structures of the low-energy conformers of **6c**, calculated at the B3LYP-GD3BJ/6-31G(d,p) level.

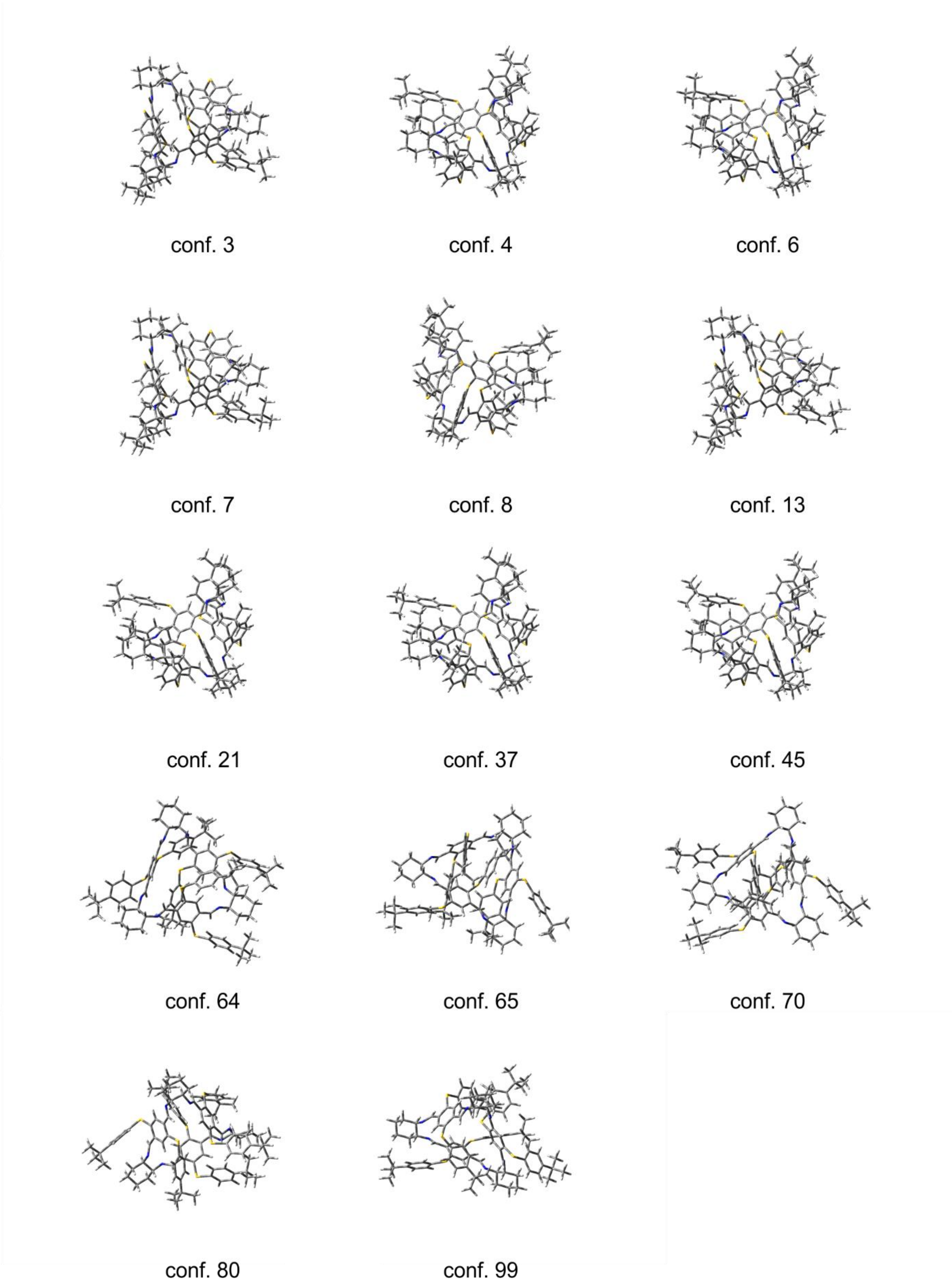

Figure S37. Structures of the low-energy conformers of **6c**, calculated at the M06L/6-31G(d,p) level.

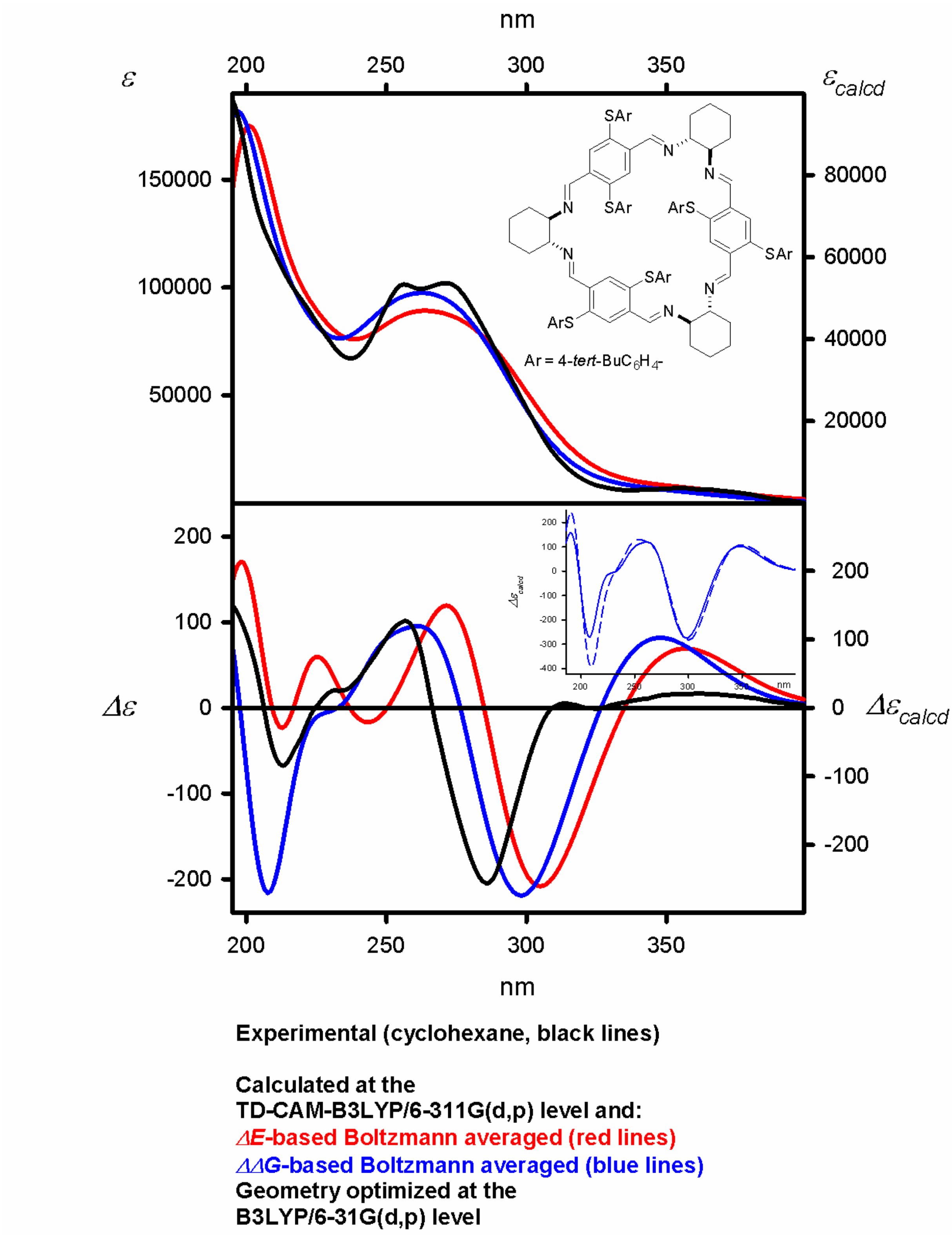

Figure S38. UV (upper panel) and ECD (lower panel) spectra of **6c** measured in cyclohexane (solid black lines) and calculated at the TD-CAM-B3LYP/6-311G(d,p) level for geometries optimized at the B3LYP/6-31G(d,p) level. The calculated ECD spectra were Boltzmann-averaged based on *ΔE* (red lines) and *ΔΔG* values (blue lines). Wavelengths were corrected to match the experimental UV maxima. The insert shows the comparison between the ECD spectra calculated for the lowest energy conformer of a given compound (dashed blue lines) and the *ΔΔ*G-based and Boltzmann averaged (solid blue lines). *Δε* values are given in mol^{-1} cm^{-1} dm^{3}.

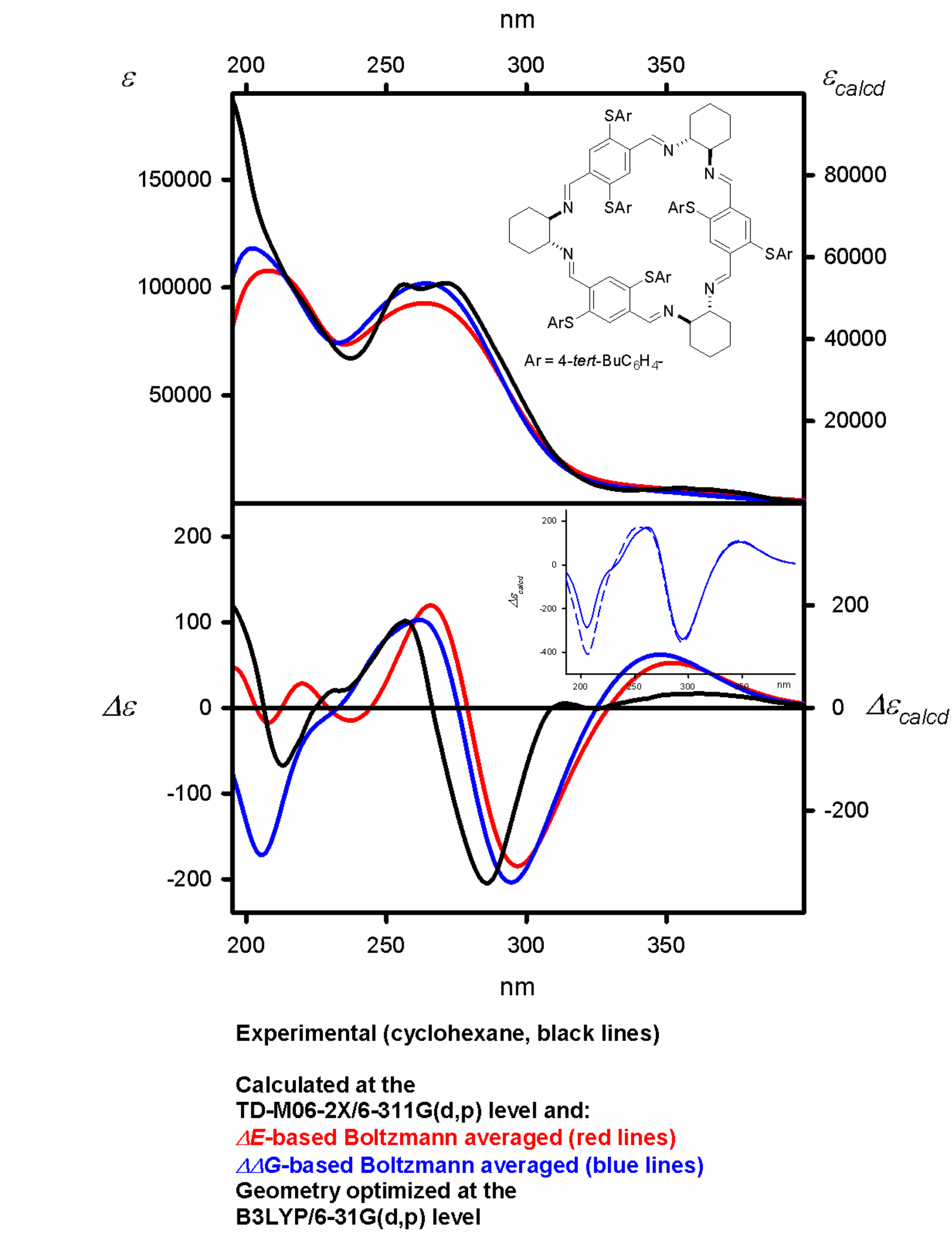

Figure S39. UV (upper panel) and ECD (lower panel) spectra of **6c** measured in cyclohexane (solid black lines) and calculated at the TD-M06-2X/6-311G(d,p) level for geometries optimized at the B3LYP/6-31G(d,p) level. The calculated ECD spectra were Boltzmann-averaged based on *ΔE* (red lines) and *ΔΔG* values (blue lines). Wavelengths were corrected to match the experimental UV maxima. The insert shows the comparison between the ECD spectra calculated for the lowest energy conformer of a given compound (dashed blue lines) and the ΔΔG-based and Boltzmann averaged (solid blue lines). *Δε* values are given in mol^{-1} cm^{-1} dm^{3}.

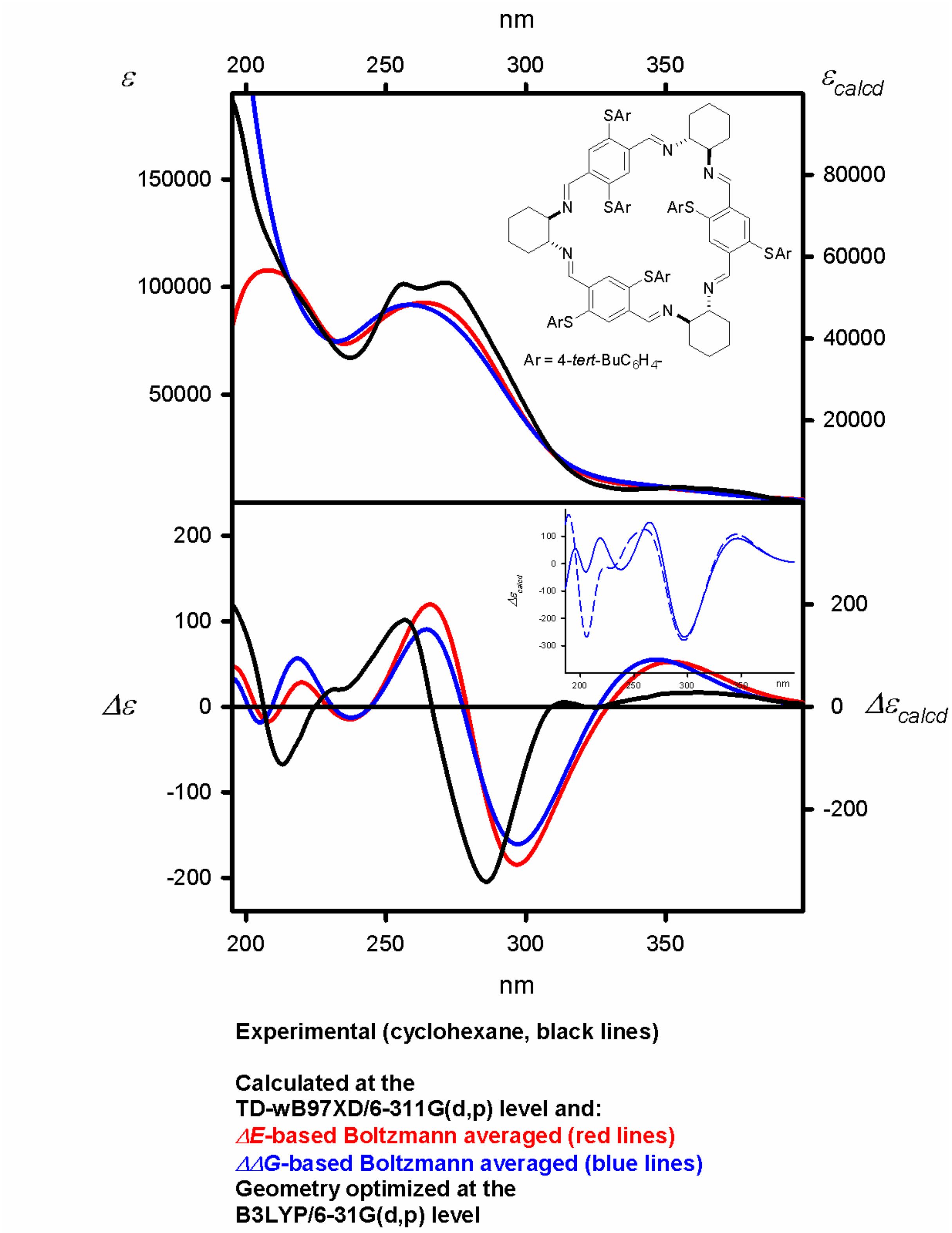

Figure S40. UV (upper panel) and ECD (lower panel) spectra of **6c** measured in cyclohexane (solid black lines) and calculated at the TD-wB97XD/6-311G(d,p) level for geometries optimized at the B3LYP/6-31G(d,p) level. The calculated ECD spectra were Boltzmann-averaged based on *ΔE* (red lines) and *ΔΔG* values (blue lines). Wavelengths were corrected to match the experimental UV maxima. The insert shows the comparison between the ECD spectra calculated for the lowest energy conformer of a given compound (dashed blue lines) and the *ΔΔ*G-based and Boltzmann averaged (solid blue lines *Δε* values are given in mol^{-1} cm^{-1} dm^{3}.

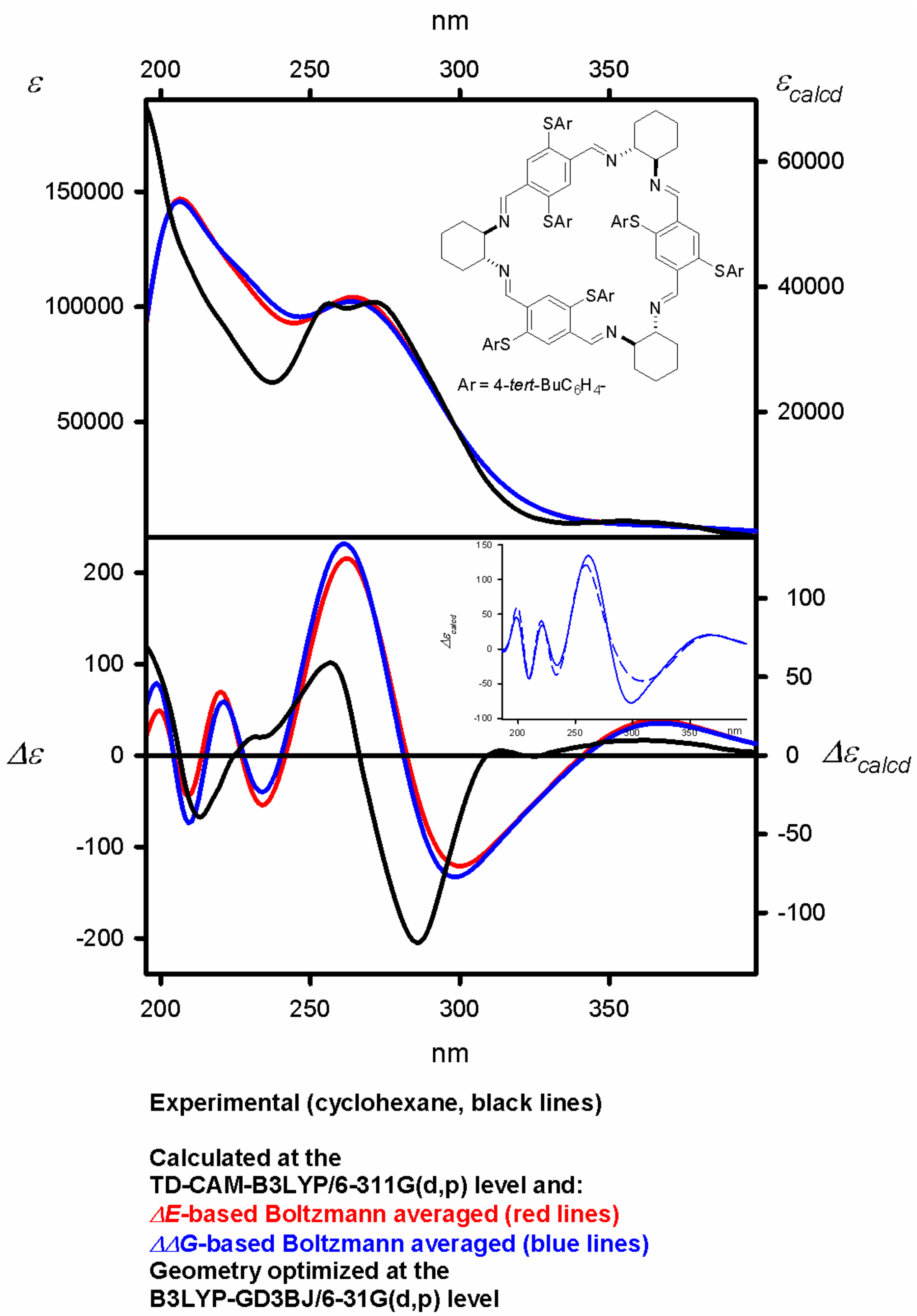

Figure S41. UV (upper panel) and ECD (lower panel) spectra of **6c** measured in cyclohexane (solid black lines) and calculated at the TD-CAM-B3LYP/6-311G(d,p) level for geometries optimized at the B3LYP-GD3BJ/6-31G(d,p) level. The calculated ECD spectra were Boltzmann-averaged based on *ΔE* (red lines) and *ΔΔG* values (blue lines). Wavelengths were corrected to match the experimental UV maxima. The insert shows the comparison between the ECD spectra calculated for the lowest energy conformer of a given compound (dashed blue lines) and the *ΔΔ*G-based and Boltzmann averaged (solid blue lines). *Δε* values are given in mol^{-1} cm^{-1} dm^{3}.

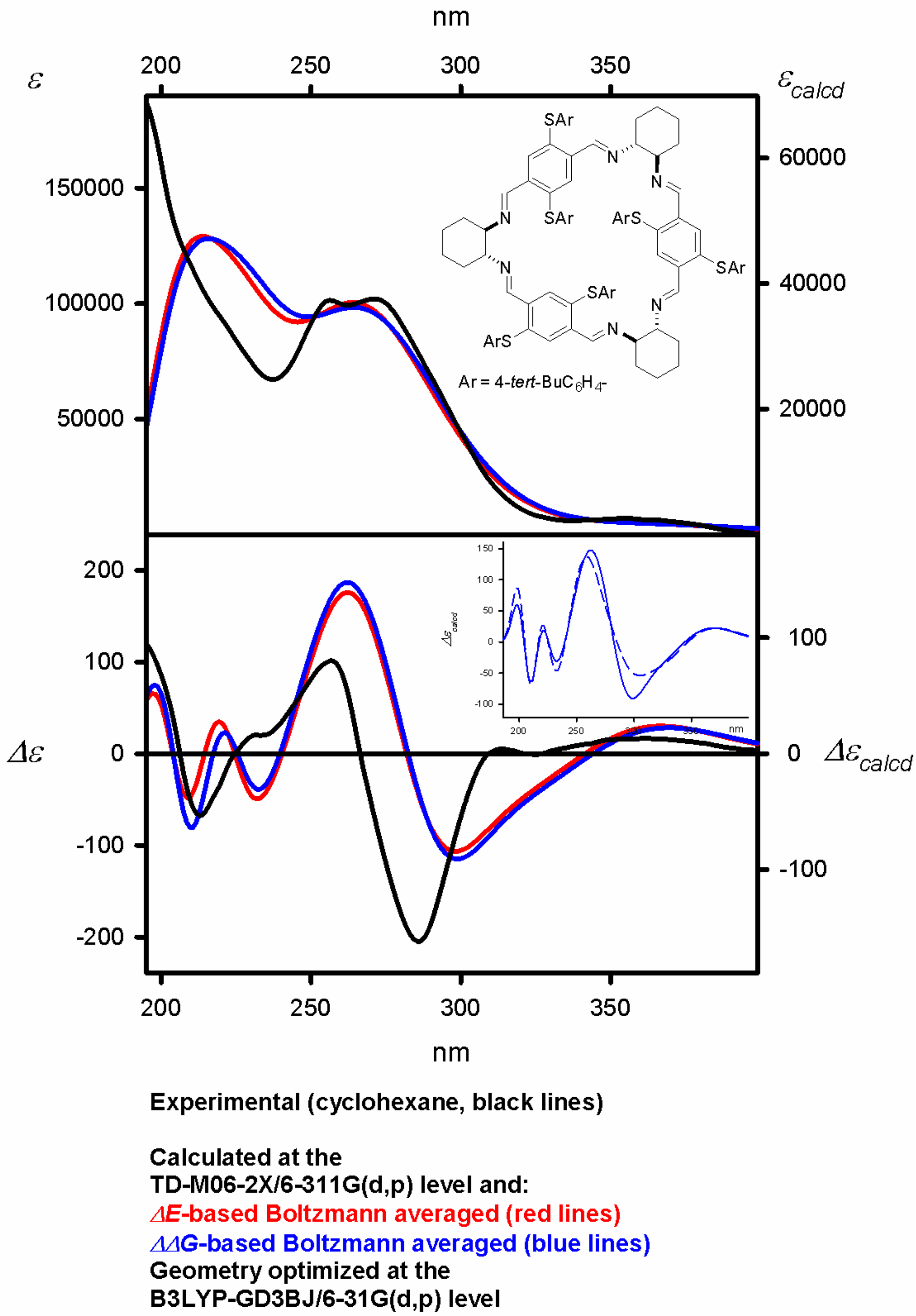

Figure S42. UV (upper panel) and ECD (lower panel) spectra of **6c** measured in cyclohexane (solid black lines) and calculated at the TD-M06-2X/6-311G(d,p) level for geometries optimized at the B3LYP-GD3BJ/6-31G(d,p) level. The calculated ECD spectra were Boltzmann-averaged based on *ΔE* (red lines) and *ΔΔG* values (blue lines). Wavelengths were corrected to match the experimental UV maxima. The insert shows the comparison between the ECD spectra calculated for the lowest energy conformer of a given compound (dashed blue lines) and the *ΔΔ*G-based and Boltzmann averaged (solid blue lines). *Δε* values are given in mol^{-1} cm^{-1} dm^3.

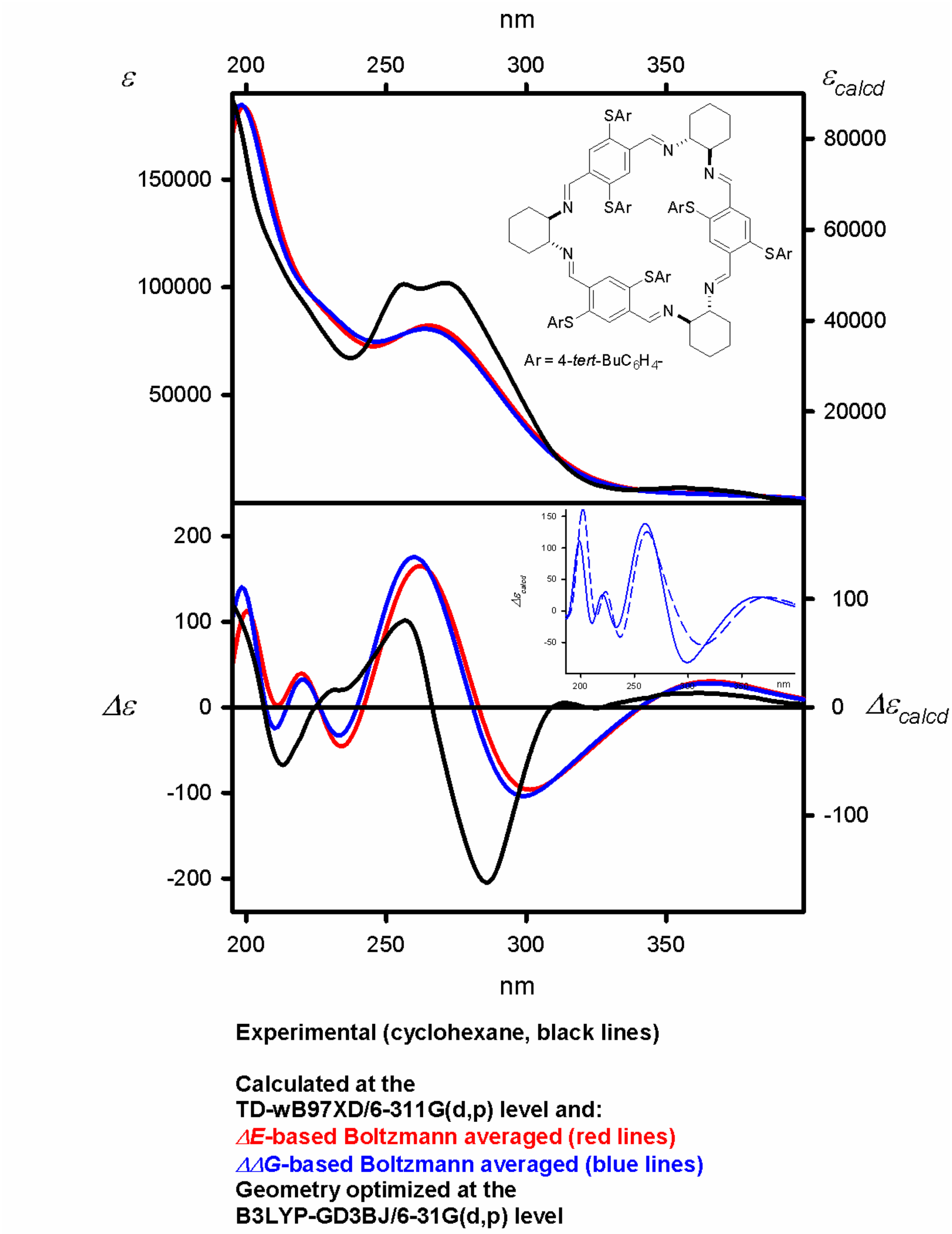

Figure S43. UV (upper panel) and ECD (lower panel) spectra of **6c** measured in cyclohexane (solid black lines) and calculated at the TD-wB97XD/6-311G(d,p) level for geometries optimized at the B3LYP-GD3BJ/6-31G(d,p) level. The calculated ECD spectra were Boltzmann-averaged based on *ΔE* (red lines) and *ΔΔG* values (blue lines). Wavelengths were corrected to match the experimental UV maxima. The insert shows the comparison between the ECD spectra calculated for the lowest energy conformer of a given compound (dashed blue lines) and the *ΔΔ*G-based and Boltzmann averaged (solid blue lines). *Δε* values are given in mol^{-1} cm^{-1} dm^{3}.

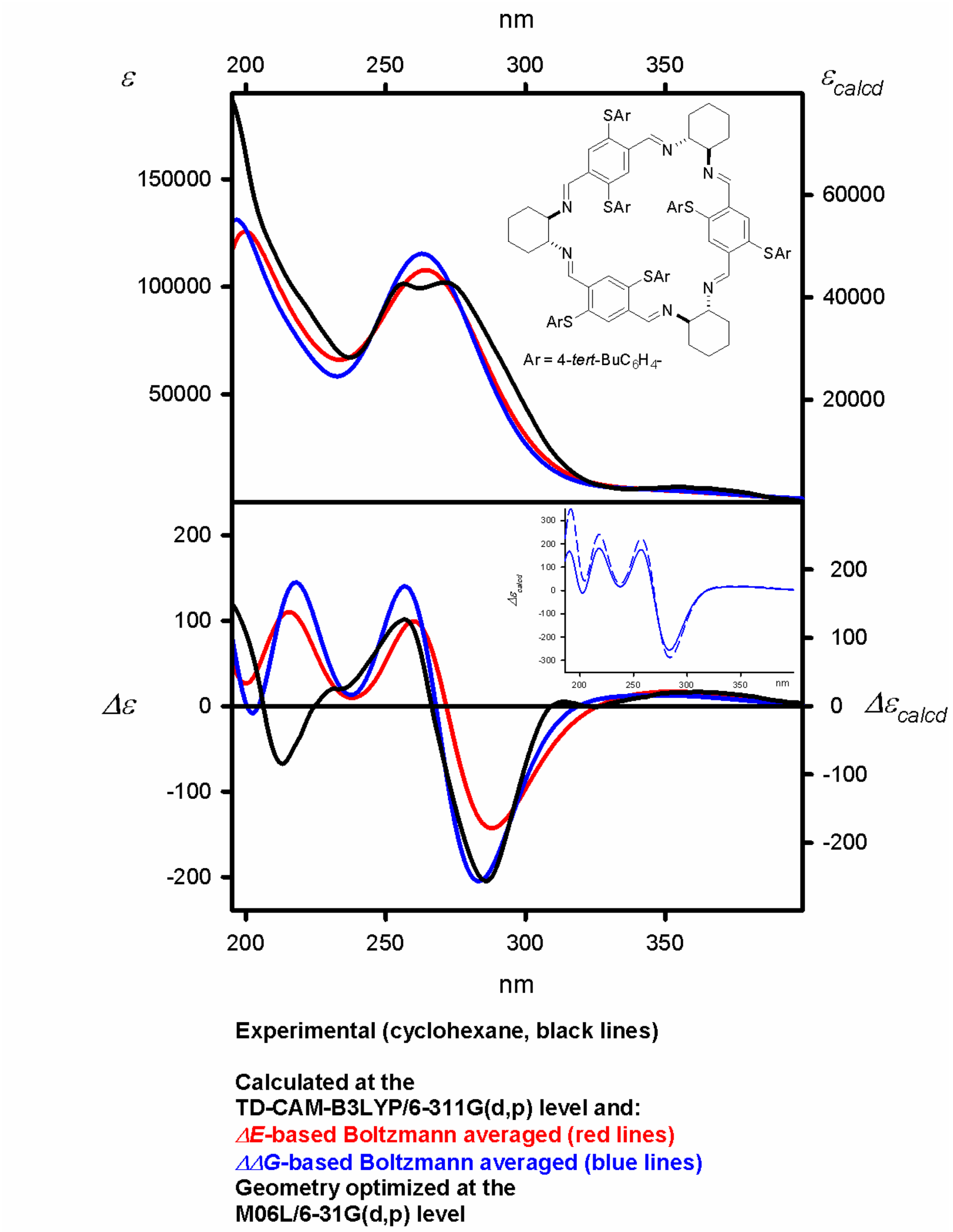

Figure S44. UV (upper panel) and ECD (lower panel) spectra of **6c** measured in cyclohexane (solid black lines) and calculated at the TD-CAM-B3LYP/6-311G(d,p) level for geometries optimized at the M06L/6-31G(d,p) level. The calculated ECD spectra were Boltzmann-averaged based on ΔE (red lines) and $\Delta\Delta G$ values (blue lines). Wavelengths were corrected to match the experimental UV maxima. The insert shows the comparison between the ECD spectra calculated for the lowest energy conformer of a given compound (dashed blue lines) and the $\Delta\Delta$G-based and Boltzmann averaged (solid blue lines). $\Delta\varepsilon$ values are given in mol^{-1} cm^{-1} dm^{3}.

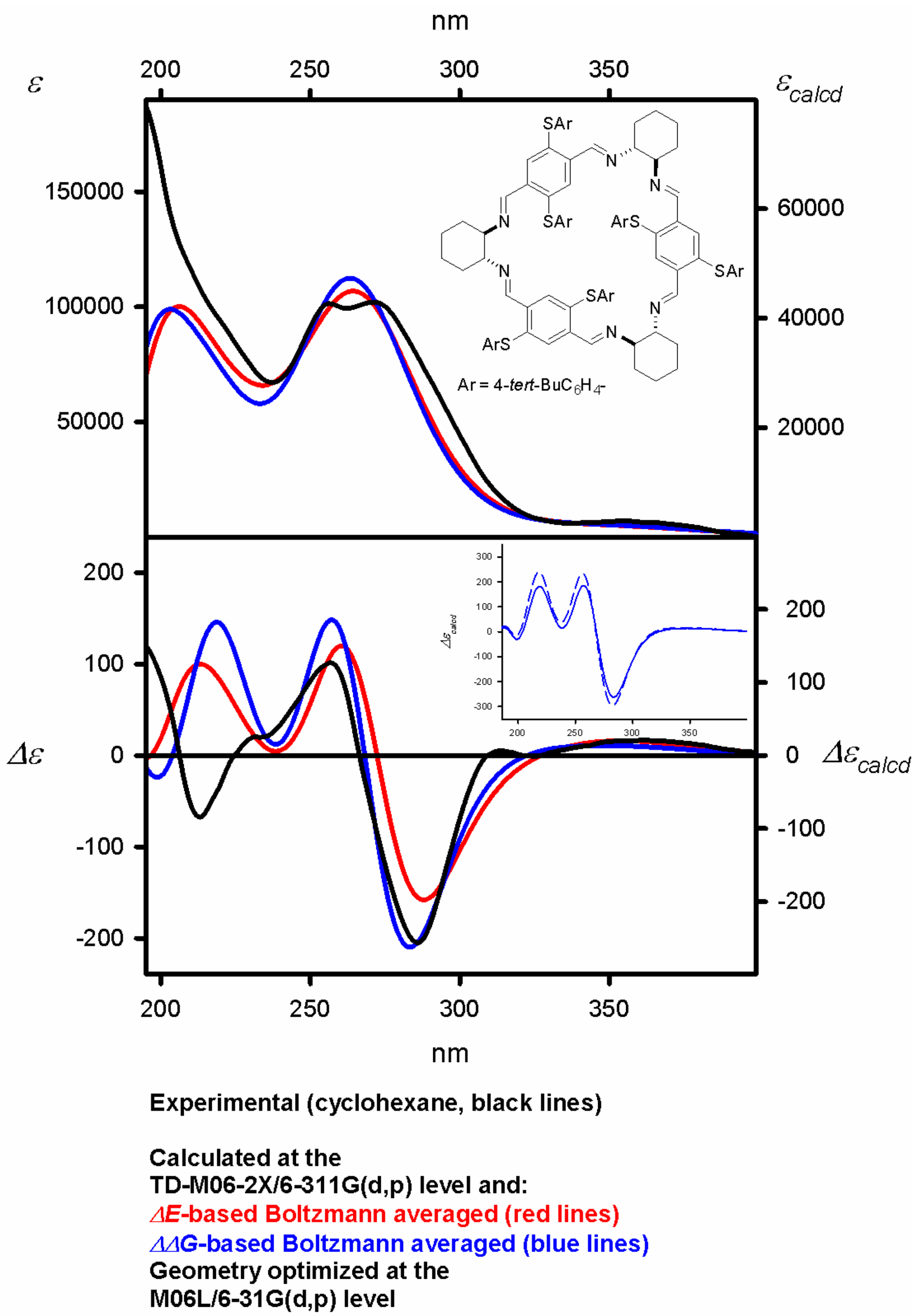

Figure S45. UV (upper panel) and ECD (lower panel) spectra of **6c** measured in cyclohexane (solid black lines) and calculated at the TD-M06-2X/6-311G(d,p) level for geometries optimized at the M06L/6-31G(d,p) level. The calculated ECD spectra were Boltzmann-averaged based on *ΔE* (red lines) and *ΔΔG* values (blue lines). Wavelengths were corrected to match the experimental UV maxima. The insert shows the comparison between the ECD spectra calculated for the lowest energy conformer of a given compound (dashed blue lines) and the *ΔΔ*G-based and Boltzmann averaged (solid blue lines). *Δε* values are given in mol^{-1} cm^{-1} dm^{3}.

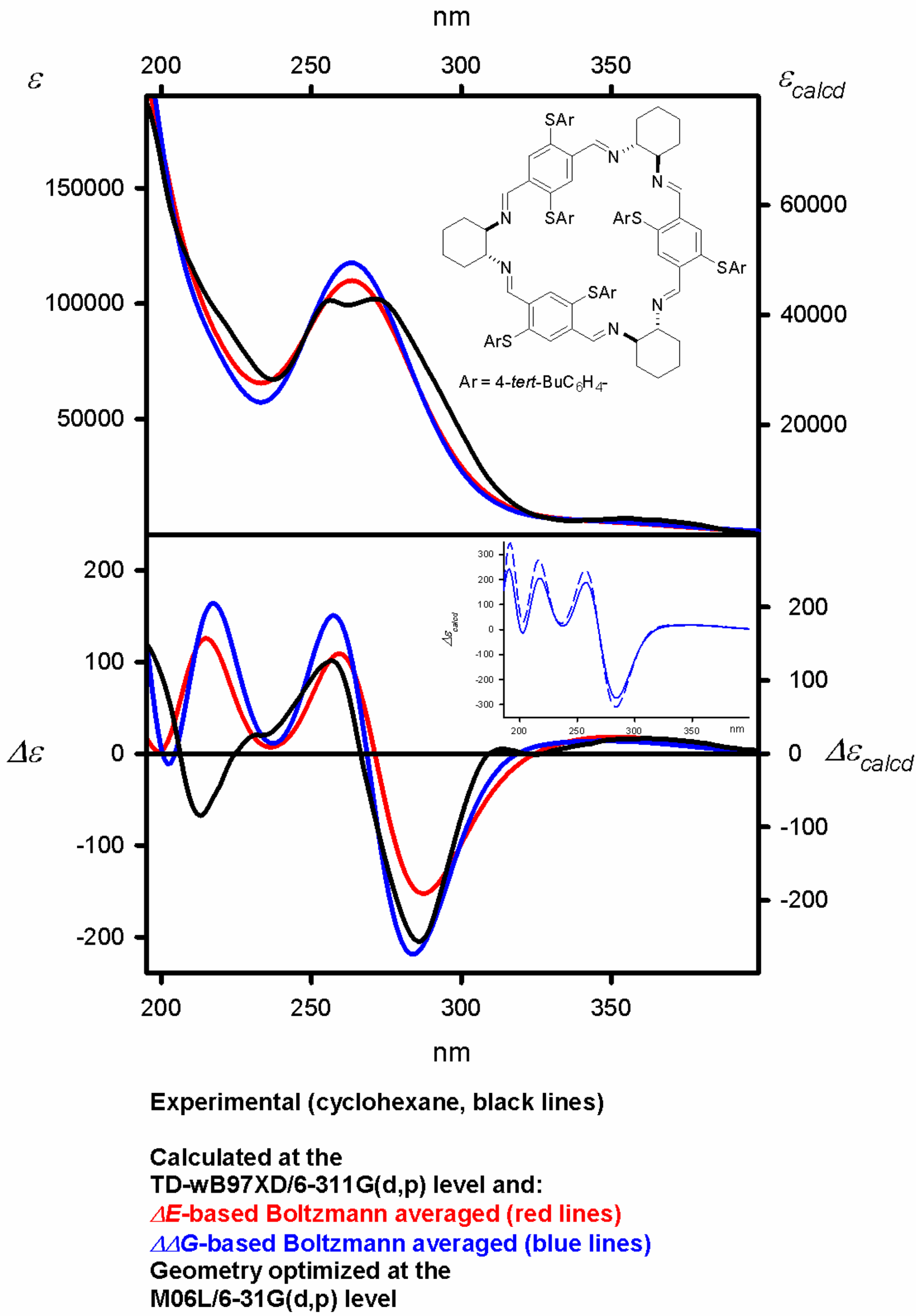

Figure S46. UV (upper panel) and ECD (lower panel) spectra of **6c** measured in cyclohexane (solid black lines) and calculated at the TD-wB97XD/6-311G(d,p) level for geometries optimized at the M06L/6-31G(d,p) level. The calculated ECD spectra were Boltzmann-averaged based on ΔE (red lines) and $\Delta\Delta G$ values (blue lines). Wavelengths were corrected to match the experimental UV maxima. The insert shows the comparison between the ECD spectra calculated for the lowest energy conformer of a given compound (dashed blue lines) and the $\Delta\Delta G$-based and Boltzmann averaged (solid blue lines). $\Delta\varepsilon$ values are given in mol^{-1} cm^{-1} dm^{3}.

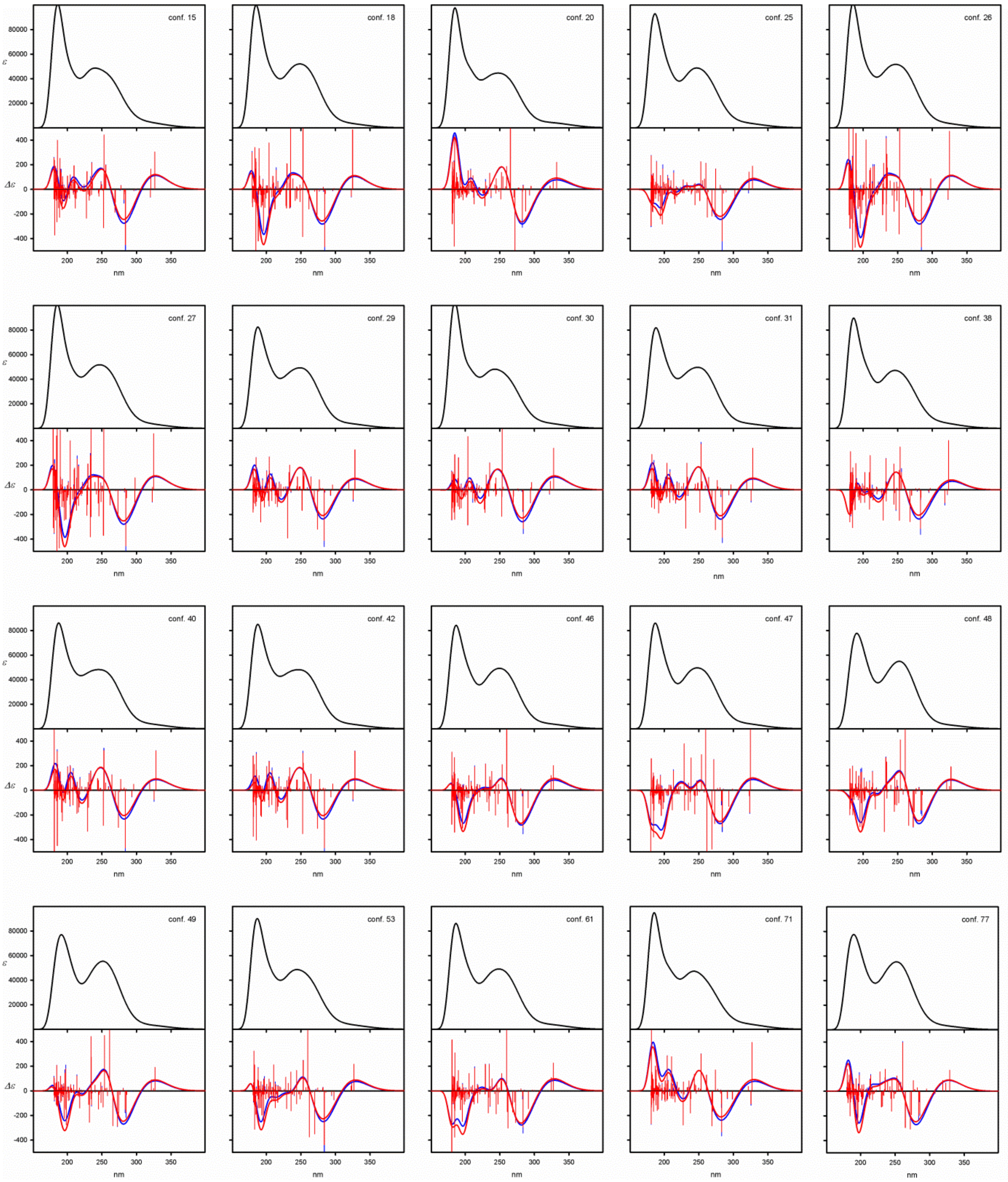
conf. 15
conf. 18
conf. 20
conf. 25
conf. 26
conf. 27
conf. 29
conf. 30
conf. 31
conf. 38
conf. 40
conf. 42
conf. 46
conf. 47
conf. 48
conf. 49
conf. 53
conf. 61
conf. 71
conf. 77
ε
Δε
nm
80000
60000
40000
20000
400
200
0
-200
-400
200
250
300
350

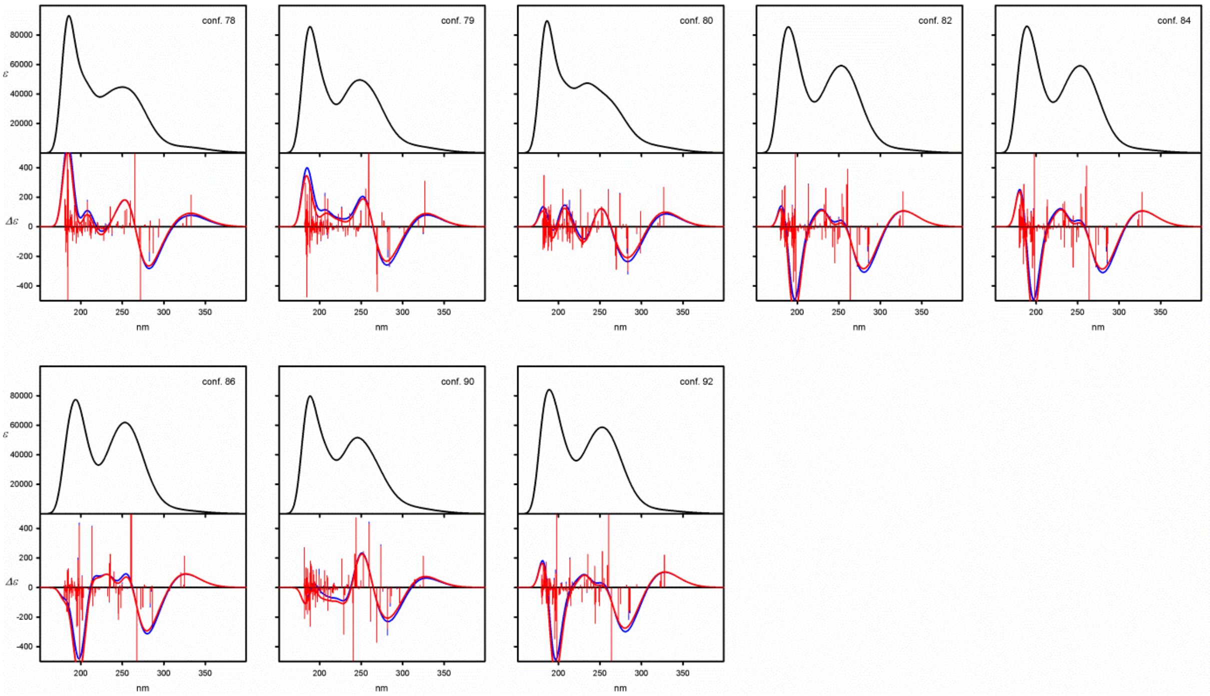

Figure S47. UV (upper panels) and ECD (lower panels) spectra calculated at the TD-CAM-B3LYP/6-311G(d,p) level for individual low-energy conformers of **6c**. Wavelengths were not corrected. Geometries were optimized at the B3LYP/6-31G(d,p) level. *Δε* values are given in mol^{-1} cm^{-1} dm^3.

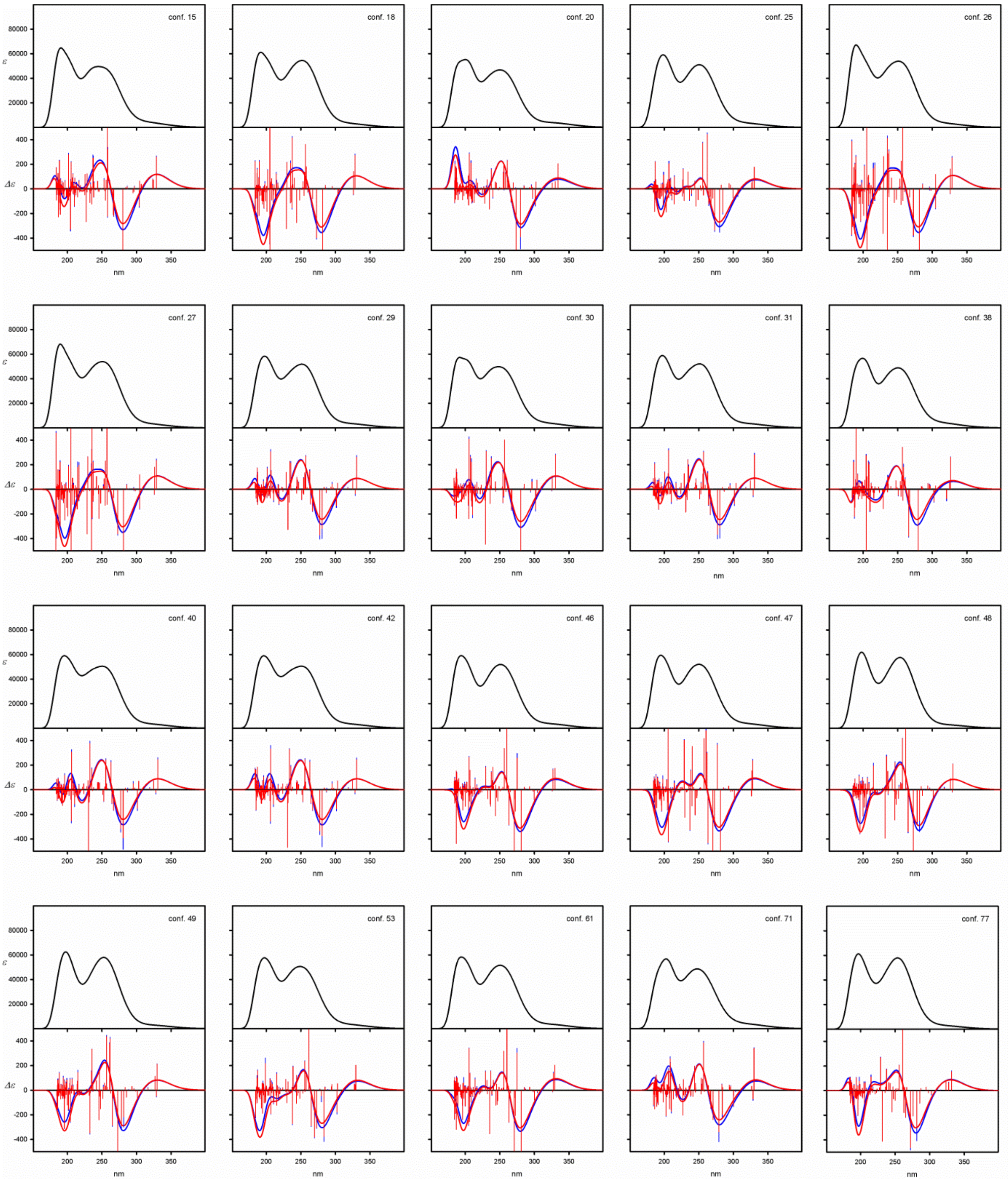
conf. 15
conf. 18
conf. 20
conf. 25
conf. 26
conf. 27
conf. 29
conf. 30
conf. 31
conf. 38
conf. 40
conf. 42
conf. 46
conf. 47
conf. 48
conf. 49
conf. 53
conf. 61
conf. 71
conf. 77
80000
60000
40000
20000
ε
400
200
0
-200
-400
Δε
200
250
300
350
nm

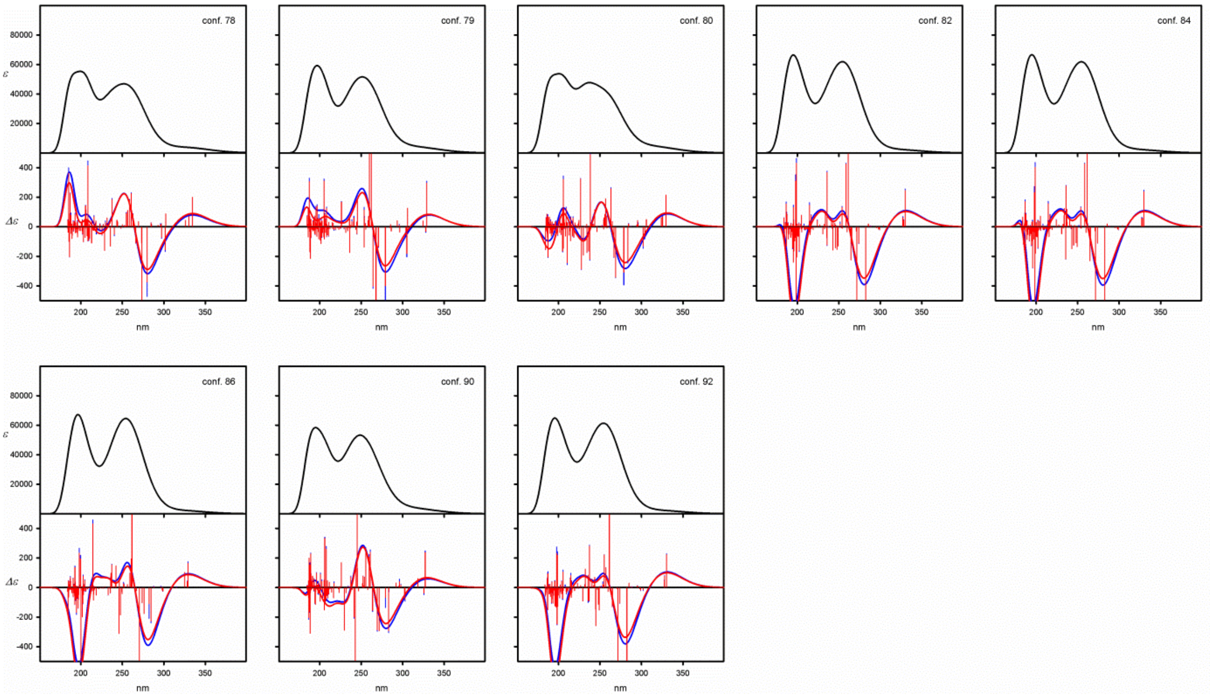

Figure S48. UV (upper panels) and ECD (lower panels) spectra calculated at the TD-M06-2X/6-311G(d,p) level for individual low-energy conformers of **6c**. Wavelengths were not corrected. Geometries were optimized at the B3LYP/6-31G(d,p) level. *Δε* values are given in mol^{-1} cm^{-1} dm^{3}.

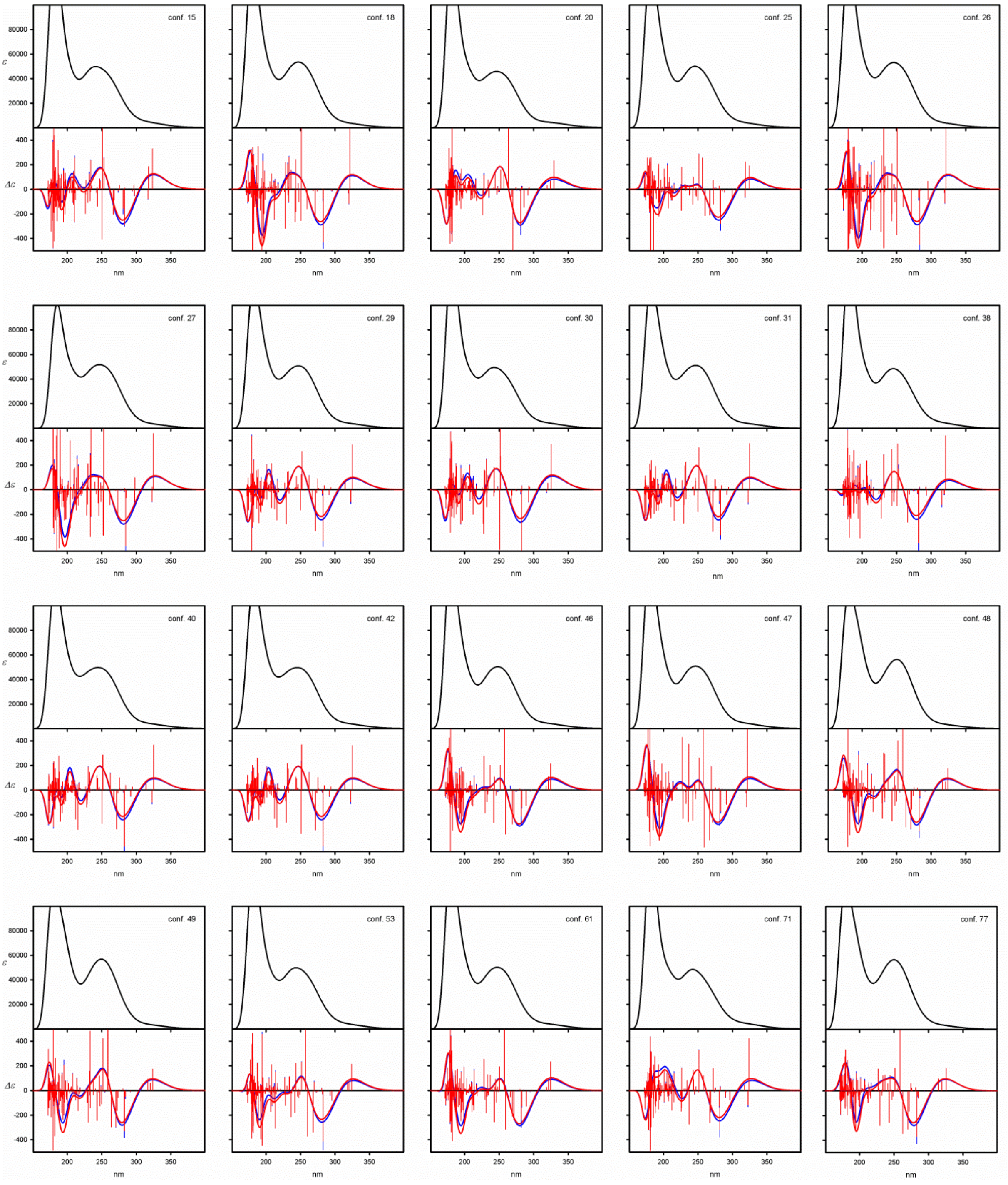
conf. 15
conf. 18
conf. 20
conf. 25
conf. 26
conf. 27
conf. 29
conf. 30
conf. 31
conf. 38
conf. 40
conf. 42
conf. 46
conf. 47
conf. 48
conf. 49
conf. 53
conf. 61
conf. 71
conf. 77
ε
Δε
nm

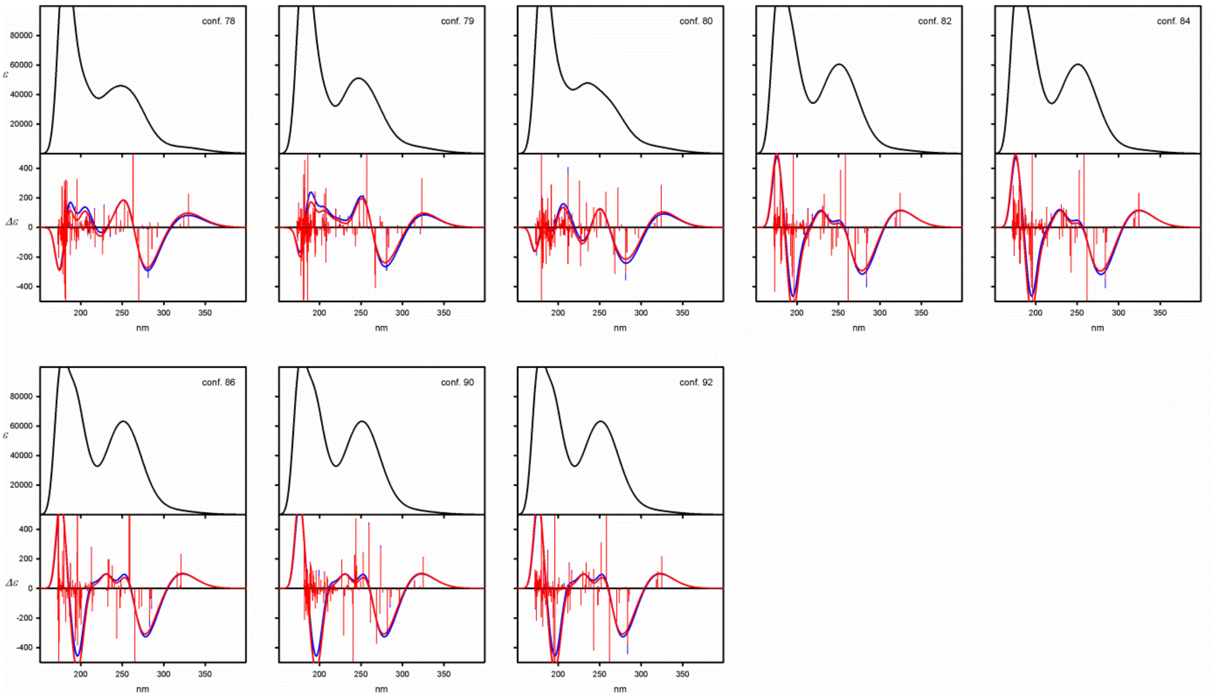

Figure S49. UV (upper panels) and ECD (lower panels) spectra calculated at the TD-wB97XD/6-311G(d,p) level for individual low-energy conformers of **6c**. Wavelengths were not corrected. Geometries were optimized at the B3LYP/6-31G(d,p) level. $\Delta\varepsilon$ values are given in mol^{-1} cm^{-1} dm^3.

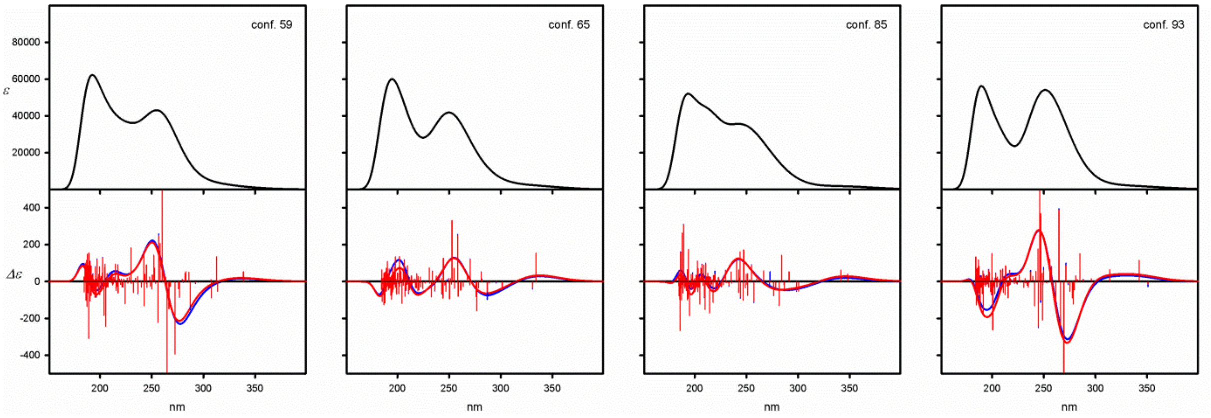

Figure S50. UV (upper panels) and ECD (lower panels) spectra calculated at the TD-CAM-B3LYP/6-311G(d,p) level for individual low-energy conformers of **6c**. Wavelengths were not corrected. Geometries were optimized at the B3LYP-GD3BJ/6-31G(d,p) level. $\Delta\varepsilon$ values are given in mol^{-1} cm^{-1} dm^3.

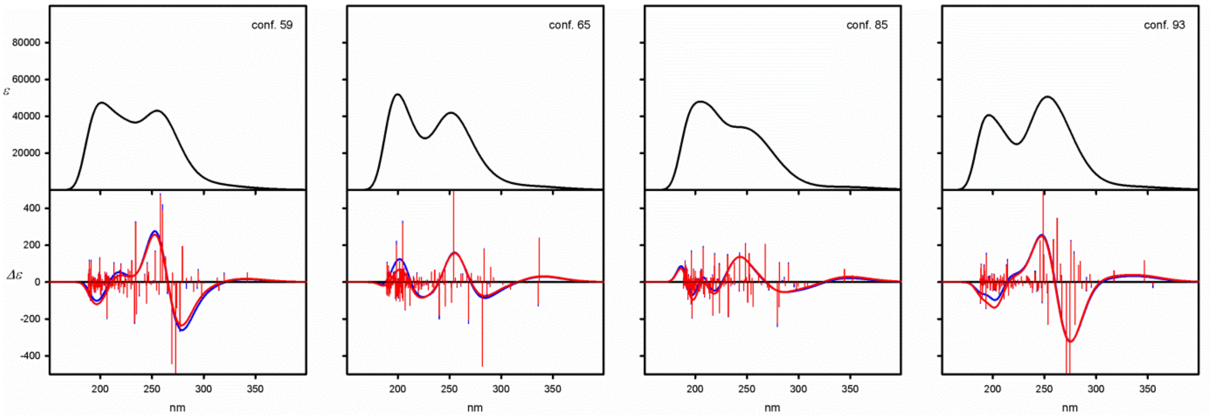

Figure S51. UV (upper panels) and ECD (lower panels) spectra calculated at the TD-M06-2X/6-311G(d,p) level for individual low-energy conformers of **6c**. Wavelengths were not corrected. Geometries were optimized at the B3LYP-GD3BJ/6-31G(d,p) level. *Δε* values are given in mol^{-1} cm^{-1} dm^3.

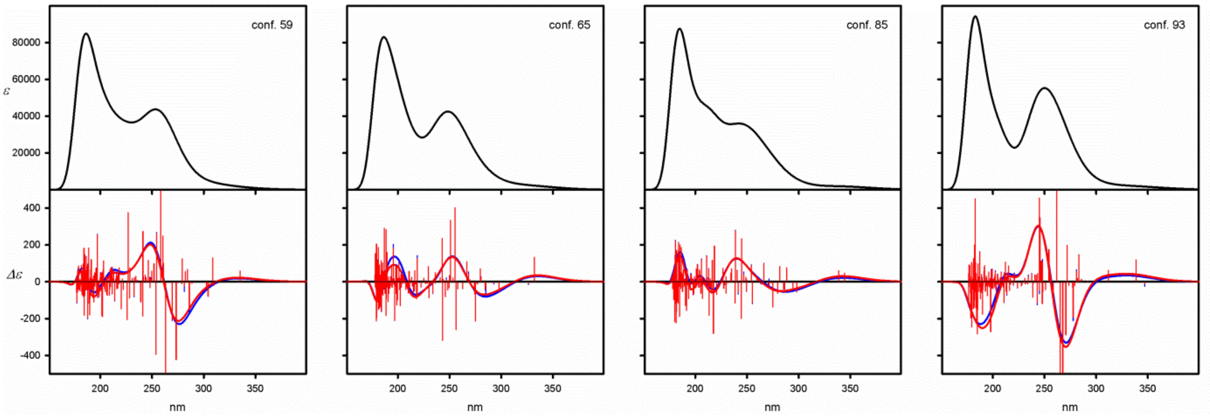

Figure S52. UV (upper panels) and ECD (lower panels) spectra calculated at the TD-wB97XD/6-311G(d,p) level for individual low-energy conformers of **6c**. Wavelengths were not corrected. Geometries were optimized at the B3LYP-GD3BJ/6-31G(d,p) level. *Δε* values are given in mol^{-1} cm^{-1} dm^3.

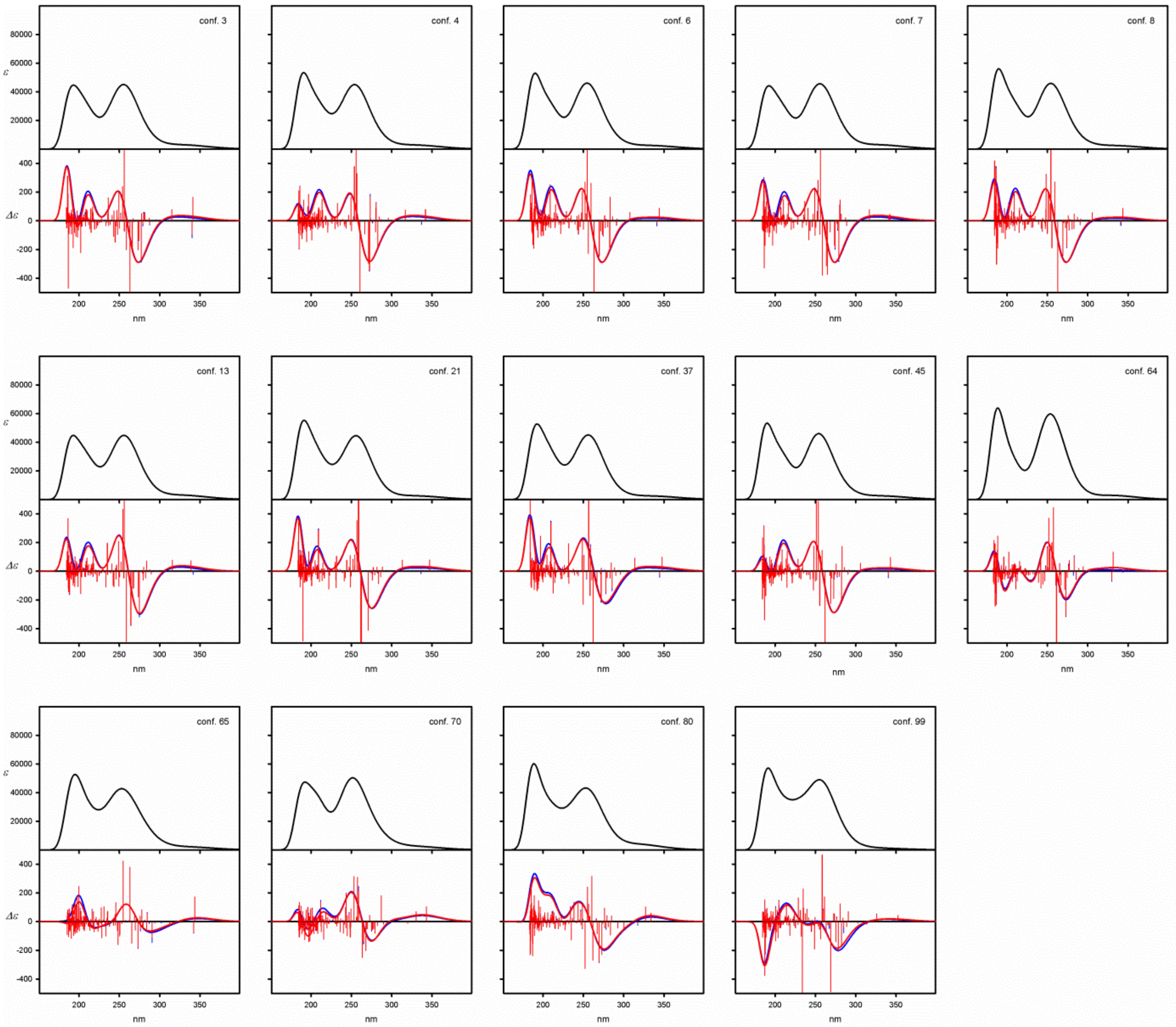

Figure S53. UV (upper panels) and ECD (lower panels) spectra calculated at the TD-CAM-B3LYP/6-311G(d,p) level for individual low-energy conformers of **6c**. Wavelengths were not corrected. Geometries were optimized at the M06L/6-31G(d,p) level. *Δε* values are given in mol^{-1} cm^{-1} dm^{3}.

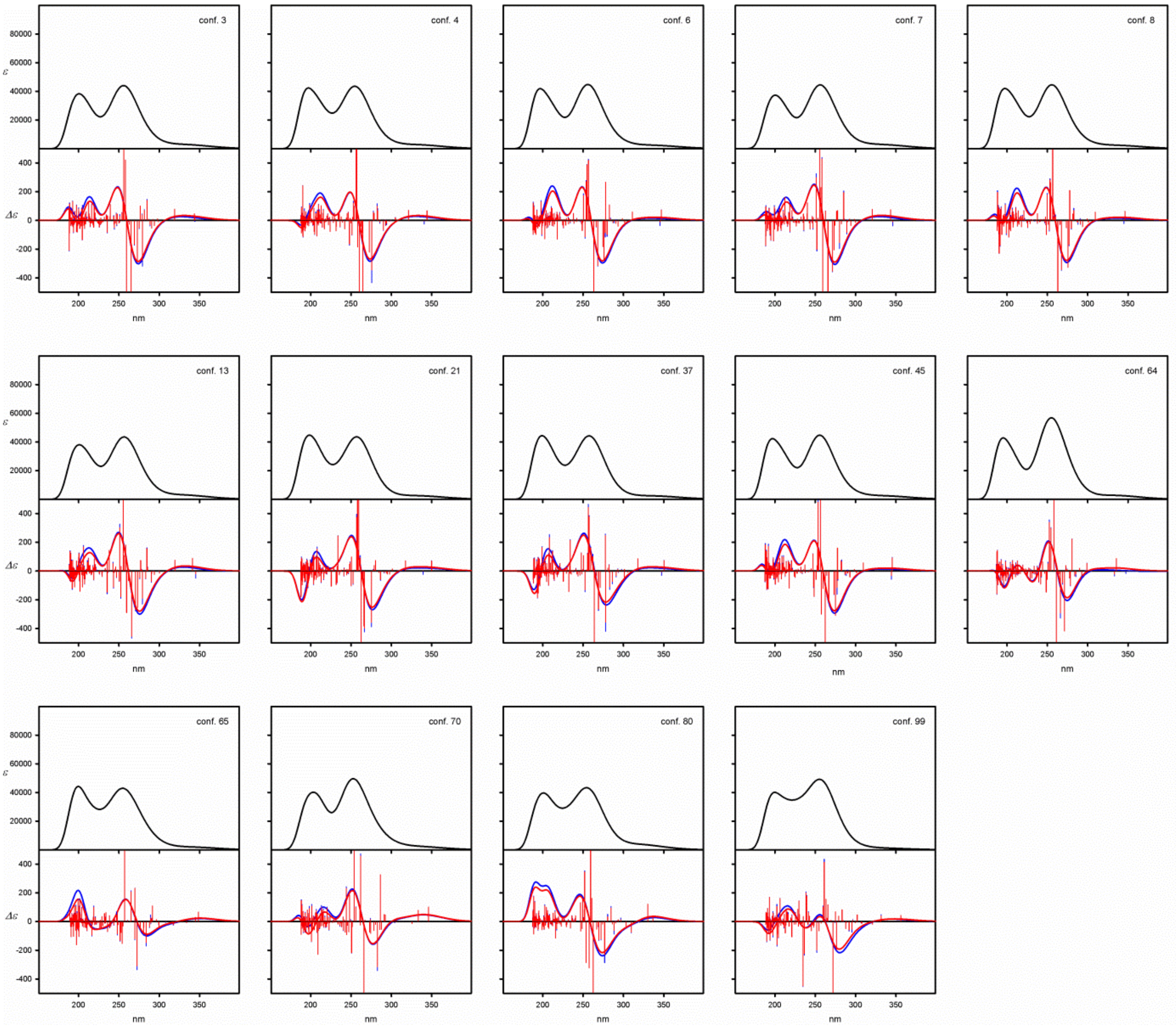

Figure S54. UV (upper panels) and ECD (lower panels) spectra calculated at the TD-M06-2X/6-311G(d,p) level for individual low-energy conformers of **6c**. Wavelengths were not corrected. Geometries were optimized at the M06L/6-31G(d,p) level. *Δε* values are given in mol^{-1} cm^{-1} dm^3.

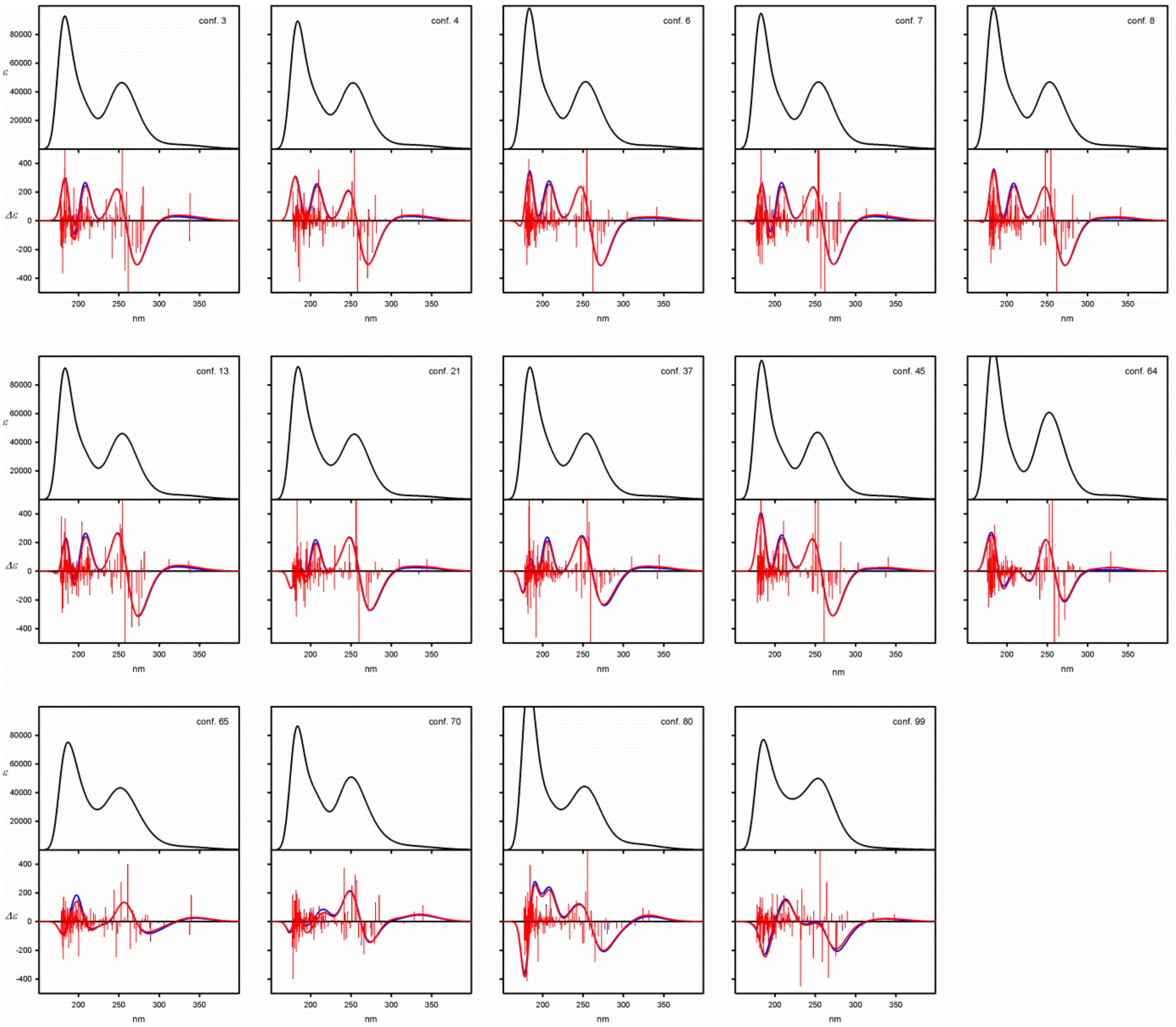

Figure S55. UV (upper panels) and ECD (lower panels) spectra calculated at the TD-wB97XD/6-311G(d,p) level for individual low-energy conformers of **6c**. Wavelengths were not corrected. Geometries were optimized at the M06L/6-31G(d,p) level. $\Delta\varepsilon$ values are given in mol^{-1} cm^{-1} dm^{3}.

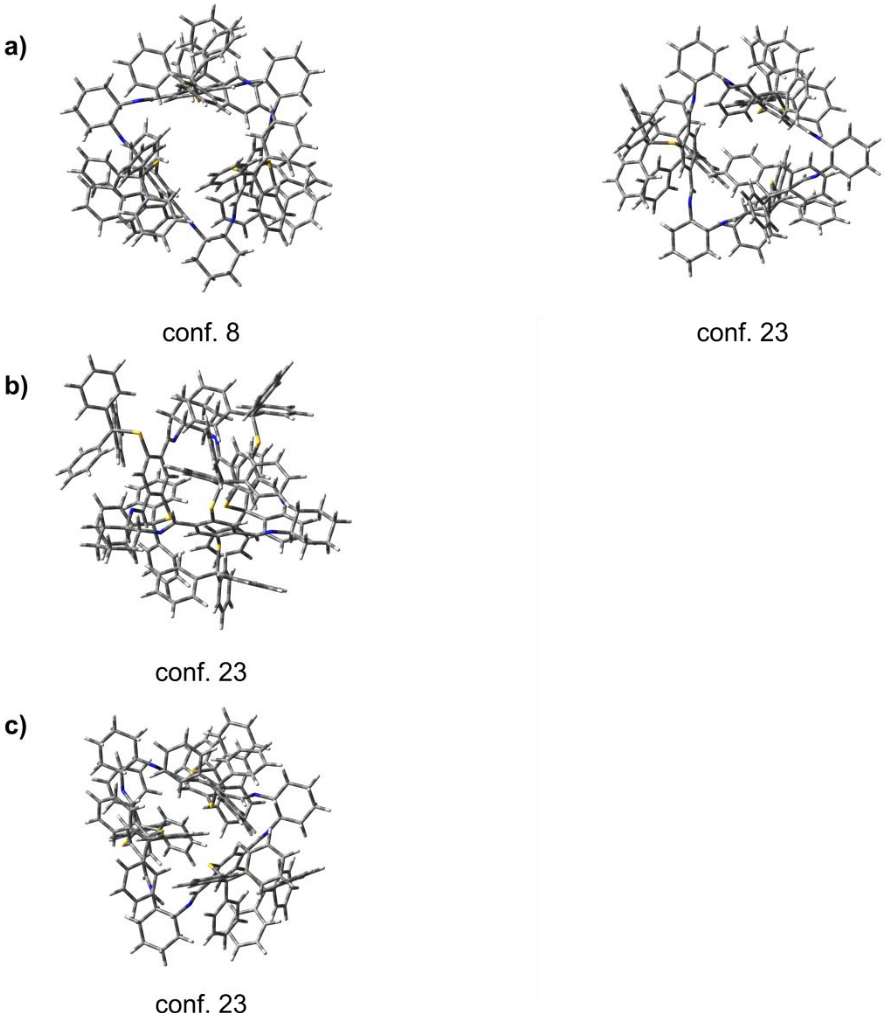

Figure S56. Structures of the low-energy conformers of **6f**, calculated at a) the B3LYP/6-31G(d), b) the B3LYP-GD3BJ/6-31G(d) and c) the M06L/6-31G(d) level of theory.

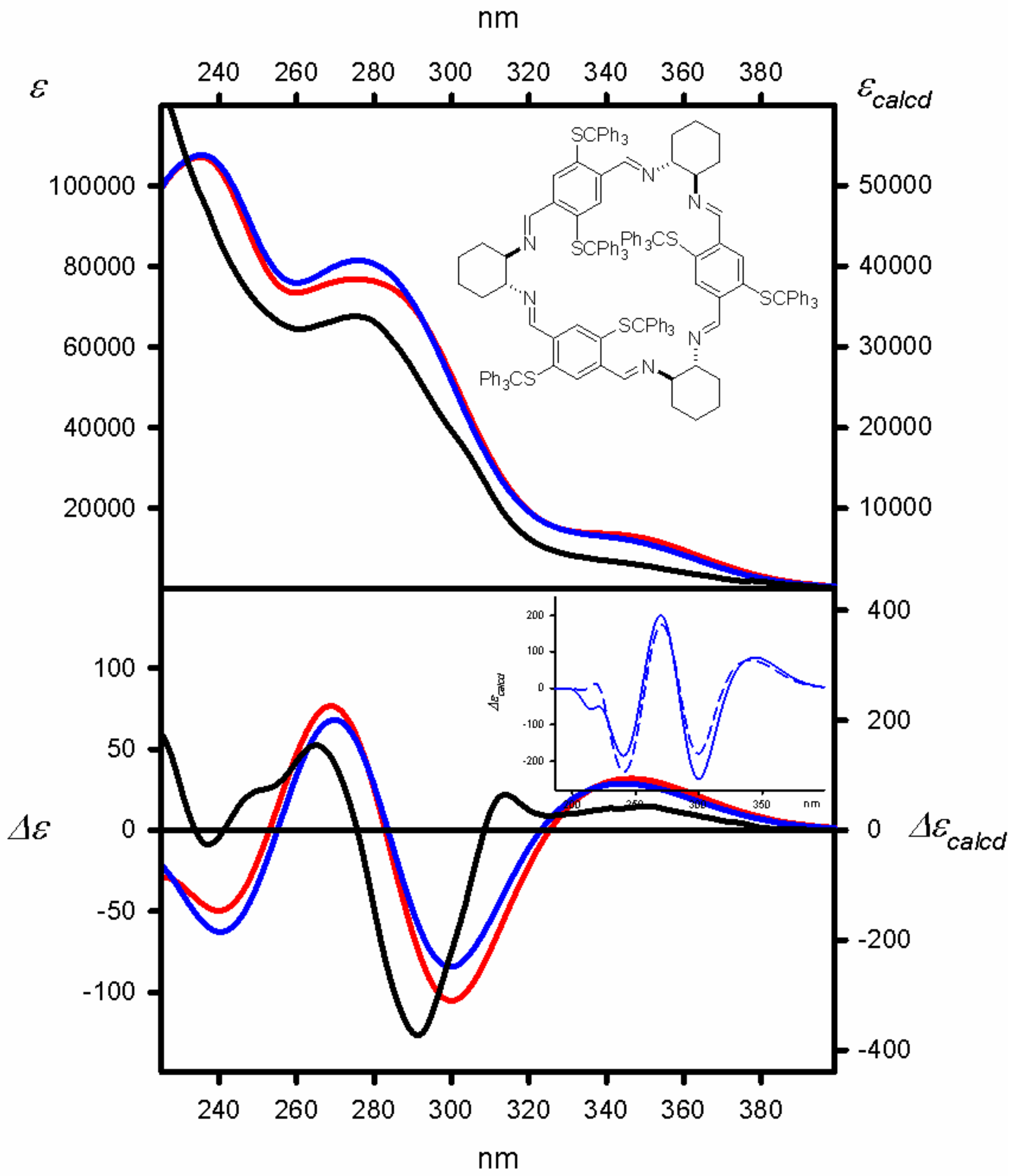

Experimental (dichloromethane, black lines)

Calculated at the
TD-CAM-B3LYP/6-31G(d,p) level and:
***ΔE*-based Boltzmann averaged (red lines)**
***ΔΔG*-based Boltzmann averaged (blue lines)**
Geometry optimized at the
B3LYP/6-31G(d) level

Figure S57. UV (upper panel) and ECD (lower panel) spectra of **6f** measured in dichloromethane (solid black lines) and calculated at the TD-CAM-B3LYP/6-31G(d,p) level for geometries optimized at the B3LYP/6-31G(d) level. The calculated ECD spectra were Boltzmann-averaged based on *ΔE* (red lines) and *ΔΔG* values (blue lines). Wavelengths were corrected to match the experimental UV maxima. The insert shows the comparison between the ECD spectra calculated for the lowest energy conformer of a given compound (dashed blue lines) and the *ΔΔG*-based and Boltzmann averaged (solid blue lines). *Δε* values are given in mol^{-1} cm^{-1} dm^{3}.

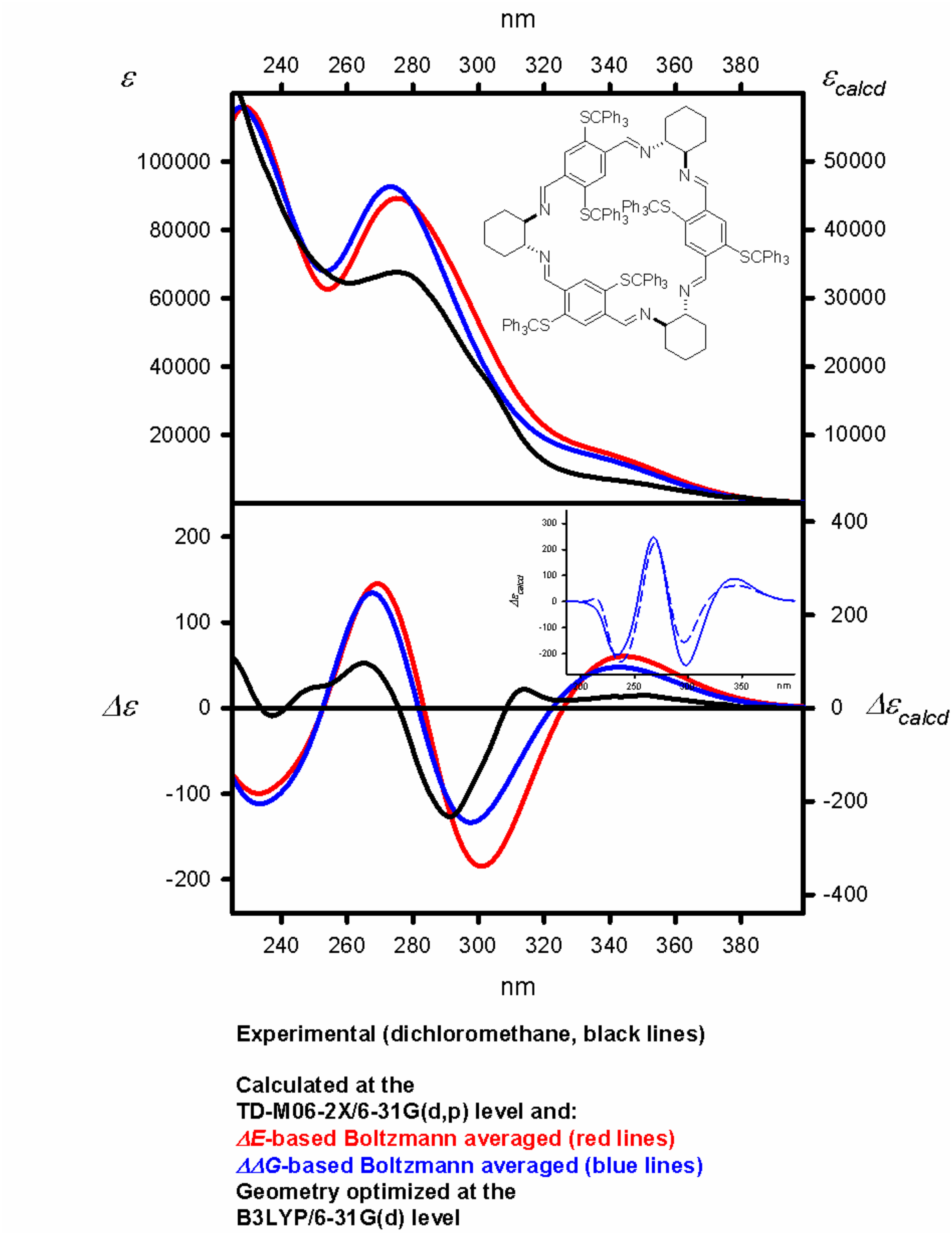

Figure S58. UV (upper panel) and ECD (lower panel) spectra of **6f** measured in dichloromethane (solid black lines) and calculated at the TD-M06-2X/6-31G(d,p) level for geometries optimized at the B3LYP/6-31G(d) level. The calculated ECD spectra were Boltzmann-averaged based on *ΔE* (red lines) and *ΔΔG* values (blue lines). Wavelengths were corrected to match the experimental UV maxima. The insert shows the comparison between the ECD spectra calculated for the lowest energy conformer of a given compound (dashed blue lines) and the *ΔΔ*G-based and Boltzmann averaged (solid blue lines). *Δε* values are given in mol^{-1} cm^{-1} dm^3.

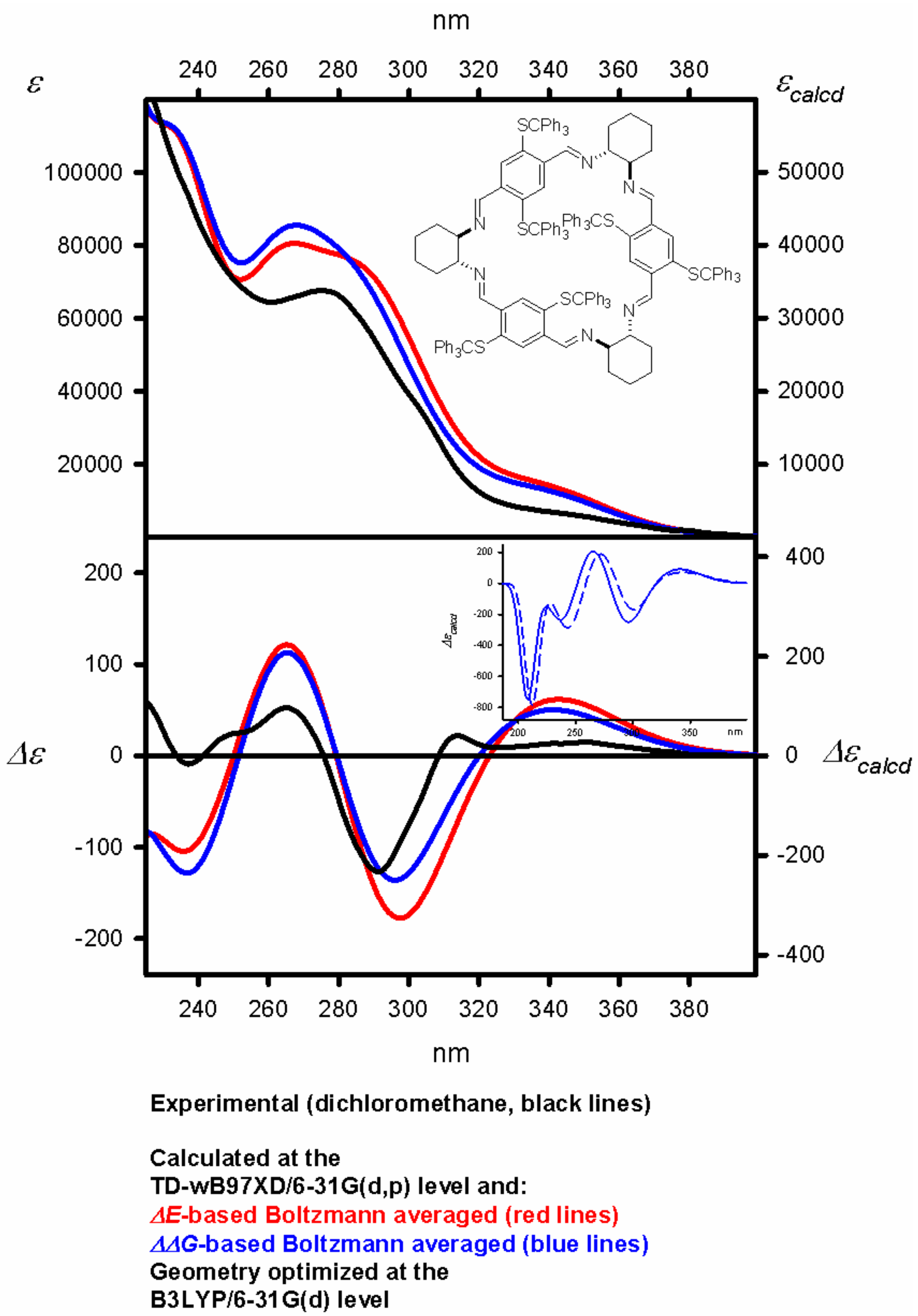

Figure S59. UV (upper panel) and ECD (lower panel) spectra of **6f** measured in dichloromethane (solid black lines) and calculated at the TD-wB97XD/6-31G(d,p) level for geometries optimized at the B3LYP/6-31G(d) level. The calculated ECD spectra were Boltzmann-averaged based on *ΔE* (red lines) and *ΔΔG* values (blue lines). Wavelengths were corrected to match the experimental UV maxima. The insert shows the comparison between the ECD spectra calculated for the lowest energy conformer of a given compound (dashed blue lines) and the *ΔΔ*G-based and Boltzmann averaged (solid blue lines *Δε* values are given in mol^{-1} cm^{-1} dm^3.

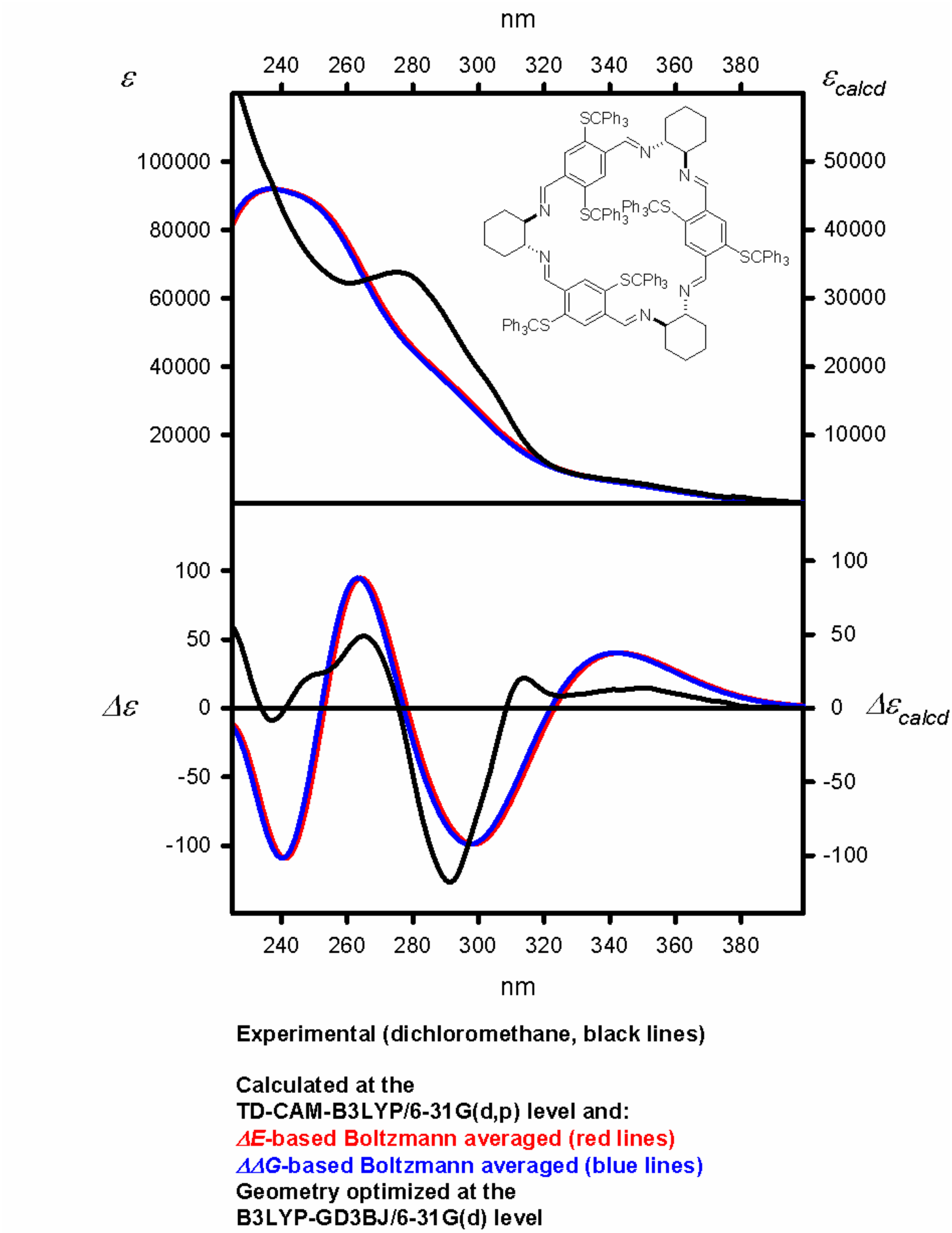

Figure S60. UV (upper panel) and ECD (lower panel) spectra of **6f** measured in dichloromethane (solid black lines) and calculated at the TD-CAM-B3LYP/6-31G(d,p) level for geometry optimized at the B3LYP-GD3BJ/6-31G(d) level. Wavelengths were corrected to match the experimental UV maxima. *Δε* values are given in mol^{-1} cm^{-1} dm^{3}.

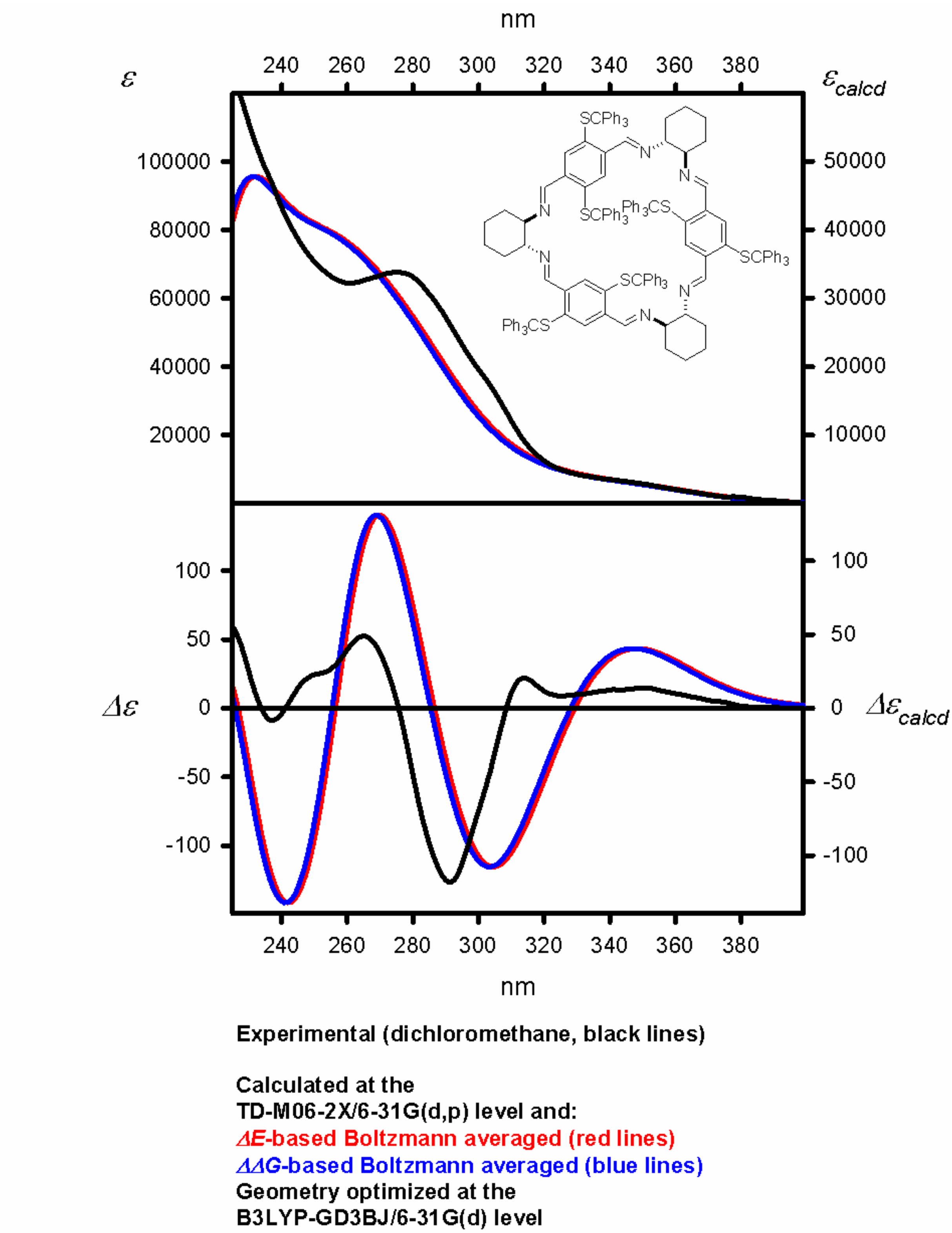

Figure S61. UV (upper panel) and ECD (lower panel) spectra of **6f** measured in dichloromethane (solid black lines) and calculated at the TD-M06-2X/6-31G(d,p) level for geometry optimized at the B3LYP-GD3BJ/6-31G(d) level. Wavelengths were corrected to match the experimental UV maxima. *Δε* values are given in mol^{-1} cm^{-1} dm^{3}.

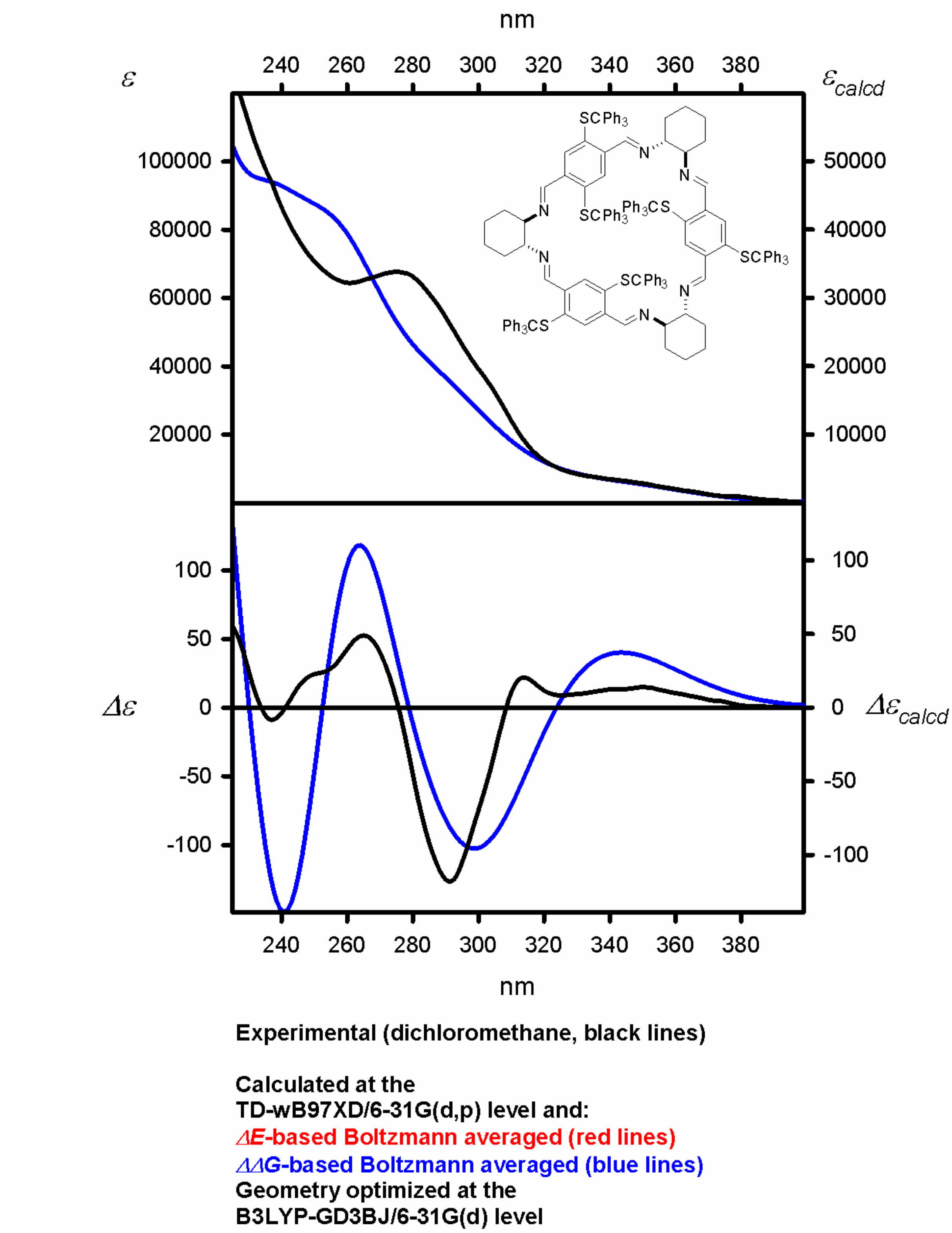

Figure S62. UV (upper panel) and ECD (lower panel) spectra of **6f** measured in dichloromethane (solid black lines) and calculated at the TD-wB97XD/6-31G(d,p) level for geometry optimized at the B3LYP-GD3BJ/6-31G(d) level. Wavelengths were corrected to match the experimental UV maxima. $\Delta\varepsilon$ values are given in mol^{-1} cm^{-1} dm^{3}.

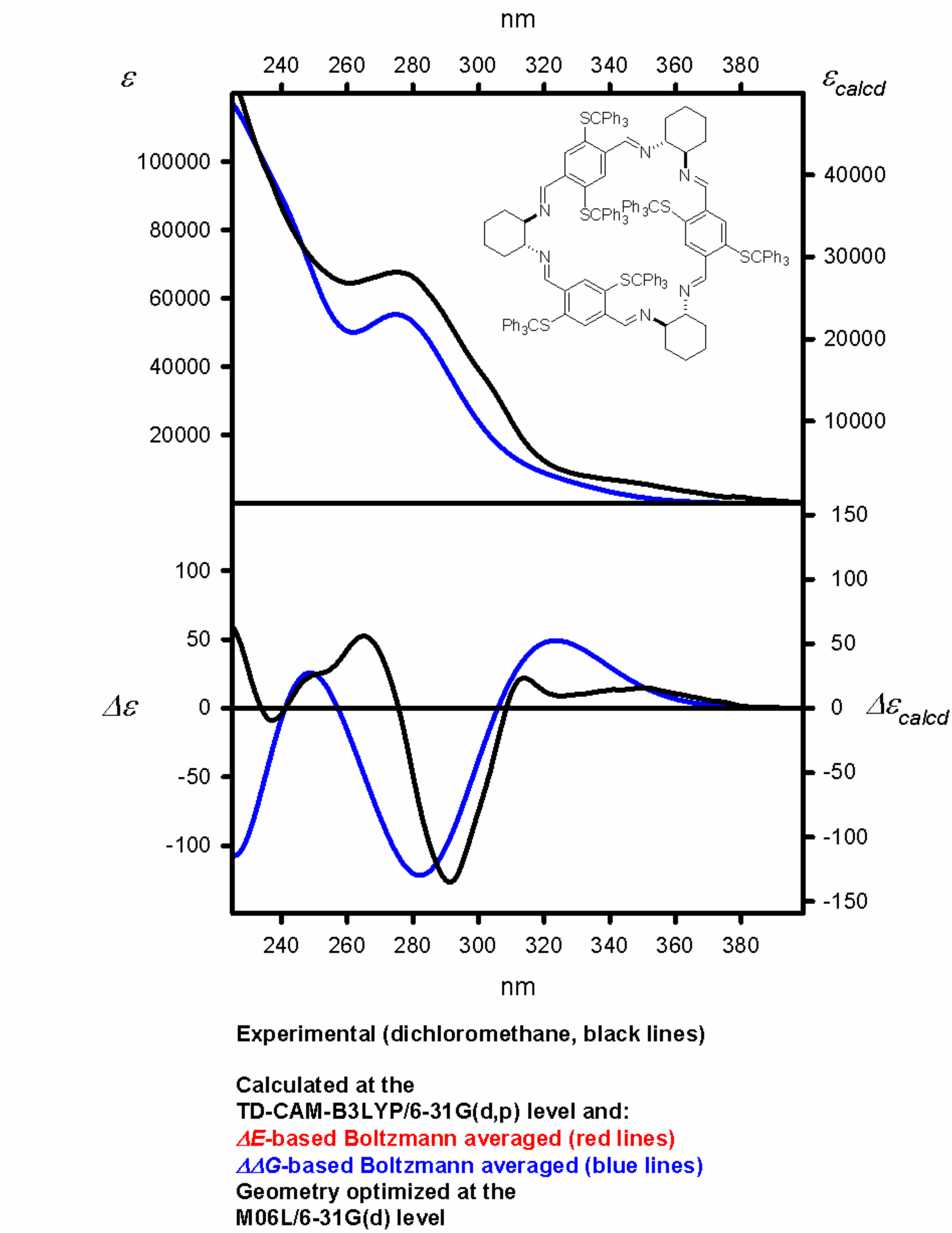

Figure S63. UV (upper panel) and ECD (lower panel) spectra of **6f** measured in dichloromethane (solid black lines) and calculated at the TD-CAM-B3LYP/6-31G(d,p) level for geometry optimized at the M06L/6-31G(d) level. Wavelengths were corrected to match the experimental UV maxima. $\Delta\varepsilon$ values are given in mol^{-1} cm^{-1} dm^{3}.

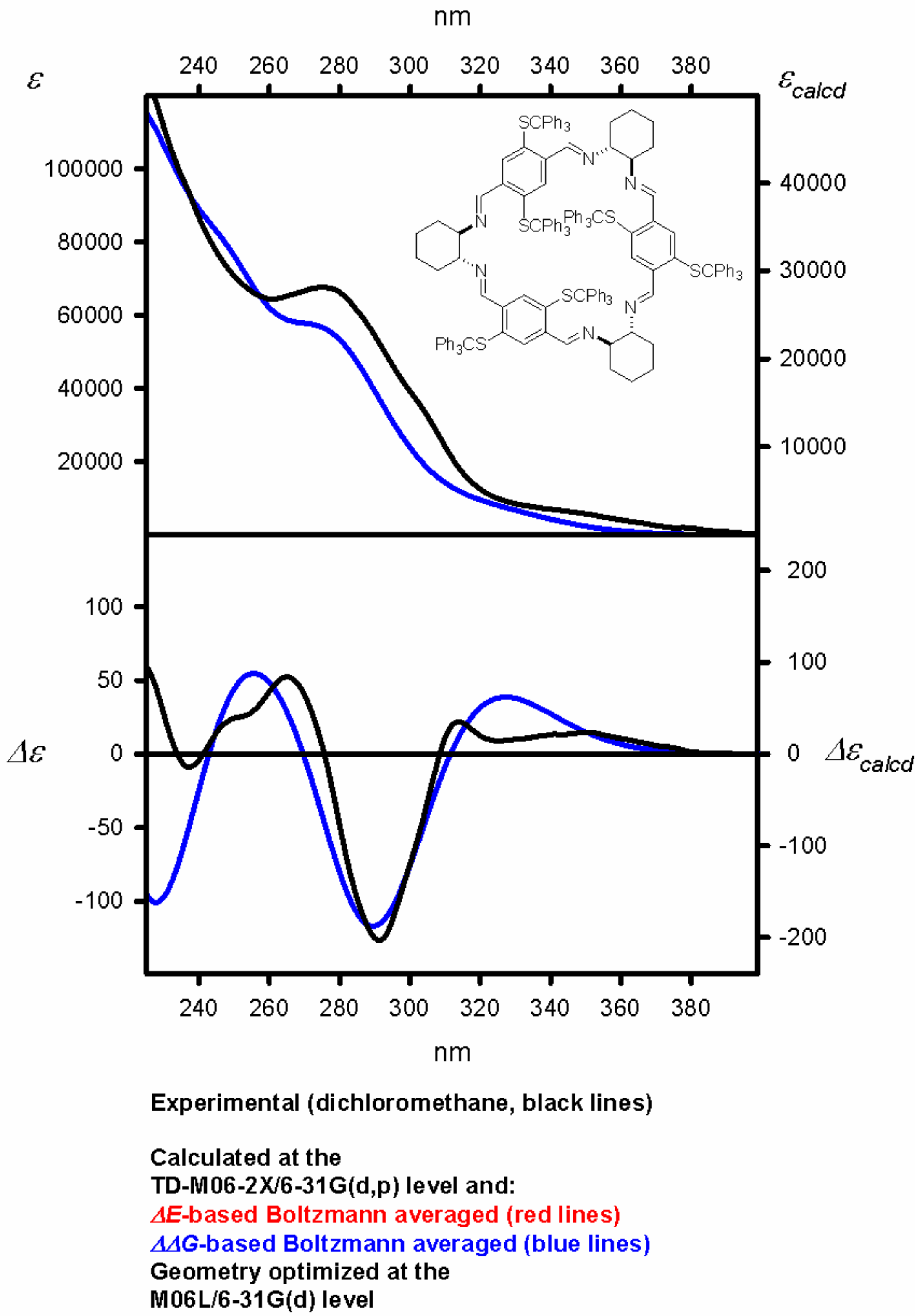

Figure S64. UV (upper panel) and ECD (lower panel) spectra of **6f** measured in dichloromethane (solid black lines) and calculated at the TD-M06-2X/6-31G(d,p) level for geometry optimized at the M06L/6-31G(d) level. Wavelengths were corrected to match the experimental UV maxima. *Δε* values are given in mol^{-1} cm^{-1} dm^{3}.

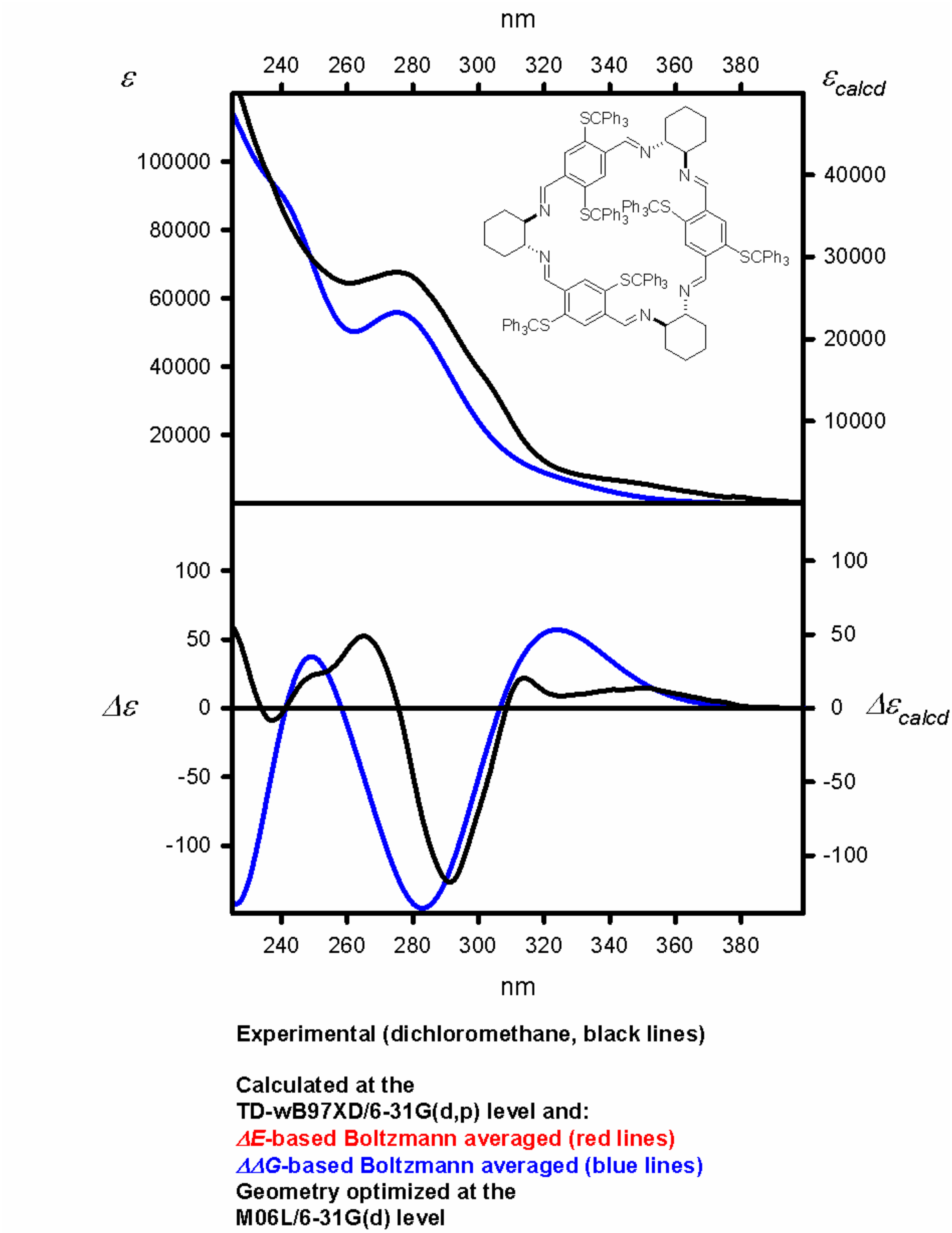

Figure S65. UV (upper panel) and ECD (lower panel) spectra of **6f** measured in dichloromethane (solid black lines) and calculated at the TD-wB97XD/6-31G(d,p) level for geometry optimized at the M06L/6-31G(d) level. Wavelengths were corrected to match the experimental UV maxima. *Δε* values are given in mol^{-1} cm^{-1} dm^{3}.

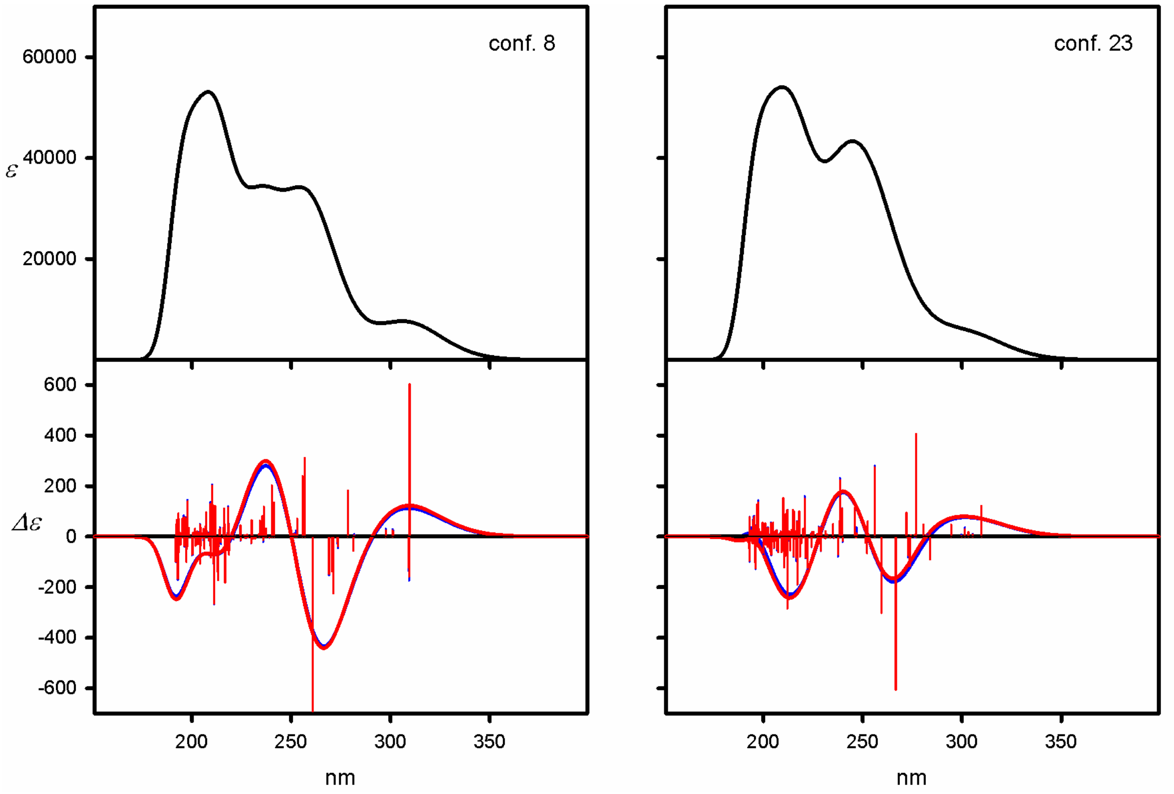

Figure S66. UV (upper panels) and ECD (lower panels) spectra calculated at the TD-CAM-B3LYP/6-31G(d,p) level for individual, low-energy conformers of **6f**. Wavelengths were not corrected. Geometries were optimized at the B3LYP/6-31G(d) level. *Δε* values are given in mol^{-1} cm^{-1} dm^{3}.

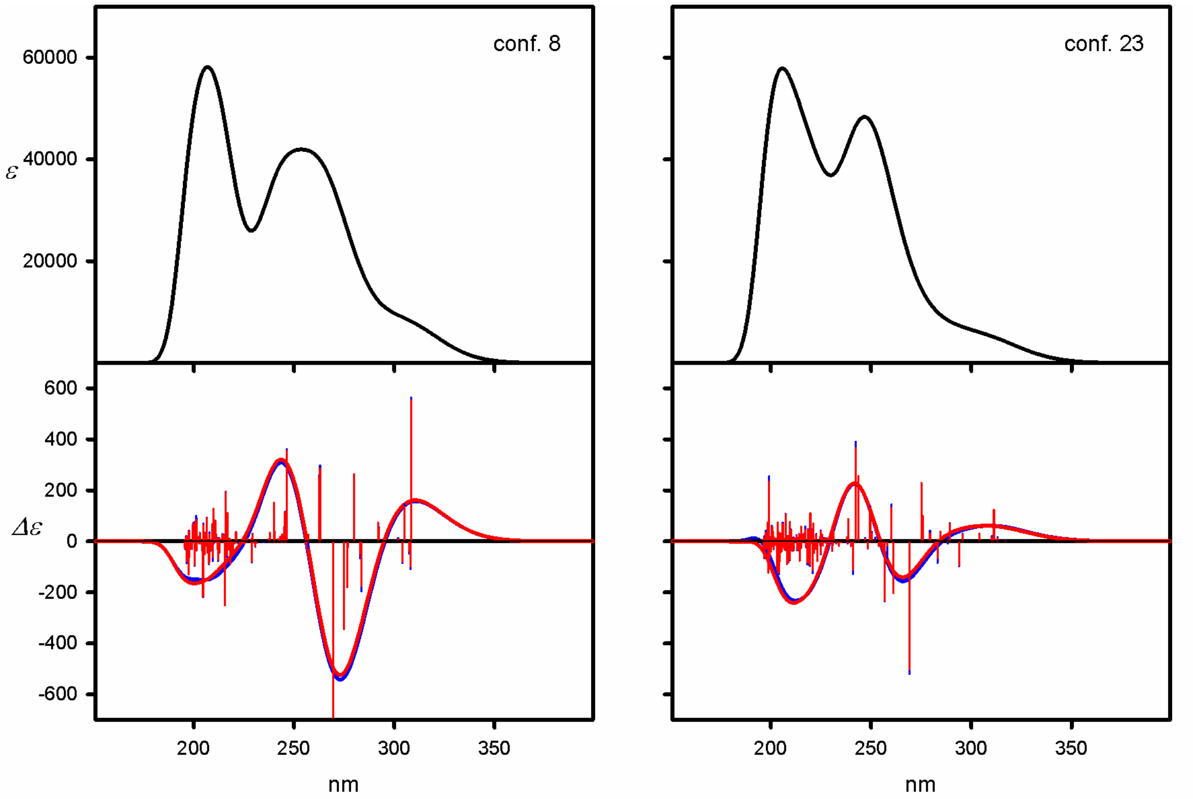

Figure S67. UV (upper panels) and ECD (lower panels) spectra calculated at the TD-M06-2X/6-31G(d,p) level for individual, low-energy conformers of **6f**. Wavelengths were not corrected. Geometries were optimized at the B3LYP/6-31G(d) level. *Δε* values are given in mol^{-1} cm^{-1} dm^{3}.

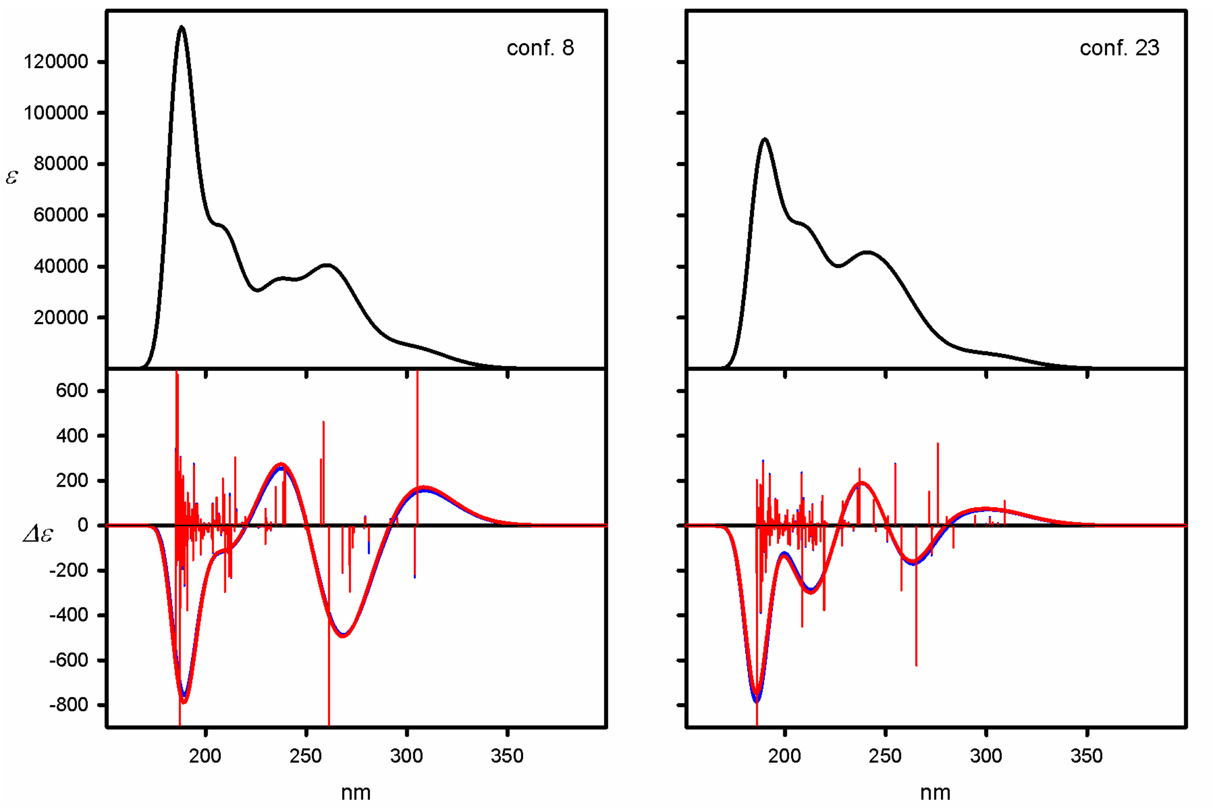

Figure S68. UV (upper panels) and ECD (lower panels) spectra calculated at the TD-wB97XD/6-31G(d,p) level for individual, low-energy conformers of **6f**. Wavelengths were not corrected. Geometries were optimized at the B3LYP/6-31G(d) level. *Δε* values are given in mol^{-1} cm^{-1} dm^3.

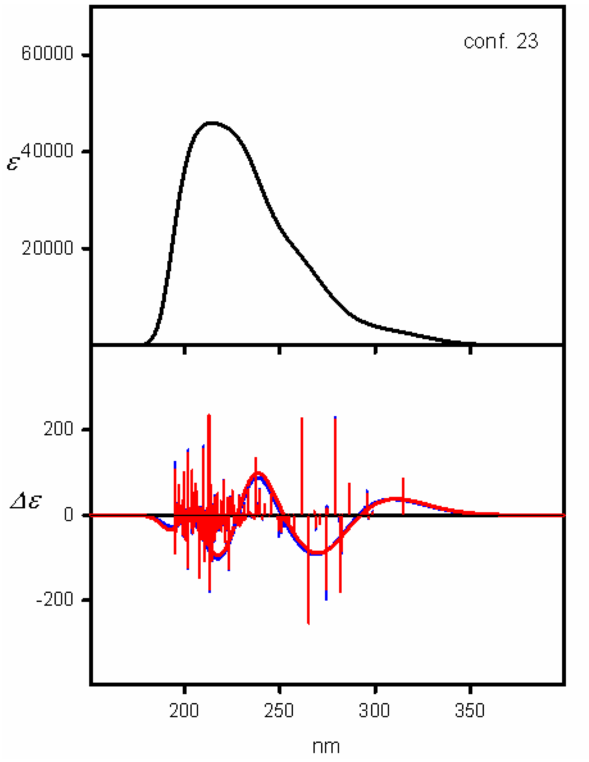

Figure S69. UV (upper panels) and ECD (lower panels) spectra calculated at the TD-CAM-B3LYP/6-31G(d,p) level for individual, low-energy conformers of **6f**. Wavelengths were not corrected. Geometries were optimized at the B3LYP-GD3BJ/6-31G(d) level. $\Delta\varepsilon$ values are given in mol^{-1} cm^{-1} dm^{3}.

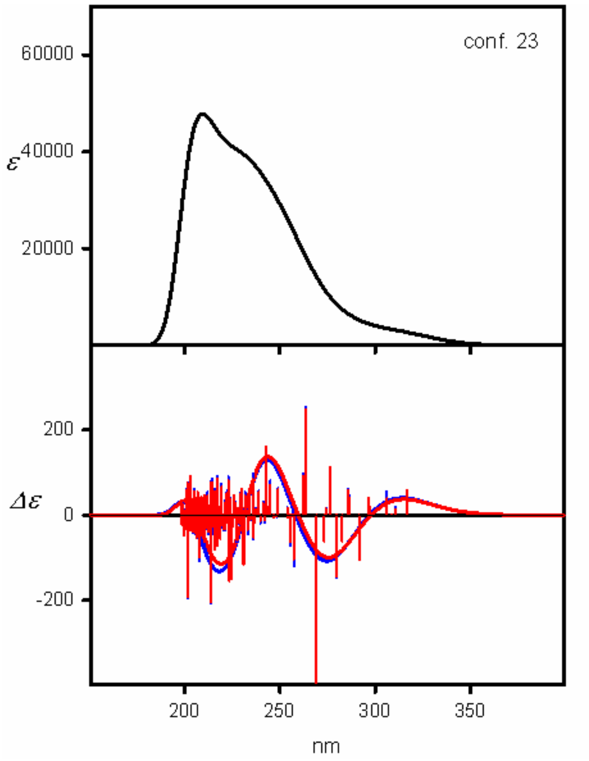

Figure S70. UV (upper panels) and ECD (lower panels) spectra calculated at the TD-M06-2X/6-31G(d,p) level for individual, low-energy conformers of **6f**. Wavelengths were not corrected. Geometries were optimized at the B3LYP-GD3BJ/6-31G(d) level. $\Delta\varepsilon$ values are given in mol^{-1} cm^{-1} dm^{3}.

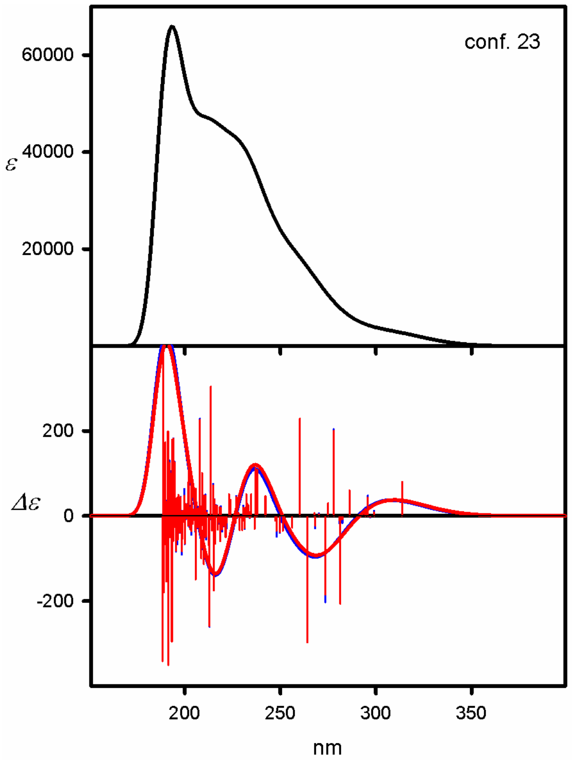

Figure S71. UV (upper panels) and ECD (lower panels) spectra calculated at the TD-wB97XD/6-31G(d,p) level for individual, low-energy conformers of **6f**. Wavelengths were not corrected. Geometries were optimized at the B3LYP-GD3BJ/6-31G(d) level. *Δε* values are given in mol^{-1} cm^{-1} dm^3.

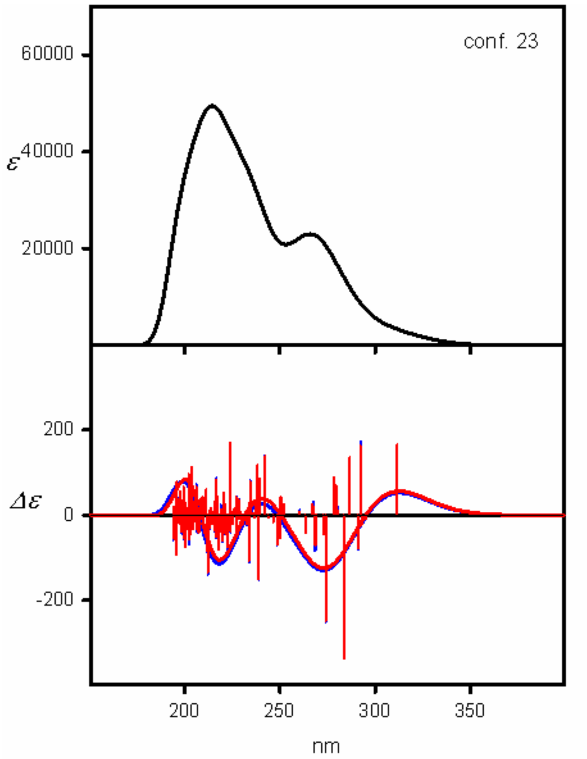

Figure S72. UV (upper panels) and ECD (lower panels) spectra calculated at the TD-CAM-B3LYP/6-31G(d,p) level for individual, low-energy conformers of **6f**. Wavelengths were not corrected. Geometries were optimized at the M06L/6-31G(d) level. *Δε* values are given in mol^{-1} cm^{-1} dm^3.

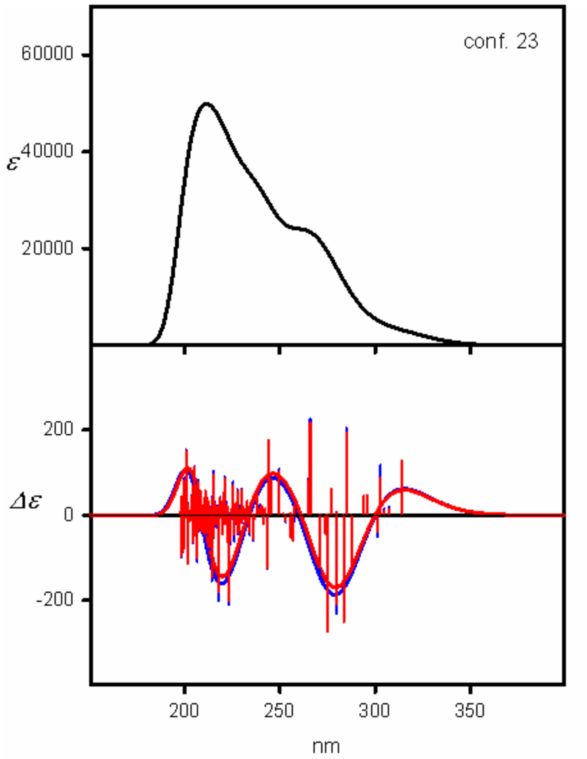

Figure S73. UV (upper panels) and ECD (lower panels) spectra calculated at the TD-M06-2X/6-31G(d,p) level for individual, low-energy conformers of **6f**. Wavelengths were not corrected. Geometries were optimized at the M06L/6-31G(d) level. *Δε* values are given in mol^{-1} cm^{-1} dm^{3}.

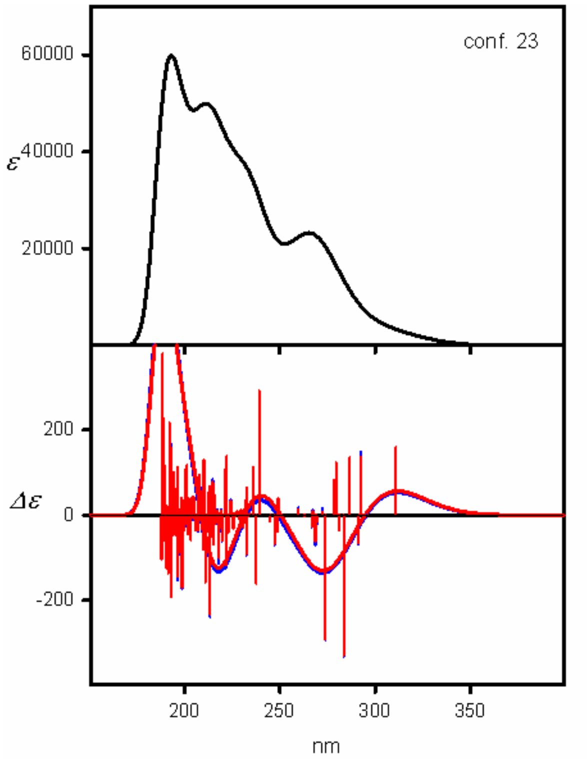

Figure S74. UV (upper panels) and ECD (lower panels) spectra calculated at the TD-wB97XD/6-31G(d,p) level for individual, low-energy conformers of **6f**. Wavelengths were not corrected. Geometries were optimized at the M06L/6-31G(d) level. *Δε* values are given in mol^{-1} cm^{-1} dm^{3}.

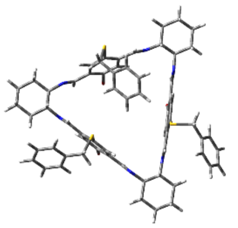

conf. sym-1

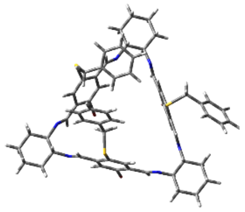

conf. sym-29

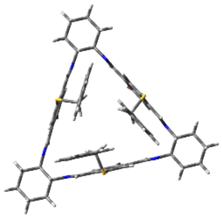

conf. sym-41

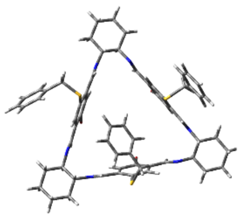

conf. sym-67

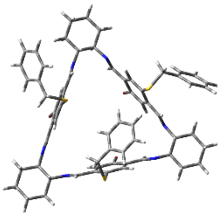

conf. sym-68

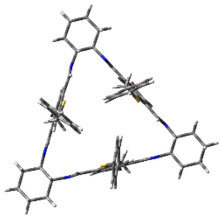

conf. sym-74

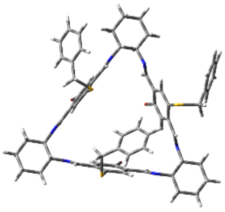

conf. sym-77

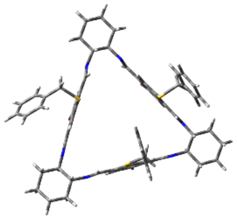

conf. sym-87

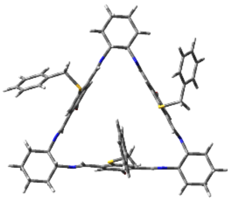

conf. sym-98

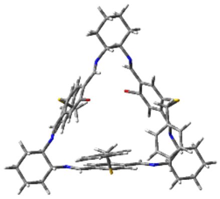

conf. 2

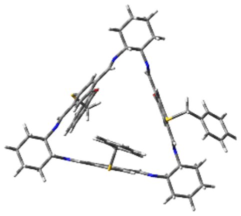

conf. 6

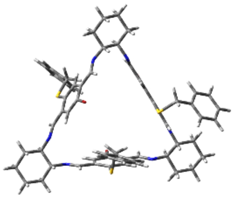

conf. 8

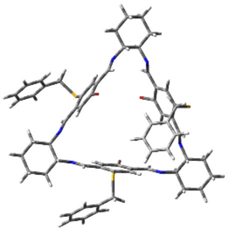

conf. 9

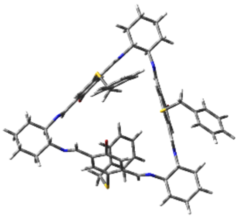

conf. 10

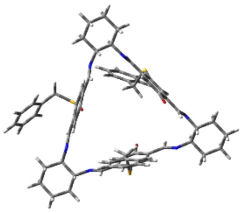

conf. 11

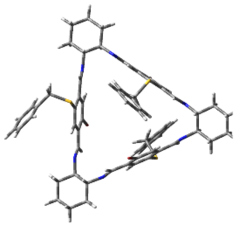

conf. 12

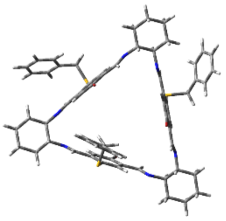

conf. 13

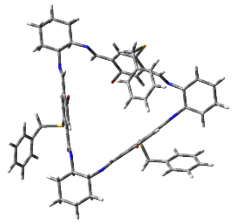

conf. 14

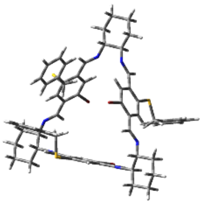

conf. 15

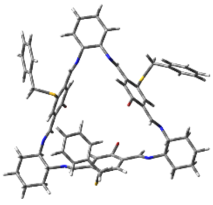

conf. 16

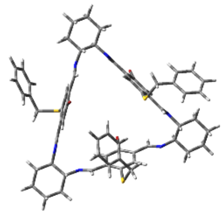

conf. 17

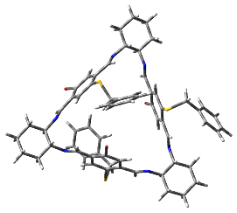

conf. 20

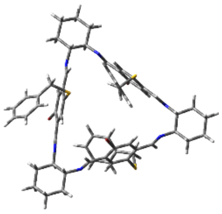

conf. 22

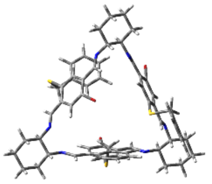

conf. 24

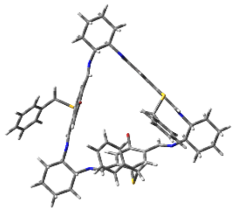

conf. 25

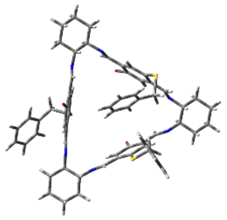

conf. 26

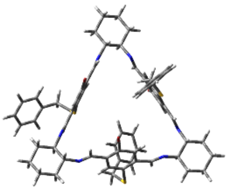

conf. 27

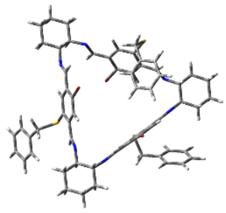

conf. 28

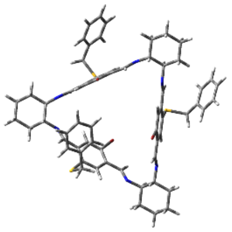

conf. 30

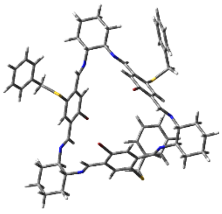

conf. 31

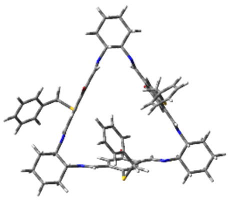

conf. 34

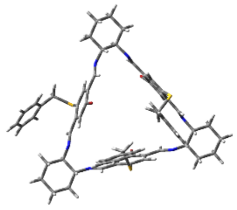

conf. 36

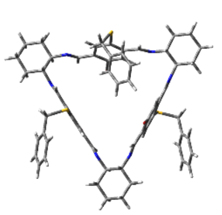

conf. 37

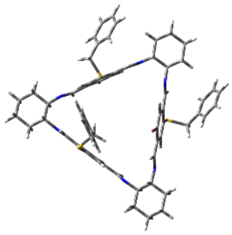

conf. 40

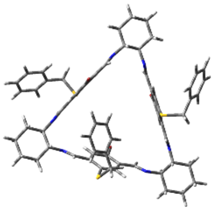

conf. 41

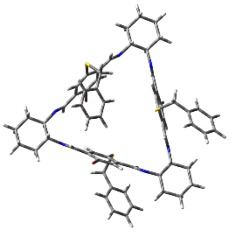

conf. 44

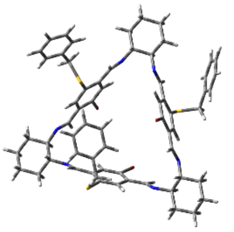
conf. 45

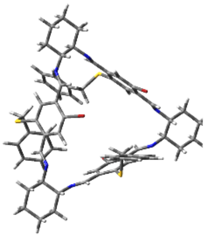
conf. 46

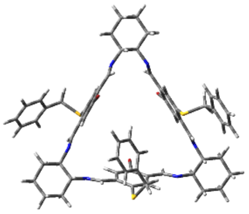
conf. 48

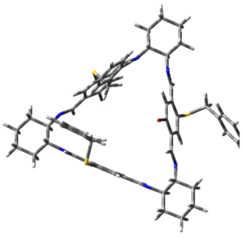
conf. 49

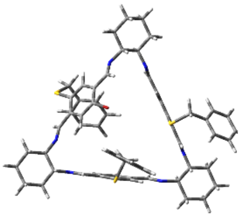
conf. 52

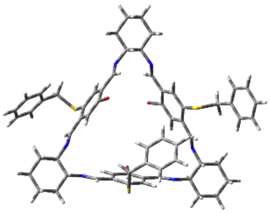
conf. 53

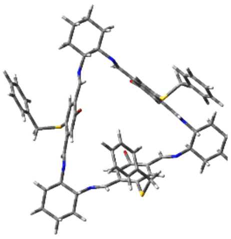
conf. 58

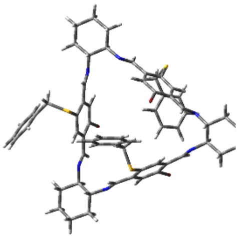
conf. 59

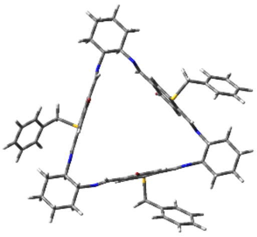
conf. 61

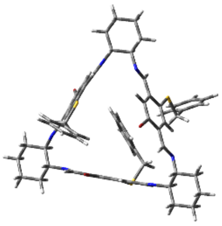
conf. 62

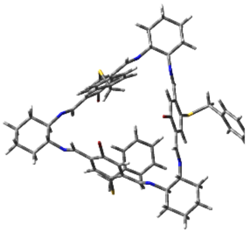
conf. 63

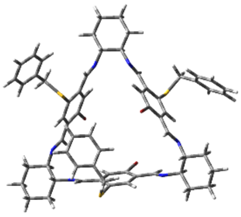
conf. 64

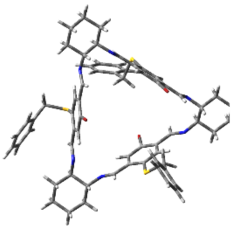
conf. 65

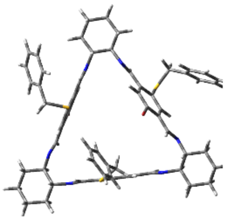
conf. 66

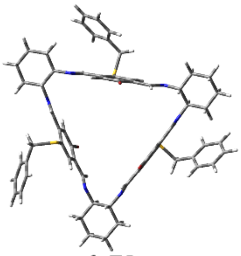
conf. 72

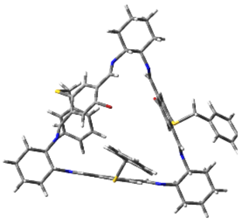
conf. 73

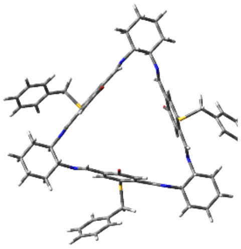
conf. 75

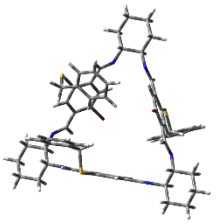
conf. 76

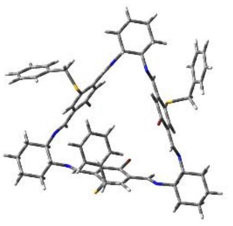

conf. 77

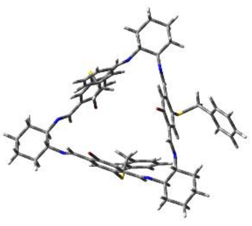

conf. 78

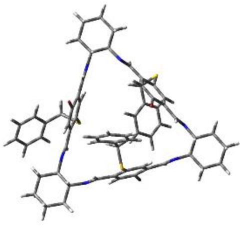

conf. 79

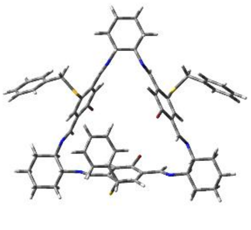

conf. 82

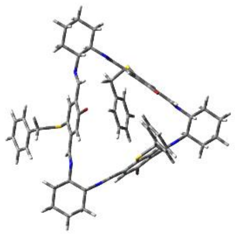

conf. 83

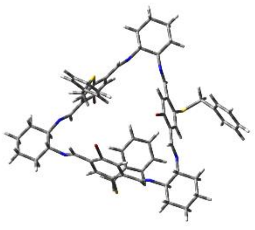

conf. 84

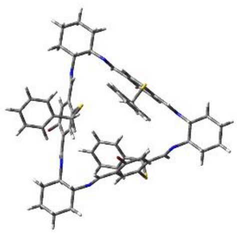

conf. 87

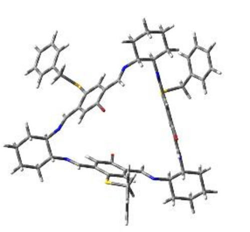

conf. 89

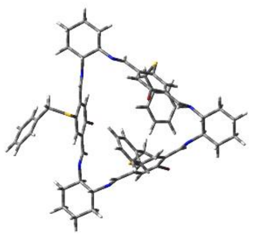

conf. 90

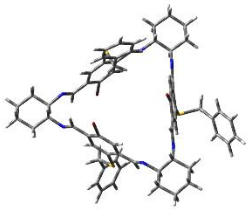

conf. 92

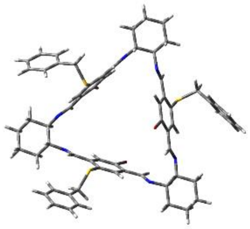

conf. 94

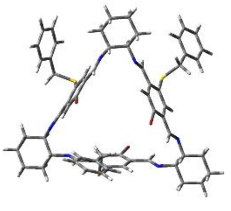

conf. 95

Figure S75. Structures of the low-energy conformers of **6g**, calculated at the B3LYP/6-311G(d,p) level. Prefix “sym” denotes a symmetrical conformer.

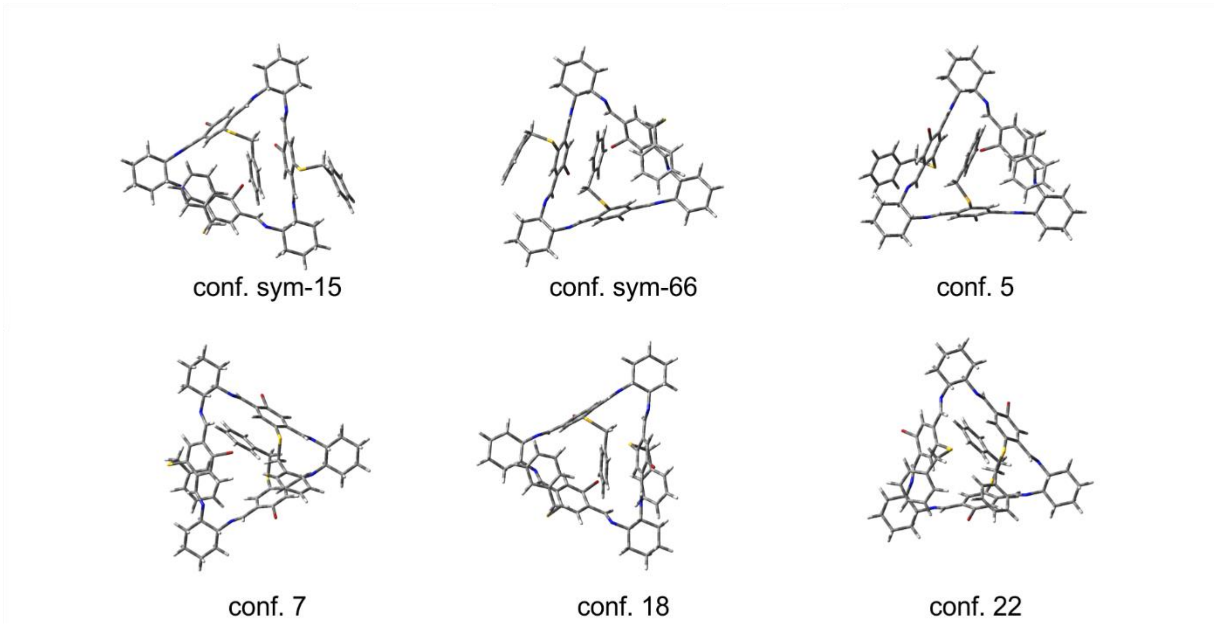

Figure S76. Structures of the low-energy conformers of **6g**, calculated at the B3LYP-GD3BJ/6-311G(d,p) level. Prefix "sym" denotes a symmetrical conformer.

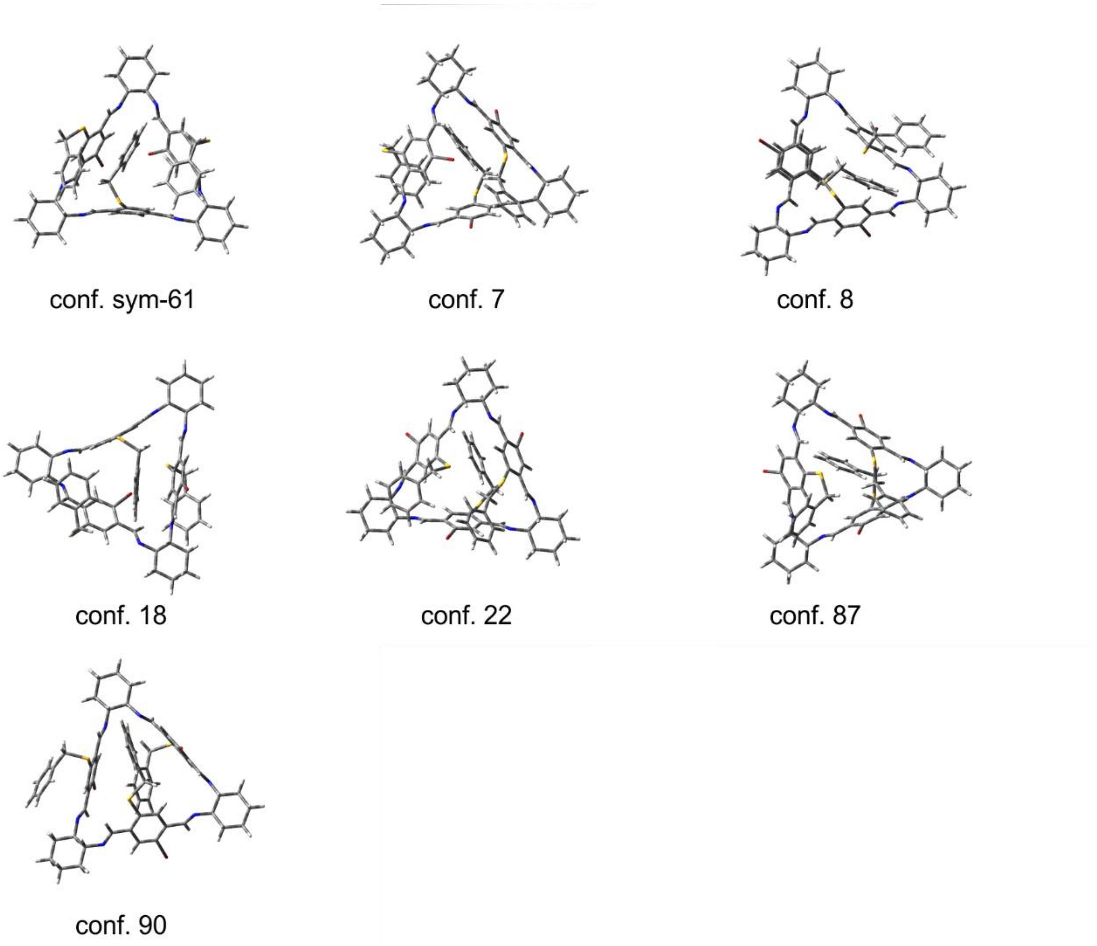

Figure S77. Structures of the low-energy conformers of **6g**, calculated at the M06L/6-311G(d,p) level. Prefix "sym" denotes a symmetrical conformer.

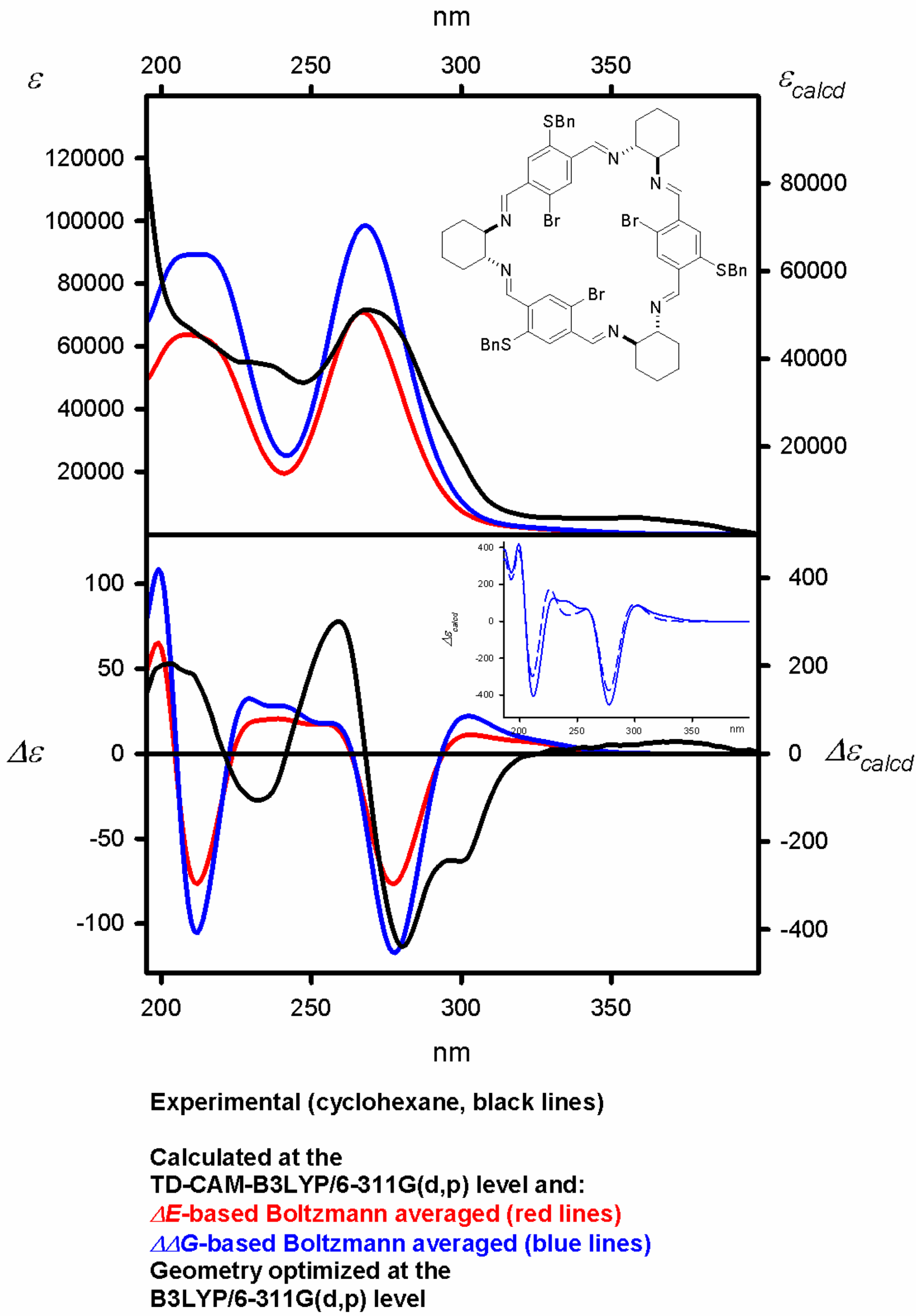

Figure S78. UV (upper panel) and ECD (lower panel) spectra of **6g** measured in cyclohexane (solid black lines) and calculated at the TD-CAM-B3LYP/6-311G(d,p) level for geometries optimized at the B3LYP/6-311G(d,p) level. The calculated ECD spectra were Boltzmann-averaged based on ΔE (red lines) and $\Delta\Delta G$ values (blue lines). Wavelengths were corrected to match the experimental UV maxima. The insert shows the comparison between the ECD spectra calculated for the lowest energy conformer of a given compound (dashed blue lines) and the $\Delta\Delta$G-based and Boltzmann averaged (solid blue lines). $\Delta\varepsilon$ values are given in mol^{-1} cm^{-1} dm^{3}.

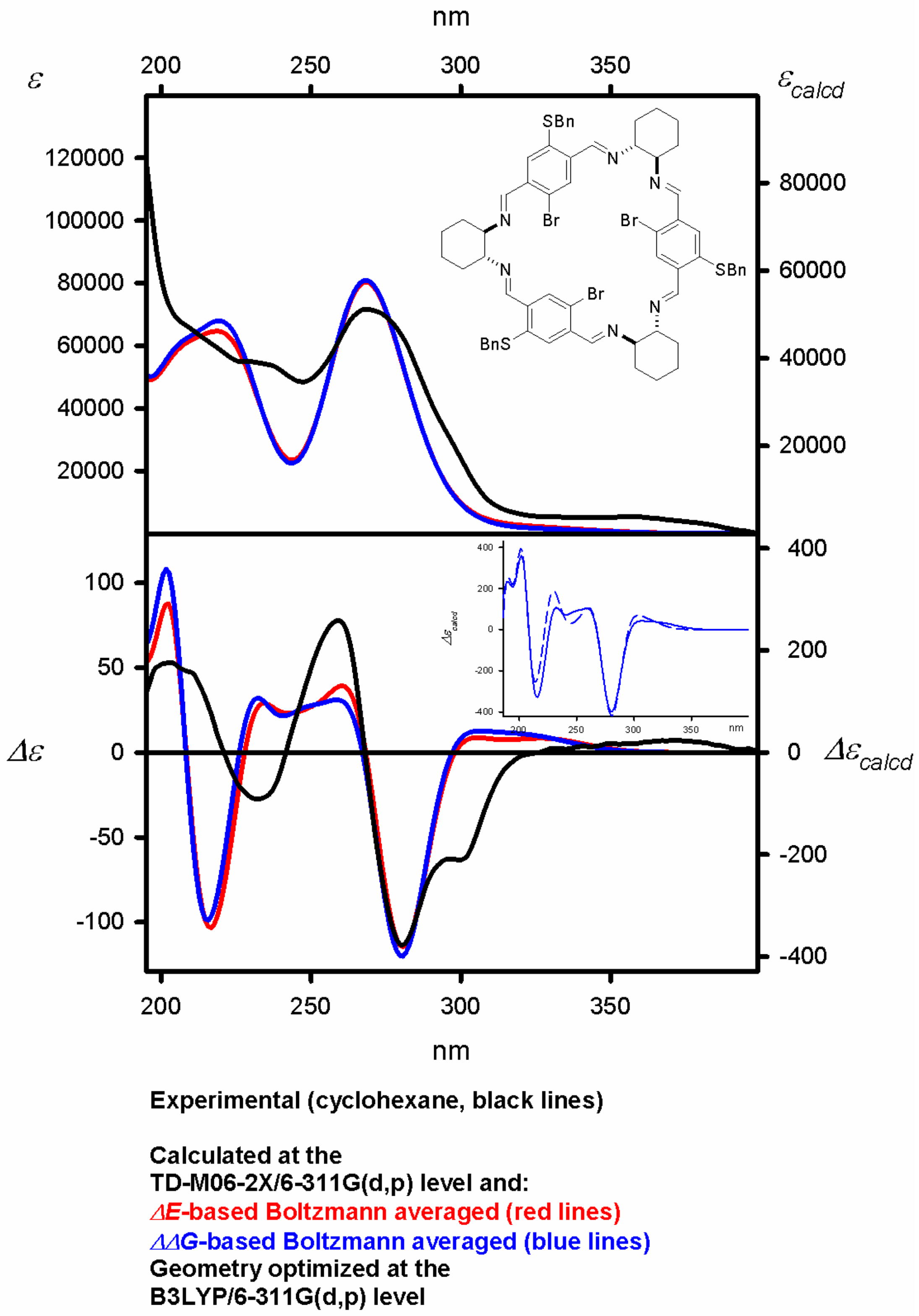

Figure S79. UV (upper panel) and ECD (lower panel) spectra of **6g** measured in cyclohexane (solid black lines) and calculated at the TD-M06-2X/6-311G(d,p) level for geometries optimized at the B3LYP/6-311G(d,p) level. The calculated ECD spectra were Boltzmann-averaged based on ΔE (red lines) and $\Delta\Delta G$ values (blue lines). Wavelengths were corrected to match the experimental UV maxima. The insert shows the comparison between the ECD spectra calculated for the lowest energy conformer of a given compound (dashed blue lines) and the $\Delta\Delta$G-based and Boltzmann averaged (solid blue lines). $\Delta\varepsilon$ values are given in mol^{-1} cm^{-1} dm^{3}.

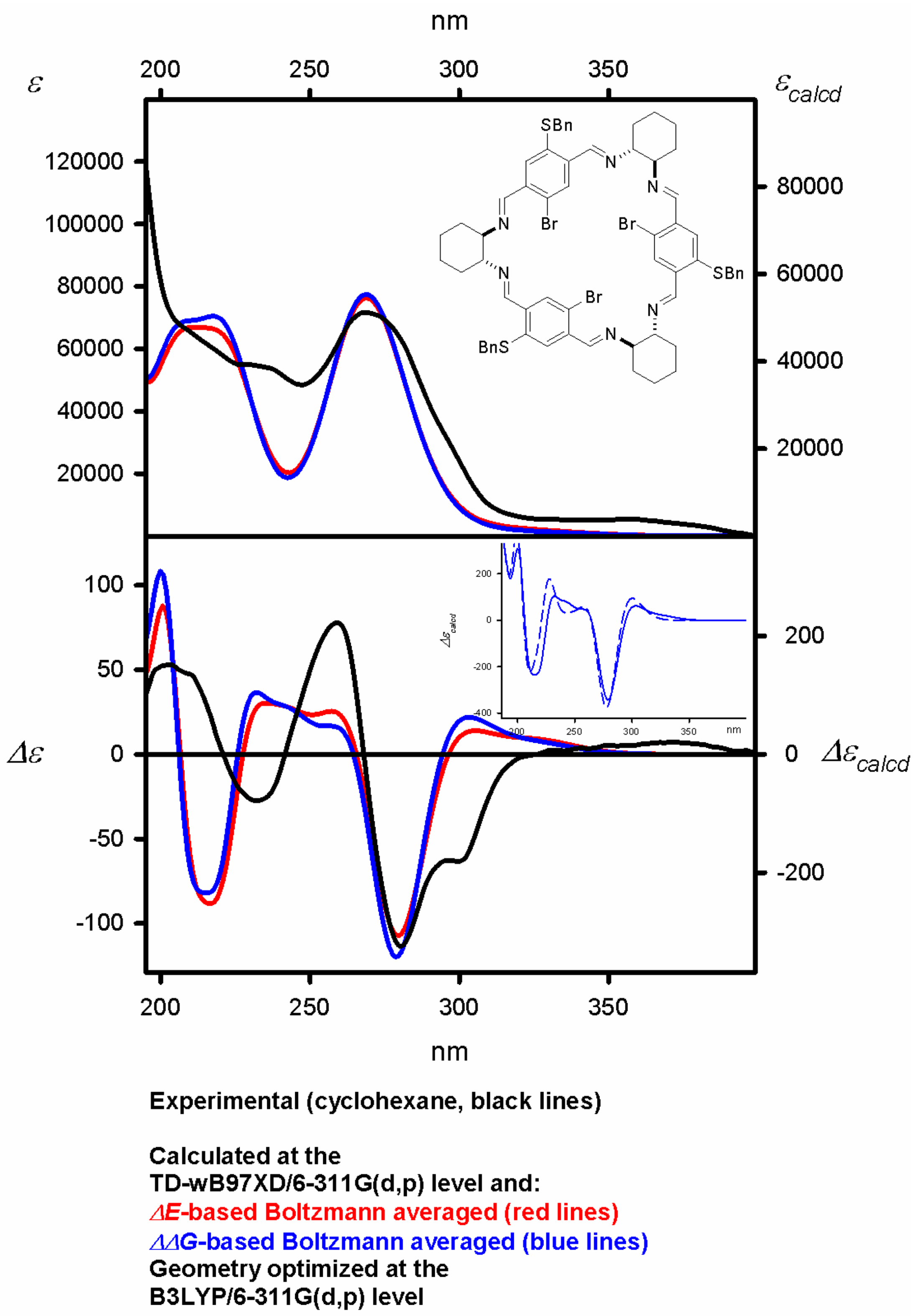

Figure S80. UV (upper panel) and ECD (lower panel) spectra of **6g** measured in cyclohexane (solid black lines) and calculated at the TD-wB97XD/6-311G(d,p) level for geometries optimized at the B3LYP/6-311G(d,p) level. The calculated ECD spectra were Boltzmann-averaged based on *ΔE* (red lines) and *ΔΔG* values (blue lines). Wavelengths were corrected to match the experimental UV maxima. The insert shows the comparison between the ECD spectra calculated for the lowest energy conformer of a given compound (dashed blue lines) and the *ΔΔ*G-based and Boltzmann averaged (solid blue lines). *Δε* values are given in mol^{-1} cm^{-1} dm^{3}.

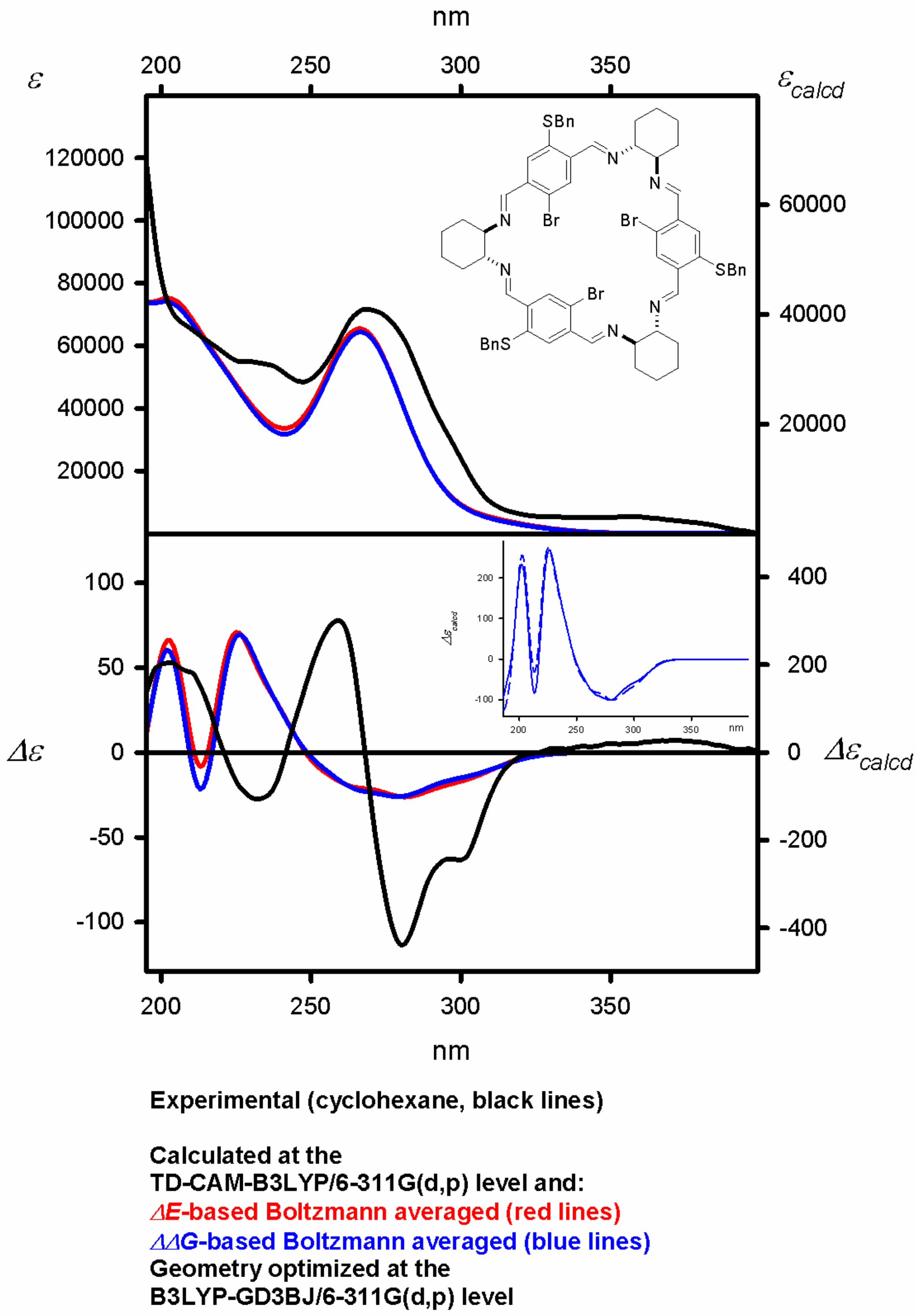

Figure S81. UV (upper panel) and ECD (lower panel) spectra of **6g** measured in cyclohexane (solid black lines) and calculated at the TD-CAM-B3LYP/6-311G(d,p) level for geometries optimized at the B3LYP-GD3BJ/6-311G(d,p) level. The calculated ECD spectra were Boltzmann-averaged based on ΔE (red lines) and $\Delta\Delta G$ values (blue lines). Wavelengths were corrected to match the experimental UV maxima. The insert shows the comparison between the ECD spectra calculated for the lowest energy conformer of a given compound (dashed blue lines) and the $\Delta\Delta$G-based and Boltzmann averaged (solid blue lines). $\Delta\varepsilon$ values are given in mol^{-1} cm^{-1} dm^{3}.

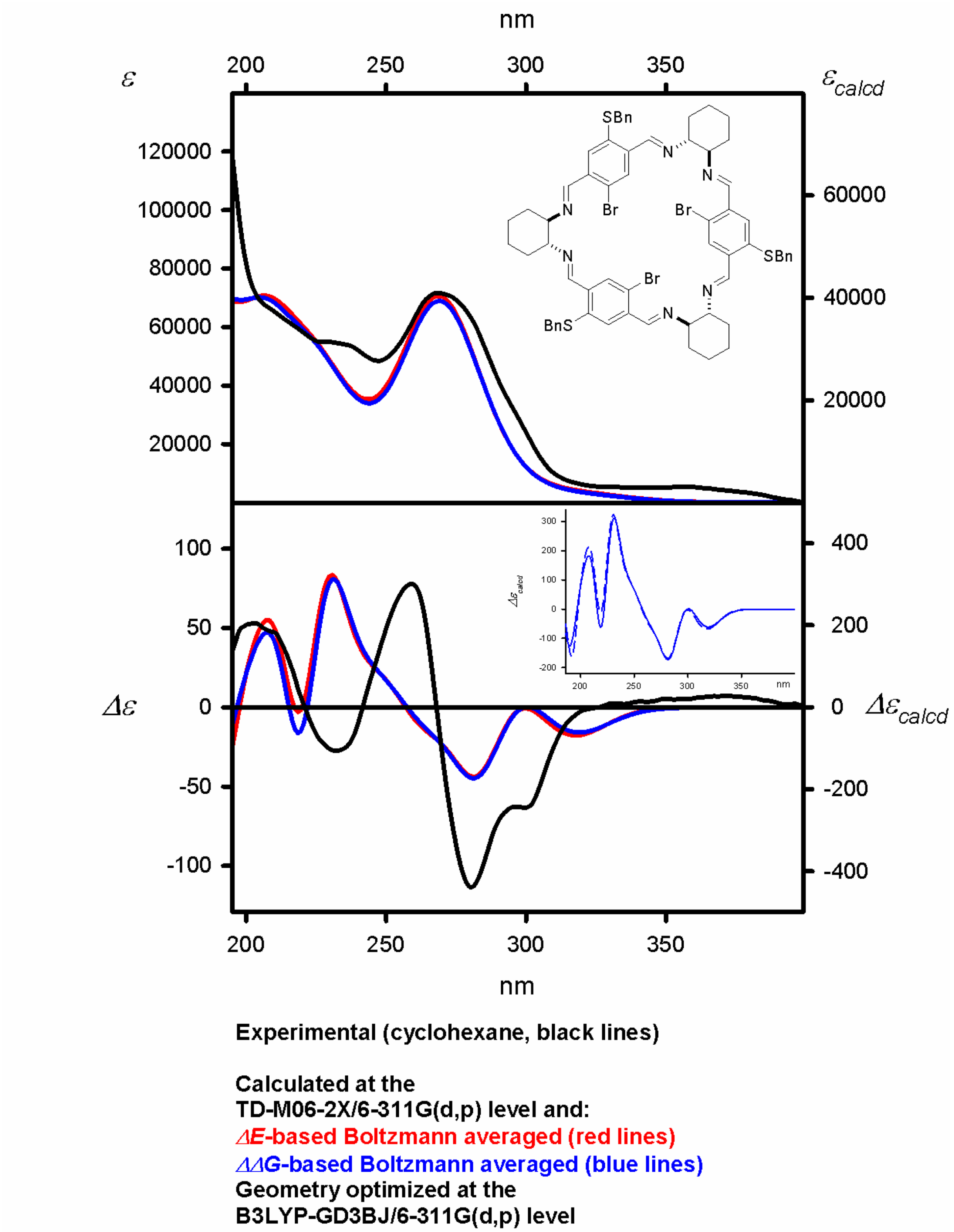

Figure S82. UV (upper panel) and ECD (lower panel) spectra of **6g** measured in cyclohexane (solid black lines) and calculated at the TD-M06-2X/6-311G(d,p) level for geometries optimized at the B3LYP-GD3BJ/6-311G(d,p) level. The calculated ECD spectra were Boltzmann-averaged based on *ΔE* (red lines) and *ΔΔG* values (blue lines). Wavelengths were corrected to match the experimental UV maxima. The insert shows the comparison between the ECD spectra calculated for the lowest energy conformer of a given compound (dashed blue lines) and the *ΔΔ*G-based and Boltzmann averaged (solid blue lines). *Δε* values are given in mol^{-1} cm^{-1} dm^{3}.

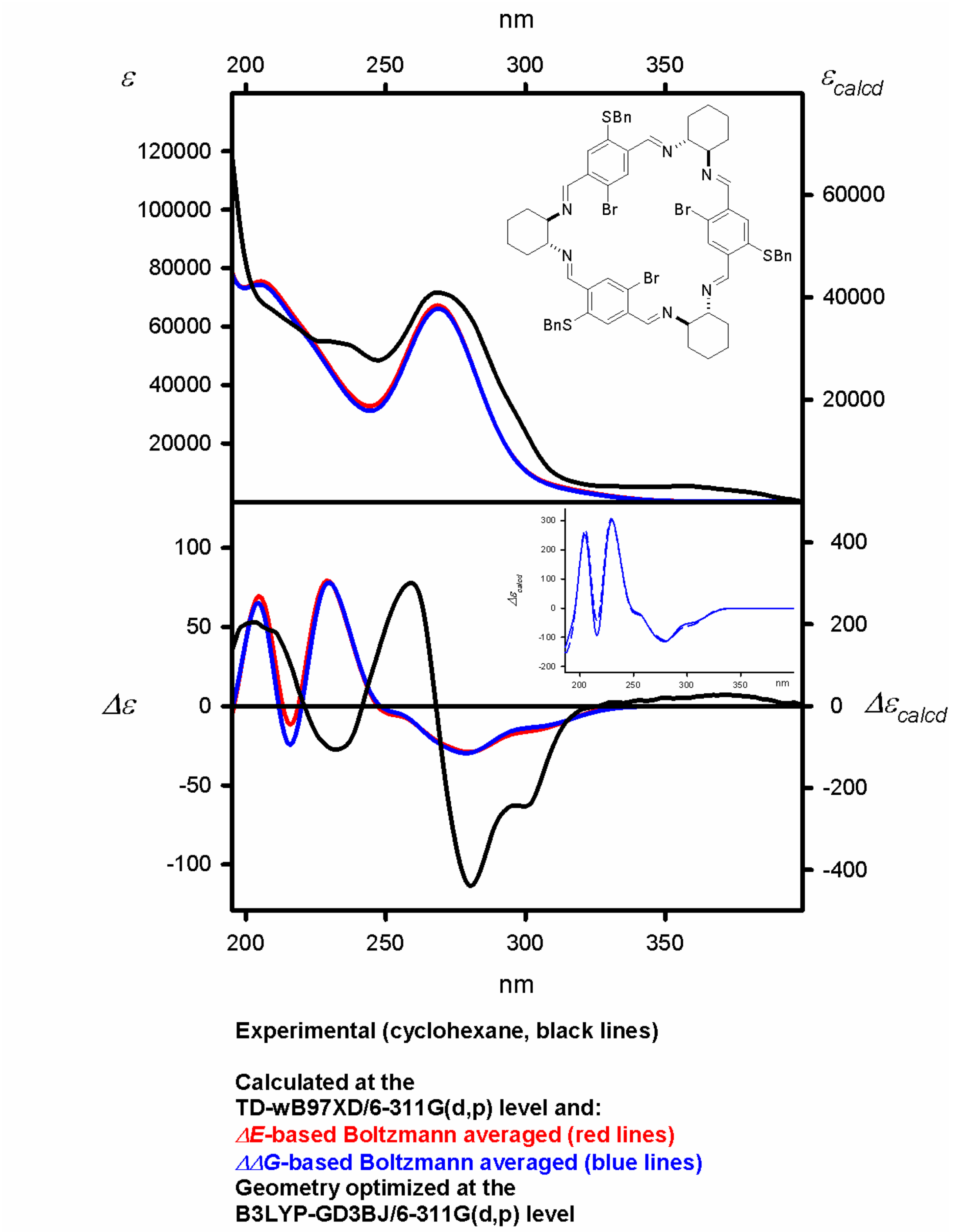

Figure S83. UV (upper panel) and ECD (lower panel) spectra of **6g** measured in cyclohexane (solid black lines) and calculated at the TD-wB97XD/6-311G(d,p) level for geometries optimized at the B3LYP-GD3BJ/6-311G(d,p) level. The calculated ECD spectra were Boltzmann-averaged based on *ΔE* (red lines) and *ΔΔG* values (blue lines). Wavelengths were corrected to match the experimental UV maxima. The insert shows the comparison between the ECD spectra calculated for the lowest energy conformer of a given compound (dashed blue lines) and the *ΔΔ*G-based and Boltzmann averaged (solid blue lines). *Δε* values are given in mol^{-1} cm^{-1} dm^{3}.

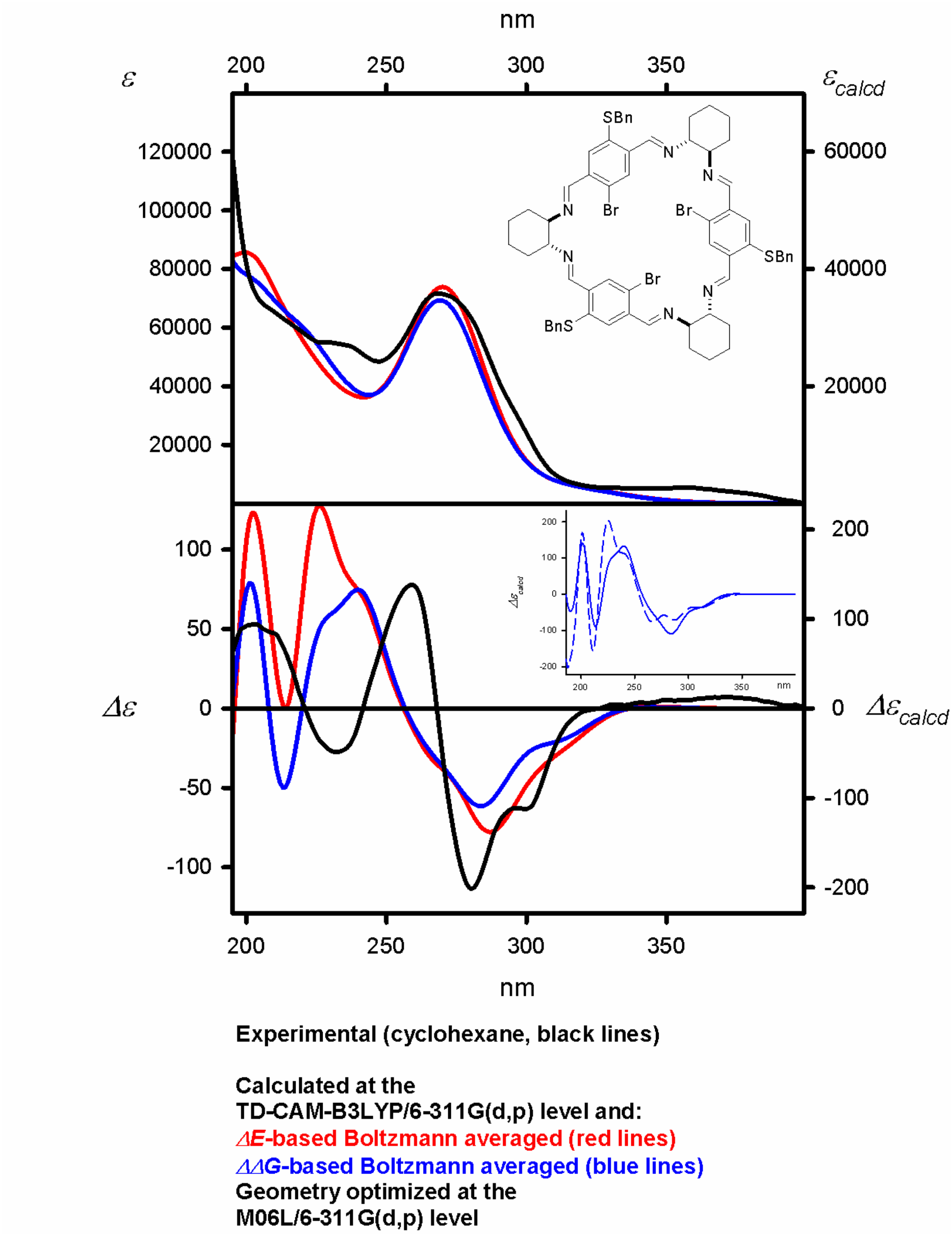

Figure S84. UV (upper panel) and ECD (lower panel) spectra of **6g** measured in cyclohexane (solid black lines) and calculated at the TD-CAM-B3LYP/6-311G(d,p) level for geometries optimized at the M06L/6-311G(d,p) level. The calculated ECD spectra were Boltzmann-averaged based on *ΔE* (red lines) and *ΔΔG* values (blue lines). Wavelengths were corrected to match the experimental UV maxima. The insert shows the comparison between the ECD spectra calculated for the lowest energy conformer of a given compound (dashed blue lines) and the *ΔΔ*G-based and Boltzmann averaged (solid blue lines). *Δε* values are given in mol^{-1} cm^{-1} dm^{3}.

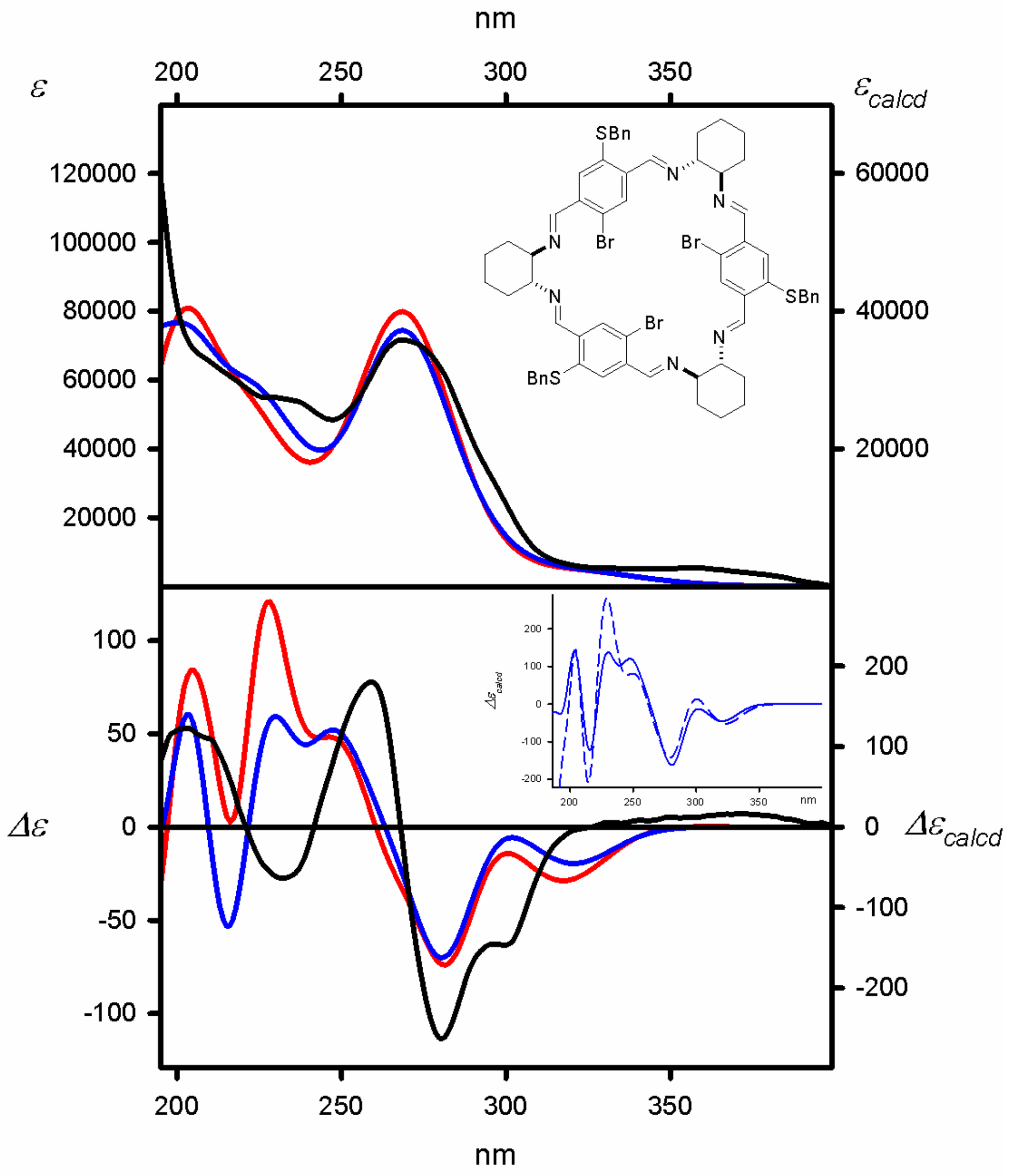

Experimental (cyclohexane, black lines)

Calculated at the
TD-M06-2X/6-311G(d,p) level and:
ΔE-based Boltzmann averaged (red lines)
$\Delta\Delta G$-based Boltzmann averaged (blue lines)
Geometry optimized at the
M06L/6-311G(d,p) level

Figure S85. UV (upper panel) and ECD (lower panel) spectra of **6g** measured in cyclohexane (solid black lines) and calculated at the TD-M06-2X/6-311G(d,p) level for geometries optimized at the M06L/6-311G(d,p) level. The calculated ECD spectra were Boltzmann-averaged based on ΔE (red lines) and $\Delta\Delta G$ values (blue lines). Wavelengths were corrected to match the experimental UV maxima. The insert shows the comparison between the ECD spectra calculated for the lowest energy conformer of a given compound (dashed blue lines) and the $\Delta\Delta G$-based and Boltzmann averaged (solid blue lines). $\Delta\varepsilon$ values are given in mol^{-1} cm^{-1} dm^{3}.

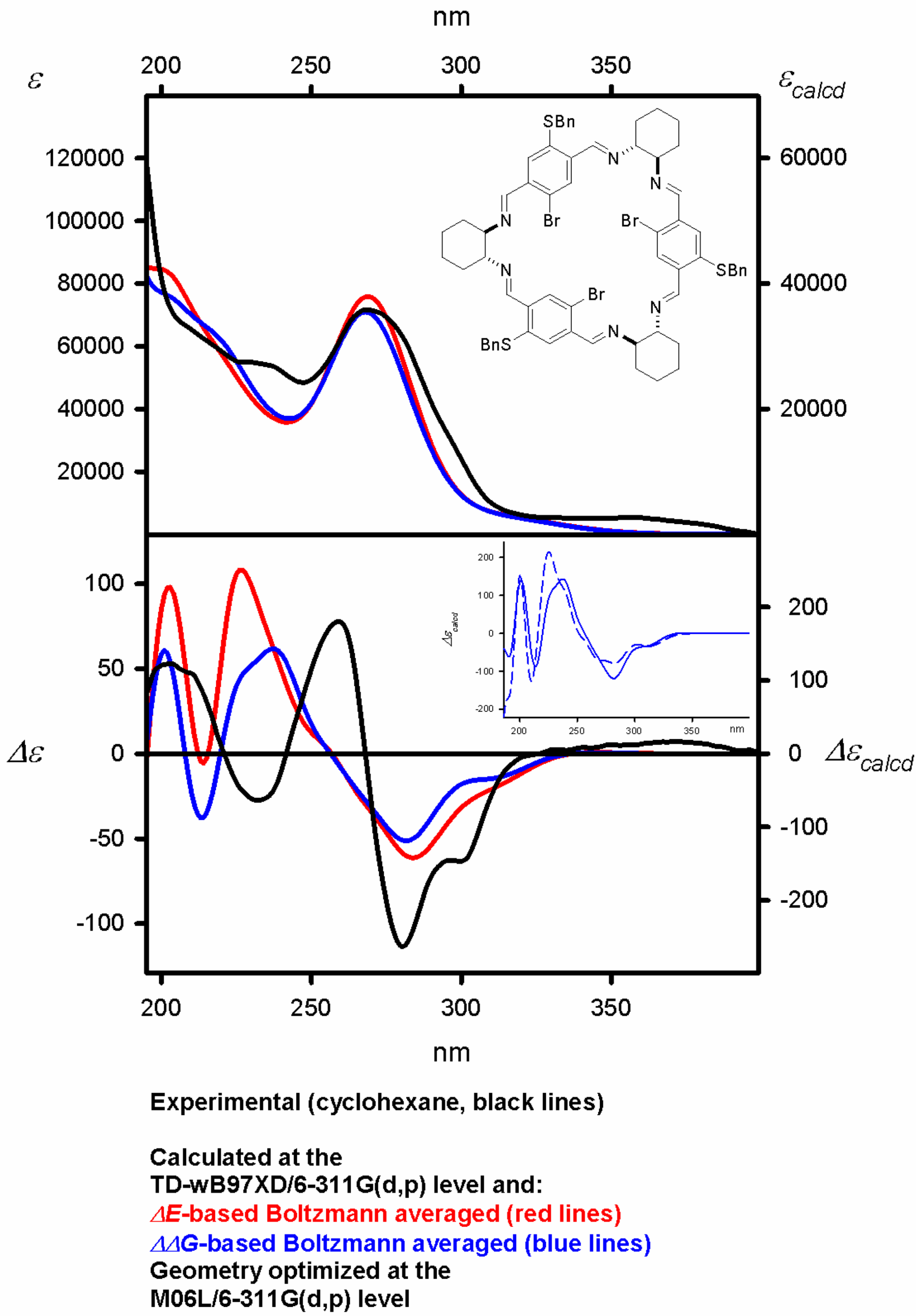

Figure S86. UV (upper panel) and ECD (lower panel) spectra of **6g** measured in cyclohexane (solid black lines) and calculated at the TD-wB97XD/6-311G(d,p) level for geometries optimized at the M06L/6-311G(d,p) level. The calculated ECD spectra were Boltzmann-averaged based on ΔE (red lines) and $\Delta\Delta G$ values (blue lines). Wavelengths were corrected to match the experimental UV maxima. The insert shows the comparison between the ECD spectra calculated for the lowest energy conformer of a given compound (dashed blue lines) and the $\Delta\Delta$G-based and Boltzmann averaged (solid blue lines). $\Delta\varepsilon$ values are given in mol^{-1} cm^{-1} dm^{3}.

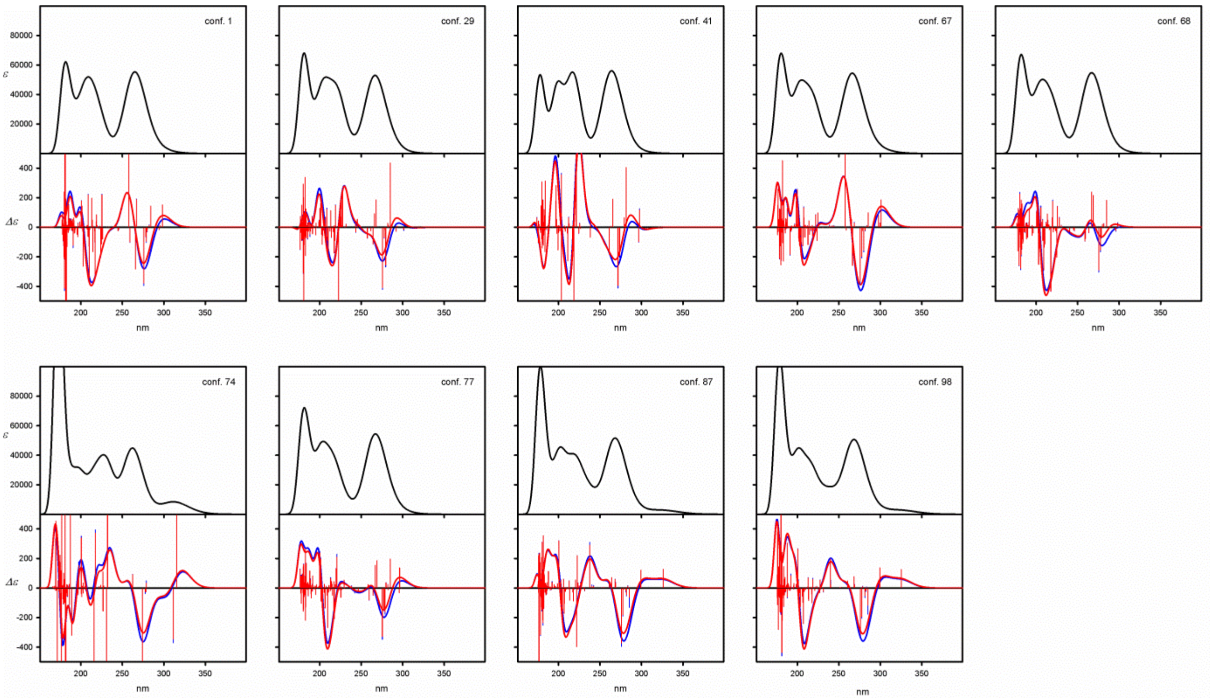

Figure S87. UV (upper panels) and ECD (lower panels) spectra calculated at the TD-CAM-B3LYP/6-311G(d,p) level for individual, symmetrical low-energy conformers of **6g**. Wavelengths were not corrected. Geometries were optimized at the B3LYP/6-311G(d,p) level. *Δε* values are given in mol^{-1} cm^{-1} dm^{3}.

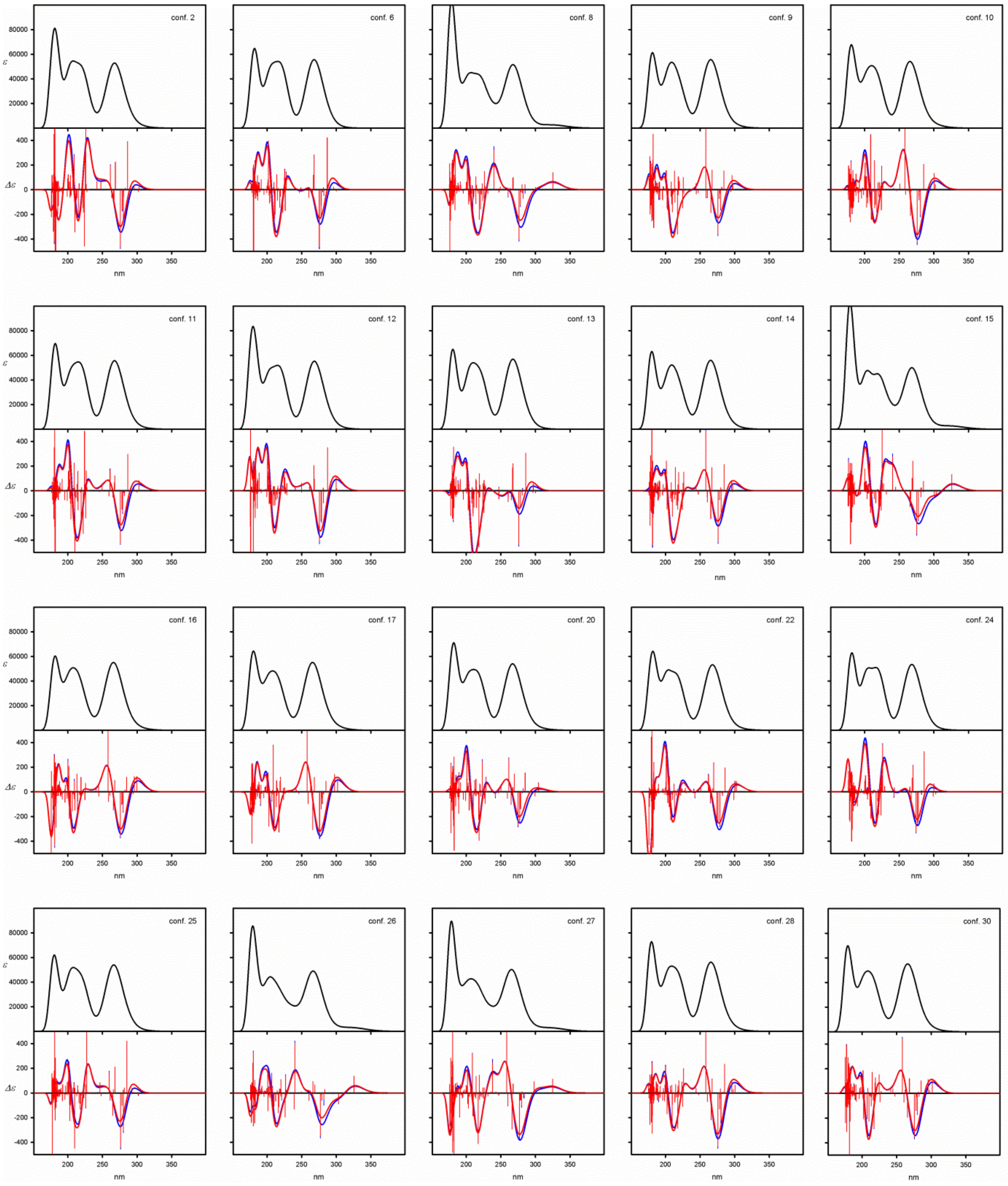
conf. 2
conf. 6
conf. 8
conf. 9
conf. 10
conf. 11
conf. 12
conf. 13
conf. 14
conf. 15
conf. 16
conf. 17
conf. 20
conf. 22
conf. 24
conf. 25
conf. 26
conf. 27
conf. 28
conf. 30
ε
Δε
nm

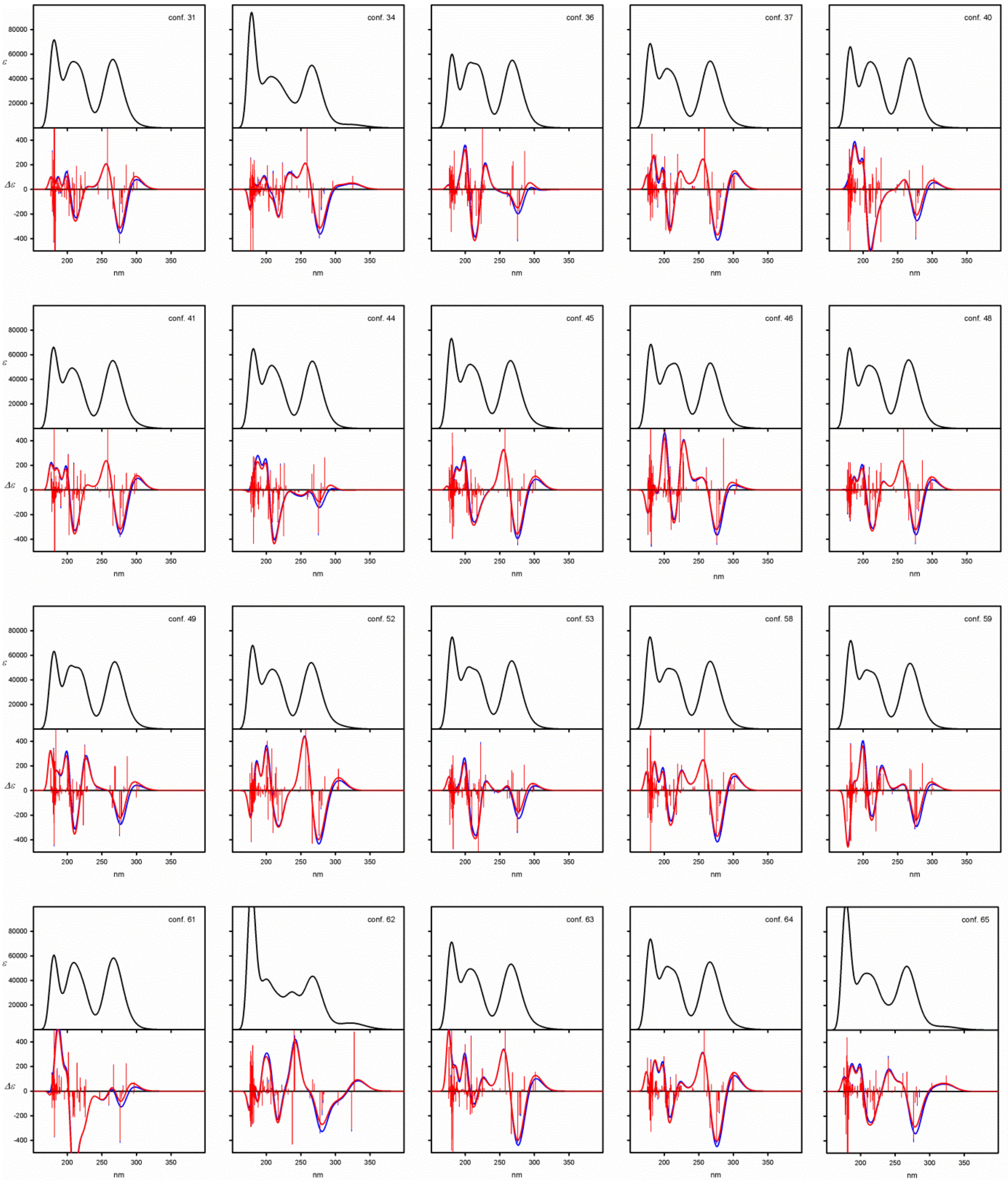
conf. 31
conf. 34
conf. 36
conf. 37
conf. 40
conf. 41
conf. 44
conf. 45
conf. 46
conf. 48
conf. 49
conf. 52
conf. 53
conf. 58
conf. 59
conf. 61
conf. 62
conf. 63
conf. 64
conf. 65
nm
ε
Δε

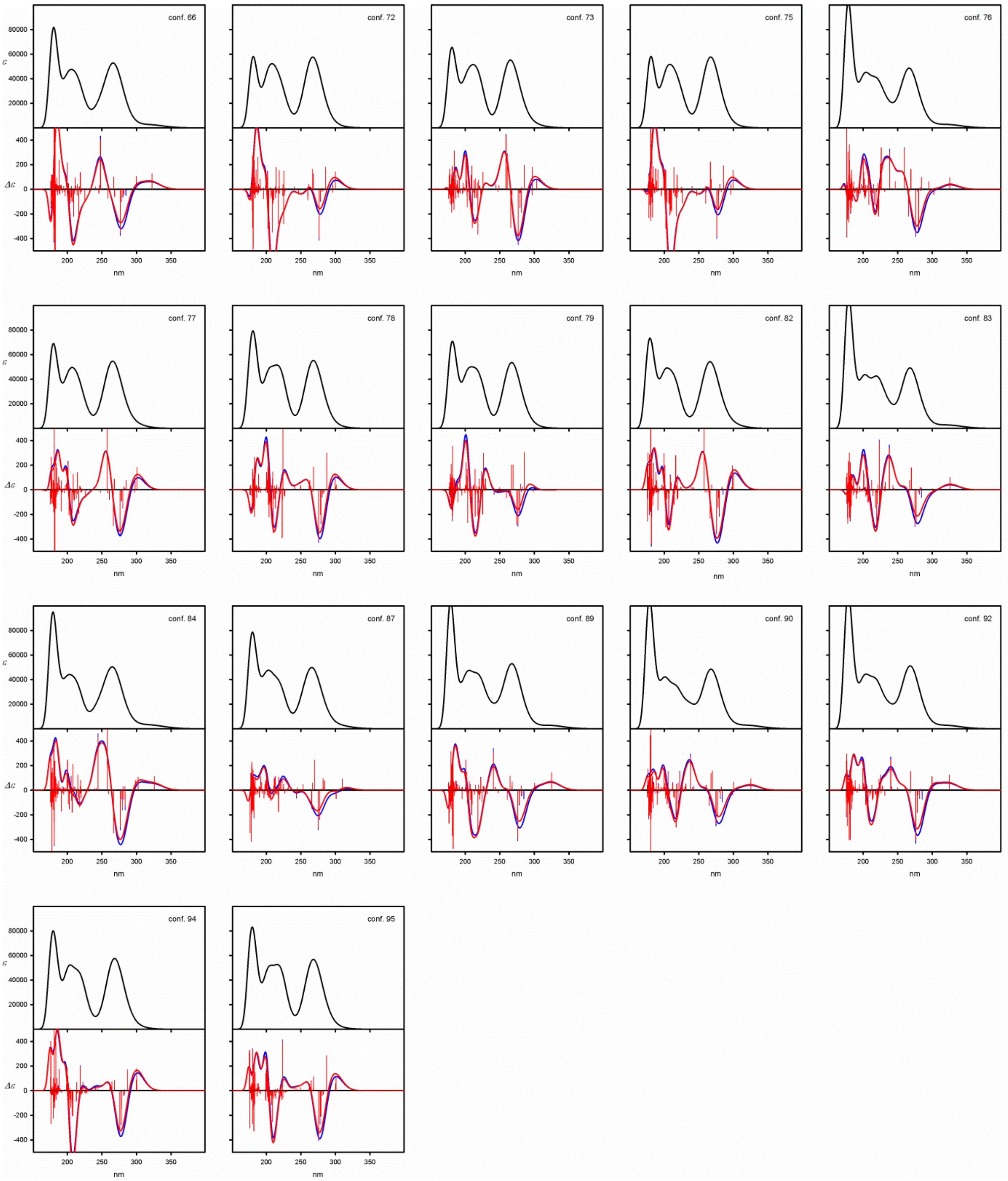

Figure S88. UV (upper panels) and ECD (lower panels) spectra calculated at the TD-CAM-B3LYP/6-311G(d,p) level for individual, non-symmetrical low-energy conformers of **6g**. Wavelengths were not corrected. Geometries were optimized at the B3LYP/6-311G(d,p) level. *Δε* values are given in mol^{-1} cm^{-1} dm^{3}.

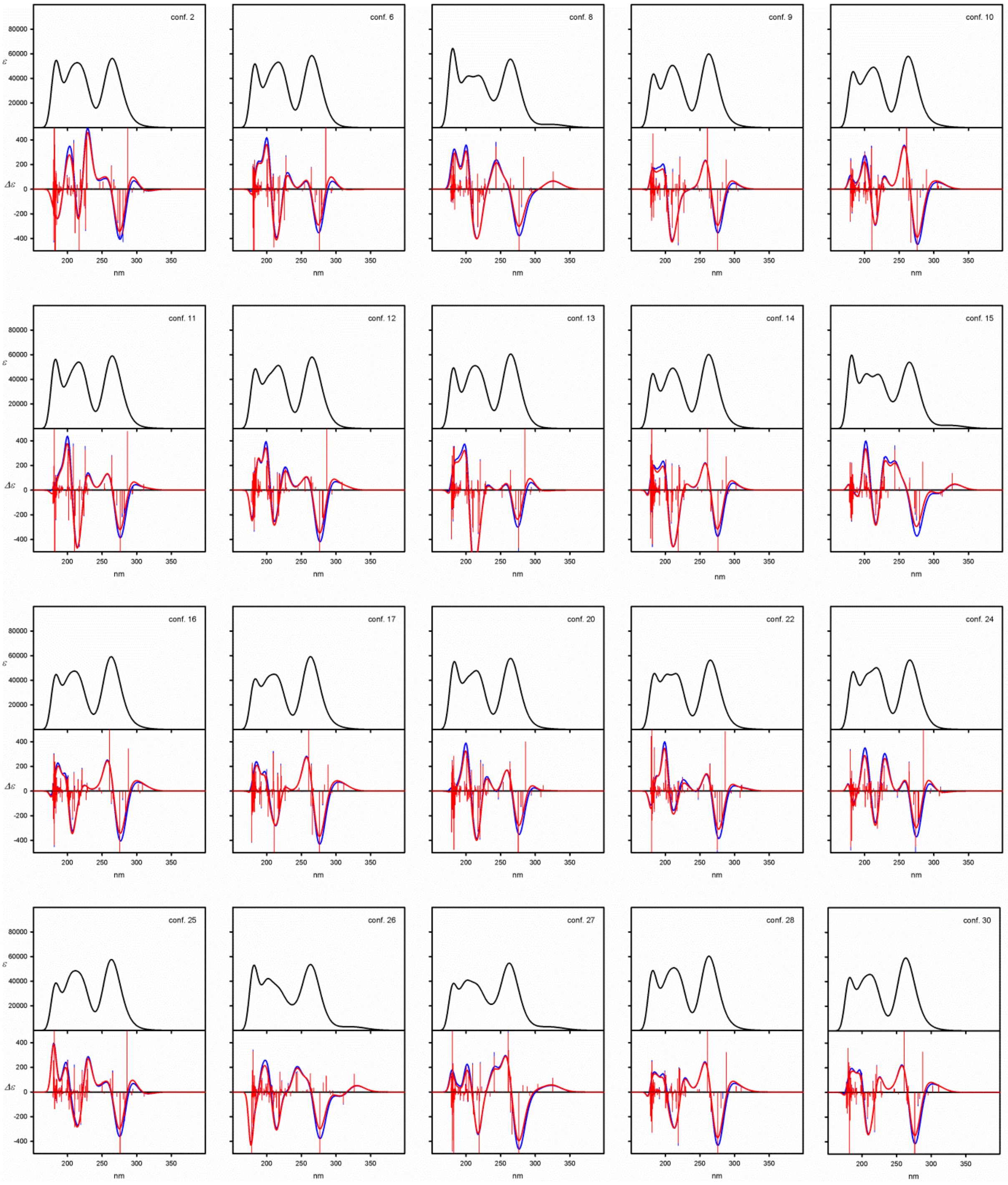
conf. 2
conf. 6
conf. 8
conf. 9
conf. 10
conf. 11
conf. 12
conf. 13
conf. 14
conf. 15
conf. 16
conf. 17
conf. 20
conf. 22
conf. 24
conf. 25
conf. 26
conf. 27
conf. 28
conf. 30
ε
Δε
nm

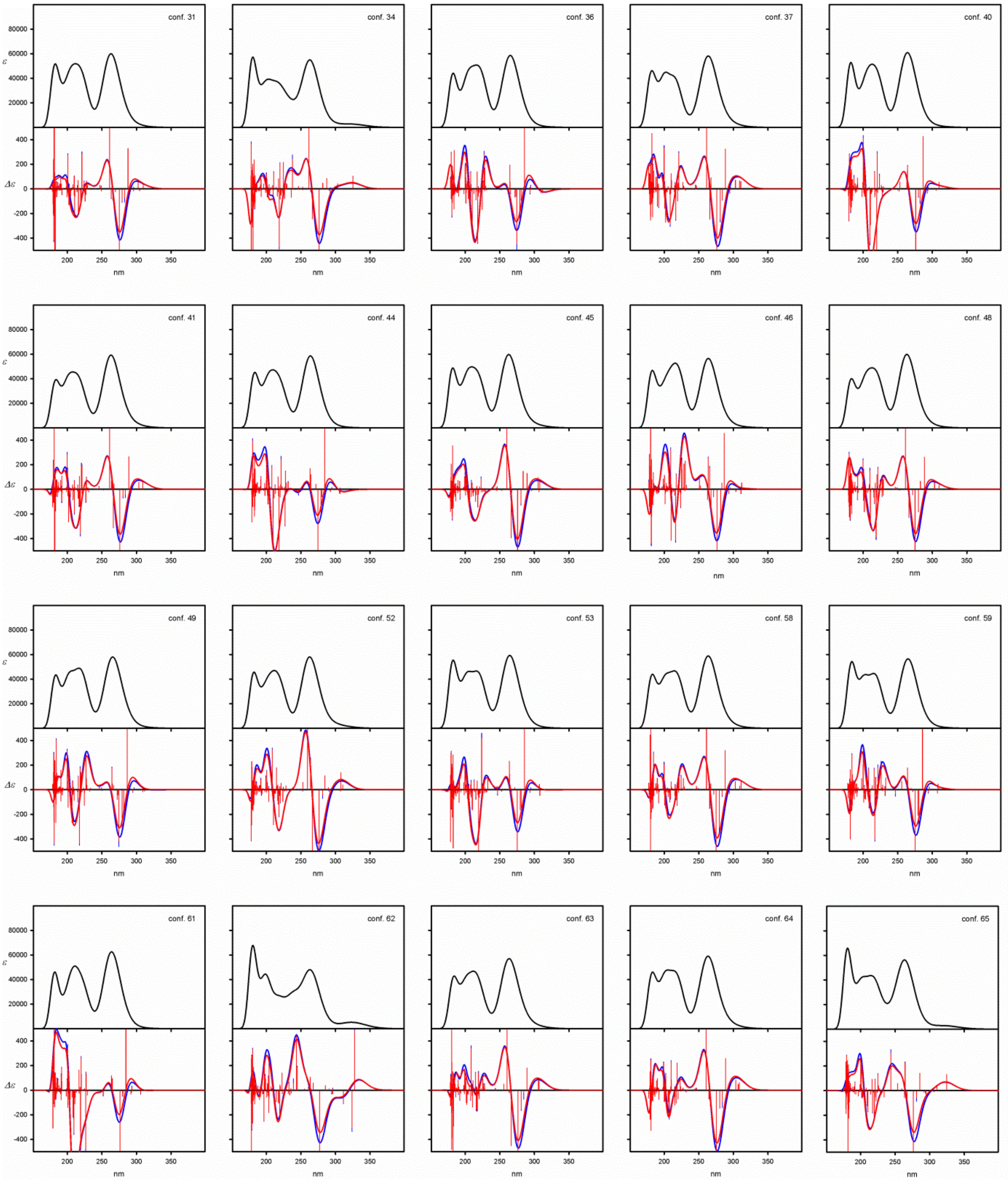
conf. 31
conf. 34
conf. 36
conf. 37
conf. 40
conf. 41
conf. 44
conf. 45
conf. 46
conf. 48
conf. 49
conf. 52
conf. 53
conf. 58
conf. 59
conf. 61
conf. 62
conf. 63
conf. 64
conf. 65
ε
Δε
nm

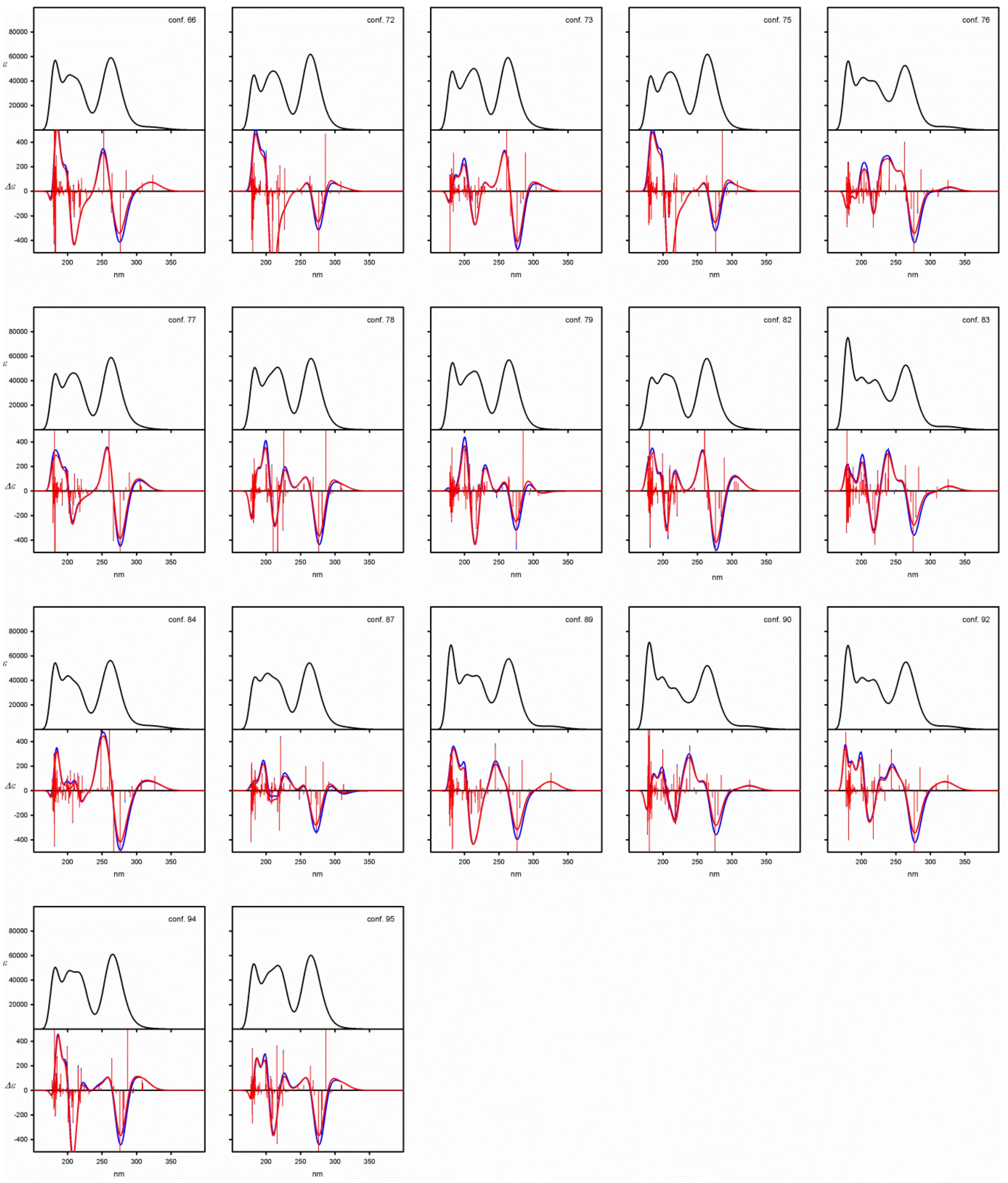

Figure S89. UV (upper panels) and ECD (lower panels) spectra calculated at the TD-M06-2X/6-311G(d,p) level for individual, symmetrical low-energy conformers of **6g**. Wavelengths were not corrected. Geometries were optimized at the B3LYP/6-311G(d,p) level. *Δε* values are given in mol^{-1} cm^{-1} dm^{3}.

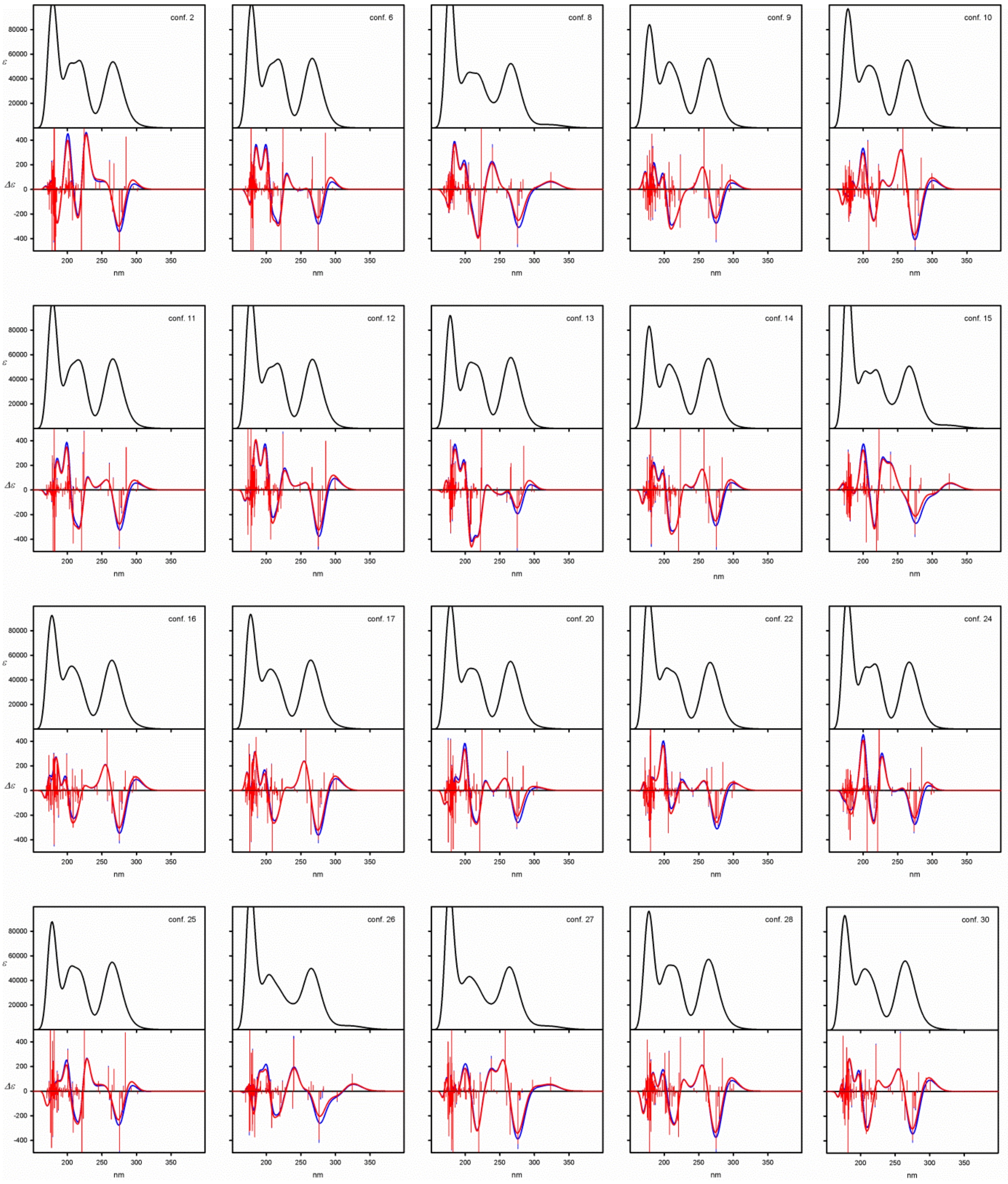
conf. 2
conf. 6
conf. 8
conf. 9
conf. 10
conf. 11
conf. 12
conf. 13
conf. 14
conf. 15
conf. 16
conf. 17
conf. 20
conf. 22
conf. 24
conf. 25
conf. 26
conf. 27
conf. 28
conf. 30
ε
80000
60000
40000
20000
Δε
400
200
0
-200
-400
200
250
300
350
nm

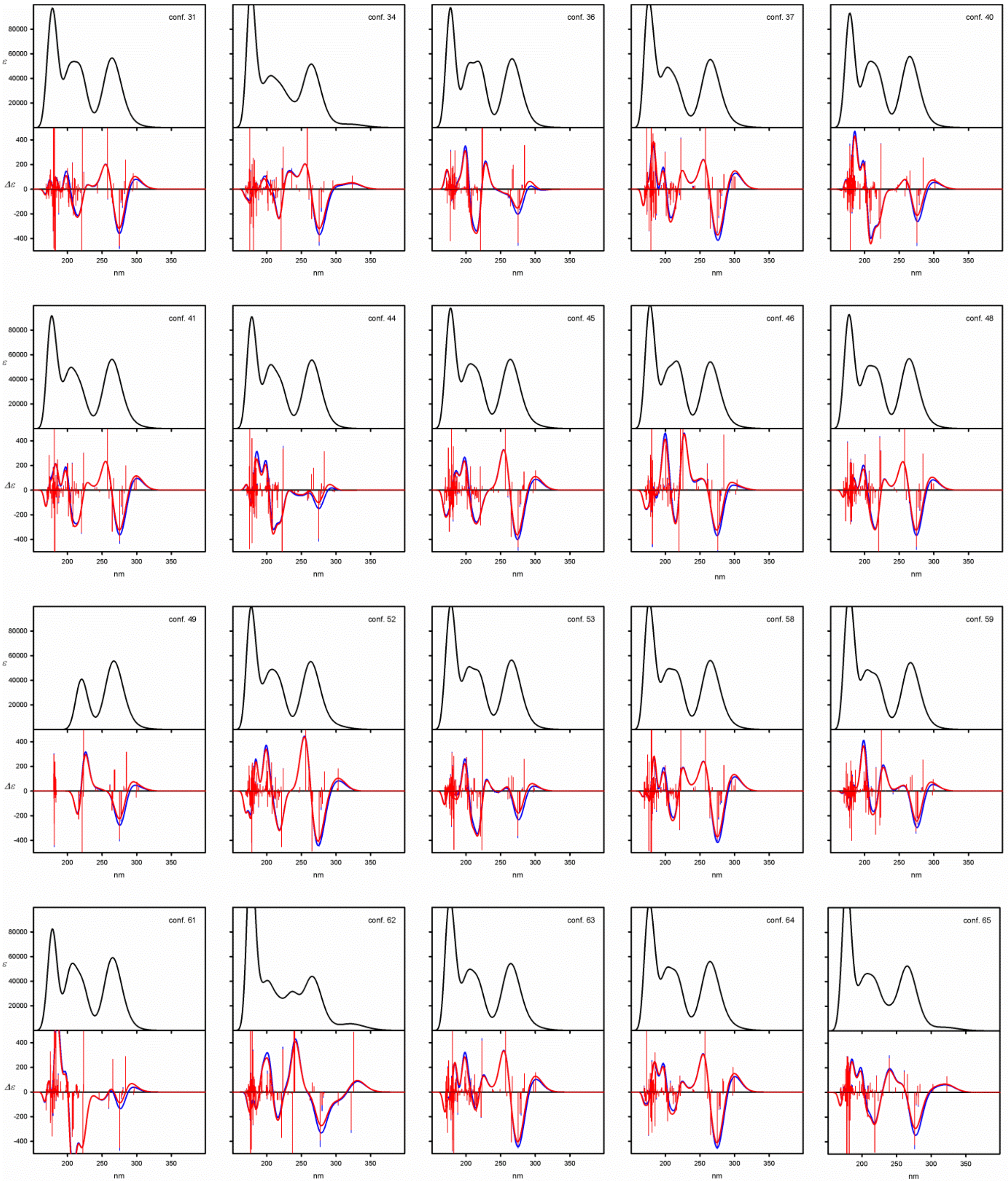
conf. 31
conf. 34
conf. 36
conf. 37
conf. 40
conf. 41
conf. 44
conf. 45
conf. 46
conf. 48
conf. 49
conf. 52
conf. 53
conf. 58
conf. 59
conf. 61
conf. 62
conf. 63
conf. 64
conf. 65
ε
Δε
nm

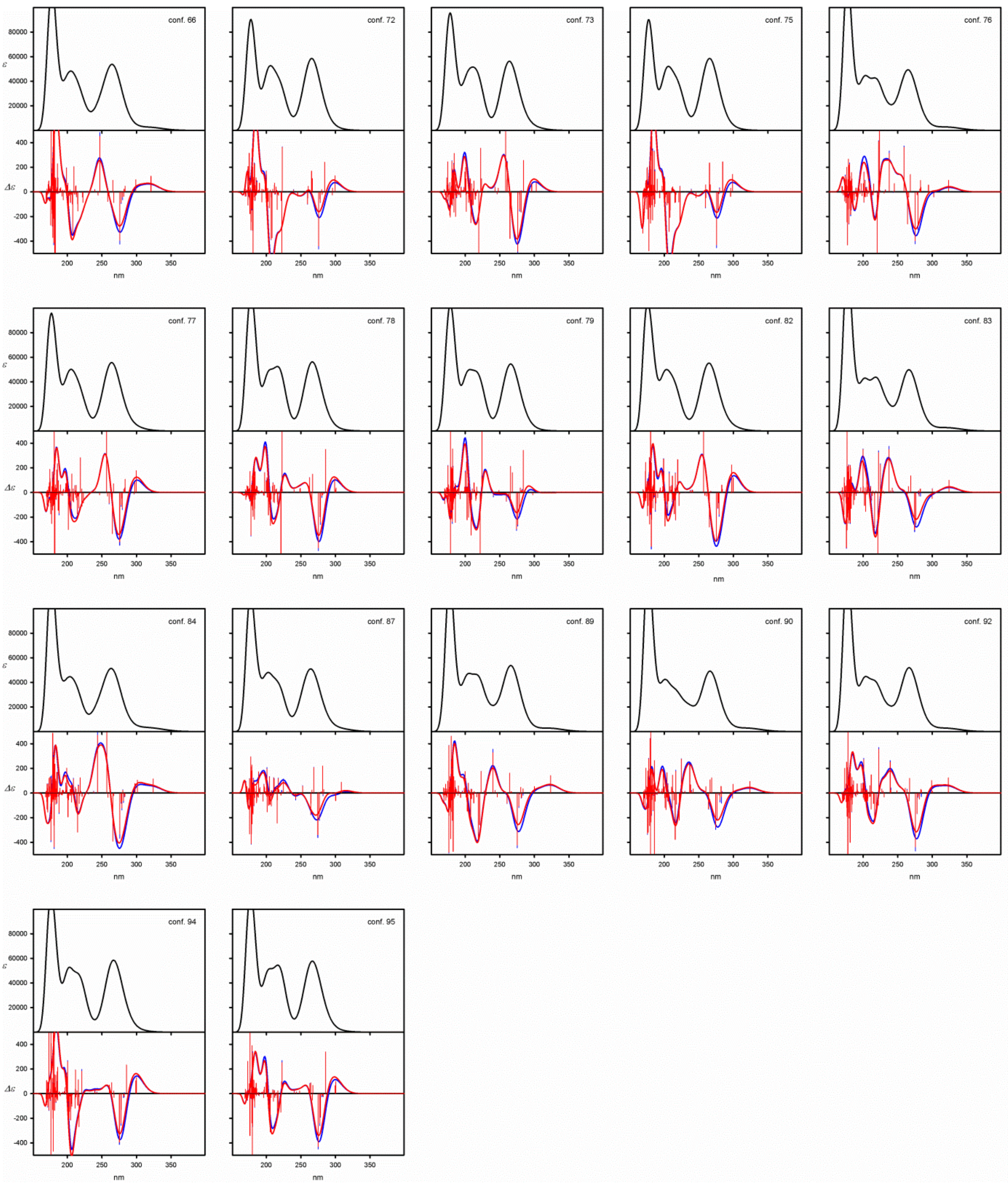

Figure S90. UV (upper panels) and ECD (lower panels) spectra calculated at the TD-M06-2X/6-311G(d,p) level for individual, non-symmetrical low-energy conformers of **6g**. Wavelengths were not corrected. Geometries were optimized at the B3LYP/6-311G(d,p) level. $\Delta\varepsilon$ values are given in mol^{-1} cm^{-1} dm^{3}.

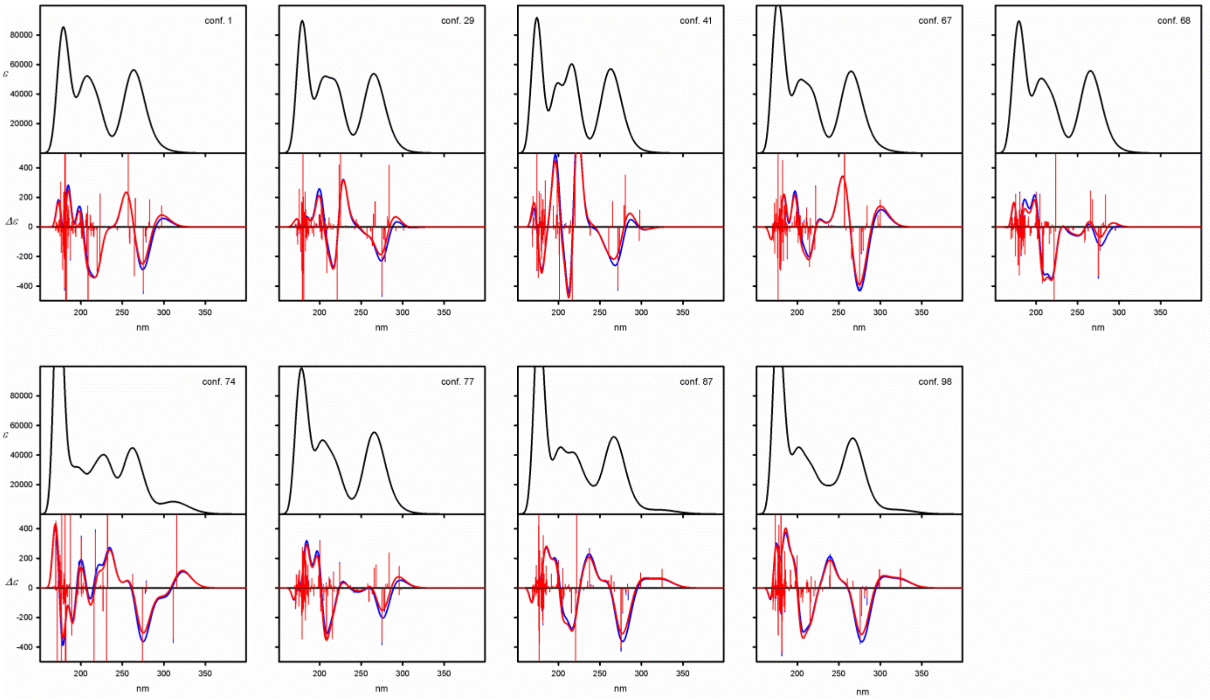

Figure S91. UV (upper panels) and ECD (lower panels) spectra calculated at the TD-wB97XD/6-311G(d,p) level for individual, symmetrical low-energy conformers of **6g**. Wavelengths were not corrected. Geometries were optimized at the B3LYP/6-311G(d,p) level. *Δε* values are given in mol^{-1} cm^{-1} dm^3.

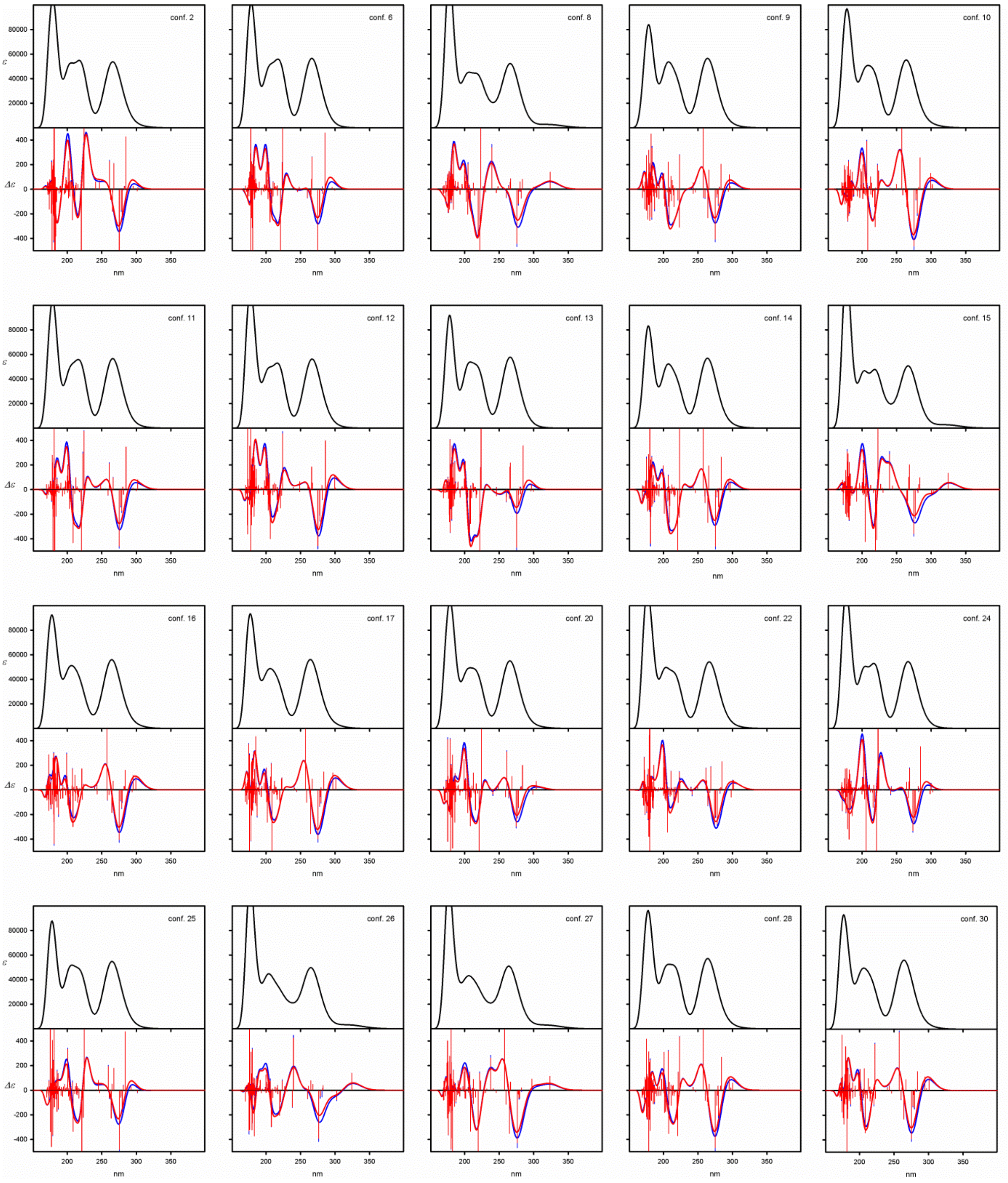
conf. 2
conf. 6
conf. 8
conf. 9
conf. 10
conf. 11
conf. 12
conf. 13
conf. 14
conf. 15
conf. 16
conf. 17
conf. 20
conf. 22
conf. 24
conf. 25
conf. 26
conf. 27
conf. 28
conf. 30
ε
80000
60000
40000
20000
Δε
400
200
0
-200
-400
200
250
300
350
nm

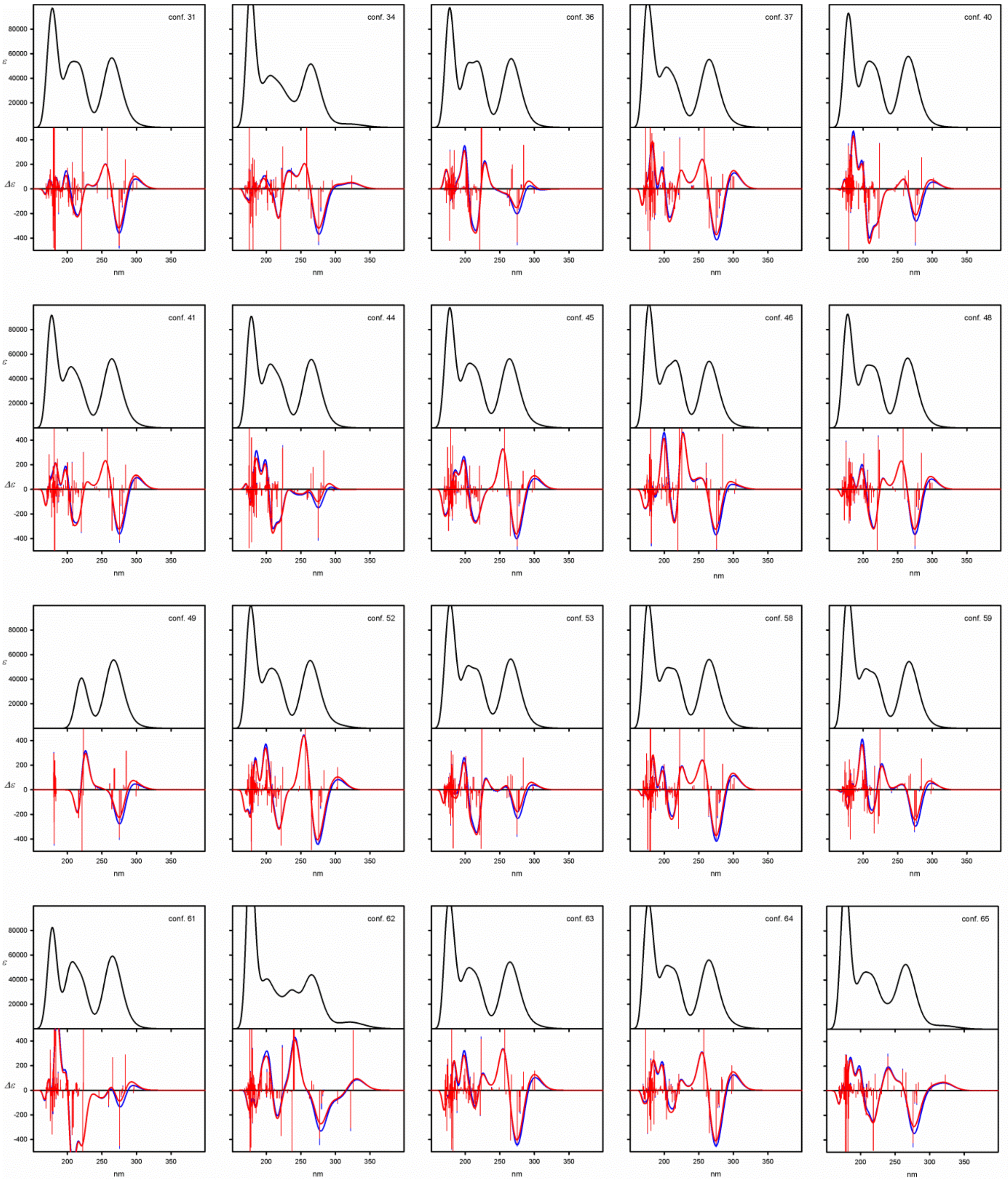
conf. 31
conf. 34
conf. 36
conf. 37
conf. 40
conf. 41
conf. 44
conf. 45
conf. 46
conf. 48
conf. 49
conf. 52
conf. 53
conf. 58
conf. 59
conf. 61
conf. 62
conf. 63
conf. 64
conf. 65
ε
Δε
nm
80000
60000
40000
20000
400
200
0
-200
-400
200
250
300
350

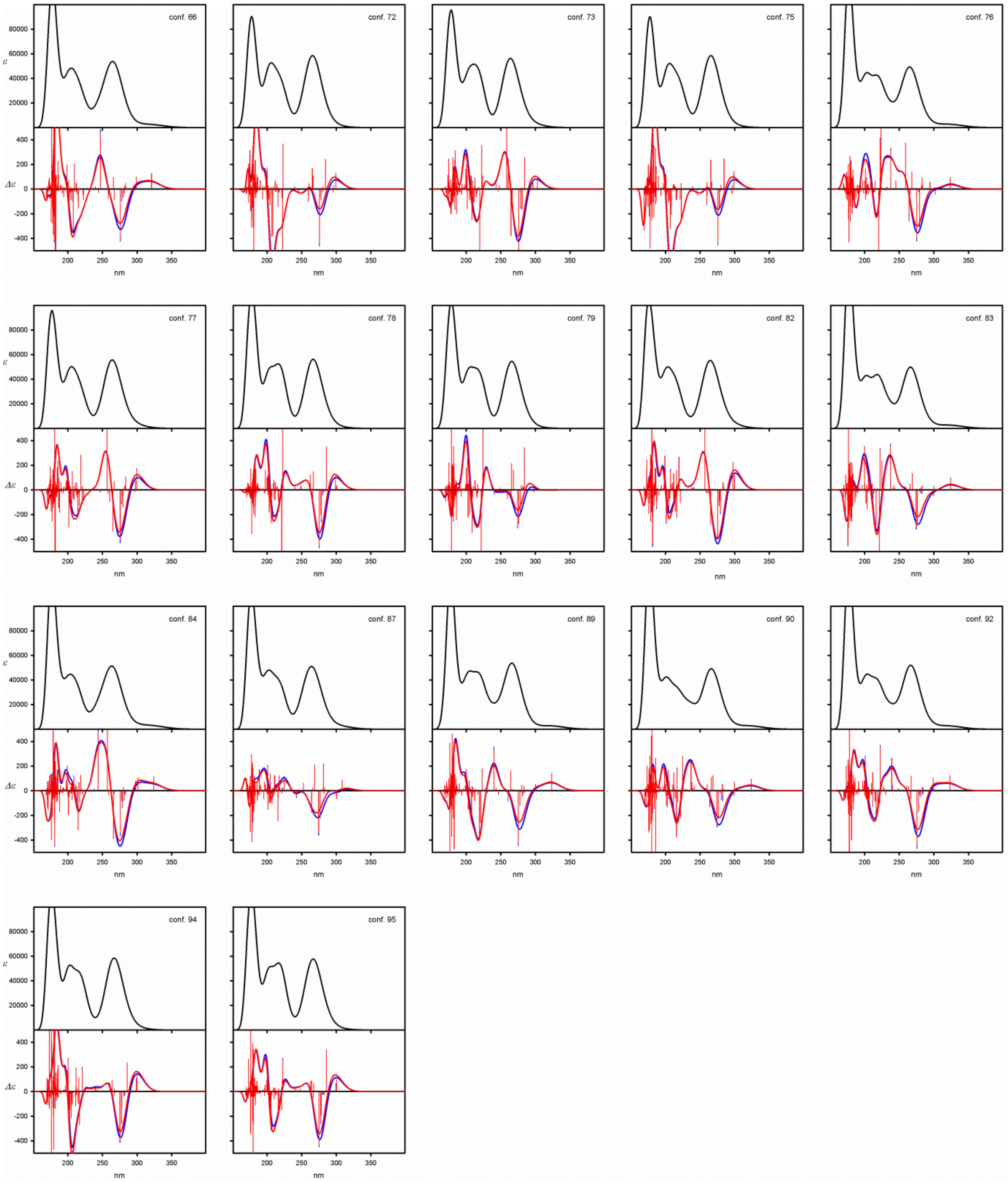

Figure S92. UV (upper panels) and ECD (lower panels) spectra calculated at the TD-wB97XD/6-311G(d,p) level for individual, non-symmetrical low-energy conformers of **6g**. Wavelengths were not corrected. Geometries were optimized at the B3LYP/6-311G(d,p) level. *Δε* values are given in mol^{-1} cm^{-1} dm^{3}.

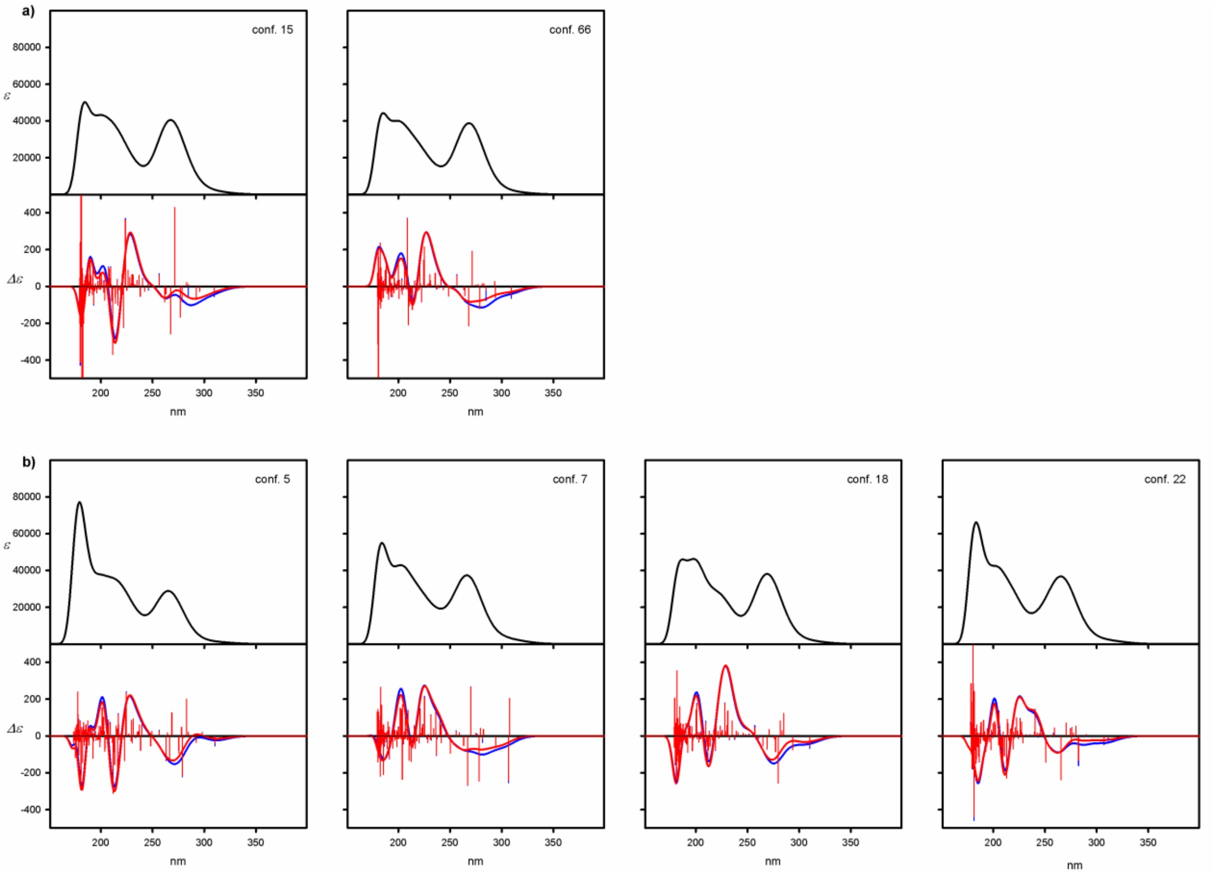

Figure S93. UV (upper panels) and ECD (lower panels) spectra calculated at the TD-CAM-B3LYP/6-311G(d,p) level for individual, a) symmetrical and b) non-symmetrical low-energy conformers of **6g**. Wavelengths were not corrected. Geometries were optimized at the B3LYP-GD3BJ/6-311G(d,p) level. $\Delta\varepsilon$ values are given in mol^{-1} cm^{-1} dm^{3}.

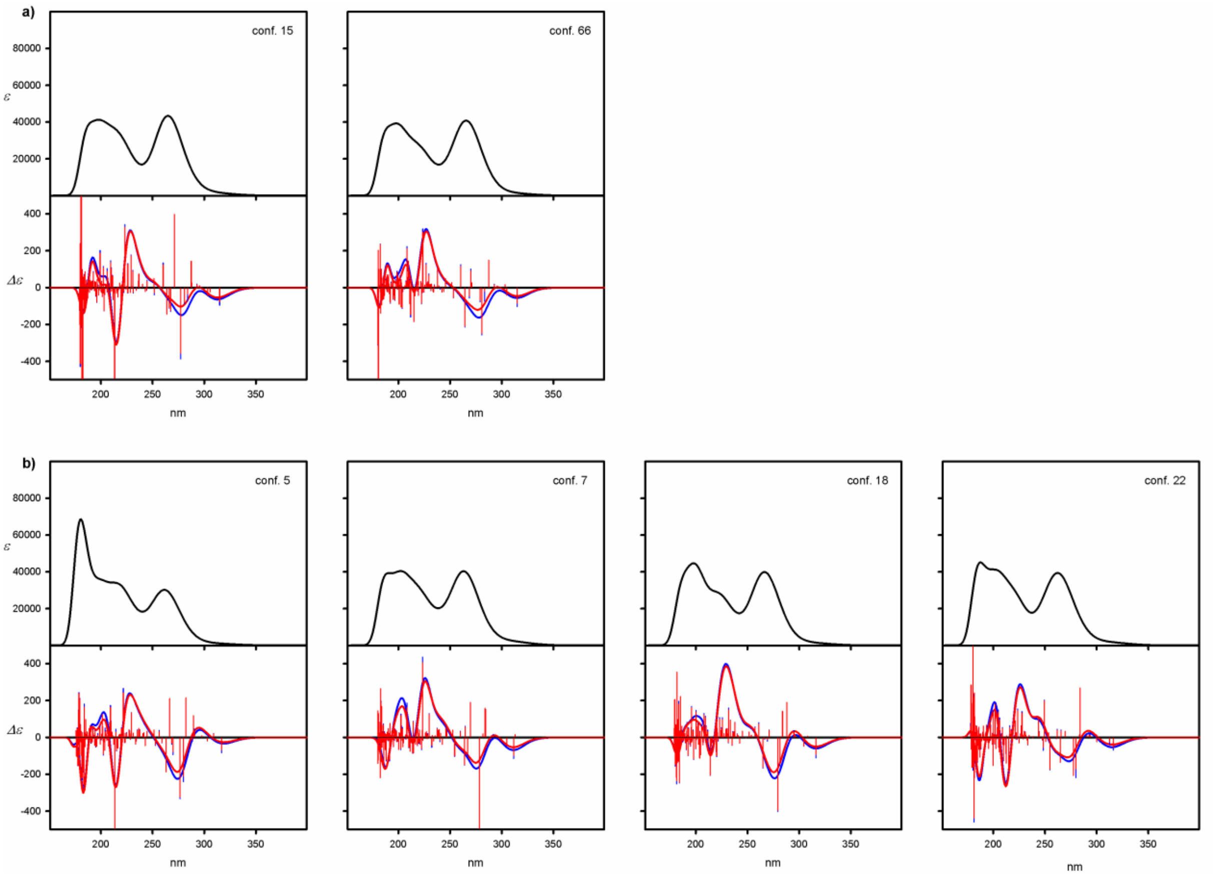

Figure S94. UV (upper panels) and ECD (lower panels) spectra calculated at the TD-M06-2X/6-311G(d,p) level for individual, a) symmetrical and b) non-symmetrical low-energy conformers of **6g**. Wavelengths were not corrected. Geometries were optimized at the B3LYP-GD3BJ/6-311G(d,p) level. *Δε* values are given in mol^{-1} cm^{-1} dm^3.

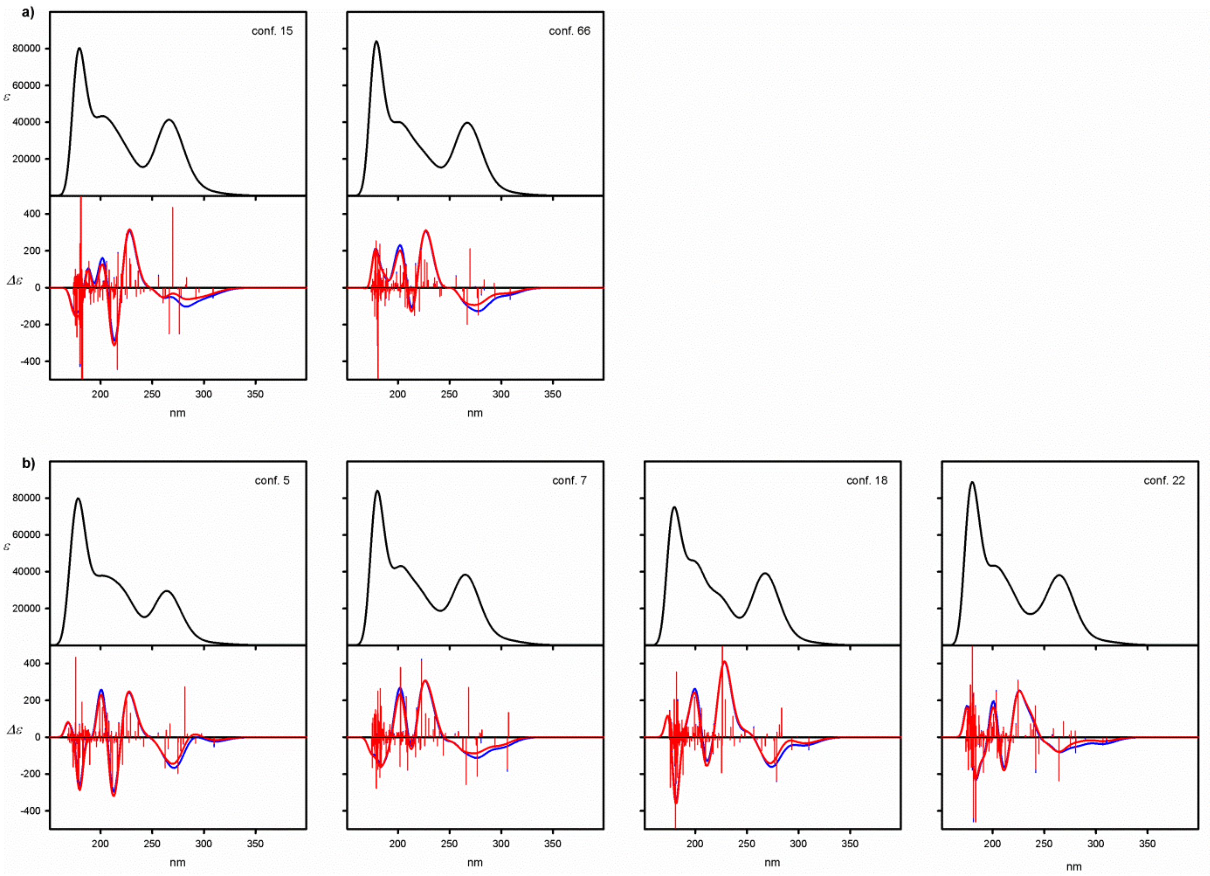

Figure S95. UV (upper panels) and ECD (lower panels) spectra calculated at the TD-wB97XD/6-311G(d,p) level for individual, a) symmetrical and b) non-symmetrical low-energy conformers of **6g**. Wavelengths were not corrected. Geometries were optimized at the B3LYP-GD3BJ/6-311G(d,p) level. $\Delta\varepsilon$ values are given in mol^{-1} cm^{-1} dm^{3}.

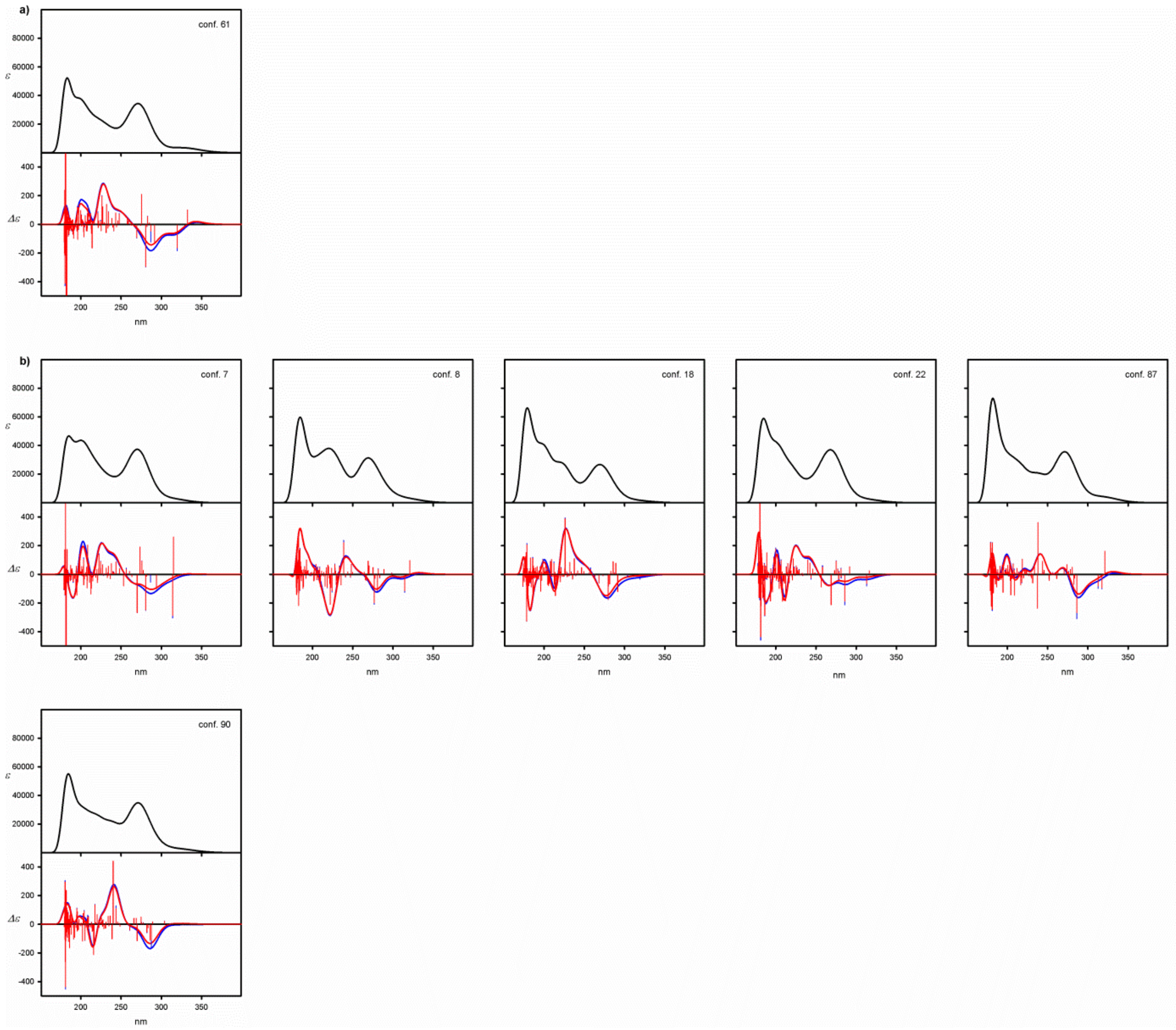

Figure S96. UV (upper panels) and ECD (lower panels) spectra calculated at the TD-CAM-B3LYP/6-311G(d,p) level for individual, a) symmetrical and b) non-symmetrical low-energy conformers of **6g**. Wavelengths were not corrected. Geometries were optimized at the M06L/6-311G(d,p) level. $\Delta\varepsilon$ values are given in mol^{-1} cm^{-1} dm^{3}.

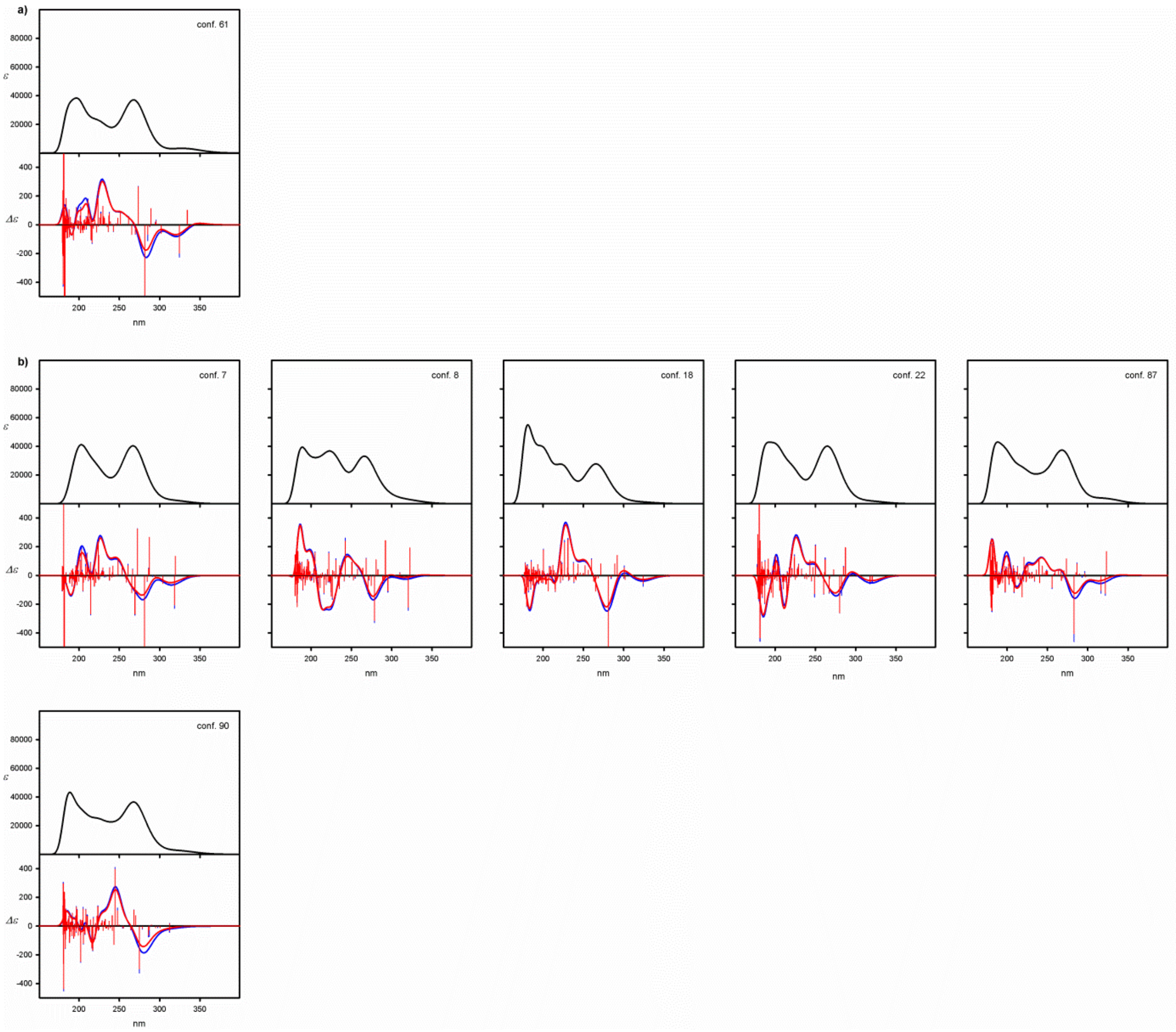

Figure S97. UV (upper panels) and ECD (lower panels) spectra calculated at the TD-M06-2X/6-311G(d,p) level for individual, a) symmetrical and b) non-symmetrical low-energy conformers of **6g**. Wavelengths were not corrected. Geometries were optimized at the M06L/6-311G(d,p) level. $\Delta\varepsilon$ values are given in mol^{-1} cm^{-1} dm^{3}.

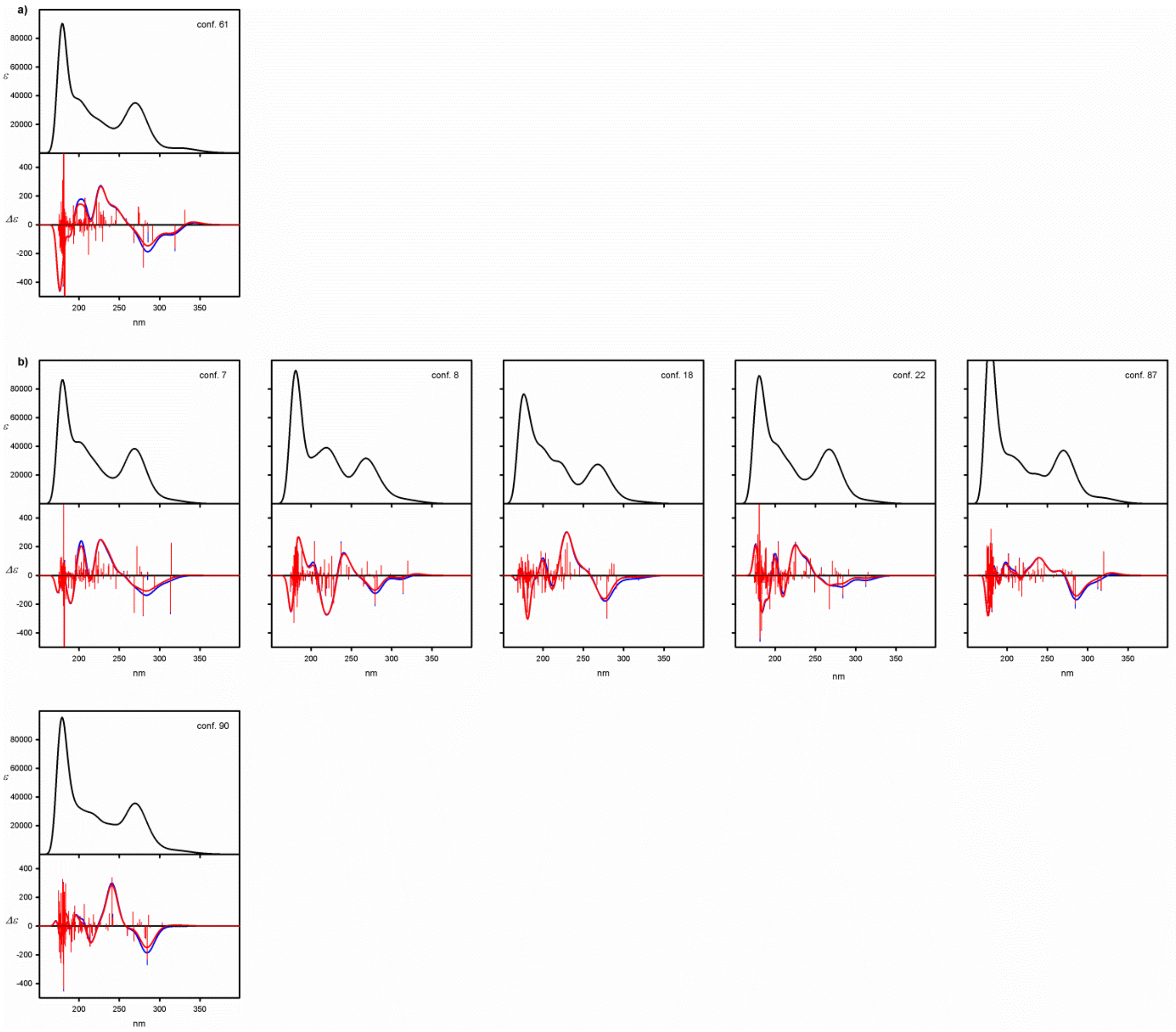

Figure S98. UV (upper panels) and ECD (lower panels) spectra calculated at the TD-wB97XD/6-311G(d,p) level for individual, a) symmetrical and b) non-symmetrical low-energy conformers of **6g**. Wavelengths were not corrected. Geometries were optimized at the M06L/6-311G(d,p) level. $\Delta\varepsilon$ values are given in mol^{-1} cm^{-1} dm^{3}.

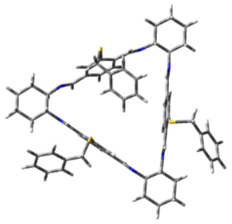

conf. sym-1

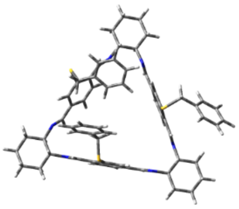

conf. sym-29

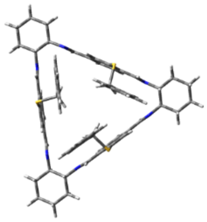

conf. sym-41

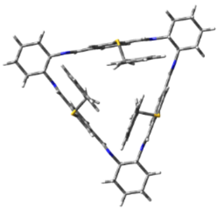

conf. sym-45

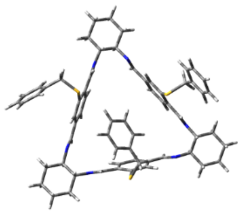

conf. sym-67

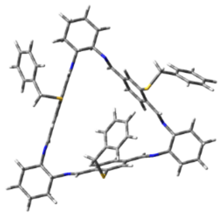

conf. sym-68

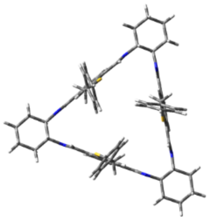

conf. sym-74

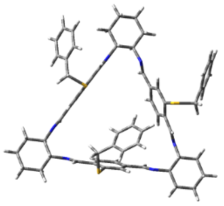

conf. sym-77

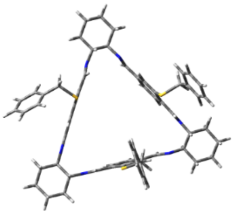

conf. sym-87

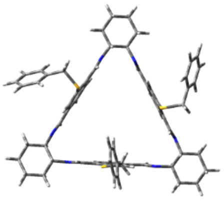

conf. sym-98

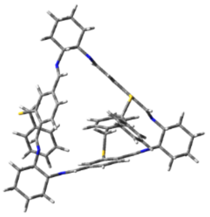

conf. 2

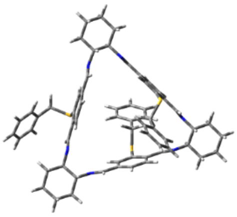

conf. 6

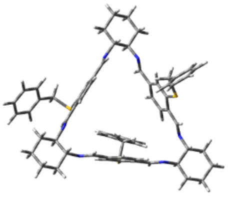

conf. 8

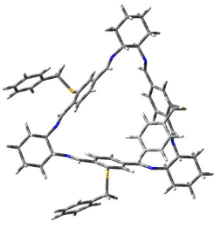

conf. 9

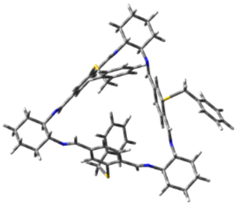

conf. 10

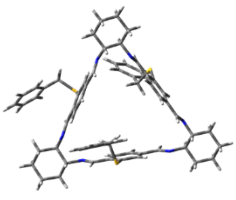

conf. 11

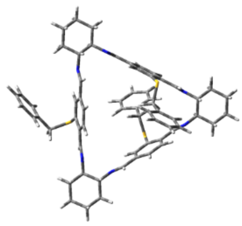

conf. 12

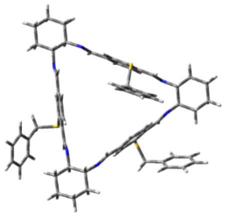

conf. 13

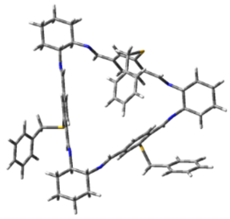

conf. 14

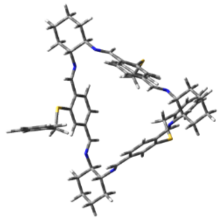

conf. 15

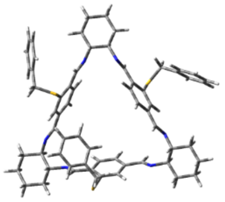

conf. 16

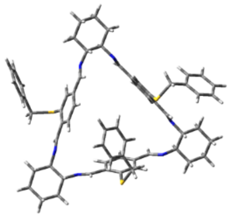

conf. 17

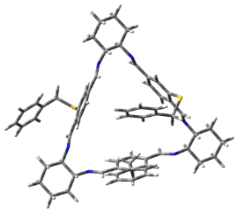

conf. 19

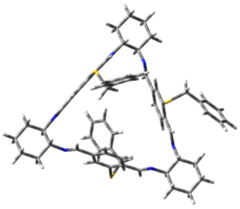

conf. 20

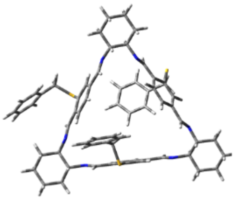

conf. 21

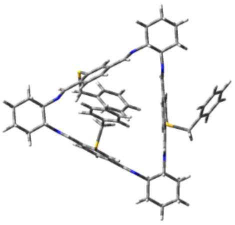

conf. 22

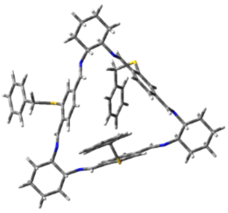

conf. 23

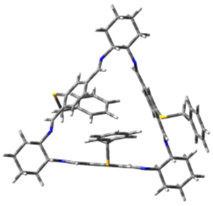

conf. 24

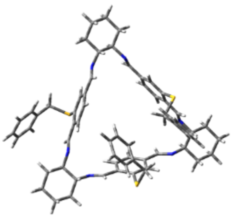

conf. 25

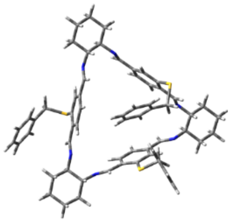

conf. 26

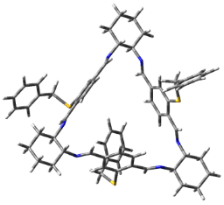

conf. 27

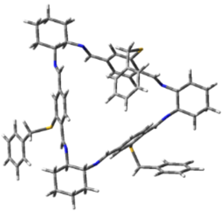

conf. 28

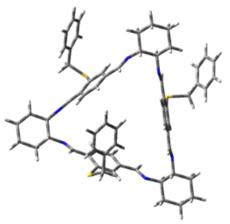

conf. 30

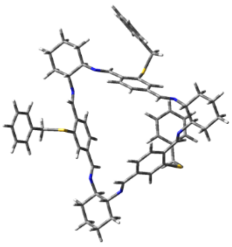

conf. 31

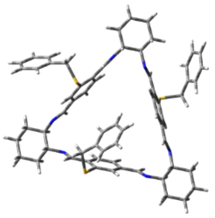

conf. 33

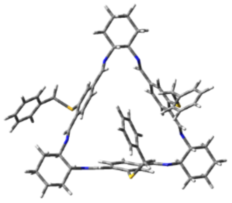

conf. 34

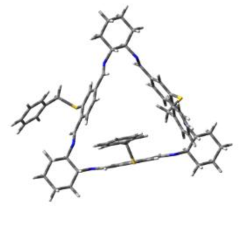
conf. 36

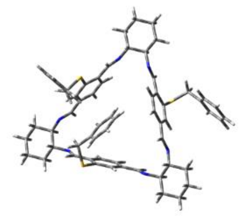
conf. 38

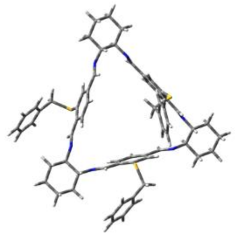
conf. 40

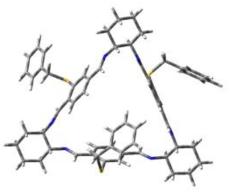
conf. 42

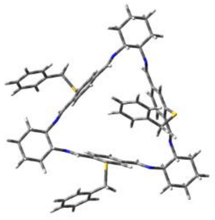
conf. 44

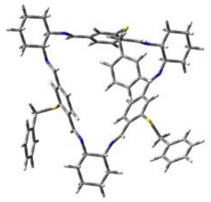
conf. 45

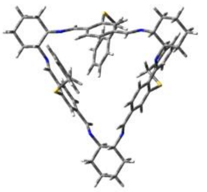
conf. 46

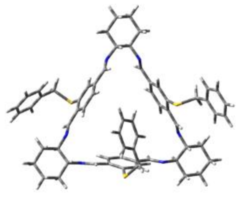
conf. 48

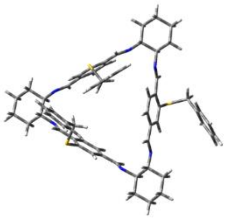
conf. 49

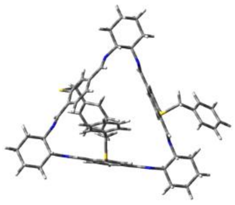
conf. 52

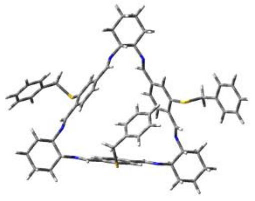
conf. 53

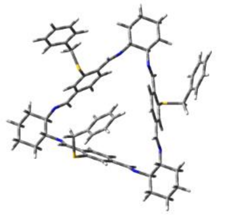
conf. 57

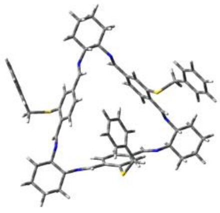
conf. 58

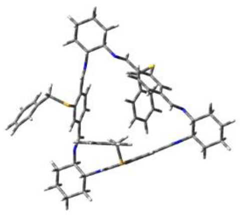
conf. 59

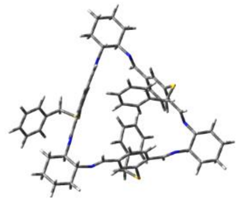
conf. 60

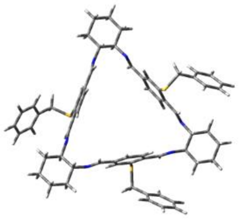
conf. 61

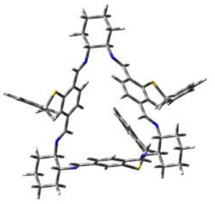
conf. 62

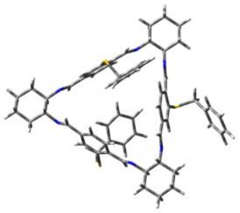
conf. 63

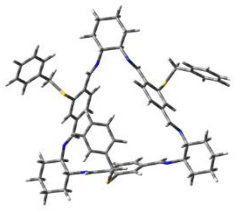
conf. 64

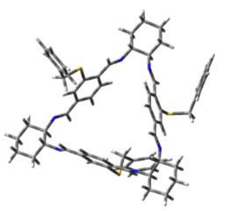
conf. 65

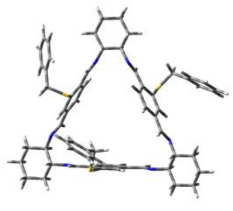
conf. 66

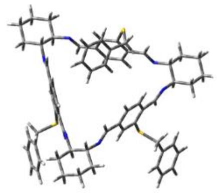
conf. 69

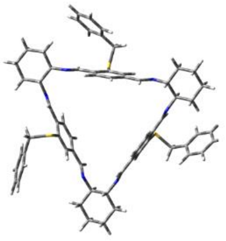
conf. 72

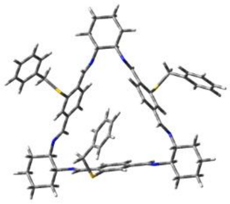
conf. 74

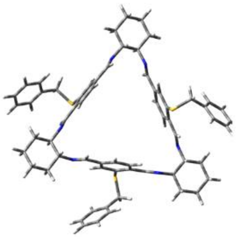
conf. 75

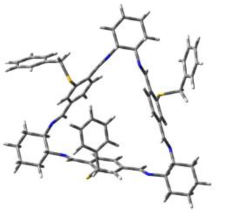
conf. 77

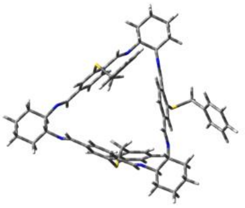
conf. 78

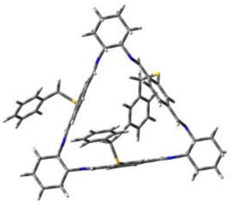
conf. 79

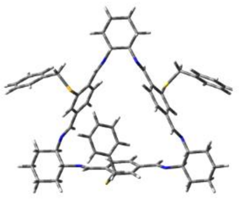
conf. 82

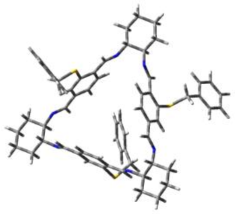
conf. 83

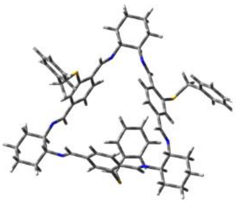
conf. 84

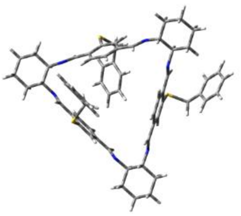
conf. 86

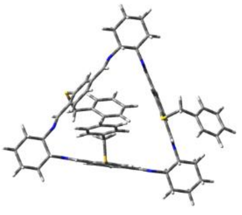
conf. 87

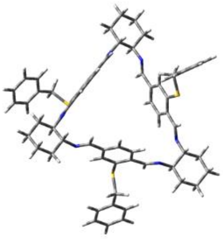
conf. 89

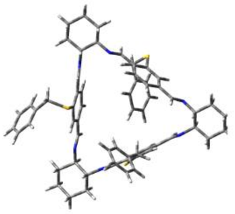
conf. 90

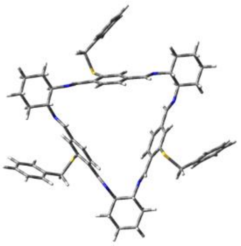
conf. 91

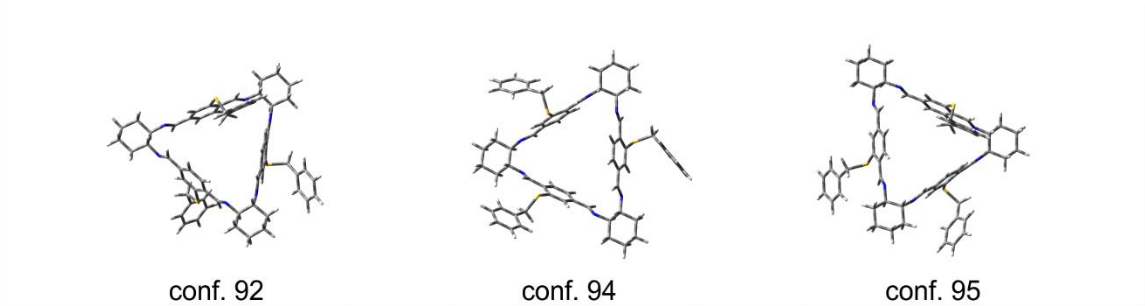

Figure S99. Structures of the low-energy conformers of **6h**, calculated at the B3LYP/6-311G(d,p) level. Prefix "sym" denotes symmetrical conformer.

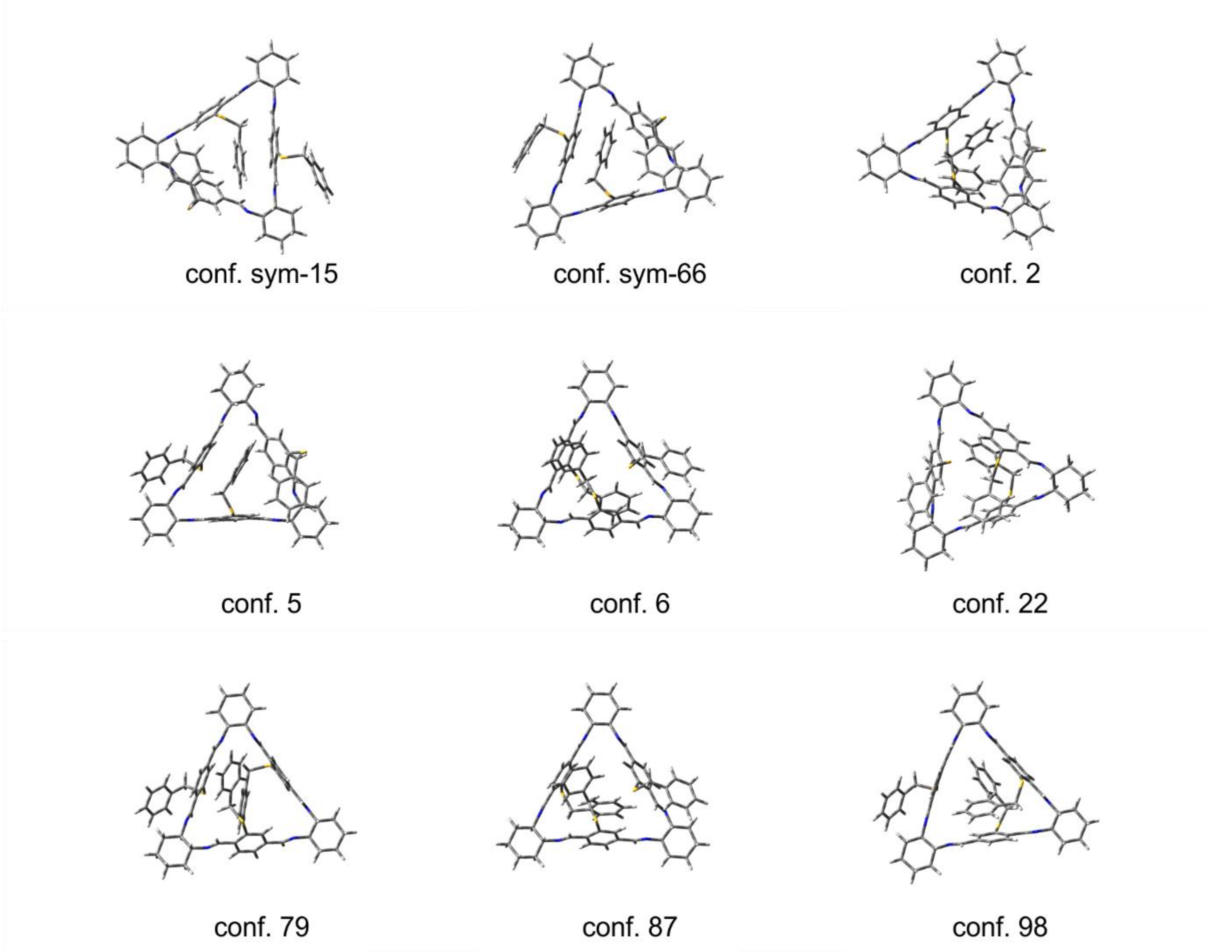

Figure S100. Structures of the low-energy conformers of **6h**, calculated at the B3LYP-GD3BJ/6-311G(d,p) level. Prefix "sym" denotes symmetrical conformer.

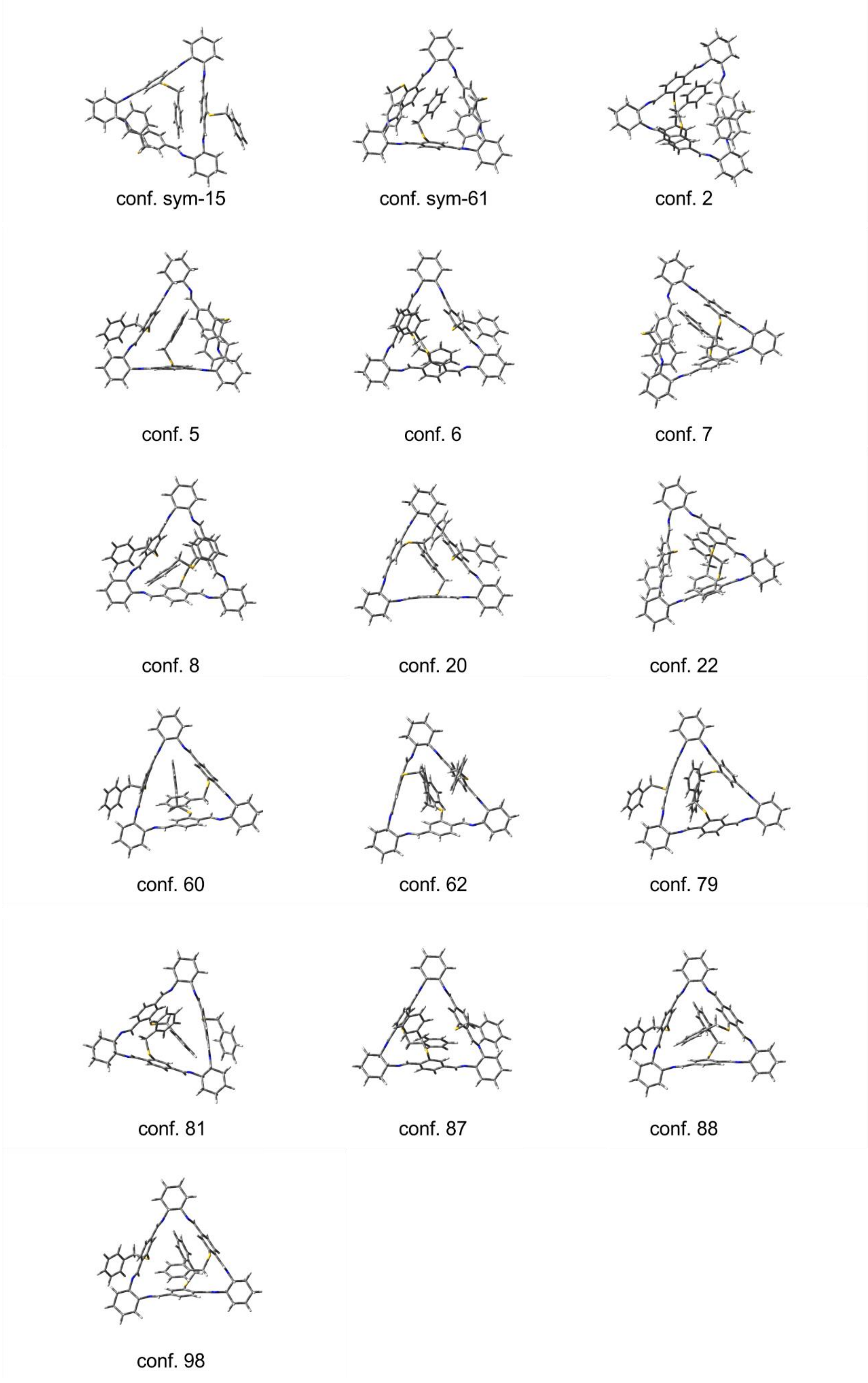

Figure S101. Structures of the low-energy conformers of **6h**, calculated at the M06L/6-311G(d,p) level. Prefix "sym" denotes symmetrical conformer.

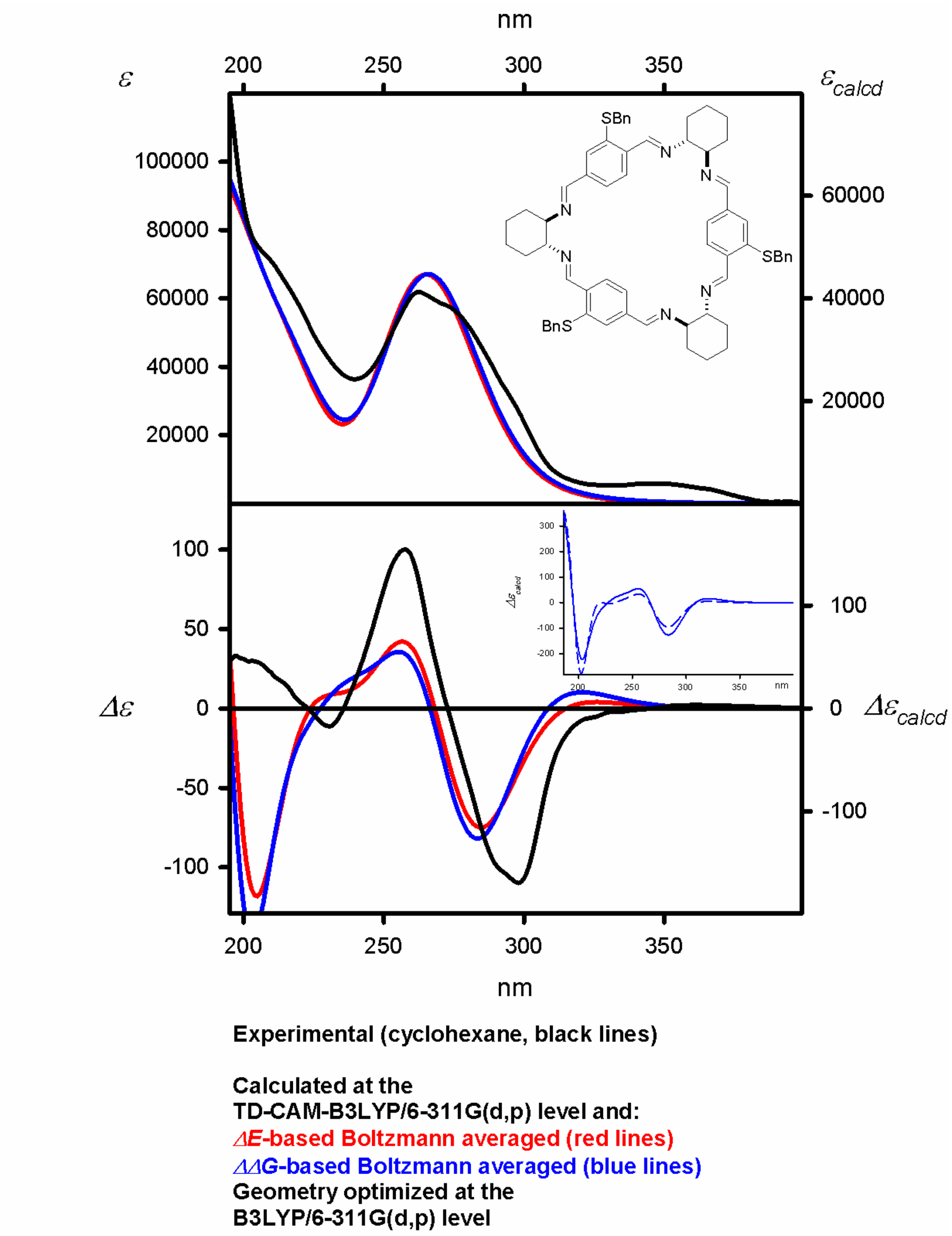

Figure S102. UV (upper panel) and ECD (lower panel) spectra of **6h** measured in cyclohexane (solid black lines) and calculated at the TD-CAM-B3LYP/6-311G(d,p) level for geometries optimized at the B3LYP/6-311G(d,p) level. The calculated ECD spectra were Boltzmann-averaged based on *ΔE* (red lines) and *ΔΔG* values (blue lines). Wavelengths were corrected to match the experimental UV maxima. The insert shows the comparison between the ECD spectra calculated for the lowest energy conformer of a given compound (dashed blue lines) and the *ΔΔ*G-based and Boltzmann averaged (solid blue lines). *Δε* values are given in mol^{-1} cm^{-1} dm^{3}.

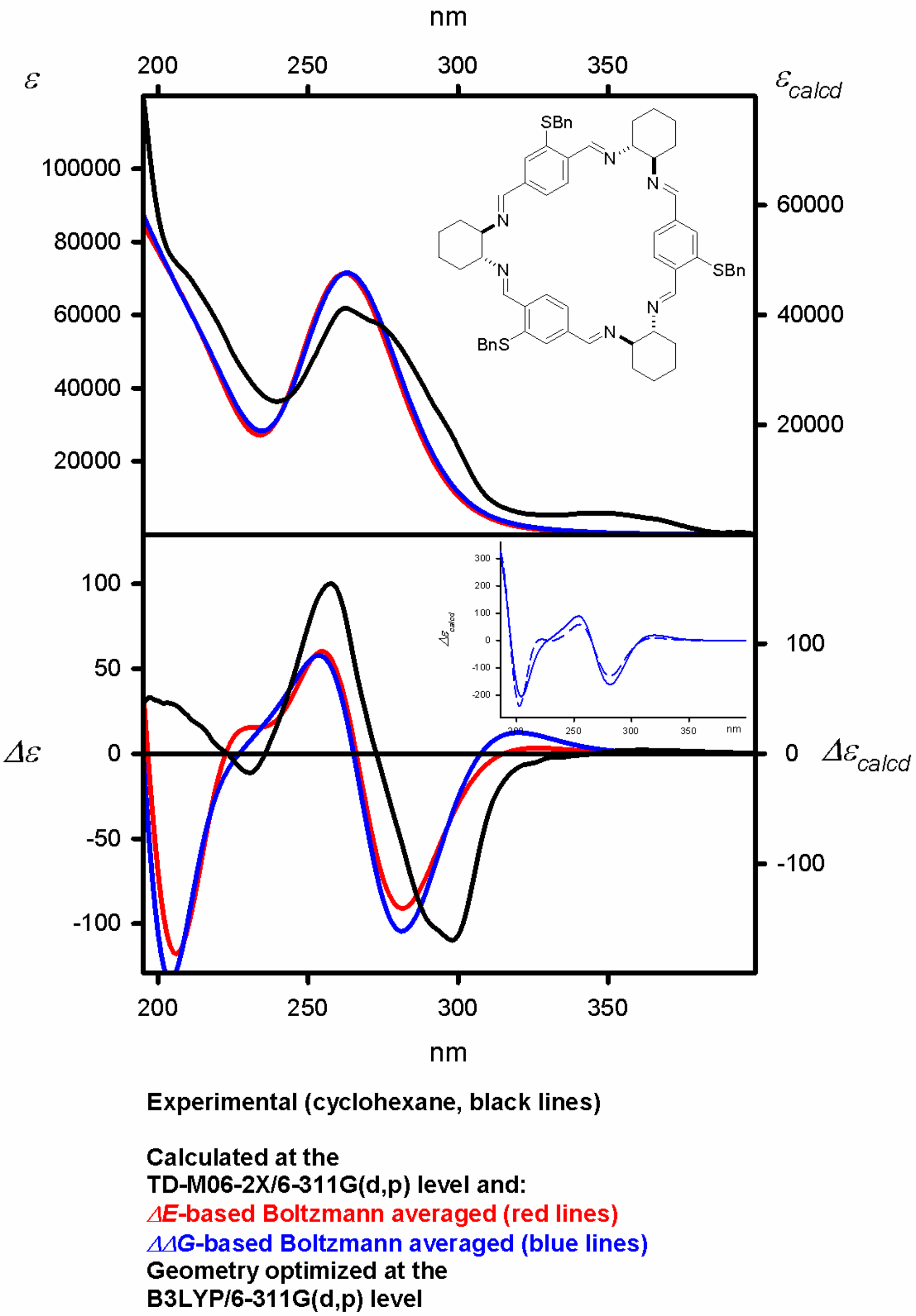

Figure S103. UV (upper panel) and ECD (lower panel) spectra of **6h** measured in cyclohexane (solid black lines) and calculated at the TD-M06-2X/6-311G(d,p) level for geometries optimized at the B3LYP/6-311G(d,p) level. The calculated ECD spectra were Boltzmann-averaged based on *ΔE* (red lines) and *ΔΔG* values (blue lines). Wavelengths were corrected to match the experimental UV maxima. The insert shows the comparison between the ECD spectra calculated for the lowest energy conformer of a given compound (dashed blue lines) and the *ΔΔ*G-based and Boltzmann averaged (solid blue lines). *Δε* values are given in mol^{-1} cm^{-1} dm^{3}.

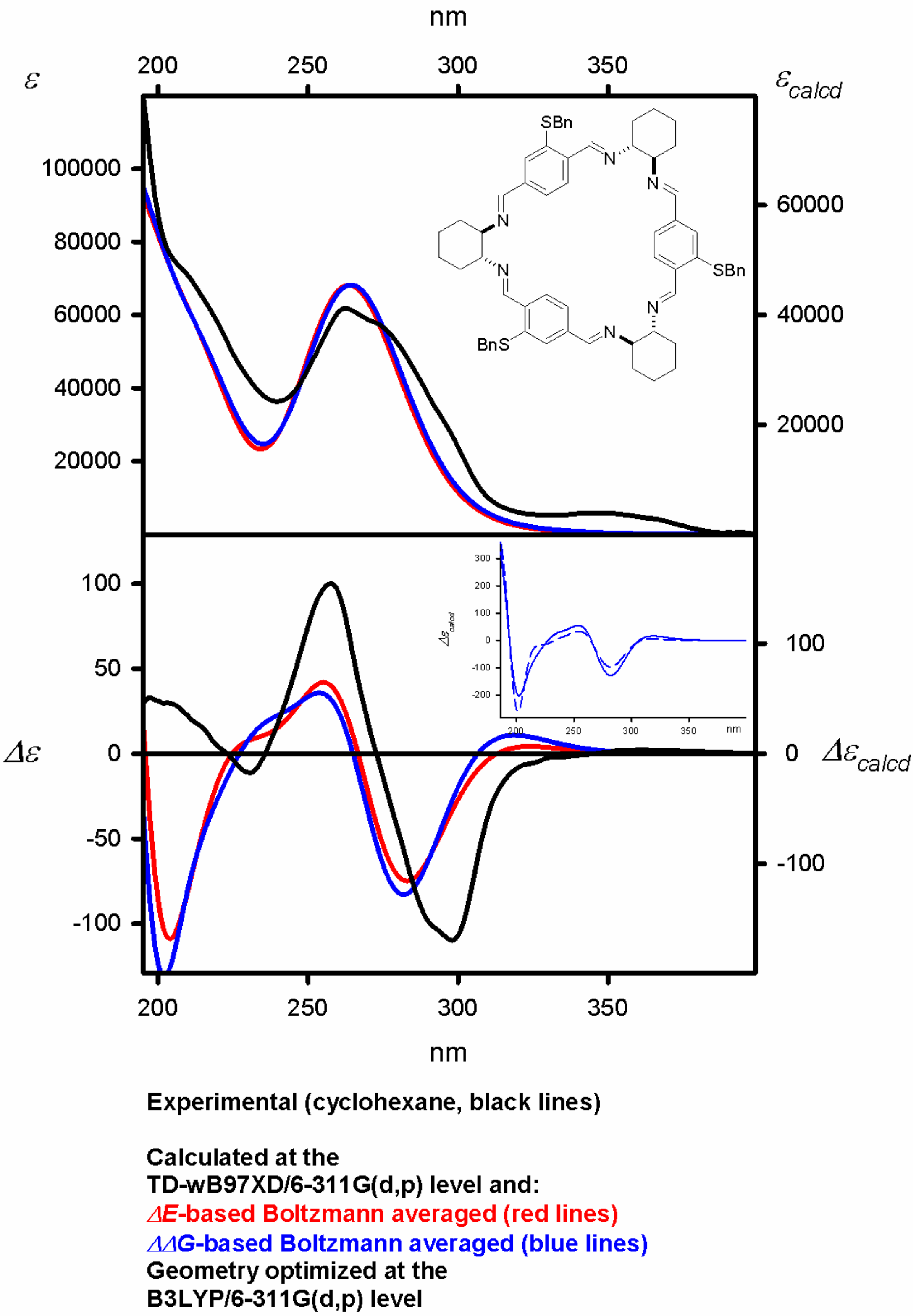

Figure S104. UV (upper panel) and ECD (lower panel) spectra of **6h** measured in cyclohexane (solid black lines) and calculated at the TD-wB97XD/6-311G(d,p) level for geometries optimized at the B3LYP/6-311G(d,p) level. The calculated ECD spectra were Boltzmann-averaged based on ΔE (red lines) and $\Delta\Delta G$ values (blue lines). Wavelengths were corrected to match the experimental UV maxima. The insert shows the comparison between the ECD spectra calculated for the lowest energy conformer of a given compound (dashed blue lines) and the $\Delta\Delta$G-based and Boltzmann averaged (solid blue lines). $\Delta\varepsilon$ values are given in mol^{-1} cm^{-1} dm^{3}.

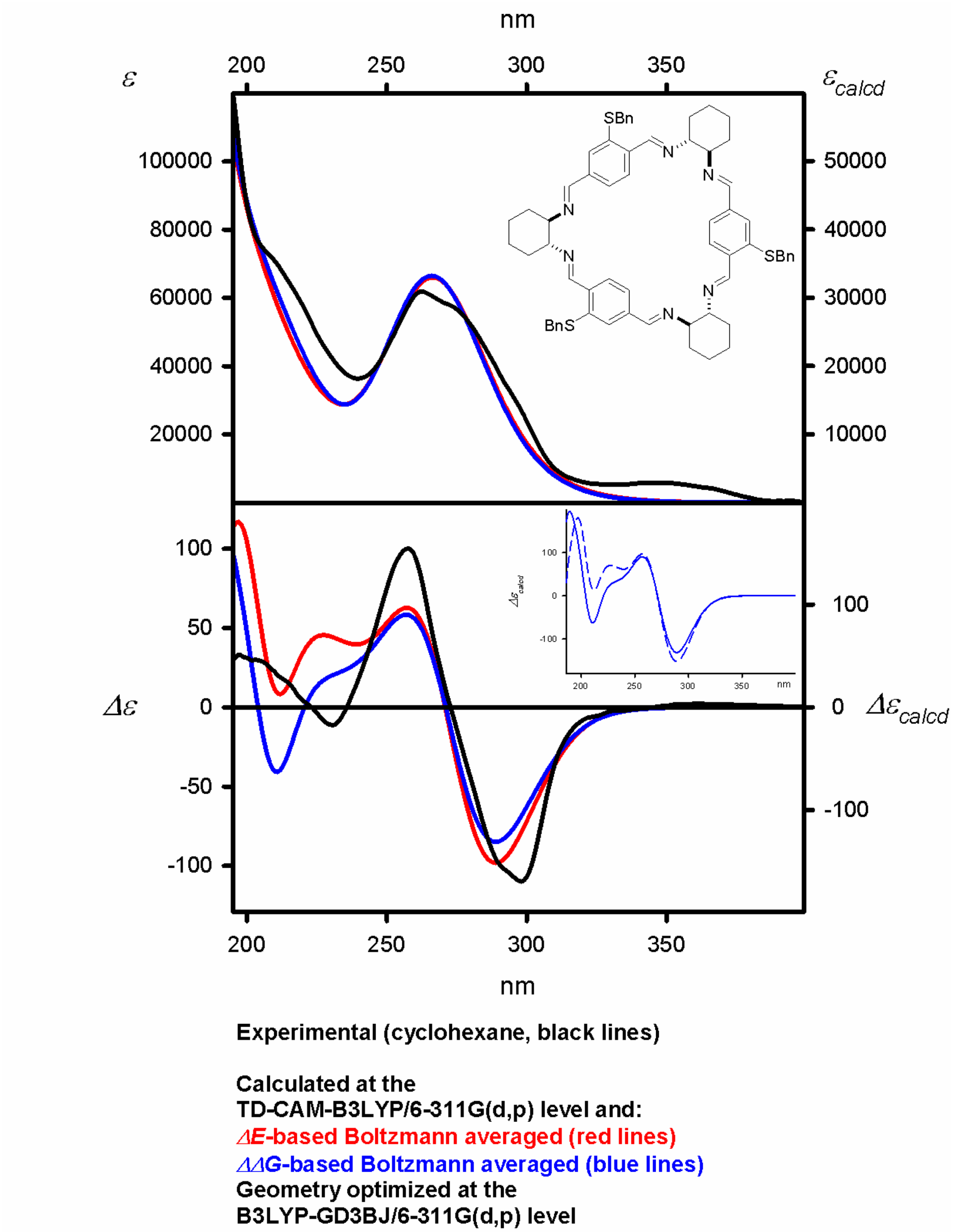

Figure S105. UV (upper panel) and ECD (lower panel) spectra of **6h** measured in cyclohexane (solid black lines) and calculated at the TD-CAM-B3LYP/6-311G(d,p) level for geometries optimized at the B3LYP-GD3BJ/6-311G(d,p) level. The calculated ECD spectra were Boltzmann-averaged based on *ΔE* (red lines) and *ΔΔG* values (blue lines). Wavelengths were corrected to match the experimental UV maxima. The insert shows the comparison between the ECD spectra calculated for the lowest energy conformer of a given compound (dashed blue lines) and the *ΔΔ*G-based and Boltzmann averaged (solid blue lines). *Δε* values are given in mol^{-1} cm^{-1} dm^{3}.

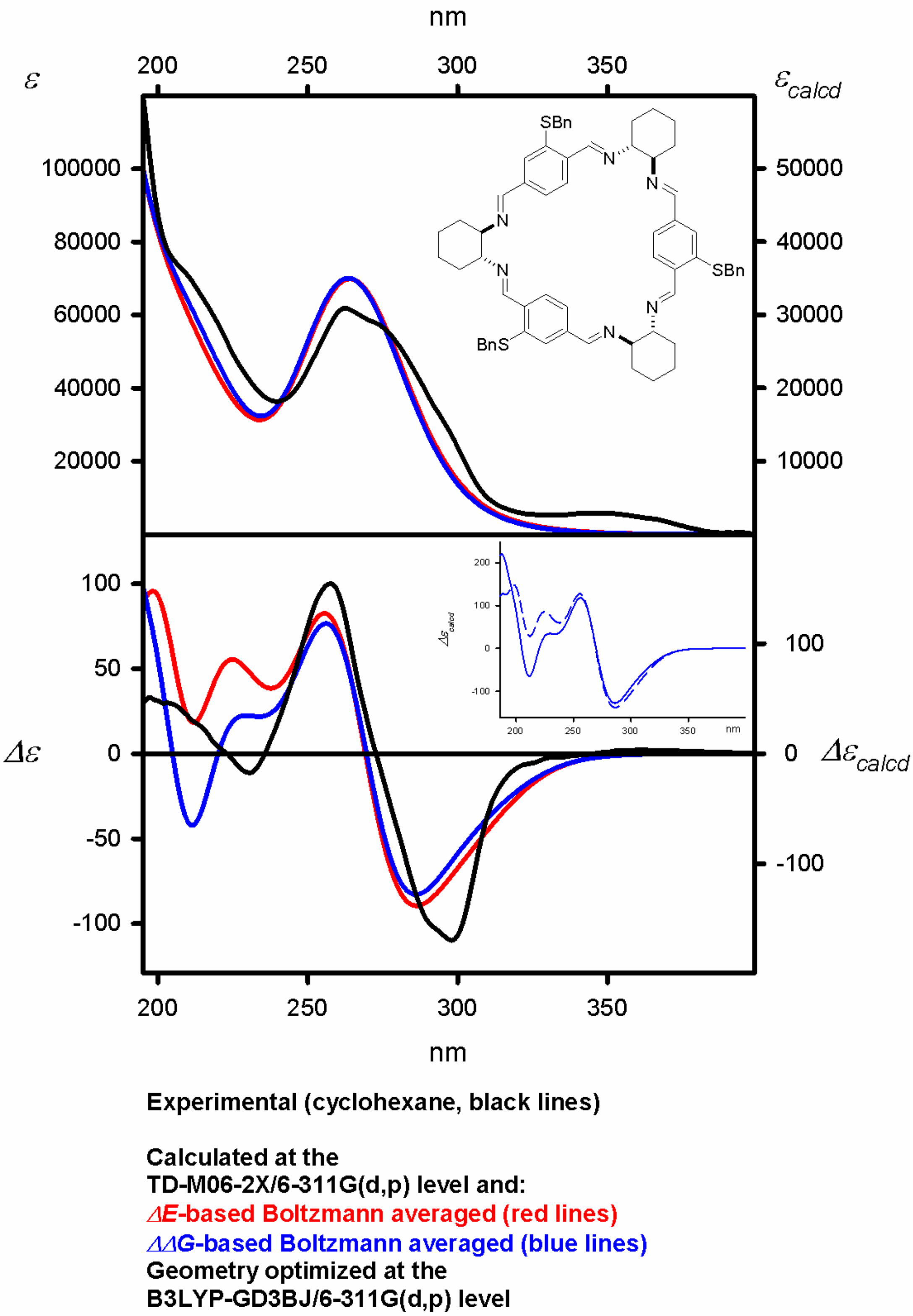

Figure S106. UV (upper panel) and ECD (lower panel) spectra of **6h** measured in cyclohexane (solid black lines) and calculated at the TD-M06-2X/6-311G(d,p) level for geometries optimized at the B3LYP-GD3BJ/6-311G(d,p) level. The calculated ECD spectra were Boltzmann-averaged based on *ΔE* (red lines) and *ΔΔG* values (blue lines). Wavelengths were corrected to match the experimental UV maxima. The insert shows the comparison between the ECD spectra calculated for the lowest energy conformer of a given compound (dashed blue lines) and the *ΔΔ*G-based and Boltzmann averaged (solid blue lines). *Δε* values are given in mol^{-1} cm^{-1} dm^{3}.

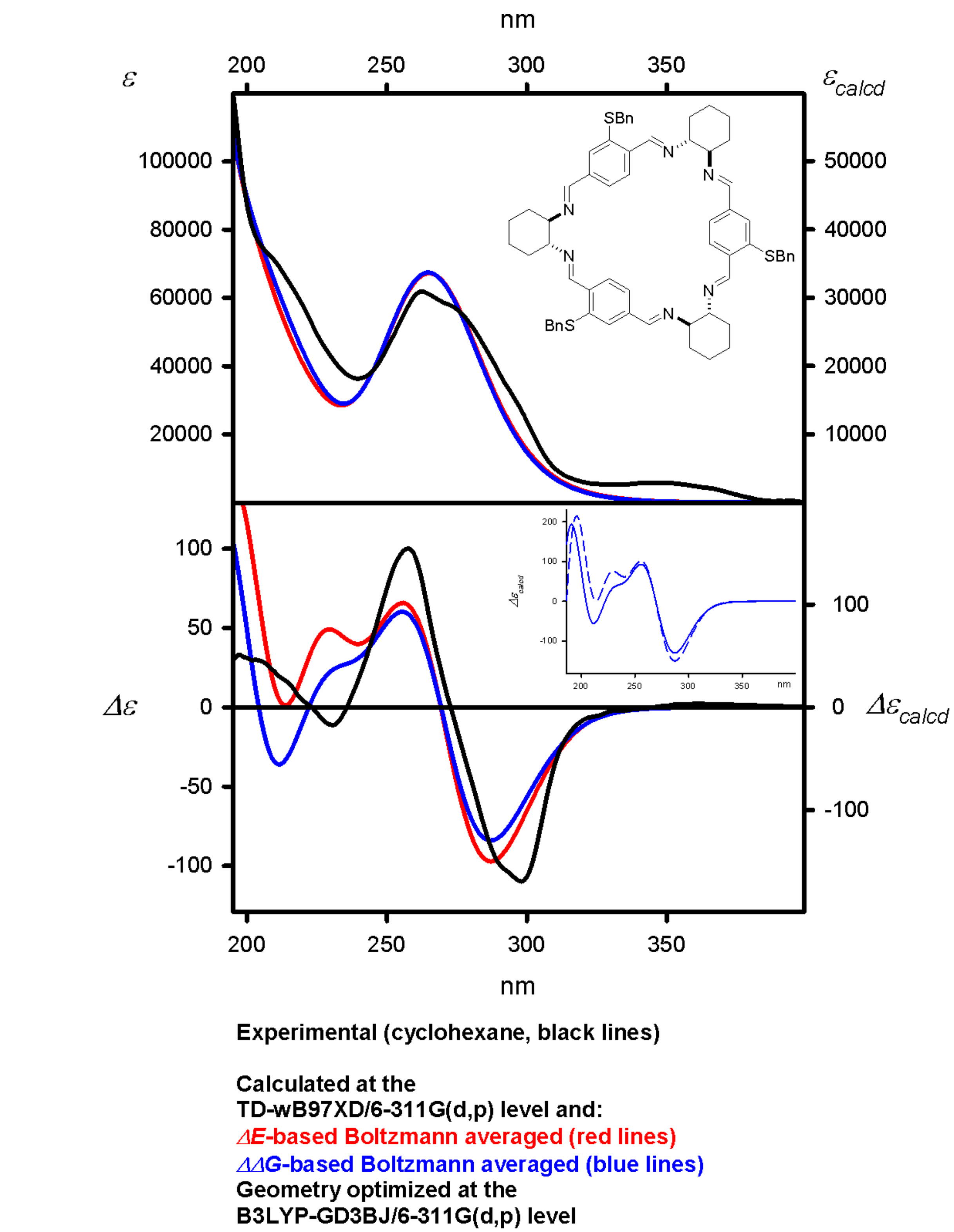

Figure S107. UV (upper panel) and ECD (lower panel) spectra of **6h** measured in cyclohexane (solid black lines) and calculated at the TD-wB97XD/6-311G(d,p) level for geometries optimized at the B3LYP-GD3BJ/6-311G(d,p) level. The calculated ECD spectra were Boltzmann-averaged based on *ΔE* (red lines) and *ΔΔG* values (blue lines). Wavelengths were corrected to match the experimental UV maxima. The insert shows the comparison between the ECD spectra calculated for the lowest energy conformer of a given compound (dashed blue lines) and the *ΔΔ*G-based and Boltzmann averaged (solid blue lines). *Δε* values are given in mol^{-1} cm^{-1} dm^{3}.

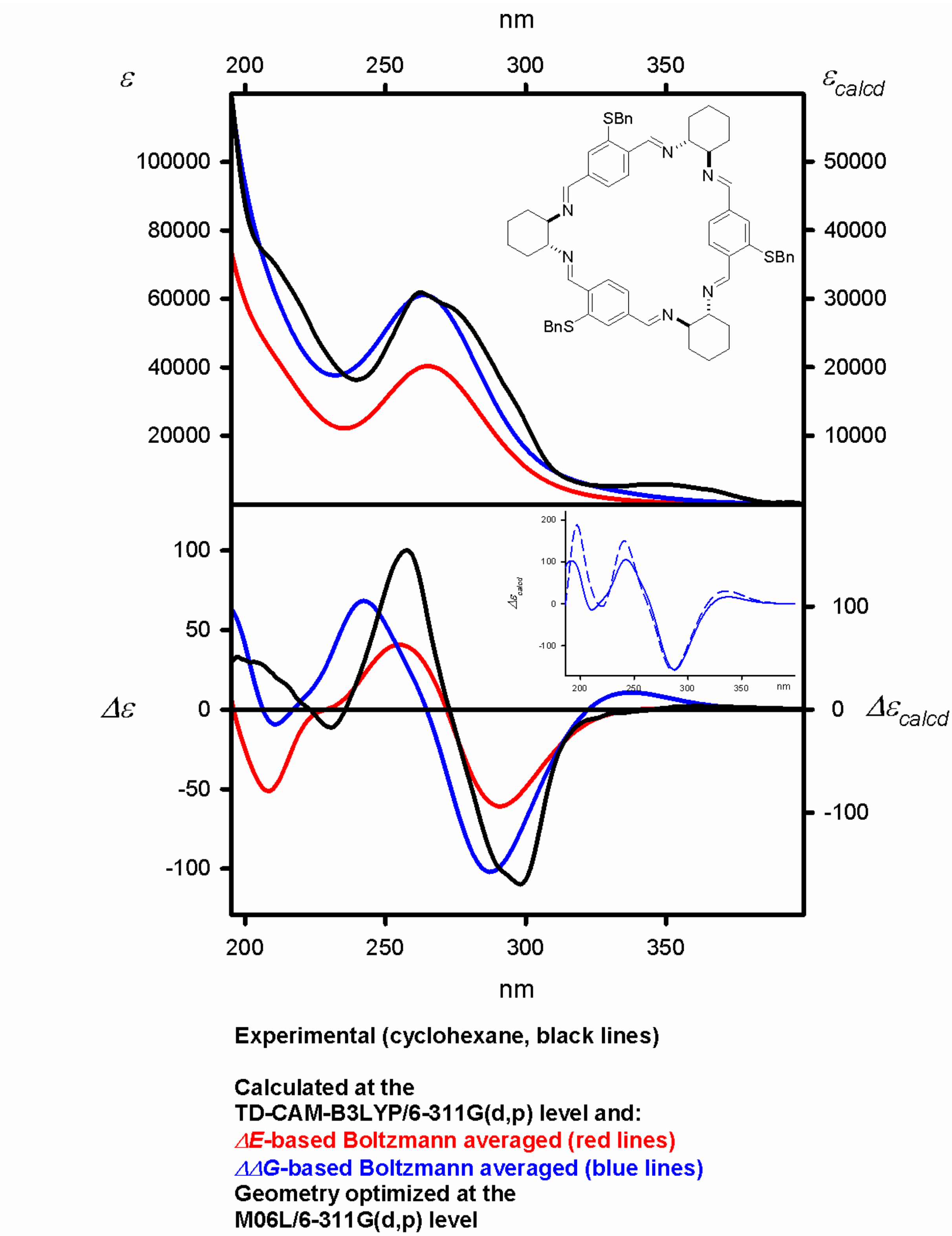

Figure S108. UV (upper panel) and ECD (lower panel) spectra of **6h** measured in cyclohexane (solid black lines) and calculated at the TD-CAM-B3LYP/6-311G(d,p) level for geometries optimized at the M06L/6-311G(d,p) level. The calculated ECD spectra were Boltzmann-averaged based on *ΔE* (red lines) and *ΔΔG* values (blue lines). Wavelengths were corrected to match the experimental UV maxima. The insert shows the comparison between the ECD spectra calculated for the lowest energy conformer of a given compound (dashed blue lines) and the *ΔΔ*G-based and Boltzmann averaged (solid blue lines). *Δε* values are given in mol^{-1} cm^{-1} dm^{3}.

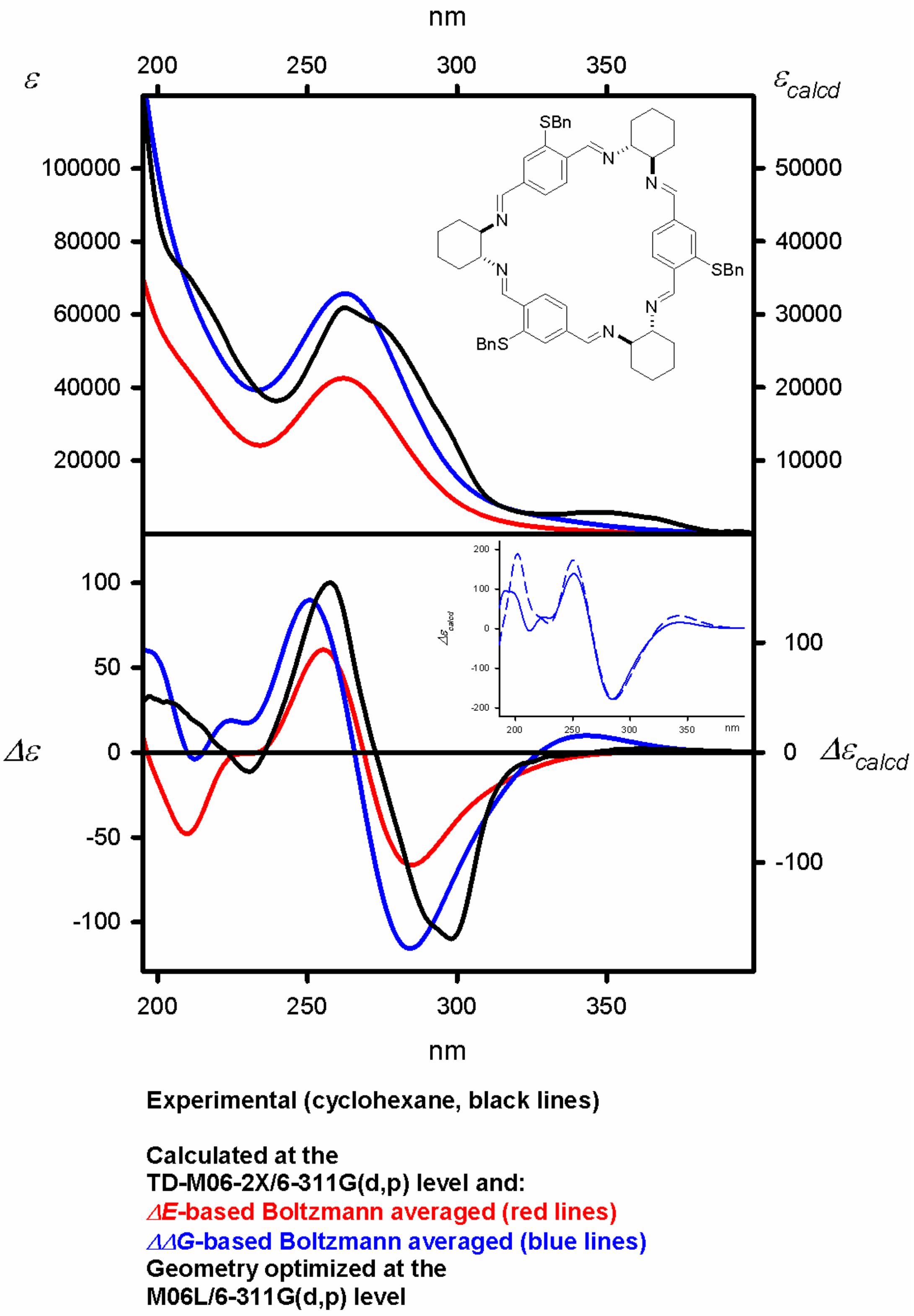

Figure S109. UV (upper panel) and ECD (lower panel) spectra of **6h** measured in cyclohexane (solid black lines) and calculated at the TD-M06-2X/6-311G(d,p) level for geometries optimized at the M06L/6-311G(d,p) level. The calculated ECD spectra were Boltzmann-averaged based on *ΔE* (red lines) and *ΔΔG* values (blue lines). Wavelengths were corrected to match the experimental UV maxima. The insert shows the comparison between the ECD spectra calculated for the lowest energy conformer of a given compound (dashed blue lines) and the *ΔΔ*G-based and Boltzmann averaged (solid blue lines). *Δε* values are given in mol^{-1} cm^{-1} dm^{3}.

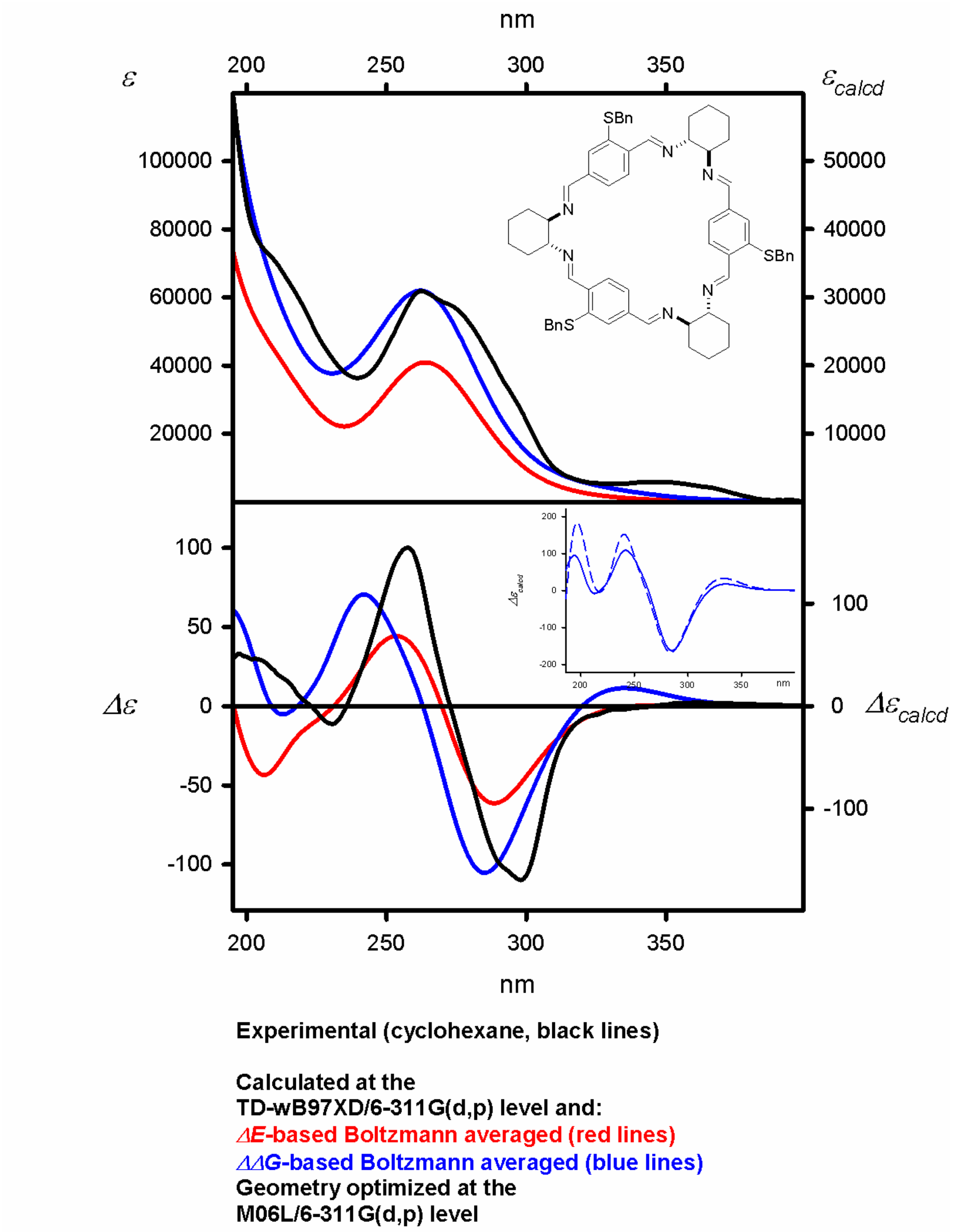

Figure S110. UV (upper panel) and ECD (lower panel) spectra of **6h** measured in cyclohexane (solid black lines) and calculated at the TD-wB97XD/6-311G(d,p) level for geometries optimized at the M06L/6-311G(d,p) level. The calculated ECD spectra were Boltzmann-averaged based on ΔE (red lines) and $\Delta\Delta G$ values (blue lines). Wavelengths were corrected to match the experimental UV maxima. The insert shows the comparison between the ECD spectra calculated for the lowest energy conformer of a given compound (dashed blue lines) and the $\Delta\Delta$G-based and Boltzmann averaged (solid blue lines). $\Delta\varepsilon$ values are given in mol^{-1} cm^{-1} dm^{3}.

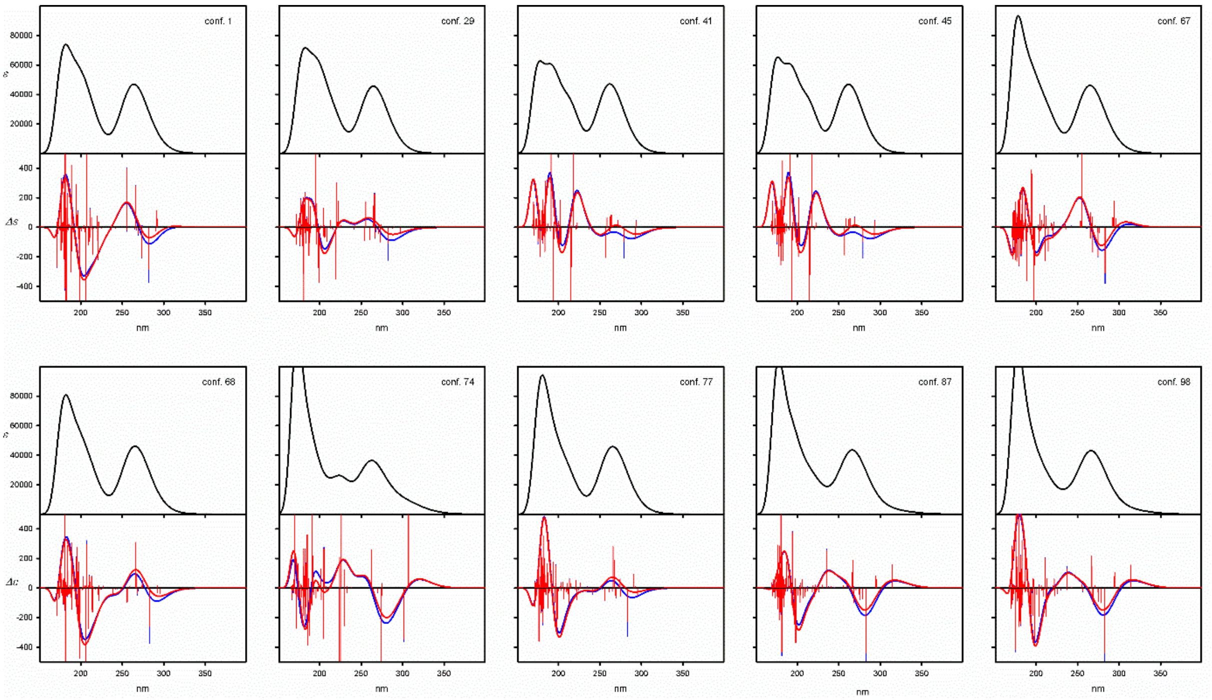

Figure S111. UV (upper panels) and ECD (lower panels) spectra calculated at the TD-CAM-B3LYP/6-311G(d,p) level for individual, symmetrical low-energy conformers of **6h**. Wavelengths were not corrected. Geometries were optimized at the B3LYP/6-311G(d,p) level. $\Delta\varepsilon$ values are given in mol^{-1} cm^{-1} dm^{3}.

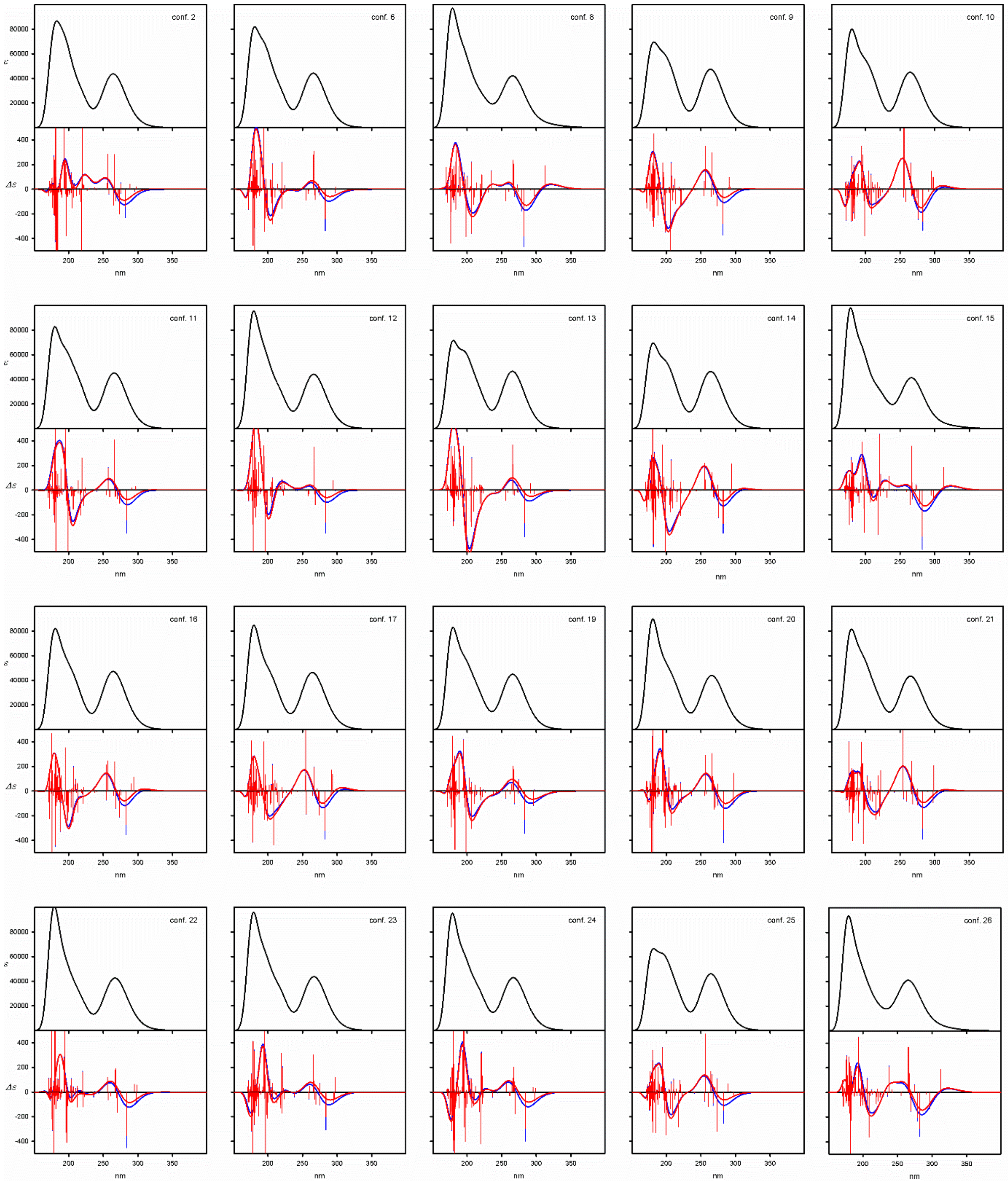
conf. 2
conf. 6
conf. 8
conf. 9
conf. 10
conf. 11
conf. 12
conf. 13
conf. 14
conf. 15
conf. 16
conf. 17
conf. 19
conf. 20
conf. 21
conf. 22
conf. 23
conf. 24
conf. 25
conf. 26
ε
Δε
nm

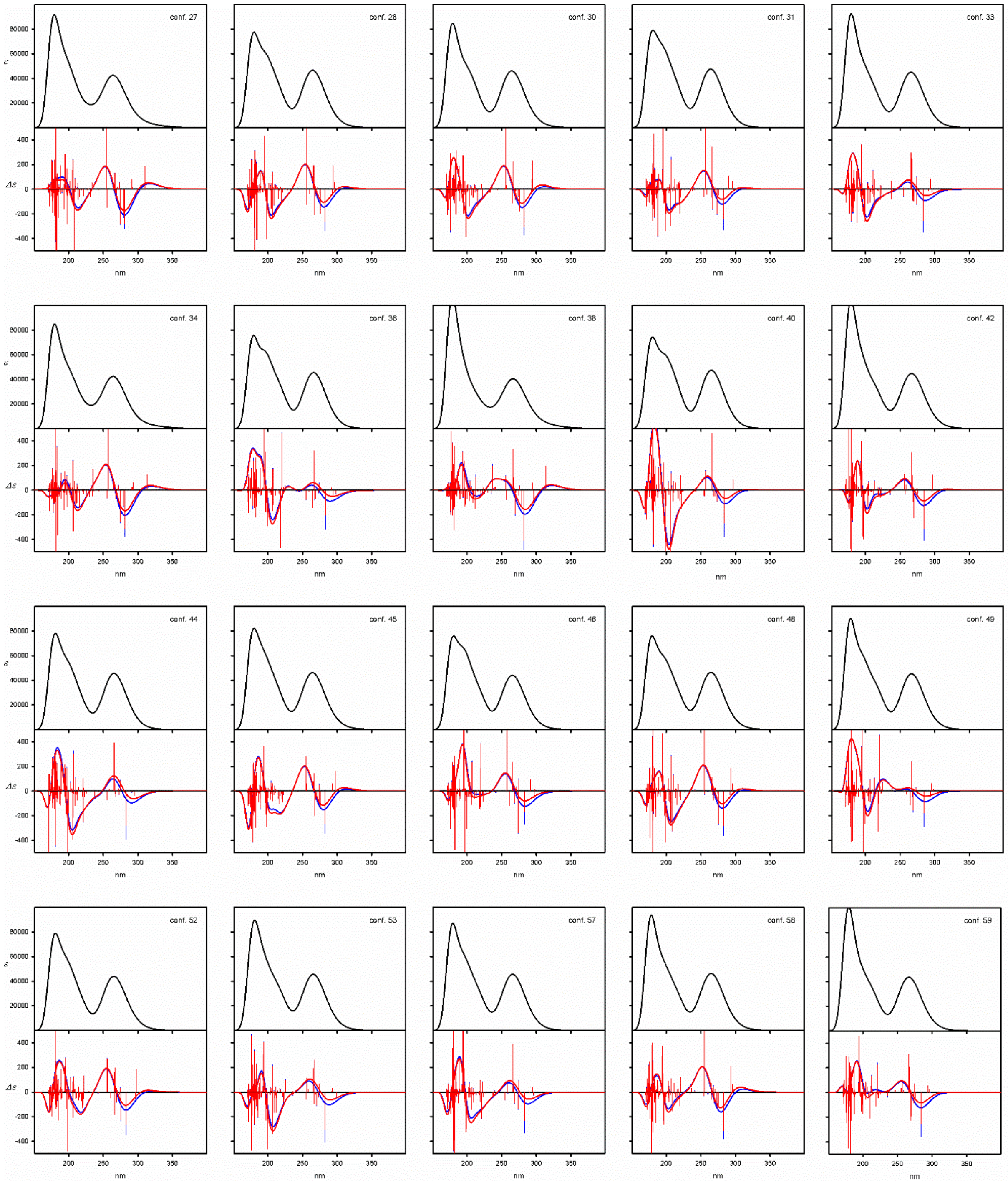
conf. 27
conf. 28
conf. 30
conf. 31
conf. 33
conf. 34
conf. 36
conf. 38
conf. 40
conf. 42
conf. 44
conf. 45
conf. 46
conf. 48
conf. 49
conf. 52
conf. 53
conf. 57
conf. 58
conf. 59
nm

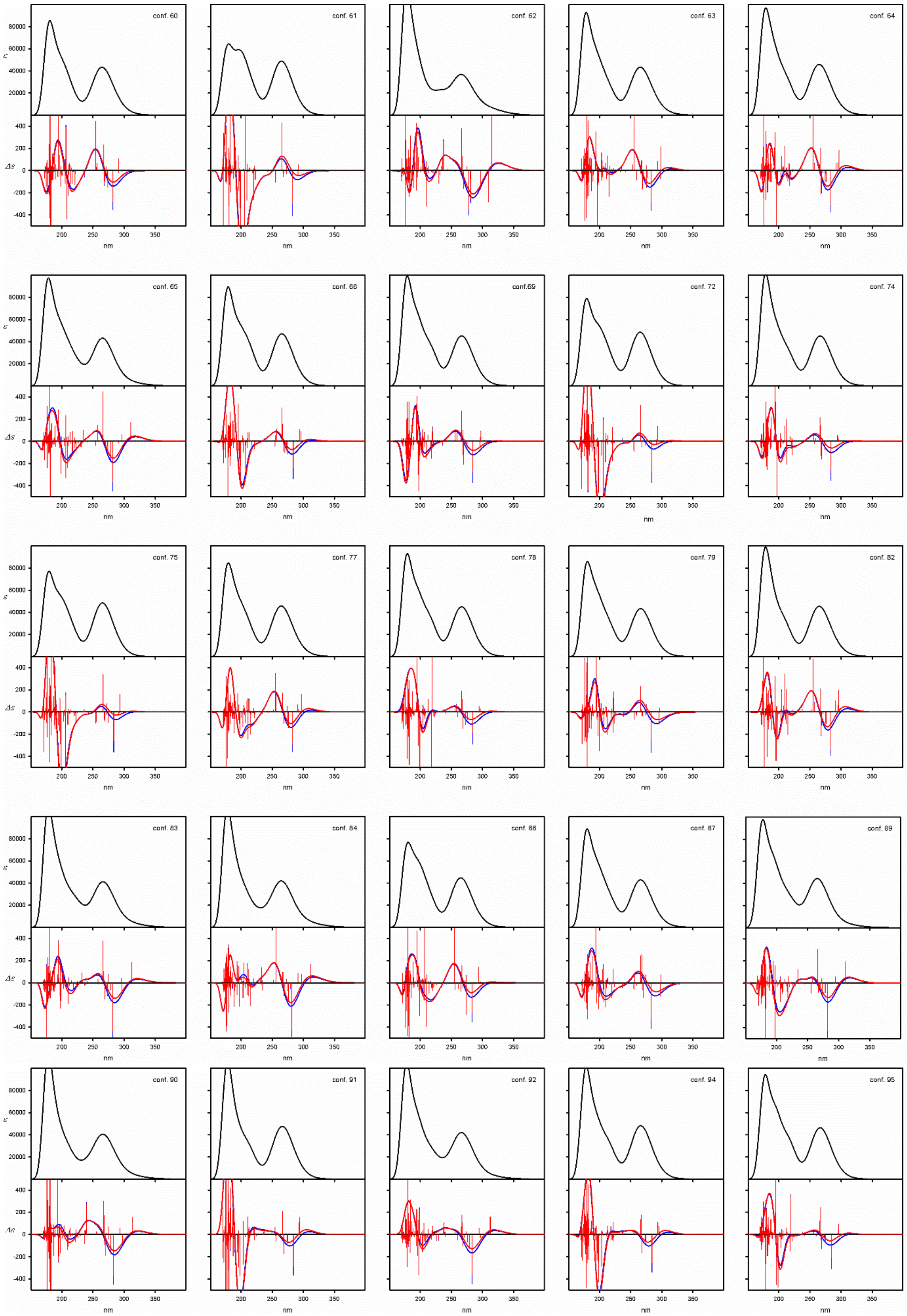

Figure S112. UV (upper panels) and ECD (lower panels) spectra calculated at the TD-CAM-B3LYP/6-311G(d,p) level for individual, non-symmetrical low-energy conformers of **6h**. Wavelengths were not corrected. Geometries were optimized at the B3LYP/6-311G(d,p) level. *Δε* values are given in mol^{-1} cm^{-1} dm^{3}.

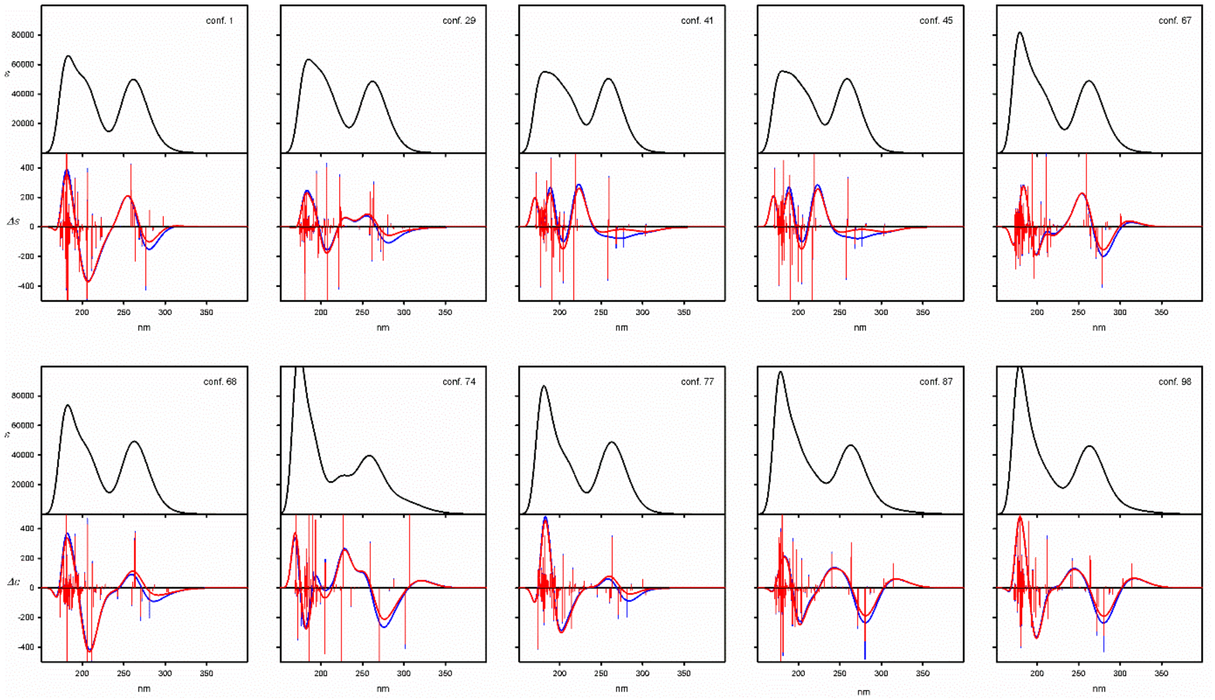

Figure S113. UV (upper panels) and ECD (lower panels) spectra calculated at the TD-M06-2X/6-311G(d,p) level for individual, symmetrical low-energy conformers of **6h**. Wavelengths were not corrected. Geometries were optimized at the B3LYP/6-311G(d,p) level. $\Delta\varepsilon$ values are given in mol^{-1} cm^{-1} dm^{3}.

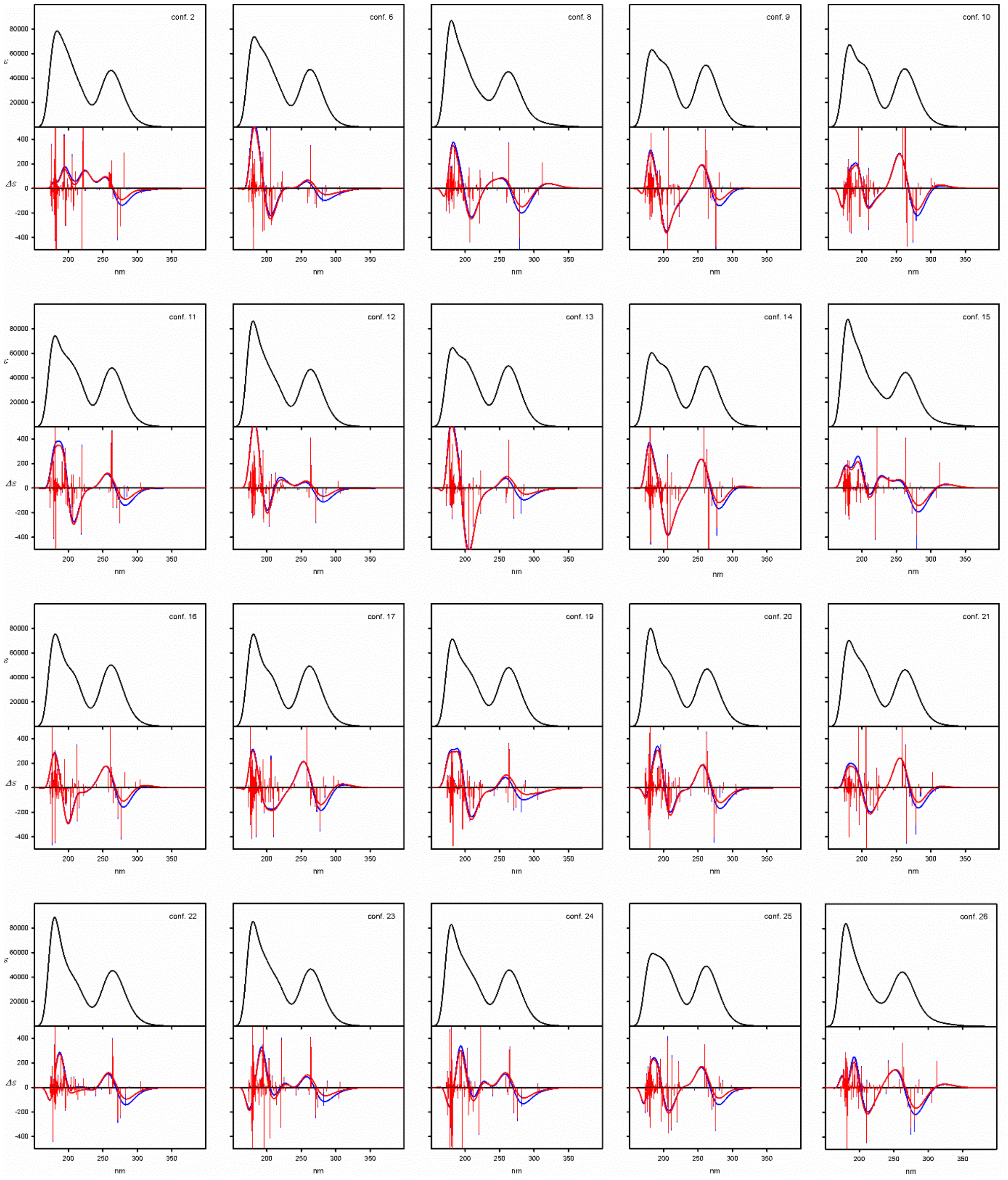
conf. 2
conf. 6
conf. 8
conf. 9
conf. 10
conf. 11
conf. 12
conf. 13
conf. 14
conf. 15
conf. 16
conf. 17
conf. 19
conf. 20
conf. 21
conf. 22
conf. 23
conf. 24
conf. 25
conf. 26
ε
Δε
80000
60000
40000
20000
400
200
0
-200
-400
200
250
300
350
nm

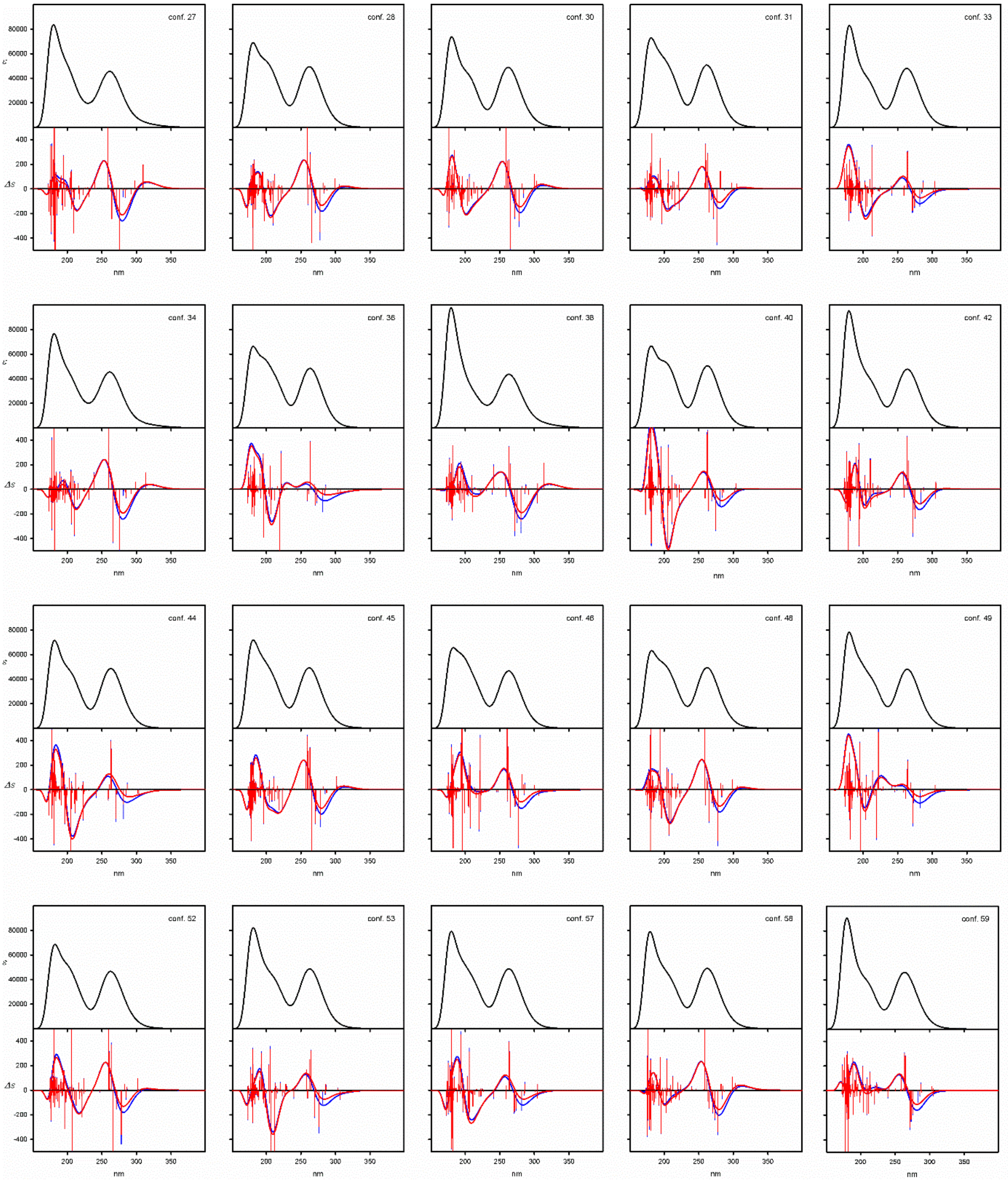
conf. 27
conf. 28
conf. 30
conf. 31
conf. 33
conf. 34
conf. 36
conf. 38
conf. 40
conf. 42
conf. 44
conf. 45
conf. 46
conf. 48
conf. 49
conf. 52
conf. 53
conf. 57
conf. 58
conf. 59
ε
Δε
nm

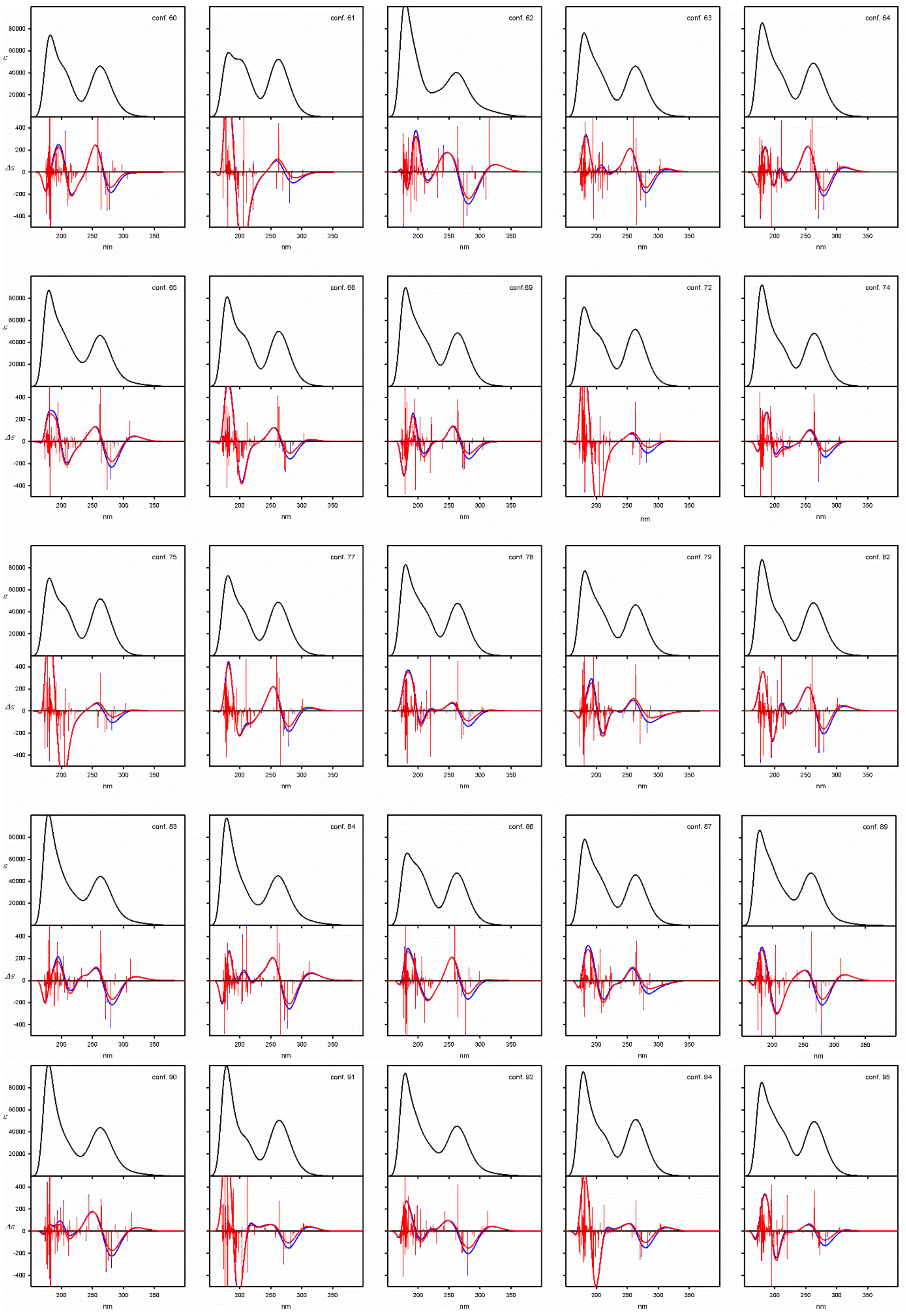

Figure S114. UV (upper panels) and ECD (lower panels) spectra calculated at the TD-M06-2X/6-311G(d,p) level for individual, non-symmetrical low-energy conformers of **6h**. Wavelengths were not corrected. Geometries were optimized at the B3LYP/6-311G(d,p) level. *Δε* values are given in mol^{-1} cm^{-1} dm^{3}.

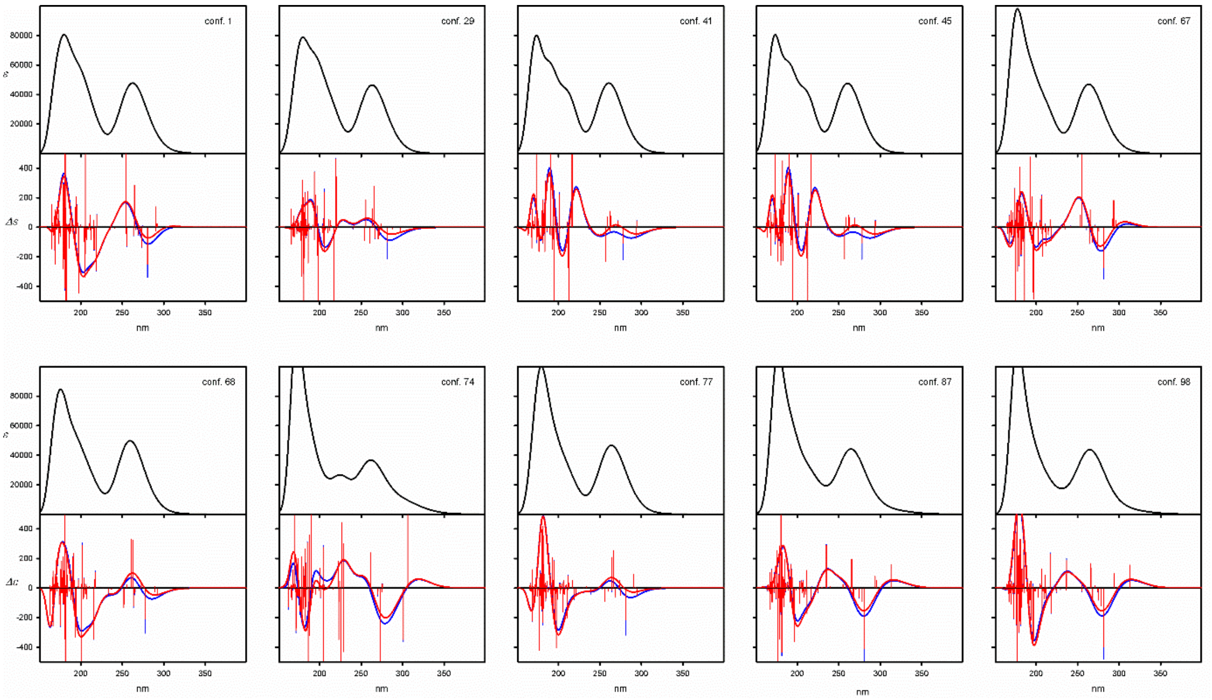

Figure S115. UV (upper panels) and ECD (lower panels) spectra calculated at the TD-wB97XD/6-311G(d,p) level for individual, symmetrical low-energy conformers of **6h**. Wavelengths were not corrected. Geometries were optimized at the B3LYP/6-311G(d,p) level. *Δε* values are given in $mol^{-1} cm^{-1} dm^3$.

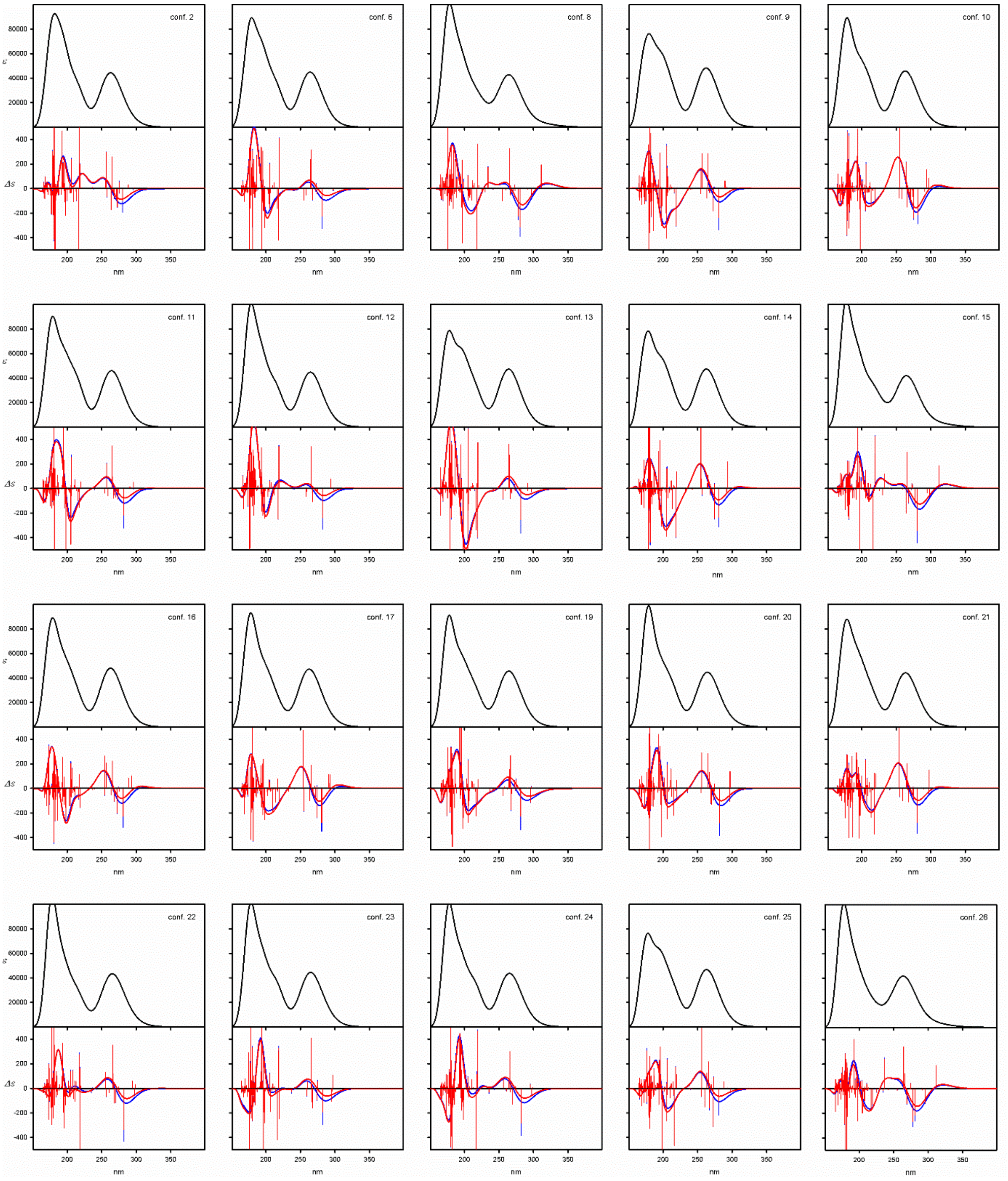
conf. 2
conf. 6
conf. 8
conf. 9
conf. 10
conf. 11
conf. 12
conf. 13
conf. 14
conf. 15
conf. 16
conf. 17
conf. 19
conf. 20
conf. 21
conf. 22
conf. 23
conf. 24
conf. 25
conf. 26
ε
Δε
80000
60000
40000
20000
400
200
0
-200
-400
200
250
300
350
nm

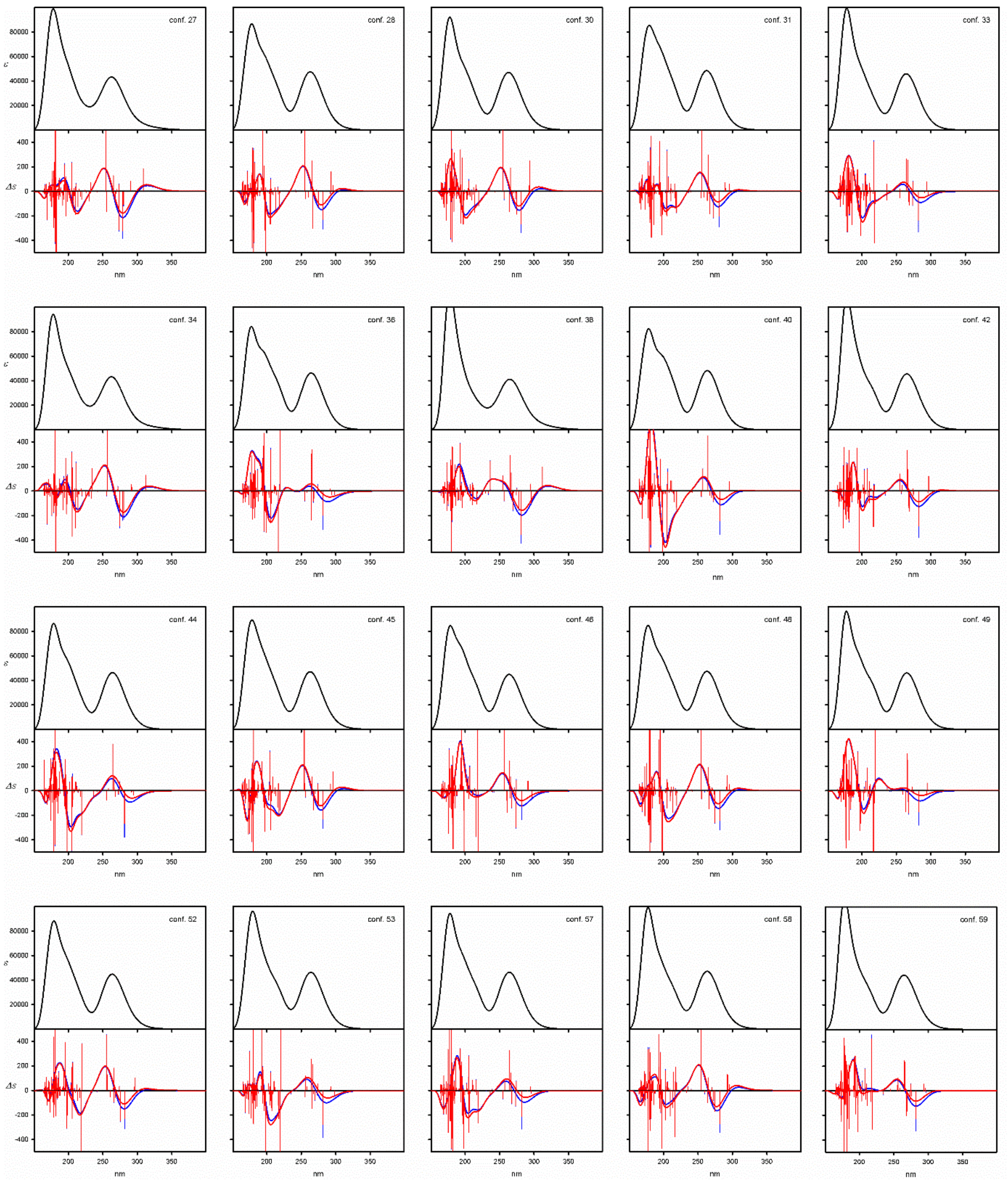
conf. 27
conf. 28
conf. 30
conf. 31
conf. 33
conf. 34
conf. 36
conf. 38
conf. 40
conf. 42
conf. 44
conf. 45
conf. 46
conf. 48
conf. 49
conf. 52
conf. 53
conf. 57
conf. 58
conf. 59
ε
Δε
nm

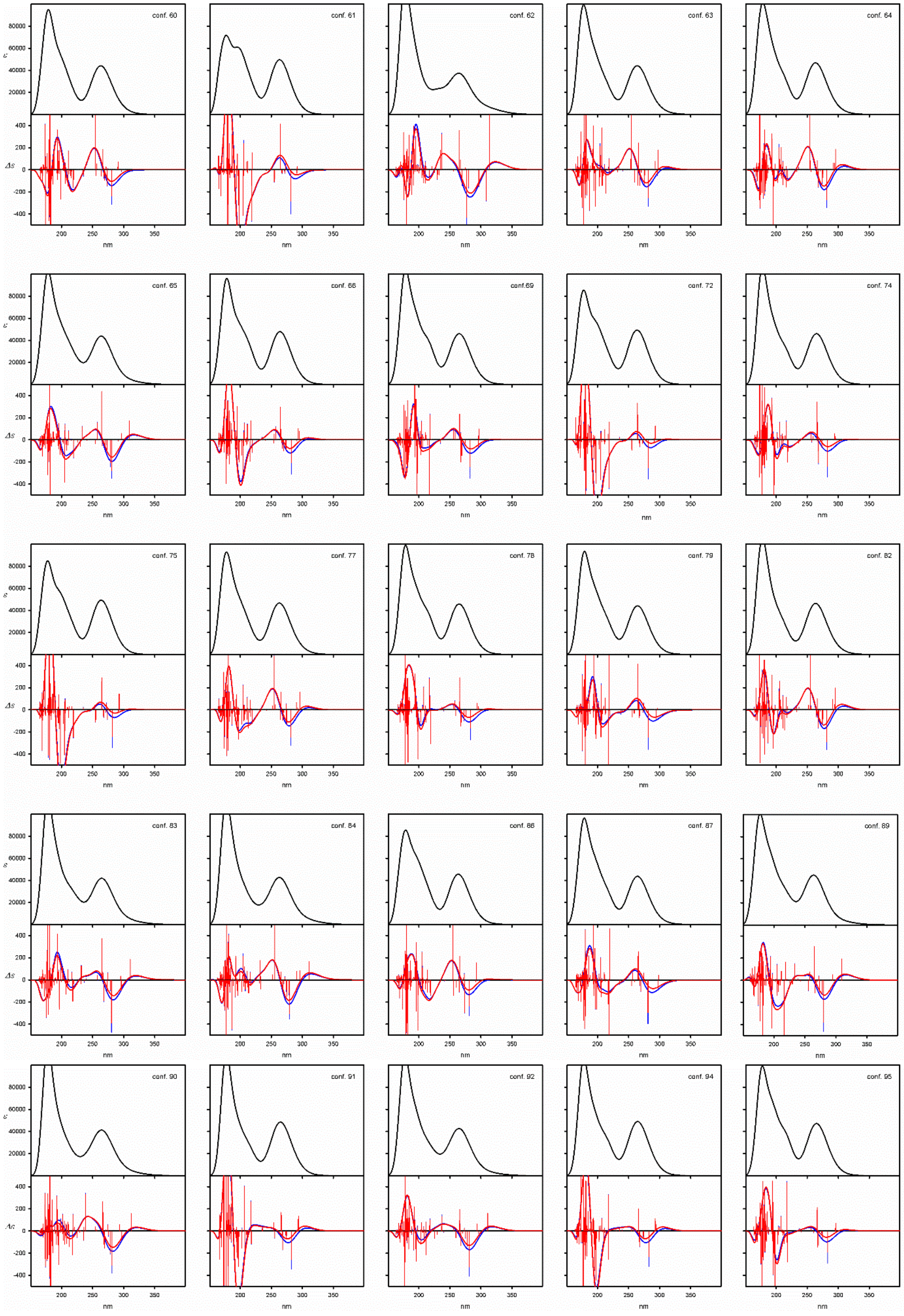

Figure S116. UV (upper panels) and ECD (lower panels) spectra calculated at the TD-wB97XD/6-311G(d,p) level for individual, non-symmetrical low-energy conformers of **6h**. Wavelengths were not corrected. Geometries were optimized at the B3LYP/6-311G(d,p) level. *Δε* values are given in mol^{-1} cm^{-1} dm^{3}.

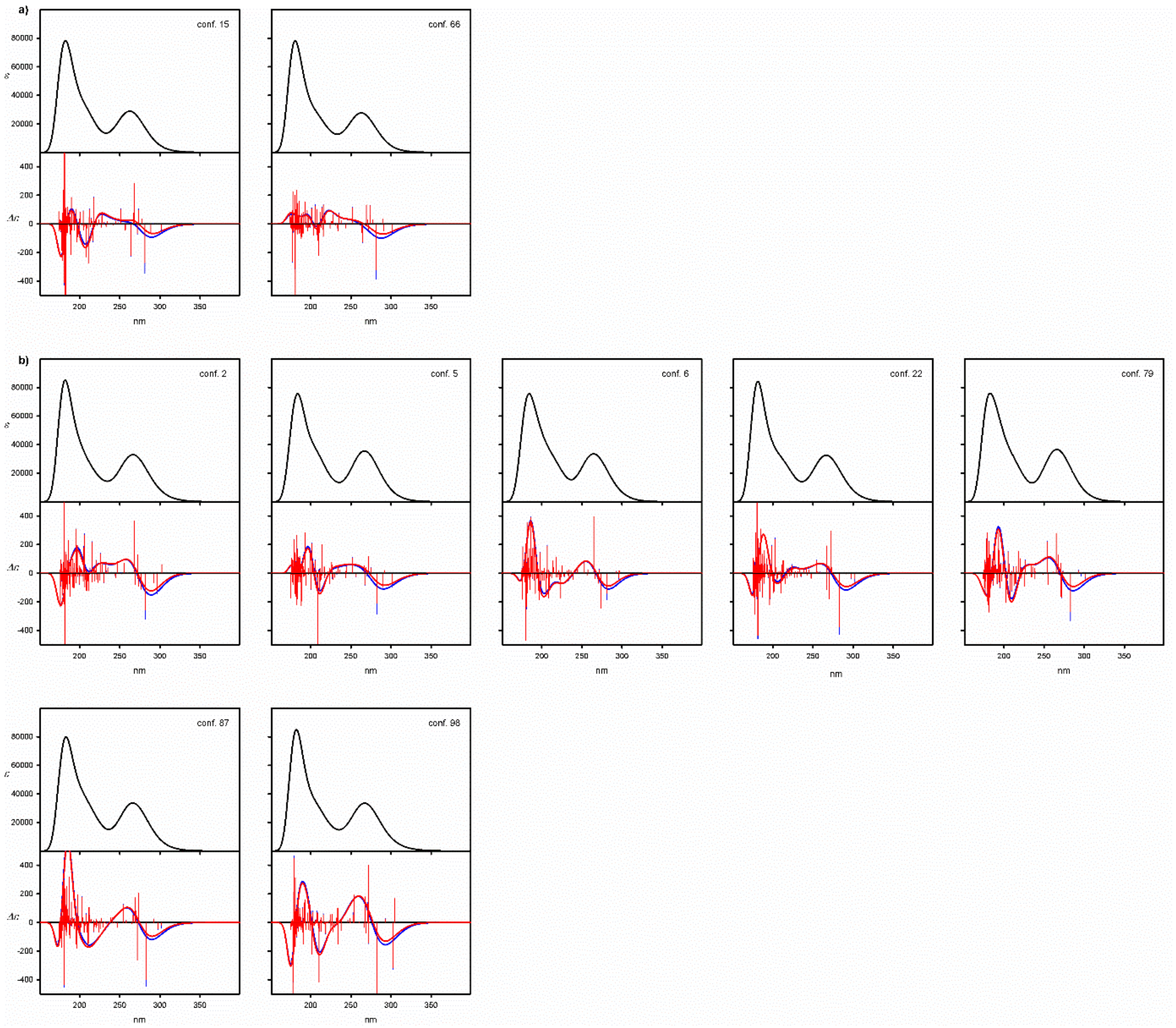

Figure S117. UV (upper panels) and ECD (lower panels) spectra calculated at the TD-CAM-B3LYP/6-311G(d,p) level for individual, a) symmetrical and b) non-symmetrical low-energy conformers of **6h**. Wavelengths were not corrected. Geometries were optimized at the B3LYP-GD3BJ/6-311G(d,p) level. $\Delta\varepsilon$ values are given in mol^{-1} cm^{-1} dm^{3}.

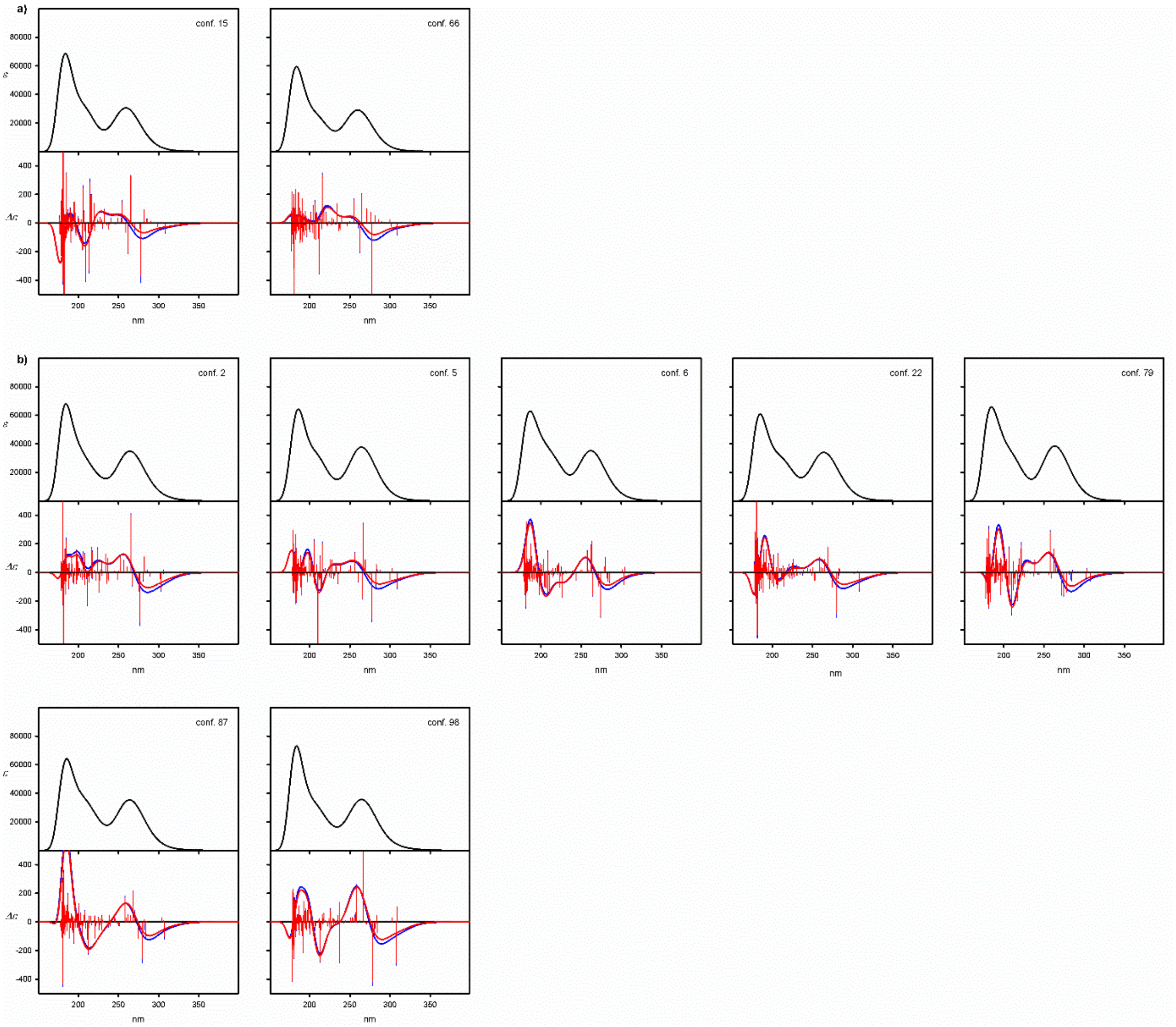

Figure S118. UV (upper panels) and ECD (lower panels) spectra calculated at the TD-M06-2X/6-311G(d,p) level for individual, a) symmetrical and b) non-symmetrical low-energy conformers of **6h**. Wavelengths were not corrected. Geometries were optimized at the B3LYP-GD3BJ/6-311G(d,p) level. $\Delta\varepsilon$ values are given in mol^{-1} cm^{-1} dm^{3}.

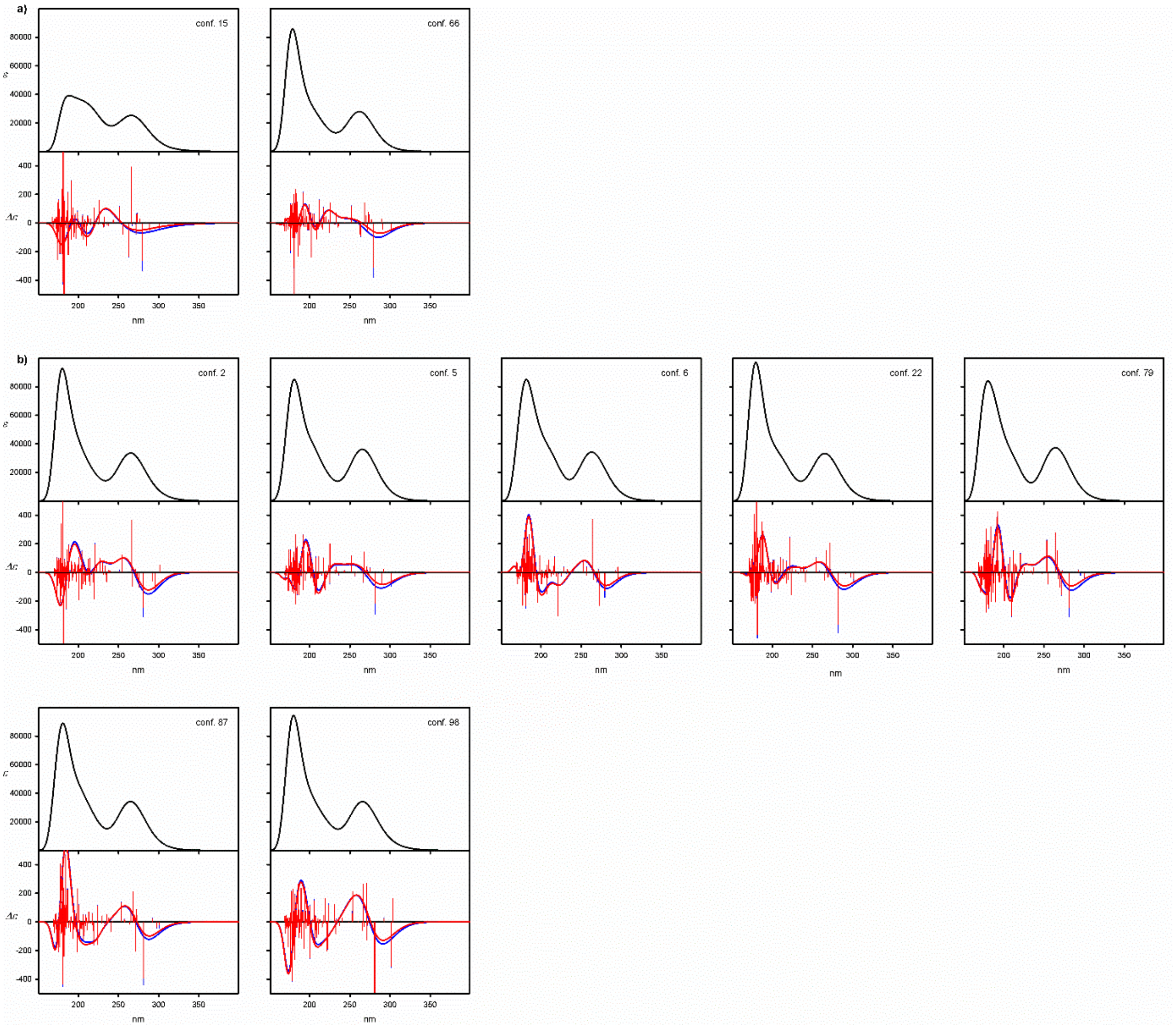

Figure S119. UV (upper panels) and ECD (lower panels) spectra calculated at the TD-wB97XD/6-311G(d,p) level for individual, a) symmetrical and b) non-symmetrical low-energy conformers of **6h**. Wavelengths were not corrected. Geometries were optimized at the B3LYP-GD3BJ/6-311G(d,p) level. $\Delta\varepsilon$ values are given in mol^{-1} cm^{-1} dm^{3}.

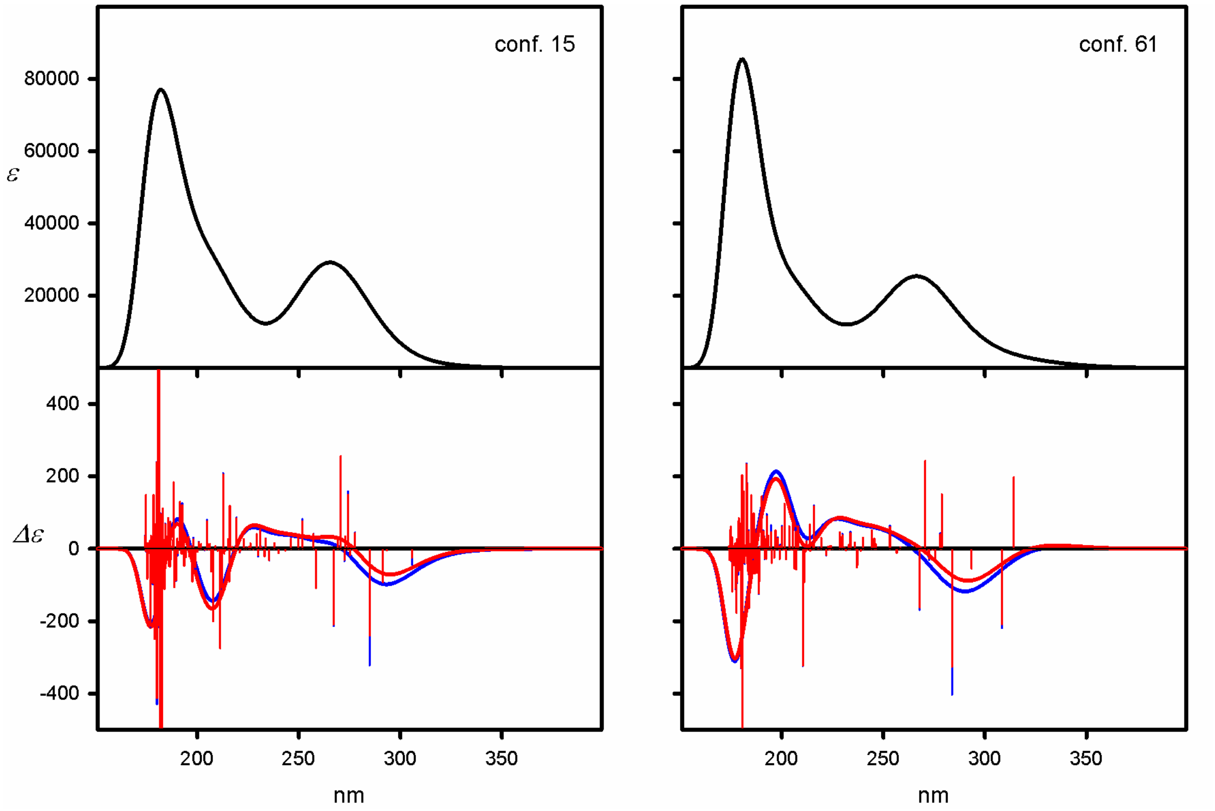

Figure S120. UV (upper panels) and ECD (lower panels) spectra calculated at the TD-CAM-B3LYP/6-311G(d,p) level for individual, symmetrical low-energy conformers of **6h**. Wavelengths were not corrected. Geometries were optimized at the M06L/6-311G(d,p) level. *Δε* values are given in mol^{-1} cm^{-1} dm^{3}.

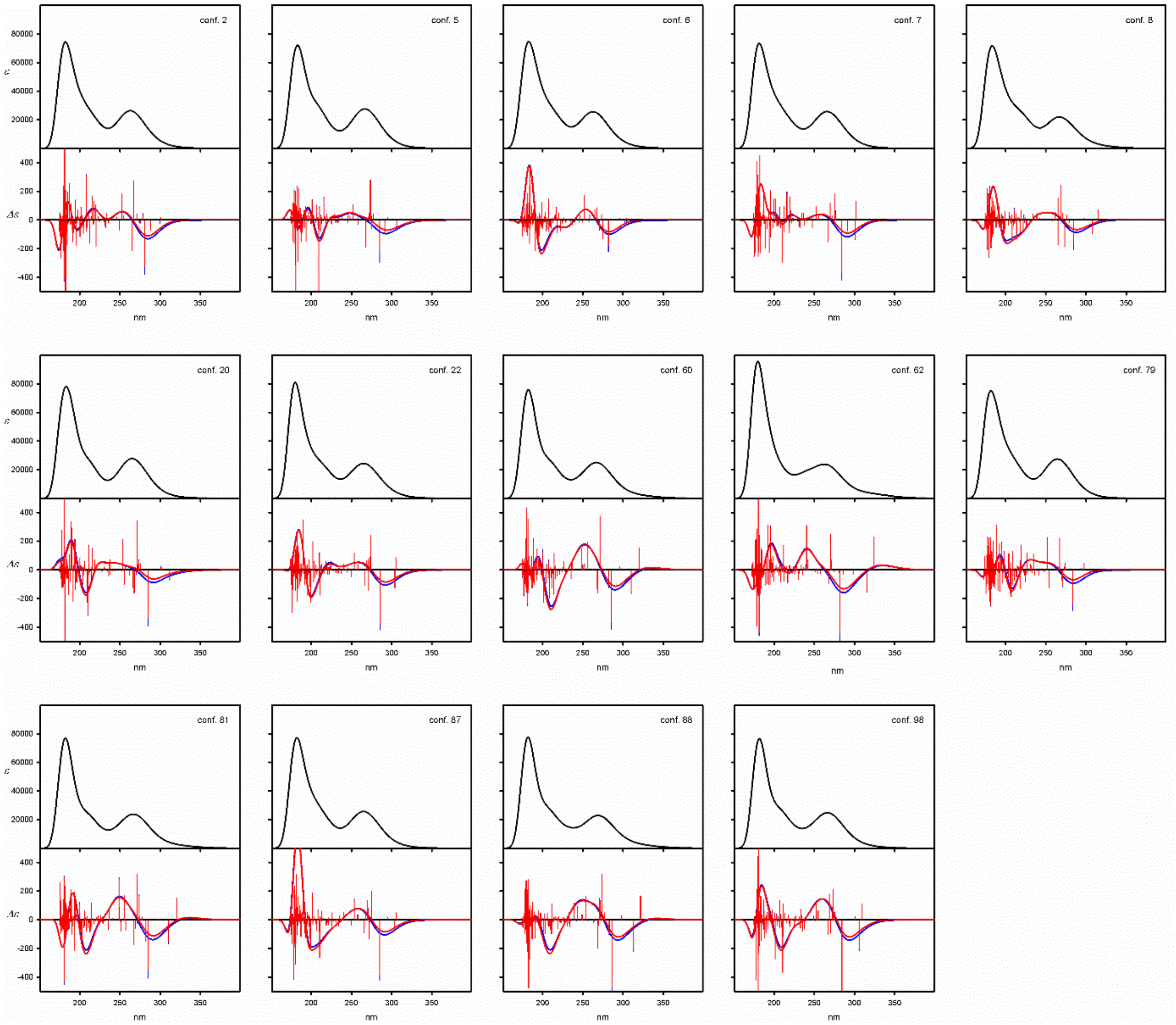

Figure S121. UV (upper panels) and ECD (lower panels) spectra calculated at the TD-CAM-B3LYP/6-311G(d,p) level for individual, non-symmetrical low-energy conformers of **6h**. Wavelengths were not corrected. Geometries were optimized at the M06L/6-311G(d,p) level. $\Delta\varepsilon$ values are given in mol^{-1} cm^{-1} dm^{3}.

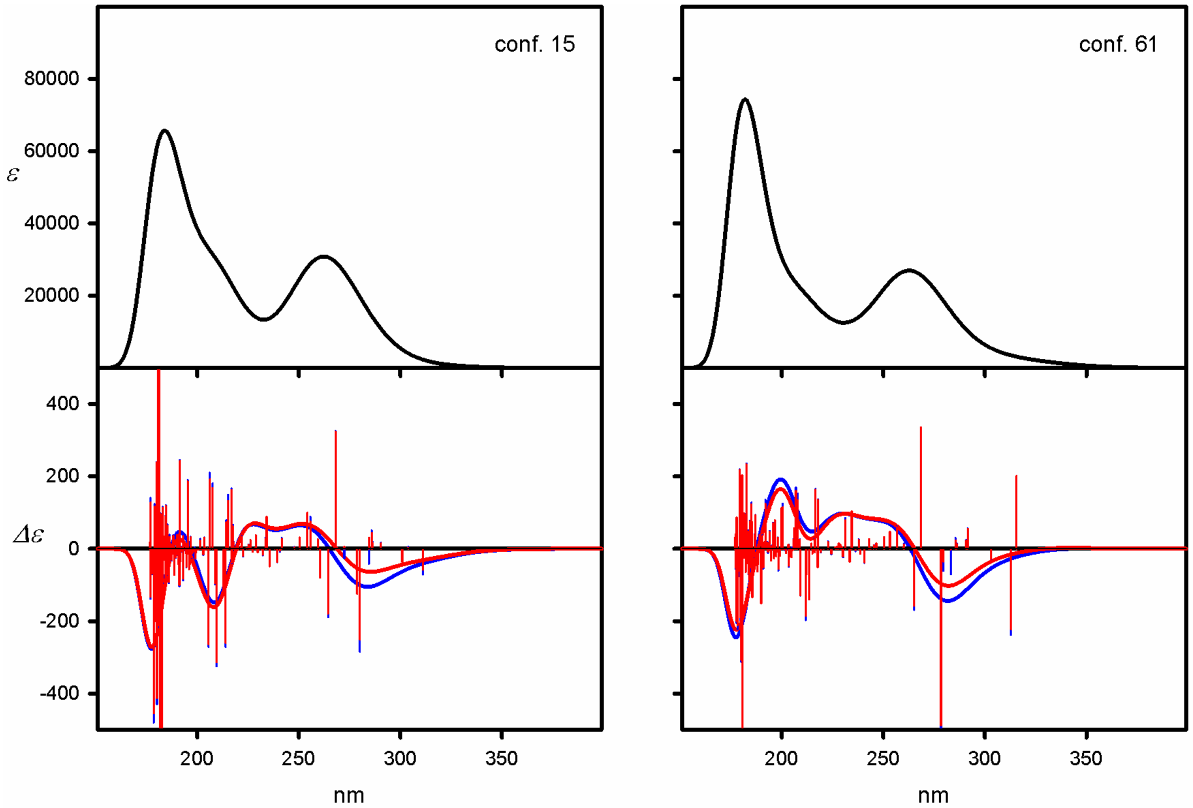

Figure S122. UV (upper panels) and ECD (lower panels) spectra calculated at the TD-M06-2X/6-311G(d,p) level for individual, symmetrical low-energy conformers of **6h**. Wavelengths were not corrected. Geometries were optimized at the M06L/6-311G(d,p) level. *Δε* values are given in mol^{-1} cm^{-1} dm^{3}.

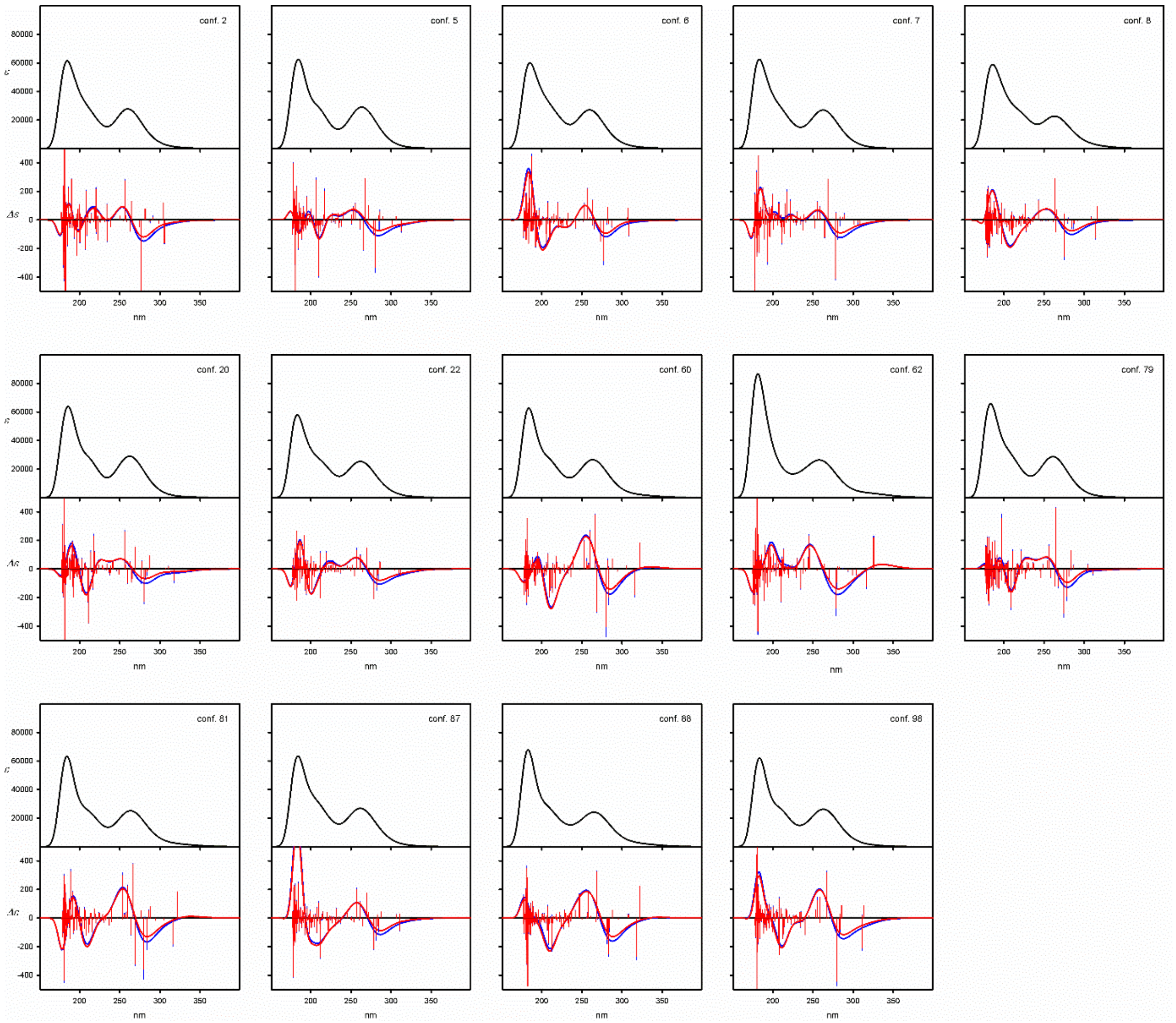

Figure S123. UV (upper panels) and ECD (lower panels) spectra calculated at the TD-M06-2X/6-311G(d,p) level for individual, non-symmetrical low-energy conformers of **6h**. Wavelengths were not corrected. Geometries were optimized at the M06L/6-311G(d,p) level. $\Delta\varepsilon$ values are given in $mol^{-1}\ cm^{-1}\ dm^{3}$.

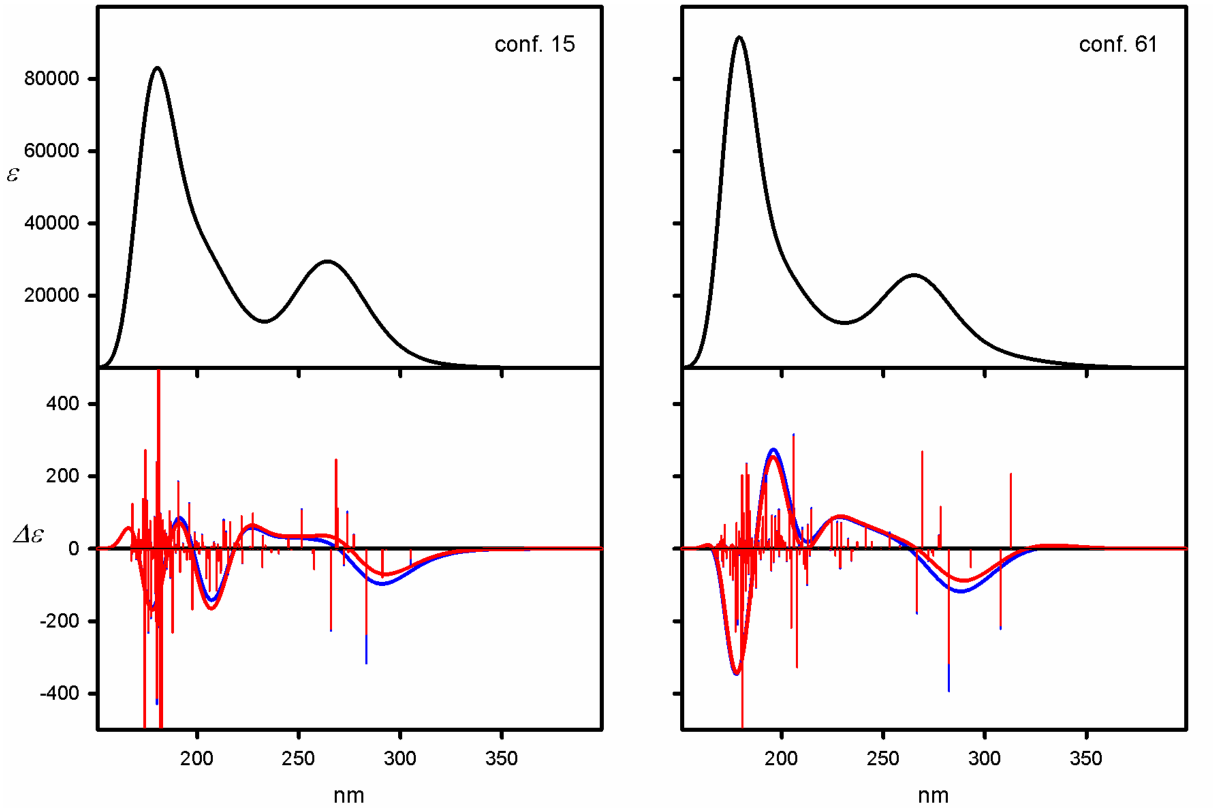

Figure S124. UV (upper panels) and ECD (lower panels) spectra calculated at the TD-wB97XD/6-311G(d,p) level for individual, symmetrical low-energy conformers of **6h**. Wavelengths were not corrected. Geometries were optimized at the M06L/6-311G(d,p) level. $\Delta\varepsilon$ values are given in mol^{-1} cm^{-1} dm^{3}.

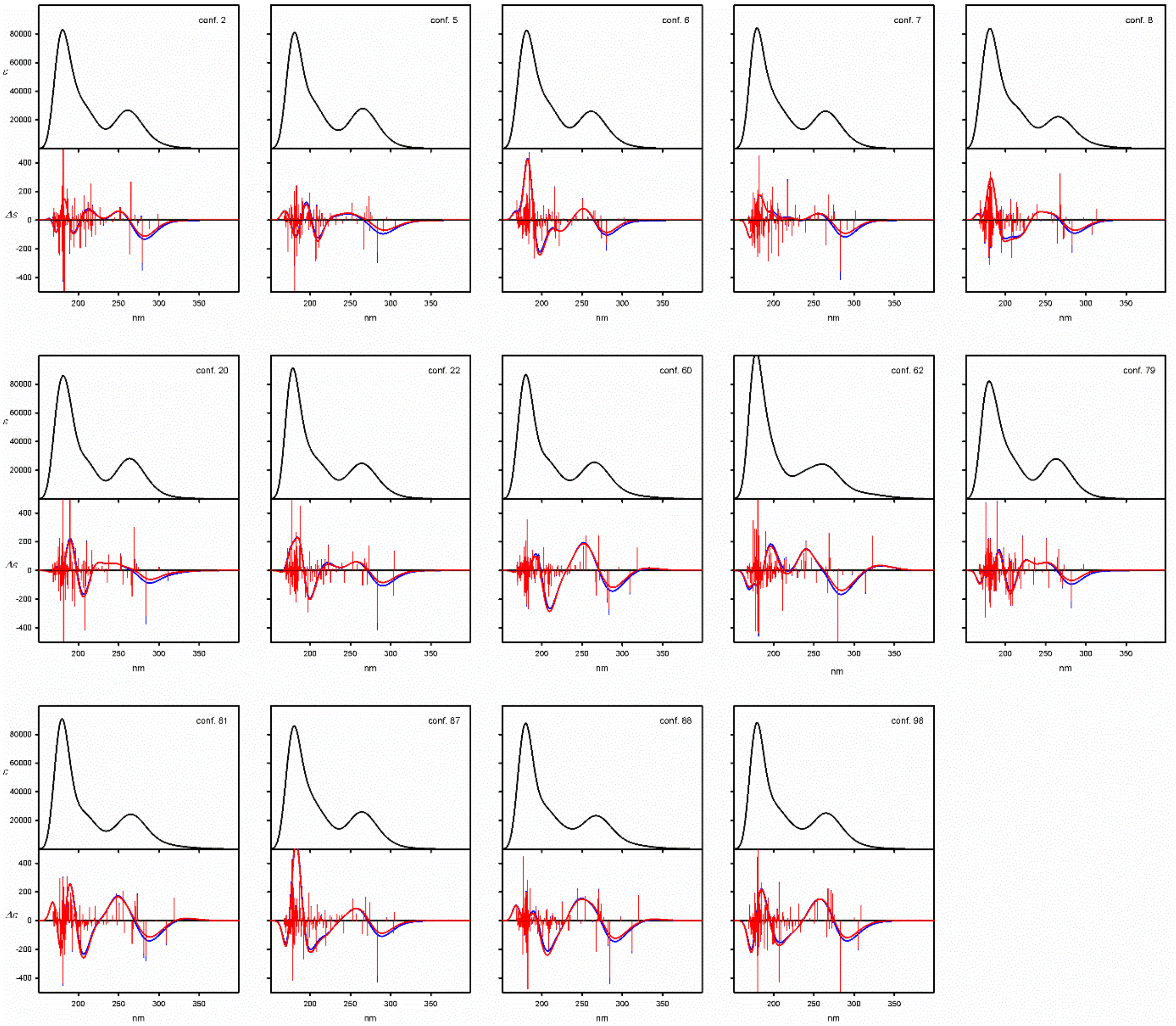

Figure S125. UV (upper panels) and ECD (lower panels) spectra calculated at the TD-wB97XD/6-311G(d,p) level for individual, non-symmetrical low-energy conformers of **6h**. Wavelengths were not corrected. Geometries were optimized at the M06L/6-311G(d,p) level. $\Delta\varepsilon$ values are given in mol^{-1} cm^{-1} dm^{3}.

4 Single crystals X-ray analysis

All crystals subjected to X-ray analysis were mounted on loops by crystal protection grease. Reflection intensities for all samples were measured on an Oxford Diffraction SuperNova Atlas diffractometer equipped with a Cu *Kα* radiation source (λ = 1.54184 Å) and an Atlas CCD detector. In all experiments, the diffraction data were collected at 130 K and the temperature was controlled with an Oxford Instruments Cryosystem cold nitrogen-gas blower. Data collection, reduction and analysis were carried out with CrysAlisPro software.[14] All crystal structures were solved by direct methods using SHELXT-2018 program,[15] and refined by full matrix least squares method on F2 using SHELXL-2018 program.[16] Non-hydrogen atoms were refined using anisotropic thermal parameters. Hydrogen atoms bonded to carbon atoms were placed in idealized positions and refined using the riding model, and their isotropic displacement parameters were set equal to 1.2Ueq(C).
Absolute structures of the compounds were specified by the synthetic procedure – from the known absolute configuration of *trans*-(*R*,*R*)-1,2-diaminocyclohexane, which was used as a starting material in the syntheses; and for measurements with a Cu *Kα* radiation source also confirmed using Flack parameter.[17] Graphical images were prepared using Olex2,[18] and Mercury programs.[19] Crystallographic data and refinement details are collected in **Table S17**. Selected geometrical parameters are juxtaposed in **Table S18**.

Analyzed crystal of 6f was twinned and modeled with BASF parameter refined to 0.4212(9). In crystal **6f** solvent molecules have been identified on subsequent difference electron density maps but their disorder has not been precisely modelled. Instead, the electron density corresponding to these included solvent molecules was taken into account using the solvent mask procedure as implemented in Olex2 software.[16] A solvent mask was calculated and 256 electrons were found in a Volume of 1930 Å^3 in 1 void per unit cell. This is consistent with the presence of 1 hexane, 1 dichloromethane, 3 water molecules per asymmetric unit, which account for 244 electrons per unit cell. In macrocycle 6f, one phenyl ring was modelled for disorder with the site occupation factors 0.55 and 0.45.

The crystals structure of **6g** contains a some amount of disordered solvent caged in intermolecular hole. A solvent mask was calculated and 46 electrons were found in a volume of 203 Å^3 in 1 void per unit cell. This is consistent with the presence of diethyl ether ($C_4H_{10}O$) per asymmetric unit which account for 42 electrons per unit cell. The solvent mask procedure, implemented in Olex2 software was included in structure refinement. Additionally, the molecule of **6g** is disordered. In crystal structure symmetry of macrocyclic molecule is C_1 with one of the three bromine atoms on the opposite side of the ring plane. However, the arrangement of substituent for 20% of the molecules is different and all three bromine atoms are on the same side of the molecule (the refined occupancy factor is 0.194).

CCDC **2394844** (**6b**), **2387654** (**6f**) and **2394845** (**6g**) contain the supplementary crystallographic data for this paper. These data can be obtained free of charge from The Cambridge Crystallographic Data Centre via www.ccdc.cam.ac.uk/data%5Frequest/cif.

Table S17. Selected crystal data and structure refinement details.

	**6b**	**6f**	**6g**
CCDC number	**2394844**	**2387654**	**2394845**
Chemical formula	$C_{90}H_{96}N_6S_6$	$C_{156}H_{132}N_6S_6 \cdot C_6H_{14} \cdot CH_2Cl_2 \cdot 3H_2O$	$C_{63}H_{63}Br_3N_6S_3$
M_r	1454.08	2508.17	1240.10
Crystal system, space group	Monoclinic, $P2_1$	Monoclinic, $P2_1$	Triclinic, $P1$
Temperature (K)	130	130	130
a, *b*, *c* (Å)	12.56390 (11), 17.58334 (11), 18.66386 (19)	20.1919 (1), 17.5652 (1), 20.9011 (1)	9.7084 (3), 12.0742 (4), 14.9689 (4)
α, β, γ (°)	103.5053 (9)	90.865 (1)	92.032 (3), 102.902 (3), 107.401 (3)
V (Å^3)	4009.12 (6)	7412.25 (7)	1622.20 (10)
Z	2	2	1
Radiation type	Cu $K\alpha$		
μ (mm^{-1})	1.95	1.62	3.52
Crystal size (mm)	0.2 × 0.1 × 0.03	0.9 × 0.3 × 0.1	0.1 × 0.05 × 0.01
T_{min}, T_{max}	0.803, 1.000	0.269, 0.269	0.921, 1.000
No. of measured, independent and observed [$I > 2\sigma(I)$] reflections	49709, 16379, 15519	80527, 80527, 76491	24968, 11767, 10295
R_{int}	0.029	?	0.038
$(\sin \theta/\lambda)_{max}$ (Å^{-1})	0.631	0.629	0.631
$R[F^2 > 2\sigma(F^2)]$, $wR(F^2)$, S	0.035, 0.091, 1.02	0.067, 0.189, 1.05	0.046, 0.115, 1.03
No. of reflections	16379	80527	11767
No. of parameters	1257	1508	698
No. of restraints	55	109	211
$\Delta\rangle_{max}$, $\Delta\rangle_{min}$ (e Å^{-3})	0.28, -0.32	0.37, -0.63	0.48, -0.52
Absolute structure parameter	-0.005 (4)	0.056 (7)	-0.028 (13)

Table S18. The helicity of trityl groups in molecule **6f** in the crystal structure. The conformation of each phenyl ring in a given trityl group was defined by its helicity which can be either M (90 < ϕ < 0), P (0 < ϕ < 90) or 0 (for ϕ angles deviating from planarity by ± 5).

	φ **(°)**	**helicity**
**S1-Tr**	-23.2(5)	
	34.8(5)	*MPP*
	74.0(5)	
**S2-Tr**	83.5(4)	
	6.5(5)	*PPP*
	43.2(4)	
**S3-Tr**	81.7(5)	
	11.6(9) / 30.6(9)	*PPP*
	37.5(6)	
**S4-Tr**	87.0(4)	
	-9.4(6)	*PMP*
	35.8(5)	
**S5-Tr**	-48.7(5)	
	-58.4(6)	*MMM*
	-6.9(6)	
**S6-Tr**	84.2(4)	
	-3.8(5)	*P0P*
	35.8(5)	

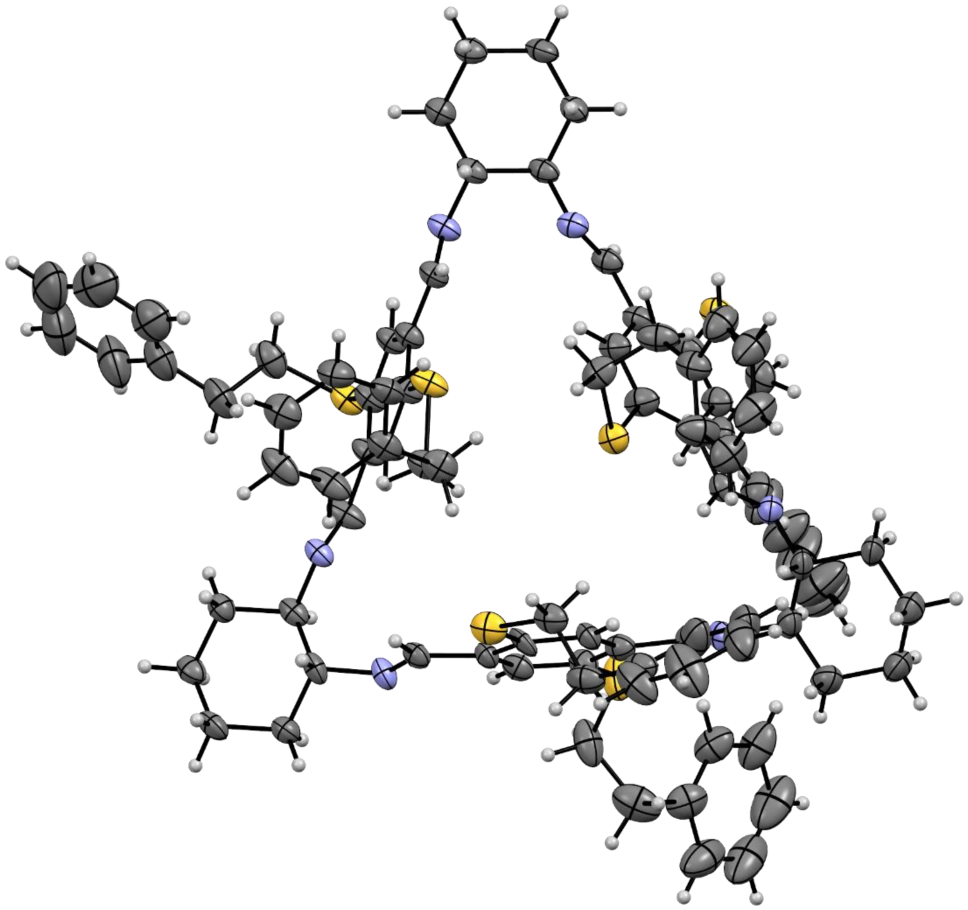

Figure S126. Structure of the macrocyclic molecule **6b**. Displacement ellipsoids are drawn at the 30% probability level. Hydrogen atoms are represented in arbitrary radii.

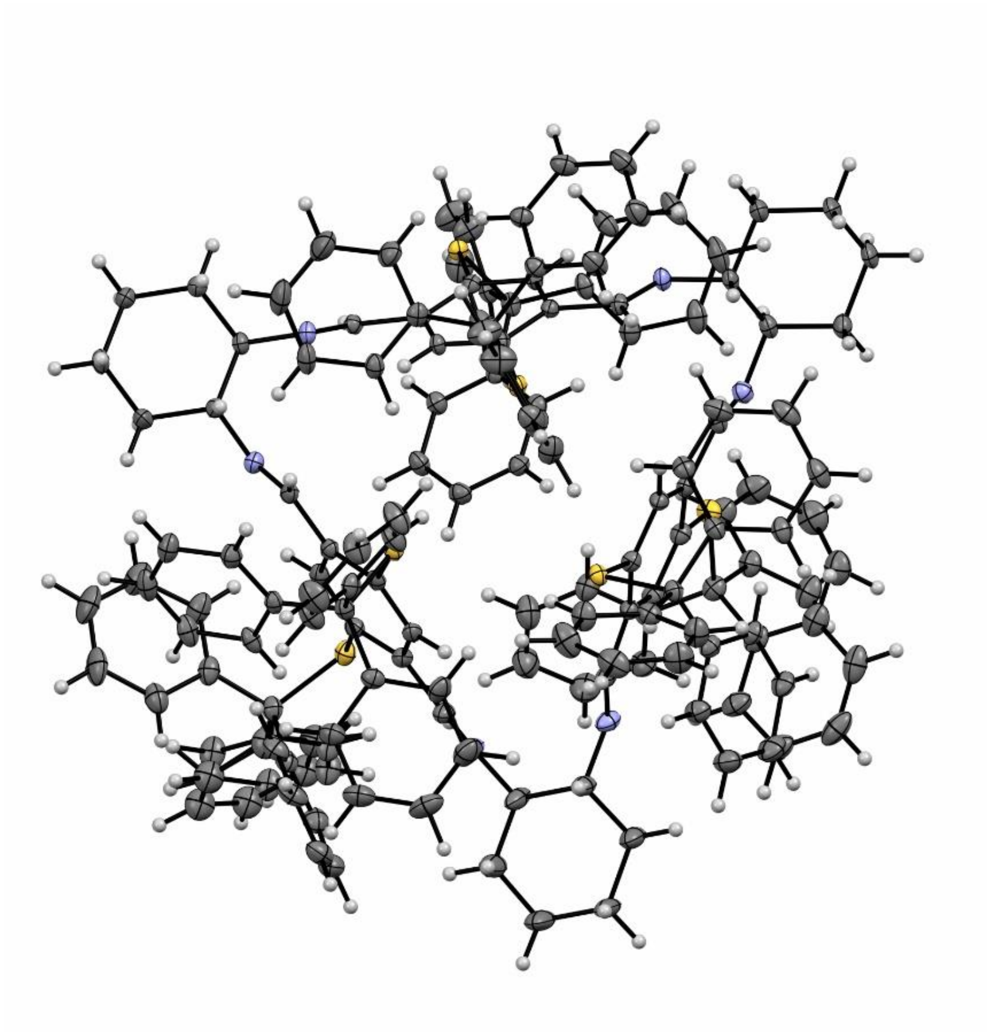

Figure S127. Structure of the macrocyclic molecule **6f**. Displacement ellipsoids are drawn at the 30% probability level. Hydrogen atoms are represented in arbitrary radii.

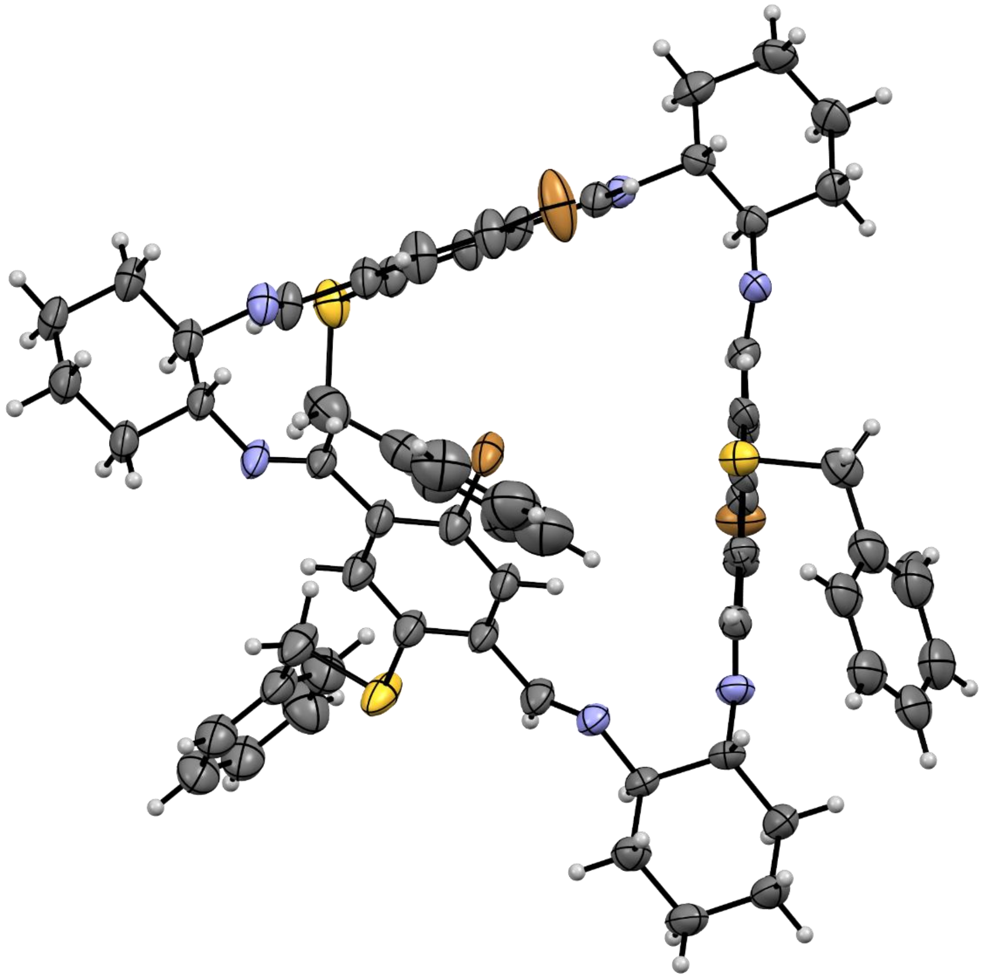

Figure S128. Structure of the macrocyclic molecule **6g**. Displacement ellipsoids are drawn at the 30% probability level. Hydrogen atoms are represented in arbitrary radii.

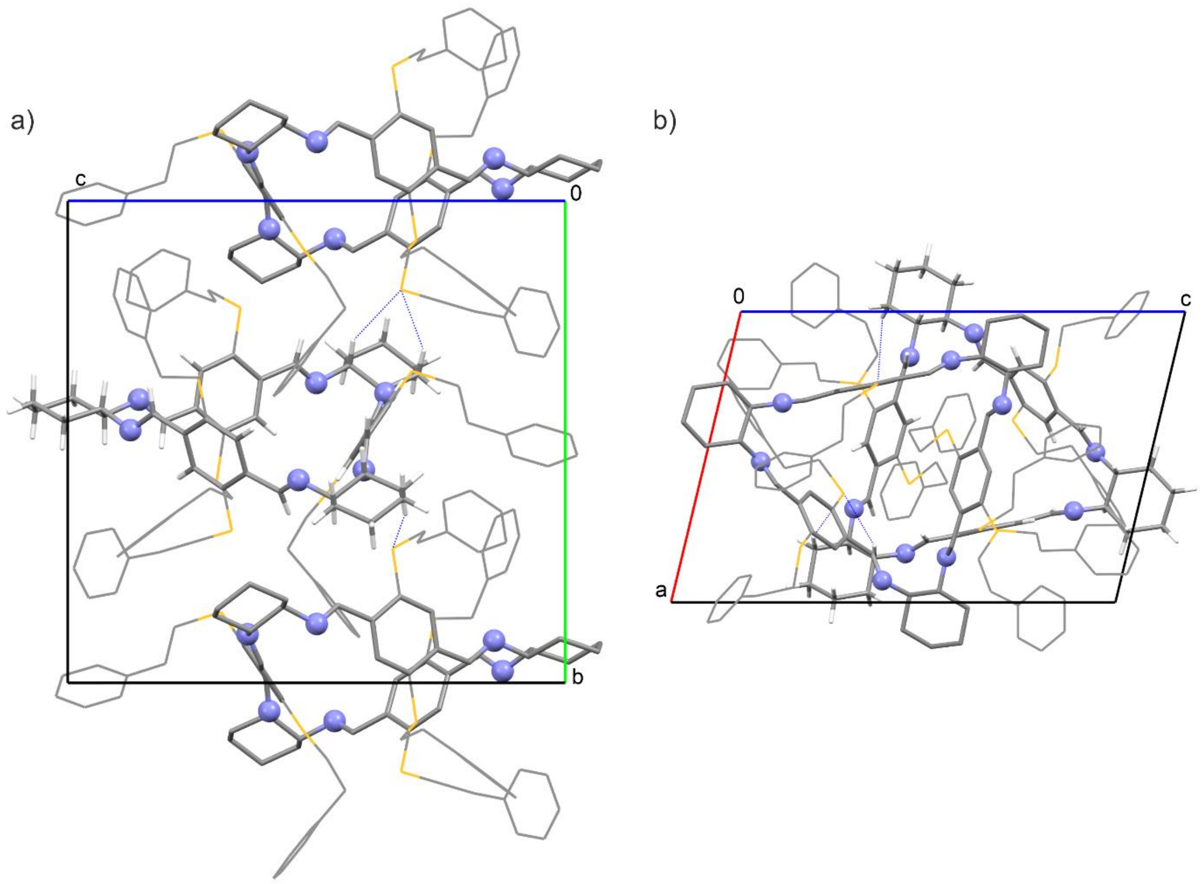

Figure S129. C-H···S interactions (showed as blue dashed lines) in supramolecular columns in crystal of **6b**: a) side view and b) top view. For clarity some hydrogen atoms are omitted.

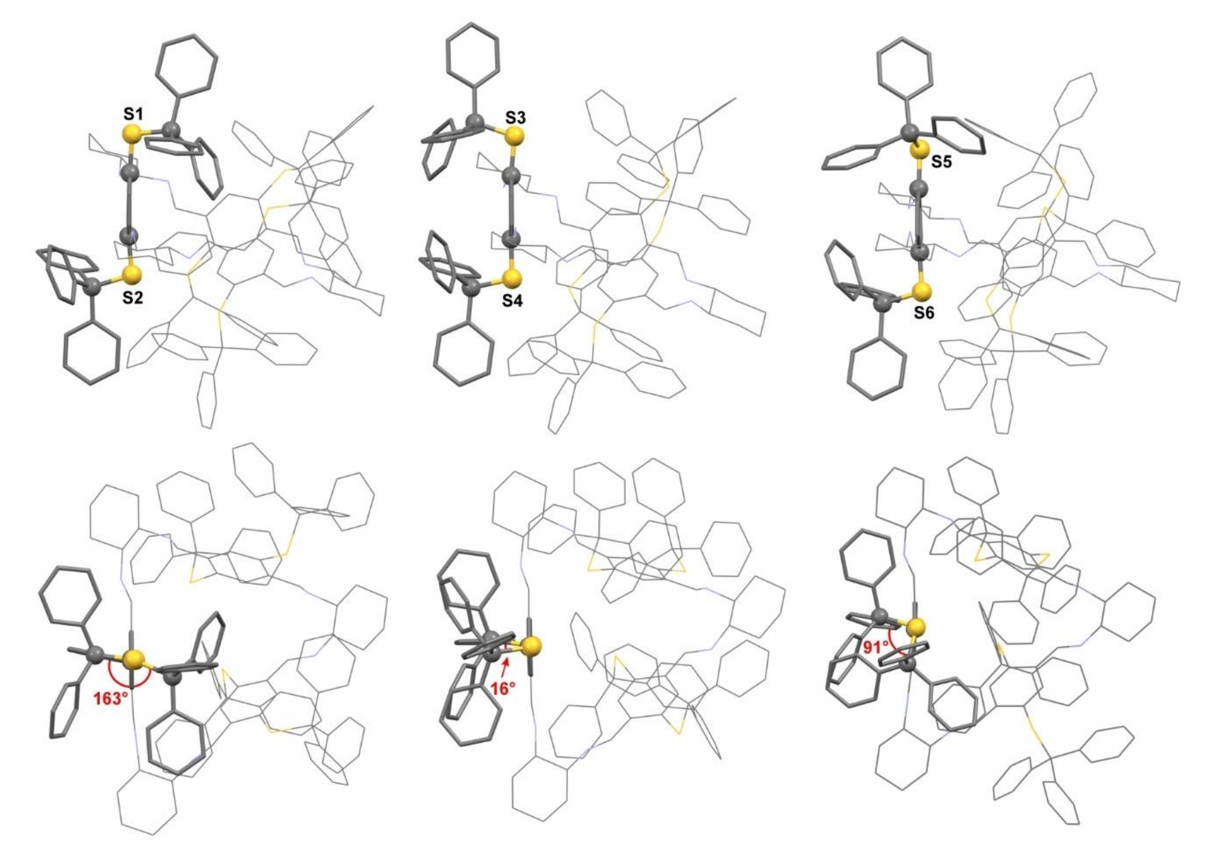

Figure S130. Arrangement of aromatic linkers with anchored –STr groups in the molecule **6f** in crystal. The *pseudo*torsion angles values, C(Tr)-S-(Ar)-S-C(Tr), are highlighted in red.

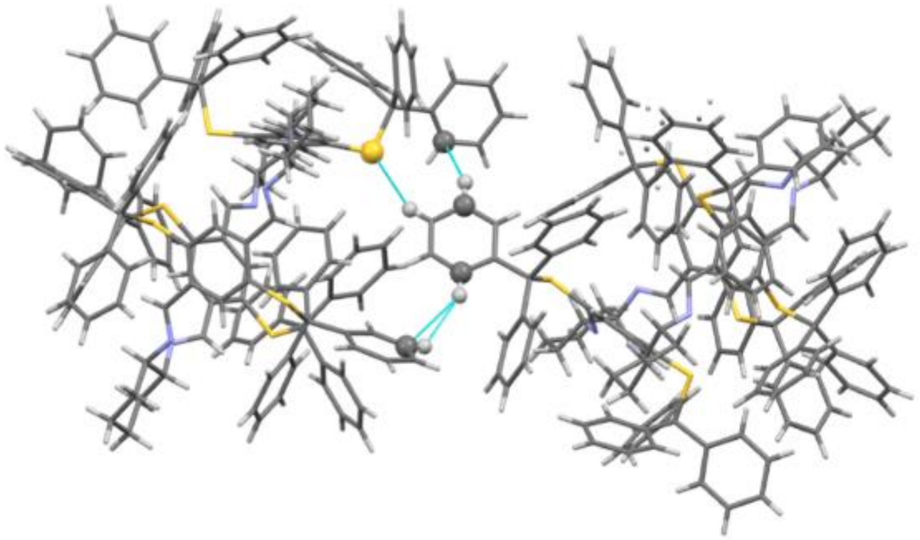

Figure S131. Supramolecular architecture in **6f** crystals created between macrocycles. Selected intermolecular interactions are indicated in blue dash lines.

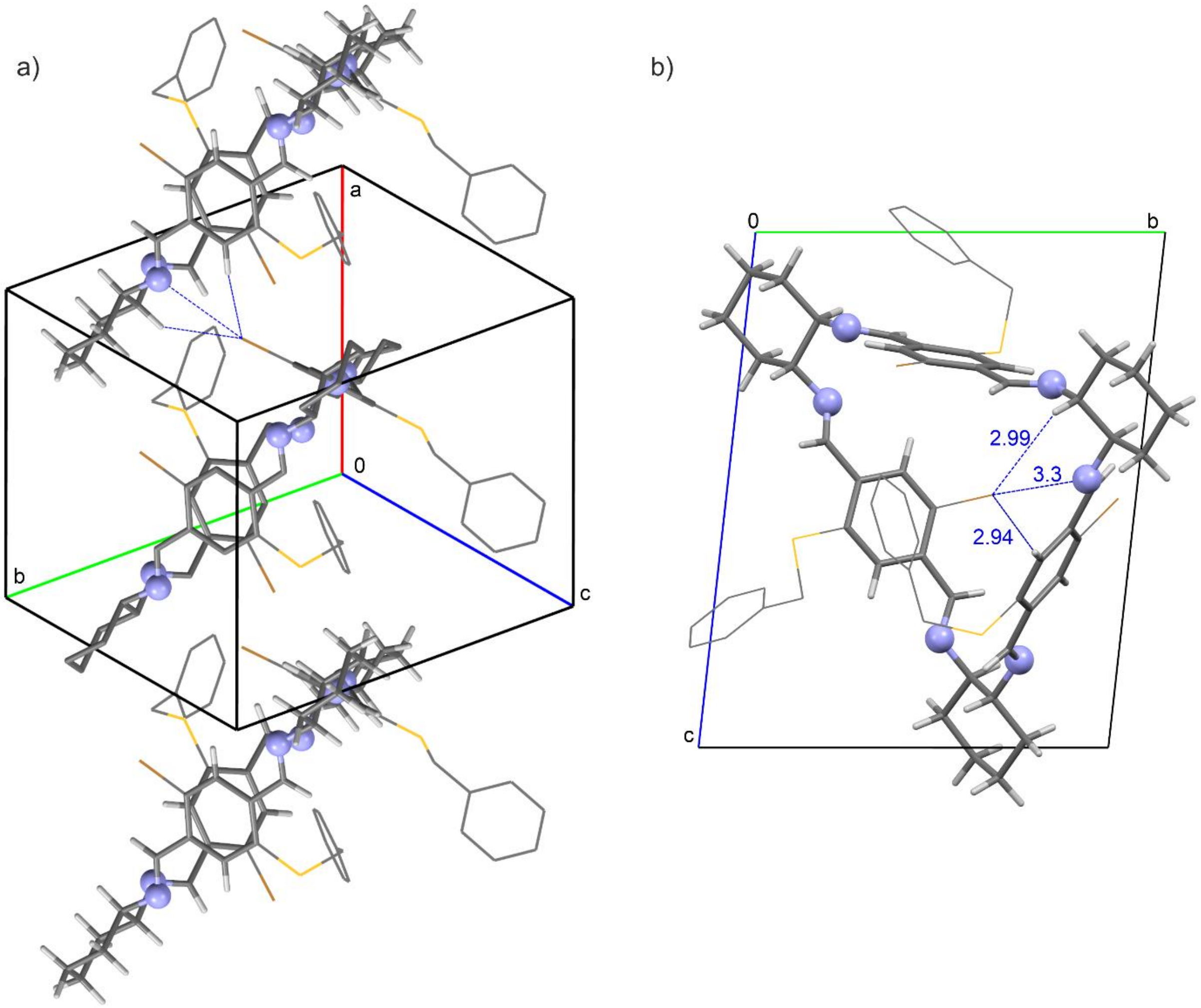

Figure S132. Intermolecular interactions (showed as blue dashed lines) in supramolecular columns in crystal of **6g**: a) side view and b) top view. For clarity some hydrogen atoms are omitted. The distances are given in Å.

5 Copies of ^{1}H and ^{13}C NMR spectra

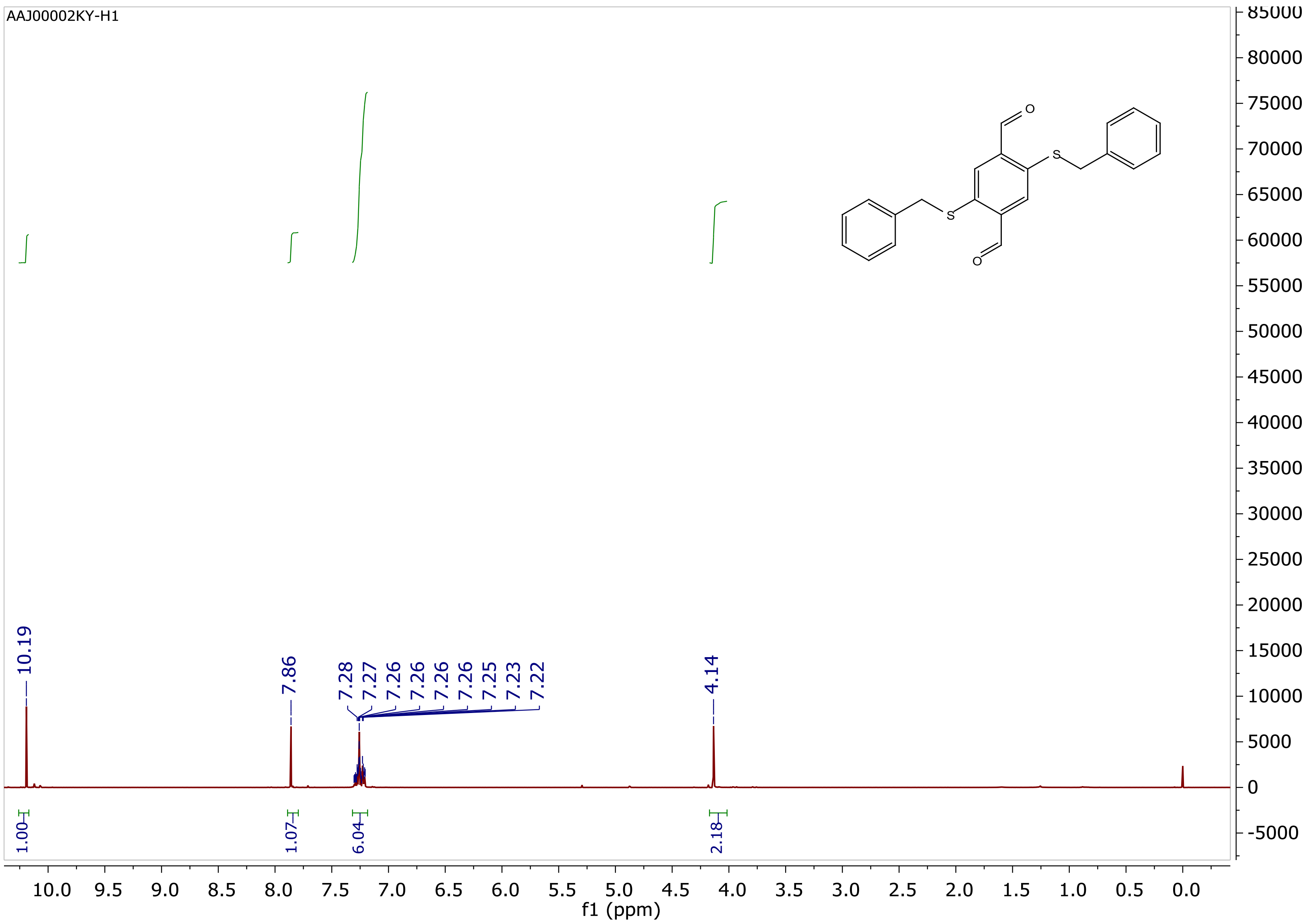

Figure S131. Copy of ^{1}H NMR spectrum ($CDCl_3$, 400 MHz, RT) of **5a**.

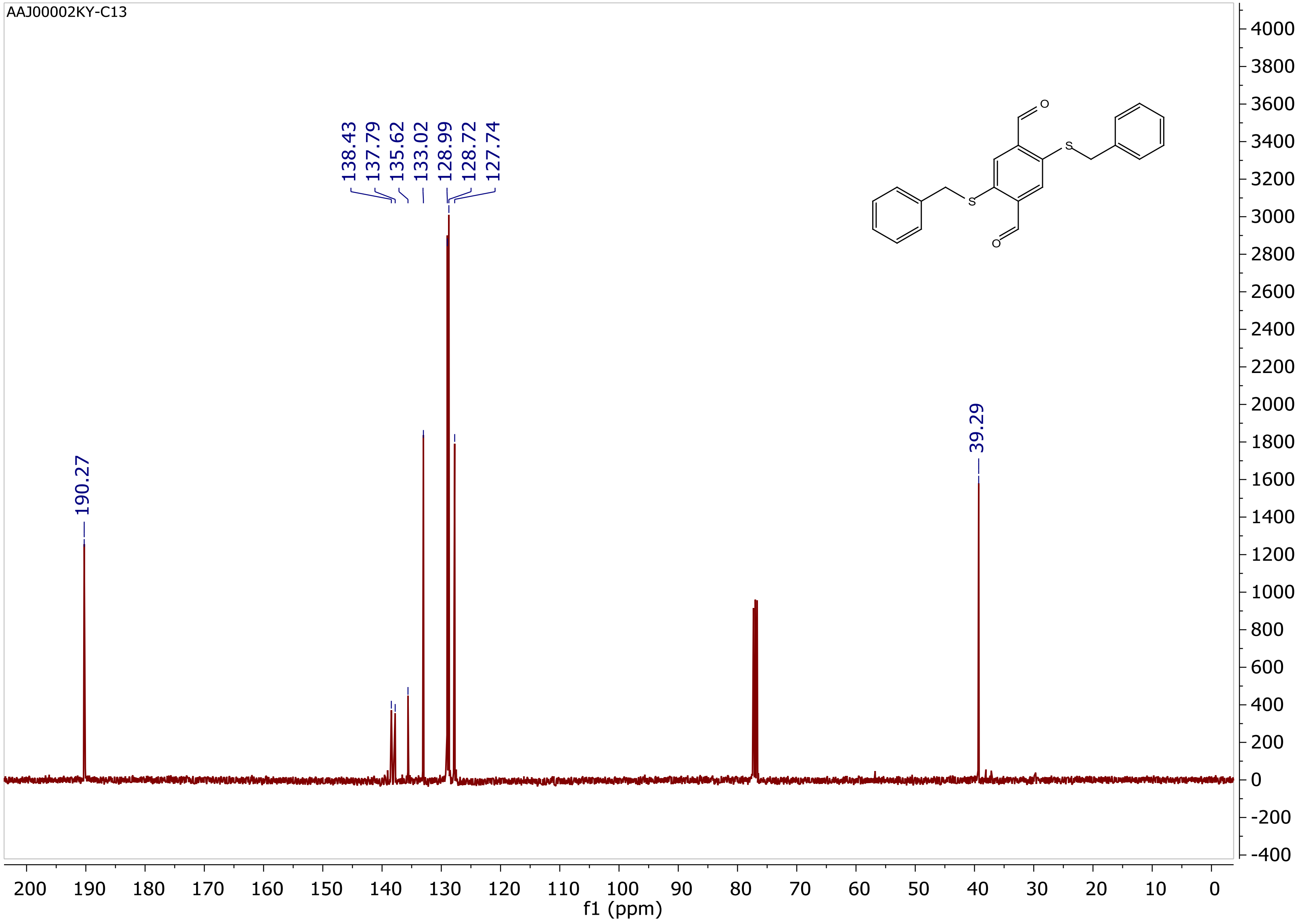

Figure S132. Copy of $^{13}C\{^{1}H\}$ NMR spectrum ($CDCl_3$, 101 MHz, RT) of **5a.**

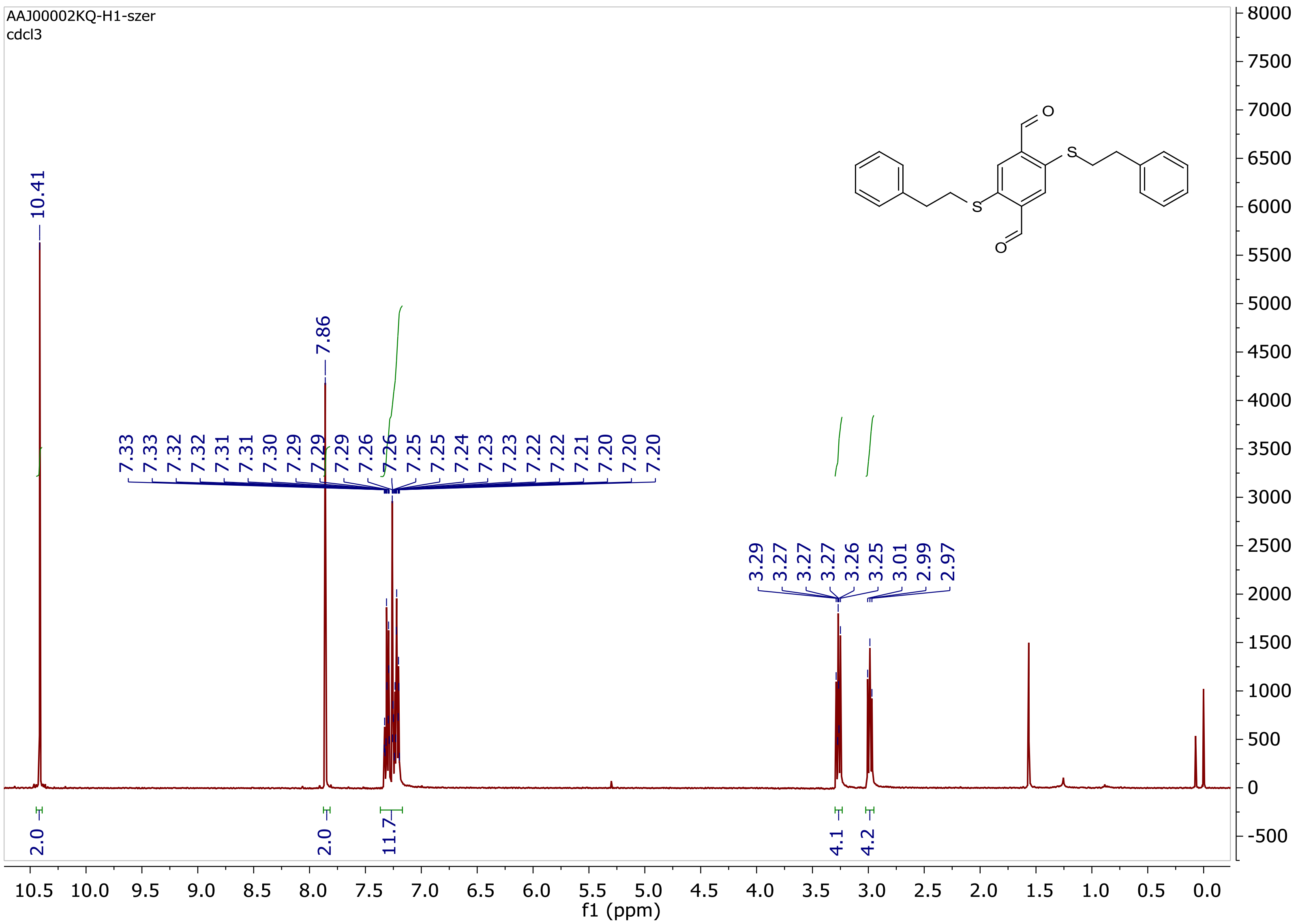

Figure S133. Copy of ^{1}H NMR spectrum ($CDCl_3$, 400 MHz, RT) of **5b**.

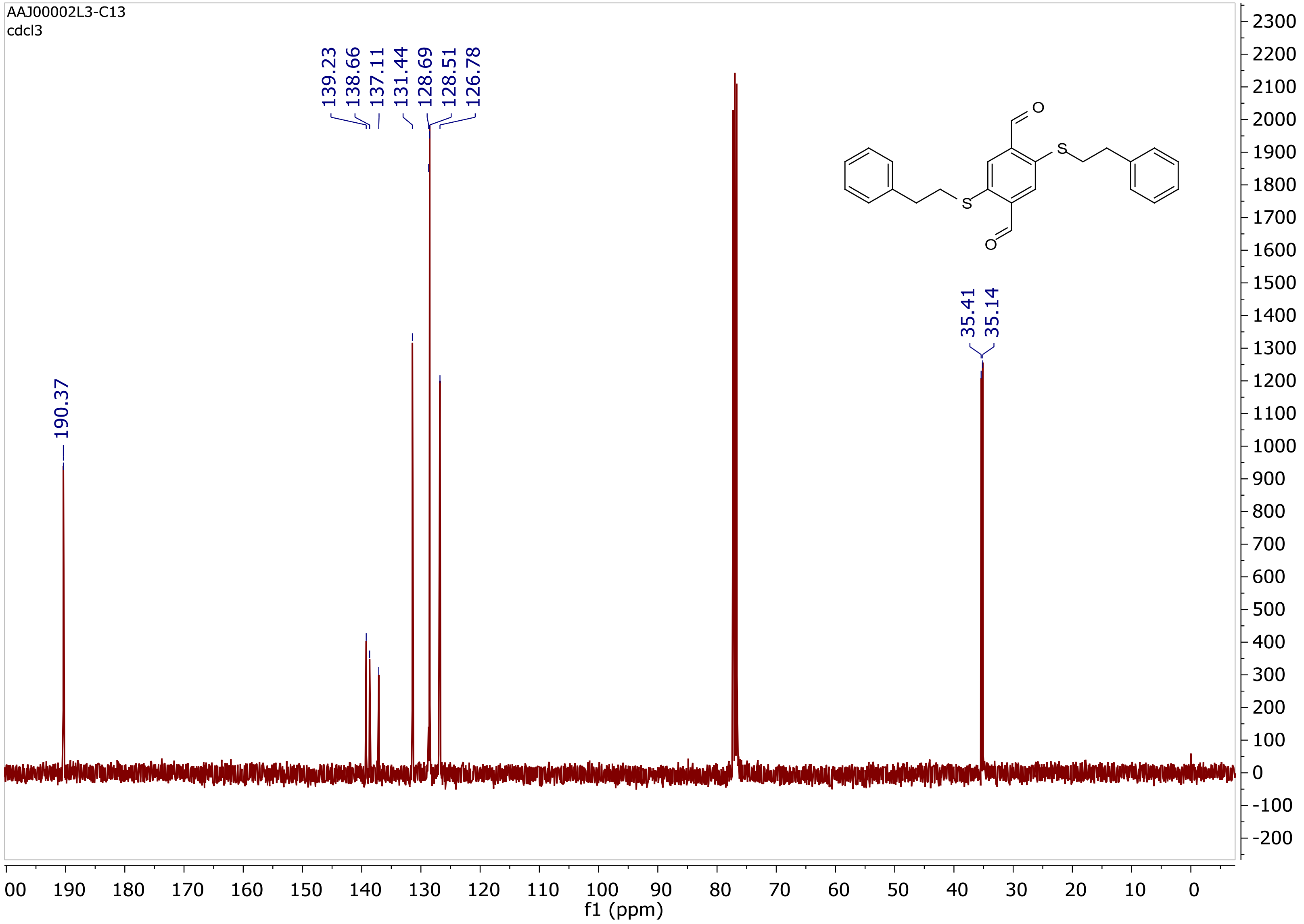

Figure S134. Copy of $^{13}C\{^1H\}$ NMR spectrum ($CDCl_3$, 101 MHz, RT) of **5b.**

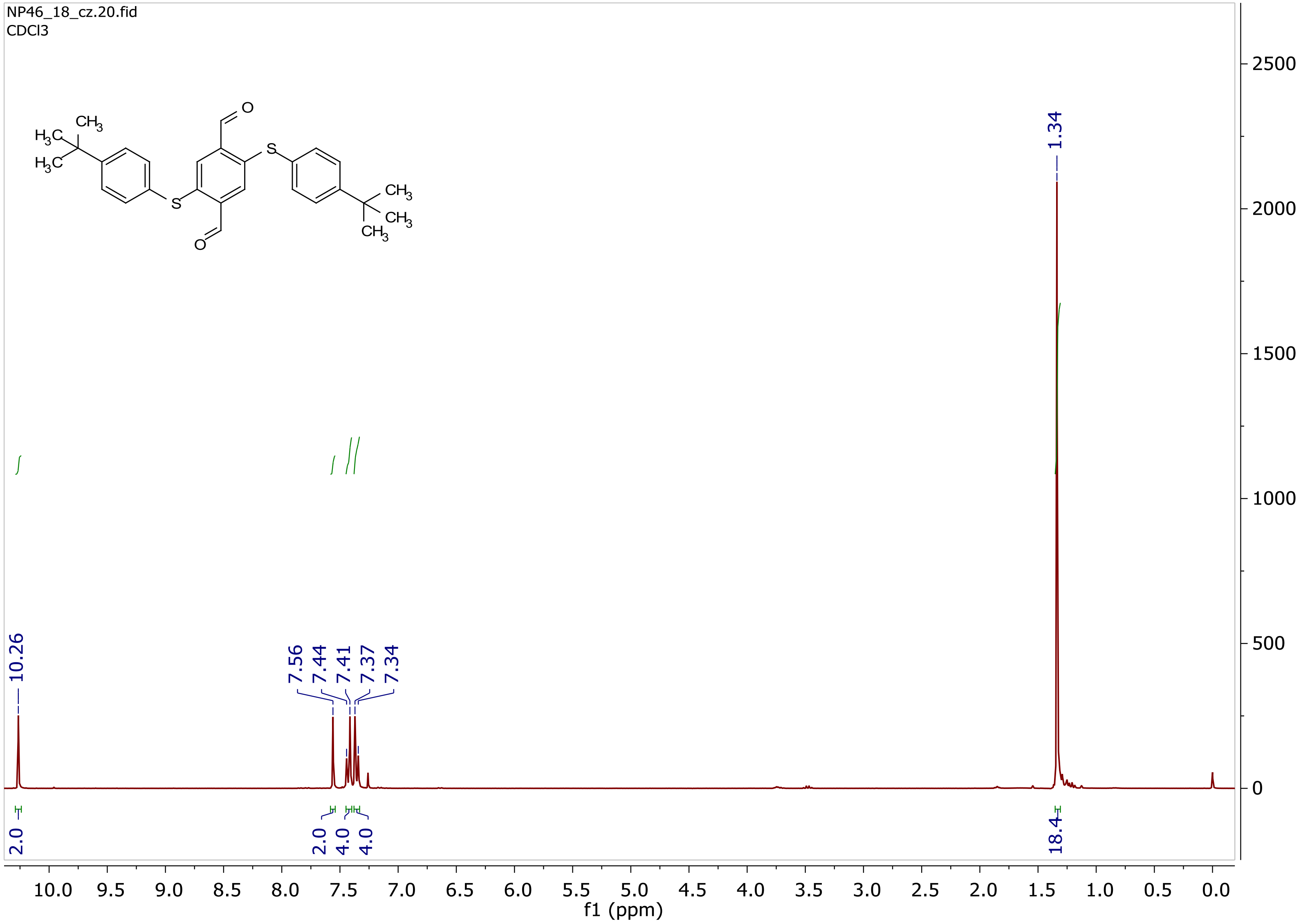

Figure S135. Copy of ^{1}H NMR spectrum ($CDCl_3$, 300 MHz, RT) of **5c**.

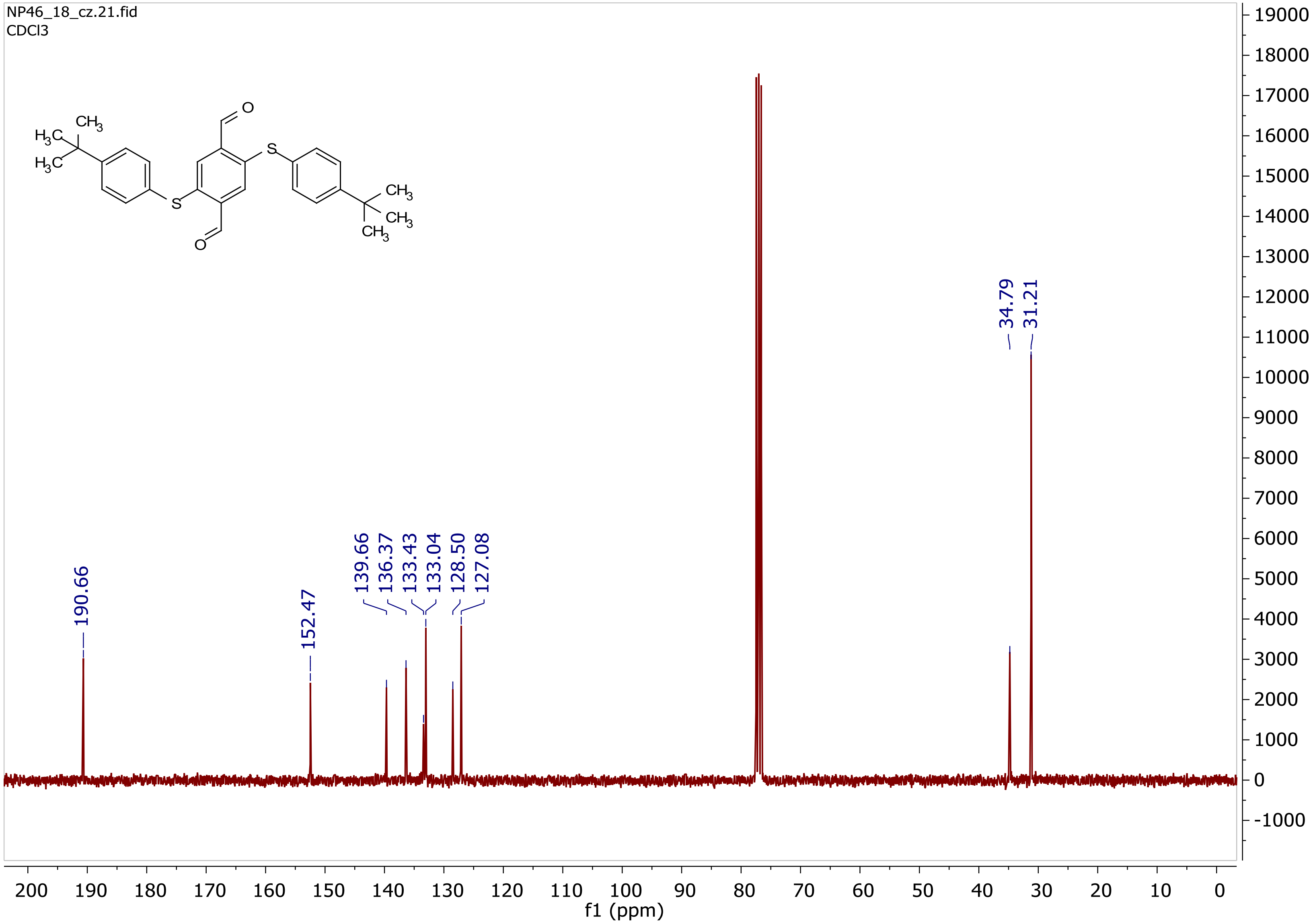

Figure S136. Copy of $^{13}C\{^1H\}$ NMR spectrum ($CDCl_3$, 75 MHz, RT) of **5c.**

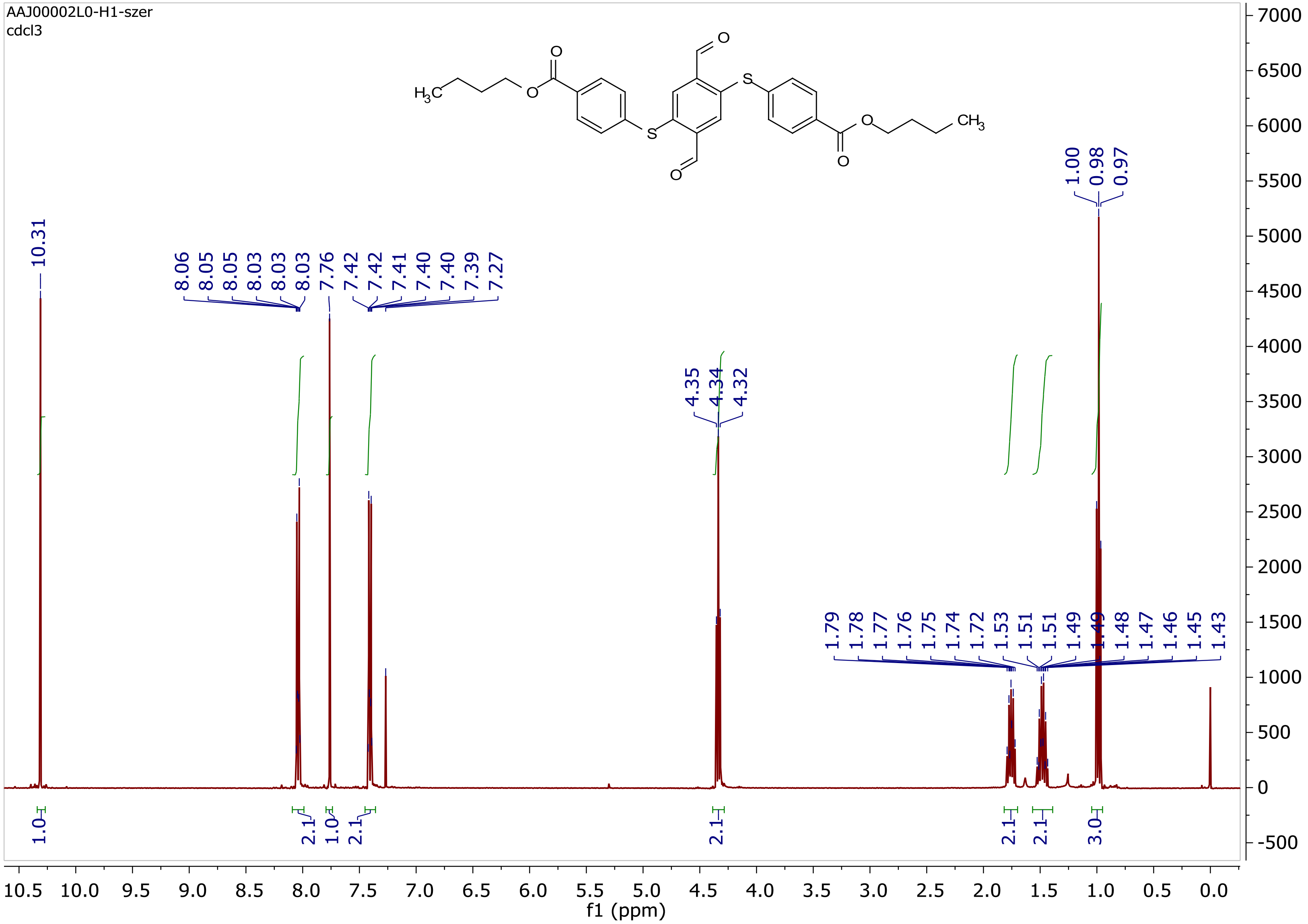

Figure S137. Copy of ^{1}H NMR spectrum ($CDCl_3$, 400 MHz, RT) of **5d**.

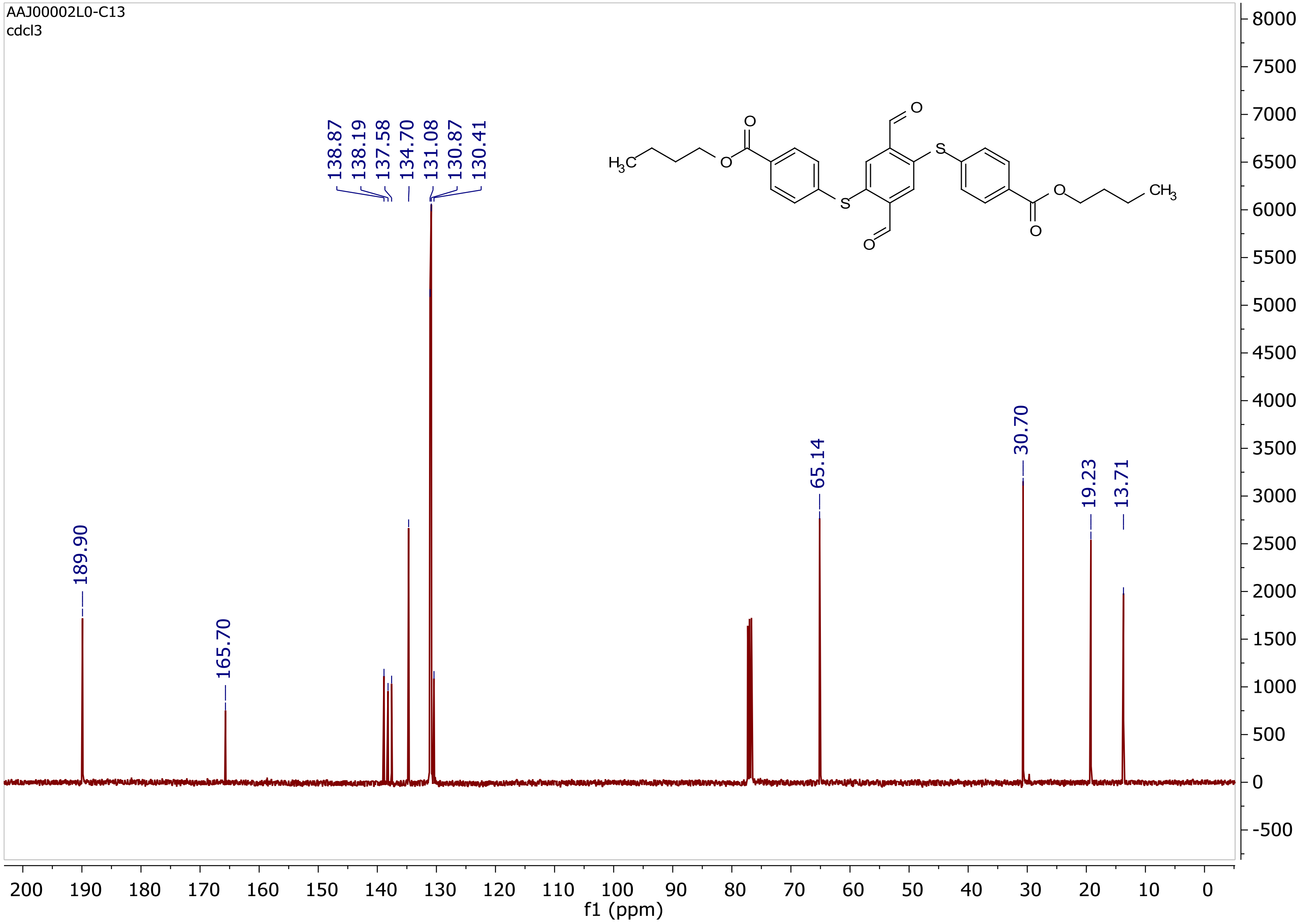

Figure S138. Copy of $^{13}C\{^{1}H\}$ NMR spectrum ($CDCl_3$, 101 MHz, RT) of **5d.**

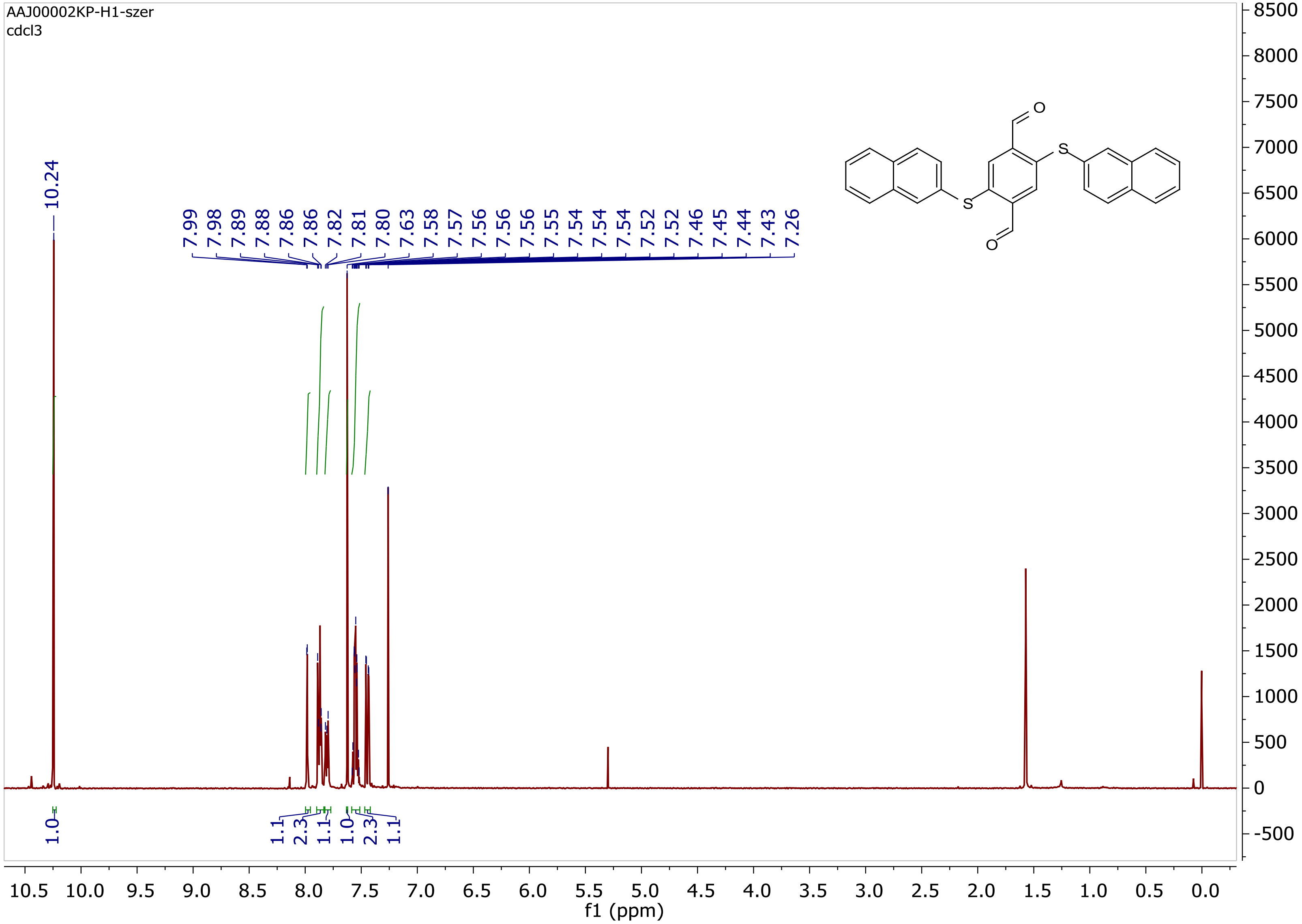

Figure S139. Copy of ^{1}H NMR spectrum ($CDCl_3$, 400 MHz, RT) of **5e**.

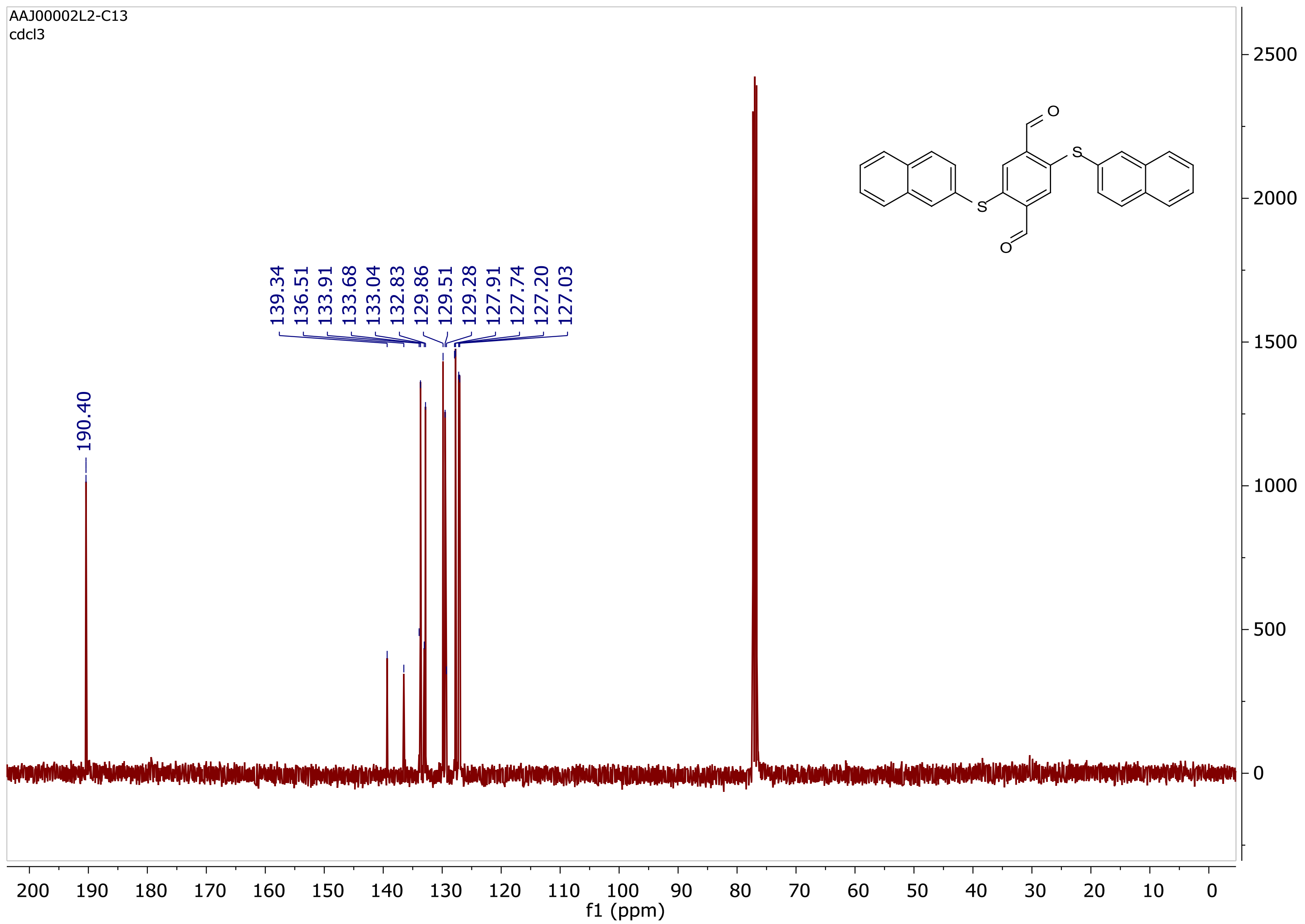

Figure S140. Copy of $^{13}C\{^{1}H\}$ NMR spectrum ($CDCl_3$, 101 MHz, RT) of **5e.**

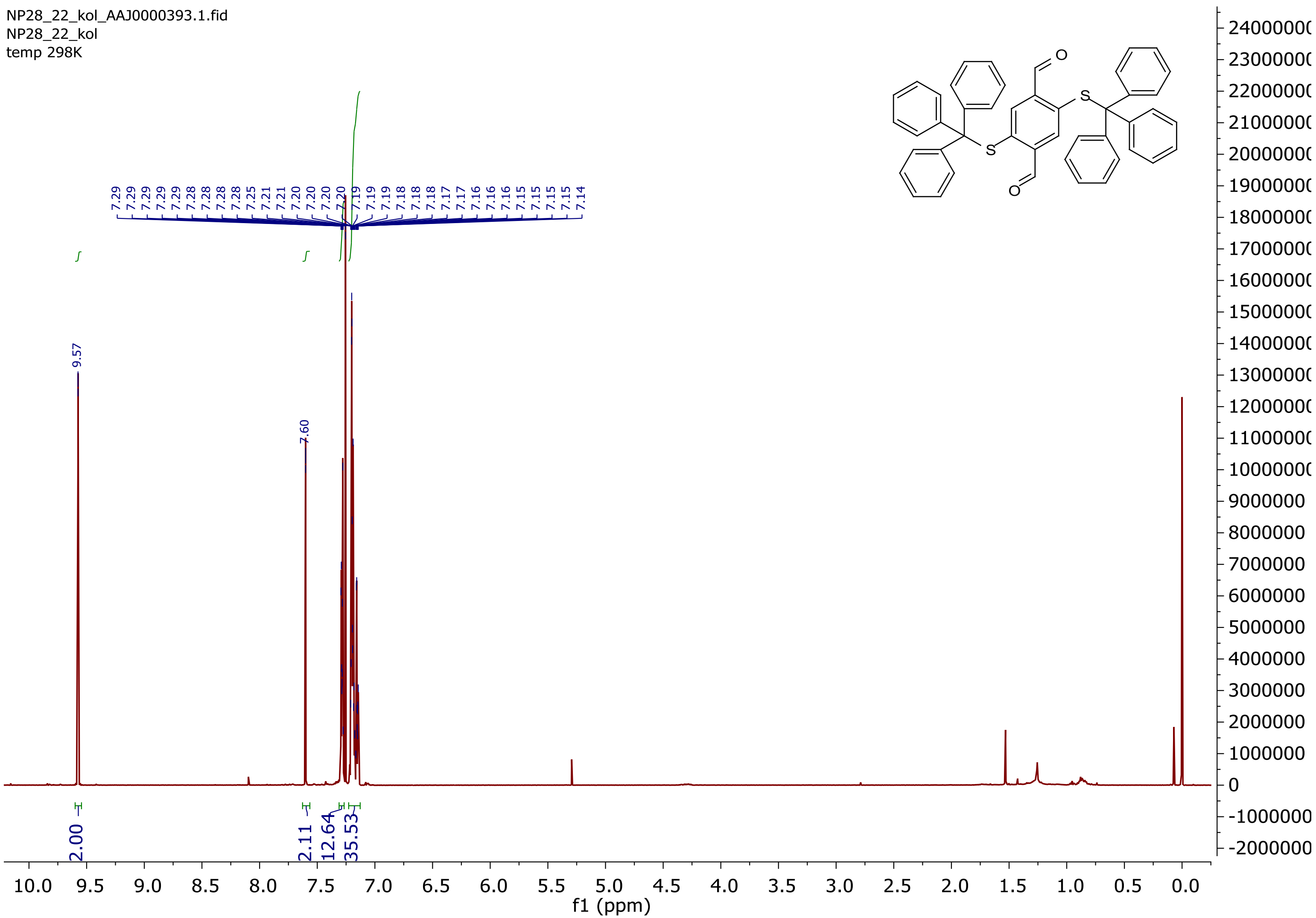

Figure S141. Copy of ^{1}H NMR spectrum ($CDCl_3$, 600 MHz, RT) of **5f**.

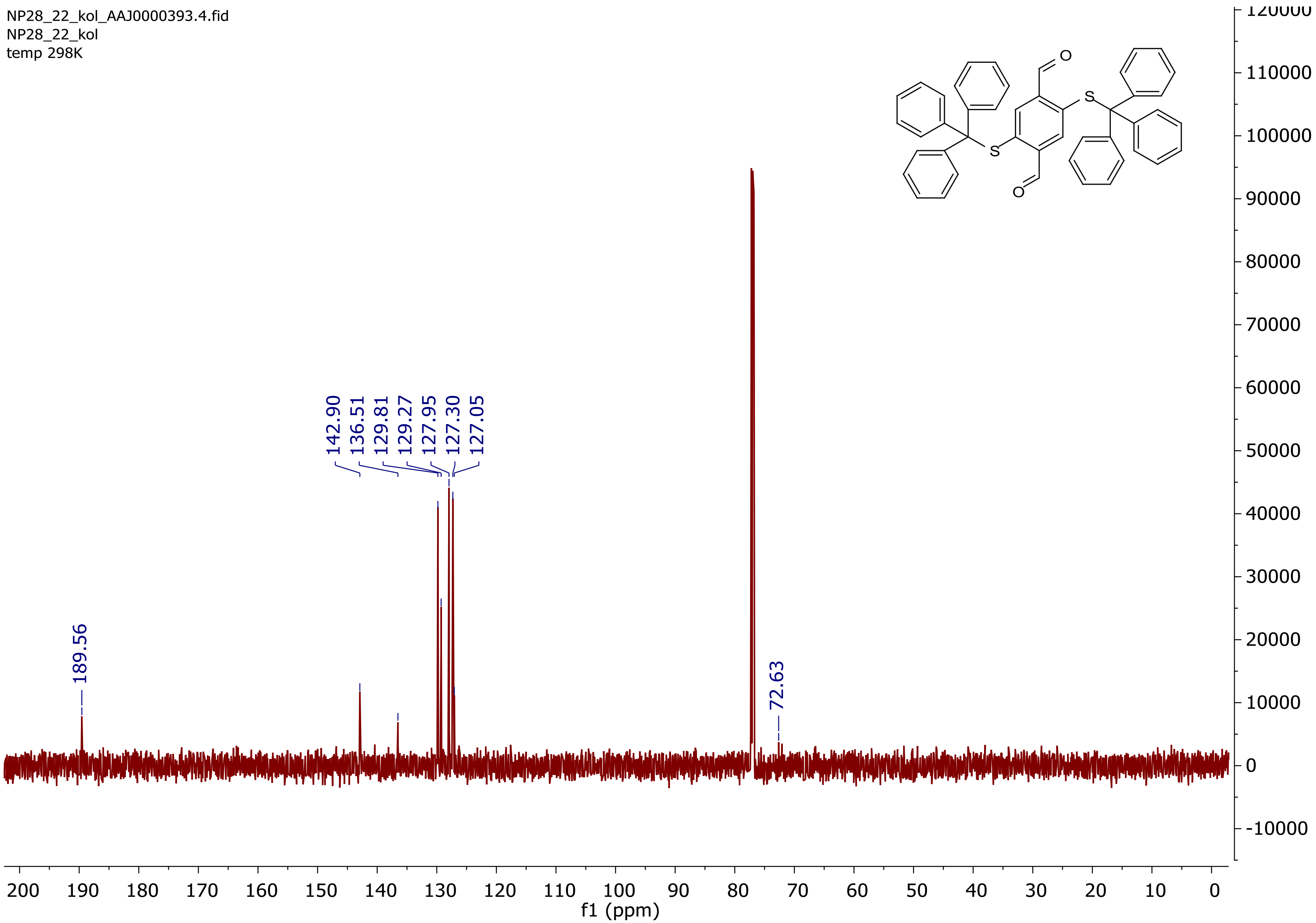

Figure S142. Copy of $^{13}C\{^{1}H\}$ NMR spectrum ($CDCl_3$, 151 MHz, RT) of **5f.**

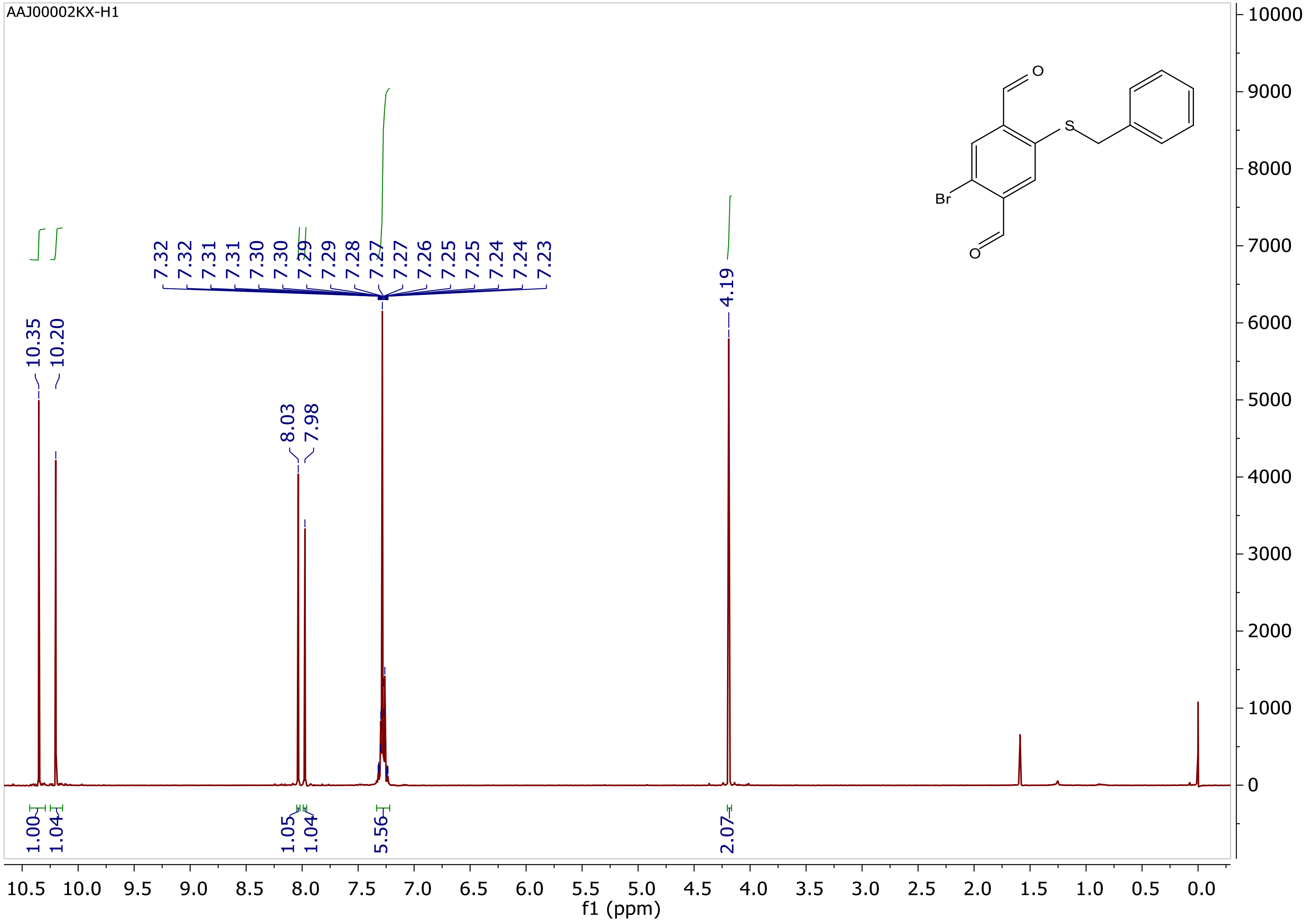

Figure S143. Copy of ^{1}H NMR spectrum ($CDCl_3$, 400 MHz, RT) of **5g**.

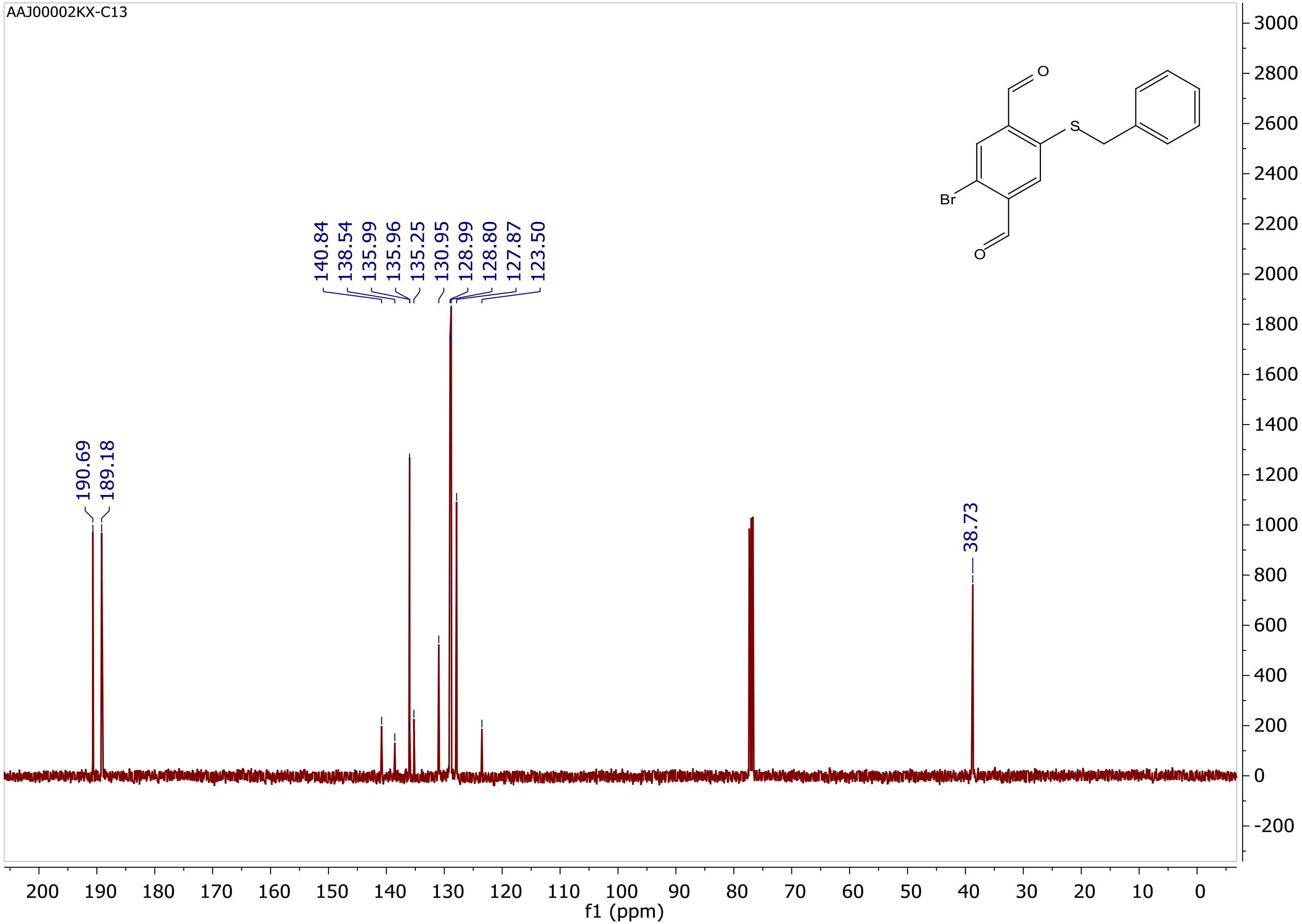

Figure S144. Copy of $^{13}C\{^{1}H\}$ NMR spectrum ($CDCl_3$, 101 MHz, RT) of **5g.**

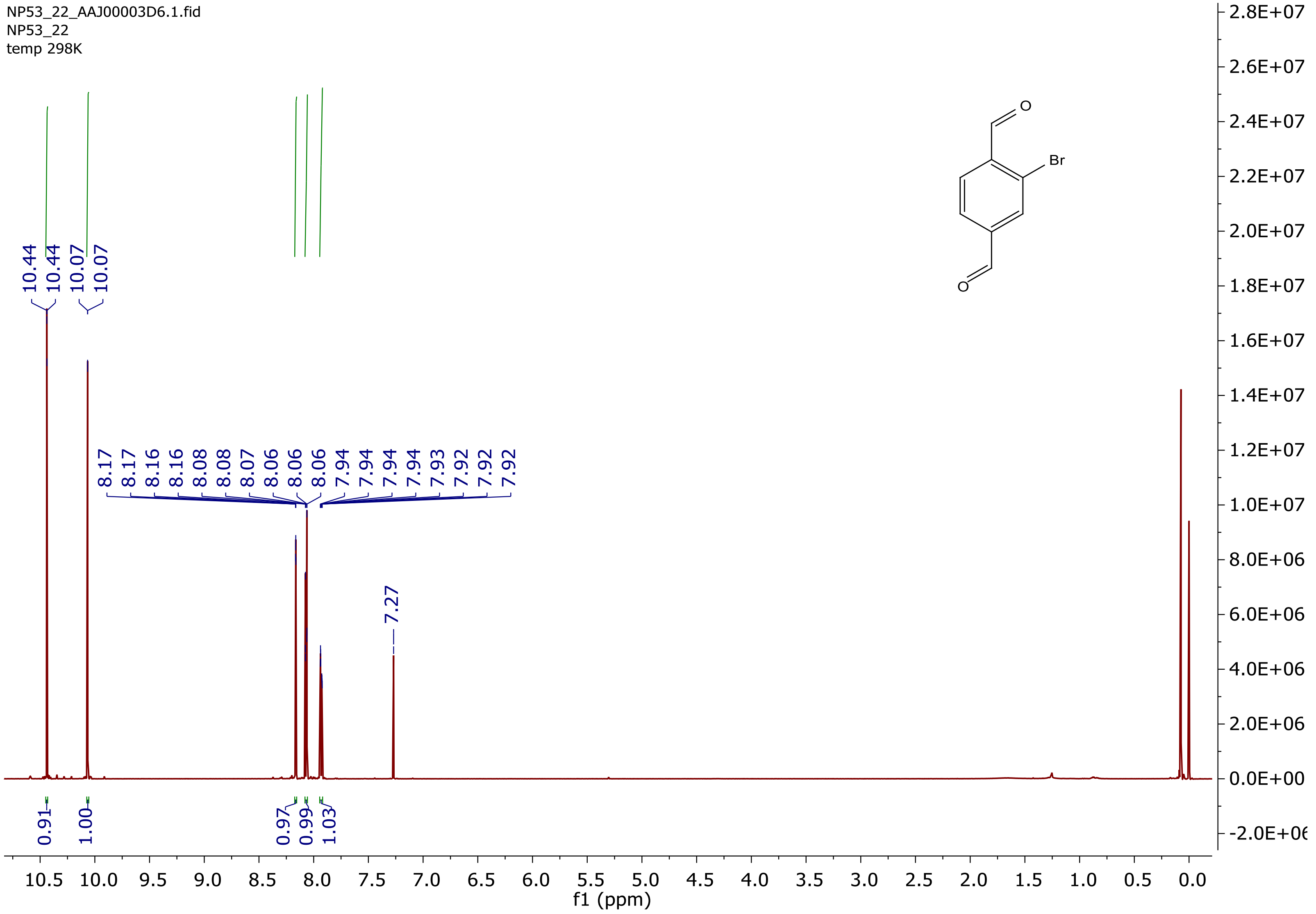

Figure S145. Copy of ^{1}H NMR spectrum ($CDCl_3$, 600 MHz, RT) of **5h**.

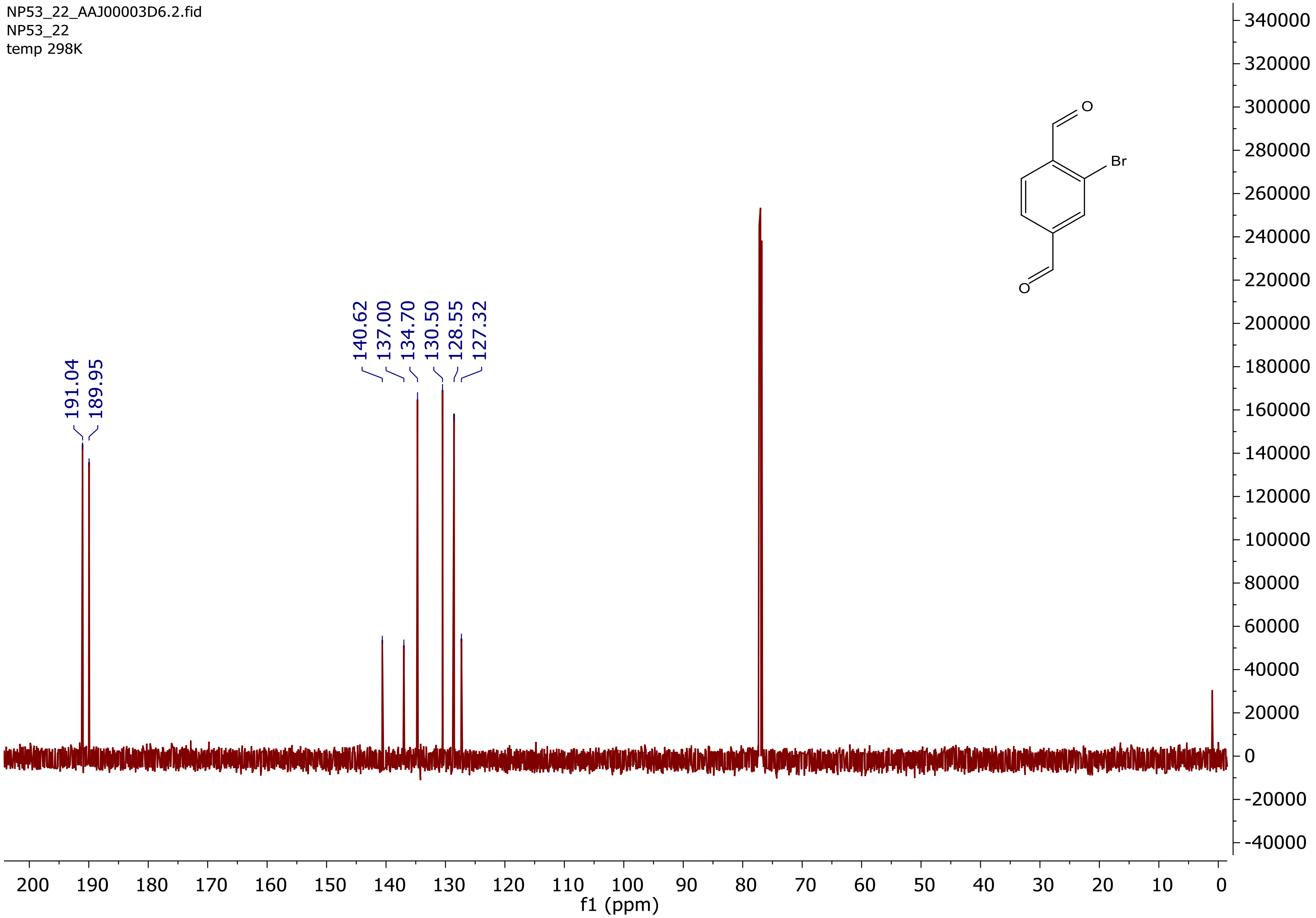

Figure S146. Copy of $^{13}C\{^{1}H\}$ NMR spectrum ($CDCl_3$, 151 MHz, RT) of **5h.**

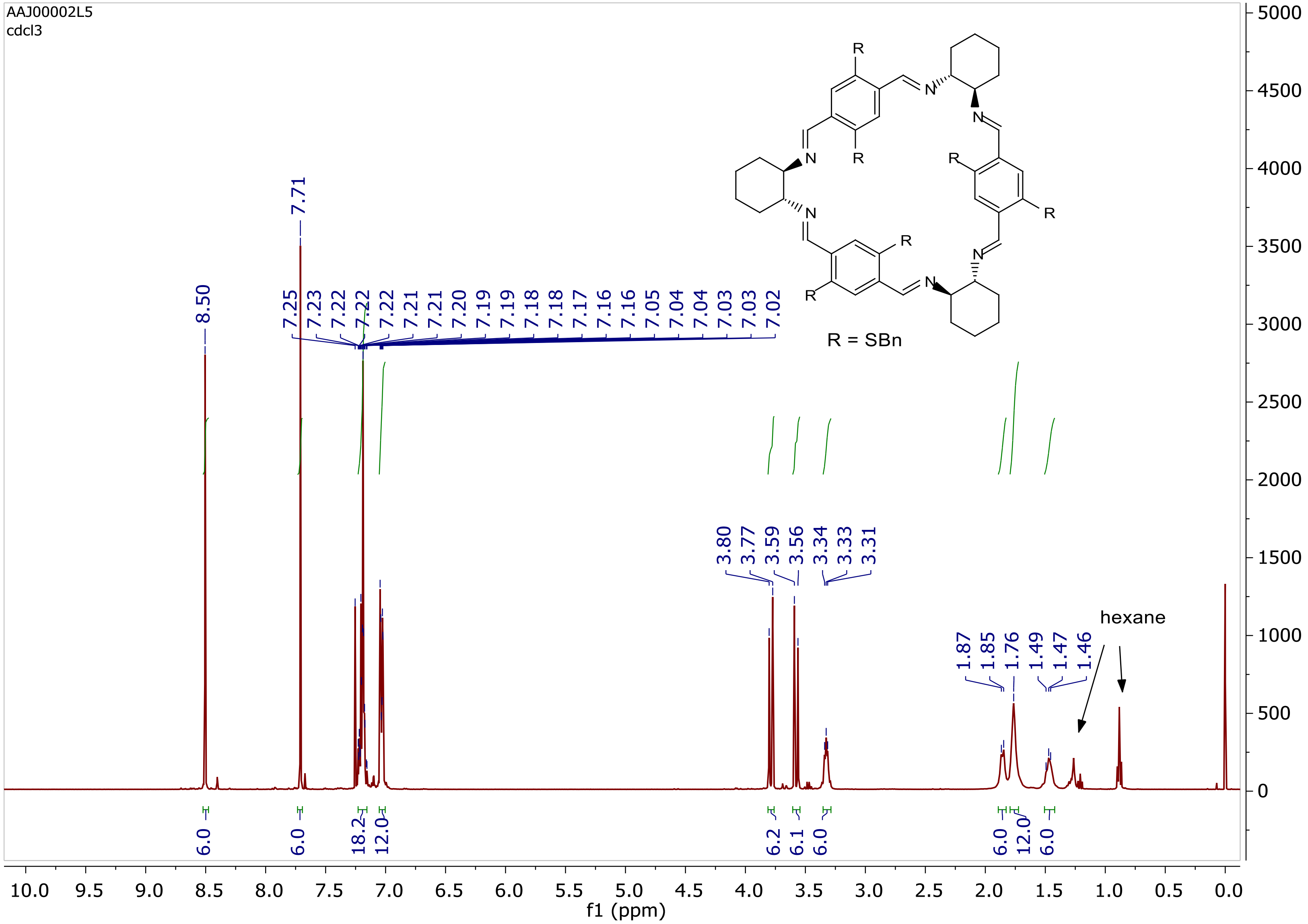

Figure S147. Copy of ^{1}H NMR spectrum ($CDCl_3$, 400 MHz, RT) of **6a**.

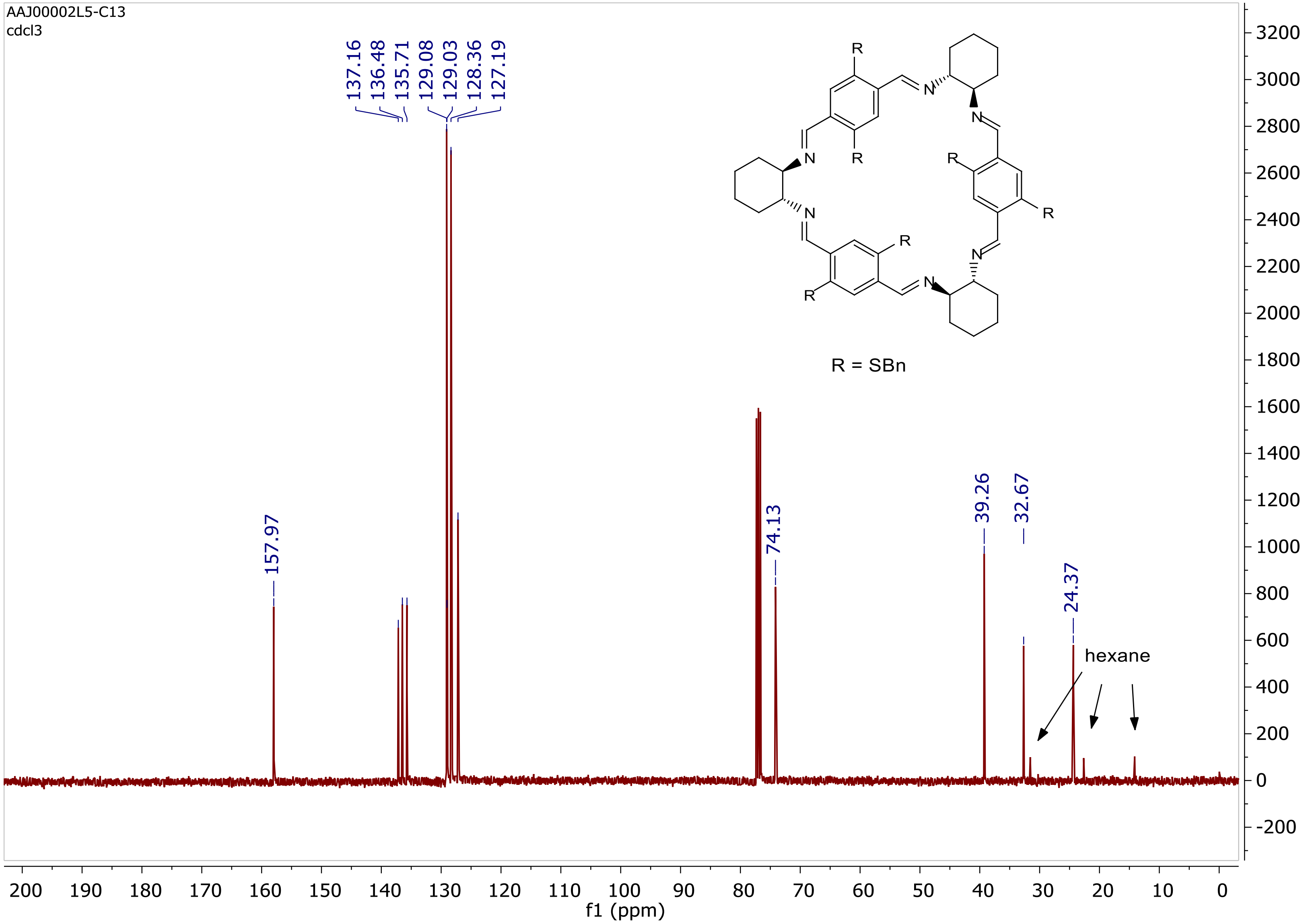

Figure S148. Copy of $^{13}C\{^{1}H\}$ NMR spectrum ($CDCl_3$, 101 MHz, RT) of **6a.**

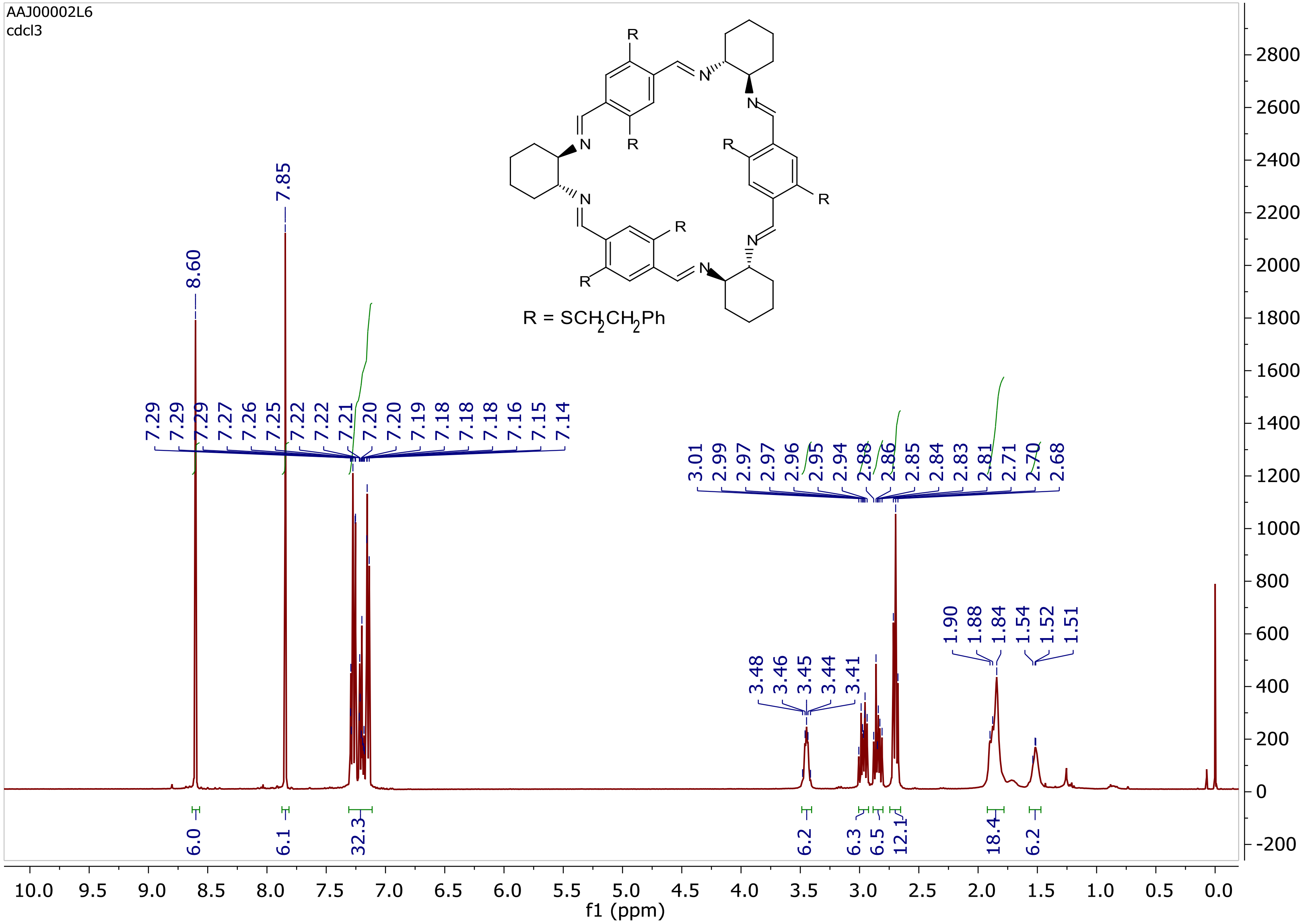

Figure S149. Copy of ^{1}H NMR spectrum ($CDCl_3$, 400 MHz, RT) of **6b**.

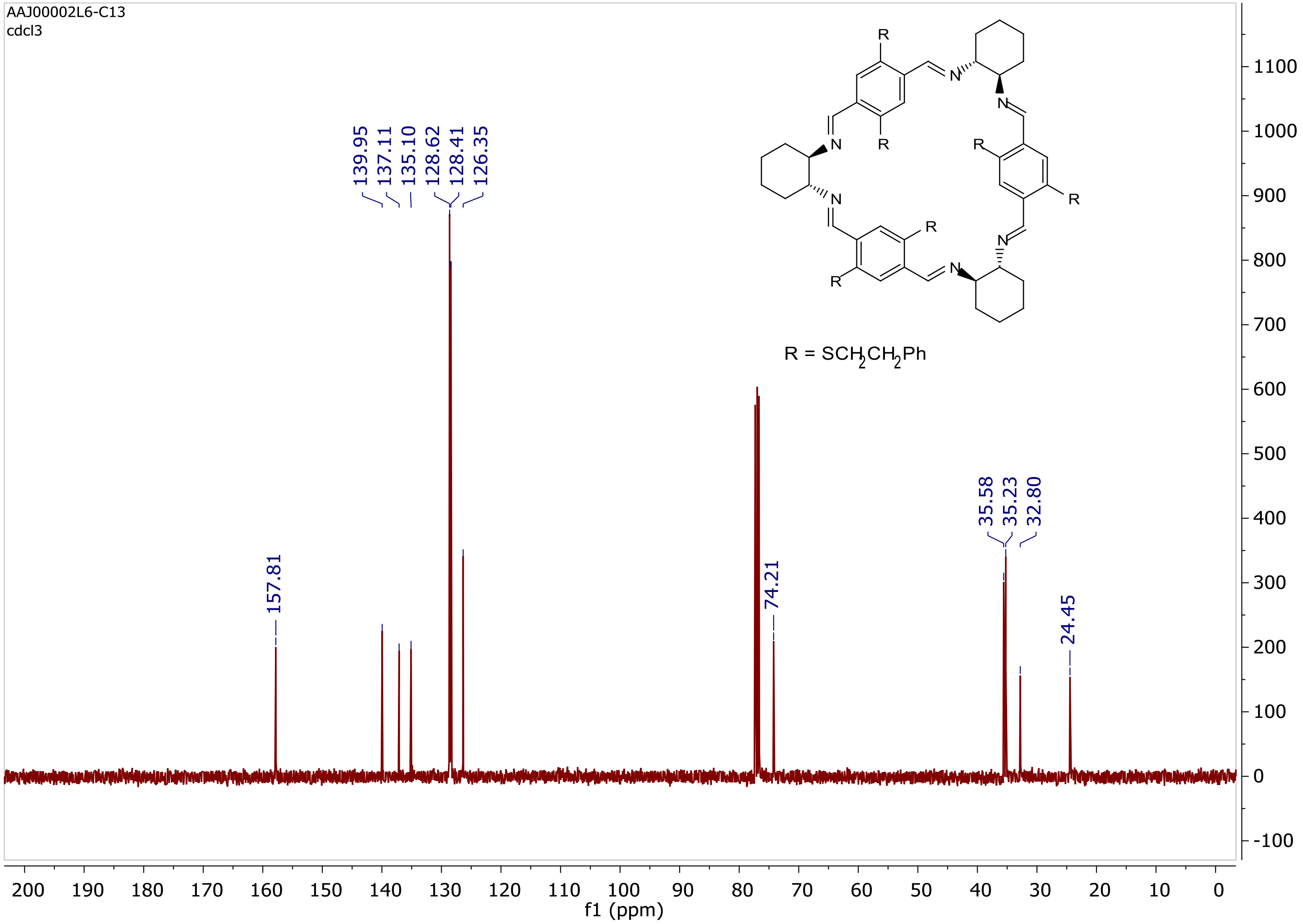

Figure S150. Copy of $^{13}C\{^{1}H\}$ NMR spectrum ($CDCl_3$, 101 MHz, RT) of **6b.**

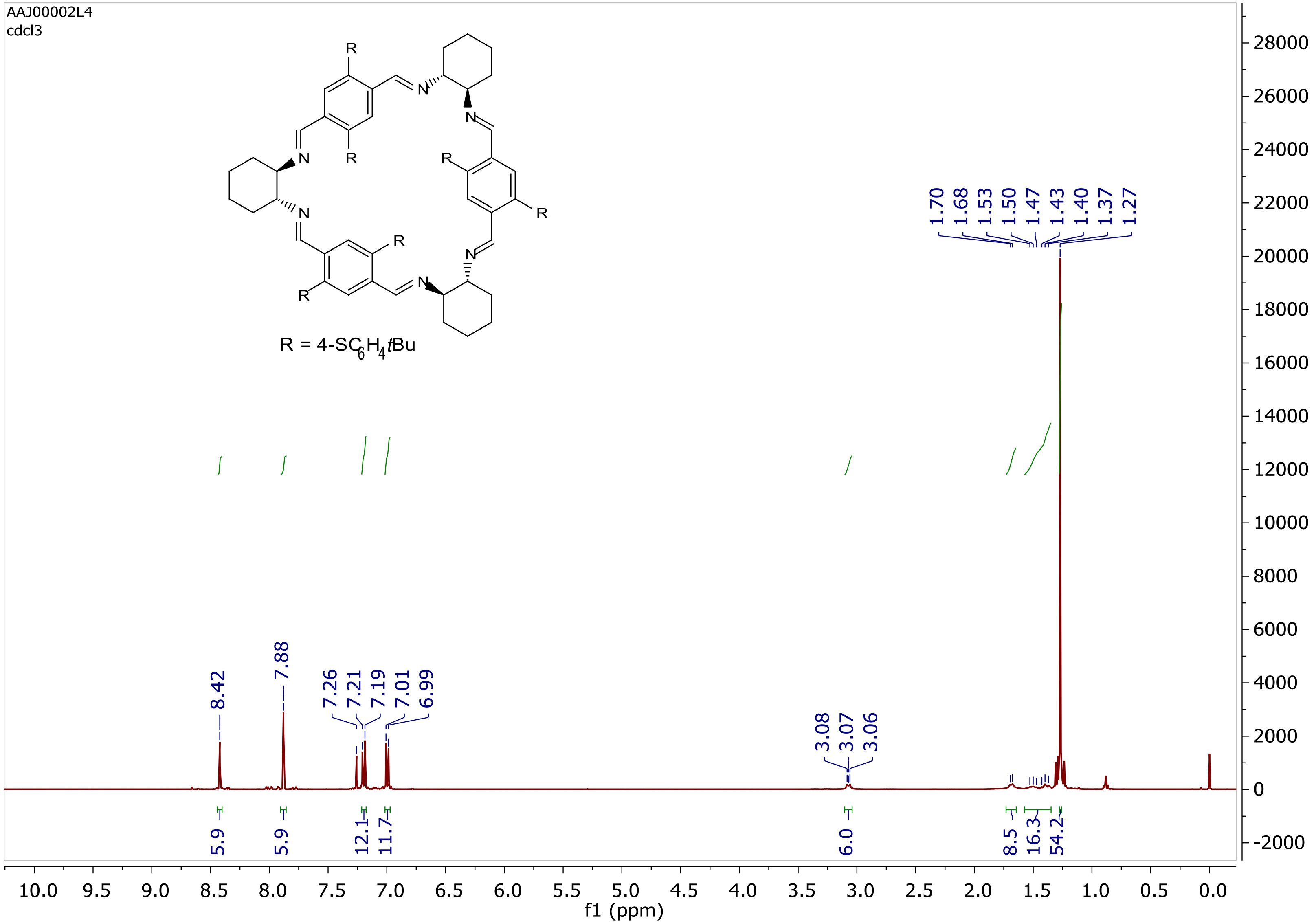

Figure S151. Copy of ^{1}H NMR spectrum ($CDCl_3$, 400 MHz, RT) of **6c**.

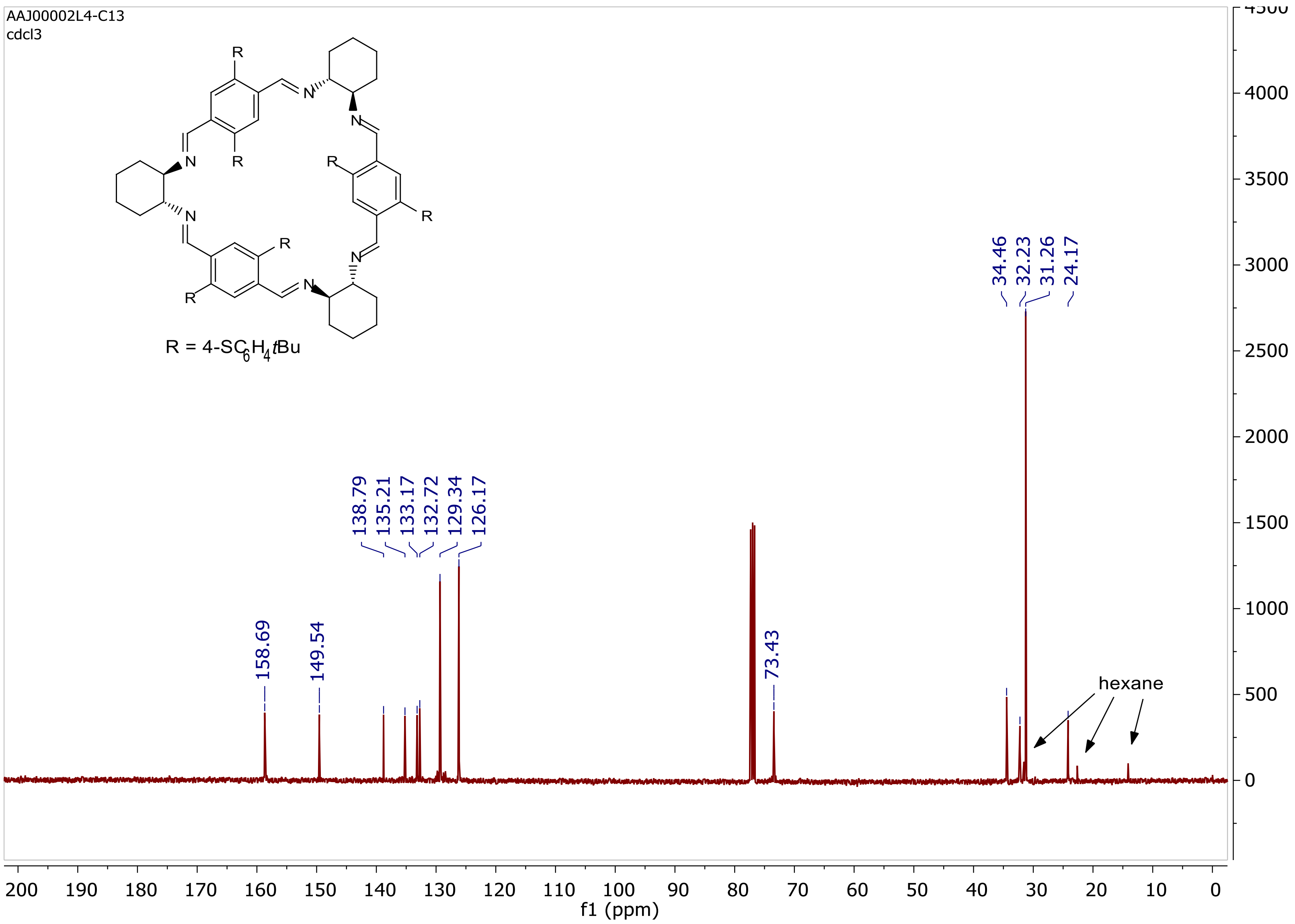

Figure S152. Copy of ^{1}H NMR spectrum ($CDCl_3$, 600 MHz, RT) of **6c**.

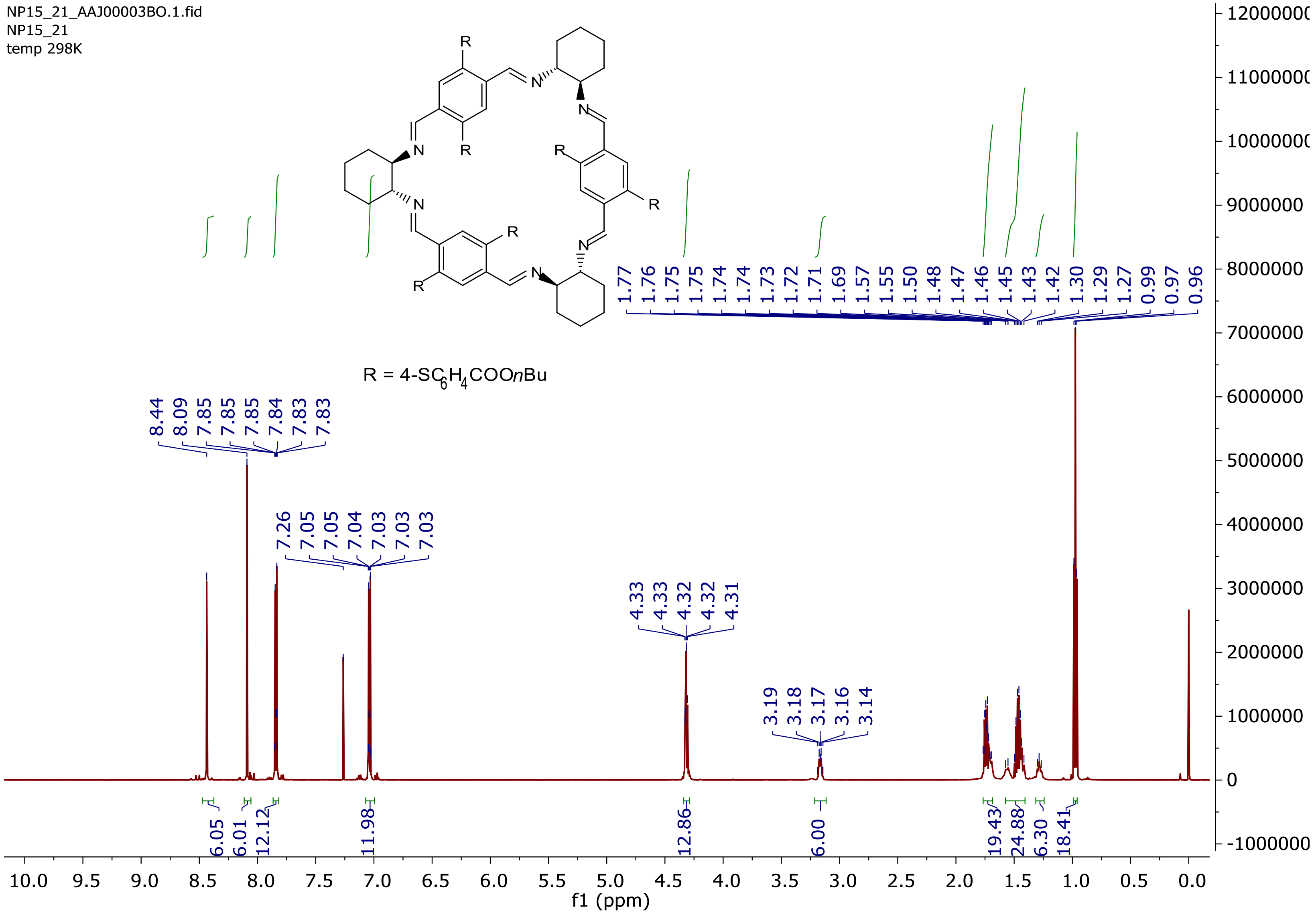

Figure S153. Copy of ^{1}H NMR spectrum ($CDCl_3$, 600 MHz, RT) of **6d**.

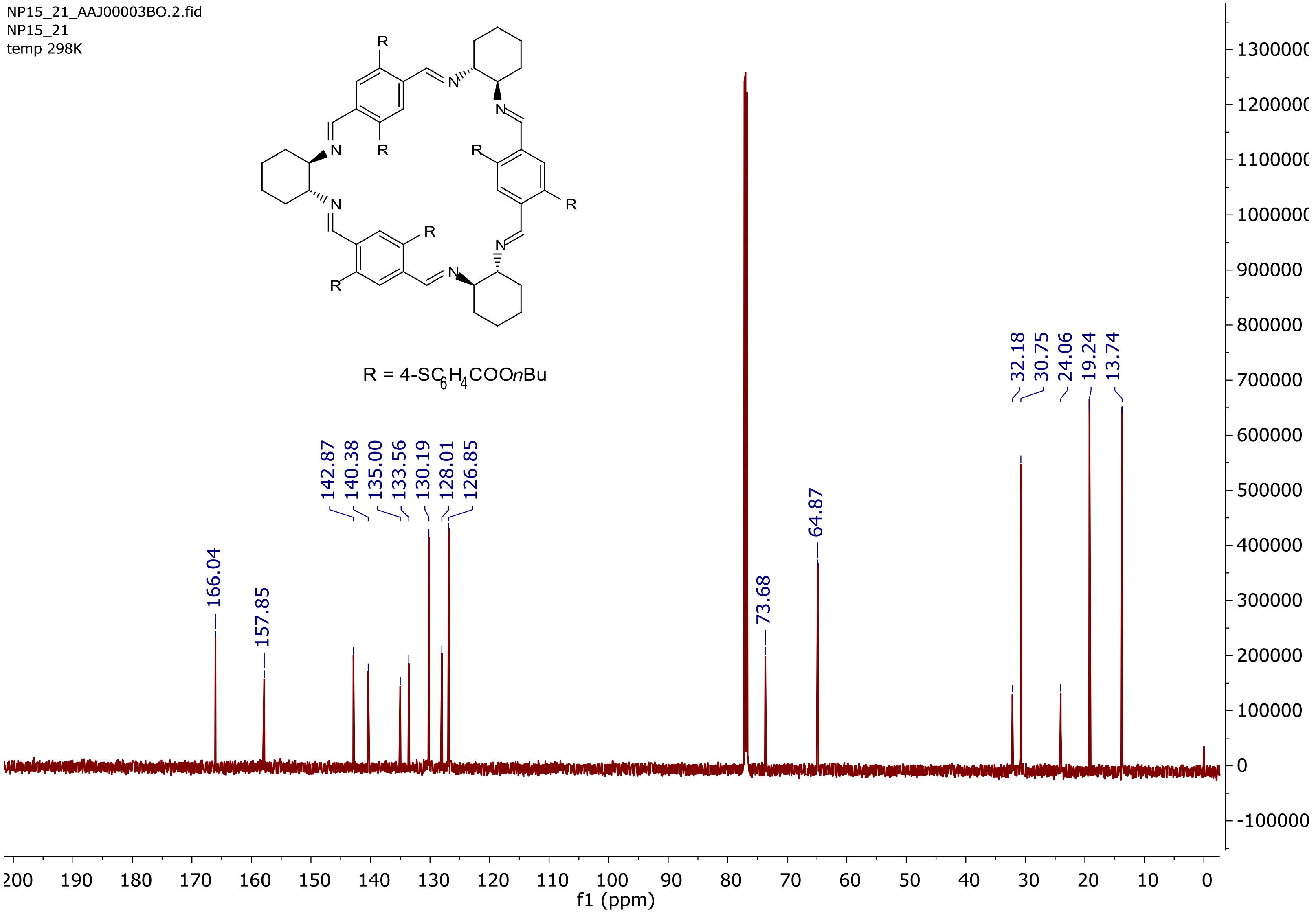

Figure S154. Copy of $^{13}C\{^{1}H\}$ NMR spectrum ($CDCl_3$, 151 MHz, RT) of **6d.**

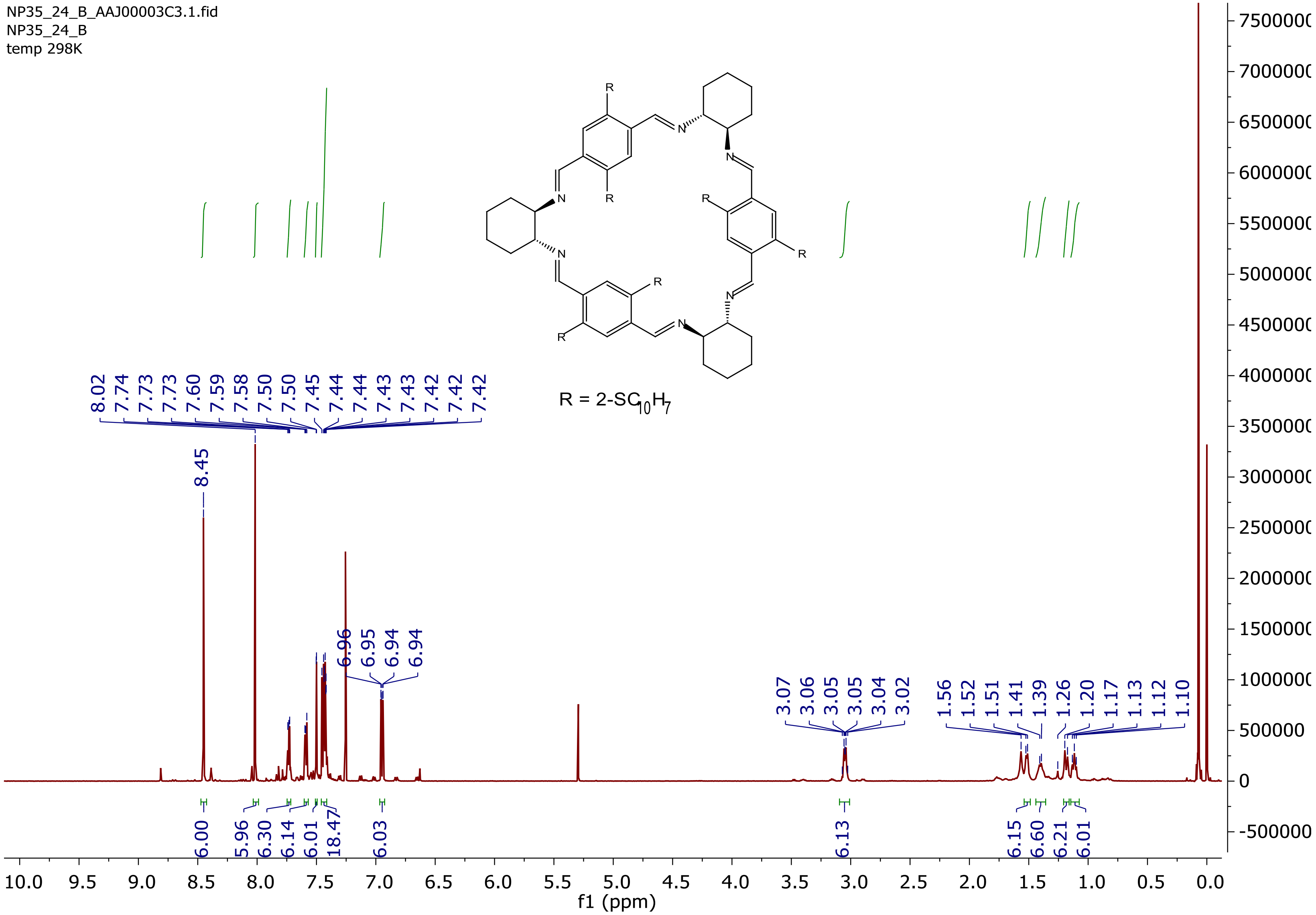

Figure S155. Copy of $^{13}C\{^{1}H\}$ NMR spectrum ($CDCl_3$, 151 MHz, RT) of **6e.**

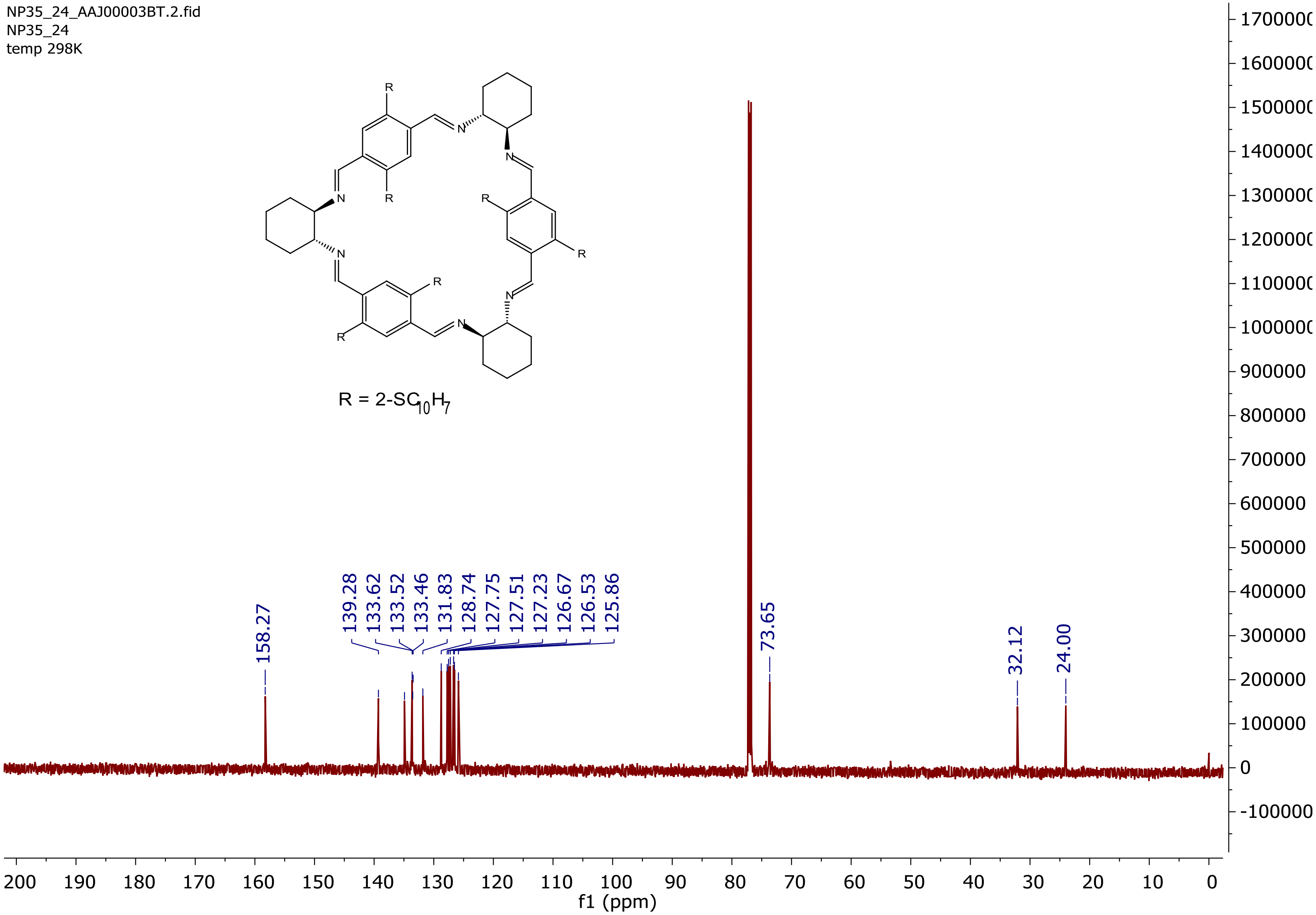

Figure S156. Copy of $^{13}C\{^{1}H\}$ NMR spectrum ($CDCl_3$, 151 MHz, RT) of **6e.**

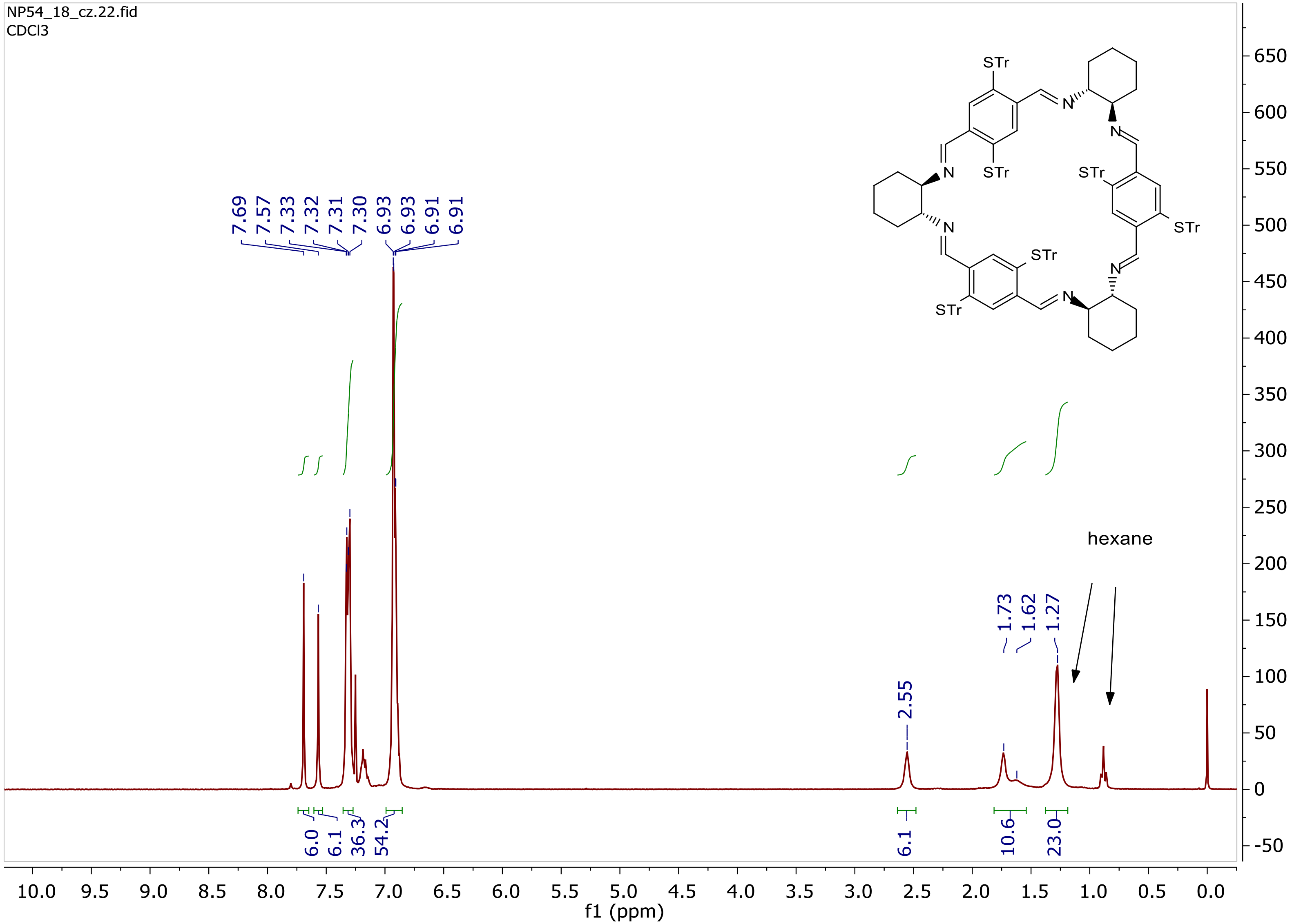

Figure S157. Copy of ^{1}H NMR spectrum ($CDCl_3$, 300 MHz, RT) of **6f.**

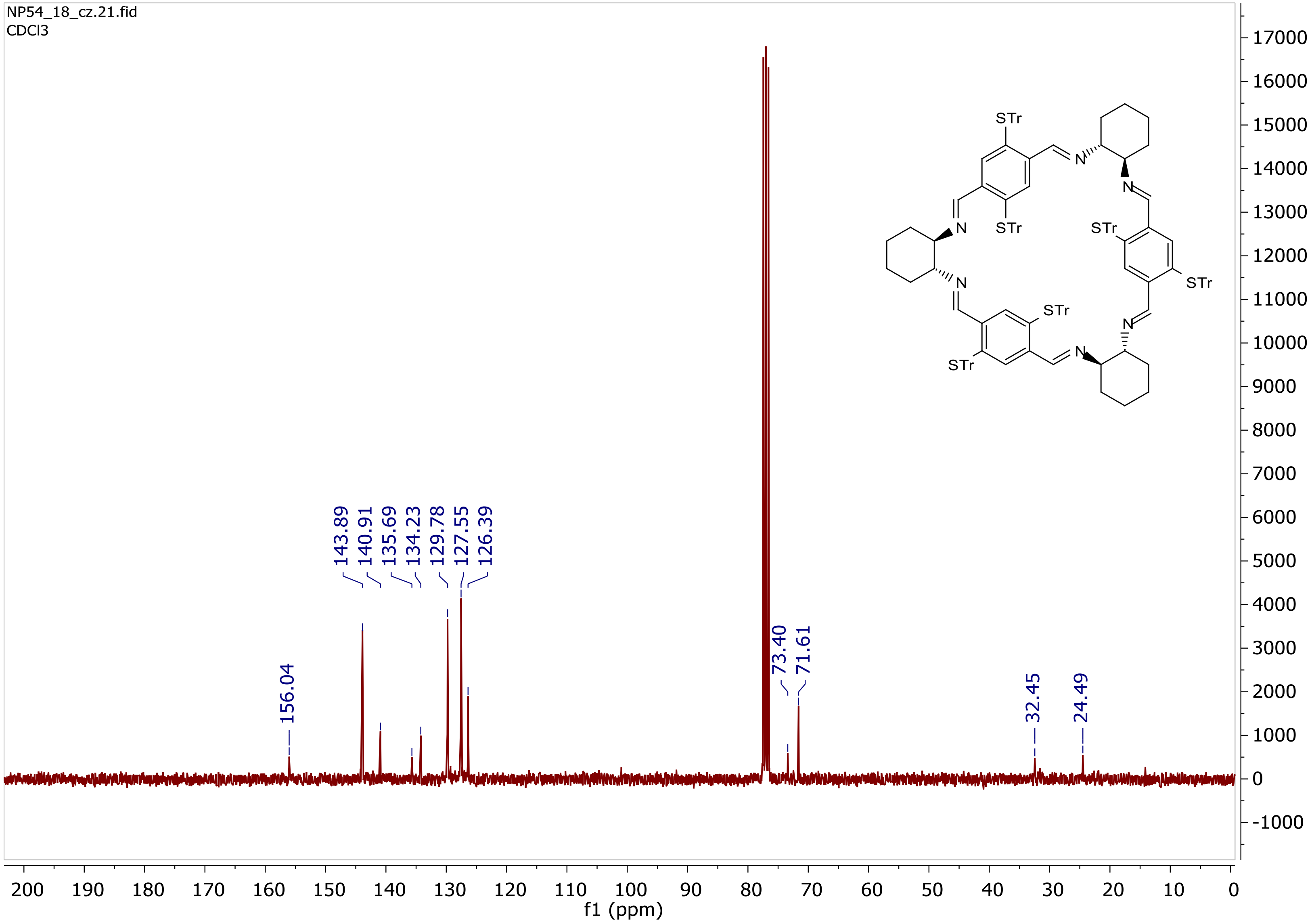

Figure S158. Copy of $^{13}C\{^{1}H\}$ NMR spectrum ($CDCl_3$, 75 MHz, RT) of **6f.**

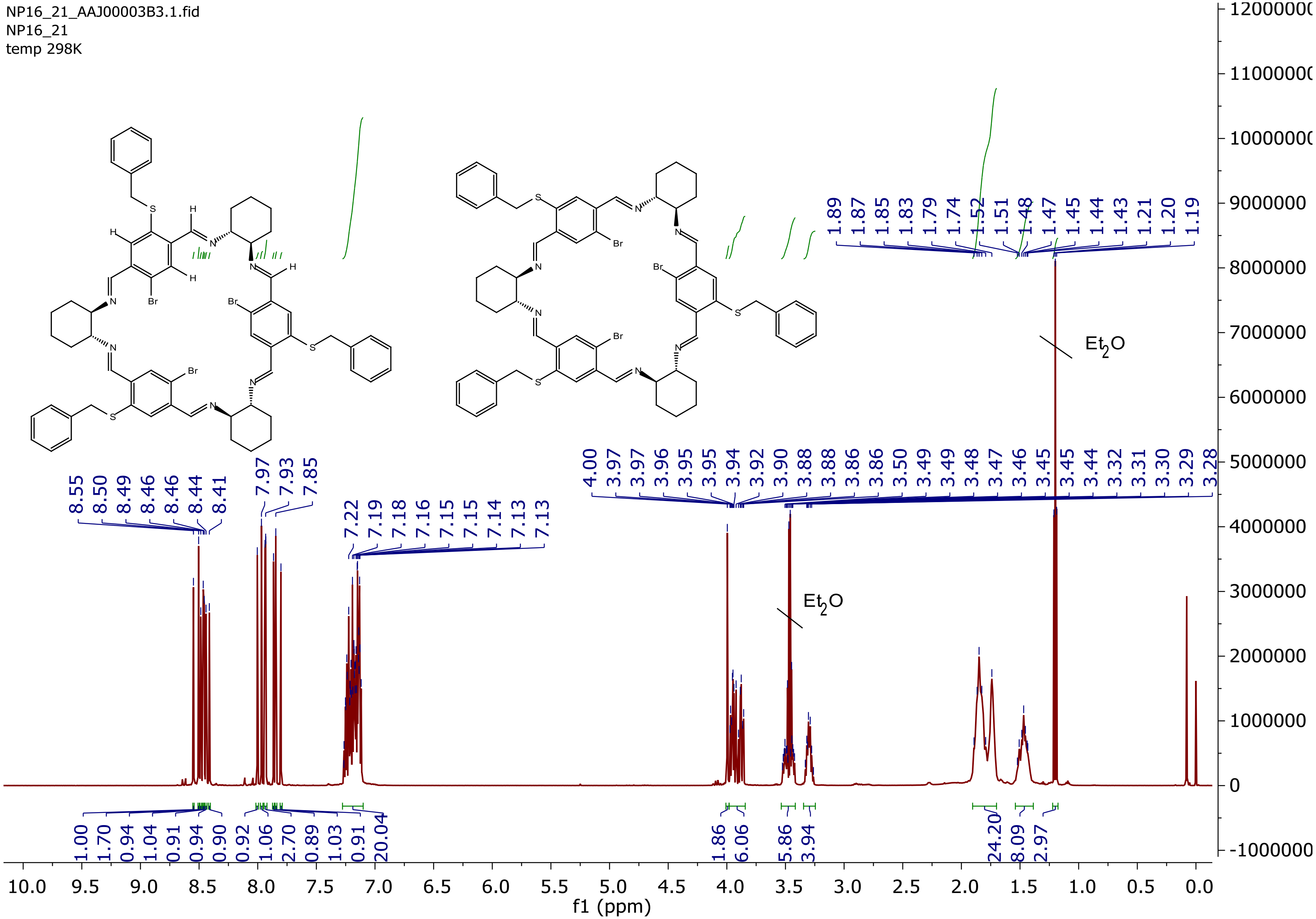

Figure S159. Copy of ^{1}H NMR spectrum ($CDCl_3$, 600 MHz, RT) of **6g.**

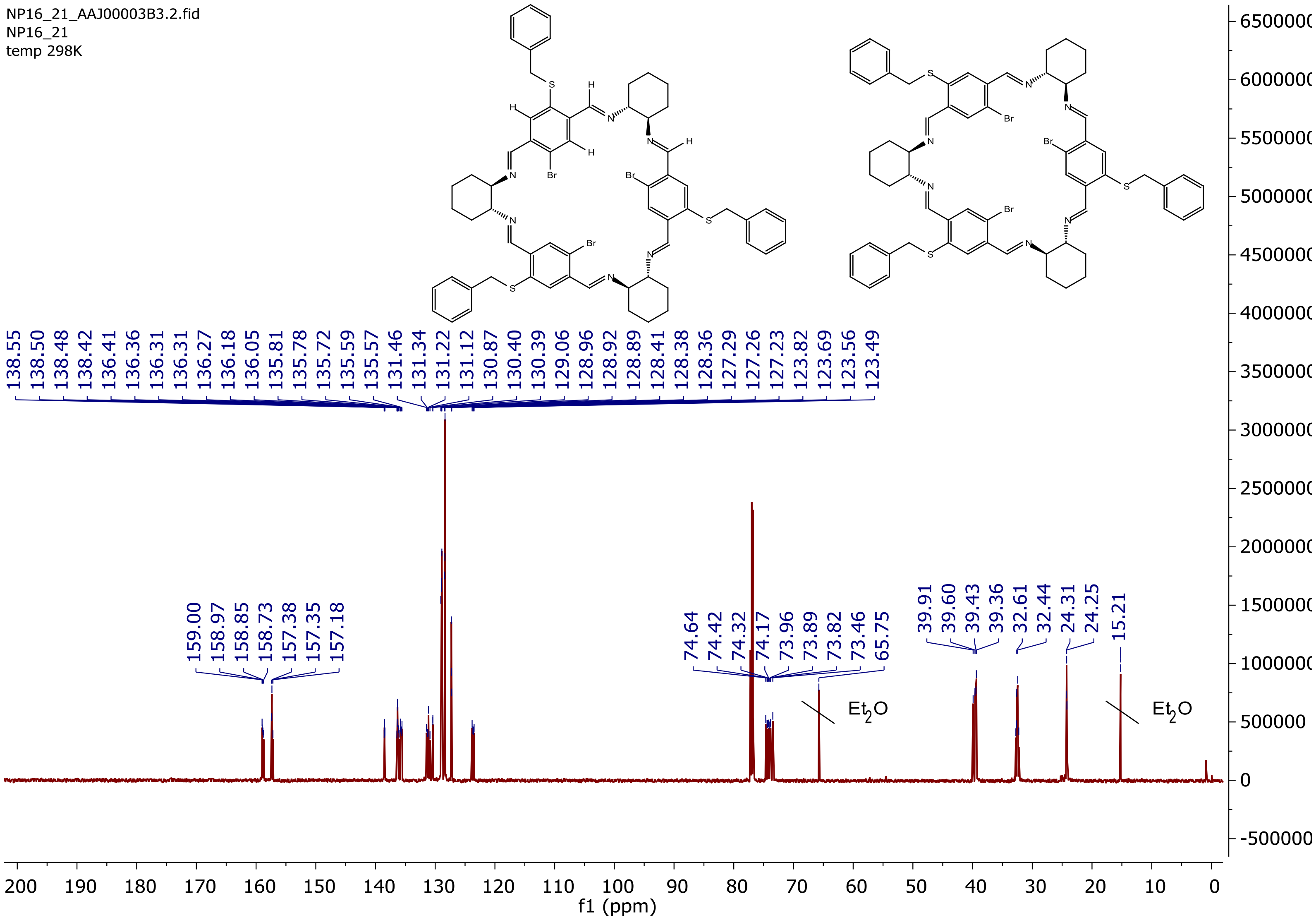

Figure S160. Copy of $^{13}C\{^{1}H\}$ NMR spectrum ($CDCl_3$, 151 MHz, RT) of **6g.**

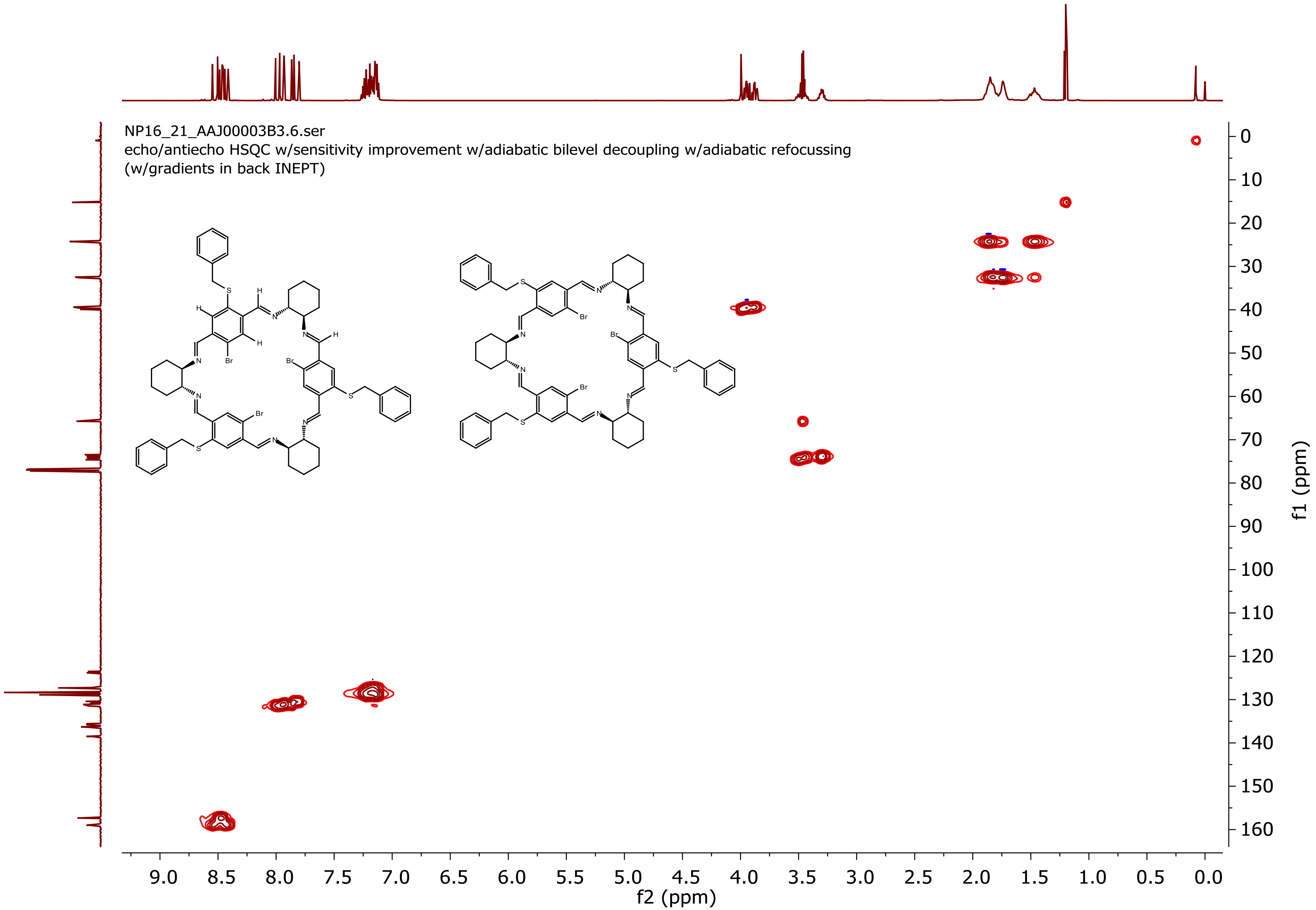

Figure S161. Copy of $^{13}C\{^1H\}$, ^1H HSQC spectrum ($CDCl_3$, 151 MHz, RT) of **6g.**

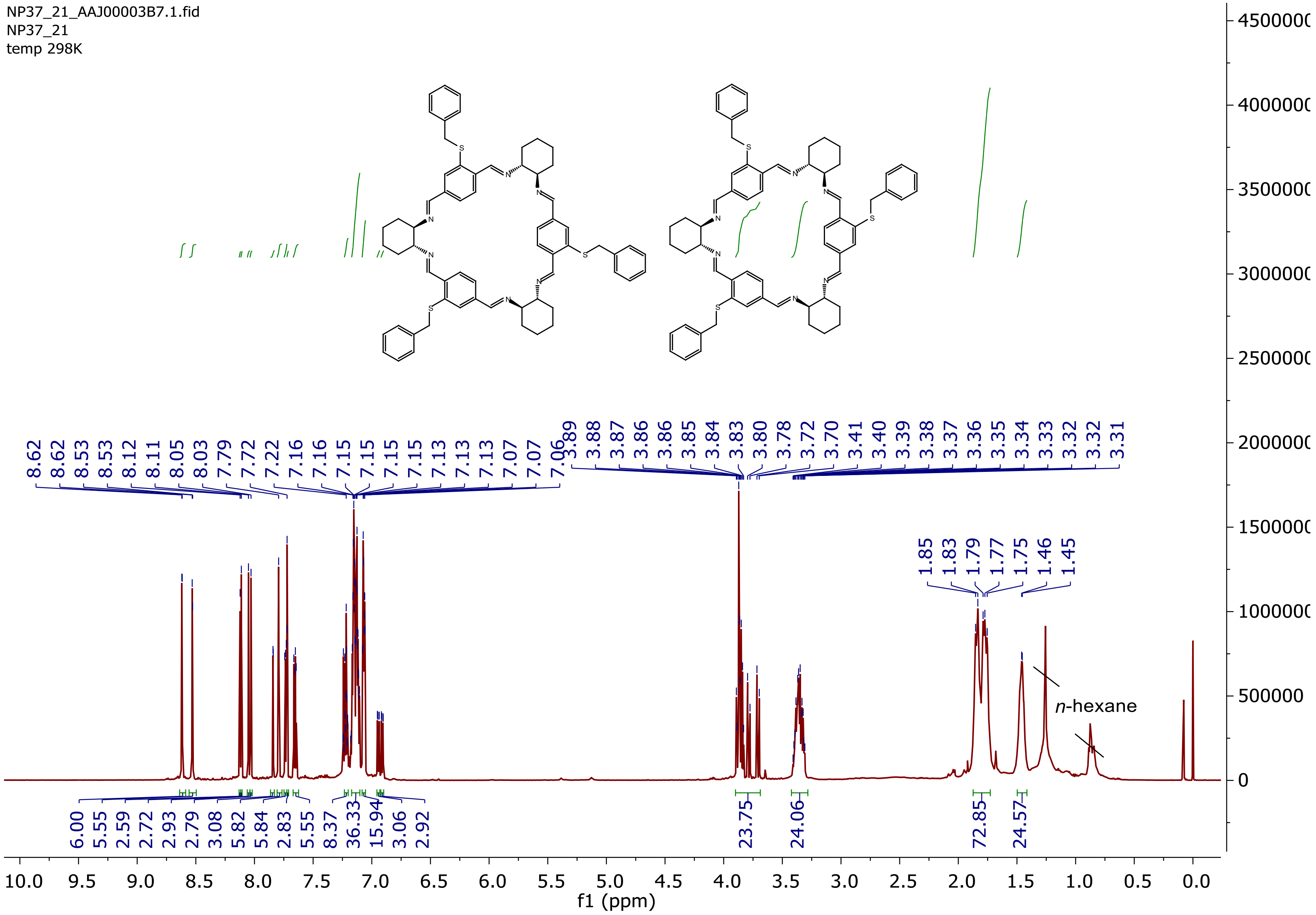

Figure S162. Copy of ^{1}H NMR spectrum ($CDCl_3$, 600 MHz, RT) of **6h.**

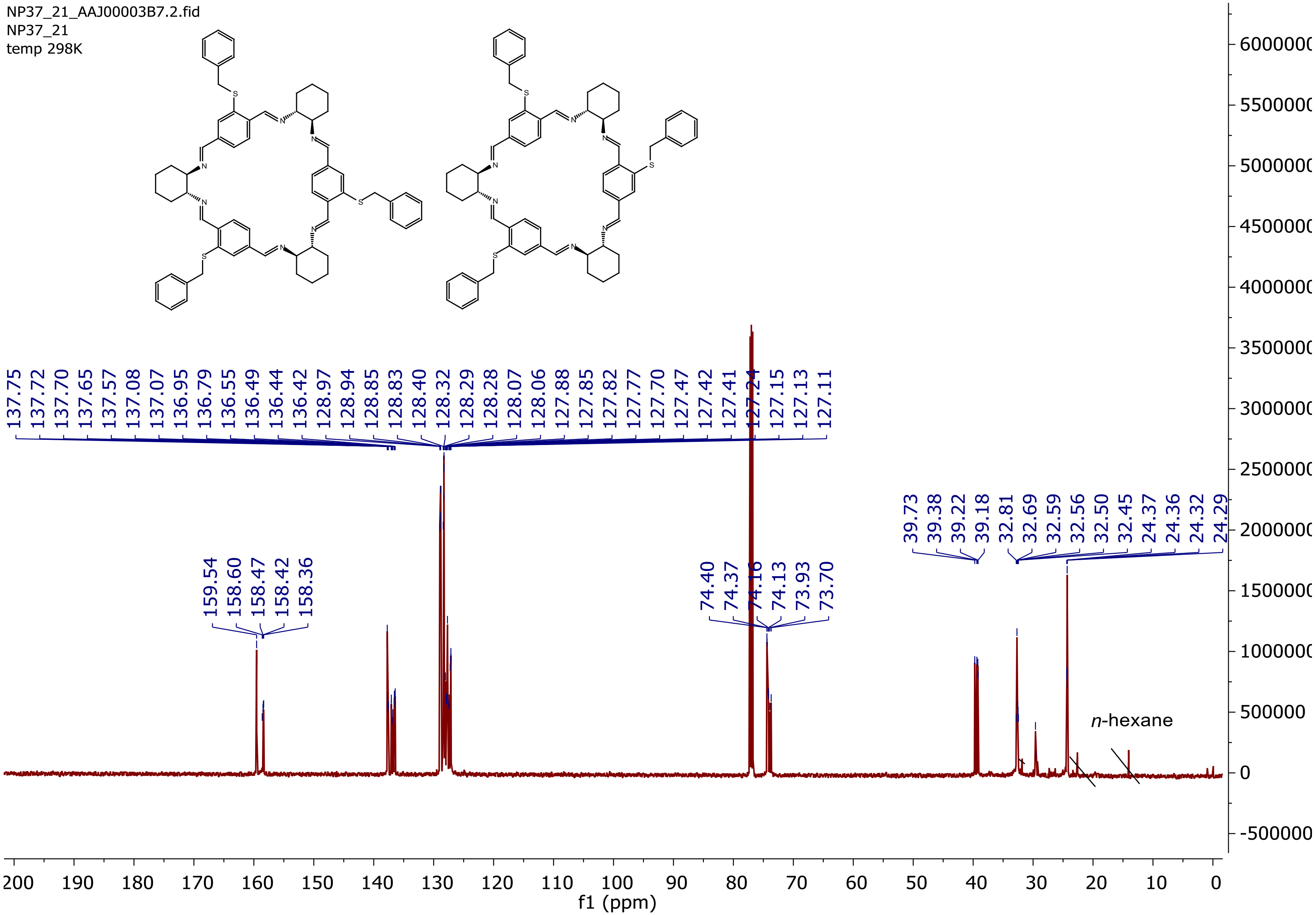

Figure S163. Copy of $^{13}C\{^{1}H\}$ NMR spectrum ($CDCl_3$, 151 MHz, RT) of **6h.**

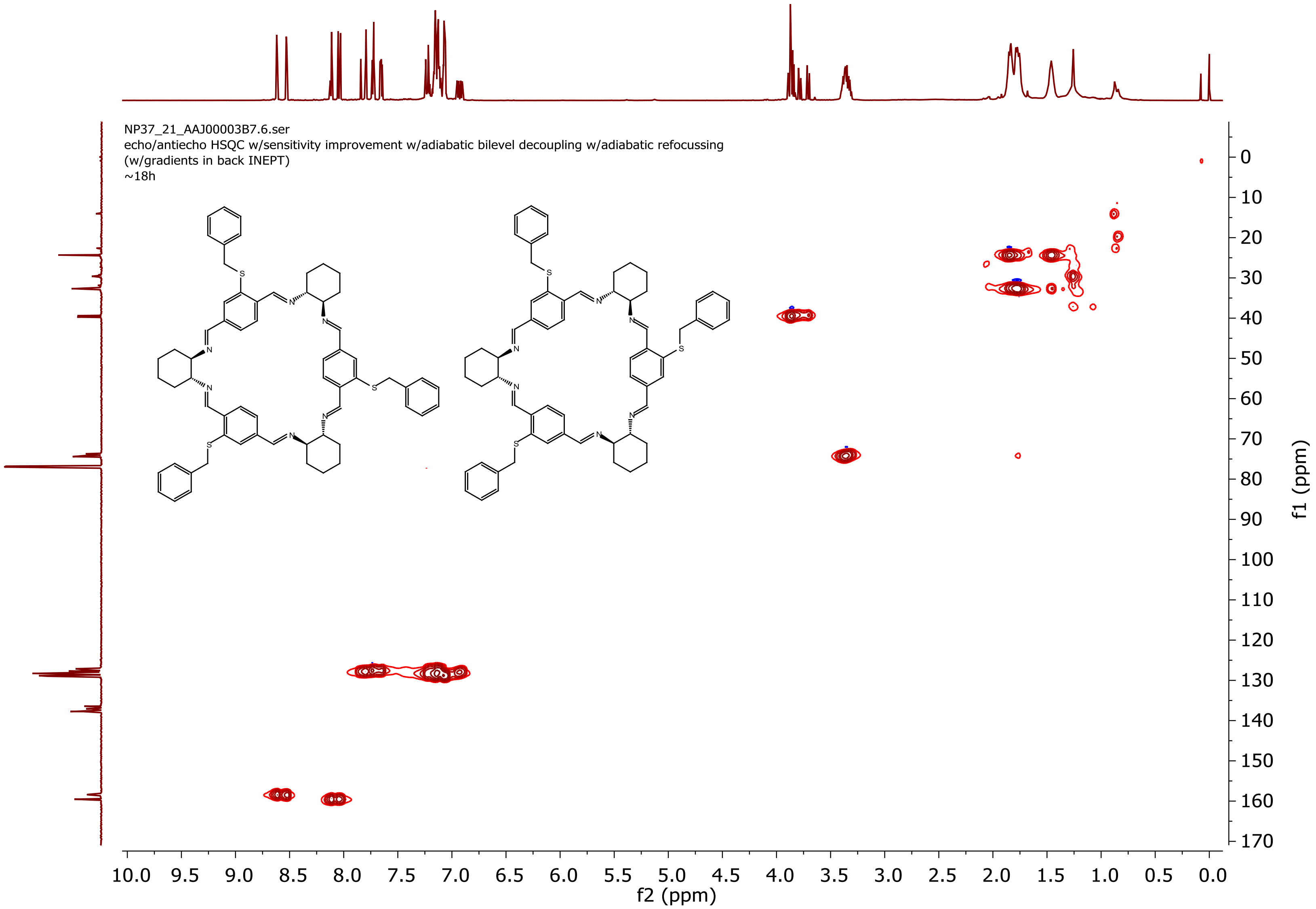

Figure S164. Copy of $^{13}C\{^{1}H\}$, ^{1}H HSQC spectrum ($CDCl_3$, 151 MHz, RT) of **6h.**

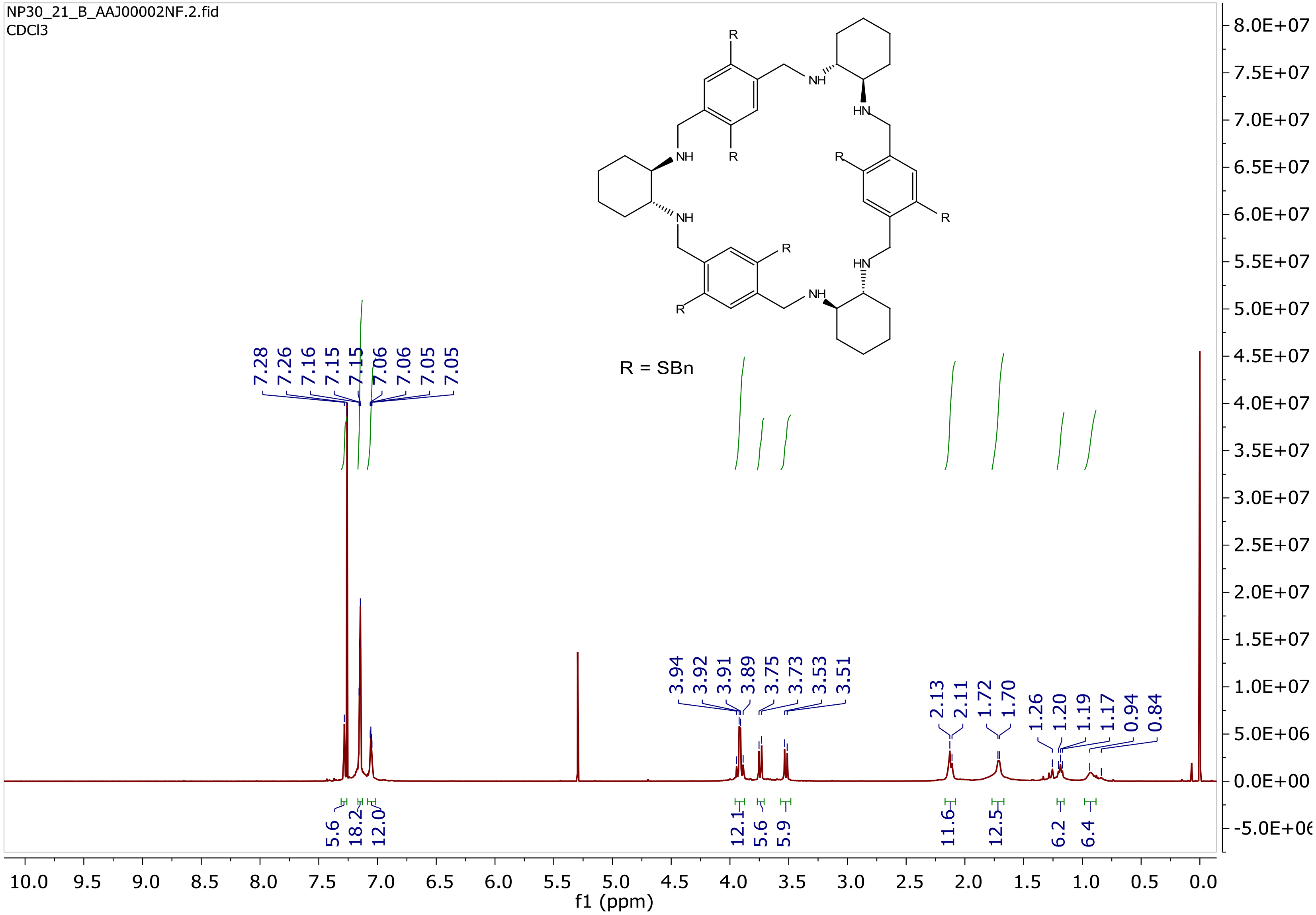

Figure S165. Copy of ^{1}H NMR spectrum ($CDCl_3$, 600 MHz, RT) of **7.**

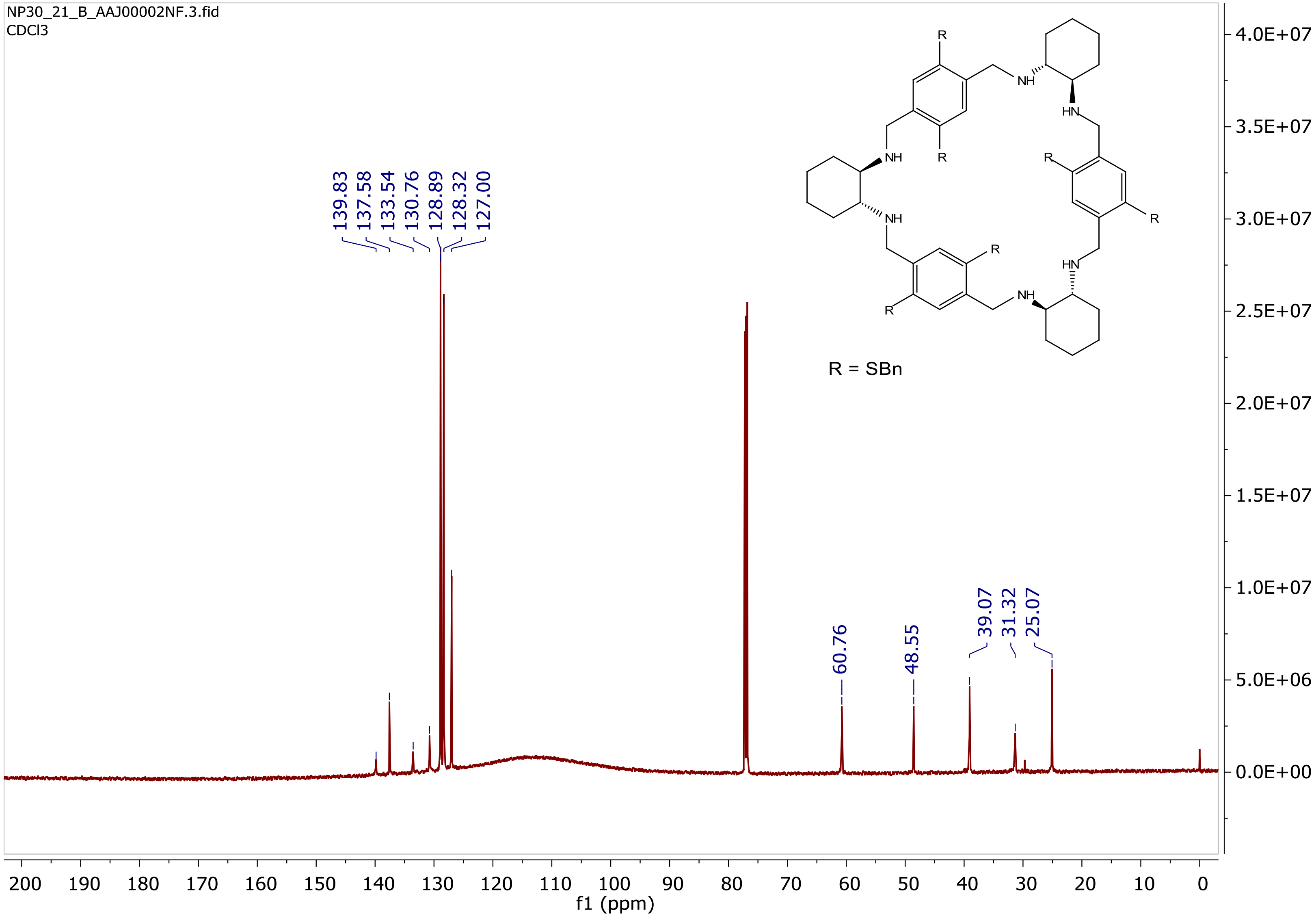

Figure S166. Copy of $^{13}C\{^{1}H\}$ NMR spectrum ($CDCl_3$, 151 MHz, RT) of **7.**

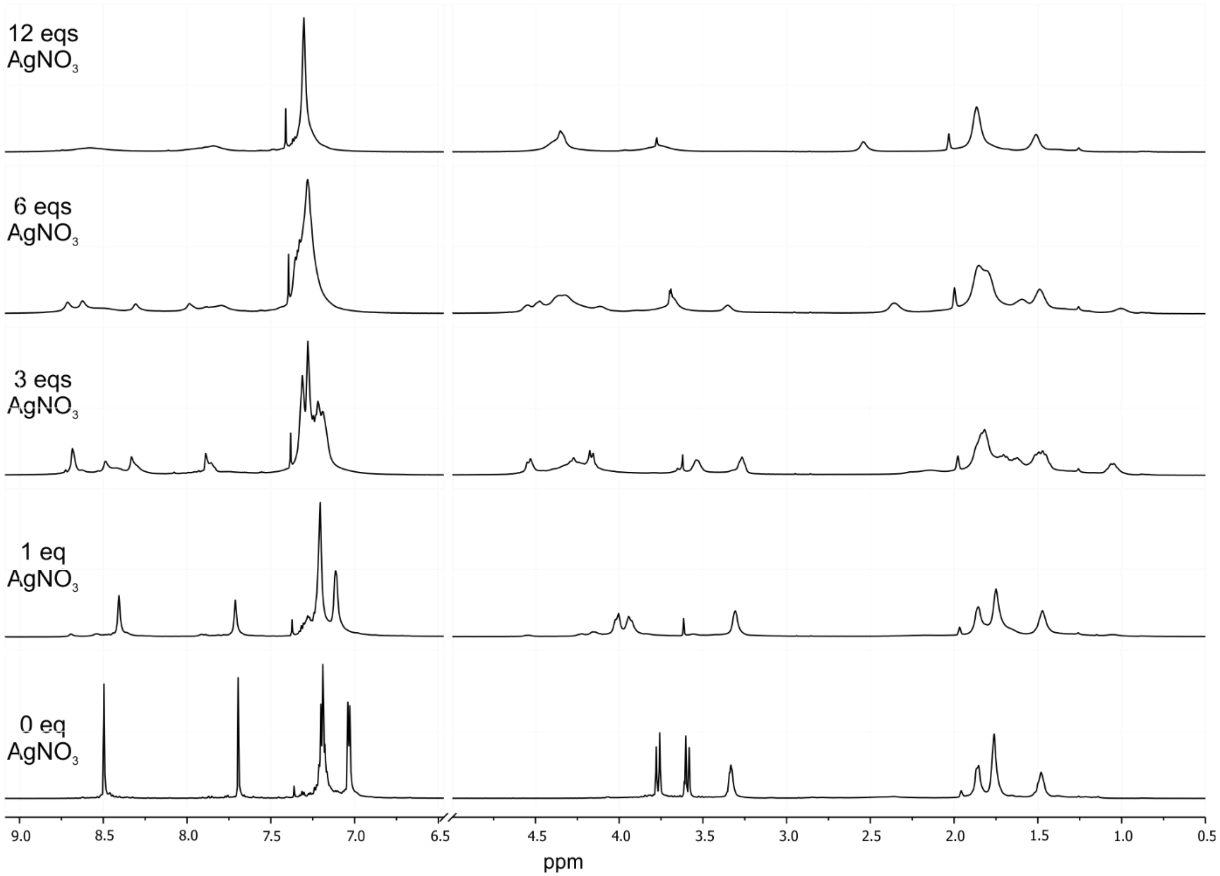

Figure S167. Parts of the ^{1}H NMR spectra measured in $CDCl_3$ and MeCN-*d*3 solution, during the titration of amine **6a** with silver nitrate.

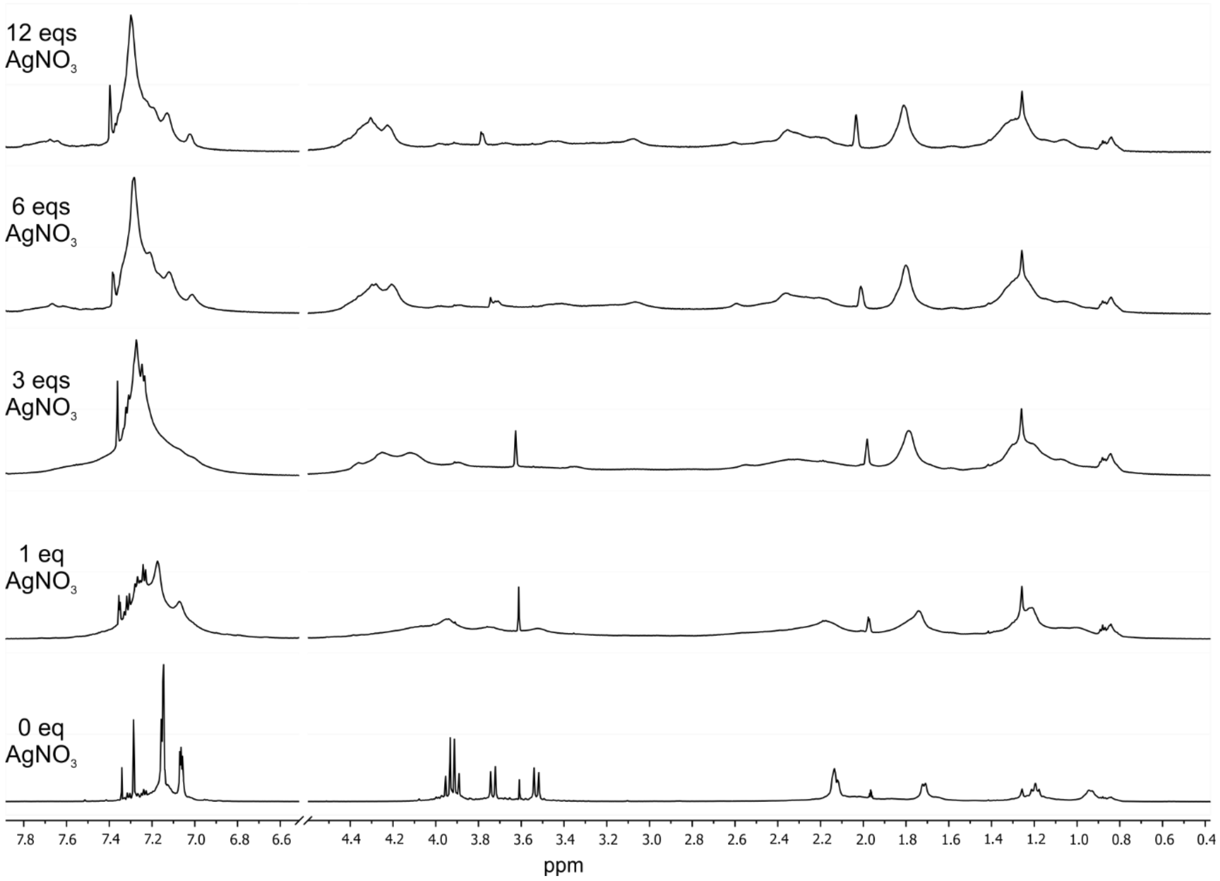

Figure S168. Parts of the ^{1}H NMR spectra measured in $CDCl_3$ and MeCN-*d*3 solution, during the titration of amine **7** with silver nitrate.

6 References

[1] Prusinowska, N., Bardziński, M., Janiak, A., Skowronek, P., Kwit, M. Sterically Crowded Trianglimines—Synthesis, Structure, Solid-State Self-Assembly, and Unexpected Chiroptical Properties. *Chem. Asian J.*, **13**, 2691-2699 (2018).

[2] Scigress, version 2.5; Fujitsu Ltd.: Tokyo, Japan, 2013.

[3] Frisch, M. J. et al. Gaussian 16, Revision C.01, Gaussian, Inc.: Wallingford, CT, USA, 2016.

[4] Becke, A. D. Density-functional thermochemistry. III. The role of exact exchange. *J. Chem. Phys.* **98**, 5648-5652 (1993).

[5] Grimme, S., Ehrlich, S., Goerigk, L. Effect of the damping function in dispersion corrected density functional theory. *J. Comp. Chem*. **32**, 1456-1465 (2011).

[6] Zhao, Y., Truhlar, D. G. The M06 suite of density functionals for main group thermochemistry, thermochemical kinetics, noncovalent interactions, excited states, and transition elements: Two new functionals and systematic testing of four M06-class functional and 12 other functionals. *Theor. Chem. Acc.* **120**, 215–241 (2008).

[7] Petryk, M., Troć, A., Gierczyk, B., Danikiewicz, W., Kwit, M. Dynamic Formation of Noncovalent Calixsalen Aggregates. *Chem. Eur. J*. **21**, 10318-10321 (2015).

[8] Pescitelli, G., Bruhn, T. Good Computational Practice in the Assignment of Absolute Configurations by TDDFT Calculations of ECD Spectra. *Chirality* **28**, 466-474 (2016).

[9] Pescitelli, G., Di Bari, L., Berova, N. Application of electronic circular dichroism in the study of supramolecular systems. *Chem. Soc. Rev.* **43**, 5211-5233 (2014).

[10] Pescitelli, G., Di Bari, L., Berova, N. Conformational aspects in the studies of organic compounds by electronic circular dichroism. *Chem. Soc. Rev.* **40**, 4603-4625 (2011).

[11] Yanai, T., Tew, D., Handy, N. A new hybrid exchange-correlation functional using the Coulomb-attenuating method (CAM-B3LYP). *Chem. Phys. Lett.* **393**, 51–57 (2004).

[12] Chai, J.-D., Head-Gordon, M. Long-range corrected hybrid density functionals with damped atom-atom dispersion corrections. *Phys. Chem. Chem. Phys.* **10**, 6615-6620 (2008).

[13] Prusinowska, N., Szymkowiak, J., Kwit, M. Unravelling Structural Dynamics, Supramolecular Behavior and Chiroptical Properties of Enantiomerically Pure Macrocyclic Tertiary Ureas and Thioureas. *J. Org. Chem.* **88**, 285–299 (2023)..

[12] CrysAlis PRO 1.171.41.110a, Rigaku Oxford Diffraction, 2021.

[13] Sheldrick, G. M. SHELXT - Integrated space-group and crystal-structure determination. *Acta Crystallogr.* **A71**, 3-8 (2015).

[14] Sheldrick, G. M. Crystal structure refinement with SHELXL. *Acta Crystallogr.* **C71**, 3-8 (2015).

[15] Parsons, S., Flack, H. D., Wagner, T. Use of intensity quotients and differences in absolute structure refinement. *Acta Crystallogr*. **B69**, 249-259 (2013).

[16] Dolomanov, O.V., Bourhis, L.J., Gildea, R.J, Howard, J.A.K., Puschmann, H. OLEX2: a complete structure solution, refinement and analysis program. *J. Appl. Cryst.* 42, 339-341 (2009).

[17] Macrae, C. F., Sovago, I., Cottrell, S. J., Galek, P. T. A., McCabe, P., Pidcock, E., Platings, M., Shields, G. P., Stevens, J. S., Towler, M., Wood, P. A. Mercury 4.0: from visualization to analysis, design and prediction. *J. Appl. Cryst.* **53**, 226-235 (2020).
